# Statistical modelling of CG interdistance across multiple organisms

**DOI:** 10.1186/s12859-018-2303-2

**Published:** 2018-10-15

**Authors:** Merlotti A., Faria do Valle I., Castellani G., Remondini D.

**Affiliations:** 1 Department of Physics and Astronomy, University of Bologna, Bologna, Italy; 2 Center for Complex Network Research and Physics Department, Northeastern University, Boston, MA USA

**Keywords:** CG dinucleotide, Interdistance distribution, Distribution fitting, Classification

## Abstract

**Background:**

Statistical approaches to genetic sequences have revealed helpful to gain deeper insight into biological and structural functionalities, using ideas coming from information theory and stochastic modelling of symbolic sequences. In particular, previous analyses on CG dinucleotide position along the genome allowed to highlight its epigenetic role in DNA methylation, showing a different distribution tail as compared to other dinucleotides.

In this paper we extend the analysis to the whole CG distance distribution over a selected set of higher-order organisms. Then we apply the best fitting probability density function to a large range of organisms (>4400) of different complexity (from bacteria to mammals) and we characterize some emerging global features.

**Results:**

We find that the Gamma distribution is optimal for the selected subset as compared to a group of several distributions, chosen for their physical meaning or because recently used in literature for similar studies. The parameters of this distribution, when applied to our larger set of organisms, allows to highlight some biologically relavant features for the considered organism classes, that can be useful also for classification purposes.

**Conclusions:**

The quantification of statistical properties of CG dinucleotide positioning along the genome is confirmed as a useful tool to characterize broad classes of organisms, spanning the whole range of biological complexity.

**Electronic supplementary material:**

The online version of this article (10.1186/s12859-018-2303-2) contains supplementary material, which is available to authorized users.

## Background

Recent studies revealed that dinucleotide interdistances can be a powerful tool for detecting DNA properties [1, 2], such as the identification of CpG islands [3] and the characterization of epigenomic regulation through methylation [4, 5]. In a previous paper [4], we higlighted a peculiar feature of mammals CG dinucleotides: the tail of CG interdistance distributions showed an exponential decay, at difference with non CG’s which had a heavier tail more similar to a power law. This might be due to the specific role that CGs play inside mammals genomes, since they are the preferential sites of methylation, a fundamental epigenetic mechanism involved in gene regulation [6–10] and structural conformation of chromatine [11, 12]. In light of these preliminary observations, we believe that a characterization of the complete CG distribution would provide a better comprehension of their role inside genomes of all organisms, with the idea that similar functionalities should share similar statistical properties. Moreover, the identified distribution can be the basis for hypothesizing specific physical models to describe the observed DNA sequence characteristics.

We previously noticed that the distinction between CG and non-CG interdistance distributions is less sharp in non-mammal organisms, by considering a set of 21 genomes, belonging to 10 mammal and 11 non-mammal organisms [4]. We have now extended the study to CG interdistance distributions from 4425 genomes, belonging to a wide range of organism categories (bacteria, protozoa, plants, fungi, invertebrates, mammal and non-mammal vertebrates) in order to better understand the heterogenous scenario found among non-mammals and to obtain a global picture associated to this particular feature.

## Methods

### Data

The organism DNA sequences were downloaded from GenBank NCBI database [13]. We defined a subset of organisms, namely the DNA sequences of 9 mammal model organisms: *Bos taurus*, *Canis familiaris*, *Equus caballus*, *Homo sapiens*, *Macaca mulatta*, *Mus musculus*, *Ornithorhynchus anatinus*, *Pan troglodytes* and *Rattus norvegicus*, to test the goodness of fit of the chosen probability density functions, since in a previous work [4] they showed very homogeneous characteristics in terms of CG distribution.

An extended analysis was then performed on a dataset composed of 4425 genomes (see Additional file 1 for a detailed list on organisms and measured parameters), selected among 7 of the 11 categories represented on the NCBI database: bacteria, fungi, invertebrates, plants, protozoa, mammal vertebrates and non-mammal vertebrates (see Table 1). In order to ensure minimal quality criteria on the reconstructed genome sequences, we chose to study only fasta files at chromosome and scaffold levels, discarding those for which only contigs were available.

**Table 1:** Number and size of genome assemblies downloaded from GenBank database, divided into categories

Category	Number of genomes	Size
Vertebrates non-mammals	200	210 Gb
Vertebrates mammals	219	525 Gb
Plants	297	288 Gb
Protozoa	348	17 Gb
Invertebrates	507	168 Gb
Bacteria	1251	5 Gb
Fungi	1603	44 Gb

### Computation of CG interdistance distributions

The first step of our analysis consisted in the estimation of CG interdistance relative frequency distributions $\hat p(\tau)$ in the selected organism set. We pre-processed the data by extracting the longest sequence from each genome, except sex chromosomes [4], and removing the unknown bases, identified with the “N” symbol in the fasta files. This operation did not affect the computation of $\hat p(\tau)$, because the ratio of N inside the sequences was in general low (see Table 2 and Additional file 1) and they were mainly located contiguously at the centromere and telomere regions, thus producing only a very small number of large distances (that could eventually be easily removed from the analysis). Subsequently we found the positions *x*_*j*_ of each CG dinucleotide inside the sequence, and we calculated the distance between two consecutive CG as *τ*_*j*_=*x*_*j*+1_−*x*_*j*_; finally, for each distance value *τ*, we counted its abundance along the sequence and estimated its relative frequency $\hat {p}(\tau)$, as described in Eq. 1. In this way we obtained a relative frequency distribution that we called CG interdistance distribution.

1 $$ \hat{p}(\tau) = \frac{\#\{ j| \tau_{j} = \tau \}}{\# \{ \tau_{j} \}}  $$

**Table 2:** Percentage of unkown bases N inside each analyzed sequence of the first set of organisms

Organism	Sequence	N (%)
Bos taurus	chromosome 1	0.7
Canis familiaris	chromosome 1	0.5
Equus caballus	chromosome 1	1.2
Homo sapiens	chromosome 1	7.4
Macaca mulatta	chromosome 1	6.5
Mus musculus	chromosome 1	7.9
Ornithorhynchus anatinus	chromosome 3	6.4
Pan troglodytes	chromosome 1	2.1
Rattus norvegicus	chromosome 1	5.2

### Choice of best distribution

In order to find a complete characterization of mammal CG distribution, we firstly represented $\hat p(\tau)$ for the 9 mammal model organisms in semilogarithmic scale. In this way, we immediately recognized an exponentially decaying trend in the tails (not shown, see Supplementary Materials in [4]), which led us to consider the following functions: exponential and double exponential distributions, which can be associated to physical processes respectively governed by a single and a double characteristic scale (that would correspond to characteristic CG distances along the genome); stretched exponential and gamma distributions, which are related to physical processes involving both a characteristic scale and a power-law trend [14–23]. We also took into account the q-exponential distribution, as suggested by a recent work [5] that studied CG interdistance distributions on a small interval of about 0−300 dinucleotide distance values for human genome. In our study we consider the whole distance distribution up to about 2000 nucleotides for the same organism, and of the same order of magnitude for the other higher-order organisms of the considered subset. The proposed distributions were fitted to the data by using a non-linear least square method (*fit* function, Mathworks Matlab software).

2 $$ p(\tau) = ae^{-\tau/b}  $$

3 $$ p(\tau) = ae^{-\tau/b}+ce^{-\tau/d}  $$

4 $$ p(\tau) = ce^{-\tau^{a}/b}  $$

5 $$ p(\tau) = [1+(1-a)\tau]^{\frac{-1}{(1-a)}}  $$

6 $$ p(\tau) = c\tau^{a-1}e^{-\tau/b}  $$

We noticed that the extreme region of the right tail of our CG distributions adversely affected fit results, due to poor sampling (see Additional file 1 for details), therefore we decided to exclude from the fit procedure all distances beyond the 90th percentile (leaving an interval of distances from 0 up to about 1000−2000 bases in all 9 higher-order organisms). The goodness of fit was initially estimated by *r*^2^ parameter (Eq. 7), defined as:

7 $$ r^{2} = 1 - \frac{SSR}{SST}  $$

where SSR represents the sum of squares of the regression and SST the sum of squares about the mean, also called total sum of squares. Due to the large number of distances fitted for these organisms, any correction for sample size to the goodness of fit estimation was not relevant. A comparison of *r*^2^ values allowed to discard some distributions with a clear low fitting performance. In order to find the best fitting distribution among the remaining, we considered additionally the mean value of residual distribution (reported in Table 3), that allowed a further discrimination, also supported by visual inspection (see Additional file 1).

**Table 3:** Residual mean values of gamma, stretched exponential (S. Exp), double exponential (D. Exp), exponenital (Exp) and q-exponential (Q-exp) fit of mammal CG interdistance distributions

Mammal	Gamma	S. Exp	D. Exp	Exp	Q-exp
Bos taurus	-1.96E-11	-5.19E-6	1.05E-7	-7.65E-12	1.17E-1
Canis familiaris	-8.88E-11	-4.05E-6	3.26E-7	2.47E-8	1.18E-1
Equus caballus	6.53E-10	-5.86E-9	-3.67E-4	6.64E-12	1.51E-1
Homo sapiens	7.69E-10	-1.05E-6	1.21E-7	2.41E-9	1.40E-1
Macaca mulatta	3.13E-11	-2.93E-8	1.26E-7	1.02E-8	1.39E-1
Mus musculus	-2.04E-11	-2.93E-8	2.70E-7	4.00E-8	1.23E-1
Ornithorhynchus anatinus	8.37E-11	-1.25E-7	2.56E-7	3.63E-8	1.10E-1
Pan troglodytes	7.77E-10	-1.79E-6	1.90E-7	2.83E-9	1.39E-1
Rattus norvegicus	7.31E-10	-3.14E-9	1.35E-7	-3.09E-12	1.49E-1

### Multiple genome analysis

Once obtained the best fitting probability density function for the mammal organism set, we applied it to all organisms chosen for our analysis. The fit parameters associated to the best distribution, together with the goodness-of-fit parameters, were used to describe the analyzed organisms, individually or grouped by category, allowing to obtain a global picture from a point of view of organism complexity. We expected that genomes with similar CG interdistance distributions would show similar fit parameter values, reflecting similarities in the functional roles of CG dinucleotides in these organisms. Even if for some organism categories the chosen distribution is not optimal as for the initial subset, we hypothesize that organisms with similar distributions (even if not corresponding to the chosen one) should present similar parameters anyway, allowing a global classification with a unified approach. Anyway, to filter out possible fit errors due to bad genome sequence reconstruction, we only considered for our analyses the organisms which goodness-of-fit exceeded a value *r*^2^=0.9. With this filter we discarded on average about 15% of our genomes (from 2% in bacteria to 25% in non-mammal vertebrates), homogeneously distributed along the considered categories, resulting in 3857 genomes left for our analysis.

## Results

**Fig. 1:**
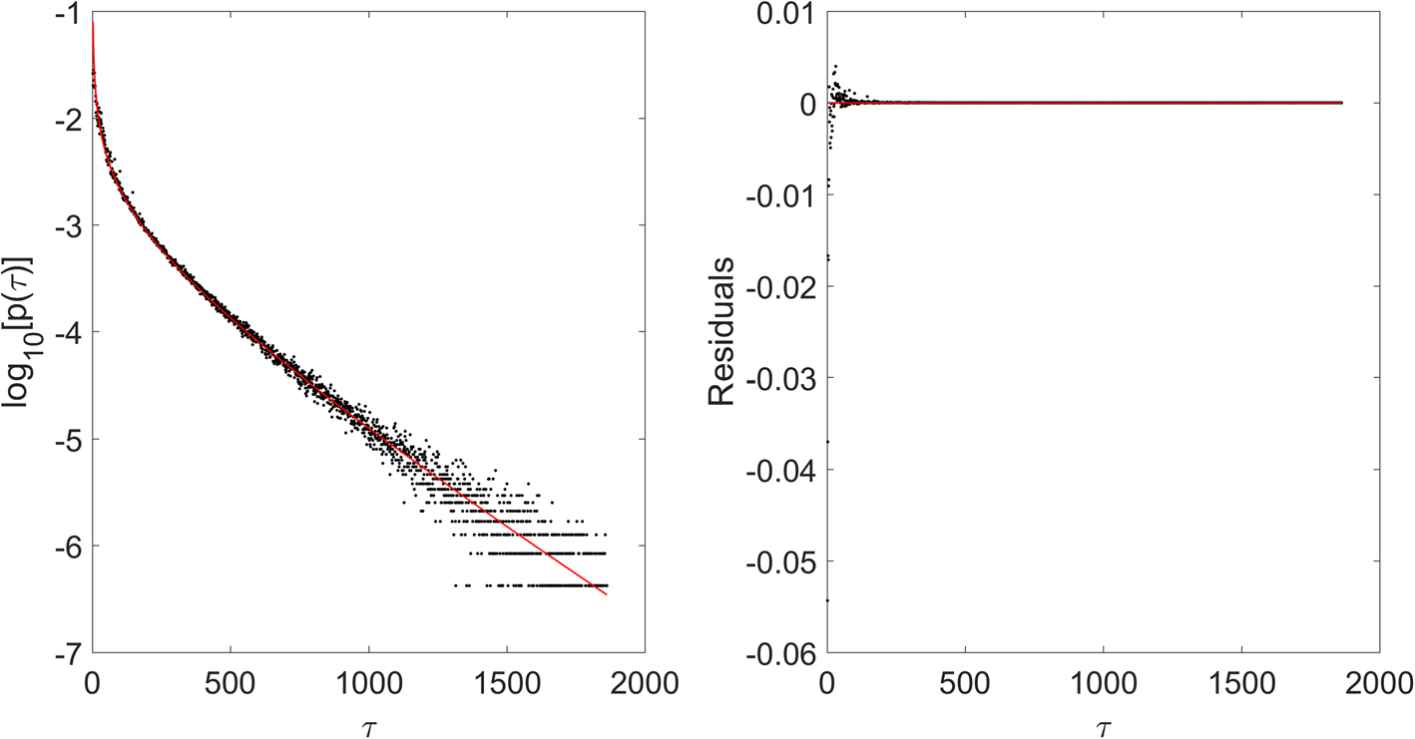
Log-linear plot of gamma fit result for Homo sapiens CG interdistance distribution in chromosome 1 (left-hand side), together with residual plot in linear scale (right-hand side)

Goodness-of-fit parameters showed that gamma distribution (Eq. 6) is the function that best describes CG interdistance distribution for the 9 mammal subset (see Fig. 1 for the case of human genome). In particular, if we look at *r*^2^ values in Table 4, we can see that the worst fit results are given by q-exponential distribution, since the corresponding *r*^2^ values are the lowest ones, followed by single exponential distribution. The choice of best fit distribution among the remaining was more difficult, because *r*^2^ values were very similar or even identical. Therefore, we also considered the mean values of residual distribution, that provided a clear distinction among the considered distributions (see Table 3), with values around 10^−11^ for gamma fit, 10^−8^ for stretched exponential fit, 10^−7^ for double exponential fit, 10^−8^ for exponential fit and 10^−1^ for q-exponential fit. These values confirmed that q-exponential was the worst fitting distribution, and showed that gamma is the best fit function for mammal CG interdistance distributions (see Table 5 for fit results).

**Table 4:** R-squared values of gamma, stretched exponential (S. Exp), double exponential (D. Exp), exponenital (Exp) and q-exponential (Q-exp) fit of mammal CG interdistance distributions

Mammal	Gamma	S. Exp	D. Exp	Exp	Q-exp
Bos taurus	0.982	0.982	0.981	0.961	0.805
Canis familiaris	0.981	0.981	0.977	0.947	0.832
Equus caballus	0.986	0.987	0.775	0.964	0.797
Homo sapiens	0.985	0.985	0.983	0.962	0.799
Macaca mulatta	0.987	0.987	0.986	0.965	0.804
Mus musculus	0.983	0.985	0.983	0.960	0.803
Ornithorhynchus anatinus	0.978	0.981	0.978	0.949	0.831
Pan troglodytes	0.986	0.985	0.984	0.963	0.800
Rattus norvegicus	0.984	0.987	0.985	0.958	0.800

**Table 5:** Gamma fit parameter values for the first set of 9 mammals. Errors on parameters are estimated at 95% confindence level and rounded to the first significant digit

Mammal	Sequence	a	b	c	r^2^
Bos taurus	chromosome 1	0.25 ± 0.03	316 ± 5	0.10 ± 0.02	0.982
Canis familiaris	chromosome 1	0.03 ± 0.03	324 ± 7	0.23 ± 0.04	0.981
Equus caballus	chromosome 1	0.17 ± 0.03	226 ± 4	0.16 ± 0.03	0.986
Homo sapiens	chromosome 1	0.16 ± 0.03	280 ± 5	0.14 ± 0.02	0.985
Macaca mulatta	chromosome 1	0.17 ± 0.03	267 ± 4	0.15 ± 0.02	0.987
Mus musculus	chromosome 1	0.22 ± 0.03	330 ± 6	0.12 ± 0.02	0.983
Ornithorhynchus anatinus	chromosome 3	0.15 ± 0.04	250 ± 6	0.16 ± 0.03	0.978
Pan troglodytes	chromosome 1	0.16 ± 0.03	281 ± 5	0.14 ± 0.02	0.986
Rattus norvegicus	chromosome 1	0.09 ± 0.03	281 ± 5	0.21 ± 0.04	0.984

**Fig. 2:**
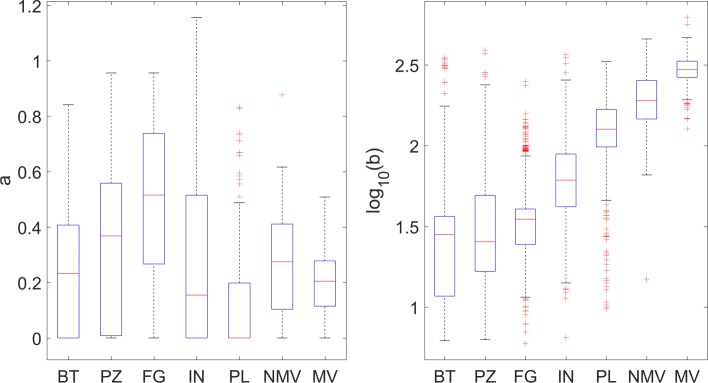
Boxplot of gamma shape parameter a (left-hand side) and gamma scale parameter b (right-hand side) for the seven considered categories: bacteria (BT), protozoa (PZ), fungi (FG), invertebrates (IN), plants (PL), non-mammal vertebrates (NMV) and mammal vertebrates (MV)

**Fig. 3:**
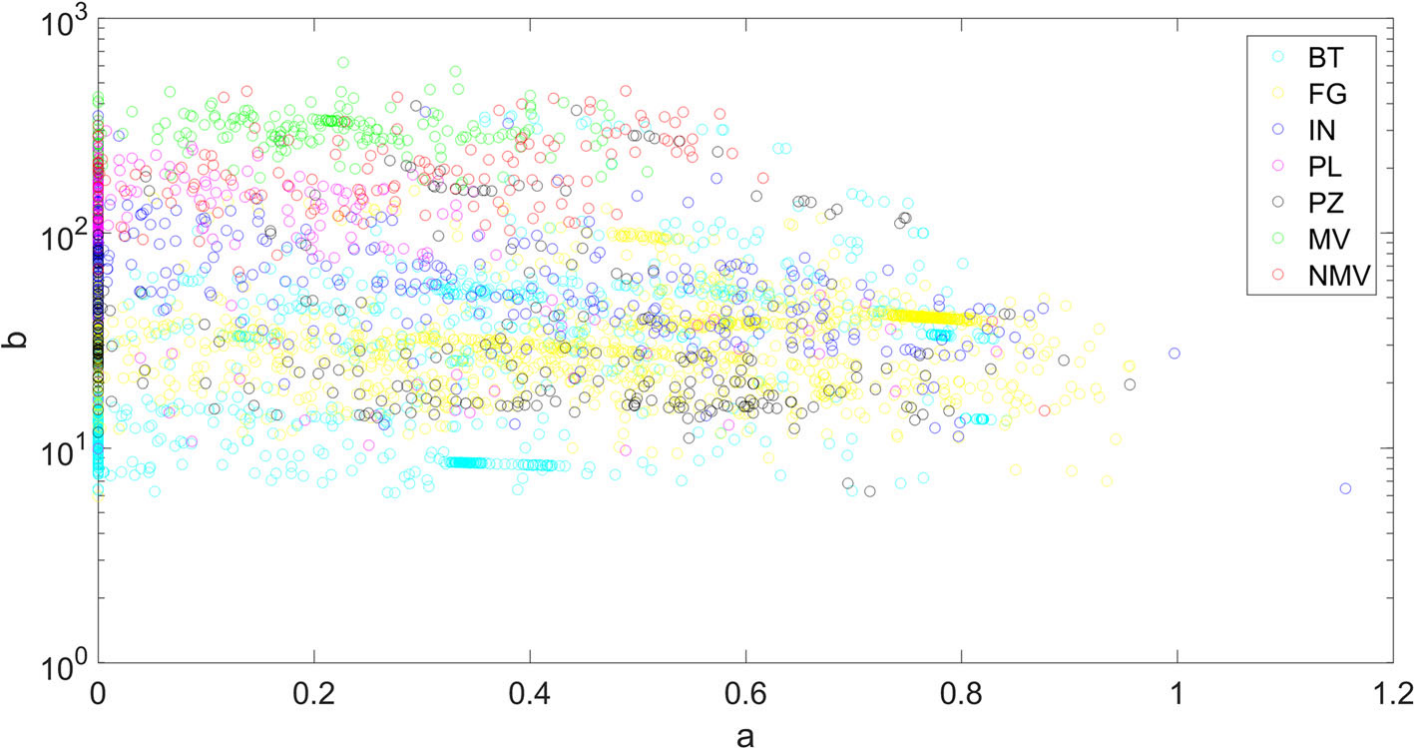
Semilogarithmic plot of gamma scale parameter b as function of gamma shape parameter a for the 4425 analyzed genomes, divided into seven categories: bacteria (BT), fungi (FG), invertebrates (IN), plants (PL), protozoa (PZ), mammal vertebrates (MV) and non-mammal vertebrates (NMV)

Looking at Fig. 2, we notice that *b* is the parameter that mainly discriminates between the organism categories while the value *a* of the power term in gamma distribution is equally spread across all organisms of all categories (see also Fig. 3). Furthermore, *b* values seem to increase with the “biological complexity” of the considered categories, being minimum for bacteria and protozoa, and maximum for vertebrates (higher in mammals than in non-mammals) and with an intermediate value for invertebrates. Vertebrate categories have a median value of *b* in the range 200−300, while it is an order of magnitude lower for bacteria (about 30). We remark that this value is very close to the typical length of DNA enveloped around a histone (146 bp envelope around histone octamer plus a linker region summing up to about 200-220 bp), thus there might be a relation between DNA enveloping around histones and our observation in term of CG distances, even if we cannot provide an explanation for this.

Since we are considering a large class of organisms, with DNA sequence size differing by several orders of magnitude (from 10^8^ for mammals to 10^4^−10^5^ for bacteria and protozoa), we checked if *b* parameter could be associated with the length of the analyzed genomic sequence. This does not seem the case, since the Pearson’s correlation coefficient *r* between the logarithm of *b* and the logarithm of the length of the analyzed genome sequences is very close to zero: *r*=−0.12.

**Fig. 4:**
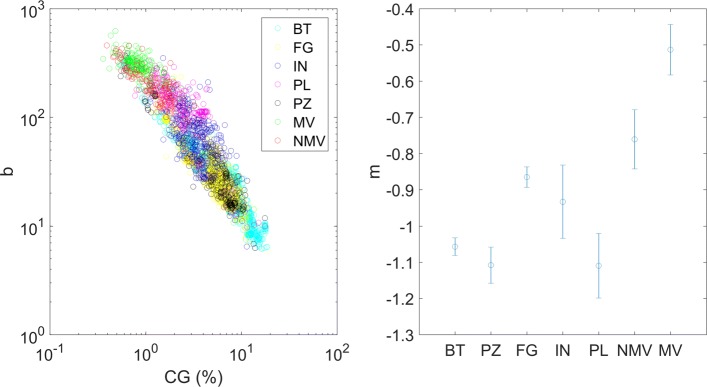
Double logarithmic plot of gamma scale parameter b as a function of CG percentage for each of the 4425 genomes belonging to the seven considered categories: bacteria (BT), protozoa (PZ), fungi (FG), invertebrates (IN), plants (PL), non-mammal vertebrates (NMV) and mammal vertebrates (MV), (left-hand side). Plot of the angular coefficient m obtained from linear regression of CG-b relationship, for each considered category (right-hand side)

In light of these observations, we also tested whether the gamma scale parameter (i.e., *b*) could depend on CG density inside the sequence (number of CG dinucleotides with respect to sequence length), representing *b* as a function of %CG in double logarithmic scale (see Fig. 4). In a simple null model, the average distance between dinucleotides should decrease proportionally to the inverse of dinucleotide density inside the sequence, thus with a slope equal to −1 in double logarithmic plot. Therefore, we fitted the *b* vs %CG double logarithmic plot to a straight line using linear least square method, obtaining the results shown in Table 6. We observe that the relation between *b* and %CG is in general very close to the fitted lines for each organism category, with average value of Pearson’s coefficient 〈*r*〉=−0.65 (minimum correlation *r*_*MIN*_=−0.54 for invertebrates, maximum correlation *r*_*MAX*_=−0.75 for protozoa). From this analysis we can identify two groups of organisms, according to values of the coefficient *m*, corresponding to the slope of the line in log-log plot and thus to the exponent of the polynomial relation *b*∝*%CG*^*m*^: bacteria, plants, fungi, protozoa and invertebrates have an exponent approximately equal to −1, while mammal vertebrates and non-mammal vertebrates have a smaller exponent in absolute value closer to 0.5, significantly different from the others in terms of 95% confidence interval. Some organism categories thus seem to verify the null model hypothesis, while for vertebrates the significant deviation from the null model suggests a different mechanism for CG dinucleotide placement along the genome rather than a “maximum entropy” process.

**Table 6:** Linear regression parameters of CG-b relationship, together with r-squared values

Category	m	q	r^2^
Bacteria	-1.06 ± 0.02	2.23 ± 0.02	0.858
Protozoa	-1.11 ± 0.05	2.3 ± 0.04	0.875
Fungi	-0.87 ± 0.03	2.07 ± 0.02	0.726
Invertebrates	-0.9 ± 0.1	2.3 ± 0.06	0.460
Plants	-1.11 ± 0.09	2.5 ± 0.04	0.707
Vertebrates non-mammals	-0.76 ± 0.08	2.34 ± 0.02	0.704
Vertebrates mammals	-0.51 ± 0.07	2.43 ± 0.01	0.523

## Discussion

A possible biological interpretation of this grouping could be a different role of CG methylation in these two classes of organisms. CG methylation is known to be an important mechanism in higher-order organisms (like vertebrates, that in our analysis show a slope significantly smaller than −1), with an active role on gene transcription regulation [24]. For most of the biological categories that showed an exponent close to −1 it is not clear how (or even if) the CG methylation mechanism is used [25–27], since in some cases different nucleotide sequences are involved in methyl group binding (like the GATC motif in *E. Coli*, or other motifs in plants [28]) and in general is not used for gene regulation, if not only during embryonic development [29]. We speculate that a characterization of CG distribution parameters for a specific organism could be an index to hypothesize a role of CG methylation at a single organism level, even if we did not go further in the analysis in this direction. In order to extend the range of applications, we think that the method developed in this work can be applied to further repeated genomic sequences (e.g. transcription-factor-binding-site motifs mapped in ENCODE project [30] and repeated sequences associated to transposable elements [31]) in order to gain a deeper insight into DNA properties of single organisms or for comparison between oganism categories. Moreover, considering our approach as providing a null model for CG (or other dinucleotide) distribution, we can look for deviations from such null model and study their possible biological meaning (e.g. in relation to CpG islands).

## Conclusions

We considered several probability density functions to fit the CG interdistance distribution of a selected set of mammal organisms, and we observed that it is best described by a Gamma distribution. Applying this function on a wide set of organisms, taken from different taxonomic categories, we noticed that the scale parameter *b* of the Gamma distribution could be associated to the biological complexity of the organism category, increasing from bacteria to vertebrates. Moreover, we tested for possible factors affecting this parameter, like genome sequence length and CG density. While the first was not related to our observations, the second revealed stronger correlations; in particular, for a group of organisms, comprising those of minor biological complexity (bacteria, protozoa, fungi, invertebrates and plants), the relation between *b* and CG density could be explained by a minimal null model, while for higher order organisms (vertebrates) this null model did not explain the observations. We argue that this difference could be related to the different role that CG methylation plays in these classes of organisms.

## Additional file

Additional file 1 The additional file contains: a section where we show our fitting method performance on different synthetic data sets; a section where we show the plot of gamma and stretched exponential fit results for CG interdistance distribution of *Homo sapiens*; a section where we show how we calculated errors on r-squared, based on Olkin and Finn’s approximation; a final section where we collected into two tables all the informations about the analysis perfomed on the 4425 organisms. The first table contains informations about organism type and identification on NCBI website; the second contains gamma fit parameters, ratio of unkown nucleotides (%N), ratio of CG dinucleotides (%CG) and r-squared values. (PDF 1960 kb)

## Abbreviations

BT: Bacteria
DNA: DeoxyriboNucleic acid
FG: Fungi
IN: Invertebrates
PL: Plants
PZ: Protozoa
MV: Mammal vertebrates
NMV: Non-mammal vertebrates
