# Supplementary material for: Statistical modelling of CG interdistance across multiple organisms
Source: BMC Bioinformatics. 2018 Oct 15;19(Suppl 10):355. doi: 10.1186/s12859-018-2303-2 (PMC6191944; doi:10.1186/s12859-018-2303-2)
Supplement: Supplementary file 1 — The additional file contains: a section where we show our fitting method performance on different synthetic data sets; a section where we show the plot of gamma and stretched exponential fit results for CG interdistance distribution of Homo sapiens; a section where we show how we calculated errors on r-squared, based on Olkin and Finn’s approximation; a final section where we collected into two tables all the informations about the analysis perfomed on the 4425 organisms. The first table contains informations about organism type and identification on NCBI website; the second contains gamma fit parameters, ratio of unkown nucleotides (%N), ratio of CG dinucleotides (%CG) and r-squared values. (PDF 1960 kb) [file 12859_2018_2303_MOESM1_ESM.pdf]

SUPPLEMENTARY
Statistical modelling of CG interdistance across multiple organisms

Merlotti A.[1], *Faria do Valle I.*[2], *Castellani G.*[1], *Remondini D.*[1]

1 Fitting method performance

We generated several sets of 2.000.000 and 10.000.000 random number drawn from a gamma distribution with shape parameter $a = 0.2$ and scale parameter $b = 250$, in order to test the performance of our fitting procedure.
We represented the data by taking the logarithm of y-axis values and applying a non-linear least squares regression (via Matlab `fit` function); we considered firstly the entire distribution, and subsequently a truncated version by removing samples from 95th, 90th and 85th percentile.

1.1 Results

In Table 1 and 2 are shown respectively fit results for the first (2.000.000 gamma random numbers) and the second (10.000.000 gamma random numbers) synthetic dataset. We can see that by removing at least the right 10% tail we obtain a better parameter estimation.

**a**	**b**	**c**	r^2	**cutoff**
0[1]	320 ± 5	0.32 ± 0.02	0.965 ± 0.004	none
0.10 ± 0.04	272 ± 6	0.25 ± 0.05	0.983 ± 0.002	95th percentile
0.18 ± 0.04	252 ± 5	0.18 ± 0.03	0.986 ± 0.002	90th percentile
0.21 ± 0.04	245 ± 5	0.16 ± 0.03	0.986 ± 0.002	85th percentile

Table 1: *Gamma fit parameter values obtained by performing non-linear least squares regression on semilogarithmic scale. Errors are estimated at 95% confindence level.*

[1] In these simulations the algorithm did not converge.

**a**	**b**	**c**	r^2	**cutoff**
0^2	292 ± 3	0.41 ± 0.02	0.980 ± 0.002	none
0.15 ± 0.04	258 ± 4	0.23 ± 0.04	0.989 ± 0.001	95th percentile
0.19 ± 0.03	250 ± 4	0.19 ± 0.03	0.990 ± 0.001	90th percentile
0.22 ± 0.03	244 ± 4	0.17 ± 0.03	0.992 ± 0.001	85th percentile

Table 2: *Gamma fit parameter values obtained by performing non-linear least squares regression on semilogarithmic scale. Errors are estimated at 95% confindence level.*

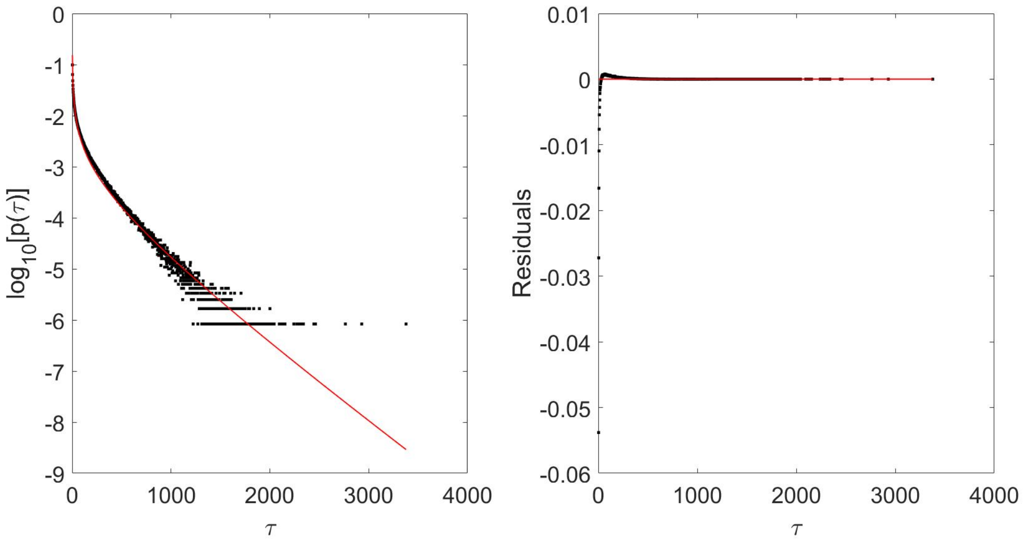

Figure 1: *Gamma fit results for the complete distribution, obtained by 2.000.000 synthetic gamma random numbers (left-hand side), together with residual plot (right-hand side).*

[2] The algorithm did not converge.

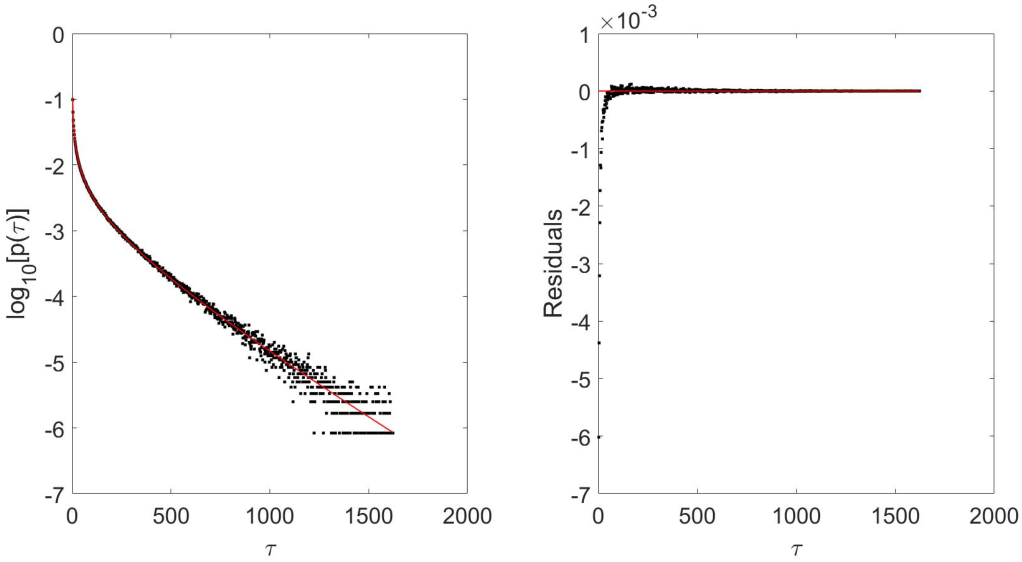

Figure 2: *Gamma fit results for the distribution truncated at 90th percentile and obtained by 2.000.000 synthetic gamma random numbers (left-hand side), together with residual plot (right-hand side).*

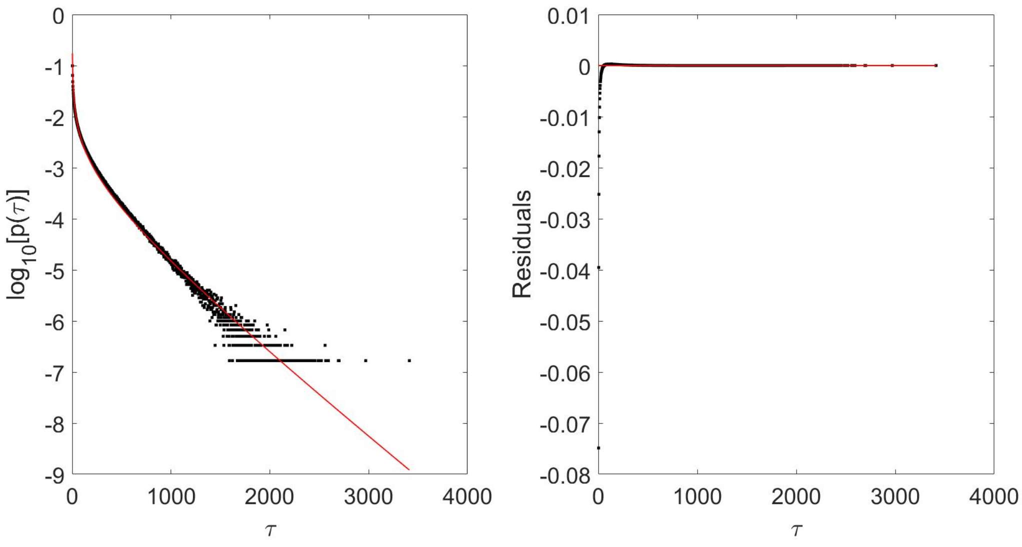

Figure 3: *Gamma fit results for the complete distribution, obtained by 10.000.000 synthetic gamma random numbers (left-hand side), together with residual plot (right-hand side).*

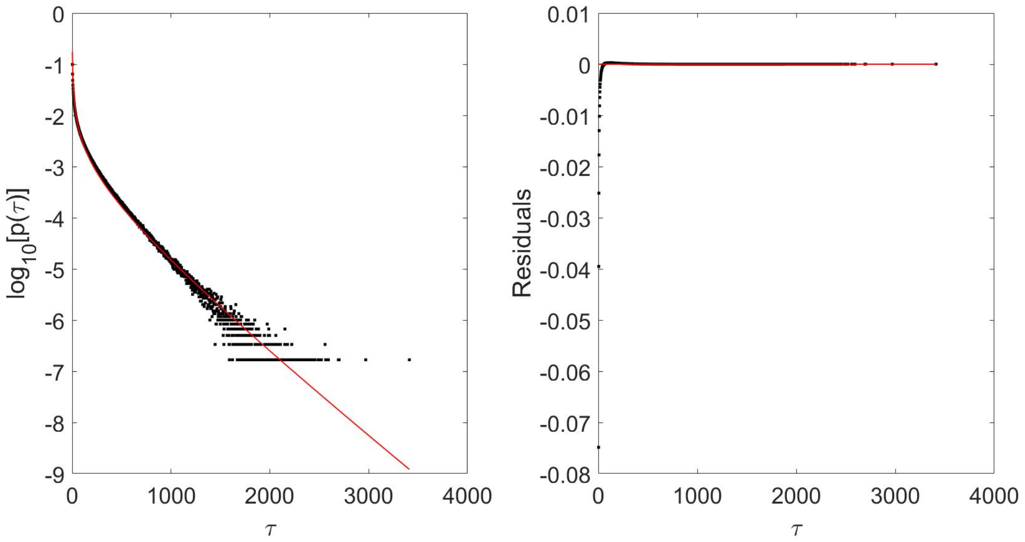

Figure 4: *Gamma fit results for the distribution truncated at 90th percentile and obtained by 10.000.000 synthetic gamma random numbers (left-hand side), together with residual plot (right-hand side).*

2 Fit result plots

We show visually a difference in the goodness of fit between Gamma and Stretched exponential distributions: as it can be seen in Figures. 5,6, the Gamma distribution has a better adherence to real data in the initial part of the distribution, while both distributions have similar performance on the empirical distribution tail.

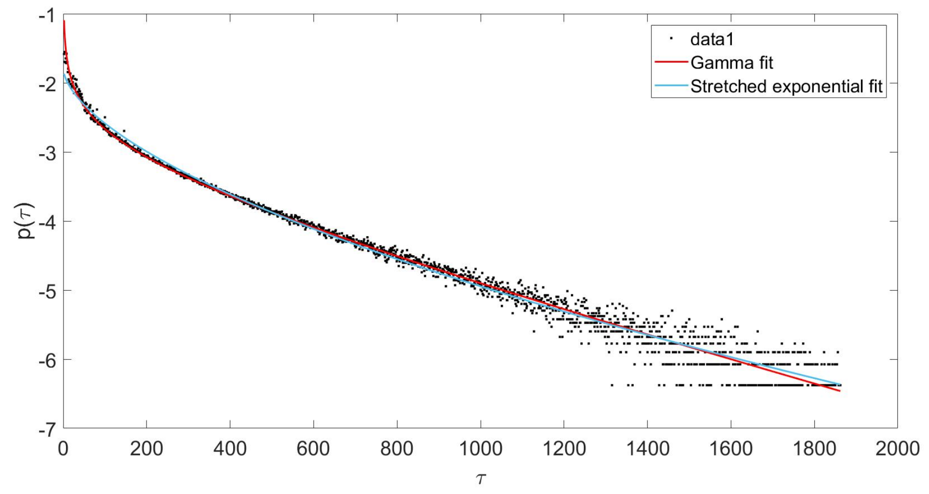

Figure 5: *Gamma (red) and stretched exponential (light blue) fit of Homo sapiens CG interdistance distribution inside chromosome 1.*

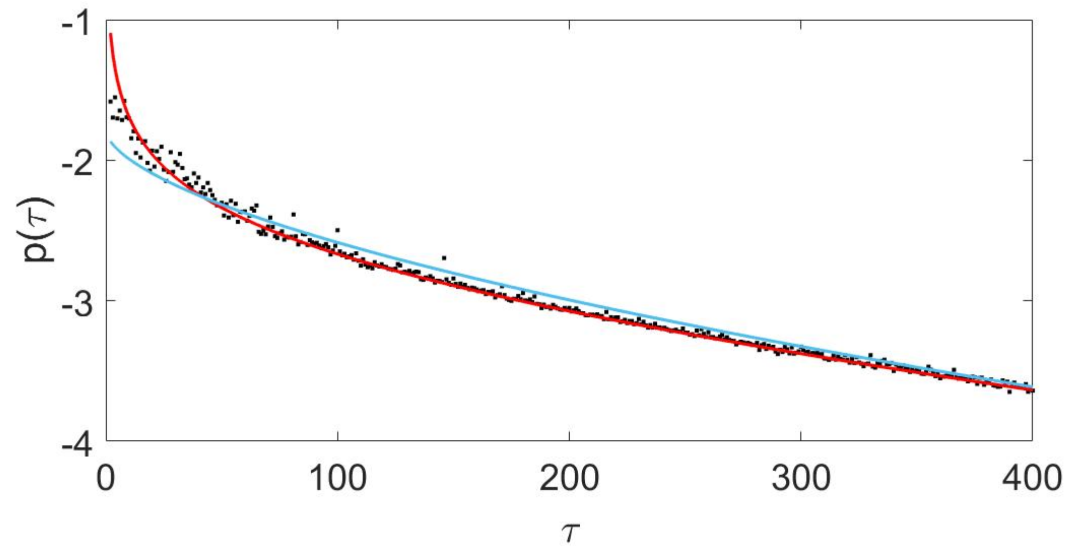

Figure 6: *Zoom of gamma (red) and stretched exponential (light blue) fit of Homo sapiens CG interdistance distribution inside chromosome 1.*

3 Errors on R-squared

For each r^2 value we calculated an error estimate at 95% of confidence interval (see eq. 1), using the following definition:

$$r^2 \pm 2 \cdot SE \tag{1}$$

where SE is the standard error for r^2, based on Olkin and Finn's approximation:

$$SE = \left(\frac{4r^2(1-r^2)^2(n-k-1)^2}{(n^2-1)(3+n)} \right)^{\frac{1}{2}} \tag{2}$$

where k is the number of predictors in the model and n the total sample size.

**Mammal**	**Gamma**	**S. Exp**	**D. Exp**	**Exp**	**Q-exp**
Bos taurus	0.982 ± 0.002	0.982 ± 0.002	0.981 ± 0.002	0.961 ± 0.004	0.81 ± 0.02
Canis familiaris	0.981 ± 0.002	0.981 ± 0.002	0.977 ± 0.002	0.947 ± 0.005	0.83 ± 0.01
Equus caballus	0.986 ± 0.001	0.987 ± 0.001	0.775 ± 0.002	0.964 ± 0.004	0.80 ± 0.02
Homo sapiens	0.985 ± 0.001	0.985 ± 0.001	0.983 ± 0.002	0.962 ± 0.003	0.80 ± 0.02
Macaca mulatta	0.987 ± 0.001	0.987 ± 0.001	0.986 ± 0.006	0.965 ± 0.003	0.80 ± 0.02
Mus musculus	0.983 ± 0.002	0.985 ± 0.001	0.983 ± 0.002	0.960 ± 0.004	0.80 ± 0.02
Ornithorhynchus anatinus	0.978 ± 0.002	0.981 ± 0.002	0.978 ± 0.002	0.949 ± 0.005	0.83 ± 0.02
Pan troglodytes	0.986 ± 0.001	0.985 ± 0.001	0.984 ± 0.002	0.963 ± 0.003	0.80 ± 0.02
Rattus norvegicus	0.984 ± 0.001	0.987 ± 0.001	0.985 ± 0.001	0.958 ± 0.004	0.80 ± 0.02

Table 3: *R-squared values of gamma, stretched exponential (S. Exp), double exponential (D. Exp), exponenital (Exp) and q-exponential (Q-exp) fit for CG interdistance distributions of higher-order organisms. Errors are estimated at 95% confindence interval and rounded to the first significant digit.*

4 Tables containing fit results on 4425 organisms

We provide two tables, one with information on organism type and identification on NCBI website, and the other with all fit parameters of the Gamma distributions, ratio of unknown nucleotides (%N), ratio of CG dinucleotides (%CG) and goodness-of-fit r^2 value.

Sequence ID	a gamma	error	b gamma	error	c gamma	error	Sequence length	%N	%CG	r2
'AE014297.3'	0.00	-1.00	56.51	1.02	0.39	0.03	32079331	0.08	4.23	0.98
'CM000663.2'	0.16	0.03	280.18	4.61	0.14	0.02	248956422	7.42	0.95	0.99
'CM000314.3'	0.16	0.03	280.93	4.53	0.14	0.02	228573443	2.09	1.00	0.99
'CM000550.1'	0.20	0.03	273.38	4.50	0.13	0.02	229942017	6.04	0.93	0.98
'CM000994.2'	0.19	0.03	334.94	5.93	0.14	0.02	195471971	1.82	0.76	0.98
'CP002684.1'	0.00	0.07	117.14	4.58	0.34	0.10	30427671	0.54	2.29	0.97
'CM000070.3'	0.00	-1.00	72.45	2.14	0.21	0.03	30819483	1.67	4.16	0.95
'CM000072.5'	0.09	0.03	280.64	4.95	0.21	0.04	282763074	5.20	0.95	0.98
'GL010027.1'	0.51	0.03	192.51	3.18	0.05	0.01	129759341	1.53	0.86	0.98
'DS995899.1'	0.20	0.13	32.37	1.83	0.31	0.13	6407042	0.22	5.11	0.98
'CM002888.1'	0.00	-1.00	212.88	3.11	0.20	0.01	76625712	0.08	1.94	0.97
'KB941397.1'	0.03	0.08	99.72	5.59	0.31	0.10	6102535	18.39	2.15	0.97
'CH003449.1'	0.27	0.03	275.37	4.10	0.10	0.01	234033217	4.30	0.84	0.99
'CM000463.1'	0.26	0.03	276.85	4.45	0.10	0.02	234808360	0.61	0.89	0.98
'BL000001.2'	0.16	0.04	286.24	5.61	0.15	0.03	158329839	1.92	0.99	0.98
'CM000209.2'	0.22	0.03	330.00	5.52	0.12	0.02	199526509	7.93	0.70	0.98
'BA000047.1'	0.31	0.05	330.39	14.29	0.08	0.02	12263273	8.97	0.65	0.95
'CM000054.5'	0.00	-1.00	210.03	6.92	0.04	0.00	29893408	8.61	4.48	0.92
'KN913731.1'	0.00	0.08	170.74	13.76	0.26	0.09	2525675	6.23	1.93	0.95
'CM000308.1'	0.17	0.03	266.55	4.14	0.14	0.02	228252215	6.49	1.00	0.99
'CM000231.2'	0.12	0.03	276.57	4.80	0.19	0.03	256108954	5.04	0.93	0.98
'CM000411.1'	0.15	0.04	249.86	6.17	0.16	0.03	59581953	6.44	1.02	0.98
'CM000039.3'	0.08	0.03	359.78	7.12	0.18	0.03	123869142	0.55	0.98	0.98
'CM000368.1'	0.33	0.02	565.52	6.26	0.05	0.01	748055161	1.96	0.43	0.98
'CM000377.2'	0.17	0.03	226.39	3.97	0.16	0.03	185838109	1.22	1.13	0.99
'CM000093.4'	0.35	0.03	243.61	3.69	0.08	0.01	196202544	0.55	0.88	0.99
'CM000915.2'	0.00	-1.00	64.52	1.79	0.13	0.01	33571687	8.66	6.09	0.96
'CM000278.3'	0.11	0.07	76.92	2.68	0.28	0.08	31381287	5.67	2.70	0.98
'CM000455.1'	0.01	0.11	53.81	3.42	0.39	0.14	3120417	0.10	4.42	0.98
'DS544890.1'	0.00	-1.00	618.97	38.69	0.08	0.01	5387533	5.17	1.67	0.87
'CH991767.1'	0.56	0.13	26.66	1.51	0.13	0.05	1886627	0.20	4.89	0.98
'CH464491.1'	0.00	-1.00	50.13	1.75	0.30	0.04	5261801	0.04	5.12	0.96
'CM001232.1'	0.26	0.12	26.73	1.26	0.27	0.11	8319966	0.04	6.18	0.99
'CR382126.1'	0.93	0.10	35.67	1.50	0.04	0.01	2602197	0.00	2.86	0.98
'CR382131.1'	0.35	0.13	31.91	1.75	0.20	0.08	4224103	0.01	4.82	0.98
'CR380958.2'	0.54	0.10	60.93	3.78	0.08	0.03	1455689	0.00	2.44	0.96
'DS496108.1'	0.00	-1.00	22.29	0.69	0.45	0.06	6494944	5.86	8.51	0.98
'CM000169.1'	0.00	-1.00	96.53	9.91	0.07	0.02	4918979	0.88	5.37	0.79
'DS027059.1'	0.00	0.25	511.37	216.68	0.02	0.02	4489098	0.03	5.46	0.64
'FR796432.1'	0.41	0.12	15.75	0.73	0.27	0.08	2682151	0.00	8.62	0.99
'AE014187.2'	0.50	0.06	283.47	17.42	0.04	0.01	3291871	0.00	0.64	0.91
'CM000337.2'	0.22	0.05	158.79	3.96	0.15	0.03	48367220	1.72	1.43	0.98
'DS113177.1'	0.41	0.12	86.88	9.31	0.09	0.04	584929	0.36	2.05	0.92
'AM269894.1'	0.00	-1.00	74.58	4.94	0.25	0.04	1347714	34.01	2.82	0.92
'FR799010.1'	0.42	0.12	18.41	0.85	0.24	0.08	2686643	0.20	7.43	0.99
'AM270981.1'	0.11	0.14	34.12	2.22	0.36	0.15	3626085	0.12	5.50	0.98
'CH991543.1'	0.00	0.22	35.96	3.74	0.31	0.21	3607471	3.36	6.25	0.95
'FR796468.1'	0.31	0.14	16.41	0.94	0.32	0.12	2673956	0.04	8.66	0.99
'CU329670.1'	0.69	0.08	43.03	1.56	0.06	0.02	5579133	0.00	2.88	0.98
'BX284605.5'	0.22	0.09	60.39	2.42	0.22	0.08	20924180	0.00	3.05	0.98
'LN857024.1'	0.54	0.08	68.05	2.80	0.07	0.02	5235760	1.49	2.14	0.97
'CM000812.5'	0.09	0.03	348.18	5.96	0.18	0.03	274330532	0.00	0.96	0.98
'GK000001.2'	0.25	0.03	316.31	5.48	0.10	0.02	158337067	0.66	0.81	0.98
'CAAJ0100193	0.95	0.18	245.28	175.02	0.01	0.01	16829	0.00	1.30	0.32
'CM000695.1'	0.00	0.03	289.29	5.79	0.27	0.05	279330936	41.80	0.72	0.98
'EQ962652.1'	0.27	0.12	34.18	1.74	0.24	0.09	5640589	0.14	4.78	0.98
'CM000761.2'	0.00	-1.00	152.86	2.82	0.13	0.01	77691612	1.20	3.50	0.96
'CM000177.6'	0.24	0.03	317.45	5.49	0.10	0.02	158972876	0.17	0.82	0.98
'CM000441.2'	0.48	0.06	313.80	19.45	0.04	0.01	4094363	0.04	0.60	0.91
'CR940347.1'	0.05	0.09	156.96	13.01	0.26	0.10	2592520	0.00	1.77	0.94
'CH476615.1'	0.00	-1.00	71.08	5.84	0.09	0.02	7891746	0.87	5.43	0.83
'EQ999973.1'	0.00	0.06	>1000	>1000	0.01	0.00	10302040	0.60	1.98	0.70
'GL014402.1'	0.00	0.09	129.21	10.03	0.27	0.10	3191517	14.04	2.18	0.95
'CM000790.1'	0.06	0.03	324.52	5.71	0.19	0.03	194850757	3.59	1.12	0.98
'CM000714.1'	0.33	0.10	51.68	2.52	0.17	0.06	5269725	0.46	3.21	0.98
'FN597038.1'	0.09	0.04	252.94	7.87	0.20	0.04	30274277	1.85	1.19	0.97
'GG666612.1'	0.00	0.08	133.09	6.91	0.22	0.07	11512737	3.96	2.75	0.96
'CH408076.1'	0.49	0.11	34.11	1.72	0.13	0.05	2423571	0.12	4.17	0.98
'GG657448.1'	0.00	0.06	>1000	>1000	0.02	0.01	8174884	1.00	1.63	0.72
'CM000742.1'	0.32	0.09	54.51	2.56	0.17	0.05	5561906	0.36	3.10	0.98
'CM000741.1'	0.37	0.09	51.77	2.30	0.15	0.05	5790501	0.71	3.10	0.98
'CH477186.1'	0.00	0.11	66.93	4.00	0.39	0.15	5856339	2.41	3.48	0.97
'GL048072.1'	0.23	0.09	740.47	295.68	0.09	0.03	472108	13.87	0.68	0.79
'CM002924.1'	0.10	0.05	173.03	5.09	0.22	0.05	39782674	1.79	1.50	0.98
'GL020027.1'	0.02	0.09	282.42	41.15	0.25	0.09	908834	4.82	1.31	0.91
'CM000831.1'	0.30	0.10	55.19	3.72	0.17	0.06	1255352	1.17	3.40	0.97
'KE546988.1'	0.68	0.10	39.80	1.91	0.07	0.02	2876736	0.17	3.08	0.98
'CM004443.1'	0.15	0.03	236.76	4.16	0.16	0.03	194866763	3.11	1.23	0.99
'GL348713.1'	0.00	-1.00	116.93	1.95	0.31	0.02	33132539	9.53	2.31	0.98
'GL192338.1'	0.00	-1.00	234.71	6.82	0.26	0.02	6047896	1.24	1.52	0.95
'CM000851.2'	0.00	0.05	222.47	6.27	0.28	0.06	58018742	1.78	1.56	0.98
'FR847123.2'	0.00	0.12	62.38	3.51	0.46	0.21	21540570	1.95	3.15	0.96
'CM000126.1'	0.00	-1.00	112.24	1.71	0.19	0.01	47244934	3.43	3.91	0.98
'CM000856.1'	0.13	0.03	312.20	5.36	0.17	0.03	210400635	5.34	0.91	0.98
'CM000151.3'	0.29	0.05	390.67	20.83	0.07	0.02	8484197	0.02	0.64	0.93
'GL277768.1'	0.00	-1.00	60.21	2.37	0.14	0.02	9412112	9.90	6.17	0.94
'GL277950.1'	0.00	-1.00	58.89	2.10	0.15	0.02	9412414	9.39	6.20	0.95
'GL290984.1'	0.00	-1.00	198.24	7.00	0.23	0.02	3242543	0.01	2.07	0.95
'KE141673.1'	0.39	0.03	294.66	5.22	0.06	0.01	99113652	3.00	0.69	0.98
'GG738845.1'	0.68	0.09	121.98	7.69	0.03	0.01	1555553	4.21	1.05	0.93
'CM007647.1'	0.00	0.09	183.79	7.93	0.06	0.02	307041717	1.20	4.34	0.94
'CH902617.1'	0.00	-1.00	65.55	1.06	0.35	0.02	23697760	0.96	3.99	0.99
'CH954177.1'	0.00	0.09	54.82	2.22	0.41	0.14	26641161	0.82	4.16	0.98
'CH916366.1'	0.07	0.07	67.53	2.28	0.31	0.08	24565398	1.98	3.41	0.99
'CH933806.1'	0.00	-1.00	88.57	1.72	0.24	0.02	34148556	0.69	3.71	0.97
'CH479179.1'	0.00	-1.00	53.61	1.36	0.37	0.04	11822988	2.09	4.33	0.97
'CH480815.1'	0.01	0.08	59.03	2.32	0.37	0.10	21120651	1.31	4.13	0.99
'CH940647.1'	0.00	-1.00	62.95	1.12	0.36	0.03	25233164	1.20	3.96	0.98
'CAAI0100073(	0.69	0.17	239.88	145.75	0.03	0.01	37075	0.01	1.37	0.56
'AP008207.2'	0.00	-1.00	112.92	1.70	0.17	0.01	45064769	3.97	4.06	0.98
'GJ065086.1'	0.46	0.08	270.59	34.00	0.05	0.02	826179	13.72	0.70	0.86
'CM000880.2'	0.00	-1.00	104.23	1.79	0.15	0.01	75119905	0.05	4.46	0.97
'CM000885.1'	0.14	0.03	345.35	6.88	0.15	0.03	299839927	58.36	0.36	0.98
'CM000357.1'	0.00	-1.00	51.61	1.07	0.25	0.02	61545105	2.30	5.41	0.98
'CH964272.1'	0.21	0.07	70.10	2.45	0.20	0.06	16660200	0.39	2.95	0.98

'CM000160.2'	0.10	0.07	51.12	1.61	0.30	0.08	28832112	0.75	4.22	0.99
'AAAC0100000	0.35	0.10	51.45	2.53	0.16	0.05	5093554	0.83	3.16	0.98
'EQ966233.1'	0.00	-1.00	27.69	1.47	0.42	0.08	1539559	15.36	6.19	0.95
'EQ963472.1'	0.12	0.12	36.01	1.98	0.35	0.13	4469204	0.21	5.05	0.98
'DS235882.1'	0.00	0.11	88.90	7.03	0.25	0.11	2788692	1.50	3.66	0.95
'GG692395.1'	0.25	0.08	131.74	8.62	0.14	0.05	2474448	0.32	1.60	0.95
'AM910996.2'	0.00	-1.00	85.47	3.47	0.32	0.04	3159095	4.26	2.90	0.94
'GG680918.1'	0.00	-1.00	47.07	2.40	0.34	0.05	1800699	0.74	4.86	0.94
'AAEL0200035	0.64	0.12	86.08	9.60	0.04	0.02	282140	0.00	1.67	0.90
'CR382138.2'	0.64	0.10	48.92	2.57	0.07	0.02	2305761	0.01	2.67	0.97
'DS989726.1'	0.39	0.08	265.90	25.08	0.06	0.02	1324930	0.00	0.84	0.89
'CM002045.1'	0.00	-1.00	29.21	0.91	0.40	0.05	7486190	0.21	7.05	0.97
'AE005174.2'	0.00	-1.00	37.03	1.50	0.23	0.04	5528445	0.08	7.22	0.96
'BN001308.1'	0.00	-1.00	46.00	2.64	0.23	0.05	4876093	0.02	5.70	0.91
'BX908798.1'	0.52	0.09	59.16	2.96	0.09	0.03	2414465	0.00	2.46	0.97
'CP000048.1'	0.76	0.08	135.87	10.55	0.02	0.01	922307	0.00	0.96	0.90
'CP000143.2'	0.00	-1.00	9.71	0.45	0.68	0.16	3188524	0.00	13.90	0.98
'CP000805.1'	0.82	0.16	13.61	0.77	0.12	0.05	1139457	0.04	7.50	0.99
'CP000878.1'	0.60	0.09	74.76	4.25	0.06	0.02	1688963	0.01	1.87	0.96
'CP001139.1'	0.62	0.10	36.34	1.70	0.09	0.03	2905029	0.01	3.50	0.98
'CP001277.1'	0.15	0.15	49.04	4.14	0.28	0.14	2110331	0.00	3.89	0.96
'CP001752.1'	0.82	0.16	13.61	0.77	0.12	0.05	1139281	0.04	7.51	0.99
'CU928162.2'	0.00	-1.00	31.36	1.16	0.31	0.05	5209548	0.00	7.29	0.97
'CU928163.2'	0.00	-1.00	32.75	1.29	0.28	0.04	5202090	0.00	7.39	0.96
'CU928179.1'	0.85	0.12	38.58	2.07	0.04	0.02	1865392	0.00	2.80	0.97
'FM864216.2'	0.38	0.08	175.68	16.29	0.08	0.02	846214	0.17	1.19	0.92
'FM992688.1'	0.20	0.09	137.63	9.31	0.17	0.06	3214061	0.00	1.61	0.94
'FN392235.1'	0.00	-1.00	27.65	1.05	0.38	0.06	4026286	0.02	7.68	0.97
'FN392319.1'	0.81	0.10	39.85	1.82	0.05	0.02	2798491	0.00	2.78	0.97
'FN545816.1'	0.36	0.06	345.53	23.54	0.06	0.02	4191339	0.00	0.67	0.91
'CU468230.2'	0.30	0.12	48.91	3.12	0.18	0.08	3421954	0.00	3.36	0.97
'CU633749.1'	0.26	0.23	8.97	0.67	0.53	0.28	3416911	0.00	13.33	0.99
'AM999887.1'	0.76	0.10	70.41	4.16	0.03	0.01	1482455	0.01	1.70	0.95
'CM000937.1'	0.36	0.03	218.92	3.08	0.07	0.01	263920458	4.48	1.01	0.99
'GL345134.1'	0.43	0.07	176.23	12.08	0.06	0.02	1888931	8.65	0.99	0.93
'BA000046.3'	0.12	0.04	279.93	8.24	0.17	0.03	32799110	0.28	1.09	0.97
'CP001574.1'	0.04	0.13	26.11	1.71	0.44	0.16	2053059	0.00	8.03	0.99
'AE016819.5'	0.02	0.13	31.46	2.14	0.44	0.16	1836693	0.00	6.71	0.98
'AE017341.1'	0.42	0.15	27.99	1.78	0.19	0.08	2300533	0.01	5.02	0.97
'X59720.2'	0.63	0.14	45.24	4.49	0.07	0.03	316613	0.00	2.98	0.94
'AP006502.2'	0.58	0.16	12.80	0.71	0.22	0.08	1621617	0.00	8.74	0.99
'AL590450.1'	0.87	0.16	32.03	2.97	0.05	0.02	267509	0.00	3.35	0.95
'FN692037.1'	0.58	0.10	50.37	2.74	0.08	0.03	2043161	0.03	2.75	0.97
'CP000581.1'	0.09	0.16	10.73	0.70	0.58	0.20	1152508	0.00	15.10	0.99
'CU928171.1'	0.50	0.14	27.29	1.76	0.15	0.06	1720065	0.00	5.18	0.98
'GL349433.1'	0.00	-1.00	19.16	1.21	0.37	0.08	716227	1.67	10.96	0.95
'DS028118.1'	0.18	0.15	25.05	1.48	0.35	0.15	6928287	6.54	6.23	0.98
'GL349630.1'	0.00	-1.00	122.47	4.13	0.26	0.02	3073041	3.44	2.78	0.96
'GL376636.1'	0.00	0.18	27.71	2.54	0.40	0.20	1829366	5.97	7.55	0.97
'GL629765.1'	0.00	0.26	47.72	6.46	0.13	0.11	4437291	1.73	7.33	0.90
'GG742441.1'	0.00	0.25	34.29	4.85	0.29	0.21	962033	0.00	7.45	0.93
'GL377302.1'	0.00	-1.00	18.24	0.57	0.63	0.09	7102125	0.71	8.86	0.98
'CH408029.1'	0.00	0.17	25.59	1.83	0.51	0.26	6612759	1.35	7.22	0.98
'GL377338.1'	0.00	-1.00	94.82	1.76	0.25	0.01	15679321	10.30	3.50	0.98
'GL377565.1'	0.00	-1.00	271.20	16.65	0.05	0.01	6951972	0.67	3.93	0.85
'GL378323.1'	0.00	-1.00	39.76	1.82	0.24	0.04	5981844	4.40	6.47	0.94
'JH798064.1'	0.11	0.13	49.98	3.53	0.32	0.14	2452738	0.20	3.86	0.97
'DS547091.1'	0.00	0.25	127.20	25.49	0.06	0.05	3566367	2.56	4.88	0.79
'CM000961.1'	0.16	0.24	11.70	1.05	0.54	0.29	2349953	0.01	11.38	0.98
'GL379786.1'	0.67	0.09	41.13	1.60	0.07	0.02	4147112	1.23	3.05	0.98
'GL385395.1'	0.00	-1.00	23.64	0.59	0.53	0.06	8015784	6.76	7.47	0.99
'BK006938.2'	0.75	0.11	41.06	2.28	0.06	0.02	1531933	0.00	2.85	0.97
'CP001952.1'	0.90	0.14	45.74	4.24	0.03	0.01	236244	0.00	2.39	0.93
'CM000962.2'	0.55	0.03	225.14	2.87	0.03	0.00	190651702	1.45	0.72	0.99
'CM000960.1'	0.00	-1.00	27.98	0.98	0.39	0.05	4639637	0.69	7.42	0.97
'CM001648.1'	0.23	0.03	256.83	4.70	0.12	0.02	163208435	6.14	0.95	0.98
'CH445325.1'	0.00	-1.00	60.18	4.59	0.12	0.03	2531949	0.51	6.24	0.87
'CH476621.1'	0.14	0.12	56.27	3.85	0.28	0.11	2777073	0.60	3.52	0.97
'GL429767.1'	0.13	0.03	338.28	7.86	0.15	0.03	64990205	1.15	0.92	0.98
'GL475358.1'	0.43	0.12	41.46	2.74	0.14	0.05	1087348	6.49	3.66	0.97
'GL438234.1'	0.00	-1.00	92.28	3.50	0.19	0.02	2671896	1.97	4.51	0.95
'GL451853.1'	0.00	-1.00	44.14	2.05	0.17	0.02	2276656	1.32	8.90	0.95
'JH119215.1'	0.34	0.10	156.98	19.71	0.09	0.03	509487	4.01	1.36	0.90
'GL455988.1'	0.00	-1.00	105.51	1.84	0.21	0.01	17457517	0.17	4.04	0.98
'GL433835.1'	0.13	0.15	20.92	1.29	0.41	0.16	3119887	5.18	7.99	0.99
'CM001015.1'	0.00	-1.00	8.82	0.30	0.80	0.15	6742175	0.08	14.54	0.99
'CM001020.1'	0.00	0.35	17.11	2.19	0.27	0.25	6866064	2.89	11.61	0.95
'CM001040.1'	0.00	-1.00	128.45	2.42	0.31	0.02	47181002	27.54	1.61	0.97
'GL476399.1'	0.00	-1.00	68.14	2.65	0.30	0.04	4695893	22.67	3.11	0.95
'GG697331.1'	0.00	-1.00	118.76	14.43	0.05	0.01	1824042	0.12	6.63	0.79
'GL501517.1'	0.82	0.07	35.66	1.01	0.05	0.01	11292355	8.92	2.84	0.99
'CH236920.1'	0.00	-1.00	34.69	1.46	0.43	0.07	4306251	0.38	5.72	0.95
'CM000694.1'	0.83	0.11	35.46	1.70	0.05	0.02	2314951	0.11	3.10	0.98
'CP003820.1'	0.57	0.13	26.17	1.36	0.13	0.05	2291499	0.00	4.88	0.98
'CH003412.1'	0.03	0.08	96.33	4.95	0.31	0.09	6019272	2.78	2.76	0.97
'CM000138.1'	0.00	-1.00	112.83	1.75	0.19	0.01	42596331	2.49	3.93	0.98
'CH476732.1'	0.43	0.08	76.24	3.20	0.10	0.03	5735491	1.19	2.17	0.97
'GG704911.1'	0.00	-1.00	>1000	>1000	0.01	0.00	8482323	0.00	4.31	0.65
'CH408043.1'	0.76	0.11	40.51	2.26	0.05	0.02	1481020	0.42	2.84	0.97
'CM000040.1'	0.59	0.13	25.15	1.35	0.13	0.05	2297073	1.54	4.93	0.98
'CM000638.1'	0.83	0.12	23.21	1.03	0.07	0.03	3042585	0.37	4.59	0.98
'CH408155.1'	0.65	0.10	31.89	1.50	0.09	0.03	2092950	0.11	3.92	0.98
'CH672346.1'	0.48	0.08	96.25	4.66	0.07	0.02	3194068	0.13	1.67	0.96
'KQ410557.1'	0.59	0.13	26.59	1.49	0.12	0.05	1506362	3.12	4.57	0.98
'CH396049.1'	0.00	-1.00	54.24	1.33	0.37	0.04	11741455	1.71	4.28	0.97
'DS268407.1'	0.58	0.09	43.15	1.77	0.09	0.03	4500979	0.72	3.17	0.98
'CM000578.1'	0.00	-1.00	43.51	1.75	0.36	0.05	6219215	0.16	4.93	0.95
'CH476655.1'	0.00	-1.00	196.27	17.62	0.04	0.01	7343276	7.30	4.17	0.78
'CH476594.1'	0.07	0.15	27.31	1.81	0.43	0.18	2751824	0.39	6.78	0.98
'DS027696.1'	0.00	-1.00	120.17	13.50	0.04	0.01	6287576	0.02	5.54	0.76
'CH671918.1'	0.42	0.09	594.47	186.64	0.04	0.02	377975	1.82	0.66	0.74
'CH981524.1'	0.41	0.09	71.98	3.87	0.11	0.04	3549218	0.27	2.32	0.96
'KE387274.1'	0.00	0.17	30.10	2.16	0.38	0.20	6945667	0.47	7.02	0.97
'DS565998.1'	0.45	0.16	17.19	1.14	0.23	0.09	1244001	5.02	7.25	0.98
'JH159151.1'	0.04	0.14	22.96	1.27	0.49	0.21	13391543	4.48	7.48	0.99
'CH980625.1'	0.37	0.21	31.61	4.67	0.19	0.11	197521	2.23	4.93	0.94

'DS016058.1'	0.64	0.12	>1000	>1000	0.02	0.01	102309	13.07	0.64	0.44
'DS016981.1'	0.00	0.16	41.13	3.90	0.39	0.20	1537740	5.25	5.00	0.96
'KE651166.1'	0.41	0.11	30.19	1.42	0.18	0.06	4291692	3.81	4.63	0.98
'DS022300.1'	0.73	0.08	43.58	1.63	0.06	0.02	4440149	0.35	2.78	0.98
'DS028093.1'	0.00	-1.00	>1000	>1000	0.01	0.00	5707946	0.74	4.83	0.61
'DS178262.1'	0.00	-1.00	282.02	19.28	0.07	0.01	3081398	2.65	3.15	0.87
'CM000589.1'	0.00	-1.00	41.44	1.37	0.41	0.05	6854980	1.94	4.87	0.97
'DS231615.1'	0.33	0.12	24.68	1.12	0.25	0.09	6767051	1.09	6.02	0.99
'KI544492.1'	0.00	-1.00	30.99	1.09	0.35	0.05	6950490	0.52	7.02	0.97
'DS480378.1'	0.68	0.11	79.97	6.76	0.04	0.01	497159	0.14	1.72	0.93
'DS268109.1'	0.00	-1.00	>1000	>1000	0.01	0.00	7870074	0.31	4.60	0.63
'DS268118.1'	0.00	0.16	39.23	3.84	0.43	0.21	1258480	6.18	5.04	0.96
'GG663363.1'	0.00	0.24	190.76	46.58	0.04	0.04	3777229	0.21	4.39	0.71
'DS499594.1'	0.00	-1.00	153.97	20.55	0.04	0.01	4896001	0.37	5.42	0.74
'DS544195.1'	0.00	-1.00	236.76	41.87	0.03	0.01	5372819	1.23	5.08	0.68
'DS544250.1'	0.00	0.15	41.89	3.87	0.39	0.19	1308457	4.83	5.09	0.96
'DS544493.1'	0.04	0.19	37.85	4.83	0.40	0.23	655914	8.44	4.91	0.95
'DS985241.1'	0.78	0.08	45.47	1.47	0.05	0.01	13260704	8.07	2.29	0.98
'DS499746.1'	0.85	0.11	>1000	>1000	0.02	0.01	22989	10.04	0.67	0.22
'DS505602.1'	0.90	0.11	>1000	>1000	0.02	0.01	16341	5.89	0.76	0.15
'DS509930.1'	0.81	0.19	>1000	>1000	0.02	0.01	19991	7.02	0.90	0.16
'DS514921.1'	0.80	0.11	>1000	>1000	0.02	0.01	18390	7.45	0.77	0.38
'DS519693.1'	0.84	0.13	>1000	>1000	0.02	0.01	17581	3.98	0.72	0.26
'DS524649.1'	0.91	0.11	>1000	>1000	0.02	0.01	19127	9.89	0.59	0.12
'DS529121.1'	0.98	0.11	>1000	>1000	0.01	0.00	15451	11.31	0.65	0.08
'KE123623.1'	0.52	0.07	302.94	30.50	0.03	0.01	1441215	12.36	0.57	0.86
'KE123511.1'	0.54	0.06	267.36	16.33	0.03	0.01	3130636	7.91	0.59	0.91
'KN305531.1'	0.00	-1.00	370.77	60.21	0.03	0.01	3651953	0.50	4.07	0.71
'KE503206.1'	0.66	0.10	40.22	1.73	0.07	0.02	4622725	2.71	3.04	0.98
'DS981520.1'	0.10	0.07	166.69	10.46	0.21	0.06	6239366	37.65	1.07	0.95
'DS560032.1'	0.06	0.22	37.43	6.39	0.37	0.22	218953	3.03	4.89	0.94
'DS560667.1'	0.00	-1.00	70.29	8.05	0.26	0.05	226773	5.55	3.80	0.88
'DS561724.1'	0.09	0.20	39.71	6.28	0.34	0.19	207255	2.67	4.56	0.94
'DS562368.1'	0.17	0.19	33.77	4.22	0.31	0.17	385115	4.79	5.07	0.95
'DS572695.1'	0.00	-1.00	26.24	1.04	0.47	0.07	2667998	3.17	7.18	0.97
'KN293992.1'	0.00	-1.00	>1000	507.20	0.03	0.00	2221074	0.76	3.71	0.69
'KN275957.1'	0.00	-1.00	516.39	97.12	0.03	0.00	3931613	1.23	4.04	0.70
'EQ090207.1'	0.00	-1.00	43.60	0.94	0.36	0.03	14321806	3.21	5.48	0.98
'EQ099730.1'	0.00	-1.00	54.22	1.08	0.31	0.02	11967124	1.81	5.04	0.98
'DS572940.1'	0.00	0.23	110.74	59.77	0.26	0.16	39586	3.35	4.25	0.87
'DS985214.1'	0.31	0.14	19.68	1.07	0.29	0.12	4782674	9.04	6.78	0.99
'EQ151724.1'	1.03	0.25	824.71	>1000	0.01	0.01	19632	7.72	0.63	0.04
'EQ169210.1'	0.64	0.61	>1000	>1000	0.03	0.04	17437	0.16	2.31	0.11
'EQ185432.1'	1.07	0.26	632.12	824.88	0.01	0.01	21601	6.93	0.59	0.07
'EQ202275.1'	0.64	0.20	441.90	789.50	0.03	0.02	15371	3.76	1.60	0.47
'EQ220184.1'	0.33	0.17	>1000	>1000	0.09	0.04	21351	3.58	1.78	0.68
'CM000605.1'	0.00	-1.00	37.02	1.97	0.30	0.06	2535400	0.91	6.26	0.93
'DS989822.1'	0.29	0.13	32.41	1.81	0.24	0.10	5365613	0.56	4.80	0.98
'DS990636.1'	0.00	-1.00	>1000	>1000	0.01	0.00	7050398	0.54	3.47	0.66
'GG692419.1'	0.00	-1.00	>1000	>1000	0.01	0.00	6121860	7.01	2.96	0.66
'HF943549.1'	0.11	0.10	48.66	2.37	0.32	0.12	9686481	14.12	3.40	0.98
'DS995701.1'	0.31	0.12	33.86	1.84	0.22	0.08	3830334	0.59	4.60	0.98
'DS995718.1'	0.00	-1.00	58.36	4.33	0.25	0.05	1215537	1.82	4.75	0.90
'GG663735.1'	0.00	-1.00	9.92	0.47	0.49	0.11	2211167	2.07	17.56	0.98
'GG745328.1'	0.00	0.37	31.78	5.71	0.10	0.11	3145054	3.17	11.34	0.88
'KE346360.1'	0.00	-1.00	29.12	0.82	0.42	0.05	3794338	0.91	7.46	0.98
'GG698896.1'	0.16	0.13	28.90	1.59	0.35	0.13	4937060	0.00	5.95	0.98
'GG700648.1'	0.07	0.15	38.20	2.82	0.40	0.20	4087159	1.27	4.71	0.97
'GG698477.1'	0.00	-1.00	43.67	1.89	0.43	0.06	1196472	1.12	4.79	0.96
'CM000924.1'	0.75	0.10	40.81	2.03	0.05	0.02	1489402	0.21	2.85	0.97
'ACYE0100012	0.49	0.19	28.55	3.28	0.15	0.08	265846	0.00	4.88	0.95
'ADAS0200000	0.00	-1.00	211.38	13.94	0.09	0.01	3059345	23.89	2.68	0.88
'KK583189.1'	0.00	0.25	15.09	1.59	0.62	0.38	1615555	3.97	10.65	0.98
'JH379045.1'	0.00	-1.00	63.28	5.92	0.16	0.04	808866	0.64	5.67	0.86
'GG749407.1'	0.00	-1.00	>1000	>1000	0.02	0.00	2378039	7.58	3.26	0.70
'DF090316.1'	0.00	-1.00	77.06	2.20	0.25	0.03	16203812	10.54	3.45	0.96
'FN429986.1'	0.00	-1.00	144.65	6.84	0.16	0.02	2785039	2.03	3.07	0.92
'FN668638.1'	0.00	-1.00	51.85	2.02	0.26	0.03	1966360	0.04	6.01	0.96
'EQ973772.1'	0.25	0.08	142.32	8.04	0.14	0.04	4693355	1.19	1.51	0.95
'CH379587.1'	0.00	0.16	75.21	14.68	0.32	0.16	164660	1.22	4.11	0.93
'DS562855.1'	0.22	0.03	334.11	6.82	0.11	0.02	88675666	1.88	0.78	0.98
'FN330975.1'	0.46	0.09	81.01	4.70	0.09	0.03	1730213	6.63	1.86	0.96
'CM000518.1'	0.51	0.03	327.62	4.64	0.03	0.00	156412533	0.47	0.59	0.98
'DF000001.1'	0.00	-1.00	338.05	70.13	0.23	0.02	174441	7.83	1.84	0.87
'KN525718.1'	0.46	0.04	254.19	5.64	0.05	0.01	43245305	4.89	0.68	0.98
'JH472452.1'	0.00	0.09	287.25	41.24	0.24	0.09	1003560	4.94	1.44	0.91
'KN672347.1'	0.31	0.03	353.34	7.82	0.07	0.01	68039765	5.48	0.66	0.97
'FR853080.2'	0.25	0.03	260.18	4.08	0.11	0.02	228908639	4.42	0.95	0.99
'KN676130.1'	0.47	0.06	182.49	7.56	0.05	0.01	6052217	6.96	0.91	0.96
'CM000438.1'	0.00	-1.00	8.92	0.40	0.53	0.12	3453093	0.83	17.55	0.98
'CM000287.4'	0.47	0.06	315.15	19.31	0.04	0.01	4109689	0.04	0.61	0.91
'CM002299.1'	0.41	0.18	13.77	0.82	0.30	0.14	4351117	0.01	8.63	0.99
'CM000440.1'	0.27	0.05	>1000	>1000	0.04	0.01	2429698	0.06	0.23	0.72
'CM000637.1'	0.38	0.06	345.09	21.51	0.05	0.01	4129288	0.08	0.66	0.92
'CM000661.1'	0.56	0.06	303.10	18.13	0.03	0.01	4078976	0.11	0.56	0.90
'CM000657.1'	0.58	0.06	301.51	17.96	0.02	0.01	3998408	0.12	0.54	0.90
'CM000658.1'	0.51	0.06	311.41	19.03	0.03	0.01	4092698	0.08	0.58	0.91
'CM002917.1'	0.18	0.24	12.94	1.06	0.51	0.30	4468465	0.02	9.97	0.98
'CM000950.1'	0.00	-1.00	10.27	0.65	0.42	0.15	8133379	6.14	13.81	0.95
'CM000951.1'	0.00	-1.00	8.91	0.32	0.84	0.17	9313494	2.77	13.58	0.98
'CM000604.1'	0.45	0.05	329.85	19.57	0.04	0.01	4123727	0.05	0.61	0.92
'CM000659.1'	0.58	0.06	301.98	18.04	0.03	0.01	4016699	0.10	0.55	0.90
'CM000660.1'	0.45	0.06	320.86	20.02	0.04	0.01	3914166	0.11	0.62	0.91
'CM000662.1'	0.00	-1.00	36.14	1.43	0.24	0.04	5578816	0.02	7.25	0.96
'CM000487.1'	0.00	0.12	44.39	2.87	0.41	0.16	4214547	0.00	4.90	0.98
'CM000489.1'	0.00	-1.00	44.59	1.29	0.41	0.04	4187615	0.01	4.89	0.98
'CM000490.1'	0.00	-1.00	44.28	1.28	0.41	0.04	4214643	0.01	4.90	0.98
'CM000953.1'	0.64	0.09	72.40	4.09	0.05	0.02	1604632	0.24	1.88	0.96
'CM000955.1'	0.26	0.12	102.50	9.23	0.14	0.06	1916301	0.43	2.05	0.93
'CM000952.1'	0.59	0.10	53.46	2.69	0.07	0.03	2899588	0.58	2.51	0.97
'CM000770.1'	0.19	0.13	94.13	9.21	0.19	0.09	1823326	5.00	2.20	0.93
'CM001024.1'	0.05	0.31	7.70	0.75	0.87	0.57	3079840	0.05	14.94	0.99
'CM000715.1'	0.31	0.08	58.95	2.57	0.17	0.05	5939023	0.41	2.97	0.98
'CM000716.1'	0.33	0.09	55.54	2.56	0.16	0.05	5730178	0.35	3.08	0.98
'CM000717.1'	0.31	0.10	58.04	2.88	0.17	0.06	5699545	0.27	2.99	0.97
'CM000718.1'	0.41	0.09	49.64	2.23	0.13	0.04	5547057	0.36	3.17	0.98

'CM000719.1'	0.40	0.09	51.79	2.16	0.14	0.04	5674808	0.34	3.09	0.98
'CM000720.1'	0.32	0.10	53.15	2.57	0.17	0.06	5586253	0.41	3.16	0.98
'CM000721.1'	0.29	0.10	54.40	2.76	0.18	0.06	5234797	0.20	3.16	0.97
'CM000722.1'	0.38	0.10	51.29	2.51	0.14	0.05	5246493	0.24	3.09	0.97
'CM000723.1'	0.36	0.10	52.71	2.54	0.15	0.05	5436173	0.34	3.09	0.98
'CM000724.1'	0.36	0.10	51.74	2.43	0.15	0.05	5568034	0.33	3.16	0.98
'CM000725.1'	0.39	0.09	51.84	2.26	0.14	0.04	5576566	0.51	3.10	0.98
'CM000726.1'	0.36	0.10	53.13	2.55	0.15	0.05	5397450	0.22	3.08	0.98
'CM000727.1'	0.32	0.09	54.45	2.45	0.17	0.05	5584055	0.41	3.12	0.98
'CM000728.1'	0.33	0.10	55.87	2.67	0.16	0.05	5859854	0.29	3.04	0.98
'CM000729.1'	0.32	0.09	57.18	2.69	0.17	0.06	5766278	0.35	3.00	0.98
'CM000730.1'	0.24	0.09	57.04	2.74	0.20	0.07	6041497	1.91	3.23	0.98
'CM000731.1'	0.31	0.09	56.90	2.57	0.17	0.05	5878111	0.32	3.04	0.98
'CM000732.1'	0.39	0.09	50.11	2.32	0.14	0.05	5202904	0.37	3.18	0.98
'CM000733.1'	0.44	0.09	46.60	1.91	0.13	0.04	4999804	2.12	3.28	0.98
'CM000734.1'	0.35	0.09	55.82	2.50	0.15	0.05	5769987	0.56	2.98	0.98
'CM000735.1'	0.35	0.09	54.44	2.38	0.15	0.05	5923634	0.30	3.05	0.98
'CM000736.1'	0.32	0.08	59.14	2.54	0.16	0.05	6133179	0.44	2.93	0.98
'CM000737.1'	0.39	0.09	52.83	2.25	0.14	0.04	5799451	0.43	3.06	0.98
'CM000738.1'	0.36	0.10	53.66	2.71	0.15	0.05	5594747	0.57	3.03	0.97
'CM000739.1'	0.33	0.09	53.42	2.52	0.16	0.05	5656704	0.65	3.14	0.98
'CM000740.1'	0.35	0.09	52.96	2.37	0.16	0.05	5789540	0.72	3.10	0.98
'CM000743.1'	0.40	0.09	56.37	2.38	0.13	0.04	5841497	1.05	2.83	0.98
'CM000744.1'	0.39	0.08	56.70	2.38	0.13	0.04	5713954	0.54	2.87	0.98
'CM000745.1'	0.40	0.08	56.33	2.23	0.13	0.04	5782514	0.53	2.85	0.98
'CM000746.1'	0.23	0.10	60.48	3.10	0.21	0.07	5625906	0.39	3.06	0.97
'CM000747.1'	0.37	0.09	54.67	2.47	0.14	0.05	6026843	0.31	2.99	0.98
'CM000748.1'	0.34	0.10	56.54	2.80	0.15	0.05	6323123	0.39	2.96	0.97
'CM000749.1'	0.31	0.10	57.65	2.77	0.17	0.06	6107746	0.79	2.95	0.97
'CM000750.1'	0.34	0.10	54.71	2.72	0.16	0.06	6037513	1.02	3.00	0.97
'CM000751.1'	0.36	0.09	53.90	2.48	0.15	0.05	5527568	0.61	3.04	0.98
'CM000752.1'	0.20	0.09	65.05	3.27	0.22	0.08	6489024	0.39	2.96	0.97
'CM000753.1'	0.33	0.10	57.03	2.85	0.16	0.06	6260142	0.40	2.96	0.97
'CM000754.1'	0.32	0.10	54.30	2.61	0.17	0.06	5488844	0.47	3.13	0.98
'CM000755.1'	0.35	0.09	55.42	2.59	0.15	0.05	6031475	0.31	3.02	0.98
'CM000756.1'	0.26	0.08	63.24	2.86	0.18	0.06	6231196	0.45	2.91	0.98
'CM000757.1'	0.26	0.10	59.63	3.03	0.19	0.07	6002603	0.43	3.04	0.97
'CM000758.1'	0.23	0.09	65.33	3.11	0.20	0.07	6731790	0.36	2.86	0.97
'CM000759.1'	0.34	0.08	58.06	2.39	0.15	0.04	6612432	0.58	2.91	0.98
'CM000776.2'	0.60	0.08	89.10	5.06	0.05	0.02	1623845	0.05	1.62	0.95
'CM000787.2'	0.34	0.22	8.55	0.58	0.49	0.24	4397373	0.31	12.68	0.99
'CM000788.2'	0.34	0.22	8.55	0.58	0.49	0.24	4397242	0.93	12.57	0.99
'CM000789.2'	0.34	0.22	8.55	0.58	0.49	0.24	4397243	0.68	12.61	0.99
'CM000913.1'	0.00	-1.00	8.85	0.32	0.79	0.16	6760392	0.37	14.48	0.98
'CM000854.1'	0.53	0.09	111.35	7.30	0.05	0.02	1702833	1.66	1.38	0.94
'CM000855.1'	0.58	0.08	111.85	6.50	0.05	0.01	1710125	1.38	1.32	0.95
'CM001834.1'	0.08	0.20	16.06	1.17	0.57	0.30	5868202	1.84	8.81	0.98
'CM001149.1'	0.02	0.28	7.73	0.69	0.90	0.53	5259057	0.07	15.36	0.99
'CM000920.1'	0.00	0.22	24.15	2.35	0.31	0.19	3636659	2.46	9.36	0.97
'KN196458.1'	0.37	0.05	539.21	42.78	0.05	0.01	3349855	0.79	0.46	0.89
'KE950501.1'	0.23	0.06	454.22	34.41	0.09	0.02	4032379	0.40	0.67	0.91
'DS362266.1'	0.34	0.07	171.88	12.39	0.09	0.03	1977105	21.67	0.98	0.94
'KB632434.1'	0.16	0.04	311.88	8.72	0.15	0.03	38452041	3.01	0.86	0.97
'DG000025.1'	0.00	-1.00	107.34	1.59	0.20	0.01	45064769	18.15	3.33	0.98
'GL530680.1'	0.06	0.17	88.64	23.41	0.27	0.14	96315	4.22	3.47	0.91
'KI635855.1'	0.44	0.05	111.03	3.21	0.08	0.02	18507834	2.29	1.55	0.98
'CM000436.1'	0.27	0.08	217.05	20.21	0.11	0.04	1344712	0.75	1.10	0.91
'AAGK0100000	0.45	0.09	84.70	4.79	0.09	0.03	2540030	0.00	1.99	0.96
'AAXT0100000	0.50	0.11	39.54	2.05	0.12	0.04	2593320	0.00	3.57	0.98
'CT867985.1'	0.60	0.08	224.02	21.01	0.03	0.01	981684	0.11	0.75	0.87
'CM007668.1'	0.00	0.03	404.92	8.09	0.23	0.04	159902609	2.66	1.05	0.98
'CP000395.1'	0.68	0.09	148.64	13.24	0.03	0.01	905394	0.00	0.97	0.88
'CM001022.1'	0.58	0.28	11.74	1.04	0.24	0.16	2630170	0.00	8.72	0.97
'GL537212.1'	0.59	0.18	20.28	1.82	0.15	0.07	300442	0.01	6.09	0.97
'GL538361.1'	0.00	-1.00	886.77	329.48	0.03	0.01	1422600	2.04	4.18	0.69
'GL541643.1'	0.35	0.17	16.04	1.19	0.29	0.13	867920	3.47	8.34	0.98
'CM001403.1'	0.28	0.12	32.93	1.62	0.24	0.09	8408322	0.01	4.77	0.98
'CM001023.1'	0.79	0.07	53.09	1.79	0.04	0.01	4787724	0.02	2.19	0.98
'CM001025.1'	0.13	0.19	14.29	0.95	0.53	0.26	6270867	0.18	9.76	0.99
'AABW010000(	0.34	0.12	67.02	5.23	0.14	0.06	1250021	0.00	2.69	0.95
'AACF0100000	0.56	0.12	67.71	6.55	0.07	0.03	279155	0.00	2.28	0.94
'AACG020000C	0.68	0.15	38.29	3.42	0.07	0.03	363585	0.00	3.28	0.95
'AACH0100000	0.80	0.23	37.44	8.09	0.05	0.03	45066	0.00	3.26	0.85
'JH797040.1'	0.49	0.23	54.85	15.34	0.10	0.06	54254	0.01	3.25	0.84
'GL985056.1'	0.00	0.28	96.09	19.19	0.05	0.06	3756989	0.09	6.23	0.78
'AAQW010000(	0.00	-1.00	14.15	0.58	0.44	0.09	6492423	0.00	12.09	0.97
'AASL0100000	0.57	0.09	102.91	6.32	0.05	0.02	1616175	0.00	1.46	0.94
'AAXK0100263	0.71	0.16	>1000	>1000	0.03	0.02	10877	0.00	1.27	0.43
'GL636486.1'	0.00	-1.00	>1000	396.62	0.02	0.00	3473653	0.48	4.67	0.64
'ABCY0200000	0.73	0.08	139.52	10.62	0.02	0.01	913294	0.00	0.98	0.91
'ABDF0200000	0.00	-1.00	37.78	1.25	0.45	0.05	3456807	0.00	5.37	0.97
'ABIZ01000001	0.11	0.21	17.40	1.28	0.53	0.31	8220857	0.00	7.88	0.98
'JH597776.1'	0.31	0.15	26.24	1.87	0.25	0.10	1234263	4.55	5.64	0.98
'ABYD0100000	0.78	0.11	33.49	1.76	0.06	0.02	1043181	0.00	3.38	0.98
'ABYE0100000	0.78	0.11	33.62	1.78	0.06	0.02	1043182	0.00	3.38	0.98
'ABYF0100000	0.78	0.10	33.39	1.70	0.06	0.02	1048006	0.00	3.38	0.98
'ABYG0100000	0.78	0.10	33.42	1.72	0.06	0.02	1046064	0.00	3.38	0.98
'ACDA0100504	0.66	0.36	>1000	>1000	0.06	0.05	2193	0.05	3.60	0.31
'ACFJ0100000	0.78	0.10	33.44	1.73	0.06	0.02	1042757	0.00	3.38	0.98
'ACFK0100000	0.53	0.09	109.13	9.04	0.06	0.02	642011	0.00	1.51	0.93
'ACUI0100000	0.74	0.12	33.72	1.96	0.07	0.02	1076624	0.00	3.45	0.97
'ACUJ0100000	0.77	0.10	36.18	1.82	0.06	0.02	1088736	0.02	3.17	0.98
'CM001368.1'	0.00	-1.00	17.39	0.97	0.31	0.08	4102695	0.00	11.26	0.95
'CM001435.1'	0.00	0.14	34.32	2.19	0.49	0.21	5049928	0.01	5.60	0.98
'HE602535.1'	0.02	0.06	106.56	3.65	0.31	0.08	23260604	3.62	2.63	0.98
'ADBW010000(	0.38	0.23	85.83	39.23	0.11	0.07	23943	0.00	3.04	0.80
'AAXM010000C	0.94	0.28	>1000	>1000	0.04	0.03	2670	0.00	1.65	0.06
'CAAE0101458	0.02	0.07	96.80	4.51	0.31	0.08	7611840	10.69	2.62	0.98
'ADBV0100000	0.37	0.18	121.68	42.98	0.09	0.05	62423	0.00	2.23	0.81
'AAXL0100000	0.56	0.36	>1000	>1000	0.07	0.06	2821	0.00	3.40	0.44
'AASU0100786	0.00	0.29	153.87	219.49	0.29	0.19	11390	0.00	4.75	0.83
'AASW010162!	0.00	-1.00	88.46	21.69	0.31	0.06	26222	0.00	4.64	0.88
'AAXN0100000	0.97	0.12	>1000	>1000	0.05	0.02	2132	0.28	1.17	0.06
'KQ034056.1'	0.60	0.06	69.03	2.03	0.06	0.01	13425595	13.29	1.77	0.98
'BABP0100221	0.43	0.12	162.48	30.76	0.07	0.03	202166	0.00	1.40	0.85
'GL739410.1'	0.00	0.10	193.69	33.27	0.26	0.10	470468	2.12	2.04	0.92

'ADMZ0208206	0.20	0.27	>1000	>1000	0.15	0.10	9681	0.00	3.47	0.71
'JH798146.1'	0.18	0.04	297.40	7.31	0.13	0.02	60238417	8.69	0.91	0.98
'GL873520.1'	0.19	0.04	312.19	7.17	0.14	0.03	73491278	3.81	0.79	0.98
'CM001378.2'	0.06	0.03	305.47	5.24	0.21	0.03	240380223	1.26	1.11	0.99
'AAFW0200014	0.75	0.11	40.98	2.18	0.06	0.02	1478136	0.00	2.86	0.97
'GL622787.1'	0.13	0.07	97.09	3.85	0.22	0.06	12041450	2.15	2.57	0.98
'ABLO0201997	0.75	0.29	890.47	>1000	0.05	0.04	3038	0.00	2.37	0.30
'ABPO0100000	0.60	0.19	41.26	6.18	0.08	0.04	137163	0.11	3.41	0.91
'CM002639.1'	0.00	-1.00	109.27	1.59	0.22	0.01	36915442	0.79	3.75	0.98
'ACCZ0100047	0.64	0.36	>1000	>1000	0.07	0.06	2228	0.09	3.05	0.43
'ACFL0100003	0.76	0.14	40.24	3.79	0.05	0.02	224850	0.00	2.97	0.95
'KQ257450.1'	0.58	0.13	24.56	1.36	0.13	0.05	2242449	0.50	5.08	0.98
'CP001408.1'	0.00	0.38	10.52	1.38	0.36	0.31	4098576	0.00	17.70	0.97
'HE605206.1'	0.67	0.09	49.90	2.33	0.06	0.02	3023470	0.00	2.54	0.97
'CABT0200000	0.20	0.13	29.18	1.73	0.31	0.12	3256781	0.40	5.84	0.98
'CP002338.1'	0.41	0.11	50.10	3.13	0.13	0.05	2067702	0.00	3.17	0.97
'CM002236.1'	0.00	-1.00	41.03	0.95	0.40	0.04	9798893	0.11	5.46	0.98
'CP017623.1'	0.52	0.08	93.59	4.64	0.07	0.02	3188341	0.03	1.67	0.96
'ABRE0101798	0.80	0.32	19.66	4.82	0.09	0.06	24044	0.00	5.53	0.89
'AABS0100000	0.00	-1.00	117.18	6.01	0.28	0.03	972361	0.82	2.84	0.94
'CM001487.1'	0.14	0.14	29.94	1.95	0.35	0.14	3384656	0.03	5.77	0.98
'JH712066.1'	0.64	0.10	53.67	3.11	0.06	0.02	1325655	1.53	2.44	0.97
'CM001046.1'	0.19	0.14	44.29	3.27	0.27	0.12	3223176	9.61	3.69	0.96
'CM001048.1'	0.34	0.11	41.28	2.35	0.18	0.07	3115943	7.40	3.58	0.98
'CM001049.1'	0.26	0.12	44.45	2.73	0.22	0.09	2914007	4.07	3.69	0.97
'CM001050.1'	0.43	0.11	43.99	2.34	0.14	0.05	3133385	6.16	3.21	0.98
'CM001051.1'	0.46	0.10	39.95	1.93	0.13	0.04	3140155	4.74	3.48	0.98
'CM001052.1'	0.35	0.10	43.17	2.26	0.17	0.06	2972337	5.51	3.45	0.98
'CM001043.1'	0.35	0.22	8.53	0.58	0.48	0.24	4404672	1.66	12.43	0.99
'CM001044.1'	0.35	0.22	8.50	0.58	0.48	0.24	4404714	1.18	12.51	0.99
'CM001045.1'	0.35	0.22	8.52	0.57	0.48	0.23	4397271	1.19	12.52	0.99
'GL573169.1'	0.00	0.18	42.71	5.50	0.40	0.20	452809	5.11	5.23	0.95
'CM001058.1'	0.00	-1.00	133.21	1.99	0.32	0.02	38222195	5.57	2.08	0.98
'BA000049.1'	0.03	0.13	38.29	2.26	0.44	0.19	6520266	1.90	4.96	0.98
'GL577060.1'	0.00	-1.00	179.02	8.04	0.05	0.01	9559734	8.78	4.66	0.90
'JH801365.1'	0.25	0.04	299.71	7.10	0.11	0.02	72219252	8.67	0.71	0.97
'GL589130.1'	0.38	0.07	181.75	11.18	0.08	0.02	2754868	2.11	1.07	0.94
'GL582980.1'	0.29	0.04	269.51	6.44	0.09	0.02	48689161	4.84	0.84	0.98
'CP000289.1'	0.13	0.20	35.35	3.44	0.36	0.22	2233618	0.21	4.79	0.95
'GG663757.1'	0.31	0.14	>1000	>1000	0.07	0.03	70306	14.52	1.08	0.67
'GG664959.1'	0.60	0.12	751.24	546.01	0.02	0.01	120285	0.00	0.76	0.54
'CM000488.1'	0.00	0.12	44.39	2.87	0.41	0.16	4214598	0.01	4.90	0.98
'KE952971.1'	0.12	0.07	427.84	55.68	0.15	0.04	1452584	2.81	0.92	0.90
'GL637601.1'	0.32	0.08	49.45	1.48	0.18	0.05	33334924	3.43	3.29	0.99
'GL833120.1'	0.00	-1.00	11.84	0.64	0.30	0.08	3520325	2.78	17.61	0.96
'AEUI02000001	0.68	0.11	32.27	1.70	0.08	0.03	1459637	0.25	3.76	0.98
'CM002306.1'	0.54	0.12	28.84	1.60	0.13	0.05	1515570	0.00	4.80	0.98
'GL698470.1'	0.00	0.20	48.84	5.65	0.27	0.17	1836718	4.50	5.25	0.93
'ADNJ0200000	0.18	0.15	26.61	1.54	0.36	0.16	7409503	4.69	5.78	0.98
'CM001062.1'	0.00	-1.00	27.65	1.11	0.30	0.05	4740379	0.02	8.47	0.96
'GL732523.1'	0.00	-1.00	77.38	3.52	0.19	0.03	4193030	3.20	4.30	0.93
'GL738599.1'	0.00	-1.00	83.07	2.79	0.17	0.02	3918663	3.54	5.26	0.96
'AEUT0200000	0.52	0.10	71.45	4.13	0.08	0.03	2164480	0.00	2.15	0.96
'GL764026.1'	0.00	-1.00	79.51	2.45	0.16	0.01	6355204	5.04	5.55	0.96
'JH157811.1'	0.00	-1.00	78.22	2.57	0.23	0.02	5466090	1.02	4.30	0.96
'CM001064.2'	0.00	-1.00	176.18	2.61	0.32	0.02	98543444	12.61	1.38	0.97
'CM001450.1'	0.35	0.05	132.82	3.00	0.10	0.02	51042256	7.85	1.43	0.98
'CM001076.1'	0.64	0.10	47.93	2.38	0.07	0.02	2141837	0.23	2.70	0.97
'GL832955.1'	0.00	-1.00	42.17	1.43	0.31	0.03	2682829	4.24	6.30	0.97
'GL849905.1'	0.23	0.05	621.78	46.09	0.09	0.02	5315331	4.05	0.48	0.91
'GG662845.1'	0.51	0.06	341.31	26.69	0.03	0.01	2216158	0.09	0.56	0.88
'CM001081.1'	0.77	0.11	40.17	2.12	0.05	0.02	1442732	0.05	2.85	0.97
'CM001097.1'	0.75	0.10	41.14	2.09	0.05	0.02	1453315	0.08	2.84	0.97
'CM001129.1'	0.79	0.11	39.81	2.10	0.05	0.02	1422509	0.08	2.84	0.97
'CM001113.1'	0.76	0.11	40.89	2.28	0.05	0.02	1448153	0.05	2.82	0.97
'GL870876.1'	0.28	0.10	126.60	12.09	0.13	0.05	858724	0.42	1.68	0.93
'GL870941.1'	0.45	0.11	355.39	100.09	0.04	0.02	285244	0.07	0.89	0.74
'CM001142.1'	0.00	-1.00	27.91	0.90	0.44	0.06	4509864	0.13	6.97	0.98
'CM001148.1'	0.16	0.20	19.08	1.63	0.41	0.22	1891448	0.01	7.98	0.98
'CM001151.1'	0.00	-1.00	28.52	1.15	0.28	0.05	4842911	0.01	8.46	0.96
'CM001153.1'	0.00	-1.00	28.90	1.25	0.27	0.05	4658698	0.00	8.51	0.96
'GL872453.1'	0.65	0.20	47.98	8.90	0.07	0.04	93422	23.40	2.24	0.88
'GL873277.1'	0.70	0.16	42.94	4.77	0.06	0.03	242186	4.85	2.79	0.93
'GL875079.1'	0.58	0.21	51.09	10.39	0.08	0.04	94104	10.13	2.63	0.86
'GL875736.1'	0.68	0.16	45.49	5.36	0.06	0.03	193407	5.07	2.72	0.92
'GL875992.1'	0.57	0.17	50.68	8.13	0.08	0.04	108962	6.58	2.82	0.90
'GL876586.1'	0.59	0.16	50.62	6.64	0.08	0.04	179539	5.40	2.68	0.92
'GL877404.1'	0.86	0.14	29.10	2.33	0.05	0.02	243087	0.01	3.66	0.96
'GL877354.1'	0.00	-1.00	74.04	10.41	0.05	0.02	1895500	0.43	8.75	0.78
'GL876966.1'	0.04	0.14	22.67	1.30	0.49	0.19	6150421	11.59	6.99	0.99
'CM000832.1'	0.00	-1.00	10.18	0.49	0.39	0.10	4118977	0.09	17.66	0.97
'CM000774.1'	0.00	-1.00	9.27	0.45	0.47	0.12	3976928	0.04	17.90	0.97
'CM001156.1'	0.00	-1.00	9.41	0.50	0.46	0.12	3983232	2.14	17.47	0.97
'FP885897.1'	0.00	-1.00	10.57	0.37	0.72	0.12	3417386	0.03	13.26	0.99
'GL882879.1'	0.73	0.08	43.22	1.60	0.06	0.02	4429270	0.18	2.80	0.98
'GL883010.1'	0.04	0.06	182.42	10.79	0.23	0.06	4166812	0.00	1.89	0.96
'GL883090.1'	0.00	0.11	205.43	21.83	0.12	0.06	4071029	3.21	2.70	0.89
'GL888128.1'	0.00	-1.00	84.69	2.09	0.23	0.02	5247136	0.85	4.57	0.98
'DS891538.1'	0.13	0.10	38.26	1.89	0.32	0.10	3952103	6.40	4.89	0.99
'JH573670.1'	0.24	0.04	424.39	17.12	0.09	0.02	14349515	4.16	0.64	0.95
'DF145384.1'	0.19	0.11	182.86	31.83	0.15	0.06	277264	0.10	1.69	0.90
'LT594789.1'	0.07	0.05	203.67	6.27	0.24	0.05	41236440	4.92	1.33	0.97
'FR823391.1'	0.00	-1.00	28.06	1.10	0.32	0.06	6985512	0.04	7.94	0.96
'DS571145.1'	0.53	0.09	540.73	125.47	0.03	0.01	530629	0.85	0.55	0.72
'CH473309.1'	0.10	0.13	37.45	3.08	0.34	0.14	990720	0.40	5.44	0.97
'DS548800.1'	0.67	0.09	347.06	68.57	0.02	0.01	339000	0.77	0.64	0.71
'CM000437.1'	0.62	0.10	42.32	2.03	0.08	0.03	3510247	0.00	3.11	0.98
'DS231813.1'	0.00	0.11	71.55	4.98	0.34	0.13	3873040	1.65	3.78	0.97
'DS469507.1'	0.00	-1.00	138.07	7.27	0.15	0.02	3256212	6.15	3.12	0.90
'GL889000.1'	0.83	0.10	38.51	2.12	0.04	0.01	898533	2.84	2.79	0.97
'GL889064.1'	0.81	0.12	39.36	2.53	0.05	0.02	900937	2.67	2.79	0.96
'GL889345.1'	0.77	0.15	41.56	4.64	0.05	0.02	145354	1.25	2.85	0.93
'GL889895.1'	0.62	0.25	66.75	28.82	0.06	0.04	30196	35.18	1.91	0.76
'GL890460.1'	0.73	0.11	41.72	2.59	0.06	0.02	972418	0.05	2.87	0.97
'CH991540.1'	0.47	0.12	189.51	38.06	0.05	0.02	204096	0.20	1.24	0.83
'FN653015.1'	0.43	0.10	32.95	1.58	0.16	0.05	3167015	1.91	4.60	0.98

'FN654275.1'	0.30	0.17	39.50	5.21	0.19	0.09	269355	5.00	4.39	0.95
'BX526834.1'	0.39	0.09	166.28	15.62	0.08	0.03	1164703	0.05	1.19	0.91
'AP009608.1'	0.46	0.08	212.76	20.64	0.05	0.02	1004014	0.20	0.93	0.89
'FP929036.1'	0.66	0.09	61.72	3.06	0.05	0.02	3164379	1.50	2.10	0.96
'FP929053.1'	0.40	0.12	38.32	2.16	0.15	0.06	3545606	0.96	3.87	0.97
'FP929046.1'	0.07	0.15	24.32	1.64	0.45	0.19	3214418	1.51	6.99	0.98
'FP929051.1'	0.06	0.17	43.32	4.11	0.34	0.19	2249085	0.80	4.60	0.95
'FP929062.1'	0.00	-1.00	36.64	1.15	0.38	0.04	3601020	4.00	5.88	0.98
'FP929044.1'	0.48	0.19	22.69	1.72	0.15	0.08	2943413	2.69	5.78	0.97
'FP929042.1'	0.41	0.10	50.01	2.59	0.13	0.05	3344951	2.57	3.08	0.97
'FP929043.1'	0.36	0.10	54.67	2.94	0.14	0.05	3698419	3.56	2.90	0.97
'FP929048.1'	0.46	0.09	97.35	5.72	0.08	0.02	2209938	3.60	1.67	0.95
'FP929049.1'	0.21	0.12	42.85	2.59	0.25	0.10	4143550	2.00	4.15	0.97
'FP929054.1'	0.28	0.12	42.17	2.52	0.21	0.08	3757491	1.74	3.97	0.97
'FP929055.1'	0.47	0.12	42.42	2.45	0.12	0.05	3341681	1.91	3.34	0.97
'FP929047.1'	0.00	-1.00	12.34	0.46	0.56	0.10	3608022	7.72	11.93	0.98
'FP929033.1'	0.03	0.11	46.56	2.54	0.40	0.15	5976145	2.93	4.34	0.98
'FP929052.1'	0.61	0.19	20.31	1.43	0.12	0.07	2573208	2.32	5.77	0.97
'FP929058.1'	0.52	0.10	40.09	1.93	0.11	0.04	3096657	5.20	3.29	0.98
'FN554974.1'	0.00	0.14	37.56	2.67	0.47	0.22	4531609	0.10	5.33	0.97
'FN645450.1'	0.00	-1.00	85.09	3.81	0.25	0.03	1266078	0.33	4.06	0.95
'AP011615.1'	0.48	0.12	34.69	1.78	0.13	0.05	6788435	1.41	4.06	0.98
'FN668375.1'	0.36	0.06	329.55	20.55	0.06	0.02	4308325	0.04	0.69	0.92
'FP929037.1'	0.44	0.13	27.86	1.47	0.17	0.07	3769775	4.27	4.79	0.98
'FP929038.1'	0.41	0.12	39.04	2.21	0.15	0.06	3522704	1.68	3.77	0.97
'FP929032.1'	0.00	0.26	27.20	3.31	0.17	0.14	3763317	3.02	10.61	0.94
'FP929039.1'	0.49	0.13	39.49	2.50	0.12	0.05	3123007	7.52	3.35	0.97
'FP929041.1'	0.52	0.10	52.52	2.95	0.09	0.03	1966750	15.54	2.42	0.97
'FP929059.1'	0.38	0.22	23.36	2.17	0.19	0.12	2836123	2.01	5.91	0.96
'FP929050.1'	0.17	0.11	43.68	2.38	0.28	0.10	4286292	2.83	4.15	0.98
'FP929060.1'	0.51	0.14	26.61	1.55	0.15	0.06	3108859	21.48	3.90	0.98
'FP929061.1'	0.54	0.09	55.88	2.61	0.08	0.03	3114788	2.05	2.53	0.97
'FP929056.1'	0.00	-1.00	18.95	0.83	0.42	0.08	2728333	9.95	9.28	0.97
'FP929045.1'	0.00	-1.00	24.74	0.90	0.45	0.07	3321367	2.20	7.60	0.97
'FP929034.1'	0.00	-1.00	14.86	0.57	0.61	0.10	2384987	0.52	10.50	0.98
'FP929040.1'	0.00	-1.00	22.60	0.84	0.39	0.06	4908759	5.19	8.38	0.97
'FR720325.1'	0.20	0.13	44.03	3.04	0.26	0.11	2936689	0.00	4.05	0.97
'FQ482149.1'	0.76	0.11	38.30	2.15	0.06	0.02	1164076	0.59	3.00	0.97
'BA000015.5'	0.10	0.06	105.60	3.66	0.25	0.07	23810767	0.00	2.34	0.98
'ADNL0100321	0.75	0.21	>1000	>1000	0.03	0.02	13634	0.00	1.15	0.26
'CM001159.1'	0.21	0.12	44.91	2.89	0.25	0.10	3021822	0.02	4.00	0.97
'CM001161.1'	0.03	0.29	8.63	0.83	0.86	0.54	3139378	0.00	13.96	0.99
'CM001167.1'	0.64	0.08	71.47	3.41	0.05	0.02	2991798	0.00	1.90	0.97
'CM000492.1'	0.25	0.03	279.09	4.30	0.11	0.02	234808386	0.61	0.89	0.99
'CM001165.1'	0.00	-1.00	8.55	0.37	0.64	0.16	7359380	0.00	16.08	0.98
'GL891382.1'	0.00	-1.00	47.36	1.52	0.28	0.04	9915204	1.51	5.39	0.96
'GL890999.1'	0.00	0.13	41.03	2.42	0.36	0.16	9465266	2.45	5.45	0.98
'CM001168.1'	0.69	0.13	36.46	2.32	0.07	0.03	1143519	0.72	3.25	0.97
'CAID0100000'	0.25	0.23	10.30	0.88	0.46	0.24	1096037	0.07	13.59	0.99
'CM001175.1'	0.00	0.12	88.68	5.97	0.12	0.06	18145390	4.09	5.12	0.95
'CM001475.1'	0.00	0.24	21.71	2.13	0.29	0.20	4446267	0.00	10.38	0.96
'GL896898.1'	0.12	0.04	339.66	8.95	0.17	0.03	52375790	3.60	0.83	0.97
'GL905323.1'	0.00	-1.00	67.92	2.11	0.17	0.02	7770198	2.74	6.09	0.96
'GL913935.1'	0.18	0.10	164.99	22.21	0.16	0.06	504747	0.08	1.76	0.92
'GL931709.1'	0.24	0.10	135.92	15.62	0.14	0.05	535469	0.05	1.87	0.93
'GL921761.1'	0.20	0.10	154.94	21.43	0.15	0.06	502403	0.03	1.82	0.91
'CM001187.1'	0.02	0.07	105.18	3.97	0.33	0.09	19836964	0.00	2.46	0.98
'CM002368.1'	0.19	0.05	>1000	>1000	0.06	0.02	2154007	0.01	0.31	0.75
'GL945428.1'	0.00	-1.00	236.21	9.73	0.14	0.01	5733305	0.55	2.34	0.92
'GL945474.1'	0.00	-1.00	236.12	9.89	0.15	0.01	5619144	10.97	1.98	0.92
'FN393063.1'	0.76	0.11	40.69	2.23	0.05	0.02	1376149	0.05	2.84	0.97
'EQ258278.1'	0.56	0.18	14.12	0.94	0.21	0.10	1568541	3.24	7.70	0.98
'CM001194.1'	0.20	0.13	44.60	2.91	0.26	0.11	3184320	0.14	3.90	0.97
'CM001220.2'	0.00	0.06	177.51	5.76	0.33	0.09	56582383	3.62	1.49	0.97
'CM001196.1'	0.00	-1.00	74.52	14.98	0.04	0.02	6088797	0.00	7.16	0.65
'CM001195.1'	0.12	0.23	9.94	0.74	0.67	0.37	4336393	0.24	12.45	0.99
'GL982313.1'	0.12	0.05	>1000	283.46	0.12	0.03	2942792	4.23	0.43	0.86
'AGTD0100000	0.00	0.16	28.64	2.09	0.38	0.19	5239207	0.00	7.37	0.98
'GL983042.1'	0.42	0.08	>1000	>1000	0.03	0.01	373042	0.12	0.51	0.67
'CM001225.1'	0.36	0.22	8.51	0.57	0.48	0.23	4342996	0.46	12.56	0.99
'CM001226.1'	0.35	0.22	8.51	0.57	0.48	0.24	4364777	0.35	12.60	0.99
'CM001227.1'	0.35	0.22	8.51	0.57	0.48	0.24	4342459	0.26	12.61	0.99
'KB457523.1'	0.00	-1.00	72.61	2.22	0.29	0.03	3722508	0.01	4.26	0.97
'GL985818.1'	0.00	-1.00	156.74	10.73	0.04	0.01	5876085	1.67	6.46	0.85
'GL988041.1'	0.15	0.11	31.34	1.43	0.33	0.11	6909506	0.02	5.82	0.99
'FR874244.1'	0.20	0.03	267.67	4.37	0.13	0.02	229594800	9.09	0.91	0.99
'AEVR0200001	0.54	0.18	9.44	0.56	0.31	0.12	2200242	0.05	11.15	0.99
'GL990379.1'	0.30	0.10	80.67	6.24	0.14	0.05	1122115	3.48	2.42	0.95
'GL992820.1'	0.28	0.09	84.32	5.26	0.15	0.05	2181227	2.61	2.34	0.96
'GL991508.1'	0.30	0.10	79.60	6.15	0.14	0.05	1073948	1.92	2.48	0.95
'GL995175.1'	0.24	0.09	84.87	5.99	0.17	0.06	1480773	3.10	2.54	0.96
'CM001228.1'	0.33	0.13	57.50	4.45	0.15	0.07	1874244	0.82	2.95	0.95
'DF970692.1'	0.00	0.11	158.44	16.66	0.17	0.08	2549845	10.40	2.50	0.92
'JH000001.1'	0.43	0.05	404.38	18.07	0.04	0.01	8779783	1.31	0.49	0.93
'GL996469.1'	0.66	0.09	57.84	2.85	0.06	0.02	2228510	3.83	2.22	0.97
'GL996527.1'	0.49	0.11	43.67	2.26	0.11	0.04	2534490	1.53	3.32	0.97
'GL996499.1'	0.57	0.09	61.95	3.04	0.07	0.02	2640565	0.17	2.27	0.97
'AECO0100019	0.16	0.25	>1000	>1000	0.15	0.09	13198	0.00	3.05	0.73
'FR839628.1'	0.80	0.10	40.05	1.78	0.05	0.02	2891190	0.24	2.78	0.98
'KK211454.1'	0.00	-1.00	27.21	1.49	0.48	0.08	711979	1.74	7.11	0.96
'AFXJ0100000'	0.00	-1.00	14.80	0.65	0.40	0.09	6742964	0.00	12.01	0.97
'AFXK0100000	0.00	-1.00	14.40	0.61	0.43	0.09	6719211	0.00	12.01	0.97
'JH113294.1'	0.24	0.09	158.29	14.43	0.13	0.05	2536880	48.18	0.77	0.93
'HT000001.2'	0.00	-1.00	113.80	2.30	0.30	0.02	10041005	3.47	2.61	0.98
'KB458607.1'	0.00	0.08	95.22	4.81	0.27	0.08	7425378	0.01	3.51	0.98
'KB459691.1'	0.00	-1.00	85.14	2.85	0.24	0.02	3555979	0.01	4.04	0.96
'KB461572.1'	0.00	-1.00	88.42	3.01	0.25	0.02	2419304	0.03	3.89	0.96
'KN039985.1'	0.00	-1.00	30.53	1.06	0.37	0.05	4825539	2.20	6.90	0.97
'KN042471.1'	0.00	-1.00	28.39	0.98	0.42	0.06	4029688	1.49	7.00	0.97
'KN044612.1'	0.00	-1.00	28.48	0.94	0.44	0.06	4468542	2.86	6.84	0.97
'KN000222.1'	0.00	-1.00	28.26	0.89	0.44	0.06	6845168	2.43	6.92	0.97
'KN000235.1'	0.00	-1.00	28.70	0.89	0.43	0.05	5443361	3.07	6.85	0.98
'JH119053.1'	0.45	0.11	30.49	1.50	0.16	0.06	3323103	0.48	4.66	0.98
'CM001230.1'	0.00	-1.00	27.04	1.23	0.23	0.04	4943773	0.02	9.54	0.95
'JH126399.1'	0.00	-1.00	>1000	>1000	0.01	0.00	6263243	0.15	6.76	0.59
'JH126562.1'	0.20	0.06	177.41	7.45	0.15	0.04	10736886	14.05	1.22	0.97

'JH154184.1'	0.00	-1.00	85.26	3.68	0.35	0.04	1222598	2.38	2.98	0.95
'JH137791.1'	0.02	0.07	164.52	9.24	0.29	0.09	7090834	5.37	1.64	0.96
'CP003002.1'	0.00	-1.00	86.55	4.93	0.06	0.01	10931058	0.00	6.94	0.88
'CP003009.1'	0.00	-1.00	50.06	1.91	0.16	0.02	10101509	0.00	7.32	0.95
'KV751188.1'	0.00	0.17	912.85	517.51	0.02	0.02	1615270	1.44	4.56	0.71
'AM920437.1'	0.01	0.11	38.42	2.06	0.45	0.17	6387817	0.05	5.25	0.98
'CU640366.1'	0.13	0.13	33.55	2.03	0.34	0.14	4657536	0.92	5.44	0.98
'DF093573.1'	0.29	0.11	31.98	1.57	0.24	0.08	4559051	0.75	5.01	0.98
'FQ790278.1'	0.47	0.13	35.56	2.27	0.14	0.05	1469722	2.34	3.92	0.97
'HE580267.1'	0.69	0.08	58.20	2.50	0.05	0.01	2054635	0.27	2.19	0.98
'FR799623.2'	0.34	0.16	16.14	1.05	0.31	0.13	2713248	1.81	8.47	0.99
'CAEQ0100117	0.43	0.23	29.13	5.20	0.17	0.09	67719	0.00	5.18	0.93
'FQ311430.1'	0.23	0.18	14.26	1.02	0.42	0.19	2448206	1.90	9.62	0.99
'FP929139.1'	0.00	0.14	120.14	13.38	0.13	0.07	4258568	1.00	4.35	0.90
'JH173571.1'	0.23	0.05	377.46	21.72	0.10	0.03	7787482	7.31	0.64	0.93
'JH208680.1'	0.18	0.05	270.62	10.77	0.13	0.03	16024077	2.79	1.09	0.96
'JH226299.1'	0.15	0.09	228.40	36.53	0.16	0.05	556907	19.33	1.20	0.91
'AFBI03000001	0.64	0.08	108.52	5.84	0.04	0.01	1667150	0.52	1.28	0.95
'JH226130.1'	0.48	0.13	21.59	1.07	0.19	0.07	4245248	0.01	6.11	0.99
'CM001253.1'	0.18	0.03	260.04	4.22	0.14	0.02	229590362	10.51	0.94	0.99
'CM001276.1'	0.20	0.03	262.88	4.38	0.13	0.02	232296185	19.64	0.81	0.99
'CM001240.1'	0.65	0.06	132.98	4.92	0.03	0.01	6477357	0.00	1.06	0.96
'CM001274.1'	0.00	-1.00	31.00	1.39	0.24	0.04	4878746	0.01	8.47	0.95
'CM001466.1'	0.00	0.32	7.75	0.76	0.93	0.63	4763852	0.20	15.19	0.99
'CM001241.2'	0.00	-1.00	110.06	1.67	0.24	0.01	33916305	5.46	3.29	0.98
'JH370130.1'	0.88	0.13	49.49	4.26	0.03	0.01	368097	1.04	2.14	0.93
'HE569754.1'	0.17	0.08	78.76	3.90	0.22	0.06	4999318	28.62	2.12	0.98
'JH720293.1'	0.55	0.05	427.45	28.03	0.02	0.01	3762730	0.40	0.42	0.88
'JH719946.1'	0.50	0.07	344.23	29.28	0.03	0.01	2173071	1.83	0.55	0.87
'CM003279.1'	0.00	-1.00	315.98	7.90	0.07	0.01	159038749	4.83	1.81	0.90
'KB462838.1'	0.00	-1.00	80.66	1.77	0.26	0.02	10656099	0.01	4.04	0.98
'CM001488.1'	0.45	0.13	29.81	1.58	0.16	0.06	3974658	0.06	4.64	0.98
'JH376955.1'	0.71	0.13	41.07	2.96	0.06	0.03	961215	7.00	2.74	0.96
'FR799573.1'	0.29	0.15	17.06	0.98	0.33	0.13	3343498	0.13	8.44	0.99
'CP002498.1'	0.70	0.10	40.19	1.95	0.06	0.02	1601921	0.00	3.07	0.98
'JH378105.1'	0.17	0.04	296.20	7.00	0.14	0.03	72162052	4.52	0.89	0.98
'JH392917.1'	0.48	0.18	25.31	2.68	0.16	0.08	227101	1.19	5.44	0.96
'JH379599.1'	0.11	0.10	86.26	5.23	0.26	0.09	3946504	1.13	2.64	0.96
'CM001371.1'	0.70	0.09	39.23	1.53	0.07	0.02	3635360	0.32	3.08	0.98
'CM001373.1'	0.72	0.09	38.59	1.48	0.06	0.02	3689573	0.19	3.09	0.98
'JH393279.1'	0.40	0.03	436.50	9.23	0.05	0.01	58279134	1.97	0.46	0.97
'KB464369.1'	0.00	-1.00	67.64	1.56	0.35	0.03	5555046	0.03	3.94	0.98
'KB452396.1'	0.00	-1.00	85.11	2.97	0.27	0.02	1708276	0.29	3.89	0.97
'KB465221.1'	0.00	0.08	78.34	3.75	0.33	0.10	7884553	0.00	3.57	0.98
'DF142838.1'	0.80	0.11	26.90	1.17	0.07	0.02	2050842	0.02	4.02	0.98
'CP003147.1'	0.44	0.13	20.79	1.01	0.21	0.08	5475670	0.00	6.17	0.99
'HE612856.1'	0.67	0.09	70.51	3.87	0.05	0.01	1377933	0.07	1.92	0.96
'FR871757.1'	0.58	0.12	37.68	2.31	0.10	0.04	1755458	0.52	3.49	0.97
'HE576752.1'	0.69	0.09	56.49	2.47	0.05	0.02	3019861	0.00	2.28	0.97
'HE577327.1'	0.00	-1.00	10.40	0.50	0.69	0.16	3023440	0.06	13.38	0.97
'CM001376.1'	0.00	-1.00	18.89	1.06	0.38	0.09	1675934	0.02	10.38	0.95
'CM001377.1'	0.63	0.16	19.39	1.25	0.14	0.06	1880838	0.01	5.97	0.98
'HE601624.1'	0.24	0.05	83.64	1.97	0.18	0.04	65476681	0.51	2.32	0.99
'JH413862.1'	0.40	0.13	30.23	1.80	0.18	0.07	2399772	0.19	4.81	0.98
'JH556617.1'	0.53	0.03	268.08	5.66	0.03	0.01	39887635	0.56	0.65	0.98
'AGTA0300000	0.37	0.05	139.07	4.31	0.09	0.02	14997410	2.17	1.41	0.98
'JH417973.1'	0.00	-1.00	226.58	4.08	0.19	0.01	38702089	2.02	1.90	0.97
'JH419206.1'	0.30	0.06	120.64	4.20	0.12	0.03	12616905	13.50	1.51	0.98
'JH422273.1'	0.41	0.05	131.94	3.77	0.08	0.02	23840500	9.86	1.29	0.98
'JH425323.1'	0.37	0.06	126.20	5.02	0.09	0.02	7036168	8.41	1.43	0.97
'HE587301.1'	0.45	0.07	100.41	3.87	0.08	0.02	7801961	0.66	1.64	0.97
'JH432010.1'	0.18	0.10	85.58	6.17	0.21	0.07	1344237	0.14	2.68	0.96
'KN955244.1'	0.19	0.12	103.71	12.13	0.18	0.07	551686	0.76	2.29	0.93
'DF126447.1'	0.00	0.12	39.45	2.64	0.44	0.17	2077433	0.42	5.44	0.98
'CM000574.1'	0.15	0.13	35.16	1.84	0.34	0.14	11697295	0.59	4.88	0.98
'JH470562.1'	0.68	0.19	18.46	1.77	0.13	0.06	233313	3.45	6.07	0.97
'CM001398.1'	0.51	0.14	28.97	1.83	0.14	0.06	1833925	0.01	4.66	0.98
'JH556661.1'	0.33	0.06	160.71	6.29	0.09	0.02	7907290	7.76	1.26	0.97
'AGTM0115263	0.42	0.11	>1000	>1000	0.04	0.02	86436	0.06	0.87	0.62
'CM002655.1'	0.30	0.04	213.05	4.17	0.09	0.02	77392008	8.54	1.04	0.98
'CM001400.1'	0.58	0.11	37.35	1.93	0.10	0.04	2846739	0.03	3.47	0.98
'CM001402.1'	0.00	0.18	40.43	3.48	0.29	0.17	4955439	0.00	6.16	0.96
'CP003244.1'	0.00	-1.00	27.42	1.12	0.36	0.06	4861101	0.00	7.73	0.96
'CM001407.1'	0.11	0.04	228.45	5.07	0.19	0.03	73198282	8.31	1.26	0.98
'AFRZ0100000	0.61	0.08	92.84	4.49	0.05	0.01	2952682	0.00	1.52	0.96
'CM001437.1'	0.16	0.11	68.00	4.36	0.25	0.10	4301842	0.12	2.85	0.96
'JH594607.1'	0.30	0.04	177.07	4.18	0.11	0.02	45942467	7.37	1.11	0.98
'HE616742.1'	0.92	0.11	29.61	1.37	0.04	0.01	1418021	0.02	3.44	0.98
'CM001484.1'	0.00	0.30	8.07	0.77	0.94	0.60	4538746	0.02	14.52	0.99
'CM001441.1'	0.33	0.12	37.73	1.98	0.20	0.08	5660978	0.01	4.17	0.98
'CM001442.1'	0.32	0.20	8.91	0.52	0.49	0.23	7784016	0.00	12.89	0.99
'CM001439.1'	0.00	-1.00	8.47	0.34	0.92	0.20	5965593	0.01	14.04	0.98
'CM001440.1'	0.00	-1.00	8.17	0.33	0.87	0.19	5408301	0.01	14.68	0.98
'CM001438.1'	0.56	0.17	8.74	0.46	0.32	0.13	5600627	0.00	11.86	0.99
'CP002374.1'	0.80	0.15	13.72	0.73	0.13	0.05	1139330	0.02	7.51	0.99
'CP002375.1'	0.80	0.15	13.73	0.74	0.13	0.05	1139744	0.01	7.51	0.99
'CP002376.1'	0.81	0.16	13.63	0.77	0.12	0.05	1139417	0.01	7.51	0.99
'CP003115.1'	0.80	0.15	13.74	0.75	0.13	0.05	1139971	0.01	7.51	0.99
'KI669561.1'	0.41	0.12	22.43	1.08	0.21	0.07	4724858	30.86	4.38	0.99
'JH602043.1'	0.20	0.03	406.75	9.49	0.11	0.02	78885850	11.81	0.65	0.97
'CM003021.1'	0.22	0.03	345.56	6.47	0.11	0.02	161108492	7.16	0.72	0.98
'CM001990.1'	0.28	0.03	428.69	7.98	0.08	0.01	157563209	0.51	0.56	0.97
'JH604633.1'	0.33	0.10	109.77	9.64	0.11	0.04	963716	0.66	1.78	0.93
'CM001469.1'	0.23	0.13	42.87	2.93	0.24	0.10	2776529	1.27	3.98	0.97
'JH605123.1'	0.40	0.16	15.86	0.96	0.27	0.11	2648581	0.22	8.58	0.99
'KQ973609.1'	0.00	-1.00	32.12	1.10	0.35	0.05	6392340	3.05	6.77	0.97
'JH605023.1'	0.26	0.10	121.65	11.09	0.14	0.05	954566	0.47	1.74	0.93
'CM001471.1'	0.00	-1.00	33.45	1.58	0.22	0.04	4672567	0.01	8.27	0.95
'CM000492.1'	0.25	0.03	279.09	4.30	0.11	0.02	234808386	0.61	0.89	0.99
'CM001474.1'	0.00	-1.00	28.17	1.06	0.37	0.06	4580866	0.26	7.39	0.97
'FN667742.1'	0.00	0.13	50.79	3.65	0.36	0.16	4432590	0.00	4.62	0.97
'FR668087.1'	0.27	0.06	>1000	762.20	0.06	0.02	1017232	0.05	0.43	0.77
'FP475956.1'	0.00	-1.00	12.77	0.44	0.71	0.12	3738778	0.21	11.30	0.98
'FR845719.1'	0.00	-1.00	8.31	0.31	0.80	0.18	8226158	0.11	15.10	0.98
'JH621224.1'	0.80	0.12	13.13	0.53	0.13	0.04	3231181	0.08	7.74	0.99
'CM001467.1'	0.17	0.12	82.56	7.16	0.21	0.09	1369927	0.01	2.62	0.95

'CM001477.1'	0.42	0.06	599.49	56.88	0.03	0.01	3333039	0.12	0.42	0.84
'JH622822.1'	0.47	0.24	52.70	16.24	0.10	0.07	53808	17.82	2.88	0.84
'KQ983268.1'	0.00	-1.00	27.39	0.89	0.47	0.06	4176651	1.62	6.94	0.97
'JH624252.1'	0.37	0.25	94.78	57.30	0.11	0.07	53928	58.15	1.34	0.77
'JH625538.1'	0.41	0.27	54.05	24.67	0.12	0.08	53242	67.44	1.39	0.83
'JH626928.1'	0.48	0.23	76.14	34.07	0.09	0.05	54075	54.66	1.40	0.79
'JH631522.1'	0.63	0.24	60.01	21.30	0.06	0.04	54222	53.65	1.40	0.79
'JH630079.1'	0.33	0.27	79.88	39.49	0.12	0.09	53749	28.55	2.27	0.77
'JH632993.1'	0.37	0.27	65.70	27.62	0.12	0.09	54043	26.42	2.47	0.79
'JH628500.1'	0.45	0.23	59.77	18.16	0.10	0.06	54221	16.41	2.71	0.84
'JH638420.1'	0.34	0.23	65.33	22.74	0.13	0.08	53678	31.30	2.38	0.85
'JH635991.1'	0.40	0.26	43.33	13.78	0.14	0.09	53414	34.65	2.90	0.87
'JH634502.1'	0.61	0.22	52.94	14.32	0.07	0.04	54218	41.59	1.82	0.84
'JH649412.1'	0.42	0.26	90.55	56.11	0.10	0.06	54210	63.73	1.13	0.75
'JH648157.1'	0.51	0.24	52.42	14.86	0.09	0.06	54040	16.29	2.77	0.84
'JH646584.1'	0.49	0.23	55.08	16.27	0.09	0.06	54216	22.84	2.55	0.84
'JH645006.1'	0.49	0.23	61.77	19.76	0.09	0.05	54217	31.86	2.17	0.83
'JH641959.1'	0.55	0.23	48.15	11.76	0.09	0.05	54223	0.94	3.26	0.86
'JH641353.1'	0.47	0.22	59.50	16.43	0.10	0.06	54222	11.29	2.82	0.85
'JH643483.1'	0.57	0.23	47.44	11.44	0.08	0.05	54057	13.63	2.89	0.86
'JH639923.1'	0.48	0.22	53.57	13.87	0.10	0.06	54133	10.13	3.01	0.86
'JH929243.1'	0.88	0.10	966.72	677.66	0.01	0.00	97841	0.02	0.52	0.27
'KL544027.1'	0.00	0.18	25.82	1.98	0.50	0.26	4641914	0.26	7.14	0.97
'CP012110.1'	0.00	-1.00	33.69	1.13	0.40	0.05	4286821	0.02	6.40	0.97
'CM003385.1'	0.31	0.03	281.01	4.97	0.08	0.01	247869975	49.44	0.42	0.98
'CP003045.1'	0.57	0.10	53.63	2.67	0.08	0.03	2715000	0.02	2.57	0.97
'CM001483.1'	0.00	0.16	38.91	3.18	0.33	0.16	3740238	0.08	6.41	0.97
'JH650723.1'	0.62	0.30	8.97	0.98	0.27	0.18	496755	0.02	11.07	0.98
'CM000166.1'	0.05	0.07	53.23	1.78	0.35	0.09	28442294	9.84	3.90	0.99
'CM000364.1'	0.06	0.07	52.82	1.69	0.34	0.08	27517382	5.64	4.09	0.99
'CP003426.1'	0.67	0.08	209.39	19.98	0.02	0.01	919477	0.00	0.74	0.86
'JH650827.1'	0.00	0.19	46.95	4.62	0.31	0.21	4599047	0.06	4.89	0.94
'JH650968.1'	0.00	-1.00	51.90	3.07	0.24	0.05	5895957	0.48	4.90	0.91
'JH651225.1'	0.53	0.06	354.94	22.18	0.03	0.01	3433454	3.78	0.49	0.90
'JH651385.1'	0.00	-1.00	117.96	13.32	0.06	0.01	5064356	0.04	4.81	0.76
'JH657918.1'	0.00	-1.00	51.08	3.49	0.25	0.05	3361134	0.25	4.89	0.89
'JH658272.1'	0.00	-1.00	38.61	1.36	0.48	0.06	4544391	0.09	4.96	0.97
'JH658799.1'	0.35	0.15	31.57	2.47	0.20	0.09	937530	0.58	4.90	0.97
'JH658362.1'	0.06	0.17	36.79	2.98	0.42	0.22	3043968	0.27	4.90	0.96
'JH651516.1'	0.02	0.04	290.41	9.05	0.25	0.05	41204401	14.48	1.02	0.97
'JH655878.1'	0.11	0.03	402.61	9.49	0.15	0.03	89887394	3.38	0.86	0.97
'JH659329.1'	0.00	0.27	92.45	15.86	0.08	0.09	6345831	0.09	4.88	0.80
'DG000040.1'	0.76	0.11	40.87	2.31	0.05	0.02	1471808	0.16	2.85	0.97
'JH659590.1'	0.44	0.06	372.04	24.41	0.04	0.01	3543150	2.36	0.54	0.90
'KL650049.1'	0.31	0.16	17.99	1.16	0.31	0.13	1690939	0.16	7.92	0.99
'CM001486.1'	0.70	0.08	83.31	3.93	0.04	0.01	2540159	0.08	1.55	0.96
'CM001489.1'	0.00	-1.00	7.96	0.25	0.83	0.15	7590853	0.16	15.63	0.99
'JH668279.1'	0.13	0.10	61.71	3.53	0.27	0.10	3253989	1.73	3.40	0.97
'JH660758.1'	0.02	0.21	21.03	1.90	0.57	0.33	1953434	0.43	7.75	0.97
'JH660948.1'	0.50	0.10	61.60	3.65	0.09	0.03	2169277	5.79	2.35	0.96
'CM001515.1'	0.35	0.22	8.51	0.57	0.48	0.24	4412883	1.32	12.49	0.99
'CM003536.1'	0.00	-1.00	112.62	1.97	0.15	0.01	58970307	1.06	4.29	0.97
'JH668223.1'	0.70	0.13	36.01	2.42	0.07	0.03	900750	0.01	3.33	0.96
'CM001513.1'	0.14	0.19	15.14	1.03	0.51	0.26	6179543	0.00	9.19	0.98
'CM001512.1'	0.00	-1.00	16.29	0.63	0.55	0.10	6602611	0.01	9.68	0.97
'CM001514.1'	0.26	0.17	14.47	0.82	0.40	0.18	6297495	0.00	9.11	0.99
'CM001490.1'	0.00	0.26	15.85	1.48	0.53	0.37	6980251	0.04	10.19	0.97
'CM001491.1'	0.17	0.03	262.13	4.30	0.14	0.02	220367699	1.59	1.07	0.99
'JH687379.1'	0.30	0.17	21.18	1.41	0.29	0.14	3827153	2.64	6.51	0.98
'JH687538.1'	0.00	0.28	23.63	2.72	0.38	0.31	4675683	1.52	8.07	0.95
'JH687733.1'	0.00	-1.00	21.66	1.30	0.33	0.08	2214130	4.68	9.50	0.94
'CP003563.1'	0.08	0.22	10.73	0.78	0.68	0.36	6476459	0.00	12.27	0.99
'JH689321.1'	0.31	0.11	76.43	6.89	0.14	0.06	887685	2.55	2.51	0.95
'CM001518.2'	0.00	-1.00	69.24	1.81	0.24	0.03	33007066	2.50	4.07	0.96
'AKBU0100000	0.00	-1.00	9.55	0.42	0.72	0.16	3188779	0.00	13.90	0.98
'AKBV0100000	0.00	-1.00	27.90	0.98	0.39	0.05	4638970	0.00	7.47	0.97
'CM001525.1'	0.76	0.11	40.71	2.28	0.05	0.02	1524084	4.49	2.72	0.97
'AP012200.1'	0.39	0.09	100.48	6.65	0.10	0.03	1891014	0.00	1.77	0.95
'FN668944.1'	0.40	0.06	342.00	21.25	0.05	0.01	4178227	0.12	0.64	0.92
'CM001538.1'	0.24	0.14	25.54	1.55	0.30	0.13	3426323	0.24	6.20	0.98
'JH711484.1'	0.00	-1.00	22.46	1.09	0.44	0.09	4834015	0.21	8.25	0.95
'JH711783.1'	0.00	0.40	32.89	5.97	0.11	0.15	5133840	3.32	8.93	0.86
'JH717967.1'	0.00	0.07	>1000	>1000	0.01	0.00	9598269	11.37	2.29	0.73
'JH711573.1'	0.00	-1.00	41.50	2.74	0.18	0.04	4542293	1.18	6.68	0.89
'JH711528.1'	0.00	-1.00	47.53	1.35	0.42	0.04	6012395	1.64	4.44	0.97
'JH717896.1'	0.00	-1.00	99.20	9.96	0.07	0.02	6198950	0.01	4.89	0.79
'JH717839.1'	0.00	-1.00	118.61	12.87	0.06	0.02	5466126	0.47	4.76	0.78
'CM001557.1'	0.31	0.11	56.77	3.60	0.17	0.06	1834009	1.68	3.00	0.97
'AKVW010000(	0.00	-1.00	9.71	0.45	0.68	0.16	3188521	0.00	13.90	0.98
'AKVX0100000	0.00	-1.00	27.90	0.98	0.39	0.05	4638970	0.00	7.47	0.97
'AKVY0100000	0.44	0.09	58.39	3.18	0.11	0.04	2163340	0.00	2.73	0.97
'AFRY0100000	0.61	0.09	51.36	2.66	0.07	0.02	1908100	0.00	2.62	0.97
'AKGH010000C	0.00	0.17	31.85	2.65	0.48	0.25	3041360	0.00	5.92	0.97
'CM001542.1'	0.77	0.10	40.38	2.06	0.05	0.02	1473530	0.68	2.83	0.97
'JH719397.1'	0.64	0.15	13.82	0.69	0.18	0.07	3410954	2.09	8.00	0.99
'JH721862.1'	0.14	0.03	352.31	8.42	0.15	0.03	63520090	3.33	0.84	0.98
'CP003394.1'	0.53	0.10	96.33	7.02	0.06	0.02	1109051	0.01	1.62	0.94
'JH722767.1'	0.45	0.11	>1000	>1000	0.04	0.01	133136	19.66	0.60	0.58
'JH723757.1'	0.33	0.09	403.21	82.53	0.06	0.02	621857	15.07	0.72	0.82
'CP002723.1'	0.87	0.15	37.16	3.31	0.04	0.02	240865	0.00	2.90	0.94
'JH739887.1'	0.58	0.04	358.00	9.09	0.02	0.00	30495534	0.71	0.46	0.96
'CP003530.1'	0.80	0.13	66.89	7.39	0.03	0.01	231554	0.00	1.85	0.90
'JH725150.1'	0.00	-1.00	30.97	1.26	0.43	0.06	2084429	0.13	6.82	0.97
'JH725437.1'	0.00	-1.00	430.33	5.74	0.22	0.01	77654946	13.48	0.84	0.97
'JH767132.1'	0.00	0.21	15.10	1.35	0.61	0.32	1813439	18.69	9.11	0.98
'JH767554.1'	0.00	-1.00	84.74	9.73	0.08	0.02	1078546	0.36	6.47	0.82
'AKHW030005;	0.39	0.03	277.54	5.47	0.06	0.01	83598005	2.14	0.75	0.98
'CM001558.1'	0.29	0.21	12.80	0.86	0.41	0.21	6368168	0.00	9.74	0.98
'CM001559.1'	0.00	-1.00	14.43	0.45	0.68	0.11	6666321	0.02	10.23	0.98
'CP007549.3'	0.09	0.11	60.41	3.80	0.32	0.12	3846504	0.14	3.36	0.97
'JH767723.1'	0.29	0.04	225.75	4.41	0.10	0.02	79733107	3.35	1.03	0.98
'HE576794.1'	0.00	-1.00	31.72	1.33	0.31	0.05	2474718	0.40	7.76	0.96
'CP003318.1'	0.35	0.11	66.88	5.03	0.13	0.05	1269774	0.00	2.66	0.95
'CP003319.1'	0.36	0.13	62.21	5.10	0.14	0.06	1263719	0.00	2.77	0.95
'HE616890.1'	0.08	0.22	10.55	0.81	0.67	0.35	4305723	0.00	12.54	0.99
'JH768609.1'	0.00	-1.00	139.65	12.24	0.27	0.03	383464	4.16	2.57	0.91

'CAGI0100015(	0.00	-1.00	329.77	44.71	0.11	0.01	542606	0.09	2.87	0.84
'JH790073.1'	0.37	0.13	>1000	>1000	0.05	0.02	130934	24.23	0.63	0.63
'JH792473.1'	0.00	-1.00	30.71	1.50	0.44	0.07	1065947	1.90	6.62	0.96
'JH793700.1'	0.45	0.15	24.71	1.85	0.18	0.07	708350	0.57	5.89	0.98
'JH795166.1'	0.42	0.15	24.95	1.94	0.19	0.08	459401	0.54	5.96	0.98
'JH795855.1'	0.00	0.25	147.58	37.46	0.04	0.04	2114445	5.85	5.59	0.76
'JH795954.1'	0.00	-1.00	60.89	2.26	0.36	0.04	3215631	0.33	3.79	0.96
'CM001575.1'	0.75	0.11	39.68	2.32	0.06	0.02	1246548	1.38	2.95	0.97
'CM001561.1'	0.00	-1.00	14.72	0.45	0.63	0.10	6256692	0.56	10.27	0.98
'CM001809.1'	0.77	0.10	40.51	2.08	0.05	0.02	1532046	2.64	2.77	0.97
'JH802061.1'	0.42	0.03	223.80	4.17	0.06	0.01	83735184	11.86	0.79	0.98
'JH977583.1'	0.36	0.16	15.06	0.89	0.30	0.12	3220982	0.75	9.10	0.99
'JH814812.1'	0.80	0.16	20.27	1.39	0.09	0.04	949399	0.12	5.22	0.97
'JH823257.1'	0.32	0.04	246.72	5.54	0.08	0.02	63721086	16.40	0.75	0.98
'JH835289.1'	0.05	0.05	272.37	10.89	0.21	0.05	17429815	10.12	1.15	0.97
'JH817099.1'	0.00	0.09	179.43	16.78	0.28	0.10	1964558	7.01	1.78	0.94
'JH863719.1'	0.46	0.04	306.79	7.96	0.04	0.01	40202977	16.39	0.53	0.97
'JH859027.1'	0.00	-1.00	74.53	1.95	0.35	0.03	7262926	7.52	3.29	0.97
'JH881270.1'	0.35	0.05	315.18	13.80	0.07	0.02	8785736	3.98	0.68	0.95
'CM001580.1'	0.17	0.16	39.68	3.16	0.30	0.15	2266799	0.11	4.34	0.96
'CM001581.1'	0.30	0.13	36.40	2.37	0.22	0.09	2307912	0.17	4.32	0.97
'CM001582.2'	0.17	0.03	309.90	4.83	0.14	0.02	275406953	0.90	0.90	0.99
'JH878907.1'	0.75	0.09	28.66	1.06	0.07	0.02	3795215	1.99	3.93	0.99
'JH921428.1'	0.00	0.18	37.70	3.31	0.40	0.24	5185404	0.41	5.50	0.96
'JH947130.1'	0.39	0.04	251.22	5.47	0.06	0.01	49259779	9.00	0.74	0.98
'KQ039398.1'	0.53	0.11	46.59	2.87	0.10	0.04	1738350	1.47	3.09	0.97
'JH925096.1'	0.32	0.15	15.47	0.86	0.32	0.12	4348789	1.13	9.05	0.99
'JH971385.1'	0.67	0.15	21.88	1.25	0.12	0.05	2973556	1.24	5.09	0.98
'CP015457.1'	0.00	0.25	42.24	5.43	0.32	0.27	3550205	0.01	5.10	0.91
'JH930468.1'	0.00	-1.00	56.83	4.77	0.08	0.02	5865343	9.57	6.53	0.84
'KE388890.1'	0.00	0.12	36.21	2.02	0.44	0.16	5975090	3.45	5.72	0.98
'JH932235.1'	0.15	0.21	25.05	2.83	0.38	0.22	530628	0.01	6.50	0.96
'JH932306.1'	0.00	-1.00	65.61	5.43	0.29	0.04	285510	0.75	4.61	0.92
'CM003198.1'	0.00	-1.00	74.28	6.45	0.10	0.03	11688822	0.10	4.93	0.81
'CP003064.1'	0.82	0.16	13.61	0.77	0.12	0.05	1140038	0.03	7.51	0.99
'HE650821.1'	0.72	0.10	50.62	2.73	0.05	0.02	1757399	0.00	2.40	0.97
'HE608151.1'	0.42	0.21	8.24	0.51	0.44	0.20	4418088	3.33	12.28	0.99
'CM001609.2'	0.17	0.03	281.52	4.71	0.14	0.02	250522664	9.30	0.92	0.98
'CP003875.1'	0.00	0.18	44.22	4.37	0.32	0.19	2484940	0.01	5.25	0.95
'CM001632.1'	0.36	0.11	36.06	1.77	0.19	0.07	6089210	2.54	4.15	0.98
'JH977611.1'	0.16	0.03	349.03	8.17	0.13	0.02	65909399	5.83	0.82	0.98
'CM001772.1'	0.26	0.12	49.78	3.08	0.21	0.09	3358062	0.02	3.43	0.97
'CM001633.1'	0.31	0.12	32.97	1.68	0.22	0.09	5530491	0.04	4.67	0.98
'CM001642.1'	0.00	-1.00	148.44	2.52	0.25	0.01	37155481	3.91	2.26	0.97
'FN649727.1'	0.00	-1.00	25.53	0.77	0.51	0.07	9289656	1.96	7.00	0.98
'CP003920.1'	0.00	-1.00	35.78	1.49	0.41	0.06	2358861	0.00	5.79	0.96
'KB019377.1'	0.11	0.05	320.45	12.73	0.18	0.04	15735958	0.53	0.90	0.96
'HE965032.1'	0.35	0.22	26.82	3.56	0.22	0.13	244112	0.50	5.55	0.95
'HF534880.1'	0.04	0.06	155.08	7.23	0.27	0.07	9149724	14.98	1.65	0.97
'KB007974.1'	0.00	-1.00	96.56	12.36	0.04	0.01	2036344	0.71	8.66	0.78
'HE797160.1'	0.67	0.22	17.81	1.77	0.14	0.08	346962	0.42	6.30	0.96
'CAFZ0100000	0.86	0.18	15.41	1.21	0.10	0.04	254007	0.01	6.52	0.98
'DG000001.5'	0.20	0.05	128.59	3.70	0.16	0.04	37400331	23.44	1.50	0.98
'CACQ020013	0.10	0.27	30.64	7.59	0.35	0.22	49362	0.00	6.82	0.93
'HE671591.1'	0.07	0.10	91.59	6.93	0.27	0.09	1451426	0.49	2.89	0.96
'HE813982.1'	0.00	0.06	202.96	7.69	0.16	0.04	35439739	14.12	2.16	0.97
'JH980293.1'	0.24	0.04	198.79	3.76	0.13	0.02	111700974	8.86	1.08	0.99
'CM001774.1'	0.04	0.12	51.67	3.42	0.38	0.16	4343079	2.24	3.86	0.97
'CM001773.1'	0.03	0.12	64.11	4.66	0.37	0.16	3873423	8.83	3.07	0.96
'KB205985.1'	0.46	0.12	28.51	1.55	0.16	0.06	2267305	0.00	4.94	0.98
'JH994112.1'	0.18	0.09	94.83	5.48	0.20	0.07	4167794	0.72	2.30	0.96
'JH992965.1'	0.01	0.14	43.42	3.73	0.42	0.19	1423878	3.50	4.86	0.97
'JH993675.1'	0.41	0.10	29.06	1.15	0.18	0.06	5708466	2.43	4.96	0.99
'JH993736.1'	0.19	0.12	33.76	1.80	0.30	0.11	4553353	0.70	5.03	0.98
'HE681719.1'	0.65	0.09	54.54	2.41	0.06	0.02	2936865	1.27	2.32	0.97
'FO082046.1'	0.67	0.11	36.55	1.87	0.07	0.02	2122704	0.00	3.48	0.98
'HE806316.1'	0.51	0.08	92.56	4.61	0.07	0.02	2697332	0.02	1.71	0.96
'JH993832.1'	0.47	0.15	46.78	4.89	0.11	0.05	311951	6.66	3.09	0.94
'CP003600.1'	0.03	0.13	33.08	2.01	0.43	0.18	6284095	0.00	6.12	0.98
'CM001672.1'	0.08	0.03	292.30	6.01	0.21	0.04	126672627	6.98	0.91	0.98
'CM001705.1'	0.00	-1.00	186.23	3.45	0.28	0.02	36147563	6.00	1.56	0.97
'CP003642.1'	0.18	0.10	54.37	2.71	0.25	0.09	7003560	0.00	3.54	0.98
'KB209920.1'	0.00	0.11	37.65	1.99	0.44	0.16	6937932	0.30	5.79	0.98
'CM001710.2'	0.22	0.03	309.57	5.47	0.11	0.02	161917960	2.40	0.82	0.98
'HF545614.1'	0.79	0.12	37.03	2.15	0.05	0.02	1171011	0.22	3.02	0.97
'AMZP0200790	0.72	0.36	6.28	0.72	0.33	0.23	795109	0.00	13.94	0.98
'HE601810.1'	0.79	0.11	33.17	1.72	0.06	0.02	1044023	0.00	3.38	0.98
'HE601798.1'	0.78	0.10	33.62	1.74	0.06	0.02	1042585	0.02	3.38	0.98
'HE601799.1'	0.77	0.11	33.65	1.80	0.06	0.02	1042140	0.01	3.38	0.98
'HE601800.1'	0.78	0.11	33.43	1.80	0.06	0.02	1042560	0.01	3.38	0.98
'HE601802.1'	0.77	0.11	33.65	1.79	0.06	0.02	1042625	0.01	3.38	0.98
'HE601803.1'	0.77	0.11	33.64	1.89	0.06	0.02	1048812	0.04	3.38	0.97
'HE601806.1'	0.78	0.10	33.55	1.67	0.06	0.02	1042789	0.02	3.38	0.98
'HE601807.1'	0.79	0.11	33.29	1.78	0.06	0.02	1042171	0.01	3.38	0.98
'HE601794.1'	0.78	0.11	33.51	1.79	0.06	0.02	1042447	0.02	3.38	0.98
'HE601958.1'	0.78	0.13	32.98	2.04	0.06	0.02	1038876	0.02	3.39	0.97
'HE601960.1'	0.78	0.13	32.98	2.04	0.06	0.02	1038881	0.02	3.39	0.97
'HE601959.1'	0.79	0.12	32.85	1.99	0.06	0.02	1038879	0.01	3.39	0.97
'HE601961.1'	0.78	0.12	33.27	2.00	0.06	0.02	1038864	0.03	3.39	0.97
'HE601962.1'	0.78	0.12	33.03	1.97	0.06	0.02	1038718	0.04	3.39	0.97
'HE601964.1'	0.78	0.12	33.09	1.97	0.06	0.02	1038864	0.02	3.39	0.97
'HE601965.1'	0.78	0.12	33.25	2.06	0.06	0.02	1038848	0.04	3.39	0.97
'HE601963.1'	0.79	0.12	32.98	2.00	0.06	0.02	1038541	0.02	3.39	0.97
'HE601957.1'	0.78	0.12	33.04	1.98	0.06	0.02	1038746	0.14	3.38	0.97
'HE601951.1'	0.78	0.12	33.29	1.97	0.06	0.02	1039428	0.03	3.39	0.97
'HE793032.1'	0.48	0.07	210.62	14.12	0.05	0.01	2889522	1.03	0.86	0.91
'HE984298.2'	0.44	0.14	34.46	2.31	0.15	0.06	1804601	0.88	4.18	0.97
'KB020503.1'	0.50	0.17	19.69	1.65	0.19	0.08	493961	0.67	6.73	0.98
'KB456260.1'	0.00	-1.00	36.06	1.26	0.35	0.05	5112742	1.44	6.25	0.97
'KI925454.1'	0.00	0.28	184.75	53.72	0.02	0.02	3591957	0.00	6.98	0.71
'KB445791.1'	0.00	-1.00	27.85	1.57	0.35	0.08	3726281	2.10	7.19	0.93
'KQ234361.1'	0.04	0.13	52.40	4.17	0.36	0.15	1849524	1.49	4.23	0.97
'KQ234858.1'	0.00	0.12	52.20	3.77	0.39	0.15	2498669	1.08	4.57	0.97
'KQ235088.1'	0.00	-1.00	72.01	3.04	0.23	0.03	3189368	1.41	4.37	0.94
'KQ235548.1'	0.20	0.15	48.40	4.35	0.24	0.12	1257005	1.10	3.95	0.95
'KB722642.1'	0.00	0.30	12.38	1.38	0.74	0.54	1534859	0.18	11.05	0.97

'KB228879.1'	0.31	0.04	361.41	12.93	0.07	0.02	17817800	3.08	0.63	0.95
'DF157104.1'	0.24	0.11	47.34	2.69	0.22	0.08	3118530	3.87	3.84	0.98
'DG000053.1'	0.00	-1.00	113.03	1.69	0.18	0.01	45037867	5.78	3.84	0.98
'KN003119.1'	0.00	-1.00	30.48	1.09	0.38	0.05	4849263	0.91	6.95	0.97
'KB031068.1'	0.47	0.03	269.55	5.14	0.04	0.01	70361549	1.26	0.67	0.98
'KB445548.1'	0.00	-1.00	113.55	2.47	0.14	0.01	35245667	1.00	4.42	0.96
'CM002476.1'	0.00	-1.00	104.71	1.51	0.23	0.01	32922458	0.31	3.79	0.99
'AFYB0100000	0.40	0.24	55.50	18.62	0.12	0.08	34080	0.00	3.65	0.85
'CM001783.1'	0.00	-1.00	149.32	4.17	0.17	0.02	29380638	7.03	2.43	0.94
'KQ004500.1'	0.23	0.04	327.96	11.08	0.11	0.02	21408224	0.95	0.80	0.96
'ADVV0100008	0.81	0.11	39.42	2.30	0.05	0.02	817836	0.00	2.85	0.97
'DF159777.1'	0.18	0.07	149.59	9.77	0.16	0.05	2381190	3.21	1.67	0.95
'CAJW0115584	0.18	0.20	316.85	371.56	0.14	0.08	36084	0.06	2.33	0.76
'CAJX0100582	0.41	0.21	57.69	15.79	0.12	0.07	37442	0.00	3.56	0.88
'KB095811.1'	0.00	-1.00	125.06	3.06	0.29	0.02	13640604	2.48	2.26	0.96
'CAOK0100290	0.21	0.11	164.91	26.39	0.14	0.05	398841	3.19	1.67	0.90
'KB199562.1'	0.14	0.04	283.06	9.87	0.16	0.03	20151152	5.23	0.99	0.97
'CM001748.1'	0.09	0.05	155.88	3.86	0.24	0.05	70713020	1.63	1.72	0.98
'KB199650.1'	0.00	-1.00	255.07	7.84	0.18	0.01	9386848	10.71	1.56	0.94
'KB205139.1'	0.00	0.19	42.38	5.75	0.42	0.24	483664	3.38	5.02	0.94
'KB291798.1'	0.03	0.11	71.10	5.60	0.33	0.13	1620044	4.91	3.35	0.96
'DG000065.1'	0.58	0.12	39.06	2.23	0.09	0.03	2424339	1.46	3.41	0.97
'CM003140.1'	0.59	0.17	14.95	0.92	0.19	0.09	2476501	0.10	7.58	0.98
'FO203509.1'	0.33	0.24	8.58	0.62	0.50	0.27	4525948	0.07	12.70	0.99
'FO203507.1'	0.19	0.23	9.19	0.64	0.62	0.32	4432426	0.03	12.71	0.99
'FO203510.1'	0.00	-1.00	13.08	0.90	0.36	0.12	4524466	0.02	12.70	0.94
'FO203508.1'	0.24	0.23	8.95	0.62	0.57	0.29	4420197	0.02	12.71	0.99
'KB205939.1'	0.00	0.18	58.50	5.97	0.26	0.17	3865581	0.09	4.47	0.93
'KB206292.1'	0.00	-1.00	115.51	5.35	0.27	0.02	1346760	0.47	2.85	0.94
'KB207269.1'	0.00	-1.00	80.67	2.58	0.32	0.03	3493687	9.72	3.22	0.97
'FO203522.1'	0.43	0.12	30.84	1.57	0.17	0.06	3703034	0.00	4.55	0.98
'KB208804.1'	0.14	0.06	146.91	5.62	0.20	0.05	12776828	4.16	1.69	0.97
'HE796683.1'	0.00	-1.00	25.35	1.21	0.24	0.05	6775444	0.12	9.16	0.95
'CM001769.1'	0.07	0.05	198.06	5.73	0.24	0.06	59463898	8.52	1.25	0.97
'CM001762.1'	0.29	0.19	7.73	0.43	0.57	0.24	7092137	0.05	14.15	0.99
'CM003407.1'	0.00	-1.00	25.12	0.72	0.46	0.05	2960390	0.00	8.70	0.98
'KB217888.1'	0.35	0.13	16.22	0.85	0.30	0.10	2225806	1.09	8.53	0.99
'KQ490441.1'	0.83	0.16	38.74	4.50	0.05	0.02	133442	0.07	2.88	0.92
'CM001763.1'	0.00	-1.00	16.35	0.50	0.61	0.09	5930035	0.96	9.33	0.98
'KB222877.1'	0.36	0.15	30.15	2.40	0.20	0.08	828981	2.31	5.09	0.97
'KB221188.1'	0.50	0.03	352.18	6.51	0.03	0.00	77483305	0.30	0.55	0.98
'KQ479111.1'	0.43	0.17	27.93	2.51	0.17	0.08	677867	0.00	5.31	0.97
'KB233244.1'	0.78	0.15	26.91	1.91	0.07	0.03	600478	0.99	4.06	0.97
'KB316842.1'	0.00	-1.00	304.19	4.77	0.21	0.01	41399143	4.39	1.39	0.97
'CM001775.1'	0.27	0.08	75.78	3.74	0.16	0.05	4263418	0.02	2.67	0.97
'CM001776.1'	0.00	-1.00	30.61	1.10	0.36	0.05	5066637	0.01	7.16	0.97
'KB285691.1'	0.28	0.11	327.82	107.31	0.09	0.04	206462	5.15	1.18	0.82
'KB290581.1'	0.27	0.13	24.08	1.35	0.28	0.10	2157225	0.01	6.64	0.99
'AOFH0200000	0.01	0.25	29.67	5.72	0.45	0.28	129477	0.00	6.91	0.94
'AOFI02000001	0.61	0.14	25.41	1.58	0.12	0.05	1399912	0.00	4.80	0.98
'AOFJ0200000	0.73	0.14	23.35	1.49	0.09	0.04	850043	0.00	4.85	0.98
'AOFK0200000	0.73	0.14	23.36	1.49	0.09	0.04	850040	0.00	4.85	0.98
'CM007320.1'	0.38	0.11	24.11	1.05	0.21	0.07	3727717	1.28	6.19	0.99
'ANAF0200018	0.19	0.20	22.39	2.49	0.35	0.18	326849	0.22	7.45	0.97
'KB317696.1'	0.00	-1.00	51.74	3.66	0.18	0.04	4751343	4.35	5.23	0.88
'DF196775.1'	0.32	0.18	12.60	0.83	0.38	0.17	2384790	1.08	9.97	0.99
'KB320563.1'	0.00	0.06	193.32	7.55	0.27	0.07	19269909	5.66	1.70	0.97
'AOBL0200000	0.55	0.17	26.16	2.15	0.13	0.07	758954	0.00	4.80	0.96
'KB375528.1'	0.59	0.04	234.32	5.56	0.03	0.01	25666195	0.89	0.66	0.97
'KB390952.1'	0.55	0.04	241.52	6.86	0.03	0.01	18327016	0.56	0.67	0.96
'KB397780.1'	0.22	0.05	268.98	9.17	0.12	0.03	19410955	1.56	0.91	0.97
'CM001785.1'	0.00	-1.00	32.02	1.15	0.48	0.06	3141054	0.04	5.92	0.97
'KB423929.1'	0.25	0.11	566.56	311.72	0.09	0.04	151003	0.89	1.09	0.79
'CM001787.1'	0.45	0.09	46.82	2.06	0.12	0.04	5255772	1.40	3.19	0.98
'ANHN0200002	0.58	0.26	15.62	2.07	0.18	0.11	132756	0.00	7.59	0.96
'KQ479425.1'	0.51	0.23	16.66	1.98	0.20	0.11	145089	0.01	7.51	0.97
'KB444497.1'	0.91	0.11	990.89	778.83	0.01	0.00	90461	0.00	0.52	0.21
'CM002524.1'	0.00	-1.00	109.93	1.70	0.20	0.01	41795227	20.12	3.16	0.98
'KB445550.1'	0.60	0.18	14.60	0.92	0.19	0.09	2027063	0.03	7.52	0.98
'KB445569.1'	0.00	-1.00	49.36	2.36	0.24	0.04	4213224	0.71	5.49	0.94
'KB445637.1'	0.00	-1.00	38.37	1.27	0.42	0.05	3642493	3.52	5.35	0.97
'KB446535.1'	0.00	-1.00	25.29	1.08	0.46	0.09	5111597	0.51	6.98	0.96
'KB446555.1'	0.00	0.30	57.57	8.69	0.10	0.11	11880248	0.70	6.08	0.85
'CM007313.1'	0.31	0.14	19.56	1.14	0.29	0.11	2890053	6.55	6.96	0.99
'KV887145.1'	0.50	0.25	15.82	1.95	0.22	0.13	218908	1.09	7.76	0.96
'CM001792.1'	0.63	0.09	49.30	2.08	0.07	0.02	3830781	0.22	2.61	0.98
'KB453278.1'	0.40	0.18	17.48	1.43	0.26	0.12	621180	0.66	7.80	0.98
'KB616792.1'	0.10	0.11	273.46	59.74	0.18	0.07	531465	20.93	1.14	0.88
'CP007203.2'	0.28	0.10	53.59	2.73	0.19	0.06	5462271	0.08	3.34	0.98
'CM001794.1'	0.22	0.12	46.82	2.98	0.23	0.09	2929700	0.31	3.87	0.97
'CM001795.1'	0.17	0.14	48.68	3.78	0.26	0.12	2757856	0.37	3.87	0.96
'CM003613.1'	0.03	0.04	222.84	6.45	0.25	0.05	48970052	5.15	1.45	0.98
'CM001796.1'	0.26	0.12	44.78	2.85	0.21	0.08	2771508	0.62	3.89	0.97
'CM001797.1'	0.19	0.13	48.67	3.32	0.24	0.10	2857526	0.25	3.82	0.97
'CM001798.1'	0.25	0.14	44.53	3.24	0.21	0.10	2843090	0.36	3.89	0.96
'KB454452.1'	0.53	0.10	44.40	2.15	0.10	0.03	2877306	4.41	3.13	0.98
'KB454484.1'	0.71	0.12	47.05	3.66	0.05	0.02	465833	0.01	2.63	0.95
'KB455460.1'	0.45	0.11	27.69	1.35	0.16	0.05	2280433	3.97	5.20	0.99
'FO203427.1'	0.34	0.15	25.26	1.61	0.24	0.11	3646098	0.05	5.59	0.98
'HF689366.1'	0.22	0.14	178.14	55.31	0.14	0.06	114435	13.75	1.70	0.86
'KB465683.1'	0.00	-1.00	50.05	1.41	0.36	0.04	4355184	0.78	4.88	0.97
'CM001804.1'	0.36	0.11	52.48	2.78	0.15	0.06	5628425	3.28	3.02	0.97
'CM001799.1'	0.33	0.16	24.91	1.64	0.25	0.12	3205805	0.11	5.72	0.98
'CP001669.1'	0.40	0.10	65.26	3.56	0.12	0.04	3677484	0.00	2.50	0.97
'KB467406.1'	0.00	-1.00	41.94	1.35	0.35	0.04	4278185	2.13	5.66	0.97
'CM001824.1'	0.31	0.21	9.53	0.60	0.49	0.24	4787769	0.04	11.84	0.99
'KB528689.1'	0.45	0.05	173.87	4.96	0.06	0.01	22916839	3.25	1.00	0.97
'KB467831.1'	0.00	-1.00	68.51	4.54	0.08	0.02	5105897	2.10	6.97	0.88
'KE504123.1'	0.00	0.39	64.43	18.44	0.08	0.10	1378269	4.04	7.72	0.77
'KB469296.1'	0.00	-1.00	35.50	2.01	0.25	0.05	4293775	5.69	6.57	0.93
'KB610562.1'	1.07	0.09	760.02	420.19	0.01	0.00	50057	0.00	0.49	0.12
'KB632399.1'	0.81	0.08	41.83	1.40	0.05	0.01	7212326	6.15	2.54	0.98
'APGM010000(	0.00	-1.00	19.20	0.65	0.47	0.07	5095761	0.00	9.61	0.98
'KB637218.1'	0.93	0.11	993.06	811.90	0.01	0.00	90753	0.66	0.51	0.17
'CM007651.1'	0.01	0.06	142.80	4.36	0.31	0.08	47851208	0.91	1.99	0.98
'KB640820.1'	0.00	-1.00	53.25	1.39	0.33	0.03	3533895	3.85	5.09	0.98

'CM001827.1'	0.10	0.05	137.51	3.93	0.23	0.06	42086771	6.15	1.74	0.98
'KB644415.1'	0.37	0.11	25.36	1.11	0.22	0.07	5959152	0.11	5.82	0.99
'CM003631.1'	0.14	0.05	193.81	5.34	0.19	0.04	54992815	8.65	1.17	0.98
'KD503936.1'	0.00	-1.00	97.82	6.18	0.25	0.03	606643	15.73	3.20	0.94
'CAGA0100000	0.21	0.17	25.52	2.26	0.32	0.15	977986	2.54	6.31	0.97
'KD050940.1'	0.00	-1.00	101.35	7.11	0.20	0.03	1066088	23.04	3.06	0.90
'KB644853.1'	0.24	0.16	36.02	3.38	0.24	0.12	732963	0.05	4.87	0.96
'KB660352.1'	0.00	-1.00	166.80	7.46	0.09	0.01	15787204	5.09	3.01	0.88
'CP004012.1'	0.00	-1.00	10.00	0.36	0.80	0.15	3715422	0.00	13.47	0.98
'CM001835.1'	0.40	0.10	61.14	3.47	0.12	0.04	2073383	0.00	2.70	0.97
'HF562300.1'	0.77	0.10	33.70	1.77	0.06	0.02	1043022	0.02	3.38	0.98
'HF562298.1'	0.77	0.10	33.70	1.77	0.06	0.02	1043022	0.02	3.38	0.98
'HE978315.1'	0.31	0.13	36.68	2.65	0.21	0.09	1410756	0.04	4.59	0.97
'KB663610.1'	0.00	-1.00	46.21	0.82	0.35	0.03	31626230	4.69	5.08	0.99
'KB664288.1'	0.00	-1.00	40.85	1.41	0.37	0.04	3396703	0.92	5.75	0.97
'KB666509.1'	0.00	-1.00	47.75	1.06	0.36	0.03	6438948	12.05	4.72	0.98
'KB668221.1'	0.00	0.11	43.57	2.72	0.42	0.15	3832769	6.65	4.89	0.98
'KB669613.1'	0.00	-1.00	49.03	1.67	0.32	0.03	2664051	3.80	5.34	0.97
'KB672286.1'	0.00	-1.00	33.61	0.76	0.36	0.04	30179321	2.02	6.70	0.98
'KB672490.1'	0.00	-1.00	46.47	1.15	0.23	0.02	22947322	4.27	6.41	0.98
'KB673756.1'	0.55	0.19	26.51	3.14	0.13	0.07	169926	0.00	5.09	0.95
'KB704125.1'	0.00	-1.00	45.17	0.87	0.34	0.03	16348944	2.75	5.46	0.98
'HE999557.1'	0.13	0.16	20.39	1.61	0.40	0.16	814907	0.24	8.96	0.98
'HE971709.1'	0.24	0.29	7.84	0.69	0.63	0.39	9466619	0.02	14.04	0.99
'KB707241.1'	0.15	0.19	37.68	5.25	0.29	0.15	264153	0.24	5.44	0.95
'KB707785.1'	0.58	0.14	34.39	2.47	0.10	0.04	705855	0.15	3.84	0.97
'KB708127.1'	0.19	0.04	290.78	6.63	0.13	0.02	79790405	14.95	0.78	0.98
'KB714707.1'	0.40	0.04	391.07	16.13	0.05	0.01	12736304	15.78	0.47	0.94
'KB722736.1'	0.00	-1.00	99.66	12.56	0.07	0.02	3951450	0.23	5.11	0.75
'KB725665.1'	0.00	-1.00	209.73	48.29	0.02	0.01	2150137	0.55	7.07	0.67
'HG001459.1'	0.33	0.14	21.83	1.41	0.25	0.10	1172314	0.83	7.19	0.98
'KB731261.1'	0.43	0.12	29.12	1.43	0.17	0.06	3690067	0.54	4.94	0.98
'KB726998.1'	0.07	0.15	36.67	2.45	0.41	0.20	6307302	8.61	4.51	0.97
'KB726999.1'	0.26	0.04	303.08	9.21	0.10	0.02	28640624	11.54	0.68	0.97
'KB733444.1'	0.36	0.11	26.20	1.28	0.22	0.07	2277156	1.17	5.69	0.99
'KB741277.1'	0.65	0.08	43.93	1.70	0.07	0.02	4163268	10.01	2.66	0.98
'CM001837.1'	0.69	0.10	46.10	2.18	0.06	0.02	3305193	0.04	2.67	0.97
'KB742833.1'	0.15	0.06	307.82	19.09	0.16	0.04	5998093	1.14	0.92	0.94
'AOUM010000	0.69	0.12	18.92	0.96	0.12	0.04	1369792	0.00	6.11	0.99
'KB908481.1'	0.00	0.18	45.25	4.30	0.25	0.15	4026890	8.98	5.71	0.95
'CM001919.1'	0.17	0.03	267.34	4.34	0.14	0.02	227556264	4.45	1.01	0.99
'KB822804.1'	0.76	0.10	40.86	2.09	0.05	0.02	1105016	0.47	2.83	0.97
'KB822858.1'	0.72	0.11	41.34	2.43	0.06	0.02	1028389	0.43	2.91	0.97
'KB822910.1'	0.76	0.10	40.84	2.10	0.05	0.02	1110206	0.38	2.84	0.97
'KB822770.1'	0.76	0.10	40.63	2.06	0.05	0.02	1471086	0.61	2.84	0.97
'KB822726.1'	0.77	0.10	40.48	2.09	0.05	0.02	1470978	0.83	2.83	0.97
'KB822711.1'	0.00	-1.00	44.74	3.38	0.16	0.04	3761227	0.02	6.58	0.87
'KB822697.1'	0.54	0.14	17.08	0.83	0.19	0.08	5726035	0.01	6.97	0.99
'KB846784.1'	0.00	-1.00	220.21	4.46	0.27	0.01	14325340	7.47	1.52	0.97
'KB851304.1'	0.00	-1.00	44.14	3.63	0.33	0.06	242476	7.20	5.61	0.93
'CM001839.1'	0.00	-1.00	25.93	1.20	0.26	0.05	4934019	0.08	9.28	0.95
'CM001841.1'	0.00	-1.00	28.21	1.33	0.23	0.04	4652956	0.09	9.25	0.95
'CM001842.1'	0.00	-1.00	25.95	1.21	0.26	0.05	4865372	0.04	9.28	0.95
'CM001838.1'	0.00	-1.00	22.63	0.84	0.36	0.06	4649420	0.48	9.29	0.97
'CM001840.1'	0.00	-1.00	28.06	1.25	0.25	0.04	4775960	0.08	9.14	0.96
'CH003497.1'	0.26	0.03	274.79	4.35	0.10	0.02	248653822	10.14	0.81	0.99
'KB838940.1'	0.27	0.08	114.38	5.74	0.14	0.04	4951160	7.40	1.62	0.96
'KB824662.1'	0.94	0.11	859.36	591.08	0.01	0.00	90019	0.00	0.52	0.20
'HF937103.1'	0.56	0.09	55.47	2.69	0.08	0.03	2864125	0.72	2.48	0.97
'KB854187.1'	0.28	0.10	68.74	4.07	0.17	0.06	2348962	2.28	2.64	0.97
'CP012035.1'	0.08	0.11	61.49	3.98	0.33	0.13	3937175	7.28	3.12	0.97
'KB871578.1'	0.11	0.06	207.12	9.58	0.19	0.05	9823298	13.79	1.28	0.97
'KB853656.1'	0.46	0.20	18.18	1.81	0.22	0.11	233904	1.51	7.23	0.97
'KB863010.1'	0.00	-1.00	26.02	1.50	0.18	0.04	3018814	1.94	11.49	0.94
'KB870805.1'	0.00	-1.00	126.33	2.54	0.31	0.02	19566579	0.79	2.27	0.97
'CP005385.1'	0.50	0.16	14.50	0.81	0.24	0.10	3098881	0.00	7.96	0.99
'KK214815.1'	0.18	0.12	29.05	1.58	0.32	0.12	4615960	5.70	5.77	0.98
'KB886750.1'	0.00	-1.00	165.43	9.20	0.12	0.01	4424121	9.43	2.78	0.88
'CM001843.1'	0.01	0.15	30.33	2.33	0.46	0.21	2426396	1.16	6.13	0.98
'ANIS0100178	0.15	0.13	>1000	>1000	0.13	0.05	100988	0.00	1.27	0.78
'KB908904.1'	0.39	0.11	25.12	1.05	0.21	0.07	5894933	0.12	5.83	0.99
'KB908914.1'	0.33	0.10	96.27	8.89	0.12	0.04	571059	11.37	1.86	0.94
'CM001844.1'	0.43	0.12	54.63	3.69	0.12	0.05	1624667	0.11	2.86	0.96
'KB913038.1'	0.46	0.03	407.49	9.37	0.04	0.01	45240865	0.87	0.49	0.97
'KB915786.1'	0.26	0.16	18.46	1.47	0.33	0.14	480852	0.00	8.52	0.98
'KB929668.1'	0.40	0.05	259.44	9.35	0.06	0.01	12308914	2.69	0.77	0.96
'KK358017.1'	0.85	0.16	>1000	>1000	0.02	0.01	51979	62.39	0.32	0.14
'KK365130.1'	0.25	0.09	551.76	139.47	0.09	0.03	746881	23.58	0.58	0.81
'CP011802.1'	0.00	-1.00	42.27	1.26	0.41	0.04	4064321	0.02	5.09	0.97
'KE476611.1'	0.00	-1.00	24.61	1.70	0.47	0.08	175201	0.39	8.46	0.95
'KE463865.1'	0.50	0.18	16.31	1.48	0.21	0.09	222494	2.56	7.57	0.98
'KE482498.1'	0.00	-1.00	21.13	1.64	0.49	0.09	96228	2.76	9.13	0.96
'CP003902.1'	0.80	0.15	13.73	0.73	0.13	0.05	1140481	0.02	7.51	0.99
'KE465639.1'	0.17	0.27	14.25	2.22	0.45	0.26	110388	3.16	10.53	0.97
'KE494039.1'	0.28	0.27	19.34	3.26	0.31	0.19	120802	4.81	7.68	0.95
'KE499521.1'	0.11	0.23	15.20	1.95	0.49	0.26	185872	0.73	10.48	0.98
'KB932201.1'	0.00	0.21	25.11	2.27	0.33	0.21	4772237	12.81	7.91	0.97
'CM001848.2'	0.50	0.14	22.77	1.30	0.17	0.07	3042753	0.04	5.63	0.98
'BAAG0100407	0.80	0.19	377.36	471.16	0.02	0.01	21998	0.00	1.29	0.30
'CM001852.1'	0.05	0.31	8.05	0.75	0.85	0.57	4219246	0.34	14.59	0.99
'KB933500.1'	0.33	0.08	60.84	2.61	0.15	0.04	5510682	10.22	2.70	0.98
'KB964490.1'	0.01	0.12	108.41	12.43	0.32	0.13	789565	1.73	2.60	0.94
'KB933264.1'	0.00	-1.00	37.41	1.59	0.44	0.06	1628638	0.03	5.45	0.96
'KD962201.1'	0.00	-1.00	124.46	7.25	0.30	0.03	698072	5.68	2.40	0.94
'ARYA0102489	0.27	0.15	319.14	216.87	0.10	0.05	59540	0.02	1.72	0.78
'KE007224.1'	0.57	0.14	29.61	2.07	0.12	0.05	793587	0.15	4.55	0.97
'CM001857.1'	0.00	-1.00	26.40	1.18	0.28	0.05	4631867	0.10	8.97	0.96
'CM001858.1'	0.00	0.23	26.51	2.68	0.35	0.23	4557062	0.04	7.93	0.96
'CM001860.1'	0.00	0.26	24.16	2.63	0.30	0.22	4860047	0.56	9.27	0.95
'CM001861.1'	0.48	0.14	23.17	1.35	0.18	0.08	3068655	0.06	5.62	0.98
'KE029017.1'	0.47	0.10	546.22	248.48	0.04	0.01	177843	7.70	0.77	0.71
'KE123234.1'	0.04	0.09	93.09	5.98	0.30	0.10	2804903	4.87	2.80	0.97
'CM001859.1'	0.00	-1.00	25.15	1.15	0.28	0.06	4867258	0.19	9.27	0.95
'CP009075.1'	0.00	-1.00	70.33	4.19	0.07	0.01	9275483	0.00	7.10	0.88
'KE113671.1'	0.03	0.05	367.19	16.78	0.22	0.05	13453139	7.08	0.93	0.96
'KE123896.1'	0.66	0.13	46.58	3.61	0.07	0.03	664271	7.04	2.58	0.95

'KE123882.1'	0.63	0.07	224.58	18.19	0.02	0.01	1763222	26.77	0.52	0.88
'KE124552.1'	0.57	0.09	426.73	106.99	0.02	0.01	410486	27.52	0.49	0.73
'CM001871.1'	0.00	-1.00	11.92	0.49	0.66	0.14	4908255	0.29	11.99	0.98
'KE124775.1'	0.51	0.09	39.23	1.82	0.11	0.03	2174208	5.99	3.54	0.98
'DF238808.1'	0.49	0.20	13.87	1.04	0.25	0.13	1398671	0.04	8.52	0.98
'CM001880.1'	0.04	0.05	207.76	6.29	0.26	0.06	42436413	2.78	1.39	0.98
'CM001897.1'	0.00	-1.00	12.13	0.50	0.63	0.13	4928563	0.12	12.00	0.98
'CM001890.1'	0.00	-1.00	12.19	0.50	0.62	0.13	4920230	0.11	12.01	0.98
'CM001889.1'	0.00	0.29	8.11	0.71	0.93	0.60	8496762	1.02	14.67	0.99
'APFE0311456	0.01	0.09	298.68	42.42	0.25	0.09	1058009	6.72	1.25	0.91
'CM001972.1'	0.00	-1.00	28.41	1.20	0.31	0.05	4693747	0.11	7.85	0.96
'CM001904.1'	0.00	-1.00	29.33	1.35	0.24	0.04	4549457	0.06	8.70	0.95
'CM001973.2'	0.00	-1.00	28.13	1.22	0.33	0.06	4264625	0.13	7.67	0.96
'CM001974.1'	0.00	-1.00	28.59	1.22	0.26	0.04	4669628	0.12	8.69	0.96
'CM001975.1'	0.00	-1.00	30.12	1.12	0.31	0.05	4214632	0.14	7.62	0.97
'CM001976.1'	0.00	-1.00	26.97	1.22	0.24	0.04	4829443	0.08	9.41	0.95
'CM001977.1'	0.00	-1.00	30.20	1.49	0.25	0.05	4552467	0.09	7.91	0.94
'CM001978.1'	0.00	-1.00	27.41	1.36	0.23	0.05	4802068	0.15	9.45	0.95
'CM001979.1'	0.00	-1.00	26.40	1.33	0.23	0.05	4993902	0.09	9.58	0.95
'CM001980.1'	0.00	-1.00	24.76	0.91	0.41	0.06	4622940	0.06	7.90	0.97
'CM001981.1'	0.00	-1.00	28.68	1.49	0.24	0.05	4681779	0.15	8.78	0.94
'CM001982.1'	0.00	-1.00	26.32	1.32	0.24	0.05	4781918	0.07	9.48	0.95
'CM001983.1'	0.00	-1.00	30.55	1.39	0.29	0.05	4306477	0.37	7.58	0.95
'CM001984.1'	0.00	-1.00	26.75	1.24	0.24	0.04	4905506	0.09	9.57	0.95
'CM001985.1'	0.00	0.22	26.53	2.53	0.35	0.23	4658967	0.07	7.90	0.96
'CM004347.1'	0.56	0.12	13.97	0.60	0.21	0.06	2618838	0.55	8.49	0.99
'KE145367.1'	0.30	0.12	32.93	1.78	0.23	0.09	3594336	0.46	4.72	0.98
'CM001948.2'	0.28	0.03	259.99	4.62	0.10	0.02	139301422	0.82	0.91	0.98
'CP004010.2'	0.82	0.16	13.61	0.77	0.12	0.05	1139633	0.00	7.51	0.99
'CP004011.1'	0.82	0.16	13.63	0.78	0.12	0.05	1139569	0.02	7.50	0.99
'CM004641.1'	0.33	0.14	16.48	0.88	0.31	0.11	2652413	1.11	8.44	0.99
'KE147309.1'	0.39	0.16	15.66	1.04	0.28	0.11	1354531	2.90	8.47	0.99
'KE148146.1'	0.00	0.13	31.21	1.98	0.46	0.19	4381405	0.34	6.58	0.98
'CM007249.1'	0.55	0.15	13.73	0.74	0.22	0.09	2596710	0.45	8.64	0.99
'AIJO01004611	0.24	0.29	29.02	6.80	0.26	0.19	71563	0.00	6.02	0.91
'KE158016.1'	0.12	0.11	149.64	21.06	0.20	0.08	514026	6.47	1.89	0.92
'KE156110.1'	0.20	0.10	78.05	5.46	0.20	0.07	1746141	3.61	2.61	0.96
'KE151199.1'	0.00	0.09	178.33	17.80	0.28	0.10	1494055	4.39	1.90	0.94
'KE159915.1'	0.66	0.13	22.80	1.34	0.11	0.04	832916	0.92	5.27	0.98
'ALWZ04S2422	0.09	0.08	243.49	24.94	0.20	0.07	1653216	12.37	1.16	0.92
'CM001986.1'	0.10	0.18	15.02	0.95	0.55	0.26	6077542	0.49	9.35	0.99
'CP006026.1'	0.18	0.14	28.60	1.94	0.31	0.12	1597628	0.00	6.47	0.98
'KE161240.1'	0.10	0.04	318.70	11.36	0.18	0.04	22122726	3.37	0.97	0.97
'CM002276.1'	0.24	0.13	38.13	2.40	0.25	0.10	2699012	7.04	4.07	0.97
'KE332545.1'	0.03	0.07	151.10	9.08	0.26	0.07	4533011	7.36	2.05	0.96
'KL558888.1'	0.00	-1.00	126.63	8.57	0.21	0.02	648085	5.71	3.20	0.92
'KE346217.1'	0.18	0.08	112.97	7.01	0.18	0.06	3477367	3.47	2.07	0.96
'ASJL01017490	0.38	0.23	99.30	57.26	0.10	0.06	23157	0.00	3.00	0.78
'KE362319.1'	0.40	0.25	37.24	10.16	0.16	0.09	29239	0.09	4.74	0.90
'KE361623.1'	0.00	-1.00	19.00	1.61	0.28	0.09	1975278	0.36	10.90	0.91
'CM002022.1'	0.33	0.22	8.55	0.58	0.50	0.24	4391869	0.54	12.66	0.99
'KE370772.1'	0.00	-1.00	106.40	4.02	0.24	0.02	3017511	25.79	2.52	0.95
'KE374994.1'	0.32	0.12	43.67	2.78	0.19	0.07	5203757	57.48	1.64	0.97
'CP006567.1'	0.00	-1.00	8.73	0.29	0.88	0.17	12700734	2.48	13.70	0.99
'KE382941.1'	0.33	0.05	467.27	23.43	0.06	0.01	8324132	2.14	0.49	0.93
'KE384729.1'	0.38	0.13	24.24	1.35	0.22	0.08	2704444	0.16	5.98	0.98
'KE385298.1'	0.35	0.18	24.99	2.41	0.22	0.11	468691	0.01	6.09	0.97
'HF951689.1'	0.59	0.15	15.27	0.78	0.19	0.07	3437861	0.31	7.43	0.99
'FP885895.1'	0.00	-1.00	10.67	0.38	0.68	0.12	3596030	0.06	13.44	0.98
'CM002023.1'	0.00	-1.00	28.35	1.30	0.23	0.04	4662278	0.11	9.20	0.95
'KE391414.1'	0.30	0.22	17.01	2.05	0.31	0.16	171320	0.05	8.62	0.97
'KE392324.1'	0.52	0.21	100.97	41.62	0.06	0.04	57883	26.53	1.64	0.75
'ATLZ01001265	0.06	0.29	46.13	18.17	0.32	0.23	30512	0.22	5.79	0.89
'CM002024.1'	0.05	0.26	12.73	1.19	0.66	0.41	2938080	0.05	10.64	0.98
'KE444511.1'	0.24	0.25	28.17	6.20	0.26	0.16	47541	1.95	6.41	0.94
'CP004849.1'	0.63	0.13	21.23	1.00	0.13	0.05	4321296	0.00	5.51	0.99
'CP006618.1'	0.79	0.12	34.84	1.97	0.06	0.02	1473302	0.01	3.19	0.97
'KE504634.1'	0.00	-1.00	159.48	4.93	0.24	0.02	4597586	17.51	2.01	0.96
'KE523052.1'	0.83	0.23	27.09	4.96	0.06	0.03	33513	0.04	4.05	0.90
'KE525347.1'	0.00	-1.00	41.29	1.06	0.38	0.04	5918260	0.59	5.67	0.98
'KE526711.1'	0.66	0.14	35.06	3.38	0.08	0.03	169254	0.00	3.66	0.96
'KE525743.1'	0.42	0.14	25.75	1.83	0.19	0.08	937461	0.00	5.62	0.98
'KE537387.1'	0.22	0.16	18.09	1.28	0.37	0.15	1294371	4.20	8.30	0.99
'KE561209.1'	0.39	0.11	75.15	6.35	0.11	0.04	719121	8.85	2.10	0.95
'KE546965.1'	0.00	-1.00	68.35	4.17	0.18	0.03	2816824	0.16	4.75	0.90
'KE549796.1'	0.57	0.17	36.80	3.79	0.10	0.05	345515	2.08	3.62	0.94
'KE558520.1'	0.00	-1.00	182.61	4.80	0.18	0.01	11539319	1.82	2.39	0.95
'KE557376.1'	0.18	0.07	312.18	30.94	0.13	0.04	1713168	0.43	0.98	0.92
'GK000053.2'	1.52	0.51	39.37	10.66	0.00	0.00	5400307	0.24	1.44	0.41
'ATMH0100992	0.00	-1.00	86.58	26.07	0.24	0.05	23937	0.03	6.09	0.85
'ATMG0101458	0.75	0.30	15.57	2.98	0.13	0.08	30297	0.24	6.94	0.93
'CM002081.1'	0.00	-1.00	175.41	2.29	0.24	0.01	68435666	21.46	1.69	0.98
'CAUG0100063	0.74	0.23	11.27	1.03	0.17	0.09	347077	0.03	9.00	0.98
'CM002048.1'	0.33	0.22	8.55	0.58	0.50	0.24	4398504	0.41	12.68	0.99
'HG316454.1'	0.80	0.10	31.43	1.46	0.06	0.02	1686157	0.14	3.57	0.98
'CM002049.1'	0.34	0.22	8.55	0.58	0.49	0.24	4402571	0.78	12.61	0.99
'CM002050.1'	0.34	0.22	8.54	0.57	0.49	0.24	4408178	0.72	12.63	0.99
'CM002258.1'	0.02	0.28	12.69	1.30	0.71	0.48	2856286	0.02	10.69	0.98
'CM002051.1'	0.40	0.22	8.31	0.55	0.45	0.22	4408147	1.28	12.56	0.99
'KE561408.1'	0.44	0.18	14.68	1.04	0.26	0.12	1220848	1.09	8.69	0.98
'KE581409.1'	0.44	0.23	20.95	3.21	0.20	0.11	76007	1.67	6.62	0.95
'CM002052.1'	0.33	0.22	8.56	0.58	0.49	0.24	4397671	0.90	12.60	0.99
'CM002053.1'	0.34	0.22	8.54	0.57	0.49	0.23	4408813	1.33	12.52	0.99
'CM002054.1'	0.34	0.22	8.50	0.57	0.49	0.24	4404319	0.54	12.67	0.99
'CM002055.1'	0.34	0.22	8.54	0.58	0.49	0.24	4393994	1.48	12.49	0.99
'CM002057.1'	0.34	0.22	8.55	0.57	0.49	0.24	4408133	0.39	12.68	0.99
'CM002056.1'	0.33	0.23	8.58	0.60	0.50	0.25	4398802	0.66	12.65	0.99
'CM002058.1'	0.34	0.22	8.55	0.57	0.49	0.24	4393218	0.57	12.64	0.99
'CM002059.1'	0.34	0.22	8.55	0.57	0.49	0.24	4404835	0.64	12.65	0.99
'CM002060.1'	0.34	0.22	8.55	0.57	0.49	0.24	4404586	0.68	12.64	0.99
'CM002061.1'	0.34	0.22	8.55	0.58	0.49	0.24	4402591	0.70	12.63	0.99
'CM002062.1'	0.35	0.22	8.53	0.58	0.48	0.23	4405148	2.88	12.25	0.99
'CM002063.1'	0.34	0.22	8.54	0.58	0.49	0.24	4403568	0.09	12.72	0.99
'CM002064.1'	0.34	0.22	8.54	0.58	0.49	0.24	4404247	1.50	12.48	0.99
'CM002065.1'	0.41	0.21	8.26	0.52	0.44	0.20	4400856	1.31	12.56	0.99
'CM002066.1'	0.34	0.22	8.55	0.58	0.49	0.24	4412421	0.84	12.60	0.99

'CM002067.1'	0.34	0.22	8.54	0.58	0.49	0.24	4407862	1.81	12.43	0.99
'CM002068.1'	0.34	0.21	8.59	0.56	0.49	0.23	4392448	2.34	12.34	0.99
'CM002069.1'	0.34	0.22	8.54	0.58	0.49	0.24	4394000	0.65	12.64	0.99
'CM002070.1'	0.36	0.22	8.49	0.57	0.48	0.24	4403042	1.97	12.41	0.99
'CM002073.1'	0.34	0.22	8.54	0.57	0.49	0.24	4392414	0.41	12.67	0.99
'CM002071.1'	0.33	0.22	8.56	0.58	0.50	0.24	4390137	0.59	12.66	0.99
'CM002072.1'	0.33	0.22	8.55	0.58	0.49	0.24	4401613	0.45	12.68	0.99
'CM002076.1'	0.33	0.22	8.55	0.58	0.49	0.24	4391527	0.34	12.68	0.99
'CM002077.1'	0.34	0.22	8.54	0.58	0.49	0.24	4403994	0.61	12.63	0.99
'CM002079.1'	0.35	0.22	8.53	0.57	0.49	0.23	4402981	1.67	12.46	0.99
'CM002074.1'	0.34	0.22	8.54	0.58	0.49	0.24	4397923	1.86	12.42	0.99
'CM002075.1'	0.34	0.22	8.54	0.58	0.49	0.24	4395537	1.25	12.53	0.99
'CM002078.1'	0.34	0.22	8.51	0.57	0.49	0.24	4386956	0.40	12.68	0.99
'CM002080.1'	0.35	0.22	8.53	0.57	0.48	0.23	4404380	2.09	12.38	0.99
'KE598558.1'	0.00	-1.00	362.63	69.45	0.03	0.01	2265542	1.84	4.38	0.70
'ATSW0101163	0.09	0.21	83.20	29.91	0.24	0.14	48692	0.09	3.93	0.88
'CM007213.1'	0.26	0.15	16.58	1.01	0.36	0.14	2678989	1.57	8.65	0.99
'CM002127.1'	0.34	0.22	8.54	0.57	0.49	0.24	4407393	1.17	12.55	0.99
'CM002126.1'	0.34	0.22	8.56	0.58	0.49	0.24	4386949	1.41	12.49	0.99
'CM002125.1'	0.35	0.22	8.53	0.57	0.49	0.23	4407883	1.58	12.47	0.99
'CM002124.1'	0.36	0.22	8.52	0.56	0.48	0.23	4388875	2.81	12.24	0.99
'CM002122.1'	0.34	0.22	8.55	0.58	0.49	0.24	4406449	0.74	12.62	0.99
'CM002121.1'	0.35	0.22	8.53	0.57	0.49	0.23	4408055	1.85	12.43	0.99
'CM002120.1'	0.32	0.22	8.62	0.58	0.51	0.24	4388029	1.08	12.55	0.99
'CM002123.1'	0.33	0.22	8.57	0.59	0.50	0.25	4405197	0.89	12.62	0.99
'CM002119.1'	0.33	0.22	8.56	0.59	0.50	0.25	4408738	0.74	12.64	0.99
'CM002114.1'	0.34	0.22	8.54	0.57	0.49	0.24	4405199	0.70	12.64	0.99
'CM002116.1'	0.34	0.22	8.54	0.57	0.49	0.24	4405944	0.75	12.63	0.99
'CM002118.1'	0.34	0.22	8.55	0.58	0.49	0.24	4402491	0.71	12.62	0.99
'CM002115.1'	0.34	0.22	8.54	0.58	0.49	0.24	4398105	0.97	12.57	0.99
'CM002117.1'	0.33	0.21	8.59	0.57	0.50	0.23	4406983	1.43	12.50	0.99
'CM002113.1'	0.34	0.22	8.54	0.58	0.49	0.24	4398277	0.86	12.60	0.99
'CM002111.1'	0.34	0.22	8.55	0.58	0.49	0.24	4405806	1.00	12.58	0.99
'CM002109.1'	0.33	0.22	8.55	0.58	0.49	0.24	4399724	0.40	12.68	0.99
'CM002112.1'	0.33	0.22	8.57	0.59	0.50	0.24	4400549	1.09	12.58	0.99
'CM002108.1'	0.34	0.22	8.54	0.57	0.49	0.24	4406371	0.63	12.65	0.99
'CM002110.1'	0.34	0.22	8.55	0.58	0.49	0.24	4402266	1.07	12.57	0.99
'CM002107.1'	0.35	0.22	8.53	0.58	0.49	0.24	4394016	2.04	12.38	0.99
'CM002106.1'	0.34	0.22	8.55	0.58	0.49	0.24	4392209	2.07	12.40	0.99
'CM002105.1'	0.34	0.22	8.55	0.58	0.49	0.24	4402661	0.54	12.66	0.99
'CM002104.1'	0.34	0.22	8.54	0.57	0.49	0.24	4407151	0.78	12.62	0.99
'CM002102.1'	0.33	0.22	8.54	0.58	0.49	0.24	4399991	0.23	12.71	0.99
'CM002103.1'	0.34	0.22	8.51	0.57	0.49	0.24	4400321	0.58	12.66	0.99
'CM002101.1'	0.34	0.22	8.53	0.58	0.49	0.24	4400052	0.72	12.62	0.99
'CM002100.1'	0.34	0.22	8.55	0.58	0.49	0.24	4408002	1.06	12.57	0.99
'CM002098.1'	0.33	0.22	8.57	0.59	0.50	0.25	4398360	1.08	12.60	0.99
'CM002099.1'	0.42	0.21	8.23	0.51	0.44	0.20	4369690	0.71	12.62	0.99
'CM002097.1'	0.34	0.22	8.54	0.57	0.49	0.24	4409116	1.00	12.58	0.99
'KE647164.1'	0.52	0.09	>1000	>1000	0.02	0.01	154531	0.20	0.56	0.56
'KE650805.1'	0.53	0.10	92.98	6.62	0.06	0.02	1354122	0.66	1.65	0.94
'KE650838.1'	0.49	0.10	96.33	7.13	0.07	0.03	1369109	1.21	1.65	0.94
'KE650869.1'	0.52	0.09	94.32	6.13	0.06	0.02	1391605	3.80	1.59	0.95
'KE650910.1'	0.45	0.10	100.38	7.31	0.08	0.03	1230377	1.70	1.64	0.94
'KE650951.1'	0.51	0.10	95.00	7.16	0.06	0.02	1076003	2.46	1.63	0.93
'KE651002.1'	0.52	0.09	95.05	5.98	0.06	0.02	1496414	1.43	1.62	0.95
'KE651030.1'	0.45	0.09	99.42	6.09	0.08	0.03	2151241	0.32	1.66	0.95
'KE651071.1'	0.55	0.10	92.03	6.51	0.06	0.02	1054346	0.03	1.67	0.94
'KE651119.1'	0.51	0.09	96.76	6.52	0.07	0.02	1331235	0.80	1.65	0.94
'KE651128.1'	0.53	0.09	93.14	6.04	0.06	0.02	1332679	0.68	1.65	0.95
'KV447881.1'	0.57	0.16	20.56	1.64	0.15	0.06	435012	0.50	6.08	0.98
'KE680611.1'	0.40	0.04	384.15	14.10	0.05	0.01	14658418	3.20	0.51	0.95
'KE652172.1'	0.00	0.28	24.69	4.96	0.49	0.34	130882	1.96	7.88	0.94
'CM002128.1'	0.36	0.10	50.86	2.76	0.15	0.05	2334592	4.94	3.12	0.97
'CM002129.1'	0.33	0.13	39.27	2.30	0.19	0.08	3447851	5.30	3.74	0.97
'CM002130.1'	0.52	0.11	53.91	3.06	0.09	0.03	2399857	3.35	2.61	0.97
'CM002131.1'	0.62	0.10	46.07	2.42	0.07	0.03	1906877	2.20	2.84	0.97
'CM002132.1'	0.52	0.09	51.66	2.60	0.09	0.03	2128142	2.50	2.75	0.97
'CM002133.1'	0.59	0.10	49.53	2.58	0.08	0.03	2360910	10.92	2.49	0.97
'CM002135.1'	0.17	0.12	59.20	4.26	0.24	0.10	2177985	1.52	3.35	0.96
'CM002136.1'	0.00	0.32	11.86	1.26	0.60	0.47	4926805	0.03	12.22	0.97
'CM002029.1'	0.00	-1.00	12.53	0.56	0.55	0.13	5164757	0.03	12.11	0.97
'CM002139.1'	0.00	-1.00	11.72	0.46	0.70	0.13	5197712	0.14	12.05	0.98
'KE695867.1'	0.28	0.05	304.72	13.25	0.09	0.02	8641424	2.35	0.81	0.96
'KE706420.1'	0.00	0.10	188.46	17.82	0.24	0.10	2183814	1.37	1.96	0.91
'KE705278.1'	0.00	-1.00	90.74	2.07	0.30	0.02	11827220	3.24	3.14	0.97
'KE720815.1'	0.00	-1.00	44.98	2.34	0.41	0.06	803103	0.49	4.79	0.95
'KE722155.1'	0.00	0.05	260.43	7.99	0.23	0.05	41607841	2.41	1.55	0.97
'KE728806.1'	0.93	0.33	7.01	0.93	0.20	0.11	60334	0.00	11.89	0.98
'KE744832.1'	0.46	0.16	>1000	>1000	0.06	0.03	26402	3.46	0.99	0.64
'KE747824.1'	0.00	-1.00	35.10	1.27	0.43	0.06	2679305	0.92	5.85	0.97
'KE749393.1'	0.49	0.10	348.36	77.34	0.04	0.01	347696	0.15	0.79	0.78
'KI026023.1'	0.41	0.09	>1000	>1000	0.04	0.01	179377	0.38	0.60	0.65
'KI197197.1'	0.15	0.12	367.24	174.10	0.15	0.06	119694	0.13	1.60	0.84
'KE856091.1'	0.16	0.09	>1000	>1000	0.11	0.04	252993	0.25	0.89	0.79
'KI127945.1'	0.38	0.12	156.63	29.52	0.08	0.04	231900	0.16	1.49	0.86
'KE993672.1'	0.00	-1.00	67.10	1.46	0.31	0.02	12844513	9.38	3.88	0.98
'KI242139.1'	1.00	0.19	370.03	521.73	0.02	0.01	8164	3.60	1.35	0.12
'KV744154.1'	0.57	0.20	16.11	1.53	0.18	0.09	234588	0.25	7.53	0.97
'CP006585.1'	0.00	-1.00	17.97	0.77	0.53	0.10	3693999	0.64	9.10	0.97
'CP006705.1'	0.00	-1.00	13.48	0.63	0.47	0.11	6043974	0.24	12.14	0.97
'CP006728.1'	0.00	-1.00	13.59	0.62	0.46	0.11	6262305	0.14	12.16	0.97
'KI273213.1'	0.00	-1.00	191.23	9.44	0.09	0.01	3598413	3.53	3.68	0.91
'HF955515.1'	0.52	0.12	36.52	2.37	0.12	0.04	947606	12.59	3.44	0.97
'LN902841.1'	0.65	0.07	37.72	1.05	0.08	0.02	20116479	0.01	3.40	0.99
'LN906329.1'	0.82	0.07	44.39	1.12	0.04	0.01	22338478	11.85	2.25	0.99
'HG326877.1'	0.55	0.09	85.40	5.10	0.06	0.02	1584486	0.38	1.76	0.95
'KI304388.1'	0.00	0.09	219.97	26.74	0.27	0.09	1141856	3.18	1.67	0.93
'CM002176.1'	0.34	0.16	16.15	1.05	0.31	0.13	2713245	1.92	8.46	0.99
'CM002177.1'	0.00	-1.00	7.66	0.29	0.90	0.18	6008321	0.30	15.53	0.99
'KI419148.1'	0.14	0.04	371.10	14.09	0.15	0.03	17726253	6.18	0.79	0.96
'KI397142.1'	0.21	0.06	129.44	4.58	0.17	0.04	15980527	5.31	1.65	0.98
'KI402176.1'	0.00	-1.00	147.38	5.53	0.21	0.02	3076144	2.09	2.69	0.95
'KI916183.1'	0.00	0.12	37.99	2.33	0.43	0.16	4945056	10.13	5.28	0.98
'AORO020049(	0.22	0.05	390.23	23.82	0.10	0.03	5457815	0.91	0.72	0.93
'KI419149.1'	0.00	-1.00	81.86	1.42	0.32	0.02	22559587	1.23	3.46	0.98
'KI433418.1'	0.35	0.25	46.27	14.67	0.16	0.10	33168	16.49	3.85	0.88

'KI422464.1'	0.16	0.17	46.36	6.05	0.26	0.13	390863	2.29	4.36	0.94
'KI915040.1'	0.00	-1.00	49.09	1.00	0.24	0.02	30017264	2.75	6.06	0.98
'KI421882.1'	0.00	-1.00	34.27	0.62	0.45	0.04	20238125	9.46	5.68	0.99
'KI919284.1'	0.06	0.18	44.36	6.87	0.34	0.17	154078	4.52	5.02	0.95
'KI915156.1'	0.00	-1.00	45.00	1.21	0.33	0.03	8183372	7.66	5.26	0.98
'CM002796.1'	0.06	0.24	11.03	0.86	0.70	0.41	7041479	8.78	10.96	0.99
'KI440842.1'	0.09	0.12	23.47	1.20	0.42	0.15	6764743	0.06	7.75	0.99
'KI516373.1'	0.00	-1.00	124.14	12.69	0.17	0.03	708014	17.36	3.12	0.86
'ATNT0100058	0.26	0.15	27.23	2.27	0.26	0.11	698268	0.00	6.16	0.97
'KI472552.1'	0.26	0.11	85.34	7.14	0.16	0.06	1098238	0.68	2.39	0.95
'KI441020.1'	0.08	0.12	178.21	35.39	0.22	0.09	328343	0.47	1.97	0.90
'AUPR02S000(	0.24	0.08	144.22	12.48	0.13	0.04	1120178	8.79	1.55	0.94
'ANOI0100001	0.04	0.12	51.67	3.62	0.37	0.15	2769458	0.00	4.05	0.97
'ARWD010000(	0.00	0.24	24.86	2.44	0.30	0.21	5514320	0.00	9.00	0.96
'KI517384.1'	0.00	0.11	174.88	12.25	0.13	0.06	21755756	0.61	2.63	0.91
'KI911114.1'	0.00	0.22	515.34	215.20	0.03	0.03	2400105	1.51	4.30	0.69
'CP006831.1'	0.00	-1.00	13.60	0.62	0.46	0.11	6265484	0.00	12.18	0.97
'CP006832.1'	0.00	-1.00	13.60	0.62	0.46	0.11	6265484	0.00	12.18	0.97
'KI519610.1'	0.23	0.06	126.28	5.19	0.15	0.04	7371162	1.67	1.88	0.97
'KQ714883.1'	0.00	-1.00	72.99	3.38	0.23	0.03	1815678	9.08	4.64	0.95
'CM002264.1'	0.00	-1.00	12.95	0.65	0.52	0.13	5446806	0.02	12.07	0.96
'CM002261.1'	0.00	-1.00	12.06	0.48	0.61	0.13	5039408	0.03	12.14	0.98
'KI536661.1'	0.00	-1.00	185.60	2.84	0.28	0.01	51047765	1.60	1.66	0.97
'HG428755.1'	0.00	0.20	29.47	2.64	0.35	0.21	4984940	0.00	7.38	0.96
'KI537095.1'	0.10	0.04	389.25	10.67	0.16	0.03	51448160	6.02	0.84	0.97
'CP006844.1'	0.37	0.10	61.68	3.81	0.13	0.05	2091879	0.03	2.74	0.96
'KI538821.1'	0.11	0.06	154.05	7.05	0.22	0.06	8759900	1.53	1.64	0.97
'CM002268.1'	0.00	-1.00	11.41	0.44	0.72	0.14	5221300	0.04	12.17	0.98
'CP006846.1'	0.05	0.20	33.97	3.66	0.43	0.26	1198622	4.42	4.98	0.95
'CP006847.1'	0.06	0.21	33.63	4.00	0.43	0.27	1196760	7.24	4.84	0.95
'CM002271.1'	0.15	0.32	7.64	0.73	0.74	0.51	7026038	0.04	14.65	0.98
'CM002273.1'	0.30	0.28	7.10	0.55	0.59	0.35	7007517	0.03	14.67	0.99
'AUUT0100000	0.20	0.10	98.00	8.73	0.18	0.06	740036	7.12	2.28	0.95
'KI545873.1'	0.74	0.15	11.55	0.59	0.17	0.06	2209810	0.02	8.83	0.99
'KV441991.1'	0.62	0.09	56.93	3.13	0.06	0.02	1399177	0.00	2.44	0.97
'KI546166.1'	0.20	0.10	149.96	20.05	0.15	0.06	561775	0.08	1.85	0.92
'CM002277.1'	0.00	-1.00	8.46	0.33	0.63	0.12	3314966	0.12	17.41	0.99
'KI546415.1'	0.80	0.16	20.32	1.42	0.09	0.04	504181	0.81	5.16	0.98
'CM004511.1'	0.00	0.28	92.64	17.02	0.08	0.08	6191809	0.07	4.90	0.78
'CM002280.1'	0.05	0.27	8.13	0.67	0.84	0.49	8514960	1.77	14.61	0.99
'CM002285.1'	0.00	0.24	9.01	0.66	0.87	0.47	9663240	2.44	13.58	0.99
'KI547134.1'	0.47	0.09	129.54	9.42	0.06	0.02	1398958	0.91	1.29	0.93
'CM002287.1'	0.14	0.20	12.99	0.98	0.52	0.26	2372966	0.14	10.57	0.99
'ATNU0100040	0.41	0.15	24.94	1.94	0.19	0.08	676375	0.02	6.05	0.98
'AYRD0201233	0.00	0.27	411.03	187.48	0.02	0.02	6247470	0.38	5.86	0.64
'AWXF010000(	0.68	0.12	57.68	4.88	0.05	0.02	471424	2.56	2.22	0.94
'HG723041.1'	0.60	0.19	26.02	2.73	0.12	0.06	290450	0.00	4.84	0.95
'HG738137.1'	0.58	0.05	70.57	1.63	0.06	0.01	28345163	2.91	2.01	0.99
'HG670307.1'	0.19	0.17	47.96	6.59	0.24	0.12	249082	0.24	4.20	0.94
'HG688746.1'	0.38	0.17	37.50	4.84	0.17	0.08	155424	2.11	4.34	0.95
'HF969015.2'	0.00	0.19	25.51	2.17	0.36	0.20	4680283	0.06	8.52	0.97
'HG673746.1'	0.00	0.15	44.60	4.09	0.44	0.21	1462819	0.19	4.71	0.96
'HG530631.1'	0.75	0.12	24.05	1.19	0.09	0.03	1509152	4.87	4.50	0.98
'HG718748.1'	0.00	0.18	65.96	11.56	0.36	0.20	229542	0.04	3.93	0.92
'HG738131.1'	0.27	0.24	12.84	1.16	0.43	0.25	1480825	0.15	9.87	0.98
'HG710173.1'	0.89	0.15	25.47	2.09	0.06	0.02	242787	0.35	4.02	0.96
'HG678410.1'	0.70	0.20	21.19	2.50	0.11	0.05	143957	0.08	5.47	0.96
'CM002295.1'	0.00	0.04	231.51	6.44	0.22	0.05	59662532	9.72	1.69	0.98
'KV724674.1'	0.79	0.20	14.89	1.40	0.12	0.06	178165	0.04	7.02	0.97
'KV737129.1'	0.56	0.28	15.83	2.48	0.19	0.12	93857	1.25	7.52	0.95
'KI570111.1'	0.37	0.14	27.58	2.08	0.21	0.08	590747	0.03	5.53	0.98
'KI578484.1'	0.30	0.09	70.07	3.26	0.16	0.05	6831003	12.72	2.30	0.97
'KI615321.1'	0.14	0.05	371.92	14.74	0.15	0.03	18898765	1.73	0.77	0.96
'KI603969.1'	0.23	0.25	227.91	343.70	0.15	0.09	13405	11.64	3.01	0.77
'KI614512.1'	0.17	0.17	>1000	>1000	0.15	0.08	24166	2.53	2.15	0.77
'KI628541.1'	0.00	-1.00	72.68	4.18	0.36	0.04	591889	0.20	3.52	0.94
'KI629971.1'	0.00	-1.00	134.85	2.08	0.23	0.01	27400720	0.40	2.87	0.98
'KI630171.1'	0.00	-1.00	117.55	3.17	0.27	0.02	4921564	2.43	2.81	0.97
'KI635482.1'	0.35	0.14	29.29	2.28	0.21	0.08	760519	0.01	5.38	0.97
'AWYD010007(	0.85	0.18	16.27	1.20	0.10	0.05	528492	0.00	6.13	0.98
'KI633359.1'	0.80	0.16	16.10	1.13	0.11	0.05	529059	0.00	6.44	0.98
'KI633058.1'	0.45	0.19	21.52	2.11	0.20	0.09	352149	0.08	6.34	0.97
'KI635736.1'	0.61	0.07	148.32	8.04	0.03	0.01	2606237	2.18	0.98	0.94
'CM002307.1'	0.00	-1.00	11.78	0.33	0.73	0.11	5052399	0.57	11.83	0.99
'KI639851.1'	0.56	0.24	22.32	3.32	0.14	0.08	108276	2.93	5.64	0.94
'CP006735.1'	0.34	0.16	25.88	1.77	0.24	0.11	2520064	0.00	5.55	0.97
'KI652990.1'	0.39	0.17	12.92	0.75	0.33	0.14	3286380	3.93	8.94	0.99
'DF239015.1'	0.51	0.10	66.69	4.86	0.08	0.03	810747	0.30	2.38	0.96
'KI657455.1'	0.51	0.10	43.49	2.22	0.11	0.03	1890151	9.58	3.08	0.98
'KI669492.1'	0.67	0.14	17.45	0.92	0.14	0.05	1516621	0.14	6.42	0.99
'KI669518.1'	0.79	0.14	16.39	0.94	0.11	0.04	674405	0.35	6.42	0.99
'KI669472.1'	0.91	0.10	24.97	1.02	0.05	0.02	2541352	0.11	4.10	0.99
'KI669547.1'	0.95	0.11	23.82	0.99	0.05	0.02	3156845	0.39	4.14	0.98
'KI669459.1'	0.96	0.09	24.04	0.83	0.05	0.01	5292169	0.06	4.14	0.99
'CM002308.1'	0.72	0.11	43.11	2.50	0.06	0.02	1105101	1.15	2.75	0.97
'CM002309.1'	0.72	0.11	43.25	2.65	0.06	0.02	1106034	1.41	2.75	0.97
'CM002310.1'	0.72	0.11	43.20	2.51	0.06	0.02	1105305	1.32	2.75	0.97
'CM002311.1'	0.74	0.12	42.16	2.64	0.05	0.02	1105640	0.10	2.79	0.96
'DF396901.1'	0.80	0.11	38.63	2.34	0.05	0.02	956626	0.09	2.91	0.97
'DF396954.1'	0.75	0.11	40.44	2.37	0.06	0.02	1016420	0.26	2.92	0.97
'KI685158.1'	0.62	0.19	17.60	1.75	0.15	0.07	171016	0.01	6.81	0.97
'KI671675.1'	0.63	0.18	17.57	1.66	0.15	0.07	168797	0.00	6.83	0.97
'KI678687.1'	0.76	0.25	14.32	1.67	0.13	0.08	155734	0.00	7.22	0.96
'KI691933.1'	0.66	0.25	15.52	1.86	0.15	0.09	159111	0.00	7.16	0.96
'KI697133.1'	0.23	0.10	153.08	18.79	0.14	0.05	507103	0.61	1.69	0.92
'KI744491.1'	0.24	0.09	129.65	10.35	0.14	0.05	1269357	0.72	1.81	0.94
'KI723259.1'	0.00	0.10	299.49	77.82	0.25	0.09	319730	1.18	1.74	0.90
'KI784202.1'	0.27	0.09	128.55	12.18	0.13	0.04	705117	1.29	1.80	0.94
'CM002317.1'	0.00	-1.00	198.47	4.69	0.19	0.01	55940097	13.95	1.64	0.94
'KQ207791.1'	0.26	0.26	14.14	1.57	0.40	0.25	656358	6.48	8.74	0.97
'CM002326.2'	0.00	-1.00	212.51	5.14	0.16	0.01	60962716	8.90	1.75	0.94
'BASJ020000(	0.45	0.04	254.03	7.71	0.05	0.01	18791394	2.41	0.70	0.96
'BATS0100000	0.43	0.18	36.89	4.15	0.15	0.08	348406	0.00	4.09	0.94
'DF277771.1'	0.23	0.17	361.74	331.52	0.12	0.06	51398	2.02	1.89	0.77
'BATU0104605	0.33	0.15	384.64	359.39	0.09	0.04	36387	0.00	1.82	0.78
'BATW010927(	0.23	0.20	>1000	>1000	0.11	0.06	27253	0.00	2.00	0.66

'BATV0102700	0.63	0.16	377.93	345.12	0.03	0.01	32113	0.00	1.31	0.58
'KI867152.1'	0.35	0.05	156.38	4.80	0.09	0.02	18549843	1.79	1.20	0.98
'CM002330.1'	0.00	0.38	24.49	3.80	0.16	0.18	6226470	0.07	10.32	0.90
'DF340865.1'	0.00	0.11	107.77	10.73	0.30	0.12	1287144	1.50	2.75	0.94
'KI894027.1'	0.00	-1.00	32.23	1.72	0.47	0.09	3950590	0.02	5.46	0.94
'KI894018.1'	0.87	0.10	22.95	0.85	0.06	0.02	4914953	0.03	4.53	0.99
'KI894007.1'	0.00	0.16	126.64	15.72	0.13	0.08	3276357	0.04	3.48	0.88
'CM002361.1'	0.18	0.06	127.78	3.92	0.18	0.04	25289714	1.10	1.83	0.98
'HG380758.1'	0.83	0.10	44.16	2.20	0.04	0.01	1087316	0.60	2.53	0.97
'HG792015.1'	0.21	0.10	32.19	1.36	0.29	0.09	8605340	3.90	5.12	0.99
'HG793134.1'	0.28	0.11	33.56	1.76	0.23	0.08	2612452	1.41	5.00	0.98
'CBVU0100000	0.00	-1.00	26.33	1.08	0.43	0.06	1883535	0.00	7.65	0.97
'CBVT0100000	0.00	-1.00	26.33	1.08	0.43	0.06	1883685	0.00	7.65	0.97
'CBVS0100000	0.00	-1.00	26.33	1.08	0.43	0.06	1883735	0.00	7.65	0.97
'KI911139.1'	0.00	-1.00	42.15	2.39	0.24	0.05	3597120	0.38	6.43	0.92
'AZIM0100008(	0.23	0.06	330.31	26.03	0.11	0.03	2844733	8.52	0.81	0.93
'KI912109.1'	0.39	0.12	27.51	1.32	0.20	0.08	7233875	0.11	5.36	0.98
'BATX0113826	0.37	0.31	269.97	649.81	0.09	0.07	12564	0.00	2.94	0.61
'AWNI0100000	0.32	0.17	12.66	0.80	0.38	0.16	2380867	0.98	10.02	0.99
'KI894189.1'	0.00	-1.00	285.30	13.55	0.14	0.01	5191289	5.33	1.73	0.90
'KI913114.1'	0.00	-1.00	27.58	1.04	0.45	0.06	2615878	11.72	6.77	0.97
'KI913954.1'	0.00	-1.00	21.41	0.77	0.49	0.07	3119476	35.22	5.61	0.98
'DF260912.1'	0.00	-1.00	10.39	0.36	0.77	0.13	4130470	0.56	13.00	0.99
'KI925184.1'	0.54	0.07	291.07	27.39	0.03	0.01	1702284	20.77	0.52	0.87
'KI925625.1'	0.53	0.08	353.18	51.78	0.03	0.01	884624	18.81	0.51	0.80
'KI926445.1'	0.51	0.09	441.38	95.96	0.03	0.01	584965	24.55	0.48	0.76
'KI926126.1'	0.52	0.06	283.49	20.33	0.03	0.01	2449757	10.98	0.57	0.90
'KI927384.1'	0.57	0.06	238.46	13.98	0.03	0.01	3543875	19.30	0.55	0.92
'KI927620.1'	0.52	0.07	285.67	21.86	0.03	0.01	2395713	11.27	0.57	0.89
'KI928025.1'	0.61	0.09	293.22	50.79	0.02	0.01	635193	39.54	0.44	0.77
'CM002372.1'	0.50	0.11	54.20	3.12	0.10	0.03	1995662	0.65	2.72	0.97
'CM002369.1'	0.54	0.10	53.26	2.93	0.09	0.03	1736780	0.23	2.70	0.97
'CM002370.1'	0.56	0.11	52.10	3.19	0.08	0.03	1778760	0.37	2.69	0.96
'CM002371.1'	0.57	0.09	51.57	2.66	0.08	0.03	1820209	0.33	2.70	0.97
'BASO0100293	0.25	0.13	122.92	25.20	0.14	0.06	173717	8.60	2.02	0.89
'CM002373.1'	0.10	0.05	133.56	3.93	0.24	0.05	34528841	5.04	1.87	0.98
'KI964537.1'	0.50	0.18	22.72	1.97	0.17	0.08	501469	0.03	5.87	0.97
'KI963918.1'	0.34	0.17	25.76	2.24	0.23	0.11	638489	0.01	5.89	0.97
'KI963911.1'	0.26	0.13	31.22	1.86	0.25	0.10	4062904	0.15	5.34	0.98
'KI965460.1'	0.45	0.11	40.74	2.22	0.13	0.05	1930430	1.49	3.70	0.98
'KI965394.1'	0.68	0.07	128.74	6.12	0.03	0.01	2452783	0.53	1.04	0.95
'JATN0100032:	0.00	-1.00	132.53	8.47	0.03	0.01	19047957	7.57	4.93	0.83
'BASP0101026	0.30	0.15	132.04	36.47	0.11	0.05	110969	2.95	2.00	0.85
'KI966371.1'	0.38	0.16	21.15	1.62	0.23	0.10	1018719	0.10	6.74	0.98
'JAQJ0101062:	0.25	0.35	45.70	30.17	0.21	0.16	9282	0.00	5.82	0.84
'JAQR0100256	0.18	0.32	>1000	>1000	0.16	0.12	5276	0.00	4.11	0.71
'KI968691.1'	0.53	0.15	22.75	1.59	0.16	0.07	870365	0.06	5.77	0.98
'HG803175.1'	0.00	-1.00	15.78	0.79	0.60	0.13	2111919	0.87	9.80	0.97
'CBTC0100000	0.65	0.15	34.54	2.79	0.08	0.04	806952	0.05	3.60	0.96
'HG530068.1'	0.00	-1.00	15.74	0.71	0.36	0.08	7586152	5.72	11.18	0.96
'HG009209.1'	0.42	0.03	423.43	7.99	0.04	0.01	88717973	0.57	0.49	0.97
'CM002401.1'	0.33	0.10	51.75	2.54	0.17	0.06	5228314	0.01	3.22	0.98
'CM002398.1'	0.33	0.10	52.01	2.60	0.17	0.06	5232192	0.10	3.21	0.97
'CM002395.1'	0.33	0.10	51.74	2.57	0.17	0.06	5227966	0.01	3.22	0.97
'CP006940.1'	0.26	0.11	43.31	2.46	0.22	0.08	2975051	0.06	3.97	0.98
'CM002407.1'	0.78	0.11	40.04	2.13	0.05	0.02	1475168	1.33	2.82	0.97
'CM002425.1'	0.76	0.11	40.32	2.22	0.05	0.02	1466790	2.13	2.80	0.97
'CM002335.1'	0.81	0.10	39.00	1.93	0.05	0.02	1467382	2.07	2.80	0.97
'CM002441.1'	0.81	0.11	38.69	2.08	0.05	0.02	1458252	1.12	2.83	0.97
'CP005829.1'	0.71	0.08	152.16	12.14	0.02	0.01	904790	0.00	0.92	0.89
'CP004146.1'	0.70	0.08	151.86	11.28	0.02	0.01	919292	0.00	0.92	0.91
'CP004217.2'	0.68	0.08	183.05	16.35	0.02	0.01	904875	0.00	0.81	0.87
'CP004267.1'	0.66	0.09	211.70	20.92	0.02	0.01	916000	0.00	0.74	0.85
'CP005851.2'	0.71	0.09	162.81	13.83	0.02	0.01	917680	0.00	0.87	0.88
'CP005745.1'	0.70	0.08	158.31	12.87	0.02	0.01	912832	0.00	0.90	0.89
'CP005706.1'	0.75	0.08	137.37	10.28	0.02	0.01	919983	0.00	0.95	0.90
'CP005680.1'	0.71	0.08	148.96	10.90	0.02	0.01	920644	0.00	0.92	0.91
'CM002455.1'	0.51	0.14	20.28	1.22	0.18	0.07	1546440	0.41	6.46	0.98
'KQ464259.1'	0.35	0.07	71.37	2.76	0.13	0.04	7146707	6.45	2.38	0.98
'CM003136.1'	0.55	0.10	53.33	3.04	0.08	0.03	1782640	0.35	2.69	0.97
'CM003135.1'	0.58	0.10	51.06	2.78	0.08	0.03	1834035	0.40	2.71	0.97
'KK060624.1'	0.25	0.12	112.40	14.70	0.14	0.06	561146	17.24	1.66	0.91
'CM002488.1'	0.00	-1.00	107.61	1.63	0.20	0.01	46096743	0.44	3.85	0.98
'CM002500.1'	0.00	-1.00	111.41	1.73	0.20	0.01	42845077	5.79	3.67	0.98
'CM002512.1'	0.00	-1.00	110.31	1.68	0.20	0.01	46529941	17.59	3.22	0.98
'CP007225.1'	0.05	0.28	12.51	1.31	0.67	0.46	2773134	0.02	10.74	0.98
'CP006963.1'	0.08	0.11	60.20	3.92	0.33	0.13	4233806	2.85	3.30	0.97
'HG793125.1'	0.81	0.12	28.25	1.49	0.06	0.02	2374218	0.32	3.88	0.98
'HG814183.1'	0.22	0.13	35.85	2.72	0.26	0.11	1273777	0.00	5.09	0.97
'HG916852.1'	0.40	0.19	10.02	0.58	0.38	0.17	4195305	0.00	11.42	0.99
'HG815136.1'	0.30	0.14	32.37	2.42	0.22	0.09	930736	0.00	5.18	0.97
'HG816029.1'	0.48	0.23	28.41	4.61	0.15	0.09	107387	1.55	4.95	0.93
'HG813308.1'	0.33	0.14	29.41	2.03	0.22	0.09	1211662	0.00	5.39	0.98
'HG813601.1'	0.31	0.12	32.30	1.98	0.22	0.08	1223141	0.00	5.07	0.98
'HF955061.1'	0.00	0.13	44.27	3.55	0.37	0.15	1675870	6.70	5.40	0.97
'CBMN010001!	0.00	-1.00	139.01	25.04	0.27	0.04	73183	0.00	3.52	0.89
'JARH0100001	0.51	0.19	21.52	1.98	0.17	0.08	596408	0.02	6.14	0.97
'CP007236.1'	0.00	0.27	11.10	1.04	0.77	0.49	3956045	0.02	12.20	0.98
'CP007268.1'	0.60	0.16	12.38	0.66	0.22	0.09	3460134	0.39	8.57	0.99
'CP006980.1'	0.00	-1.00	13.11	0.56	0.53	0.11	6543350	0.01	12.10	0.97
'CP006981.1'	0.00	-1.00	12.80	0.55	0.55	0.12	6467914	0.00	12.14	0.97
'CP006982.1'	0.00	-1.00	13.26	0.55	0.52	0.11	6591121	0.00	12.08	0.97
'CP006983.1'	0.00	-1.00	13.09	0.55	0.53	0.11	6527005	0.00	12.11	0.97
'CP006984.1'	0.00	-1.00	13.03	0.55	0.54	0.12	6509070	0.01	12.10	0.97
'CP006985.1'	0.00	-1.00	13.11	0.56	0.53	0.11	6524053	0.00	12.10	0.97
'AMGW010000	0.60	0.13	16.81	0.73	0.17	0.06	5349221	0.02	6.83	0.99
'AMGX010000(	0.69	0.11	19.20	0.74	0.12	0.04	3803025	0.05	5.92	0.99
'AMGY010000(	0.00	-1.00	32.30	1.14	0.42	0.06	4994026	0.27	6.24	0.97
'AMWN010000	0.25	0.15	24.70	1.57	0.30	0.13	4604234	0.05	6.19	0.98
'AZCJ0100000	0.70	0.10	37.70	1.99	0.07	0.02	1262187	0.94	3.23	0.98
'KK076434.1'	0.63	0.10	45.80	2.43	0.07	0.03	1671243	0.12	2.85	0.97
'CM002545.1'	0.00	-1.00	12.12	0.50	0.63	0.13	5011687	0.20	11.96	0.98
'CM002673.1'	0.00	-1.00	11.96	0.53	0.64	0.15	4918955	0.20	12.00	0.97
'CM002635.1'	0.00	-1.00	12.10	0.46	0.63	0.13	4967288	0.02	12.00	0.98
'CM002636.1'	0.00	-1.00	11.97	0.48	0.66	0.14	4925775	0.01	11.99	0.98
'CM002637.1'	0.00	-1.00	11.40	0.47	0.70	0.15	4911500	0.02	12.09	0.98

'CM002638.1'	0.00	-1.00	11.94	0.41	0.67	0.12	5053472	0.02	11.96	0.98
'CP006672.1'	0.78	0.10	33.42	1.69	0.06	0.02	1042619	0.02	3.38	0.98
'CP006675.1'	0.78	0.10	33.44	1.66	0.06	0.02	1043010	0.10	3.37	0.98
'CP006677.1'	0.78	0.11	33.41	1.85	0.06	0.02	1041595	0.03	3.38	0.97
'CP006678.1'	0.79	0.11	33.25	1.82	0.06	0.02	1043181	0.00	3.38	0.98
'CP006679.1'	0.78	0.12	33.31	1.98	0.06	0.02	1042783	0.10	3.38	0.97
'CM002782.1'	0.36	0.12	31.75	1.70	0.19	0.07	4204192	2.03	4.72	0.98
'KK076539.1'	0.00	0.09	178.30	13.52	0.28	0.11	7289689	25.66	1.28	0.93
'JELW0100000	0.00	0.18	30.72	2.50	0.50	0.28	5053378	0.01	5.97	0.97
'JAQW010000	0.25	0.14	215.93	87.35	0.12	0.06	79274	0.00	1.84	0.83
'JAQX0100000	0.18	0.15	488.07	442.29	0.14	0.06	69844	0.01	1.73	0.80
'JAQY0100000	0.16	0.14	266.42	112.62	0.15	0.07	107227	0.00	1.77	0.84
'JAQZ0100000	0.25	0.13	129.72	26.72	0.14	0.06	171681	0.03	2.04	0.89
'JARA0100000	0.26	0.13	123.73	22.20	0.14	0.06	208614	0.00	1.98	0.89
'JARB0100000	0.27	0.13	121.94	21.75	0.13	0.06	214169	0.00	1.95	0.89
'JEMT0102776	0.05	0.13	217.72	59.79	0.23	0.10	198953	0.01	1.93	0.88
'CM002651.1'	0.14	0.13	35.25	1.95	0.34	0.15	11267849	0.26	4.91	0.98
'AWGT010004	0.35	0.08	386.47	70.58	0.06	0.02	600658	5.49	0.76	0.84
'AWGU010318	0.88	0.17	14.92	1.19	0.10	0.04	163812	0.00	6.57	0.98
'KK082680.1'	0.00	0.11	91.54	6.12	0.17	0.08	8792355	7.22	4.10	0.95
'KK088411.1'	0.20	0.13	33.71	2.35	0.28	0.12	1563877	0.12	5.33	0.98
'KK090061.1'	0.46	0.17	24.15	2.01	0.18	0.09	768125	0.13	5.63	0.97
'KK090494.1'	0.00	-1.00	134.03	3.14	0.27	0.02	7482359	4.06	2.41	0.97
'HF972804.1'	0.49	0.17	21.43	1.72	0.18	0.08	619733	1.46	6.18	0.97
'HF970592.1'	0.38	0.17	25.31	2.29	0.21	0.10	588491	0.59	5.96	0.97
'HF975737.1'	0.43	0.17	23.82	2.09	0.19	0.08	522423	2.40	5.98	0.97
'HF979336.1'	0.54	0.19	20.49	1.88	0.17	0.08	402084	2.42	6.12	0.97
'CAOW01009	0.92	0.12	>1000	>1000	0.01	0.00	36400	1.44	0.60	0.12
'CM002678.1'	0.21	0.14	108.21	22.35	0.16	0.07	135362	0.00	2.56	0.90
'KK100010.1'	0.58	0.13	30.49	2.24	0.11	0.04	618369	3.18	4.26	0.97
'KK106941.1'	0.31	0.14	28.99	1.73	0.24	0.10	3800214	2.05	5.05	0.98
'KK106981.1'	0.00	-1.00	>1000	269.00	0.01	0.00	10671843	0.72	4.52	0.72
'KK107063.1'	0.00	-1.00	43.71	1.38	0.23	0.03	4675608	1.37	7.47	0.97
'KK115971.1'	0.00	-1.00	115.15	3.98	0.29	0.03	4549800	1.08	2.48	0.95
'KK198108.1'	0.00	-1.00	42.76	1.24	0.31	0.03	6439362	6.63	5.79	0.97
'CM002679.1'	0.00	0.05	152.38	3.59	0.30	0.06	147124936	7.53	1.91	0.98
'KK198755.1'	0.11	0.05	93.49	2.24	0.25	0.05	80079441	8.76	2.48	0.99
'HG803548.1'	0.36	0.16	27.31	2.04	0.22	0.11	1771200	0.18	5.31	0.97
'CBZK0100000	0.21	0.08	>1000	>1000	0.09	0.03	391865	8.26	0.68	0.79
'HG805809.1'	0.68	0.14	29.95	2.24	0.09	0.03	533758	0.00	4.07	0.97
'HG934063.1'	0.37	0.10	31.56	1.43	0.19	0.06	4142257	0.00	4.82	0.99
'KK203934.1'	0.34	0.16	32.23	2.88	0.20	0.10	685249	0.00	4.89	0.96
'KK204238.1'	0.40	0.22	31.26	5.52	0.17	0.10	100620	0.00	5.17	0.93
'KK207357.1'	0.45	0.18	29.25	2.98	0.16	0.08	502909	0.00	4.91	0.96
'KK207889.1'	0.44	0.16	29.92	2.62	0.16	0.07	598948	0.00	4.87	0.97
'KK208427.1'	0.43	0.15	29.97	2.54	0.17	0.07	664234	0.00	4.94	0.97
'KK208846.1'	0.44	0.16	30.44	2.59	0.16	0.07	596576	0.00	4.72	0.97
'KK209394.1'	0.43	0.15	29.89	2.50	0.17	0.07	667787	0.00	4.93	0.97
'KK209984.1'	0.57	0.13	27.72	1.78	0.12	0.05	825833	0.00	4.75	0.98
'KK210299.1'	0.45	0.13	28.60	1.70	0.16	0.06	1440740	0.03	4.98	0.98
'KK210757.1'	0.50	0.14	28.10	1.98	0.15	0.06	795547	0.00	4.88	0.97
'KL203742.1'	0.47	0.04	393.10	13.74	0.04	0.01	15899586	5.73	0.45	0.95
'CP007270.2'	0.00	-1.00	28.27	1.21	0.29	0.05	4685848	0.87	8.45	0.96
'CP007274.2'	0.00	-1.00	28.27	1.21	0.29	0.05	4685841	0.87	8.45	0.96
'CP007266.2'	0.00	-1.00	28.27	1.21	0.29	0.05	4685848	0.84	8.45	0.96
'CP007362.2'	0.00	-1.00	28.27	1.21	0.29	0.05	4685841	0.87	8.45	0.96
'CP007363.2'	0.00	-1.00	28.27	1.21	0.29	0.05	4685901	0.87	8.45	0.96
'CP007364.2'	0.00	-1.00	28.26	1.21	0.29	0.05	4685846	0.87	8.45	0.96
'CP007289.2'	0.00	-1.00	28.27	1.21	0.29	0.05	4685775	0.84	8.45	0.96
'CP007290.2'	0.00	-1.00	28.27	1.21	0.29	0.05	4685848	0.81	8.45	0.96
'CP007293.2'	0.00	-1.00	28.26	1.21	0.29	0.05	4685838	0.87	8.45	0.96
'CP007294.2'	0.00	-1.00	28.24	1.20	0.29	0.05	4685658	0.00	8.51	0.96
'CP007295.2'	0.00	-1.00	28.27	1.21	0.29	0.05	4686394	0.86	8.45	0.96
'CP007277.2'	0.00	-1.00	28.27	1.21	0.29	0.05	4685848	0.87	8.45	0.96
'CP007278.2'	0.00	-1.00	28.27	1.21	0.29	0.05	4685847	0.87	8.45	0.96
'CP007279.2'	0.00	-1.00	28.27	1.21	0.29	0.05	4685848	0.81	8.45	0.96
'CP007281.2'	0.00	-1.00	28.27	1.21	0.29	0.05	4685848	0.84	8.45	0.96
'CP007284.2'	0.00	-1.00	28.26	1.21	0.29	0.05	4685849	0.85	8.45	0.96
'CP007320.2'	0.00	-1.00	28.27	1.21	0.29	0.05	4686535	0.88	8.45	0.96
'CP007321.2'	0.00	-1.00	28.27	1.21	0.29	0.05	4685850	0.84	8.45	0.96
'CP007322.2'	0.00	-1.00	28.27	1.21	0.29	0.05	4685850	0.84	8.45	0.96
'CP007300.2'	0.00	-1.00	28.27	1.21	0.29	0.05	4685839	0.88	8.45	0.96
'CP007301.2'	0.00	-1.00	28.27	1.21	0.29	0.05	4685841	0.87	8.45	0.96
'CP007302.2'	0.00	-1.00	28.27	1.21	0.29	0.05	4685836	0.87	8.45	0.96
'CP007303.2'	0.00	-1.00	28.27	1.21	0.29	0.05	4685905	0.87	8.45	0.96
'CP007304.2'	0.00	-1.00	28.27	1.21	0.29	0.05	4685845	0.88	8.45	0.96
'CP007305.2'	0.00	-1.00	28.27	1.21	0.29	0.05	4685907	0.87	8.45	0.96
'CP007306.2'	0.00	-1.00	28.27	1.21	0.29	0.05	4685844	0.87	8.45	0.96
'CP007307.2'	0.00	-1.00	28.27	1.21	0.29	0.05	4685841	0.87	8.45	0.96
'CP007308.2'	0.00	-1.00	28.27	1.21	0.29	0.05	4685842	0.88	8.45	0.96
'CP007309.2'	0.00	-1.00	28.27	1.21	0.29	0.05	4685844	0.87	8.45	0.96
'CP007310.2'	0.00	-1.00	28.27	1.21	0.29	0.05	4685841	0.87	8.45	0.96
'CP007311.2'	0.00	-1.00	28.26	1.21	0.29	0.05	4685841	0.82	8.45	0.96
'CP007313.2'	0.00	-1.00	28.27	1.21	0.29	0.05	4685848	0.85	8.45	0.96
'CP007314.2'	0.00	-1.00	28.27	1.21	0.29	0.05	4685842	0.87	8.45	0.96
'CP007315.2'	0.00	-1.00	28.26	1.21	0.29	0.05	4685836	0.87	8.45	0.96
'CP007316.2'	0.00	-1.00	28.27	1.21	0.29	0.05	4685844	0.87	8.45	0.96
'CP007334.2'	0.00	-1.00	28.26	1.21	0.29	0.05	4685836	0.87	8.45	0.96
'CP007317.2'	0.00	-1.00	28.26	1.21	0.29	0.05	4685843	0.87	8.45	0.96
'CP007318.2'	0.00	-1.00	28.26	1.21	0.29	0.05	4685844	0.87	8.45	0.96
'CP007319.2'	0.00	-1.00	28.25	1.20	0.29	0.05	4685849	0.00	8.51	0.96
'CP007323.2'	0.00	-1.00	28.18	1.19	0.29	0.05	4686198	0.85	8.45	0.96
'CP007324.2'	0.00	-1.00	28.27	1.21	0.29	0.05	4685845	0.87	8.45	0.96
'CP007325.2'	0.00	-1.00	28.27	1.21	0.29	0.05	4685847	0.87	8.45	0.96
'CP007326.2'	0.00	-1.00	28.26	1.21	0.29	0.05	4685846	0.87	8.45	0.96
'CP007327.2'	0.00	-1.00	28.27	1.21	0.29	0.05	4685848	0.84	8.45	0.96
'CP007328.2'	0.00	-1.00	28.27	1.21	0.29	0.05	4685845	0.87	8.45	0.96
'CP007330.2'	0.00	-1.00	28.27	1.21	0.29	0.05	4685974	0.87	8.45	0.96
'CP007331.2'	0.00	-1.00	28.27	1.21	0.29	0.05	4685849	0.84	8.45	0.96
'CP007344.2'	0.00	-1.00	28.27	1.21	0.29	0.05	4685846	0.84	8.45	0.96
'CP007345.2'	0.00	-1.00	28.27	1.21	0.29	0.05	4685842	0.87	8.45	0.96
'CP007346.2'	0.00	-1.00	28.27	1.21	0.29	0.05	4685832	0.87	8.45	0.96
'CP007347.2'	0.00	-1.00	28.28	1.21	0.29	0.05	4685839	0.91	8.44	0.96
'CP007348.2'	0.00	-1.00	28.27	1.21	0.29	0.05	4686185	0.87	8.45	0.96
'CP007349.2'	0.00	-1.00	28.26	1.21	0.29	0.05	4685845	0.87	8.45	0.96
'CP007350.2'	0.00	-1.00	28.26	1.21	0.29	0.05	4685847	0.87	8.45	0.96

'CP007351.2'	0.00	-1.00	28.26	1.21	0.29	0.05	4685848	0.87	8.45	0.96
'CP007352.2'	0.00	-1.00	28.27	1.21	0.29	0.05	4685833	0.87	8.45	0.96
'CP007353.2'	0.00	-1.00	28.27	1.21	0.29	0.05	4685834	0.87	8.45	0.96
'CP007354.2'	0.00	-1.00	28.27	1.21	0.29	0.05	4685835	0.87	8.45	0.96
'CP007365.2'	0.00	-1.00	28.26	1.20	0.29	0.05	4686395	0.86	8.45	0.96
'CP007366.2'	0.00	-1.00	28.27	1.21	0.29	0.05	4685832	0.87	8.45	0.96
'CP007367.2'	0.00	-1.00	28.27	1.21	0.29	0.05	4685975	0.87	8.45	0.96
'CP007368.2'	0.00	-1.00	28.27	1.21	0.29	0.05	4685840	0.87	8.45	0.96
'CP007369.2'	0.00	-1.00	28.29	1.21	0.29	0.05	4685835	0.87	8.45	0.96
'CP007370.2'	0.00	-1.00	28.27	1.21	0.29	0.05	4685833	0.86	8.45	0.96
'CP007371.2'	0.00	-1.00	28.27	1.21	0.29	0.05	4685786	0.87	8.45	0.96
'CP007372.2'	0.00	-1.00	28.27	1.21	0.29	0.05	4685840	0.88	8.45	0.96
'CP007373.2'	0.00	-1.00	28.26	1.21	0.29	0.05	4685838	0.87	8.45	0.96
'CP007374.2'	0.00	-1.00	28.27	1.21	0.29	0.05	4685847	0.84	8.45	0.96
'CP007395.2'	0.00	-1.00	28.27	1.21	0.29	0.05	4685847	0.84	8.45	0.96
'CP007396.2'	0.00	-1.00	28.27	1.21	0.29	0.05	4685838	0.87	8.45	0.96
'CP007397.2'	0.00	-1.00	28.27	1.21	0.29	0.05	4685843	0.86	8.45	0.96
'CP007398.2'	0.00	-1.00	28.26	1.21	0.29	0.05	4685844	0.87	8.45	0.96
'CP007400.2'	0.00	-1.00	28.27	1.21	0.29	0.05	4686021	0.84	8.45	0.96
'CP007401.2'	0.00	-1.00	28.27	1.21	0.29	0.05	4685847	0.84	8.45	0.96
'CP007402.2'	0.00	-1.00	28.26	1.21	0.29	0.05	4685984	0.87	8.45	0.96
'CP007403.2'	0.00	-1.00	28.28	1.21	0.29	0.05	4685845	0.93	8.44	0.96
'CP007404.2'	0.00	-1.00	28.27	1.21	0.29	0.05	4685845	0.88	8.45	0.96
'CP007405.2'	0.00	-1.00	28.27	1.21	0.29	0.05	4685845	0.87	8.45	0.96
'CP007406.2'	0.00	-1.00	28.27	1.21	0.29	0.05	4685836	0.88	8.45	0.96
'CP007407.2'	0.00	-1.00	28.27	1.21	0.29	0.05	4685847	0.84	8.45	0.96
'CP007408.2'	0.00	-1.00	28.27	1.21	0.29	0.05	4685848	0.84	8.45	0.96
'CP007375.2'	0.00	-1.00	28.27	1.21	0.29	0.05	4685848	0.84	8.45	0.96
'CP007376.2'	0.00	-1.00	28.27	1.21	0.29	0.05	4685848	0.84	8.45	0.96
'CP007377.2'	0.00	-1.00	28.27	1.21	0.29	0.05	4685843	0.86	8.45	0.96
'CP007378.2'	0.00	-1.00	28.26	1.21	0.29	0.05	4686258	0.87	8.45	0.96
'CP007379.2'	0.00	-1.00	28.27	1.21	0.29	0.05	4685833	0.87	8.45	0.96
'CP007380.2'	0.00	-1.00	28.27	1.21	0.29	0.05	4685838	0.87	8.45	0.96
'CP007381.2'	0.00	-1.00	28.27	1.21	0.29	0.05	4685847	0.84	8.45	0.96
'CP007382.2'	0.00	-1.00	28.27	1.21	0.29	0.05	4685838	0.87	8.45	0.96
'CP007383.2'	0.00	-1.00	28.27	1.21	0.29	0.05	4685698	0.87	8.45	0.96
'CP007384.2'	0.00	-1.00	28.27	1.21	0.29	0.05	4685842	0.87	8.45	0.96
'CP007385.2'	0.00	-1.00	28.28	1.21	0.29	0.05	4685838	0.87	8.45	0.96
'CP007296.2'	0.00	-1.00	28.27	1.21	0.29	0.05	4685839	0.87	8.45	0.96
'CP007297.2'	0.00	-1.00	28.27	1.21	0.29	0.05	4685841	0.91	8.45	0.96
'CP007298.2'	0.00	-1.00	28.27	1.21	0.29	0.05	4685841	0.87	8.45	0.96
'CP007355.2'	0.00	-1.00	28.27	1.21	0.29	0.05	4685845	0.84	8.45	0.96
'CP007356.2'	0.00	-1.00	28.27	1.21	0.29	0.05	4685847	0.85	8.45	0.96
'CP007335.2'	0.00	-1.00	28.27	1.21	0.29	0.05	4685833	0.87	8.45	0.96
'CP007336.2'	0.00	-1.00	28.27	1.21	0.29	0.05	4685843	0.88	8.45	0.96
'CP007337.2'	0.00	-1.00	28.27	1.21	0.29	0.05	4685840	0.88	8.45	0.96
'CP007338.2'	0.00	-1.00	28.27	1.21	0.29	0.05	4685845	0.87	8.45	0.96
'CP007339.2'	0.00	-1.00	28.18	1.18	0.29	0.05	4685833	1.12	8.42	0.96
'CP007340.2'	0.00	-1.00	28.27	1.21	0.29	0.05	4685835	0.86	8.45	0.96
'CP007341.2'	0.00	-1.00	28.26	1.21	0.29	0.05	4685832	0.87	8.45	0.96
'CP007342.2'	0.00	-1.00	28.26	1.21	0.29	0.05	4685838	0.87	8.45	0.96
'CP007343.2'	0.00	-1.00	28.27	1.21	0.29	0.05	4685843	0.87	8.45	0.96
'CP007386.2'	0.00	-1.00	28.27	1.21	0.29	0.05	4685839	0.87	8.45	0.96
'CP007426.2'	0.00	-1.00	28.24	1.20	0.29	0.05	4685834	0.00	8.51	0.96
'CP007430.2'	0.00	-1.00	28.27	1.21	0.29	0.05	4685846	0.84	8.45	0.96
'CP007431.2'	0.00	-1.00	28.27	1.21	0.29	0.05	4685841	0.87	8.45	0.96
'CP007432.2'	0.00	-1.00	28.27	1.21	0.29	0.05	4685847	0.84	8.45	0.96
'CP007427.2'	0.00	-1.00	28.27	1.21	0.29	0.05	4685849	0.85	8.45	0.96
'CP007428.2'	0.00	-1.00	28.27	1.21	0.29	0.05	4685846	0.84	8.45	0.96
'CP007429.2'	0.00	-1.00	28.27	1.21	0.29	0.05	4685848	0.86	8.45	0.96
'CP007387.2'	0.00	-1.00	28.27	1.21	0.29	0.05	4685785	0.87	8.45	0.96
'CP007388.2'	0.00	-1.00	28.27	1.21	0.29	0.05	4685848	0.87	8.45	0.96
'CP007433.2'	0.00	-1.00	28.27	1.21	0.29	0.05	4685846	0.93	8.44	0.96
'CP007411.2'	0.00	-1.00	28.27	1.21	0.29	0.05	4685848	0.87	8.45	0.96
'CP007412.2'	0.00	-1.00	28.27	1.21	0.29	0.05	4685847	0.84	8.45	0.96
'CP007413.2'	0.00	-1.00	28.27	1.21	0.29	0.05	4685785	0.87	8.45	0.96
'CP007414.2'	0.00	-1.00	28.27	1.21	0.29	0.05	4685880	0.84	8.45	0.96
'CP007415.2'	0.00	-1.00	27.94	1.17	0.29	0.05	4685839	0.92	8.45	0.96
'CP007416.2'	0.00	-1.00	28.35	1.23	0.28	0.05	4685841	0.88	8.45	0.96
'CP007417.2'	0.00	-1.00	27.95	1.16	0.29	0.05	4685793	0.87	8.45	0.96
'CP007418.2'	0.00	-1.00	28.27	1.21	0.29	0.05	4685849	0.84	8.45	0.96
'CP007419.2'	0.00	-1.00	28.27	1.21	0.29	0.05	4685836	0.88	8.45	0.96
'CP007423.2'	0.00	-1.00	28.27	1.21	0.29	0.05	4685847	0.84	8.45	0.96
'CP007424.2'	0.00	-1.00	28.27	1.21	0.29	0.05	4685849	0.87	8.45	0.96
'CP007420.2'	0.00	-1.00	28.27	1.21	0.29	0.05	4685835	0.87	8.45	0.96
'CP007421.2'	0.00	-1.00	28.27	1.21	0.29	0.05	4685838	0.87	8.45	0.96
'CP007422.2'	0.00	-1.00	28.27	1.21	0.29	0.05	4685834	0.87	8.45	0.96
'CP007438.2'	0.00	-1.00	28.22	1.20	0.29	0.05	4685849	0.84	8.45	0.96
'CP007463.2'	0.00	-1.00	27.99	1.19	0.29	0.05	4685736	0.99	8.43	0.96
'CP007464.2'	0.00	-1.00	28.29	1.19	0.28	0.05	4685731	0.89	8.45	0.96
'CP007465.1'	0.00	-1.00	28.30	1.21	0.29	0.05	4707072	1.45	8.40	0.96
'CP007466.2'	0.00	-1.00	28.27	1.21	0.29	0.05	4685836	0.86	8.45	0.96
'CP007467.2'	0.00	-1.00	28.28	1.21	0.29	0.05	4685836	1.48	8.40	0.96
'CP007468.2'	0.00	-1.00	28.27	1.21	0.29	0.05	4685839	0.88	8.45	0.96
'CP007469.2'	0.00	-1.00	28.27	1.21	0.29	0.05	4685835	0.87	8.45	0.96
'CP007498.2'	0.00	-1.00	28.27	1.21	0.29	0.05	4685842	0.87	8.45	0.96
'CP007224.1'	0.00	-1.00	14.12	0.64	0.44	0.10	6444091	0.12	12.07	0.97
'CM002692.1'	0.00	-1.00	36.88	1.25	0.34	0.04	3622385	0.03	6.69	0.97
'CM002694.1'	0.00	-1.00	109.82	2.12	0.17	0.01	25201394	0.17	4.14	0.97
'KK214010.1'	0.17	0.14	33.11	2.28	0.31	0.13	1515066	0.00	5.47	0.98
'CM002705.1'	0.00	0.25	33.27	3.67	0.34	0.28	5535272	1.03	6.13	0.93
'CM002707.1'	0.15	0.05	144.66	3.74	0.18	0.04	46286544	9.85	1.71	0.98
'KK214768.1'	0.27	0.09	133.41	9.42	0.13	0.05	2377843	0.89	1.52	0.94
'CM002739.1'	0.00	-1.00	179.75	4.14	0.15	0.01	49703607	5.80	2.22	0.95
'CP007439.1'	0.00	0.24	23.63	2.35	0.33	0.23	5513353	0.03	9.01	0.96
'KK241008.1'	0.00	-1.00	56.94	1.52	0.27	0.03	8148774	7.91	4.81	0.97
'KK244445.1'	0.00	-1.00	101.46	1.77	0.31	0.02	33720124	24.77	2.10	0.98
'KK245170.1'	0.01	0.09	74.29	4.10	0.35	0.11	4952630	1.85	3.51	0.97
'KK249926.1'	0.32	0.12	63.66	5.71	0.15	0.06	685284	17.92	2.46	0.95
'CM002753.1'	0.05	0.18	15.63	1.02	0.60	0.28	6466847	9.25	8.49	0.99
'AZMS01S000(	0.00	-1.00	225.47	21.15	0.17	0.02	849588	32.22	1.70	0.88
'CP007569.1'	0.00	0.21	10.40	0.70	0.80	0.40	9583027	0.01	12.95	0.99
'CP007597.1'	0.27	0.28	9.38	0.82	0.53	0.34	4648976	0.05	12.26	0.98
'CM002755.1'	0.00	-1.00	10.05	0.38	0.79	0.15	3663593	0.35	13.46	0.98
'CM002757.1'	0.00	-1.00	14.22	0.85	0.31	0.09	3688450	0.38	13.42	0.95
'AXCF0100009	0.65	0.22	14.46	1.48	0.17	0.09	226476	0.00	7.91	0.97

'KK342514.1'	0.00	-1.00	328.08	26.72	0.13	0.01	1879797	28.51	1.50	0.87
'KK351785.1'	0.29	0.09	57.73	2.92	0.18	0.06	3329503	3.41	2.97	0.98
'KK351011.1'	0.60	0.07	50.80	1.60	0.08	0.02	10097680	0.50	2.64	0.98
'JHVZ0300000	0.24	0.17	22.67	2.13	0.31	0.14	582142	4.46	6.78	0.98
'CM002772.1'	0.00	-1.00	149.48	2.39	0.20	0.01	60807478	8.45	2.44	0.97
'KK502053.1'	0.30	0.05	216.50	7.32	0.10	0.02	14638873	2.98	1.02	0.97
'KK382893.1'	0.41	0.08	801.20	251.89	0.04	0.01	502267	0.31	0.57	0.76
'KK498653.1'	0.08	0.04	263.29	6.57	0.21	0.04	67462175	1.02	1.15	0.98
'KK409426.1'	0.37	0.10	246.07	45.78	0.07	0.03	379059	0.09	1.07	0.84
'KK448873.1'	0.49	0.08	593.76	154.67	0.03	0.01	437635	0.06	0.59	0.74
'KK494852.1'	0.27	0.08	377.61	75.64	0.09	0.03	429378	0.10	0.97	0.87
'JHTR0100000	0.00	-1.00	43.94	1.49	0.33	0.04	2302571	0.86	6.06	0.97
'JARK0100133	0.52	0.09	34.17	1.35	0.12	0.04	4802298	3.80	4.12	0.99
'AVPZ0100000	0.11	0.03	344.41	8.37	0.17	0.03	88624873	31.17	0.63	0.98
'KK499764.1'	0.47	0.10	45.50	2.04	0.12	0.04	5836930	0.31	3.23	0.98
'KK502400.1'	0.35	0.10	50.48	2.38	0.16	0.06	5869143	1.51	3.15	0.98
'HG916851.1'	0.00	-1.00	210.77	3.19	0.21	0.01	96939502	3.98	1.78	0.97
'HG966617.1'	0.70	0.20	11.04	0.69	0.20	0.10	3415905	0.06	8.93	0.99
'KK528063.1'	0.34	0.09	211.69	30.68	0.08	0.03	452085	0.25	1.19	0.89
'KK551537.1'	0.34	0.07	>1000	900.69	0.05	0.01	723747	0.10	0.46	0.73
'KK580071.1'	0.09	0.09	91.23	5.44	0.25	0.08	3345205	4.90	2.69	0.97
'KK570360.1'	0.36	0.09	235.54	35.26	0.07	0.03	490789	0.15	1.06	0.87
'KK599561.1'	0.44	0.10	316.04	68.73	0.05	0.02	357890	0.09	0.88	0.80
'KK617602.1'	0.55	0.08	550.08	130.54	0.02	0.01	472650	0.06	0.55	0.72
'JMRU0100000	0.22	0.23	22.96	3.55	0.31	0.18	126775	0.23	7.37	0.96
'KK652047.1'	0.36	0.08	508.14	103.96	0.05	0.02	562862	0.05	0.68	0.82
'KK667223.1'	0.52	0.08	92.30	4.36	0.06	0.02	3228359	2.23	1.63	0.96
'KK669244.1'	0.59	0.07	253.28	25.27	0.03	0.01	820821	0.16	0.70	0.87
'KK704440.1'	0.48	0.08	443.65	82.13	0.03	0.01	577101	0.23	0.61	0.79
'FO082874.1'	0.49	0.08	61.07	2.63	0.09	0.03	3568623	0.00	2.51	0.98
'KK719449.1'	0.54	0.04	267.67	6.76	0.03	0.01	25919742	1.57	0.63	0.97
'KK726493.1'	0.40	0.16	28.68	2.26	0.19	0.09	1050243	0.77	4.98	0.97
'KL671375.1'	0.49	0.04	303.34	11.02	0.04	0.01	12468512	1.74	0.60	0.95
'KK735119.1'	0.44	0.05	174.86	5.62	0.06	0.01	13337801	2.83	1.02	0.97
'AZBK01S0000	0.12	0.07	176.67	13.09	0.19	0.06	2047057	2.42	1.62	0.95
'KK757047.1'	0.40	0.09	477.91	125.74	0.05	0.02	399302	0.08	0.73	0.77
'AVPN0100000	0.41	0.13	66.33	7.80	0.11	0.04	320313	17.42	2.26	0.94
'JHQJ0100000	0.00	-1.00	88.32	4.07	0.24	0.03	3181581	1.43	3.51	0.93
'KK764649.1'	0.74	0.17	17.64	1.20	0.12	0.05	1192818	16.71	5.13	0.98
'CM002786.1'	0.00	0.06	150.87	5.25	0.21	0.06	64984695	8.22	2.38	0.97
'KK784873.1'	0.09	0.07	142.29	7.90	0.23	0.07	5927162	5.97	1.76	0.96
'KK765506.1'	0.53	0.09	62.31	3.28	0.08	0.03	2659181	35.48	1.53	0.97
'KK811377.1'	0.40	0.08	437.29	92.80	0.05	0.02	477412	0.08	0.74	0.81
'KK839031.1'	0.51	0.07	628.90	137.53	0.03	0.01	568132	0.11	0.51	0.75
'KK848034.1'	0.26	0.05	245.14	9.90	0.10	0.02	11517212	21.44	0.82	0.96
'JMSE0100000	0.46	0.18	19.04	1.70	0.20	0.09	423147	2.56	7.13	0.98
'KK852463.1'	0.00	-1.00	194.82	10.12	0.12	0.01	5111804	2.79	2.69	0.89
'KK854002.1'	0.00	-1.00	148.13	6.07	0.31	0.02	2247300	21.60	1.61	0.95
'KL142781.1'	0.31	0.05	465.32	36.66	0.06	0.02	3276566	0.83	0.62	0.91
'KK898996.1'	0.00	-1.00	250.24	11.98	0.24	0.02	1711714	2.19	1.62	0.94
'KK915662.1'	0.33	0.06	134.68	6.01	0.10	0.03	5289327	2.08	1.48	0.97
'KK998761.1'	0.44	0.09	253.11	30.87	0.05	0.02	769143	0.04	0.85	0.86
'KK949850.1'	0.48	0.06	208.49	9.87	0.05	0.01	4288987	0.28	0.86	0.95
'KK920213.1'	0.26	0.08	118.41	6.31	0.14	0.04	4410109	8.21	1.64	0.96
'KK961494.1'	0.00	-1.00	138.53	3.53	0.32	0.02	10378279	21.95	1.59	0.96
'KK948332.1'	0.55	0.09	270.34	35.05	0.03	0.01	612808	0.13	0.73	0.83
'KK960494.1'	0.49	0.10	78.75	6.60	0.08	0.03	540479	3.18	2.05	0.95
'KL142367.1'	0.00	0.19	35.03	3.18	0.46	0.27	3629680	0.94	5.40	0.96
'KL197709.1'	0.00	0.25	83.24	13.99	0.09	0.08	3433974	2.86	5.23	0.84
'KL198004.1'	0.00	-1.00	41.58	2.88	0.19	0.05	4830258	0.00	6.29	0.89
'KL198016.1'	0.00	-1.00	38.58	3.33	0.20	0.05	2162810	0.61	7.01	0.87
'KK998513.1'	0.44	0.12	86.21	8.83	0.09	0.04	504351	4.24	1.93	0.92
'KL027845.1'	0.11	0.09	290.38	52.96	0.17	0.06	540667	11.29	1.17	0.89
'KL023694.1'	0.00	-1.00	92.89	4.61	0.11	0.01	5261296	32.47	4.06	0.92
'JHEK0129195	0.66	0.16	>1000	>1000	0.02	0.01	33032	0.25	1.01	0.35
'CP007637.1'	0.21	0.20	13.77	0.95	0.47	0.25	6169071	0.02	9.47	0.98
'CP007638.1'	0.24	0.17	14.20	0.81	0.43	0.20	6085054	0.15	9.14	0.99
'KL206666.1'	0.38	0.05	158.07	4.72	0.08	0.02	19375980	2.16	1.24	0.97
'KL215552.1'	0.37	0.05	372.16	16.88	0.05	0.01	8694517	0.74	0.61	0.94
'KL218800.1'	0.28	0.09	57.73	2.79	0.18	0.06	4602521	22.86	2.50	0.98
'KL221432.1'	0.46	0.06	112.82	4.45	0.07	0.02	5804300	4.03	1.45	0.97
'KL217905.1'	0.49	0.04	458.53	13.27	0.03	0.01	23311805	2.41	0.39	0.95
'KL225530.1'	0.40	0.05	176.45	4.78	0.07	0.01	22859560	3.50	1.04	0.98
'KL226002.1'	0.34	0.04	177.77	4.57	0.09	0.02	28260285	4.25	1.13	0.98
'KL230200.1'	0.43	0.09	319.67	59.18	0.05	0.02	440645	0.08	0.85	0.82
'KL250487.1'	0.42	0.09	84.28	5.41	0.10	0.03	1826302	5.15	1.94	0.96
'KL274534.1'	0.08	0.11	245.07	58.66	0.19	0.08	263953	0.24	1.71	0.89
'KL314543.1'	0.28	0.10	226.72	39.18	0.10	0.04	384696	0.27	1.28	0.88
'CM002797.1'	0.00	-1.00	105.04	1.95	0.24	0.01	12740948	2.29	3.60	0.98
'KL361834.1'	0.44	0.08	504.11	108.83	0.04	0.01	485829	0.17	0.64	0.80
'JNCD0100605	0.00	0.11	252.57	51.08	0.25	0.10	580801	17.13	1.44	0.90
'JNCE0129995	0.10	0.09	197.23	24.90	0.20	0.07	811454	3.95	1.61	0.92
'KL363182.1'	0.55	0.13	28.87	1.71	0.13	0.05	1594463	1.78	4.45	0.98
'KL367474.1'	0.57	0.13	25.27	1.40	0.13	0.05	1448326	1.53	5.01	0.98
'KL371215.1'	0.39	0.09	269.32	42.73	0.06	0.02	510503	0.05	0.95	0.85
'KL886562.1'	0.39	0.06	272.94	19.29	0.06	0.02	1986275	0.98	0.80	0.93
'KL871737.1'	0.36	0.04	178.42	4.80	0.08	0.02	21921334	1.60	1.13	0.98
'KL411542.1'	0.94	0.16	10.98	0.55	0.12	0.04	2325249	0.47	8.17	0.99
'KL411536.1'	0.38	0.04	213.45	5.61	0.07	0.01	27015053	1.63	0.92	0.98
'DF236950.1'	0.16	0.16	30.49	2.92	0.32	0.14	541865	1.68	5.96	0.97
'KL430423.1'	0.33	0.09	368.58	69.91	0.07	0.02	506573	0.31	0.85	0.84
'KL446944.1'	0.66	0.07	138.69	7.41	0.03	0.01	2470179	1.11	0.99	0.94
'AMGV010000(	0.70	0.10	21.18	0.75	0.11	0.03	5365614	0.19	5.38	0.99
'KL448103.1'	0.62	0.05	179.87	5.20	0.03	0.01	14000334	1.30	0.80	0.97
'KL449087.1'	0.49	0.08	277.12	37.39	0.04	0.01	643976	0.16	0.78	0.85
'CP006581.1'	0.00	-1.00	11.02	0.34	0.76	0.13	6364365	0.07	12.35	0.99
'KL487749.1'	0.37	0.08	378.62	67.26	0.06	0.02	549738	0.14	0.79	0.84
'CM002798.1'	0.00	-1.00	38.47	0.86	0.46	0.04	13597520	0.00	5.24	0.98
'KL509103.1'	0.43	0.10	136.31	14.57	0.07	0.03	461566	0.97	1.43	0.91
'AZAH0100000	0.72	0.10	37.30	1.75	0.06	0.02	1827054	0.34	3.20	0.98
'CM002803.1'	0.35	0.10	46.88	2.20	0.17	0.05	4785602	0.02	3.54	0.98
'CM002809.1'	0.00	-1.00	102.38	1.77	0.21	0.01	22465362	0.26	4.00	0.98
'CM002811.2'	0.00	-1.00	105.08	1.78	0.21	0.01	19424428	2.54	3.86	0.98
'CM002810.1'	0.00	-1.00	104.25	1.95	0.27	0.02	14440416	1.35	3.45	0.98
'CM002812.1'	0.00	-1.00	200.50	2.33	0.30	0.01	301019445	3.97	1.32	0.98
'JJRZ0100000	0.48	0.18	31.41	3.46	0.14	0.07	283345	0.84	4.56	0.95

'JMSH0100000	0.55	0.14	26.69	1.84	0.13	0.05	807249	0.04	5.00	0.98
'CP008749.1'	0.00	-1.00	12.24	0.52	0.59	0.13	6264404	2.35	11.96	0.97
'AWOJ01S027(	0.20	0.12	83.30	9.99	0.19	0.08	369949	0.18	2.78	0.94
'AWOK01S000	0.30	0.13	74.17	8.76	0.14	0.06	408837	4.39	2.58	0.93
'AWOL01S000	0.00	0.11	105.59	10.72	0.32	0.13	1052670	4.92	2.61	0.95
'AYMY01S002	0.00	-1.00	126.35	8.39	0.27	0.03	589094	1.40	2.73	0.92
'KL567389.1'	0.38	0.14	89.65	13.62	0.10	0.04	202915	0.03	2.24	0.90
'JMSD0100000	0.39	0.08	386.18	55.92	0.05	0.02	793585	0.41	0.69	0.84
'KL596619.1'	0.76	0.09	27.83	0.85	0.08	0.02	9657489	6.00	3.74	0.99
'DF384229.1'	0.00	-1.00	129.70	8.76	0.29	0.03	831256	31.94	1.76	0.92
'KL580752.1'	0.31	0.12	57.50	4.97	0.16	0.06	668473	6.88	3.14	0.96
'CM002825.1'	0.00	-1.00	111.66	2.45	0.15	0.01	26188458	0.34	4.37	0.97
'KL584749.1'	0.46	0.16	22.95	1.47	0.19	0.09	2141954	0.01	5.80	0.98
'KL584702.1'	0.60	0.13	20.59	1.08	0.14	0.05	2484475	0.01	5.85	0.98
'KL584824.1'	0.61	0.14	22.46	1.18	0.13	0.05	2719209	0.00	5.39	0.98
'KL584974.1'	0.24	0.16	27.06	1.87	0.30	0.14	2687588	0.00	5.67	0.97
'CM002832.3'	0.09	0.05	162.42	4.55	0.23	0.05	46052510	1.18	1.70	0.98
'KN671115.1'	0.32	0.06	174.27	7.42	0.10	0.02	7596472	1.56	1.23	0.96
'HG315671.1'	0.46	0.10	52.84	2.62	0.11	0.04	4228350	0.56	2.80	0.97
'CBTN0100000	0.72	0.12	33.42	1.95	0.07	0.03	1313670	6.86	3.34	0.97
'HG810775.1'	0.52	0.06	275.07	16.80	0.04	0.01	3208305	0.40	0.63	0.91
'HG820002.1'	0.00	0.12	83.83	10.22	0.29	0.12	599721	10.17	3.55	0.95
'CM003871.1'	0.00	-1.00	112.28	1.82	0.18	0.01	37867402	0.04	4.10	0.98
'CM002865.1'	0.00	0.11	82.55	6.26	0.33	0.14	3498471	0.50	3.19	0.96
'KL647288.1'	0.00	-1.00	37.17	1.44	0.29	0.05	11360811	0.82	6.17	0.95
'JHUM0100003	0.39	0.23	16.87	1.93	0.26	0.14	265719	0.02	8.21	0.97
'CM002866.1'	0.00	-1.00	11.79	0.42	0.72	0.13	5370749	0.02	11.92	0.98
'KL648097.1'	0.29	0.17	21.61	1.74	0.28	0.13	861210	0.16	7.04	0.98
'JHOI01000634	0.25	0.18	29.60	3.02	0.27	0.13	470300	0.23	5.66	0.96
'JOWA0100011	0.00	-1.00	32.56	1.23	0.43	0.06	3171699	0.00	6.22	0.96
'JPET0100000	0.00	-1.00	34.06	1.63	0.20	0.04	9084368	0.07	7.84	0.94
'CP007023.1'	0.58	0.11	53.09	3.17	0.08	0.03	1709442	0.02	2.61	0.96
'KL652500.1'	0.27	0.06	165.57	8.72	0.11	0.03	4516015	14.51	1.23	0.96
'KL650235.1'	0.49	0.13	21.28	1.31	0.18	0.06	762200	0.12	6.41	0.99
'KL657812.1'	0.67	0.16	17.49	1.27	0.14	0.06	433445	0.07	6.60	0.98
'KL659601.1'	0.39	0.17	21.12	1.87	0.23	0.10	483811	0.15	6.84	0.98
'CM002876.1'	0.00	0.06	116.67	3.94	0.30	0.08	40886855	8.13	2.37	0.98
'KL662111.1'	0.07	0.16	27.39	2.46	0.40	0.17	1144356	5.41	7.19	0.98
'CM002964.1'	0.78	0.10	39.98	2.04	0.05	0.02	1434165	0.03	2.86	0.97
'JNVV0100000	0.05	0.14	22.16	1.28	0.49	0.19	4491949	0.66	8.01	0.99
'JPFM0100000	0.64	0.07	297.60	26.45	0.02	0.01	1303432	0.22	0.57	0.86
'JPFL01000001	0.52	0.06	968.74	193.68	0.02	0.01	1048449	0.47	0.36	0.74
'CM002954.1'	0.78	0.10	40.23	2.05	0.05	0.02	1496914	0.07	2.85	0.97
'KL668297.1'	0.25	0.05	110.28	2.93	0.15	0.03	28796675	1.40	2.07	0.99
'CP008959.1'	0.42	0.20	8.26	0.50	0.43	0.20	4539435	3.74	12.20	0.99
'CP008960.1'	0.41	0.20	8.32	0.50	0.44	0.20	4539435	4.63	12.04	0.99
'CP008961.1'	0.41	0.20	8.31	0.50	0.44	0.20	4539435	4.50	12.06	0.99
'CP008962.1'	0.43	0.21	8.23	0.51	0.43	0.20	4539435	5.68	11.90	0.99
'CP008963.1'	0.46	0.21	8.14	0.49	0.41	0.19	4539435	5.50	11.92	0.99
'CP008964.1'	0.42	0.22	8.29	0.53	0.44	0.21	4539435	5.68	11.90	0.99
'CP008965.1'	0.42	0.21	8.31	0.52	0.44	0.20	4539435	6.25	11.81	0.99
'CP008966.1'	0.43	0.21	8.25	0.51	0.43	0.20	4539435	6.20	11.83	0.99
'CP008967.1'	0.40	0.21	8.35	0.54	0.45	0.21	4539435	7.17	11.74	0.99
'CP008968.1'	0.51	0.19	7.89	0.43	0.38	0.16	4539435	5.74	11.95	0.99
'CP008969.1'	0.42	0.21	8.31	0.52	0.43	0.20	4539435	8.61	11.48	0.99
'CP008970.1'	0.40	0.20	8.35	0.51	0.45	0.20	4539435	5.21	11.97	0.99
'CP008971.1'	0.63	0.21	7.46	0.43	0.32	0.14	4539433	12.15	11.14	0.99
'CP008972.1'	0.41	0.21	8.33	0.52	0.44	0.21	4539433	6.06	11.87	0.99
'CP008973.1'	0.41	0.21	8.35	0.53	0.44	0.21	4539435	5.92	11.85	0.99
'CP008974.1'	0.39	0.21	8.37	0.53	0.45	0.21	4539435	5.88	11.87	0.99
'CP008975.1'	0.41	0.22	8.29	0.54	0.44	0.21	4539435	4.73	12.04	0.99
'CP008976.1'	0.39	0.20	8.36	0.50	0.45	0.20	4539435	4.76	12.04	0.99
'CP008977.1'	0.40	0.20	8.33	0.51	0.45	0.20	4539435	4.58	12.06	0.99
'CP008978.1'	0.38	0.22	8.40	0.56	0.46	0.22	4539435	4.53	12.08	0.99
'CP008979.1'	0.42	0.21	8.32	0.51	0.44	0.20	4539435	7.96	11.58	0.99
'CP008980.1'	0.35	0.21	8.52	0.56	0.48	0.23	4539435	4.84	12.03	0.99
'CP008981.1'	0.40	0.20	8.33	0.51	0.45	0.20	4539435	4.28	12.10	0.99
'CP008982.1'	0.40	0.20	8.33	0.51	0.45	0.20	4539435	4.28	12.10	0.99
'CP008983.1'	0.41	0.21	8.35	0.53	0.44	0.21	4539433	8.34	11.54	0.99
'CP008947.1'	0.00	-1.00	9.94	0.46	0.54	0.14	8466345	0.20	14.36	0.97
'KL772050.1'	0.17	0.11	576.24	325.58	0.12	0.05	189902	0.11	1.22	0.81
'KL808647.1'	0.26	0.12	344.95	142.35	0.10	0.04	130069	0.39	1.37	0.82
'JPMF0100376	0.00	-1.00	171.32	24.52	0.24	0.03	133928	0.05	2.83	0.89
'KL869011.1'	0.25	0.04	229.07	6.04	0.11	0.02	30461450	0.61	1.10	0.98
'KL996993.1'	0.53	0.10	74.88	5.27	0.07	0.02	1104412	3.72	1.99	0.95
'KL995831.1'	0.60	0.11	72.00	5.82	0.06	0.02	583253	3.71	1.94	0.94
'KL995122.1'	0.52	0.10	77.88	5.80	0.07	0.02	589952	4.17	1.95	0.95
'JEMP0100002	0.39	0.14	27.29	1.75	0.20	0.08	1704168	0.36	5.24	0.98
'KL997524.1'	0.52	0.04	274.30	6.10	0.03	0.01	50248042	1.14	0.63	0.97
'JDSR0100032	0.84	0.25	12.80	1.43	0.12	0.07	129862	0.00	7.63	0.97
'AKIJ01000001	0.23	0.10	114.34	11.09	0.16	0.06	927658	1.15	1.93	0.93
'KN005617.1'	0.00	-1.00	147.43	6.87	0.26	0.02	1317973	6.57	2.24	0.94
'KN042416.1'	0.36	0.11	31.67	1.47	0.20	0.07	5074928	2.26	4.70	0.98
'CP007756.1'	0.00	-1.00	33.51	1.41	0.40	0.06	2441780	0.00	6.15	0.96
'CP007789.1'	0.00	-1.00	16.24	0.80	0.57	0.12	2127512	0.00	9.83	0.97
'AP011946.1'	0.00	-1.00	79.64	2.94	0.26	0.03	2746313	0.00	4.07	0.96
'CP007707.1'	0.00	-1.00	16.30	0.87	0.56	0.13	2125890	0.09	9.83	0.96
'CM003510.1'	0.11	0.04	185.34	4.63	0.21	0.04	55601358	5.54	1.43	0.98
'CM002879.1'	0.35	0.10	51.43	2.43	0.16	0.05	5189487	0.03	3.21	0.98
'CP007640.1'	0.00	0.20	69.49	8.26	0.16	0.11	4175060	0.01	4.98	0.90
'CP008752.1'	0.33	0.10	51.89	2.54	0.17	0.06	5229650	0.06	3.21	0.98
'CP007704.1'	0.33	0.10	51.88	2.56	0.17	0.06	5230053	0.04	3.22	0.98
'KN046829.1'	0.38	0.06	102.57	4.41	0.10	0.02	3825275	3.55	1.78	0.97
'CP008838.1'	0.00	-1.00	11.43	0.43	0.77	0.15	4989312	0.00	11.72	0.98
'KN049970.1'	0.68	0.14	16.17	0.96	0.15	0.05	596216	0.01	7.01	0.99
'KN050433.1'	0.02	0.17	31.10	2.66	0.48	0.25	1994917	1.30	5.91	0.97
'KN122054.1'	0.19	0.04	283.33	9.42	0.14	0.03	22231620	1.80	0.91	0.97
'JPUL0200000	0.84	0.15	41.88	4.76	0.04	0.02	133271	0.00	2.74	0.92
'CM002881.1'	0.00	-1.00	10.81	0.39	0.68	0.13	5142886	0.19	13.50	0.98
'CM002882.1'	0.34	0.22	8.53	0.58	0.49	0.24	4410946	0.00	12.73	0.99
'DF830068.1'	0.49	0.17	11.65	0.69	0.28	0.11	2397122	0.20	10.06	0.99
'KN126570.1'	0.53	0.04	326.69	9.54	0.03	0.01	19535321	2.36	0.51	0.96
'JPHH0300000	0.12	0.20	19.08	1.61	0.45	0.24	1584712	0.00	8.45	0.98
'JPOX0100000	0.44	0.14	28.25	1.85	0.17	0.07	1284304	0.03	5.05	0.98
'CP008955.1'	0.00	-1.00	38.77	1.32	0.38	0.05	4444328	0.05	5.53	0.97
'HG974234.1'	0.00	-1.00	14.83	0.67	0.40	0.09	6762448	1.16	11.83	0.97

'LK031787.1'	0.00	-1.00	135.82	3.71	0.31	0.02	5197798	7.12	2.09	0.96
'CCEW0100000	0.53	0.27	15.43	2.20	0.21	0.13	175422	0.00	7.97	0.96
'CCOE0100028	0.39	0.05	175.90	6.67	0.07	0.02	8593483	2.49	1.11	0.97
'CM002883.1'	0.35	0.22	8.52	0.57	0.49	0.24	4410169	1.06	12.54	0.99
'CCMK0100160	0.00	-1.00	529.76	173.65	0.22	0.02	151776	0.71	1.67	0.83
'CM002884.1'	0.35	0.22	8.52	0.58	0.49	0.24	4410911	0.75	12.59	0.99
'CCDZ0100000	0.41	0.13	>1000	>1000	0.06	0.02	40762	2.07	1.22	0.68
'JQCY0200000	0.06	0.12	65.17	6.29	0.30	0.11	641448	3.96	3.76	0.96
'KN127243.1'	0.20	0.13	187.35	45.46	0.14	0.06	485353	58.91	0.72	0.86
'CM002913.1'	0.04	0.07	53.27	1.77	0.36	0.09	27160941	0.01	4.37	0.99
'KN265167.1'	0.30	0.04	320.76	7.75	0.08	0.01	43721425	1.81	0.72	0.97
'CP009098.1'	0.00	-1.00	19.17	1.16	0.37	0.09	2188974	0.05	9.79	0.94
'CP009404.1'	0.41	0.15	18.48	1.10	0.24	0.10	2610169	1.30	7.30	0.98
'CP008764.1'	0.00	-1.00	10.72	0.58	0.34	0.09	4099163	0.00	17.70	0.96
'CCSE0100000	0.00	-1.00	44.75	1.94	0.30	0.04	2191063	0.00	5.73	0.95
'CCSF0100000	0.43	0.20	11.50	0.75	0.33	0.17	3673759	0.00	9.74	0.99
'KN151326.1'	0.18	0.14	30.23	2.15	0.31	0.13	1694367	1.00	5.93	0.98
'KN151899.1'	0.40	0.08	89.32	4.71	0.10	0.03	2254159	6.85	1.88	0.97
'KN165825.1'	0.76	0.13	18.75	0.98	0.10	0.04	1446875	0.36	5.82	0.99
'KN167705.1'	0.29	0.17	24.65	2.04	0.27	0.12	1130325	0.38	6.34	0.97
'KN172139.1'	0.50	0.16	28.16	2.69	0.14	0.06	333535	1.30	4.96	0.96
'KN173723.1'	0.29	0.21	20.38	2.07	0.29	0.16	873274	2.77	7.31	0.97
'KN196491.1'	0.12	0.08	102.57	5.00	0.24	0.07	7469083	9.78	2.08	0.97
'CM002918.1'	0.25	0.14	29.11	1.78	0.27	0.11	3364521	0.12	5.50	0.98
'CP008916.1'	0.00	-1.00	10.32	0.50	0.38	0.09	4125094	0.00	17.66	0.97
'KN214215.1'	0.17	0.10	300.65	67.59	0.14	0.05	429344	0.37	1.24	0.86
'JRHR0100367	0.14	0.24	15.76	2.20	0.45	0.24	148023	0.00	10.30	0.97
'JQDR0200000	0.80	0.20	11.33	0.73	0.16	0.08	2207822	0.00	8.56	0.98
'JQFK0100000	0.75	0.12	49.83	3.75	0.05	0.02	607893	0.34	2.42	0.95
'CCBP0100001	0.33	0.18	17.00	1.35	0.31	0.15	899948	1.01	8.02	0.98
'HG918267.1'	0.32	0.05	225.18	8.18	0.09	0.02	11999608	2.88	0.95	0.96
'HG924883.1'	0.00	-1.00	182.23	3.42	0.27	0.01	14724226	3.40	1.86	0.97
'CP009578.1'	0.00	-1.00	30.46	1.17	0.32	0.05	4874804	0.00	7.41	0.96
'JRIB01000569	0.81	0.17	40.12	4.91	0.05	0.02	142656	0.14	2.88	0.92
'JRIC01000262	0.63	0.17	47.75	6.37	0.07	0.03	170627	0.16	2.87	0.91
'JRIE01000200	0.74	0.15	42.38	4.27	0.05	0.02	216801	0.09	2.81	0.93
'JRID01000075	0.71	0.16	45.36	5.43	0.06	0.03	143401	0.17	2.78	0.92
'JRIF01000017	0.79	0.12	40.71	2.87	0.05	0.02	406493	0.25	2.83	0.96
'JRIH01000084	0.82	0.13	38.62	3.04	0.05	0.02	326823	0.18	2.89	0.95
'JRIG01000095	0.69	0.17	44.61	5.91	0.06	0.03	134064	0.00	2.90	0.91
'JRIL01000146	0.61	0.14	45.88	4.35	0.08	0.03	319971	0.49	2.97	0.95
'JRII01000088.	0.78	0.17	40.68	5.11	0.05	0.02	135217	0.01	2.92	0.92
'JRIO01000036	0.65	0.13	44.69	3.73	0.07	0.03	462932	0.42	2.89	0.95
'JRIN01000192	0.74	0.13	41.07	3.11	0.06	0.02	454382	0.25	2.92	0.96
'JRIQ01000037	0.78	0.11	41.39	2.78	0.05	0.02	458709	0.22	2.84	0.96
'JRIR01000143	0.77	0.12	41.78	3.18	0.05	0.02	341843	0.22	2.83	0.96
'JRIZ01000011	0.76	0.14	40.51	3.51	0.05	0.02	320854	0.08	2.91	0.95
'JRIU01000133	0.72	0.14	42.00	3.83	0.06	0.03	336272	0.15	2.91	0.94
'JRIV01000074	0.77	0.16	40.15	3.90	0.05	0.02	334215	0.20	2.85	0.93
'JRIT01000140	0.75	0.13	40.59	2.95	0.05	0.02	580633	0.05	2.91	0.96
'JRIX01000074	0.68	0.14	44.08	4.31	0.06	0.03	298290	0.41	2.92	0.94
'JRIY01000123	0.64	0.14	45.09	4.49	0.07	0.03	343062	0.14	2.93	0.94
'JRIS01000158	0.74	0.13	41.07	3.10	0.06	0.02	454112	0.12	2.92	0.96
'JRIW01000103	0.63	0.14	44.60	4.06	0.07	0.03	389964	0.20	2.94	0.94
'JRIK01000357	0.78	0.17	40.55	4.99	0.05	0.02	150051	0.06	2.89	0.92
'JRIP01000004	0.76	0.13	40.26	3.02	0.05	0.02	540496	0.24	2.93	0.96
'KN265652.1'	0.22	0.04	231.86	6.05	0.12	0.02	30727332	0.85	1.16	0.98
'KN880357.1'	0.00	-1.00	138.12	19.36	0.04	0.01	3473262	0.07	5.57	0.73
'JNBV0100000	0.00	-1.00	65.65	5.78	0.12	0.03	2218769	0.71	6.05	0.85
'KN266224.1'	0.51	0.12	65.26	5.44	0.08	0.03	778042	5.61	2.24	0.94
'CP007555.1'	0.31	0.13	27.58	1.70	0.24	0.10	2062778	3.21	5.48	0.98
'FN827340.1'	0.00	-1.00	213.37	6.61	0.27	0.02	4320882	0.53	1.66	0.96
'KN268387.1'	0.18	0.04	373.35	12.50	0.13	0.03	27394153	0.58	0.73	0.96
'KN271213.1'	0.40	0.04	358.09	10.59	0.05	0.01	23736781	0.39	0.55	0.96
'KN277429.1'	0.18	0.04	315.37	8.48	0.14	0.03	46538883	0.42	0.85	0.97
'JRIJ01000167	0.75	0.15	41.79	4.43	0.05	0.02	244353	0.03	2.89	0.93
'JRIM01000215	0.63	0.20	55.46	12.10	0.06	0.03	57541	0.06	2.75	0.85
'KN284664.1'	0.17	0.07	190.53	14.73	0.16	0.05	2116932	1.51	1.41	0.94
'JROU0200046	0.70	0.16	23.82	1.90	0.10	0.04	330455	0.02	4.85	0.97
'KN296311.1'	0.15	0.06	180.62	7.51	0.17	0.04	9848887	1.34	1.49	0.97
'JPYM0100000	0.30	0.13	31.28	1.83	0.23	0.10	4337333	0.00	5.03	0.98
'CM002938.1'	0.74	0.10	41.17	2.12	0.06	0.02	1455881	0.00	2.85	0.97
'JPKY0100000	0.39	0.16	19.20	1.41	0.24	0.10	878651	0.00	7.46	0.98
'JQGE0100001	0.00	-1.00	66.63	4.55	0.14	0.03	6460479	0.00	4.96	0.87
'JQGD0100002	0.00	-1.00	76.48	5.76	0.13	0.03	4904830	0.00	4.80	0.86
'CP009684.1'	0.00	-1.00	38.18	1.59	0.27	0.04	3941446	0.63	6.62	0.96
'CP009656.1'	0.71	0.09	139.77	10.86	0.02	0.01	910687	0.00	0.98	0.90
'CP009270.1'	0.00	-1.00	9.62	0.50	0.42	0.11	3945449	0.00	17.97	0.97
'CP009271.1'	0.00	-1.00	9.37	0.41	0.46	0.11	4032226	0.00	17.88	0.98
'CP009153.1'	0.00	-1.00	9.87	0.52	0.39	0.10	3968347	0.01	17.97	0.97
'CM002998.1'	0.26	0.03	265.60	4.35	0.10	0.02	215897965	22.44	0.70	0.99
'KN305596.1'	0.00	-1.00	114.90	23.26	0.04	0.01	2015588	0.31	7.73	0.70
'CM002977.3'	0.16	0.03	266.52	4.41	0.15	0.02	225584828	2.42	1.06	0.99
'AJIX01000003	0.57	0.08	93.04	5.63	0.05	0.02	1321555	0.47	1.61	0.95
'AJIW01000012	0.48	0.09	96.84	6.53	0.07	0.03	1474047	1.81	1.63	0.94
'AJIU01000001	0.53	0.09	93.30	5.79	0.06	0.02	1697563	1.94	1.63	0.95
'AJIS01000033	0.50	0.08	98.67	5.52	0.07	0.02	1988385	3.08	1.57	0.96
'AJIY01000001	0.52	0.08	94.71	5.30	0.06	0.02	1651406	0.43	1.67	0.96
'AJJA01000002	0.51	0.08	96.22	5.67	0.07	0.02	1692349	0.83	1.64	0.95
'AJIQ01000005	0.48	0.09	100.14	7.01	0.07	0.03	1445339	2.84	1.60	0.94
'AJIT01000001	0.49	0.09	97.16	6.23	0.07	0.02	1680193	0.32	1.65	0.94
'JRBH0100051	0.67	0.21	>1000	>1000	0.03	0.02	11528	0.00	1.51	0.33
'CM003019.1'	0.00	-1.00	10.14	0.48	0.39	0.09	3837469	0.08	17.76	0.97
'JPPO0200000	0.57	0.11	125.41	12.05	0.04	0.02	1362551	0.00	1.26	0.88
'JPYR0100000	0.29	0.15	37.84	3.51	0.21	0.10	622763	0.66	4.51	0.96
'JOTO0120020	0.41	0.19	43.10	7.31	0.14	0.07	80531	0.21	3.88	0.92
'KN323322.1'	0.31	0.08	363.09	52.57	0.07	0.02	869281	1.18	0.80	0.87
'AJIV01000007	0.50	0.09	96.12	6.31	0.07	0.02	1511217	0.82	1.66	0.95
'AJIR01000008	0.43	0.09	105.90	7.56	0.08	0.03	1600240	0.81	1.62	0.94
'AJIZ01000001	0.52	0.09	95.54	5.89	0.06	0.02	1620026	0.42	1.66	0.95
'AJJB01000009	0.52	0.08	97.11	5.42	0.06	0.02	1830800	2.13	1.58	0.96
'AJJC01000012	0.49	0.08	99.97	5.89	0.07	0.02	1945554	1.35	1.59	0.95
'JSUU0100225	0.24	0.09	239.14	32.17	0.11	0.04	750149	6.50	1.12	0.90
'AJJE01000008	0.49	0.09	96.18	5.59	0.07	0.02	2207883	2.57	1.63	0.95
'AJJD01000004	0.49	0.08	96.29	5.02	0.07	0.02	3195629	0.69	1.65	0.96
'JSXQ0100000	0.50	0.09	97.02	5.91	0.07	0.02	1681656	0.21	1.65	0.95

'JSXP0100000	0.51	0.08	95.75	5.66	0.06	0.02	1704923	1.25	1.64	0.95
'JSXR0100001	0.54	0.08	93.75	5.14	0.06	0.02	2001971	6.20	1.53	0.96
'JPEV0100001	0.49	0.09	99.56	6.12	0.07	0.02	1839929	2.48	1.58	0.95
'JPEW0100000	0.48	0.09	97.91	6.46	0.07	0.02	1500832	1.12	1.65	0.95
'AJJF01000001	0.49	0.08	95.92	5.05	0.07	0.02	3211439	0.77	1.65	0.96
'JTBX0100002	0.42	0.09	105.60	7.37	0.09	0.03	1580593	0.99	1.64	0.94
'AJJG0100001	0.50	0.09	99.73	6.77	0.07	0.02	1421257	0.65	1.60	0.94
'CP009909.1'	0.08	0.12	37.67	2.20	0.38	0.15	5762608	0.01	4.88	0.98
'KN360965.1'	0.14	0.13	>1000	>1000	0.13	0.06	72118	1.44	1.40	0.76
'JPWU0200000	0.60	0.16	20.24	1.56	0.15	0.06	440385	0.00	6.10	0.98
'JPWV0200000	0.59	0.16	20.33	1.55	0.15	0.06	440501	0.00	6.09	0.98
'JTEQ0100032	0.64	0.27	64.73	32.40	0.06	0.04	14434	0.00	3.10	0.73
'KN707963.1'	0.26	0.26	172.13	120.44	0.10	0.08	62955	0.00	2.42	0.67
'LN625285.1'	0.68	0.19	24.83	2.56	0.10	0.05	242052	0.00	4.84	0.96
'HG428765.1'	0.59	0.10	70.06	5.00	0.06	0.02	756357	5.12	1.97	0.95
'KN389625.1'	0.49	0.13	23.85	1.40	0.16	0.06	1058811	0.00	5.78	0.98
'KN476293.1'	0.18	0.11	166.65	25.57	0.16	0.06	429644	0.45	1.75	0.91
'KN462367.1'	0.26	0.07	157.53	8.60	0.12	0.03	3397572	0.37	1.49	0.96
'KN493925.1'	0.04	0.11	329.39	108.90	0.21	0.09	222328	0.38	1.60	0.87
'DF837573.1'	0.00	-1.00	98.53	3.51	0.29	0.02	1840102	0.04	3.21	0.96
'DF357216.1'	0.26	0.10	160.52	19.01	0.12	0.04	629958	1.91	1.52	0.91
'KN398398.1'	0.03	0.12	272.05	91.14	0.22	0.09	231483	16.67	1.59	0.87
'KN521806.1'	0.27	0.05	162.15	5.05	0.11	0.02	17765049	2.09	1.43	0.98
'JTKG0100000	0.35	0.08	97.98	5.36	0.11	0.03	2147216	4.47	1.87	0.96
'KN538681.1'	0.00	-1.00	121.12	5.73	0.22	0.02	1127032	4.80	3.38	0.95
'JFBF0100000	0.29	0.08	53.11	2.12	0.18	0.05	6840757	14.18	2.96	0.98
'KN538375.1'	0.56	0.11	27.62	1.18	0.13	0.04	4309324	0.61	4.71	0.99
'KN549200.1'	0.70	0.11	31.85	1.64	0.08	0.03	1554998	12.06	3.36	0.98
'JNVN0100000	0.40	0.13	104.46	16.76	0.09	0.04	188576	0.08	1.96	0.89
'JNUS0100000	0.39	0.13	106.17	17.59	0.09	0.04	188575	0.23	1.95	0.89
'JNUT0100002	0.40	0.13	106.42	17.93	0.09	0.04	188770	0.01	1.96	0.89
'JNUU0100003	0.41	0.14	117.07	24.94	0.08	0.04	116670	0.01	1.94	0.87
'KN613455.1'	0.07	0.08	137.97	10.21	0.25	0.08	2584131	3.57	2.02	0.95
'JUJX0100000	0.00	-1.00	104.59	17.69	0.08	0.02	1124325	0.09	5.69	0.76
'JRUI02000006	0.00	-1.00	148.93	2.31	0.25	0.01	53636577	13.03	2.03	0.98
'JPDR0100000	0.43	0.14	27.60	1.99	0.18	0.07	663571	0.04	5.33	0.98
'JWIN0103414	0.00	-1.00	216.63	5.17	0.26	0.01	9719801	1.35	1.61	0.96
'JPKZ0102284	0.66	0.12	27.75	1.45	0.10	0.04	1924903	4.94	4.10	0.98
'AZHE0100000	0.00	-1.00	88.83	10.89	0.04	0.01	3946332	0.17	6.90	0.76
'JRXJ0100001	0.29	0.09	202.13	20.24	0.10	0.03	1029232	0.01	1.16	0.91
'KN709239.1'	0.00	-1.00	236.03	7.89	0.25	0.02	4484195	1.25	1.57	0.95
'KN711236.1'	0.14	0.13	30.04	1.78	0.34	0.14	3511474	0.59	6.06	0.98
'KN711580.1'	0.00	0.10	92.00	5.27	0.27	0.11	12740554	4.54	3.07	0.96
'KN713669.1'	0.58	0.09	35.86	1.35	0.10	0.03	5503185	3.14	3.63	0.99
'JRUC0100000	0.25	0.21	23.81	3.45	0.28	0.15	119371	0.00	7.08	0.96
'CM003052.1'	0.00	-1.00	12.63	0.61	0.46	0.11	4093383	0.05	13.57	0.97
'CP005977.1'	0.52	0.15	23.06	1.39	0.16	0.07	2503991	0.34	5.48	0.98
'CP010342.1'	0.34	0.10	51.59	2.42	0.16	0.05	5227435	0.58	3.20	0.98
'KN714663.1'	0.56	0.09	37.09	1.51	0.10	0.03	3762872	0.24	3.82	0.99
'JUDQ0100000	0.46	0.17	24.35	2.38	0.18	0.08	380183	0.69	5.78	0.97
'JOMC0100002	0.38	0.15	24.47	1.75	0.22	0.09	1033815	0.01	5.98	0.98
'JWJA0100451	0.22	0.22	64.33	19.23	0.19	0.12	56381	0.00	3.77	0.87
'CP007548.1'	0.81	0.15	13.63	0.74	0.12	0.05	1137653	0.02	7.51	0.99
'KN714666.1'	0.45	0.13	25.26	1.53	0.18	0.06	1056303	1.89	5.61	0.98
'JSSU0100140	0.45	0.16	34.15	3.56	0.14	0.06	236255	0.17	4.45	0.96
'AZNE0100000	0.50	0.13	22.25	1.21	0.17	0.06	2024915	0.08	5.89	0.98
'AZNH0100000	0.26	0.16	25.72	1.79	0.29	0.14	2695598	0.01	5.92	0.97
'AZNG0100000	0.44	0.11	22.99	0.92	0.20	0.06	7151295	0.04	6.04	0.99
'AZNF0100000	0.00	-1.00	49.02	2.79	0.18	0.04	5411467	0.05	5.93	0.91
'KN715323.1'	0.51	0.25	19.33	2.63	0.18	0.11	168809	0.01	6.74	0.95
'KN715895.1'	0.33	0.12	29.93	1.58	0.22	0.08	2721568	0.22	5.26	0.98
'KN716150.1'	0.59	0.10	49.10	2.52	0.08	0.03	2204563	1.61	2.80	0.97
'KN726132.1'	0.58	0.14	34.26	2.93	0.10	0.04	282465	2.00	3.95	0.96
'CP006902.1'	0.38	0.06	350.58	22.60	0.05	0.01	4077214	0.00	0.64	0.92
'CBXB0100000	0.15	0.11	130.90	15.44	0.19	0.07	618193	1.09	2.00	0.93
'HG417165.1'	0.00	-1.00	111.73	1.60	0.20	0.01	39866532	1.31	3.90	0.98
'CM003064.1'	0.00	-1.00	112.33	1.67	0.19	0.01	39671193	3.99	3.82	0.98
'CM003076.1'	0.00	-1.00	111.55	1.63	0.20	0.01	38770143	3.64	3.85	0.98
'CM003098.1'	0.12	0.13	33.08	2.02	0.36	0.15	5395810	14.23	4.80	0.98
'JTFG0200000	0.00	-1.00	323.99	58.38	0.19	0.02	207014	0.00	2.31	0.87
'KN796172.1'	0.46	0.10	47.90	2.42	0.12	0.04	3656114	5.89	2.97	0.97
'CM003111.1'	0.00	-1.00	30.99	1.14	0.49	0.07	2962552	3.21	5.78	0.97
'JROL0100704	0.00	0.18	173.21	106.00	0.24	0.13	40257	3.30	3.58	0.88
'KN805369.1'	0.67	0.07	51.35	1.61	0.06	0.02	8259430	0.30	2.56	0.98
'KN805370.1'	0.00	0.22	25.18	2.66	0.36	0.23	1674554	0.63	8.98	0.96
'JSUV0145019	0.45	0.28	22.58	3.76	0.18	0.12	132856	0.00	6.16	0.93
'CP009313.1'	0.18	0.25	8.18	0.61	0.68	0.37	7714110	0.12	13.83	0.99
'JMCE0100000	0.04	0.07	53.26	1.83	0.36	0.09	26981232	0.12	4.36	0.99
'HG977522.1'	0.08	0.12	59.58	4.02	0.33	0.14	3780190	0.77	3.38	0.97
'HG977526.1'	0.09	0.12	59.43	3.91	0.32	0.13	3788589	0.68	3.38	0.97
'CDMT010099	0.85	0.14	32.63	2.67	0.05	0.02	239477	0.00	3.30	0.96
'CDHN010000	0.45	0.09	24.72	0.86	0.18	0.05	8525130	0.00	5.80	0.99
'CDGI0100000	0.58	0.13	60.60	6.48	0.07	0.03	300412	9.52	2.20	0.93
'CDFH0109686	0.16	0.11	102.18	12.33	0.20	0.08	435040	0.16	2.45	0.93
'CDFF0107129	0.00	-1.00	35.83	3.14	0.40	0.07	158937	0.40	6.62	0.93
'CDFG0107762	0.29	0.25	19.39	2.92	0.30	0.18	171561	0.03	7.86	0.96
'CDFA0102575	0.00	-1.00	48.38	6.90	0.11	0.03	563726	0.08	9.00	0.82
'CDFD0105430	0.69	0.24	6.84	0.49	0.31	0.15	793959	0.01	13.39	0.99
'CDFK0121708	0.14	0.23	18.91	2.62	0.41	0.22	187094	0.24	8.98	0.97
'CDEZ0102249	0.00	-1.00	43.99	3.89	0.35	0.07	221343	0.01	5.65	0.92
'CDFC0106205	0.00	-1.00	179.01	41.77	0.05	0.01	255147	0.56	7.67	0.77
'CDFI0110670	0.16	0.12	99.09	12.88	0.20	0.08	380686	0.24	2.49	0.93
'CDFB0106255	0.00	-1.00	28.70	2.90	0.29	0.07	157839	0.13	9.36	0.92
'CDFN0104172	0.00	-1.00	20.27	2.33	0.50	0.13	74710	0.00	9.56	0.93
'CDFE0106259	0.00	-1.00	28.58	2.33	0.28	0.06	359022	0.25	9.14	0.92
'CDFL0121941	0.00	-1.00	37.92	5.01	0.22	0.06	113305	0.11	9.05	0.88
'CDFJ0121495	0.24	0.21	17.82	2.18	0.34	0.16	136028	0.25	8.85	0.97
'CM003114.1'	0.26	0.05	158.81	3.98	0.12	0.03	42390363	3.62	1.37	0.98
'KN805525.1'	0.10	0.06	133.18	6.19	0.23	0.06	6356336	4.07	2.00	0.97
'JTLS01000001	0.15	0.25	19.90	3.25	0.39	0.22	97325	0.00	8.70	0.96
'CP010455.1'	0.00	-1.00	28.01	0.97	0.39	0.05	4650162	0.00	7.46	0.97
'CP010456.1'	0.00	-1.00	28.01	0.97	0.39	0.05	4650152	0.00	7.46	0.97
'JRZD0101996	0.64	0.20	29.23	3.82	0.10	0.05	141285	0.09	4.31	0.94
'KN839835.1'	0.00	-1.00	47.78	4.04	0.18	0.05	2444957	1.33	5.94	0.87
'KN838536.1'	0.00	0.24	46.76	7.39	0.32	0.24	841987	1.47	5.13	0.91
'KN832554.1'	0.00	-1.00	29.14	1.81	0.28	0.07	4119651	1.37	7.72	0.92

'KN837076.1'	0.00	-1.00	80.45	5.90	0.14	0.03	3052012	12.56	4.13	0.87
'KN835132.1'	0.85	0.15	19.57	1.32	0.08	0.03	566938	0.00	5.21	0.98
'KN834749.1'	0.00	0.24	129.59	25.30	0.06	0.05	3435311	0.62	4.78	0.79
'KN833685.1'	0.24	0.22	23.75	2.47	0.32	0.20	947096	4.73	5.79	0.96
'KN832970.1'	0.00	0.21	137.41	28.13	0.07	0.06	1656816	5.95	4.49	0.82
'KN832870.1'	0.00	-1.00	82.82	5.86	0.10	0.02	6606482	0.12	4.82	0.85
'KN831944.1'	0.00	-1.00	791.71	186.48	0.02	0.00	2369842	6.47	4.06	0.72
'KN831768.1'	0.00	-1.00	33.24	1.37	0.48	0.08	3307763	3.87	5.33	0.96
'KN824823.1'	0.26	0.26	26.73	4.20	0.28	0.19	235740	0.00	5.68	0.93
'KN824277.1'	0.93	0.12	17.22	0.72	0.07	0.02	1897233	3.31	5.51	0.99
'KN822004.1'	0.00	-1.00	68.56	4.74	0.20	0.03	1200568	2.91	4.73	0.90
'KN822942.1'	0.89	0.13	15.18	0.79	0.09	0.03	927182	9.77	5.85	0.99
'KN819323.1'	0.00	-1.00	46.04	3.24	0.22	0.05	2811131	11.37	5.02	0.89
'KN818222.1'	0.85	0.13	19.13	0.96	0.08	0.03	1491530	3.95	5.14	0.98
'KN817518.1'	0.28	0.14	24.28	1.53	0.27	0.11	1726651	0.05	6.71	0.98
'JWZT0100533	0.86	0.10	41.21	2.28	0.04	0.01	1160795	18.66	2.13	0.97
'JXLN0101223	0.83	0.14	34.53	2.75	0.05	0.02	358824	0.00	3.21	0.95
'AP014655.1'	0.22	0.22	12.66	0.93	0.49	0.27	4861258	0.18	9.90	0.98
'DF933830.1'	0.06	0.14	35.57	2.28	0.42	0.18	4195530	1.34	5.10	0.98
'LD000290.1'	0.00	-1.00	206.26	23.51	0.25	0.03	296819	13.53	1.98	0.90
'CP007359.2'	0.00	-1.00	28.27	1.21	0.29	0.05	4685849	0.84	8.45	0.96
'CP007358.2'	0.00	-1.00	28.27	1.21	0.29	0.05	4685849	0.84	8.45	0.96
'LD132766.1'	0.00	-1.00	194.74	22.63	0.25	0.03	296819	13.53	1.96	0.89
'AP014548.1'	0.82	0.10	33.88	1.38	0.05	0.02	2915920	0.34	3.28	0.98
'CP010813.1'	0.32	0.10	52.10	2.58	0.17	0.06	5227410	0.00	3.22	0.98
'KN840438.1'	0.54	0.16	16.08	1.10	0.20	0.08	612519	0.04	7.85	0.98
'CDGK0100211	0.59	0.19	21.63	2.62	0.14	0.06	90123	0.00	5.92	0.96
'CP009805.1'	0.00	-1.00	83.36	4.25	0.20	0.03	4109373	0.00	3.78	0.91
'CP010287.1'	0.70	0.09	90.19	4.97	0.03	0.01	1891022	0.01	1.43	0.95
'JSUI01006542	0.48	0.18	30.69	3.43	0.14	0.07	234769	0.27	4.71	0.95
'CP009846.1'	0.00	-1.00	36.11	1.02	0.45	0.05	4616187	0.00	5.46	0.98
'CP009996.1'	0.00	-1.00	35.16	1.08	0.45	0.05	4672688	0.00	5.63	0.97
'CP010293.1'	0.00	-1.00	34.67	1.12	0.45	0.05	4563069	2.19	5.53	0.97
'CP007299.1'	0.34	0.22	8.53	0.58	0.49	0.24	4411953	0.16	12.70	0.99
'CP010005.1'	0.40	0.10	50.91	2.33	0.14	0.05	5675189	0.00	3.11	0.98
'KN846951.1'	0.34	0.14	23.57	1.15	0.26	0.11	9941542	0.10	5.80	0.98
'KN846956.1'	0.00	0.17	24.44	1.71	0.57	0.29	6061779	0.04	6.81	0.98
'KN846969.1'	0.52	0.13	21.09	1.01	0.17	0.07	5379281	0.07	6.07	0.99
'KN846980.1'	0.84	0.12	17.10	0.72	0.09	0.03	2736732	0.03	6.02	0.99
'KN847040.1'	0.42	0.13	21.17	1.00	0.22	0.09	8230860	0.01	6.27	0.99
'KN847317.1'	0.62	0.14	19.14	0.89	0.15	0.06	5554269	0.01	6.06	0.99
'KN847332.1'	0.40	0.13	23.54	1.22	0.21	0.08	4470873	0.18	5.89	0.98
'KN847475.1'	0.72	0.10	19.30	0.72	0.11	0.03	5132325	0.07	5.76	0.99
'KN848630.1'	0.83	0.14	22.10	1.40	0.08	0.03	801752	0.78	4.82	0.98
'KN848855.1'	0.60	0.15	25.42	1.67	0.12	0.06	1503451	0.02	4.88	0.97
'KN848831.1'	0.57	0.15	26.48	1.62	0.13	0.06	2152106	0.01	4.79	0.97
'KN848525.1'	0.59	0.14	26.16	1.51	0.12	0.05	2154380	0.04	4.79	0.98
'KN847492.1'	0.24	0.14	24.20	1.34	0.32	0.14	6183525	0.03	6.18	0.98
'DF818471.1'	0.51	0.17	24.80	2.00	0.16	0.07	584812	0.06	5.19	0.97
'DF818696.1'	0.67	0.18	20.21	1.74	0.12	0.06	398723	0.24	5.71	0.97
'DF819225.1'	0.39	0.16	23.32	1.91	0.22	0.10	480923	0.53	6.06	0.98
'DF820090.1'	0.84	0.11	19.82	0.94	0.08	0.03	892765	0.03	5.20	0.99
'DF822067.1'	0.00	0.12	92.90	6.14	0.27	0.12	9881032	3.59	3.09	0.95
'DF825946.1'	0.00	-1.00	86.25	2.14	0.28	0.03	16292344	1.24	3.38	0.96
'DF938580.1'	0.27	0.18	19.93	1.48	0.33	0.17	2175025	0.01	7.15	0.98
'KN847520.1'	0.56	0.10	24.18	0.94	0.14	0.04	6137194	0.01	5.22	0.99
'KN847529.1'	0.36	0.15	22.01	1.48	0.24	0.10	1367393	0.01	6.47	0.98
'KN848129.1'	0.68	0.15	24.83	1.68	0.10	0.04	1031261	0.26	4.78	0.97
'KN847973.1'	0.70	0.14	24.27	1.41	0.10	0.04	1466070	0.13	4.81	0.98
'KN847920.1'	0.75	0.14	23.07	1.32	0.09	0.04	1473963	0.04	4.87	0.98
'KN847896.1'	0.66	0.11	25.28	1.09	0.10	0.03	2144985	0.14	4.77	0.99
'KN848062.1'	0.68	0.12	18.81	0.83	0.13	0.04	2522639	0.05	6.15	0.99
'KN848947.1'	0.70	0.15	24.64	1.67	0.10	0.04	914829	0.00	4.78	0.97
'CM003138.1'	0.54	0.11	52.60	3.38	0.09	0.03	1736449	0.53	2.71	0.96
'CM003139.1'	0.62	0.10	49.49	2.65	0.07	0.02	1789956	0.57	2.71	0.97
'KN848889.1'	0.62	0.12	25.26	1.33	0.12	0.04	1959555	0.02	4.84	0.98
'KN848504.1'	0.29	0.17	31.72	2.50	0.25	0.13	2178346	0.72	4.74	0.96
'KN849052.1'	0.58	0.14	26.43	1.68	0.12	0.05	1390242	1.58	4.75	0.98
'KN848922.1'	0.66	0.12	25.24	1.18	0.10	0.04	2179612	1.03	4.73	0.98
'JXWP0100000	0.00	-1.00	31.96	1.03	0.42	0.06	9216672	6.23	5.86	0.97
'JPID01000044	0.73	0.12	35.59	2.41	0.06	0.02	564282	0.00	3.36	0.97
'JRVE0100000	0.36	0.14	31.72	2.21	0.19	0.08	1475703	0.01	4.92	0.97
'CP007437.1'	0.18	0.25	7.61	0.62	0.69	0.36	2501300	0.17	14.94	0.99
'KN849314.1'	0.00	-1.00	117.57	7.00	0.28	0.03	667620	6.24	2.72	0.94
'JWHW010286	0.10	0.10	68.77	4.78	0.28	0.10	1977235	1.85	3.38	0.97
'JWTJ0100056	0.48	0.25	10.09	1.04	0.32	0.16	238318	0.02	11.39	0.98
'CP010940.1'	0.58	0.09	54.27	2.60	0.07	0.02	2756431	0.11	2.52	0.97
'CP010941.1'	0.61	0.10	53.11	2.68	0.07	0.02	2672185	0.13	2.50	0.97
'CP010942.1'	0.64	0.08	61.89	2.81	0.06	0.02	2339868	0.12	2.16	0.97
'CP010943.1'	0.59	0.09	53.94	2.41	0.07	0.02	2730860	0.10	2.51	0.98
'CP010944.1'	0.57	0.09	54.53	2.45	0.08	0.02	2726524	0.10	2.51	0.98
'KN880431.1'	0.79	0.15	16.00	1.04	0.11	0.04	527617	0.00	6.66	0.98
'KN882095.1'	0.00	-1.00	50.99	4.18	0.16	0.04	1553176	0.72	6.58	0.88
'KN882203.1'	0.04	0.12	120.86	14.89	0.28	0.12	842595	9.28	2.09	0.93
'CP010895.1'	0.38	0.21	8.41	0.52	0.46	0.21	4392860	4.14	12.16	0.99
'KN902229.1'	0.39	0.15	49.03	5.07	0.14	0.07	421248	0.48	3.32	0.94
'CP010952.1'	0.62	0.10	52.64	2.53	0.07	0.02	2788353	0.14	2.49	0.97
'KN905583.1'	0.70	0.13	24.35	1.45	0.10	0.04	802859	0.00	4.83	0.98
'CP016603.1'	0.00	0.34	21.46	3.02	0.30	0.30	3764434	0.91	9.45	0.93
'JYGD0100011	0.00	-1.00	42.32	3.26	0.25	0.06	1533535	0.02	6.19	0.90
'KN905849.1'	0.15	0.06	149.71	6.54	0.18	0.04	8606022	2.45	1.81	0.97
'LM525555.1'	0.50	0.08	148.62	9.74	0.05	0.02	1686976	0.03	1.13	0.93
'JYIL01003741	0.50	0.18	20.92	1.79	0.18	0.09	790089	2.35	6.04	0.97
'LN736360.1'	0.86	0.12	23.58	1.18	0.07	0.02	1828985	0.00	4.45	0.98
'LN719426.1'	0.51	0.10	50.75	2.73	0.10	0.03	2293163	5.59	2.77	0.97
'LK996042.1'	0.64	0.12	63.18	5.76	0.05	0.02	360011	0.61	2.22	0.94
'LK927530.1'	0.46	0.12	55.29	4.67	0.11	0.04	593230	1.07	2.88	0.95
'CP010969.1'	0.30	0.12	69.52	5.80	0.15	0.06	1344642	0.01	2.71	0.95
'CP009770.1'	0.16	0.09	315.80	47.67	0.14	0.05	973501	0.04	1.05	0.87
'LM523158.1'	0.57	0.06	179.32	7.29	0.04	0.01	6158768	0.13	0.85	0.95
'LK995546.1'	0.65	0.07	76.47	2.55	0.05	0.01	7271431	0.03	1.75	0.98
'LK023313.1'	0.69	0.09	50.72	2.26	0.06	0.02	3088551	0.10	2.49	0.97
'LK978189.1'	0.61	0.08	56.35	2.17	0.07	0.02	4215530	0.00	2.45	0.98
'LN627129.1'	0.68	0.12	28.17	1.50	0.09	0.03	1564510	4.67	4.07	0.98
'LL999048.1'	0.38	0.06	323.68	18.21	0.06	0.02	4975635	0.00	0.64	0.93
'JQHZ0107787	0.28	0.11	196.02	40.90	0.11	0.04	250200	0.22	1.51	0.87

'DF939120.1'	0.00	-1.00	44.37	1.36	0.25	0.03	4813516	5.38	6.89	0.97
'LM676446.1'	0.59	0.17	207.61	122.08	0.04	0.02	37242	2.05	1.59	0.64
'LL188370.1'	0.06	0.15	43.59	4.77	0.35	0.15	411161	5.46	4.88	0.96
'FJNM0100000	0.78	0.13	48.77	4.75	0.04	0.02	230458	0.04	2.48	0.93
'LL569982.1'	0.69	0.21	36.56	6.39	0.07	0.04	80097	2.13	3.49	0.90
'LL233096.1'	0.81	0.15	29.61	2.44	0.06	0.03	346221	3.33	3.59	0.96
'DF836290.1'	0.71	0.12	43.18	3.33	0.06	0.02	539271	26.26	2.11	0.96
'DF933568.1'	0.06	0.10	110.62	9.62	0.27	0.10	1389596	1.76	2.46	0.95
'LK393106.1'	0.88	0.27	37.54	12.53	0.04	0.03	14979	17.00	2.88	0.78
'CM003179.1'	0.00	0.05	195.48	4.37	0.34	0.08	338368434	5.60	1.28	0.98
'LL000001.1'	0.59	0.13	71.45	10.03	0.06	0.02	141144	5.91	2.12	0.91
'LK903567.1'	0.38	0.17	181.00	92.18	0.08	0.04	45194	0.79	2.02	0.78
'LK945525.1'	0.64	0.16	30.83	2.86	0.09	0.04	410021	0.86	4.06	0.96
'LK939141.1'	0.76	0.12	27.41	1.60	0.07	0.03	823818	0.05	4.18	0.98
'KN983729.1'	0.34	0.04	270.87	7.15	0.07	0.01	35326507	5.74	0.77	0.97
'KN972984.1'	0.00	-1.00	199.05	4.54	0.25	0.01	17385548	17.70	1.46	0.96
'LK871857.1'	0.75	0.11	28.55	1.56	0.07	0.03	1023000	2.33	3.96	0.98
'LL319179.1'	0.50	0.14	45.82	4.40	0.11	0.05	427667	2.51	3.26	0.95
'LK964160.1'	0.64	0.10	66.37	4.21	0.05	0.02	1059003	0.42	2.06	0.96
'LL361184.1'	0.57	0.60	52.72	56.16	0.06	0.08	16658	0.12	4.63	0.49
'LL710276.1'	0.67	0.14	46.43	4.66	0.06	0.03	226215	1.23	2.79	0.94
'LM413552.1'	0.46	0.14	76.70	11.11	0.09	0.04	176877	2.35	2.29	0.91
'LL748445.1'	0.61	0.19	33.93	5.10	0.09	0.05	74128	0.81	3.91	0.93
'LM383430.1'	0.71	0.12	51.42	4.26	0.05	0.02	355889	0.07	2.45	0.95
'LM397340.1'	0.54	0.16	62.02	10.09	0.08	0.04	146297	8.81	2.44	0.90
'LM583056.1'	0.59	0.15	31.01	2.71	0.11	0.04	300312	5.13	4.12	0.96
'LM530258.1'	0.48	0.15	42.07	4.10	0.12	0.05	466740	0.27	3.58	0.95
'LM433384.1'	0.58	0.16	30.14	2.71	0.11	0.05	394171	2.71	4.27	0.96
'LM537584.1'	0.44	0.18	106.56	33.07	0.08	0.04	55543	0.52	2.18	0.82
'LM462759.1'	0.58	0.19	37.85	6.50	0.10	0.05	70251	20.32	3.07	0.92
'LM013521.1'	0.52	0.16	34.70	4.04	0.12	0.05	139782	3.09	4.12	0.95
'LM065042.1'	0.31	0.15	103.41	21.71	0.12	0.06	150384	0.36	2.30	0.88
'LL876846.1'	0.35	0.10	96.49	10.17	0.11	0.04	387228	0.62	2.10	0.94
'LM149319.1'	0.24	0.14	169.15	49.63	0.13	0.06	130326	3.55	1.90	0.86
'LL956979.1'	0.22	0.13	170.13	39.59	0.14	0.06	191056	2.35	1.82	0.88
'LL900201.1'	0.51	0.13	39.74	3.13	0.11	0.05	594668	1.40	3.59	0.96
'LM211380.1'	0.50	0.19	48.94	9.62	0.10	0.05	88003	4.39	3.23	0.90
'LM125182.1'	0.61	0.11	39.36	2.69	0.09	0.03	522335	0.38	3.44	0.97
'LM035857.1'	0.73	0.14	27.99	2.14	0.08	0.03	362365	3.46	4.01	0.97
'LL718354.1'	0.78	0.12	31.74	1.97	0.06	0.02	779348	0.09	3.57	0.97
'LM000001.1'	0.61	0.14	67.85	9.37	0.06	0.02	144416	0.15	2.27	0.91
'LN590686.1'	0.08	0.05	201.96	5.97	0.22	0.04	29126390	1.95	1.49	0.98
'LN000001.1'	0.86	0.19	27.47	3.61	0.06	0.03	89810	14.31	3.36	0.93
'CP010981.1'	0.00	0.24	66.21	9.11	0.08	0.07	6210300	0.00	7.12	0.88
'KN985990.1'	0.26	0.04	302.59	7.56	0.10	0.02	43518125	9.24	0.74	0.97
'LK931502.1'	0.00	-1.00	28.65	1.18	0.28	0.05	4814038	0.05	8.47	0.96
'LN681234.1'	0.63	0.06	247.31	13.14	0.02	0.01	3456852	0.22	0.62	0.92
'LK931482.1'	0.00	-1.00	28.61	1.18	0.28	0.05	4620579	0.06	8.50	0.96
'LN679998.1'	0.64	0.06	246.97	13.50	0.02	0.01	3394044	0.16	0.62	0.92
'KQ012552.1'	0.36	0.04	273.56	6.44	0.07	0.01	47169369	1.21	0.79	0.97
'KQ030498.1'	0.00	-1.00	26.00	1.40	0.40	0.08	1668383	0.20	7.77	0.95
'KQ009060.1'	0.37	0.03	278.24	5.66	0.06	0.01	63123227	1.61	0.76	0.98
'KQ012994.1'	0.17	0.04	241.56	7.28	0.15	0.03	29265399	14.15	0.96	0.97
'LD336106.1'	0.00	-1.00	68.40	1.83	0.18	0.02	10381176	5.24	5.83	0.97
'KQ001645.1'	0.41	0.12	44.89	3.31	0.14	0.05	752713	5.47	3.44	0.97
'AZNP0100000	0.00	0.10	98.73	7.54	0.33	0.12	2373158	0.18	2.87	0.96
'CP009942.1'	0.00	-1.00	8.90	0.47	0.50	0.13	3509051	0.00	17.97	0.97
'CP010430.1'	0.00	-1.00	19.26	0.73	0.47	0.08	4566144	0.03	9.10	0.97
'JZRJ0100018	0.00	-1.00	24.23	2.13	0.49	0.10	111369	0.00	8.10	0.94
'JZRK0100027	0.10	0.29	19.97	3.84	0.44	0.29	88826	0.00	8.64	0.95
'JZRL0100001	0.84	0.15	41.77	4.70	0.04	0.02	133119	0.00	2.73	0.93
'JZRM0100002	0.76	0.18	26.85	2.69	0.07	0.04	327894	0.00	4.22	0.95
'JZRN0100003	0.72	0.17	47.77	6.90	0.05	0.03	117971	0.00	2.73	0.90
'JZRO0100023	0.51	0.17	40.30	5.70	0.11	0.05	96484	0.00	3.84	0.94
'JYFM0200045	0.39	0.14	26.20	1.61	0.20	0.09	2741725	0.14	5.54	0.98
'JZUQ0100000	0.13	0.13	32.84	1.91	0.34	0.13	3878513	0.07	5.58	0.98
'JZJM0100010	0.28	0.13	31.95	1.87	0.24	0.09	3290802	0.05	5.11	0.98
'KQ031394.1'	0.20	0.06	123.24	4.34	0.17	0.04	13053552	5.77	1.85	0.98
'LAJZ0100000	0.53	0.14	26.64	1.85	0.14	0.05	602152	0.26	5.03	0.98
'LAKA0100005	0.51	0.13	27.01	1.59	0.15	0.06	1673914	0.64	4.97	0.98
'JZKD01S2518	0.00	-1.00	265.42	15.72	0.26	0.02	1508258	10.65	1.27	0.92
'JWIW0100010	0.09	0.17	31.04	2.52	0.40	0.21	2505574	0.88	5.72	0.97
'JWIV0100006	0.00	-1.00	68.12	4.86	0.14	0.03	5443851	0.52	4.82	0.86
'JWIU0100009	0.00	-1.00	31.79	1.00	0.44	0.05	3626568	2.13	6.55	0.98
'HE798385.1'	0.00	-1.00	11.13	0.32	0.69	0.10	6916670	0.20	13.15	0.99
'CM003192.1'	0.00	0.17	31.53	2.54	0.49	0.25	2961351	1.68	5.84	0.97
'FO203501.1'	0.00	0.23	23.89	2.33	0.31	0.21	5270770	0.08	9.32	0.96
'LAEV0100223	0.07	0.13	45.79	4.38	0.33	0.13	479719	0.67	5.09	0.97
'CP007357.2'	0.00	-1.00	28.27	1.21	0.29	0.05	4685841	0.87	8.45	0.96
'CP007361.2'	0.00	-1.00	28.27	1.21	0.29	0.05	4685835	0.84	8.45	0.96
'CP002710.1'	0.03	0.13	31.19	2.10	0.43	0.16	1837132	0.00	6.71	0.98
'JXSR01S0000	0.09	0.08	474.60	102.40	0.16	0.05	778843	14.61	0.90	0.88
'HE605379.1'	0.76	0.12	33.76	2.01	0.06	0.02	1042626	0.01	3.38	0.97
'HE605380.2'	0.79	0.10	33.20	1.68	0.06	0.02	1042731	0.01	3.38	0.98
'CP009836.1'	0.00	-1.00	34.83	1.05	0.46	0.05	4605437	0.00	5.64	0.98
'FO393427.1'	0.38	0.10	115.41	11.63	0.09	0.03	570238	2.23	1.68	0.93
'FO393428.1'	0.42	0.11	105.26	10.72	0.08	0.03	545458	5.44	1.66	0.92
'LAPY0100000	0.00	-1.00	86.08	4.33	0.29	0.03	659152	12.09	3.46	0.95
'CM003196.1'	0.00	-1.00	10.07	0.47	0.39	0.09	3975521	0.01	17.82	0.97
'CM003194.1'	0.00	-1.00	10.03	0.46	0.41	0.09	4084272	0.01	17.73	0.98
'KQ039406.1'	0.32	0.05	197.85	5.61	0.09	0.02	24051356	2.01	1.09	0.97
'CP010274.2'	0.50	0.11	56.48	3.14	0.09	0.03	2848408	0.00	2.65	0.96
'CP010275.2'	0.48	0.11	57.17	3.43	0.10	0.04	2847905	0.01	2.65	0.96
'CP010276.2'	0.48	0.11	57.10	3.22	0.09	0.04	2850726	0.00	2.65	0.96
'CP010277.2'	0.49	0.11	56.81	3.30	0.09	0.04	2806870	0.00	2.65	0.96
'CP010278.2'	0.49	0.11	56.74	3.24	0.09	0.04	2805502	0.01	2.65	0.96
'CP010946.1'	0.50	0.21	8.57	0.52	0.36	0.17	4898027	0.04	11.89	0.99
'KQ041443.1'	0.40	0.06	116.58	4.81	0.09	0.02	4914789	1.01	1.59	0.97
'JXMZ0211197	0.00	0.22	62.26	22.53	0.34	0.19	38109	0.00	5.20	0.91
'FO818640.1'	0.23	0.12	39.65	2.10	0.26	0.10	6228153	0.01	4.24	0.98
'CM003202.2'	0.00	-1.00	222.11	32.51	0.02	0.01	11834931	0.01	4.87	0.69
'CM003210.1'	0.26	0.09	58.97	2.20	0.20	0.06	20182852	0.00	3.04	0.98
'JPLX0100000	0.21	0.11	189.89	38.37	0.14	0.05	263083	0.50	1.68	0.89
'CP006437.1'	0.55	0.19	37.19	3.50	0.10	0.06	2069293	0.03	3.53	0.93
'CP006438.1'	0.54	0.22	35.17	3.69	0.11	0.07	2208708	0.05	3.61	0.92
'CP004102.1'	0.77	0.11	40.18	2.17	0.05	0.02	1466847	0.14	2.86	0.97

'CP006377.1'	0.58	0.18	38.02	3.38	0.09	0.05	1777883	0.04	3.42	0.94
'CP006378.1'	0.50	0.21	37.07	3.83	0.11	0.07	2444098	0.02	3.57	0.92
'CP006435.1'	0.64	0.15	37.81	2.98	0.08	0.04	1619914	0.02	3.33	0.95
'CP006436.1'	0.49	0.21	37.27	3.87	0.12	0.08	2384574	0.05	3.59	0.92
'CP006439.1'	0.68	0.15	37.47	2.85	0.07	0.03	1475342	0.03	3.29	0.95
'CP006440.1'	0.57	0.17	37.68	3.32	0.09	0.05	1822193	0.03	3.44	0.94
'CP006441.1'	0.40	0.25	36.93	4.65	0.15	0.11	2753647	0.02	3.68	0.90
'CP006442.1'	0.50	0.23	35.62	3.90	0.12	0.08	2829437	0.03	3.63	0.92
'CP006443.1'	0.60	0.17	37.95	3.27	0.09	0.05	1683453	0.02	3.39	0.94
'CP006444.1'	0.60	0.15	38.88	3.08	0.09	0.04	1631539	0.06	3.37	0.95
'CP006445.1'	0.57	0.17	38.63	3.32	0.09	0.05	1723843	0.03	3.40	0.94
'CP006446.1'	0.61	0.16	37.72	2.99	0.09	0.04	1673802	0.06	3.41	0.95
'CP006447.1'	0.56	0.19	37.39	3.43	0.10	0.06	2149348	0.05	3.48	0.93
'CP006448.1'	0.63	0.15	38.50	3.01	0.08	0.04	1606260	0.09	3.32	0.95
'CP006449.1'	0.52	0.18	38.70	3.59	0.11	0.06	1925945	0.05	3.49	0.94
'CP006450.1'	0.53	0.19	37.20	3.54	0.11	0.07	2374204	0.06	3.53	0.93
'CP006451.1'	0.50	0.20	37.61	3.86	0.11	0.07	2348550	0.05	3.56	0.93
'CP006452.1'	0.52	0.21	36.01	3.75	0.11	0.08	2586410	0.03	3.60	0.92
'CP006453.1'	0.56	0.19	37.52	3.60	0.10	0.06	1913250	0.06	3.46	0.93
'CP006454.1'	0.61	0.17	38.08	3.39	0.09	0.05	1693948	0.09	3.36	0.94
'CP006455.1'	0.62	0.16	38.03	3.09	0.08	0.04	1633533	0.02	3.36	0.95
'CP006456.1'	0.60	0.17	37.58	3.15	0.09	0.05	1769893	0.06	3.41	0.94
'CP006457.1'	0.62	0.16	37.67	3.10	0.08	0.04	1627349	0.04	3.38	0.95
'CP006458.1'	0.48	0.19	37.66	3.65	0.12	0.07	2458844	0.06	3.60	0.93
'CP006459.1'	0.64	0.17	36.38	3.02	0.08	0.04	1745332	0.02	3.41	0.94
'CP006460.1'	0.55	0.20	36.20	3.55	0.10	0.06	2345166	0.02	3.56	0.93
'CP006461.1'	0.65	0.16	37.28	2.96	0.08	0.04	1620100	0.05	3.36	0.95
'CP006462.1'	0.60	0.18	36.72	3.31	0.09	0.05	1912773	0.03	3.46	0.94
'CP006463.1'	0.56	0.20	36.21	3.51	0.10	0.06	2292469	0.03	3.55	0.93
'CP006464.1'	0.53	0.21	36.20	3.69	0.11	0.07	2456439	0.04	3.58	0.92
'CP006465.1'	0.55	0.20	36.12	3.62	0.10	0.07	2344165	0.02	3.56	0.93
'CP006466.1'	0.61	0.17	37.17	3.21	0.09	0.05	1821963	0.04	3.43	0.94
'CP006468.1'	0.56	0.19	36.71	3.53	0.10	0.06	2116741	0.02	3.51	0.93
'CP006379.1'	0.59	0.17	38.37	3.48	0.09	0.05	1703401	0.04	3.40	0.94
'CP006380.1'	0.53	0.20	36.97	3.59	0.11	0.07	2259367	0.03	3.55	0.93
'CP006381.1'	0.60	0.16	38.54	3.32	0.09	0.05	1630274	0.04	3.37	0.94
'CP006382.1'	0.59	0.17	38.11	3.29	0.09	0.05	1836195	0.04	3.41	0.94
'CP006383.1'	0.49	0.20	37.04	3.70	0.12	0.08	2368220	0.02	3.61	0.93
'CP006384.1'	0.63	0.16	37.73	3.15	0.08	0.04	1601975	0.01	3.35	0.94
'CP006385.1'	0.56	0.20	36.23	3.49	0.10	0.06	2051347	0.04	3.54	0.93
'CP006386.1'	0.58	0.19	36.98	3.45	0.09	0.05	1933519	0.04	3.46	0.93
'CP006387.1'	0.61	0.17	37.92	3.20	0.08	0.05	1655007	0.08	3.37	0.94
'CP006388.1'	0.58	0.19	36.52	3.35	0.09	0.05	2004436	0.02	3.50	0.94
'CP006389.1'	0.58	0.18	37.45	3.27	0.09	0.05	1833356	0.03	3.43	0.94
'CP006390.1'	0.60	0.16	38.11	3.23	0.09	0.05	1698738	0.04	3.41	0.94
'CP006391.1'	0.45	0.21	38.17	4.01	0.13	0.09	2396136	0.03	3.62	0.92
'CP006392.1'	0.59	0.17	37.07	3.08	0.09	0.05	1886822	0.05	3.46	0.95
'CP006393.1'	0.61	0.16	37.80	3.09	0.08	0.04	1680891	0.08	3.37	0.95
'CP006394.1'	0.61	0.16	37.84	3.13	0.08	0.04	1684403	0.01	3.38	0.95
'CP006395.1'	0.52	0.19	38.23	3.62	0.11	0.06	2324682	0.04	3.51	0.93
'CP006396.1'	0.64	0.16	37.76	3.01	0.08	0.04	1579810	0.04	3.34	0.95
'CP006397.1'	0.54	0.18	38.69	3.51	0.10	0.06	1890044	0.02	3.45	0.94
'CP006398.1'	0.58	0.18	37.16	3.21	0.09	0.05	1899842	0.04	3.45	0.94
'CP006399.1'	0.56	0.18	36.76	3.18	0.10	0.06	2249646	0.08	3.53	0.94
'CP006400.1'	0.55	0.18	37.90	3.35	0.10	0.06	1960646	0.04	3.47	0.94
'CP006401.1'	0.57	0.17	38.81	3.41	0.09	0.05	1764339	0.06	3.42	0.94
'CP006402.1'	0.60	0.17	36.69	3.09	0.09	0.05	1869821	0.04	3.47	0.94
'CP006403.1'	0.53	0.19	37.07	3.49	0.10	0.06	2186031	0.04	3.57	0.93
'CP006404.1'	0.59	0.17	37.44	3.23	0.09	0.05	1944937	0.07	3.45	0.94
'CP006405.1'	0.52	0.19	37.89	3.54	0.11	0.06	2124836	0.06	3.56	0.94
'CP006406.1'	0.53	0.18	38.62	3.64	0.10	0.06	1929697	0.03	3.45	0.93
'CP006407.1'	0.59	0.17	38.50	3.40	0.09	0.05	1655040	0.06	3.37	0.94
'CP006408.1'	0.61	0.16	37.69	3.04	0.09	0.04	1702273	0.07	3.39	0.95
'CP006409.1'	0.56	0.17	39.48	3.47	0.09	0.05	1651440	0.02	3.38	0.94
'CP006410.1'	0.57	0.15	40.49	3.15	0.09	0.04	1519943	0.15	3.30	0.95
'CP006411.1'	0.54	0.18	38.43	3.42	0.10	0.06	1869687	0.05	3.45	0.94
'CP006412.1'	0.56	0.20	36.16	3.49	0.10	0.06	2267956	0.02	3.54	0.93
'CP006413.1'	0.57	0.19	36.42	3.36	0.10	0.06	2134488	0.03	3.51	0.93
'CP006414.1'	0.60	0.16	37.37	2.99	0.09	0.05	1760045	0.01	3.41	0.95
'CP006415.1'	0.40	0.23	37.65	4.22	0.14	0.10	2886794	0.05	3.66	0.92
'CP006416.1'	0.55	0.18	37.87	3.55	0.10	0.06	1874567	0.08	3.47	0.93
'CP006417.1'	0.59	0.18	37.43	3.22	0.09	0.05	1819213	0.04	3.42	0.94
'CP006418.1'	0.54	0.21	36.24	3.67	0.10	0.07	2306049	0.05	3.56	0.92
'CP006419.1'	0.57	0.16	39.30	3.36	0.09	0.05	1511883	0.15	3.36	0.94
'CP006420.1'	0.58	0.17	38.86	3.34	0.09	0.05	1668394	0.09	3.37	0.94
'CP006421.1'	0.61	0.16	38.23	3.15	0.09	0.04	1556556	0.04	3.34	0.95
'CP006422.1'	0.50	0.20	37.41	3.66	0.11	0.07	2326173	0.04	3.61	0.93
'CP006423.1'	0.50	0.19	38.16	3.74	0.11	0.07	2134898	0.03	3.53	0.93
'CP006424.1'	0.45	0.23	36.65	4.18	0.13	0.10	2797828	0.03	3.66	0.91
'CP006425.1'	0.63	0.16	37.64	3.15	0.08	0.04	1626852	0.01	3.36	0.94
'CP006426.1'	0.55	0.18	37.52	3.44	0.10	0.06	1960911	0.02	3.50	0.94
'CP006427.1'	0.55	0.17	39.22	3.57	0.10	0.05	1726487	0.04	3.41	0.94
'CP006428.1'	0.57	0.18	37.88	3.38	0.09	0.05	1895056	0.05	3.45	0.94
'CP006429.1'	0.53	0.19	37.78	3.64	0.11	0.06	2069828	0.06	3.50	0.93
'CP006430.1'	0.47	0.21	37.07	3.72	0.12	0.08	2560762	0.04	3.64	0.93
'CP006431.1'	0.62	0.16	38.43	3.10	0.08	0.04	1540968	0.06	3.33	0.95
'CP006432.1'	0.61	0.17	36.93	3.00	0.09	0.05	1813741	0.06	3.43	0.95
'CP006433.1'	0.57	0.18	38.23	3.56	0.09	0.05	1770650	0.05	3.42	0.93
'CP006434.1'	0.58	0.18	37.09	3.27	0.09	0.05	2024659	0.04	3.46	0.94
'DF850543.1'	0.00	-1.00	201.50	12.07	0.25	0.02	910847	4.92	1.94	0.93
'CM003214.1'	0.23	0.03	305.71	5.50	0.11	0.02	157394656	6.87	0.79	0.98
'JXRI01000001	0.00	-1.00	34.39	1.52	0.40	0.06	1469055	1.49	6.42	0.96
'DF424115.1'	0.08	0.11	386.44	151.72	0.18	0.07	189744	0.39	1.53	0.86
'DF955598.1'	0.00	-1.00	79.47	4.50	0.23	0.03	657067	0.09	4.88	0.94
'DF884991.1'	0.00	-1.00	193.45	9.60	0.25	0.02	1352076	3.95	1.95	0.94
'BBSZ0101381	0.55	0.14	60.50	7.66	0.08	0.03	155814	0.06	2.59	0.92
'LK979539.1'	0.41	0.12	97.25	12.28	0.09	0.04	298330	0.22	1.94	0.91
'LK391709.1'	0.21	0.14	24.68	1.54	0.32	0.13	3518759	0.00	6.78	0.98
'HF935907.1'	0.43	0.17	27.83	2.42	0.17	0.08	603142	0.03	5.19	0.97
'HF955199.1'	0.00	-1.00	59.06	2.05	0.28	0.03	2329561	10.95	4.79	0.97
'LN625279.1'	0.56	0.10	54.41	2.79	0.08	0.03	2831396	0.01	2.52	0.97
'HG423343.1'	0.00	-1.00	25.16	0.90	0.42	0.06	5486793	0.17	7.62	0.97
'JYJC0100172(	0.84	0.17	13.38	0.89	0.12	0.05	684572	0.09	7.42	0.98
'JYJD0100245(	0.69	0.21	14.45	1.23	0.15	0.08	463343	0.03	7.36	0.98
'JYJE0100008£	0.71	0.17	14.59	1.01	0.15	0.06	589903	0.04	7.35	0.98
'CVMT010000C	0.00	-1.00	147.41	12.76	0.04	0.01	6192107	2.92	5.06	0.80

'CM003258.1'	0.00	0.05	157.49	3.71	0.31	0.06	117247901	2.69	1.88	0.98
'CP011369.1'	0.00	-1.00	14.64	0.60	0.42	0.09	7099963	0.00	11.95	0.97
'LBFM0100674	0.60	0.17	29.38	3.27	0.11	0.05	158988	0.00	4.40	0.95
'LCTU0100002	0.90	0.26	7.82	0.66	0.18	0.10	637958	0.01	11.08	0.98
'LBFL0113244	0.46	0.16	48.22	6.92	0.11	0.05	132241	0.16	3.41	0.93
'JOKZ0100000	0.60	0.13	26.65	1.95	0.12	0.04	330970	0.00	4.82	0.98
'LCTV0100002	0.85	0.25	7.90	0.61	0.20	0.11	859004	0.00	11.18	0.99
'LBFN0110047	0.10	0.19	121.43	51.85	0.22	0.12	53192	0.15	3.18	0.87
'LBFO0110135	0.50	0.18	201.93	123.62	0.05	0.03	34859	0.63	1.78	0.70
'LBNK0100000	0.64	0.11	33.06	1.70	0.09	0.03	1593247	0.05	3.88	0.98
'LCTY0100000	0.63	0.09	45.09	2.21	0.07	0.02	1856900	0.59	2.92	0.98
'JARO0200000	0.00	0.10	199.34	31.63	0.24	0.09	616488	1.82	2.12	0.92
'LCTD0100001	0.69	0.14	42.72	3.78	0.06	0.03	454854	0.84	2.94	0.95
'CP011421.1'	0.00	0.31	30.11	4.27	0.16	0.15	5081069	4.78	9.15	0.91
'LCWF0100006	0.43	0.16	28.93	2.48	0.17	0.08	648486	0.05	4.88	0.97
'LAQI01000077	0.54	0.14	15.68	0.93	0.20	0.07	982000	0.03	7.96	0.99
'LCUC0100005	0.39	0.16	21.40	1.54	0.23	0.10	867068	0.01	6.69	0.98
'LAXH0100000	0.54	0.18	17.43	1.47	0.18	0.08	395740	0.08	7.17	0.98
'LN483142.1'	0.64	0.12	24.27	1.22	0.11	0.04	2575514	1.46	5.01	0.98
'KQ061688.1'	0.42	0.14	23.88	1.50	0.20	0.07	1018633	1.37	5.88	0.98
'KQ061256.1'	0.00	-1.00	>1000	>1000	0.05	0.01	461681	2.32	4.05	0.70
'KQ062009.1'	0.00	-1.00	164.96	26.42	0.06	0.01	962926	1.23	5.48	0.76
'KQ062599.1'	0.51	0.13	23.03	1.38	0.17	0.06	1067549	1.26	5.74	0.98
'LCZI01001061	0.15	0.17	43.04	5.37	0.29	0.15	388149	0.39	4.59	0.95
'JMDK0100020	0.30	0.14	32.50	2.46	0.22	0.09	829487	0.02	5.06	0.97
'LBLR0101438	0.33	0.07	194.89	15.78	0.09	0.03	1500620	0.72	1.16	0.93
'KQ062924.1'	0.15	0.09	112.88	8.37	0.21	0.07	2346892	22.43	1.68	0.95
'CP010913.1'	0.00	-1.00	90.07	17.16	0.05	0.02	2009762	0.00	7.76	0.72
'LBGY0100231	0.44	0.14	51.41	5.14	0.12	0.05	435429	3.47	3.11	0.95
'KQ079791.1'	0.25	0.04	271.62	5.78	0.11	0.02	80319138	3.45	0.88	0.98
'JXOR0100017	0.67	0.10	59.81	3.93	0.05	0.02	860417	0.03	2.18	0.96
'JXOZ0100187	0.00	0.13	63.03	6.59	0.34	0.14	646223	0.62	4.35	0.96
'JXOY0100044	0.22	0.16	57.34	8.88	0.20	0.09	167542	0.16	3.81	0.94
'JXPA0100073	0.40	0.15	41.95	4.16	0.15	0.07	397336	0.06	3.93	0.95
'JXPM0100155	0.42	0.17	35.64	4.17	0.15	0.07	191813	3.39	4.25	0.95
'JXPK0101978	0.48	0.20	249.35	228.93	0.06	0.03	24167	1.25	1.99	0.66
'JXPD0100112	0.67	0.17	40.03	4.98	0.07	0.03	146540	0.58	3.23	0.93
'JXPJ0100654	0.20	0.14	75.68	11.41	0.19	0.09	214895	0.39	3.13	0.93
'JXPY0100050	0.26	0.16	59.08	9.91	0.18	0.08	118018	0.08	3.71	0.93
'JXPV0100243	0.41	0.13	152.40	31.17	0.08	0.03	168171	0.63	1.57	0.85
'JXPS0100066	0.64	0.15	42.76	4.28	0.07	0.03	309339	1.77	3.03	0.94
'JXPR0100295	0.15	0.19	35.18	5.00	0.30	0.15	210824	2.89	5.57	0.95
'JXPZ0100420	0.42	0.16	61.21	9.17	0.11	0.05	164509	0.09	2.90	0.91
'JXPT0100226	0.46	0.14	43.99	4.08	0.12	0.05	460909	0.05	3.59	0.96
'JXQA0100112	0.12	0.26	518.32	>1000	0.18	0.12	15162	2.17	3.26	0.75
'JXPQ0100303	0.60	0.12	58.23	6.29	0.07	0.03	187270	1.93	2.50	0.94
'LDEV0100158	0.43	0.16	30.93	3.14	0.17	0.08	265286	0.17	4.72	0.96
'JXOU0102536	0.29	0.14	962.92	>1000	0.08	0.04	65472	3.30	1.33	0.72
'JXPF0100724	0.36	0.18	123.23	43.30	0.10	0.05	68688	1.93	2.14	0.81
'JXPH0100400	0.61	0.16	69.96	14.20	0.06	0.03	68208	2.35	2.36	0.86
'JXOW0120568	0.83	0.21	42.16	9.07	0.04	0.02	35797	0.19	3.02	0.84
'JXPU0107912	0.07	0.22	57.24	17.81	0.30	0.17	47253	7.84	4.44	0.91
'JXPW0101355	0.52	0.19	52.46	11.27	0.09	0.05	65218	10.53	2.86	0.88
'JXHJ0100126	0.43	0.17	187.37	88.74	0.07	0.04	72559	13.45	1.49	0.73
'JXOV0101555	0.31	0.13	70.30	8.65	0.14	0.06	244983	0.26	2.78	0.93
'JXOX0100694	0.01	0.13	78.45	9.83	0.34	0.14	430850	0.12	3.48	0.95
'JXOS0101768	0.65	0.15	45.12	5.39	0.07	0.03	128038	0.33	2.93	0.93
'JXPB0101264	0.17	0.27	19.76	3.08	0.38	0.25	196862	2.19	7.91	0.96
'JXPC0109884	0.63	0.21	59.44	15.16	0.06	0.03	43924	7.03	2.51	0.83
'JXPG0100143	0.39	0.16	103.44	23.28	0.09	0.05	117892	1.10	2.11	0.86
'JXPE0100378	0.62	0.14	74.15	10.29	0.05	0.02	148971	2.75	1.95	0.89
'JXPL0103258	0.42	0.20	156.31	83.78	0.07	0.04	48001	2.08	1.97	0.73
'KQ079922.1'	0.00	0.11	81.27	6.16	0.35	0.14	3453715	9.10	2.80	0.96
'JXOT0110267	0.63	0.20	47.40	9.76	0.07	0.04	53150	0.62	3.14	0.88
'JXPI01004095	0.58	0.13	66.81	7.90	0.06	0.03	195547	0.21	2.23	0.92
'JXPN0105407	0.40	0.27	37.19	11.86	0.15	0.10	24266	0.91	4.91	0.88
'JXPO0102630	0.33	0.21	18.77	1.76	0.29	0.15	695271	0.12	7.33	0.97
'JXPP0100424	0.53	0.23	63.59	20.47	0.08	0.05	43393	12.11	2.54	0.81
'KQ085882.1'	0.54	0.16	17.72	1.17	0.19	0.08	1181666	0.00	6.89	0.98
'LDKB0100008	0.74	0.19	18.33	1.73	0.11	0.05	232202	0.07	6.21	0.97
'LDJE0110591	0.12	0.24	45.88	14.99	0.28	0.16	28160	0.00	5.73	0.92
'LDEE0100000	0.23	0.14	21.81	1.15	0.34	0.13	4954631	0.22	6.96	0.99
'JRVG0100012	0.42	0.12	29.18	1.50	0.18	0.07	3261259	0.54	4.90	0.98
'JRVF0100071	0.53	0.15	27.13	2.06	0.14	0.06	791881	1.37	4.91	0.97
'JRVH0100190	0.60	0.19	28.57	3.66	0.11	0.05	128850	0.00	4.56	0.94
'LDKF0100002	0.13	0.14	56.16	6.33	0.26	0.11	496470	0.98	4.10	0.95
'CP011497.1'	0.00	-1.00	8.65	0.27	0.85	0.15	8648285	0.31	14.35	0.99
'KQ087177.1'	0.30	0.19	15.55	1.38	0.33	0.15	646641	0.00	9.36	0.98
'LM524968.1'	0.55	0.06	138.50	5.36	0.05	0.01	5851329	0.02	1.12	0.96
'CP011778.1'	0.89	0.12	30.88	1.60	0.05	0.02	1865156	13.47	2.91	0.97
'CP011813.1'	0.79	0.11	40.50	2.17	0.05	0.02	1531788	1.72	2.75	0.97
'KQ087363.1'	0.00	-1.00	361.56	83.42	0.02	0.01	3271182	0.16	5.27	0.66
'BBXB0100000	0.29	0.21	14.59	1.30	0.35	0.17	655053	5.81	9.28	0.98
'LFEI01000001	0.00	-1.00	350.80	20.61	0.18	0.01	1768510	1.75	1.52	0.92
'CP011886.1'	0.23	0.03	330.34	4.99	0.11	0.02	275920749	4.61	0.73	0.98
'CAQS0100000	0.37	0.16	50.16	7.06	0.14	0.07	201054	7.81	3.24	0.93
'LK391419.1'	0.50	0.04	237.51	4.84	0.04	0.01	63182071	6.72	0.71	0.98
'CP011941.1'	0.30	0.13	25.67	1.52	0.26	0.10	2661166	0.04	6.03	0.98
'LN609529.1'	0.30	0.04	366.07	13.57	0.08	0.02	16759152	0.03	0.62	0.95
'CP011799.1'	0.04	0.27	8.00	0.67	0.87	0.50	9153597	0.02	14.85	0.99
'CP011218.1'	0.44	0.15	34.49	2.49	0.15	0.07	1631557	1.99	4.00	0.96
'CP011219.1'	0.38	0.15	36.37	2.73	0.17	0.08	1651603	1.96	3.98	0.96
'CP011220.1'	0.40	0.15	35.85	2.73	0.17	0.08	1652679	1.96	3.99	0.96
'CP011221.1'	0.38	0.15	36.15	2.64	0.18	0.08	1635255	1.98	4.00	0.96
'CP011222.1'	0.43	0.14	35.02	2.30	0.16	0.07	1668151	1.94	4.00	0.97
'CP011223.1'	0.37	0.14	36.48	2.50	0.18	0.08	1766523	1.83	4.06	0.97
'CP011224.1'	0.43	0.14	34.26	2.36	0.16	0.07	1702560	1.90	4.09	0.97
'CP011225.1'	0.50	0.14	32.71	2.15	0.13	0.06	1661948	1.95	4.03	0.97
'CP011226.1'	0.45	0.14	34.40	2.35	0.15	0.07	1624874	1.99	3.99	0.97
'CP011227.1'	0.45	0.15	33.70	2.48	0.15	0.07	1592936	2.03	4.04	0.96
'CP011228.1'	0.54	0.12	33.67	1.89	0.12	0.04	1625548	1.99	3.85	0.98
'CP011229.1'	0.54	0.14	32.77	2.13	0.12	0.05	1557754	2.08	3.88	0.97
'CP011230.1'	0.53	0.13	33.12	2.04	0.12	0.05	1596604	2.03	3.89	0.97
'CP011231.1'	0.54	0.14	32.67	2.19	0.12	0.05	1574098	2.06	3.90	0.97
'LELE0100000	0.00	-1.00	69.23	11.12	0.18	0.05	240030	0.00	5.78	0.82
'CP011488.2'	0.61	0.09	100.67	6.05	0.04	0.01	1894154	0.75	1.39	0.94

'CP011001.1'	0.58	0.10	63.91	3.91	0.07	0.02	1352914	0.01	2.20	0.96
'CP011002.1'	0.58	0.10	63.89	3.91	0.07	0.02	1352885	0.01	2.20	0.96
'CP011003.1'	0.58	0.10	63.89	3.91	0.07	0.02	1352831	0.01	2.20	0.96
'CP011967.1'	0.00	-1.00	28.25	1.16	0.29	0.05	4585229	0.04	8.48	0.96
'LBMM0100000	0.00	-1.00	52.11	4.61	0.25	0.04	167361	0.00	6.62	0.92
'CP011952.1'	0.73	0.10	43.07	2.07	0.05	0.02	2423489	0.76	2.81	0.97
'DF196826.1'	0.03	0.10	118.10	11.73	0.29	0.10	1313578	16.65	2.12	0.95
'JXPX0104705	0.53	0.16	134.58	46.29	0.06	0.03	48722	0.93	1.86	0.78
'CDHK0100000	0.00	0.23	44.83	5.18	0.26	0.20	4658073	0.58	5.62	0.92
'CDSF0100000	0.55	0.18	11.11	0.71	0.26	0.11	1189627	0.92	10.19	0.99
'CVRJ0100000	0.88	0.18	26.77	2.76	0.05	0.03	230638	0.11	3.86	0.94
'LFMI0100020	0.26	0.21	27.10	3.55	0.27	0.15	286837	0.07	5.92	0.95
'CP011550.1'	0.75	0.11	41.07	2.24	0.06	0.02	1531682	0.00	2.85	0.97
'CBUC0100000	0.00	-1.00	113.44	7.76	0.16	0.02	1145416	7.19	3.85	0.91
'CDJJ0200002	0.00	0.14	175.74	58.49	0.27	0.12	101716	6.28	2.53	0.90
'CAWC010000	0.57	0.16	66.79	11.30	0.07	0.03	132813	0.01	2.36	0.88
'CBLZ0100421	0.06	0.20	967.06	>1000	0.19	0.11	34096	0.00	2.46	0.77
'CCSG010000	0.37	0.14	32.23	2.13	0.19	0.09	2195431	1.05	4.47	0.97
'LFLD01S0000	0.14	0.06	457.07	40.91	0.13	0.03	3920154	17.50	0.67	0.92
'CP012038.1'	0.00	-1.00	23.32	0.83	0.41	0.06	5088451	0.00	8.62	0.97
'LBNP0100003	0.74	0.14	40.69	3.63	0.06	0.02	282449	0.00	2.98	0.95
'JWJK0100010	0.81	0.12	40.26	2.93	0.05	0.02	442570	0.00	2.83	0.96
'CAIX01000001	0.96	0.13	19.69	1.21	0.06	0.02	398508	0.00	4.85	0.98
'CDMY010002	0.51	0.14	19.35	1.30	0.18	0.06	678612	0.97	6.87	0.98
'CEMG011818	0.75	0.22	18.33	2.06	0.11	0.06	157939	0.52	5.97	0.96
'CEMH012030	0.77	0.15	18.94	1.44	0.10	0.04	244086	0.34	5.77	0.98
'CVRD011076	0.20	0.29	17.39	2.96	0.37	0.24	176415	0.00	9.05	0.96
'CVRE0118106	0.65	0.26	15.08	2.22	0.16	0.09	77238	0.33	7.63	0.96
'LFUI01000001	0.22	0.14	43.56	3.51	0.24	0.10	914981	0.00	4.14	0.96
'LFYR0100072	0.00	-1.00	158.86	6.80	0.19	0.02	2654544	2.75	2.68	0.94
'JTLQ0114030	0.23	0.10	>1000	>1000	0.08	0.03	102731	1.21	1.05	0.77
'KQ236993.1'	0.00	-1.00	84.27	2.42	0.32	0.03	9749705	10.19	2.82	0.96
'KQ241597.1'	0.10	0.17	52.81	7.81	0.30	0.15	357797	26.33	3.11	0.94
'JOTR0100000	0.00	-1.00	127.84	4.61	0.28	0.02	2142037	15.90	2.23	0.96
'JRES0100116	0.46	0.06	112.82	4.45	0.07	0.02	5804300	4.03	1.45	0.97
'JJRW01S0000	0.00	0.15	136.83	25.21	0.22	0.11	715838	37.22	1.90	0.89
'JZLH01S0094	0.00	-1.00	146.43	12.64	0.27	0.03	336394	3.44	2.49	0.91
'CP012177.1'	0.73	0.10	43.05	2.08	0.05	0.02	2424118	0.76	2.81	0.97
'CP006806.1'	0.00	-1.00	35.24	1.08	0.45	0.05	4629525	0.00	5.63	0.98
'CP006794.1'	0.00	-1.00	35.10	1.09	0.45	0.05	4502257	0.00	5.61	0.97
'LGAM0100000	0.44	0.09	47.95	1.99	0.12	0.04	5074932	0.26	3.34	0.98
'LFRF0100000	0.59	0.13	14.49	0.63	0.19	0.06	2309933	2.20	8.04	0.99
'LGST0100004	0.78	0.14	28.26	1.88	0.07	0.03	860371	1.28	3.92	0.97
'CM003371.1'	0.08	0.05	180.76	5.03	0.22	0.05	42234885	2.75	1.59	0.98
'CP011326.1'	0.56	0.08	62.69	2.89	0.07	0.02	2047957	0.01	2.31	0.97
'CP011327.1'	0.56	0.08	62.69	2.93	0.07	0.02	2064350	0.44	2.30	0.97
'LGLR0100017	0.00	-1.00	53.36	5.33	0.15	0.04	1567143	0.29	6.35	0.85
'AJIL01000002	0.00	-1.00	153.24	11.84	0.15	0.02	1913627	49.01	1.68	0.88
'KQ417173.1'	0.00	-1.00	165.84	7.55	0.21	0.02	4064693	9.40	2.03	0.92
'LFNC0100000	0.77	0.13	21.64	1.23	0.09	0.03	853862	0.20	5.12	0.98
'CP012251.1'	0.00	-1.00	11.85	0.47	0.64	0.13	5218943	0.01	12.29	0.98
'JXZD01S0012	0.02	0.11	247.54	53.13	0.24	0.10	458668	2.47	1.62	0.88
'LN774881.1'	0.06	0.10	349.93	76.16	0.20	0.08	532684	0.00	1.29	0.87
'LM651872.1'	0.00	-1.00	91.56	19.51	0.07	0.02	607591	2.85	7.28	0.74
'LN717261.1'	0.00	-1.00	25.19	1.00	0.46	0.07	3470504	1.44	7.72	0.97
'CDQK0100000	0.61	0.11	38.22	1.99	0.09	0.03	2451740	0.58	3.44	0.98
'CWKC010096	0.00	0.15	104.68	23.90	0.30	0.14	135613	0.17	3.46	0.92
'CWKB010092	0.28	0.13	54.30	6.65	0.18	0.07	229288	0.11	3.74	0.95
'LGRX0100000	0.00	-1.00	16.80	1.17	0.56	0.12	211102	3.16	10.13	0.96
'LGKQ0100000	0.44	0.17	42.19	5.05	0.13	0.07	247786	0.12	3.65	0.94
'CM003382.1'	0.19	0.12	44.74	2.73	0.27	0.11	2931404	0.03	4.02	0.97
'LEOU0100001	0.42	0.18	40.91	5.65	0.14	0.07	220212	4.98	3.78	0.94
'LBNR0100000	0.00	-1.00	176.16	22.92	0.04	0.01	5319023	0.04	4.84	0.74
'LFLW0100030	0.78	0.32	12.40	1.96	0.15	0.10	80041	6.51	7.53	0.95
'LGHX0100220	0.00	0.07	261.29	20.70	0.23	0.07	3277563	0.60	1.54	0.94
'LGHW010022	0.00	-1.00	258.79	9.92	0.23	0.02	3296881	0.58	1.55	0.94
'KQ412180.1'	0.00	-1.00	>1000	>1000	0.04	0.01	932855	0.53	2.98	0.73
'KQ414666.1'	0.00	0.16	80.94	7.01	0.12	0.07	9480515	3.78	5.32	0.93
'LAVV0101349	0.00	-1.00	>1000	858.83	0.14	0.02	159699	25.11	1.89	0.82
'LGIB01000001	0.22	0.15	32.07	2.46	0.28	0.13	1629114	0.32	5.32	0.97
'APLD01S0000	0.00	-1.00	235.70	4.23	0.18	0.01	81761211	6.46	1.67	0.96
'CP012342.1'	0.33	0.25	12.59	1.09	0.38	0.24	2563723	0.08	9.73	0.98
'LFFV0100021	0.39	0.17	17.15	1.42	0.25	0.10	449315	0.04	8.39	0.98
'LFFW0100002	0.00	-1.00	18.14	0.67	0.58	0.09	1667054	0.00	9.57	0.98
'LFFX0100005	0.30	0.17	18.58	1.58	0.30	0.13	494146	0.00	8.25	0.98
'LFFY0100002	0.32	0.18	16.38	1.29	0.31	0.14	900065	0.00	8.73	0.98
'LFGA0100052	0.25	0.21	19.48	2.18	0.33	0.17	282790	0.00	8.17	0.97
'LFCY0100034	0.25	0.21	19.50	2.22	0.33	0.17	282833	0.00	8.17	0.97
'LFCZ0100006	0.46	0.14	15.90	0.90	0.24	0.08	1035374	0.00	8.28	0.99
'LFDA0100006	0.53	0.12	15.24	0.72	0.21	0.06	1036329	0.01	8.28	0.99
'LFDB0100004	0.18	0.28	12.04	1.68	0.49	0.28	164742	0.08	11.95	0.98
'LFGE0100007	0.64	0.13	16.56	0.85	0.15	0.05	924257	0.01	7.12	0.99
'LFGF0100004	0.45	0.14	18.40	1.03	0.22	0.08	1575439	0.00	7.11	0.99
'LFDC0100001	0.61	0.15	16.41	0.95	0.17	0.07	1232180	0.01	7.22	0.99
'LFDD0100256	0.16	0.29	13.64	2.13	0.48	0.29	103598	0.01	11.15	0.97
'LFGG0100312	0.20	0.30	15.08	2.50	0.42	0.27	118168	0.07	10.04	0.96
'LFCX0100003	0.80	0.12	16.91	0.75	0.11	0.04	3070003	0.00	6.16	0.99
'LFGH0100165	0.14	0.28	13.59	2.02	0.50	0.29	110943	0.10	11.32	0.97
'LFFZ0100046	0.35	0.22	20.05	2.53	0.26	0.14	190820	0.00	7.48	0.96
'LFGK0100034	0.30	0.31	12.28	1.91	0.39	0.26	96793	0.03	10.58	0.97
'LFGB0100001	0.12	0.18	21.23	1.86	0.42	0.20	1423774	0.01	7.96	0.98
'LFGC0100061	0.22	0.24	15.45	2.18	0.38	0.20	110489	0.00	10.14	0.97
'LFGD0100008	0.19	0.17	17.12	1.36	0.40	0.17	710405	0.01	9.11	0.98
'LFGL0100011	0.23	0.29	14.70	2.34	0.40	0.25	118100	0.01	10.06	0.96
'LFGI01002187	0.07	0.34	14.86	2.97	0.54	0.39	91336	0.00	10.94	0.96
'LFGJ0100107	0.22	0.30	14.90	2.52	0.41	0.26	91049	0.01	9.96	0.96
'JTDY0100004	0.01	0.15	67.93	8.83	0.35	0.16	496493	0.82	3.87	0.94
'LDAR01S0000	0.24	0.05	151.87	5.64	0.13	0.03	11189062	10.01	1.45	0.97
'CP012365.1'	0.29	0.14	30.27	2.02	0.25	0.11	2397029	0.18	5.02	0.97
'CVQH0100000	0.10	0.18	23.81	2.10	0.42	0.21	1404863	7.09	6.92	0.97
'CVQI0100000	0.32	0.16	20.44	1.57	0.27	0.12	988237	0.99	7.25	0.98
'LHQP0100437	0.00	-1.00	280.41	22.70	0.25	0.02	616426	1.05	1.59	0.91
'KQ434319.1'	0.34	0.11	28.59	1.38	0.22	0.07	2947605	0.10	5.47	0.99
'KQ434324.1'	0.28	0.17	30.09	2.56	0.25	0.12	1330227	0.23	5.40	0.97
'CM003438.1'	0.32	0.05	298.43	11.94	0.08	0.02	11753682	5.10	0.73	0.95
'CM003439.1'	0.23	0.13	29.19	1.72	0.29	0.12	3220852	0.04	5.55	0.98

'KQ434809.1'	0.00	-1.00	63.28	1.58	0.22	0.02	14164310	0.35	5.53	0.97
'LAZP0100000	0.33	0.24	19.56	2.74	0.27	0.16	145105	0.11	7.62	0.96
'CM003440.1'	0.79	0.16	280.97	145.37	0.01	0.01	61247	0.00	1.00	0.47
'CP012094.1'	0.00	-1.00	9.97	0.51	0.38	0.10	3990191	0.35	17.82	0.97
'LCTW020000C	0.37	0.20	18.60	1.72	0.26	0.14	582697	0.02	7.47	0.97
'KQ435302.1'	0.62	0.12	51.70	4.43	0.07	0.03	459264	3.86	2.57	0.95
'KQ435794.1'	0.00	0.17	84.68	8.04	0.07	0.05	12087087	1.75	6.71	0.91
'CP012523.1'	0.00	-1.00	107.51	2.49	0.22	0.02	33300182	16.25	2.71	0.96
'CP012519.1'	0.32	0.10	52.28	2.56	0.17	0.06	5226878	0.00	3.22	0.98
'LHTS0100001	0.60	0.20	15.59	1.47	0.18	0.08	297062	0.00	7.57	0.98
'KQ438824.1'	0.60	0.20	15.62	1.46	0.18	0.08	297190	0.01	7.57	0.98
'KQ438555.1'	0.60	0.20	15.61	1.49	0.18	0.08	296669	0.02	7.57	0.98
'KQ439017.1'	0.50	0.20	17.01	1.82	0.21	0.10	191450	0.02	7.56	0.97
'LHTV0100002	0.42	0.24	16.47	1.98	0.25	0.14	250532	0.00	8.06	0.97
'LHTX0100001	0.76	0.21	13.49	1.25	0.14	0.07	195669	0.00	7.75	0.98
'LHTW0100002	0.60	0.19	15.57	1.43	0.18	0.08	296632	0.00	7.57	0.98
'LGAV0100000	0.20	0.19	19.77	1.71	0.37	0.19	1489072	0.01	7.99	0.98
'LGAG0100046	0.15	0.09	68.96	3.84	0.24	0.08	3082282	2.37	3.07	0.97
'LGSR0100002	0.00	0.31	170.96	50.49	0.02	0.02	5717297	1.64	7.85	0.70
'LHQQ010000C	0.33	0.17	33.60	3.53	0.20	0.10	391229	0.00	4.89	0.96
'CP012600.1'	0.18	0.11	44.47	2.58	0.27	0.10	4597707	0.01	4.12	0.98
'CP012601.1'	0.60	0.08	50.03	1.94	0.07	0.02	4215291	0.12	2.71	0.98
'CP012602.1'	0.35	0.10	41.18	1.97	0.17	0.06	5265521	0.00	3.88	0.98
'JRWH010004S	0.67	0.10	63.92	4.15	0.05	0.02	1247913	0.70	2.06	0.95
'LAII01000001.	0.54	0.04	365.03	11.80	0.03	0.01	15146312	1.66	0.47	0.95
'LJCY0100010	0.28	0.17	23.42	2.04	0.27	0.12	568959	0.01	6.81	0.98
'LJDH0100019	0.72	0.13	41.16	3.14	0.06	0.02	458397	1.17	2.92	0.96
'CCJE0111824	0.42	0.17	48.60	7.52	0.13	0.06	110069	0.00	3.45	0.92
'CYGV010000C	0.70	0.14	18.88	1.12	0.12	0.05	1159956	2.38	5.86	0.98
'JXCE0100000	0.60	0.13	25.45	1.64	0.12	0.04	829859	0.01	5.07	0.98
'CM003473.1'	0.28	0.03	272.91	4.15	0.09	0.01	228936702	0.98	0.88	0.99
'LIDP01000001	0.09	0.03	352.64	6.00	0.18	0.03	315288781	24.75	0.72	0.98
'CP011455.1'	0.35	0.21	8.49	0.53	0.48	0.22	4363945	0.12	12.72	0.99
'LGTL0100000	0.00	0.15	23.80	1.66	0.49	0.20	2995728	0.36	8.56	0.98
'CP011946.1'	0.00	-1.00	11.68	0.52	0.68	0.15	4994426	1.04	11.91	0.97
'LIHB01000001	0.75	0.15	15.54	0.88	0.13	0.05	1387526	0.24	6.74	0.99
'CM004383.1'	0.50	0.15	26.20	1.70	0.16	0.07	1898896	0.00	4.91	0.97
'KQ459606.1'	0.00	0.11	88.67	5.13	0.26	0.11	13804215	0.17	3.40	0.96
'KQ461108.1'	0.00	-1.00	85.09	2.40	0.33	0.03	6905436	0.53	3.16	0.96
'CM003540.1'	0.77	0.11	40.36	2.09	0.05	0.02	1514906	0.00	2.86	0.97
'CP012036.1'	0.21	0.09	58.23	2.70	0.22	0.07	7094556	0.01	3.37	0.98
'CM003583.1'	0.71	0.11	37.52	2.17	0.07	0.02	1274804	1.57	3.21	0.97
'LJSK0100000	0.20	0.20	22.07	2.45	0.34	0.17	326845	0.22	7.45	0.97
'LIYB01000001	0.00	-1.00	19.30	0.91	0.51	0.11	3589266	0.12	8.88	0.96
'LJJJ01000001	0.60	0.19	11.91	0.79	0.22	0.10	1776945	0.00	9.57	0.99
'CP012609.1'	0.00	-1.00	112.28	1.73	0.19	0.01	44526634	10.81	3.59	0.98
'LHCL0100000	0.35	0.14	29.37	1.93	0.21	0.09	1547020	0.00	5.22	0.98
'LKHI01000010	0.36	0.09	157.59	13.45	0.09	0.03	1295931	0.90	1.26	0.92
'LKCJ0100001	0.35	0.09	155.05	13.89	0.09	0.03	1288507	0.70	1.27	0.91
'LKHJ0100001	0.36	0.09	156.55	13.13	0.09	0.03	1281265	0.94	1.26	0.92
'LKHM0100001	0.32	0.10	162.65	15.67	0.10	0.04	1283549	0.51	1.26	0.90
'LKHN0100001	0.33	0.10	159.71	15.08	0.10	0.04	1284088	1.14	1.26	0.91
'LKHK0200001	0.31	0.10	163.83	15.79	0.10	0.04	1277761	0.31	1.27	0.90
'LKHL0100001	0.32	0.08	194.14	17.94	0.09	0.03	1258884	1.34	1.12	0.91
'LDPK0100018	0.00	-1.00	452.43	124.83	0.01	0.00	5508434	0.34	6.57	0.62
'LDPL0100004	0.00	-1.00	537.45	164.57	0.01	0.00	4044527	0.73	6.48	0.64
'JREZ0100000	0.00	0.05	206.53	7.92	0.28	0.07	17059037	1.17	1.62	0.97
'LIPI01003878.	0.14	0.07	327.69	27.67	0.16	0.05	4241357	24.91	0.69	0.92
'LIPJ01003348	0.28	0.07	296.72	27.26	0.09	0.03	2448660	29.92	0.61	0.91
'LIQJ01002592	0.37	0.06	288.12	16.97	0.06	0.02	5982234	27.25	0.52	0.92
'LKCK0100001	0.31	0.10	165.74	15.74	0.10	0.04	1296567	0.28	1.26	0.91
'LKCL0100001	0.34	0.10	158.12	14.62	0.09	0.04	1295191	1.20	1.26	0.91
'LKBE0100002	0.00	-1.00	33.94	0.89	0.45	0.04	3359036	3.46	6.02	0.98
'LJBI01000005	0.40	0.11	30.86	1.51	0.18	0.06	4047401	0.65	4.80	0.98
'LJRW0100003	0.36	0.09	157.70	13.50	0.09	0.03	1279005	0.28	1.26	0.92
'LHSL0100000	0.55	0.16	25.54	2.43	0.13	0.06	291598	0.00	5.24	0.97
'LHTN0100244	0.41	0.17	19.10	1.81	0.23	0.10	194596	0.03	7.47	0.98
'LHTM0100408	0.17	0.24	23.28	3.72	0.35	0.20	189566	0.00	7.61	0.96
'LHTP0100365	0.57	0.25	16.92	2.07	0.18	0.11	188769	0.00	7.18	0.96
'LHTO0100422	0.50	0.21	17.95	1.97	0.20	0.10	195625	0.00	7.38	0.97
'LHTQ0100049	0.49	0.22	17.58	1.97	0.20	0.10	196614	0.02	7.56	0.97
'CP010988.1'	0.15	0.06	153.40	6.91	0.18	0.05	8077450	4.59	1.69	0.97
'KQ468087.1'	0.00	-1.00	49.10	1.82	0.17	0.02	5804718	0.21	7.79	0.96
'KQ465187.1'	0.01	0.10	68.05	4.14	0.36	0.12	3185661	2.36	3.70	0.97
'LGSM010000C	0.39	0.21	20.90	2.38	0.23	0.12	267541	0.00	6.69	0.96
'LGSL0100000	0.58	0.17	18.35	1.55	0.16	0.07	376293	0.00	6.67	0.98
'LGSJ0100000	0.39	0.21	16.32	1.77	0.27	0.13	256829	0.00	8.46	0.98
'LGSN0100000	0.60	0.20	16.66	1.52	0.17	0.08	321636	0.00	7.12	0.97
'LGTU0100000	0.54	0.21	15.43	1.74	0.20	0.10	184729	0.00	8.06	0.97
'LGTR0100000	0.64	0.23	16.38	1.90	0.15	0.08	131575	0.00	6.98	0.96
'LGTS0100000	0.64	0.23	16.35	1.90	0.15	0.08	132400	0.00	6.99	0.96
'LGTT0100000	0.65	0.19	14.51	1.31	0.17	0.07	175689	0.00	7.83	0.98
'LGSK0100000	0.54	0.16	16.41	1.36	0.19	0.08	222465	0.00	7.69	0.98
'LGSO010000C	0.75	0.22	15.40	1.62	0.13	0.07	177598	0.00	6.88	0.97
'LJPC0100000	0.64	0.21	80.31	27.27	0.05	0.03	37851	16.79	2.00	0.76
'CP010059.1'	0.38	0.11	51.21	2.90	0.14	0.05	2438959	1.46	3.07	0.97
'CP010060.1'	0.47	0.11	45.83	2.68	0.12	0.05	2261297	0.22	3.16	0.97
'CP010061.1'	0.45	0.12	46.42	2.84	0.12	0.05	2281990	0.21	3.16	0.97
'CP010062.1'	0.38	0.11	51.02	2.96	0.14	0.05	2437293	1.40	3.06	0.97
'CP010063.1'	0.31	0.12	54.39	3.50	0.17	0.07	2372196	1.15	3.05	0.96
'CP010064.1'	0.10	0.14	65.34	5.53	0.30	0.14	2320599	0.76	3.06	0.95
'KQ474073.1'	0.00	-1.00	14.38	1.32	0.29	0.11	2366775	1.75	13.69	0.91
'CENE0100000	0.00	-1.00	15.85	0.67	0.59	0.11	1860355	0.05	10.15	0.97
'CCBN010000C	0.08	0.10	70.05	4.51	0.29	0.10	2581591	8.10	3.26	0.97
'LN609233.1'	0.49	0.14	17.74	0.97	0.21	0.08	2746506	5.45	6.91	0.99
'LN609196.1'	0.55	0.13	17.94	0.88	0.18	0.06	2885998	17.91	5.71	0.99
'LN881072.1'	0.00	0.14	175.74	58.49	0.27	0.12	101716	6.28	2.53	0.90
'LN877770.1'	0.00	-1.00	87.06	7.30	0.05	0.01	5027063	5.49	6.76	0.83
'HG975440.1'	0.00	-1.00	185.56	2.90	0.27	0.02	109333515	6.98	1.52	0.97
'JRUX0100140	0.39	0.11	767.62	566.73	0.05	0.02	139679	0.00	0.94	0.71
'JRUY0100547	0.48	0.09	>1000	>1000	0.03	0.01	153807	0.03	0.59	0.58
'LIHL01055726	0.03	0.08	157.66	10.85	0.28	0.09	3676996	1.37	1.86	0.95
'LHUT0100000	0.31	0.15	57.81	8.23	0.16	0.07	199390	12.13	2.95	0.93
'LJCH0100002	0.00	-1.00	132.25	5.67	0.24	0.02	1795199	6.22	2.71	0.95
'CP013020.1'	0.13	0.12	46.77	2.88	0.30	0.13	5165891	0.02	4.10	0.97
'KQ476056.1'	0.38	0.10	41.61	1.92	0.16	0.05	6607548	3.10	3.70	0.98

'CP012092.1'	0.69	0.09	92.50	5.28	0.04	0.01	1899676	0.71	1.41	0.95
'CM003560.1'	0.77	0.11	40.03	2.08	0.05	0.02	1522271	0.00	2.86	0.97
'KQ475712.1'	0.29	0.19	27.37	3.51	0.25	0.12	176502	0.01	6.13	0.96
'LLKM0100000	0.72	0.18	18.73	1.71	0.11	0.05	159373	0.00	6.06	0.97
'CP013032.1'	0.56	0.08	90.24	5.14	0.06	0.02	1683224	0.00	1.66	0.95
'CP013036.1'	0.54	0.08	93.95	5.50	0.06	0.02	1711393	0.01	1.64	0.95
'AP014599.1'	0.57	0.11	45.06	2.61	0.09	0.03	1722779	0.00	3.12	0.97
'AP012213.1'	0.56	0.10	45.60	2.42	0.09	0.03	1745387	0.00	3.13	0.97
'LJWW0100000	0.00	-1.00	135.21	7.03	0.22	0.02	2343502	16.35	2.28	0.92
'LMAW0100000	0.25	0.05	198.93	7.81	0.12	0.03	10443977	3.13	1.21	0.97
'LAQG0100045	0.07	0.30	14.59	2.73	0.53	0.34	92272	0.40	11.56	0.97
'KQ481983.1'	0.30	0.14	19.66	1.08	0.30	0.11	3905163	17.02	6.52	0.99
'LJPH0100057	0.00	-1.00	41.87	2.31	0.41	0.06	643678	0.00	5.23	0.95
'LJPJ01000294	0.52	0.14	28.33	2.04	0.14	0.06	836833	0.01	4.77	0.97
'LJPK0100017	0.00	0.18	42.52	5.13	0.43	0.22	564389	0.29	5.11	0.95
'LJPI01000825	0.00	0.17	40.37	4.20	0.43	0.23	979930	0.04	5.24	0.96
'KQ483353.1'	0.41	0.04	224.13	4.86	0.06	0.01	51414391	0.90	0.88	0.98
'CP013116.1'	0.56	0.08	106.90	6.73	0.05	0.02	1565978	0.01	1.44	0.94
'CP013111.1'	0.00	0.24	11.74	0.92	0.76	0.45	6989698	0.05	12.02	0.98
'AP014957.1'	0.00	-1.00	112.92	1.70	0.17	0.01	43270923	0.02	4.23	0.98
'CM003601.1'	0.28	0.24	7.35	0.50	0.59	0.30	8759578	0.01	14.60	0.99
'CM003594.1'	0.13	0.13	39.55	2.49	0.32	0.13	3470205	0.09	4.64	0.98
'CM003596.1'	0.14	0.14	39.32	2.73	0.33	0.15	3256231	0.01	4.57	0.97
'CM003641.1'	0.80	0.09	71.99	4.09	0.03	0.01	1133809	1.47	1.59	0.95
'CP013140.1'	0.00	-1.00	9.26	0.37	0.63	0.14	6157384	0.13	15.85	0.98
'JANR0100000	0.00	-1.00	140.07	7.37	0.07	0.01	6352280	8.04	4.82	0.89
'LMZO0100000	0.03	0.11	301.90	68.36	0.23	0.09	530254	0.00	1.46	0.87
'KQ503367.1'	0.84	0.09	>1000	>1000	0.02	0.00	32052	2.29	0.52	0.26
'KQ545315.1'	0.24	0.08	159.26	12.90	0.13	0.04	1881953	8.03	1.40	0.94
'CP013147.1'	0.35	0.11	42.98	2.27	0.17	0.07	4339653	0.68	3.69	0.98
'KQ550984.1'	0.00	-1.00	191.58	9.25	0.25	0.02	1563227	12.47	1.80	0.94
'KQ556858.1'	0.01	0.04	175.69	3.96	0.27	0.05	80077792	5.02	1.93	0.99
'LLWD0100012	0.00	-1.00	333.86	7.60	0.24	0.01	13046067	1.34	1.14	0.96
'KQ560120.1'	0.17	0.11	59.42	4.46	0.24	0.09	1305381	6.79	3.28	0.96
'KQ557225.1'	0.09	0.04	168.57	4.15	0.21	0.04	55650406	3.19	1.76	0.98
'DF967598.1'	0.00	-1.00	38.03	1.59	0.35	0.05	1791675	2.19	6.25	0.96
'LMTP0100104	0.00	-1.00	249.97	8.75	0.27	0.02	4064336	6.28	1.32	0.95
'LJJS01001513	0.44	0.16	20.29	1.67	0.21	0.09	463151	0.05	6.82	0.98
'KQ728331.1'	0.47	0.04	292.49	9.87	0.04	0.01	11828398	0.63	0.66	0.96
'JYDJ0100000	0.03	0.11	134.72	19.97	0.27	0.11	489069	0.15	2.45	0.93
'JYDK0100000	0.10	0.10	112.55	11.54	0.23	0.08	887815	0.13	2.52	0.95
'JYDU0100000	0.12	0.09	126.71	12.10	0.21	0.07	847571	0.36	2.23	0.95
'JYDL0100000	0.25	0.11	83.37	6.79	0.16	0.06	1324161	0.03	2.56	0.95
'JYDN0100000	0.05	0.09	109.25	8.86	0.27	0.09	1490669	0.27	2.70	0.96
'LDAU0100011	0.61	0.07	268.98	19.56	0.02	0.01	1988609	0.00	0.63	0.89
'JYDW0100000	0.05	0.11	128.50	15.59	0.26	0.10	699576	0.02	2.44	0.93
'JYDT0100000	0.05	0.09	136.81	13.37	0.25	0.08	1063082	0.10	2.28	0.95
'JYDI01000001	0.01	0.10	135.09	15.45	0.29	0.11	851249	0.14	2.42	0.94
'JYDH0100000	0.30	0.11	71.37	5.70	0.15	0.06	1060796	0.13	2.79	0.95
'JYDV0100000	0.10	0.09	117.20	10.26	0.23	0.08	1320437	0.14	2.42	0.95
'JYDQ0100000	0.04	0.09	121.97	12.31	0.27	0.09	848941	0.08	2.59	0.95
'JYDP0100000	0.10	0.10	115.59	10.42	0.23	0.08	1221358	0.06	2.42	0.95
'JYDR0100000	0.07	0.09	127.25	10.38	0.24	0.08	1954441	0.26	2.27	0.95
'JYDS0100000	0.00	-1.00	155.37	9.21	0.27	0.02	673645	0.00	2.38	0.94
'JYDM0100000	0.04	0.09	123.31	11.63	0.27	0.09	1100865	0.03	2.47	0.95
'JYDO0100000	0.15	0.09	122.78	10.92	0.19	0.07	1190084	0.00	2.17	0.95
'KQ748387.1'	0.02	0.07	156.85	9.31	0.28	0.08	4762499	1.48	1.91	0.96
'LMYF0100000	1.00	0.12	27.46	1.40	0.04	0.01	1534183	0.00	3.58	0.98
'CP013328.1'	0.23	0.05	>1000	>1000	0.05	0.01	2469901	0.02	0.26	0.73
'LDMZ0101262	0.60	0.17	131.39	47.49	0.04	0.02	50567	0.00	1.74	0.74
'LDNA0106528	0.52	0.20	529.97	>1000	0.05	0.03	19561	0.00	1.72	0.56
'CP013048.1'	0.00	-1.00	30.80	1.19	0.32	0.05	4689408	0.00	7.43	0.96
'LDEF0100000	0.04	0.17	22.71	1.93	0.47	0.21	1075689	0.04	8.31	0.98
'JXRH0101041	0.90	0.29	708.66	>1000	0.03	0.02	6226	28.99	1.37	0.09
'LKTN0109983	0.06	0.13	499.08	408.83	0.19	0.08	92457	10.00	1.75	0.86
'LAEG0100050	0.48	0.26	15.62	2.13	0.23	0.13	119718	0.00	8.17	0.96
'LNDK0200006	0.00	-1.00	28.40	0.81	0.45	0.05	6660791	0.03	7.18	0.98
'LKER0100000	0.00	-1.00	15.16	0.92	0.47	0.12	1779505	0.12	10.96	0.95
'CVKU0100000	0.00	0.24	217.50	61.11	0.02	0.02	3403828	0.57	6.71	0.71
'CVKY0100000	0.00	0.31	271.07	113.28	0.02	0.02	3243340	0.91	6.98	0.65
'CVKW0100000	0.00	0.34	164.34	55.61	0.03	0.03	3168126	0.62	7.09	0.69
'CVKZ0100000	0.00	0.30	97.35	21.63	0.04	0.05	3258266	0.52	7.07	0.78
'CVKV0100000	0.00	-1.00	178.67	25.84	0.02	0.01	3320137	0.59	6.82	0.72
'CVKX0100000	0.00	0.33	203.53	74.89	0.02	0.03	3205512	0.96	7.00	0.67
'CZLE0100009	0.38	0.05	227.14	8.25	0.07	0.01	9022850	0.10	0.92	0.96
'LN899469.1'	0.68	0.13	17.29	0.82	0.14	0.05	2740121	0.00	6.38	0.99
'CZRN0100000	0.07	0.03	454.07	10.75	0.18	0.03	90267253	3.18	0.79	0.97
'JRLE0100000	0.00	-1.00	101.51	2.99	0.33	0.03	5349618	18.50	2.25	0.96
'LN906597.1'	0.38	0.10	70.63	4.26	0.12	0.04	2149384	0.17	2.39	0.96
'CM003626.1'	0.00	-1.00	10.23	0.47	0.42	0.10	3967671	0.00	17.33	0.98
'KQ758850.1'	0.00	-1.00	18.38	0.61	0.32	0.05	19720504	13.93	10.21	0.98
'CP012043.1'	0.00	0.24	22.99	2.30	0.33	0.23	5491870	0.00	9.41	0.96
'LD344849.1'	0.00	-1.00	148.45	5.85	0.27	0.02	1959435	7.82	2.20	0.95
'CP013655.1'	0.41	0.09	52.22	2.55	0.13	0.04	3746111	4.35	2.89	0.98
'LN609383.1'	0.11	0.05	124.14	2.86	0.22	0.04	98476147	36.89	1.39	0.99
'LNKV0100000	0.46	0.13	31.31	1.87	0.15	0.06	1993158	0.01	4.57	0.98
'LNGN0100000	0.31	0.14	36.02	2.60	0.21	0.09	1672908	0.43	4.51	0.97
'LMBU0100000	0.00	-1.00	91.09	2.19	0.29	0.02	7126315	2.13	3.33	0.97
'LDJA0100000	0.58	0.09	43.90	1.94	0.09	0.03	4213555	1.21	3.13	0.98
'LLWO0100003	0.57	0.11	59.15	4.31	0.07	0.03	847663	0.02	2.45	0.95
'LMAA0100005	0.66	0.10	55.01	3.44	0.06	0.02	749632	0.04	2.45	0.96
'LMAB0100008	0.57	0.11	60.50	4.45	0.07	0.03	675315	0.07	2.40	0.95
'LLZY0100003	0.62	0.11	57.39	4.19	0.06	0.02	686417	0.07	2.42	0.95
'LLZZ0100010	0.64	0.10	57.68	4.01	0.06	0.02	585387	0.20	2.40	0.96
'LMAY0100011	0.58	0.10	60.84	4.28	0.07	0.02	693622	0.05	2.39	0.96
'LATX0100198	0.55	0.20	23.33	2.32	0.15	0.08	559288	0.26	5.23	0.96
'LOCP0200000	0.40	0.05	286.16	10.96	0.06	0.01	8996366	0.00	0.71	0.96
'CP012350.1'	0.59	0.22	10.93	0.78	0.24	0.13	2530708	0.00	9.60	0.98
'CP012353.1'	0.62	0.20	10.70	0.70	0.23	0.11	2470017	0.00	9.61	0.99
'CP014258.1'	0.41	0.23	7.90	0.56	0.46	0.23	6257075	5.70	12.22	0.99
'LNZH0200021	0.00	-1.00	55.30	4.95	0.17	0.04	1376240	0.03	5.79	0.86
'CP013693.1'	0.68	0.19	10.47	0.64	0.21	0.09	2399738	0.04	9.59	0.99
'JPDN0100000	0.16	0.17	35.57	3.38	0.31	0.16	1198811	0.47	5.00	0.96
'LNFP0100011	0.53	0.21	19.13	1.97	0.18	0.09	318328	0.00	6.63	0.97
'LNFO0100561	0.46	0.20	20.42	2.23	0.20	0.10	203438	0.10	6.58	0.97
'LKPD0100810	0.00	0.10	469.93	164.77	0.23	0.09	386188	2.55	1.31	0.86

'LCZG0100000	0.52	0.13	27.67	1.71	0.14	0.05	1009776	0.14	4.94	0.98
'LOQJ0100082	0.68	0.13	38.07	2.69	0.07	0.03	637318	0.21	3.28	0.96
'LOQK0100000	0.00	-1.00	5.95	0.28	1.04	0.24	1297082	0.04	18.84	0.99
'JTAI01000007	0.29	0.09	205.72	20.31	0.10	0.04	1279890	0.00	1.12	0.90
'LOQL0100720	0.35	0.04	295.27	7.01	0.07	0.01	45446104	0.45	0.73	0.97
'LOQM010024	0.34	0.03	279.84	4.95	0.07	0.01	106486165	0.47	0.80	0.98
'LPXP0100007	0.38	0.04	287.82	6.82	0.06	0.01	39801822	4.13	0.70	0.97
'LOEK0100037	0.43	0.03	275.61	4.82	0.05	0.01	94987922	0.43	0.72	0.98
'KQ760267.1'	0.00	-1.00	52.49	3.57	0.08	0.02	4677300	1.43	8.48	0.89
'LPVI01001605	0.31	0.19	21.91	2.41	0.26	0.13	292599	0.09	7.10	0.97
'LQCJ0100000	0.11	0.08	>1000	>1000	0.13	0.04	485854	2.12	0.76	0.81
'LNCG0124964	0.67	0.14	27.30	1.93	0.10	0.04	476466	0.00	4.41	0.97
'FAPD0100028	0.33	0.18	>1000	>1000	0.09	0.05	17087	2.76	2.00	0.68
'FAPB0100022	0.00	-1.00	>1000	>1000	0.15	0.02	25773	0.00	4.26	0.84
'FAPA0100073	0.34	0.19	>1000	>1000	0.08	0.05	17583	3.97	1.95	0.67
'FAPC0100068	0.38	0.19	>1000	>1000	0.08	0.04	15000	1.60	1.97	0.65
'FAPI01001401	0.13	0.21	183.31	155.10	0.19	0.11	24578	0.12	3.21	0.83
'FARF0100001	0.93	0.18	>1000	>1000	0.02	0.01	14449	0.53	0.62	0.12
'FAPL0100361	0.00	-1.00	354.61	153.14	0.21	0.03	55963	0.07	2.92	0.85
'FAPO0100899	0.00	-1.00	504.90	245.20	0.19	0.02	60436	0.45	2.77	0.86
'FAPE0100014	0.15	0.16	>1000	>1000	0.13	0.06	34946	0.25	2.02	0.77
'FAQM0100000	0.97	0.17	>1000	>1000	0.02	0.01	13211	0.00	0.60	0.07
'FAPR0100886	0.00	-1.00	304.63	113.74	0.21	0.03	52506	0.17	3.25	0.86
'FAQR0100080	0.14	0.17	>1000	>1000	0.15	0.07	21431	0.96	2.38	0.81
'FARD0100015	0.00	-1.00	>1000	>1000	0.23	0.04	22321	2.19	3.07	0.79
'FAPP0100100	0.53	0.17	36.23	3.83	0.11	0.05	293406	0.00	3.88	0.95
'FAPJ0101084	0.38	0.17	84.18	19.57	0.11	0.05	80728	0.08	2.58	0.87
'FAQN0100003	0.25	0.18	>1000	>1000	0.11	0.06	18473	0.00	2.14	0.73
'FARH0100030	0.00	0.22	643.26	>1000	0.23	0.14	23298	0.04	3.27	0.81
'FAQQ0100000	0.07	0.20	>1000	>1000	0.18	0.10	17497	1.63	2.90	0.81
'FAPG0102625	0.00	0.16	375.12	417.58	0.23	0.11	46279	0.21	2.79	0.86
'FAPH0100011	1.01	0.14	848.64	>1000	0.02	0.01	13536	0.65	0.60	0.16
'FAPK0100326	0.48	0.17	38.65	4.97	0.12	0.06	160780	0.00	3.95	0.94
'FAPF0100027	0.34	0.21	91.39	42.10	0.12	0.07	24004	1.56	3.11	0.83
'FARE0100008	0.22	0.18	>1000	>1000	0.12	0.06	19644	0.09	2.18	0.75
'FATR0100000	0.66	0.13	395.67	205.27	0.02	0.01	81121	0.16	0.96	0.58
'FATD0100053	0.08	0.24	237.57	337.02	0.22	0.13	19228	4.87	3.53	0.82
'FASU0100351	0.00	-1.00	180.51	31.92	0.26	0.03	77652	0.51	2.87	0.90
'FASN0100003	0.90	0.14	>1000	>1000	0.03	0.01	13249	1.12	0.60	0.16
'FATF0100044	0.21	0.22	>1000	>1000	0.13	0.08	14089	0.13	2.70	0.73
'FASR0100000	0.14	0.25	130.91	108.34	0.19	0.12	24300	1.64	3.52	0.81
'FATN0100002	0.25	0.20	>1000	>1000	0.11	0.06	17875	0.87	2.20	0.72
'FASX0100426	0.32	0.15	68.77	12.73	0.14	0.06	94221	0.08	3.23	0.92
'FATU0100015	0.00	-1.00	149.60	44.12	0.26	0.04	36761	0.00	3.94	0.86
'FATX0100003	0.22	0.18	>1000	>1000	0.12	0.06	21113	0.30	2.19	0.74
'FATW0100014	0.11	0.25	>1000	>1000	0.15	0.10	15982	0.48	2.95	0.71
'FATP0100069	0.39	0.17	>1000	>1000	0.07	0.03	18642	0.93	1.71	0.67
'FATM0100055	0.45	0.17	>1000	>1000	0.06	0.03	18118	1.23	1.59	0.62
'FATE0100030	0.05	0.18	>1000	>1000	0.20	0.10	19278	0.08	2.75	0.82
'FATK0100083	0.12	0.21	>1000	>1000	0.15	0.09	17389	0.25	2.60	0.76
'FATS0100046	0.24	0.23	90.23	47.07	0.16	0.10	28427	0.10	3.51	0.84
'FATJ0100003	0.05	0.19	>1000	>1000	0.18	0.10	22787	0.00	2.54	0.79
'FATB0100004	1.07	0.14	568.10	575.48	0.01	0.01	13994	0.00	0.69	0.10
'FASW010046	0.00	0.18	97.02	35.76	0.30	0.15	51133	0.22	4.12	0.90
'FATL0100019	0.37	0.17	>1000	>1000	0.08	0.04	16104	0.32	1.92	0.71
'FATQ0100119	0.04	0.20	>1000	>1000	0.20	0.11	20961	1.92	2.68	0.80
'FATO0100038	0.00	-1.00	>1000	>1000	0.23	0.04	16171	3.10	3.57	0.83
'FASM0100048	0.14	0.22	>1000	>1000	0.16	0.09	11505	0.60	2.91	0.77
'FASP0100020	0.00	-1.00	320.13	242.29	0.18	0.03	19093	1.68	5.00	0.85
'FASV0101345	0.08	0.19	106.80	42.27	0.24	0.12	52172	0.19	3.61	0.89
'LN930111.1'	0.03	0.06	186.89	7.26	0.27	0.07	17657826	1.30	1.61	0.97
'LN606820.1'	0.00	-1.00	11.91	0.51	0.63	0.14	5247934	0.20	12.16	0.97
'LN590509.1'	0.00	-1.00	12.09	0.53	0.62	0.14	5336135	0.29	12.10	0.97
'LN609302.1'	0.30	0.18	18.72	1.37	0.32	0.16	2664884	0.02	7.18	0.98
'LOQP0100812	0.27	0.09	324.25	70.55	0.09	0.03	356420	0.15	1.06	0.85
'LOQO010069	0.40	0.03	292.18	5.27	0.06	0.01	95295052	0.13	0.71	0.98
'KQ947404.1'	0.48	0.11	29.13	1.47	0.15	0.05	2762476	0.38	4.72	0.98
'LKUB0115900	0.46	0.20	39.35	7.11	0.13	0.07	73304	0.00	4.12	0.92
'LNGL0100000	0.00	-1.00	34.40	1.12	0.41	0.05	2610973	0.66	6.64	0.98
'LNGK0100000	0.00	-1.00	36.70	1.05	0.36	0.04	5637848	0.09	6.56	0.98
'FAPM0100003	0.00	-1.00	27.13	5.27	0.41	0.09	9738	1.27	10.10	0.92
'FARO0100091	1.16	0.30	6.47	0.64	0.14	0.08	168051	0.28	11.38	0.98
'CZLF0100028	0.63	0.13	18.81	1.02	0.14	0.05	1386477	0.00	6.21	0.99
'CDMC0100000	0.00	-1.00	78.28	7.42	0.07	0.02	4695267	0.90	5.98	0.81
'LN998033.1'	0.00	0.28	16.49	1.93	0.55	0.39	2177010	0.02	9.79	0.97
'LNBV0102194	0.35	0.17	41.97	5.72	0.16	0.08	146289	0.13	4.24	0.94
'LJZU0100003	0.25	0.14	44.46	4.28	0.21	0.09	489393	0.00	4.27	0.96
'LJOA0100007	0.47	0.13	30.92	2.07	0.15	0.05	726305	0.02	4.73	0.98
'LJDD0100163	0.33	0.21	19.98	2.00	0.28	0.15	478393	0.02	7.25	0.97
'LJGR0100000	0.00	-1.00	451.54	97.92	0.02	0.00	5220104	0.03	4.77	0.66
'LKBB0100000	0.02	0.17	45.02	5.79	0.37	0.19	390132	0.32	5.31	0.95
'LLKO0100000	0.00	0.15	26.11	1.86	0.49	0.21	2664519	0.05	7.70	0.98
'LLXE0100000	0.40	0.17	30.38	3.05	0.18	0.08	325200	0.00	5.09	0.96
'CM003662.1'	0.00	-1.00	113.80	1.89	0.19	0.01	46202548	4.08	3.73	0.98
'BCMY0100000	0.00	-1.00	39.85	1.10	0.43	0.04	3905891	0.00	5.44	0.98
'LAVE01S0131	0.00	0.07	202.75	11.80	0.25	0.07	7153784	4.41	1.78	0.96
'LAVF01S0052	0.04	0.06	194.20	11.75	0.24	0.07	5035720	5.71	1.59	0.96
'LCYQ01S000	0.07	0.06	180.52	8.29	0.23	0.06	9040279	5.59	1.62	0.97
'LPNX0101601	0.05	0.07	230.30	15.84	0.24	0.07	3893220	0.91	1.33	0.95
'LOQN0100000	0.31	0.07	698.37	115.88	0.06	0.02	1380479	0.03	0.52	0.83
'CM003679.1'	0.00	-1.00	231.98	3.66	0.26	0.01	50710336	19.81	1.29	0.97
'CP013656.1'	0.34	0.17	19.81	1.27	0.28	0.13	4464611	0.06	6.88	0.98
'CP013962.1'	0.00	-1.00	31.23	1.12	0.35	0.05	5610558	0.20	7.16	0.97
'LPZQ0100023	0.31	0.13	32.07	1.86	0.22	0.09	3317018	0.50	4.94	0.98
'CM003710.1'	0.46	0.03	374.70	5.95	0.04	0.01	150265477	0.81	0.56	0.98
'CM003683.1'	0.17	0.03	284.39	4.58	0.14	0.02	248231514	11.54	0.89	0.99
'LD636966.1'	0.00	0.06	281.40	17.07	0.26	0.07	6916597	3.12	1.30	0.95
'CM003813.1'	0.00	-1.00	152.28	3.07	0.17	0.01	24880688	1.53	3.09	0.97
'LPUV0100053	0.39	0.03	277.54	5.47	0.06	0.01	83598005	2.14	0.75	0.98
'LPUU0100328	0.00	-1.00	146.71	8.27	0.25	0.02	1114742	0.70	2.51	0.93
'KQ954464.1'	0.29	0.23	11.59	1.01	0.42	0.22	1017535	0.00	11.16	0.98
'LNKU0100000	0.51	0.18	9.53	0.57	0.32	0.13	2201887	0.88	11.06	0.99
'LNQQ0100000	0.44	0.23	10.00	0.78	0.36	0.19	1786154	0.46	11.02	0.99
'CM003707.1'	0.00	-1.00	31.21	1.12	0.32	0.05	5019692	0.05	7.41	0.97
'CP014135.1'	0.00	-1.00	18.39	0.67	0.50	0.09	5502003	0.17	9.21	0.97
'CP014165.1'	0.61	0.12	31.28	1.53	0.10	0.04	3745118	6.29	3.68	0.98

'JXTI01000001	0.77	0.18	27.38	2.99	0.07	0.04	184369	0.19	4.10	0.94
'LNQF0100000	0.77	0.14	40.09	3.09	0.05	0.02	641080	0.00	2.87	0.95
'LNJS0100000	0.00	-1.00	9.69	0.43	0.46	0.10	3581356	0.00	17.56	0.98
'CM003771.1'	0.00	-1.00	8.93	0.38	0.61	0.13	3496452	0.00	17.01	0.98
'CM003768.1'	0.00	-1.00	9.34	0.42	0.53	0.12	3550762	0.00	16.87	0.98
'CP014242.1'	0.67	0.13	37.30	2.25	0.07	0.03	2061902	0.00	3.31	0.97
'LOFC0100003	0.42	0.16	26.09	2.28	0.18	0.08	501659	0.15	5.79	0.97
'LOFD0100007	0.29	0.18	27.46	2.91	0.25	0.12	472660	0.06	6.19	0.96
'LOFB0100007	0.24	0.17	30.14	3.33	0.26	0.12	349171	0.01	5.88	0.96
'LOEM0100032	0.37	0.13	27.72	2.05	0.20	0.08	501225	0.05	5.76	0.98
'LOFE0100002	0.48	0.14	23.98	1.51	0.17	0.06	948369	0.10	5.93	0.98
'LOFF0100021	0.33	0.15	26.93	2.13	0.23	0.09	585182	0.05	5.99	0.98
'LAQH0100510	0.71	0.10	57.20	3.79	0.05	0.02	689533	0.00	2.24	0.96
'LSAY0100000	0.00	-1.00	38.25	2.14	0.31	0.06	3506016	0.16	5.87	0.93
'LSDU0100002	0.00	0.19	93.20	11.53	0.11	0.08	5849119	0.74	4.47	0.87
'CP014280.1'	0.00	0.22	23.28	2.09	0.34	0.22	4801156	0.05	9.11	0.97
'JEMN0100049	0.56	0.16	21.22	1.63	0.15	0.06	494648	0.00	6.04	0.98
'JFFI01001038	0.62	0.19	19.90	2.02	0.14	0.06	217493	0.04	6.20	0.97
'JFBX0100051	0.44	0.16	22.13	1.65	0.20	0.09	802711	0.06	6.40	0.98
'CP014092.1'	0.00	-1.00	33.41	1.22	0.29	0.04	5363962	0.00	7.28	0.97
'KQ962167.1'	0.00	-1.00	42.74	3.17	0.37	0.07	856993	5.26	4.78	0.91
'CP014347.1'	0.00	-1.00	11.90	0.55	0.62	0.14	5036843	0.02	12.18	0.97
'KQ964245.1'	0.29	0.15	19.22	1.10	0.31	0.13	4328376	0.01	7.53	0.99
'KQ964418.1'	0.48	0.10	137.61	16.14	0.06	0.02	546290	15.37	1.15	0.89
'KQ965521.1'	0.28	0.12	30.35	1.62	0.25	0.10	4130736	0.23	5.19	0.98
'CP014354.1'	0.37	0.13	26.44	1.61	0.21	0.08	1976175	0.70	5.63	0.98
'LSAJ0100000	0.61	0.20	14.56	1.21	0.18	0.09	588145	0.00	8.01	0.98
'KQ965731.1'	0.00	-1.00	78.70	9.62	0.08	0.02	1572201	0.31	6.66	0.80
'CP013015.1'	0.45	0.08	113.12	6.78	0.08	0.02	2540211	0.49	1.49	0.95
'CM003781.1'	0.42	0.03	253.81	3.74	0.05	0.01	175656249	0.48	0.78	0.99
'LFZO0100000	0.45	0.24	21.33	2.67	0.20	0.12	283715	0.00	6.23	0.95
'LFZN0100000	0.67	0.17	18.13	1.27	0.14	0.06	849494	0.03	6.23	0.98
'CP014210.1'	0.06	0.28	10.74	1.02	0.72	0.47	6537412	32.30	8.29	0.98
'LSTP0100000	0.54	0.10	41.08	1.89	0.10	0.03	3556246	0.94	3.44	0.98
'LSVH0100000	0.64	0.16	24.67	2.13	0.11	0.05	388898	0.01	5.02	0.97
'LRSR0101412	0.25	0.16	36.79	4.36	0.23	0.11	283682	0.00	5.01	0.96
'CP014480.1'	0.29	0.30	7.54	0.70	0.58	0.36	3742169	0.00	14.30	0.99
'CP013251.1'	0.36	0.13	29.06	1.57	0.21	0.09	5430256	0.20	4.85	0.98
'LJIH01000001	0.22	0.07	159.83	8.53	0.14	0.04	4418976	1.70	1.49	0.96
'LSYV0100000	0.34	0.13	14.60	0.67	0.32	0.11	6378020	14.94	7.99	0.99
'CP010968.1'	0.37	0.21	8.42	0.53	0.47	0.22	4433130	3.05	12.33	0.99
'CP010996.1'	0.45	0.19	7.59	0.39	0.44	0.18	5938797	0.00	13.47	0.99
'LQNK0100050	0.00	0.11	83.06	6.47	0.36	0.15	2218376	1.12	3.05	0.96
'CP011233.1'	0.00	-1.00	28.37	1.15	0.29	0.05	4882689	0.65	8.41	0.96
'LQNS0100468	0.52	0.13	39.14	2.65	0.11	0.04	1285385	12.24	3.18	0.97
'CP014645.1'	0.36	0.17	22.53	1.56	0.25	0.12	2903890	0.11	6.03	0.98
'JPDO0100000	0.52	0.19	21.41	2.30	0.17	0.08	215673	0.00	6.14	0.97
'CP014735.1'	0.79	0.11	39.74	2.05	0.05	0.02	1507119	1.25	2.80	0.97
'LJAO0100000	0.86	0.11	16.69	0.60	0.09	0.03	4887340	0.00	5.94	0.99
'LLXH0100001	0.00	-1.00	291.90	40.09	0.25	0.02	294061	1.14	1.77	0.88
'LLXK0100008	0.26	0.12	127.97	18.71	0.13	0.05	301976	2.21	1.88	0.91
'LLXI01000001	0.26	0.13	116.09	17.54	0.14	0.06	317905	1.09	1.92	0.90
'LLXL0100005	0.22	0.13	119.96	19.12	0.15	0.07	267296	1.31	2.04	0.90
'LLXJ01000001	0.00	0.12	191.52	39.13	0.28	0.12	353628	1.48	1.92	0.90
'CP014617.1'	0.33	0.23	8.57	0.60	0.50	0.25	4493502	3.32	12.31	0.99
'LLKL0100032	0.00	-1.00	31.60	1.16	0.35	0.05	5672850	1.94	6.88	0.97
'JXSJ01000001	0.45	0.05	130.01	3.55	0.07	0.01	20211669	0.68	1.34	0.98
'KQ976417.1'	0.00	0.07	92.10	4.16	0.25	0.07	8263949	1.56	3.93	0.98
'KQ982080.1'	0.00	-1.00	79.00	1.92	0.24	0.02	6036021	0.75	4.70	0.98
'KQ977279.1'	0.00	-1.00	76.44	1.84	0.23	0.02	6852325	0.47	4.87	0.98
'KQ979074.1'	0.00	-1.00	75.38	1.63	0.26	0.02	7295537	1.19	4.51	0.98
'KQ981727.1'	0.00	-1.00	75.25	1.62	0.23	0.02	10371161	1.58	4.79	0.98
'CP013963.1'	0.00	-1.00	16.15	0.82	0.56	0.12	2098391	0.00	9.88	0.96
'LDJU0100000	0.20	0.04	322.22	9.14	0.12	0.02	32128745	2.65	0.84	0.97
'KQ991163.1'	0.22	0.10	76.53	5.53	0.19	0.07	1434191	11.47	2.46	0.96
'CP014844.1'	0.09	0.24	10.47	0.85	0.67	0.38	4619440	0.00	12.85	0.99
'CP014808.1'	0.76	0.08	136.74	10.69	0.02	0.01	927432	0.00	0.96	0.90
'BCFV0100000	0.27	0.14	18.02	1.04	0.32	0.12	3521688	0.16	8.87	0.99
'BCFW010000	0.21	0.13	18.87	1.05	0.35	0.13	4014173	0.02	8.78	0.99
'BCFX0100000	0.00	-1.00	56.77	3.77	0.16	0.04	7294945	0.61	5.36	0.88
'BCFY0100000	0.00	-1.00	40.97	1.24	0.39	0.04	1655269	0.24	6.15	0.98
'BCGA010000	0.52	0.10	86.18	5.35	0.07	0.02	1922986	0.77	1.81	0.95
'BCGB010000	0.17	0.14	34.06	2.36	0.30	0.13	2570408	0.03	5.37	0.97
'BCGC010000	0.50	0.10	58.39	3.00	0.09	0.03	3254712	3.96	2.53	0.97
'BCGD010000	0.67	0.11	36.75	1.80	0.08	0.03	2347092	0.50	3.39	0.98
'BCGE010000	0.36	0.09	69.28	3.74	0.13	0.04	2974285	1.09	2.48	0.97
'BCGF0100000	0.17	0.12	25.87	1.25	0.36	0.13	8787864	0.11	6.50	0.99
'BCGI0100000	0.38	0.11	47.39	2.62	0.15	0.06	3147266	0.61	3.34	0.97
'BCGJ0100000	0.38	0.10	62.65	3.49	0.13	0.05	3127730	1.61	2.70	0.97
'BCGK010000	0.47	0.13	27.89	1.58	0.16	0.06	1924846	0.08	5.02	0.98
'BCGL0100000	0.00	-1.00	88.72	4.17	0.28	0.03	2371119	0.24	3.20	0.93
'BCGM010000	0.76	0.13	19.96	0.92	0.10	0.04	3734243	1.23	5.44	0.99
'BCGN010000	0.77	0.09	56.89	2.75	0.04	0.01	2480924	0.00	2.08	0.97
'BCGO010000	0.54	0.13	25.03	1.34	0.14	0.06	2987201	1.29	5.01	0.98
'BCGP010000	0.00	-1.00	248.80	9.77	0.23	0.02	4017233	0.56	1.54	0.93
'BCGQ010000	0.57	0.10	52.73	2.79	0.08	0.03	2934271	0.07	2.67	0.97
'BCGR010000	0.00	-1.00	38.72	2.42	0.24	0.05	2699176	0.15	6.79	0.92
'BCGS010000	0.67	0.11	39.98	1.95	0.07	0.02	3149860	0.18	3.14	0.98
'BCGT0100000	0.00	-1.00	170.59	19.40	0.03	0.01	6182763	0.01	5.26	0.75
'BCGU010000	0.00	-1.00	53.40	3.01	0.21	0.04	4318234	0.29	5.48	0.91
'BCGY010000	0.00	-1.00	65.69	4.88	0.13	0.03	3959504	0.34	5.46	0.87
'BCGZ0100000	0.19	0.16	16.75	1.03	0.42	0.18	3010126	0.58	9.00	0.99
'BCHA0100000	0.56	0.16	15.27	0.87	0.20	0.09	2501896	0.34	7.55	0.99
'BCHB0100000	0.00	-1.00	108.41	9.97	0.07	0.01	6424668	0.09	4.82	0.80
'BCHD010000	0.68	0.13	18.42	0.82	0.13	0.05	5022262	0.20	6.05	0.99
'BCHF0100000	0.00	-1.00	98.86	15.35	0.04	0.01	6530789	0.16	6.46	0.70
'BCHG010000	0.60	0.12	51.23	3.78	0.07	0.03	659822	3.69	2.57	0.96
'BCHH010000	0.00	-1.00	48.11	2.22	0.24	0.04	5232100	2.20	5.60	0.94
'BCHJ0100000	0.00	-1.00	44.37	1.12	0.40	0.04	4116995	0.11	5.42	0.98
'BCHK0100000	0.67	0.10	50.25	2.53	0.06	0.02	1870589	0.84	2.53	0.97
'BCHO010000	0.43	0.13	44.60	2.90	0.13	0.06	2409711	0.21	3.34	0.96
'BCHP0100000	0.00	0.19	29.69	2.30	0.36	0.21	7551078	0.31	7.36	0.97
'BCHT0100000	0.91	0.09	17.87	0.60	0.07	0.02	3245857	0.42	5.56	0.99
'BCHU010000	0.86	0.10	28.68	1.22	0.05	0.02	2409791	0.13	3.74	0.98
'BCHV0100000	0.71	0.17	13.81	0.83	0.15	0.07	1576301	0.28	7.71	0.99
'BCHX0100000	0.00	-1.00	85.01	12.48	0.04	0.01	7437554	0.01	7.19	0.72

'BCHY0100000	0.35	0.14	20.72	1.06	0.26	0.11	6490813	0.17	6.79	0.99
'BCHZ0100000	0.48	0.12	20.98	0.86	0.19	0.07	8684298	0.11	6.26	0.99
'BCIA01000001	0.00	-1.00	24.10	0.89	0.43	0.07	7510044	0.18	7.85	0.97
'BCIC0100000	0.00	0.24	76.49	10.85	0.09	0.08	6618042	0.55	5.68	0.85
'BCID0100000	0.00	0.26	213.15	52.50	0.02	0.03	8587311	0.09	5.18	0.70
'BCIE01000001	0.00	-1.00	38.16	1.13	0.44	0.05	3223726	3.89	5.32	0.98
'BCIF01000001	0.54	0.11	43.29	2.38	0.10	0.04	1903380	0.48	3.36	0.97
'BCIG0100000	0.00	-1.00	44.14	3.93	0.25	0.06	1320572	0.09	5.86	0.88
'BCIJ01000001	0.80	0.16	11.13	0.57	0.16	0.06	1888671	2.14	8.79	0.99
'BCIL01000001	0.40	0.17	22.71	1.57	0.23	0.11	2538488	2.57	5.68	0.98
'BCIO0100000	0.75	0.11	14.53	0.54	0.14	0.04	5326569	0.04	7.37	0.99
'BCIP01000001	0.58	0.10	69.13	4.23	0.06	0.02	1689531	1.18	2.06	0.95
'BCIS01000001	0.51	0.17	15.02	0.89	0.22	0.10	2099666	0.43	7.89	0.99
'BCIT01000001	0.67	0.11	17.77	0.80	0.14	0.04	1765974	0.27	6.50	0.99
'BCIV01000001	0.82	0.14	17.14	0.90	0.10	0.04	1972145	0.04	5.98	0.98
'BCIW0100000	0.27	0.12	31.65	1.87	0.24	0.09	2434256	0.87	5.35	0.98
'BCIX01000001	0.85	0.16	14.62	0.69	0.11	0.05	8012737	0.03	6.76	0.99
'BCIY01000001	0.00	0.29	12.62	1.46	0.73	0.50	1672916	0.06	11.09	0.97
'BCIZ01000001	0.13	0.22	11.87	0.97	0.58	0.31	1973210	0.05	11.02	0.98
'BCJA0100000	0.02	0.28	12.26	1.30	0.72	0.48	1516062	0.07	11.10	0.98
'BCJC0100000	0.67	0.15	18.21	0.87	0.14	0.06	4947887	0.11	5.98	0.99
'BCJD0100000	0.00	0.12	51.23	3.49	0.38	0.15	3339266	0.37	4.62	0.97
'BCJE0100000	0.17	0.29	11.52	1.25	0.56	0.38	1645103	0.31	10.95	0.98
'BCJF0100000	0.06	0.16	16.63	1.00	0.52	0.21	5192464	0.07	10.34	0.99
'BCJG0100000	0.35	0.12	17.32	0.75	0.28	0.09	6129537	0.06	8.39	0.99
'BCJH0100000	0.36	0.17	16.20	1.20	0.29	0.12	1167143	3.87	8.33	0.98
'BCJI01000001	0.09	0.16	21.48	1.36	0.45	0.20	5410471	0.27	8.11	0.98
'BCJN0100000	0.00	-1.00	25.58	1.29	0.27	0.06	4238441	0.31	9.78	0.95
'BCJO0100000	0.00	-1.00	21.26	0.57	0.51	0.06	5948321	0.04	9.16	0.99
'BCJU0100000	0.66	0.09	33.08	1.21	0.09	0.03	6968282	0.07	3.73	0.99
'BCJV0100000	0.79	0.09	26.53	0.93	0.07	0.02	4294417	0.14	4.18	0.99
'BCJW0100000	0.58	0.17	17.66	1.20	0.16	0.08	1604556	1.15	6.91	0.98
'BCJX0100000	0.03	0.15	21.28	1.34	0.51	0.21	3969531	0.38	8.63	0.99
'BCJZ0100000	0.65	0.12	24.28	1.26	0.11	0.04	2328526	0.42	5.05	0.98
'BCKC0100000	0.00	-1.00	42.53	0.91	0.41	0.03	7732581	0.24	5.56	0.98
'BCKD0100000	0.54	0.14	26.20	1.62	0.14	0.06	1523961	3.77	4.80	0.98
'BCKE0100000	0.46	0.12	27.56	1.53	0.16	0.06	1256026	6.67	4.85	0.98
'BCKF0100000	0.00	-1.00	57.96	1.75	0.36	0.04	4845111	0.81	4.14	0.97
'BCKG0100000	0.00	-1.00	51.09	1.40	0.37	0.03	4810746	0.97	4.71	0.98
'BCKH0100000	0.00	0.33	29.55	4.27	0.20	0.21	5152962	0.49	8.49	0.90
'BCKI01000001	0.12	0.15	24.22	1.41	0.41	0.18	5759619	0.03	7.01	0.98
'BCKJ0100000	0.00	0.32	28.99	3.88	0.18	0.18	6947394	0.23	9.53	0.91
'BCKK0100000	0.68	0.11	17.78	0.67	0.13	0.04	3588805	0.60	6.42	0.99
'BCKL0100000	0.32	0.18	15.80	1.13	0.32	0.15	1866479	1.12	8.57	0.98
'BCKN0100000	0.68	0.12	16.74	0.72	0.14	0.05	4122216	0.35	6.67	0.99
'BCKO0100000	0.74	0.11	36.77	1.87	0.06	0.02	2045646	0.12	3.26	0.97
'BCKQ0100000	0.00	-1.00	852.59	63.06	0.17	0.01	2713227	0.56	0.83	0.89
'BCKU0100000	0.00	-1.00	17.87	0.59	0.54	0.07	1665426	0.79	10.33	0.98
'BCKV0100000	0.07	0.14	20.52	1.21	0.47	0.19	5305799	0.15	8.85	0.99
'BCKW0100000	0.74	0.10	52.29	2.45	0.05	0.02	3681944	1.50	2.29	0.97
'BCKX0100000	0.09	0.15	19.44	1.31	0.45	0.18	1477453	0.09	9.22	0.99
'BCKY0100000	0.00	-1.00	15.62	0.71	0.44	0.08	1557178	0.02	12.18	0.97
'BCKZ0100000	0.12	0.09	139.66	11.13	0.22	0.08	2214157	1.54	1.75	0.94
'BCLA0100000	0.07	0.20	19.35	1.73	0.50	0.26	1489088	0.06	8.71	0.98
'BCLC0100000	0.27	0.20	15.02	1.10	0.38	0.19	2178591	0.09	8.97	0.98
'BCLD0100000	0.28	0.17	24.94	1.79	0.28	0.14	2435598	3.09	5.79	0.97
'CM003855.1'	0.51	0.06	282.62	17.57	0.04	0.01	3179732	3.81	0.61	0.91
'CM003869.1'	0.54	0.06	331.75	21.80	0.03	0.01	2947581	1.81	0.52	0.90
'CP014868.1'	0.11	0.23	15.28	1.23	0.54	0.32	6183292	0.11	9.14	0.98
'BCLF0100000	0.00	-1.00	100.73	2.32	0.22	0.01	8501895	3.15	3.99	0.97
'BCLG0100000	0.00	0.11	98.57	9.40	0.26	0.10	1041506	1.08	3.62	0.95
'BCLH0100000	0.00	-1.00	100.99	4.66	0.18	0.02	1506652	0.47	4.69	0.94
'BDAN0100012	0.70	0.15	17.87	1.11	0.13	0.05	792880	0.03	6.29	0.98
'BCLX0100000	0.00	0.11	50.95	3.17	0.39	0.15	4858313	1.20	4.55	0.98
'KV388093.1'	0.48	0.04	291.93	8.91	0.04	0.01	19285141	0.64	0.64	0.96
'KV389426.1'	0.17	0.05	243.95	8.21	0.15	0.03	21314971	4.32	1.04	0.97
'LUEZ0100000	0.66	0.11	20.34	0.87	0.12	0.04	2331425	0.37	5.70	0.99
'CP010347.1'	0.00	-1.00	10.91	0.51	0.73	0.17	4105100	0.00	13.18	0.97
'JSDN01S0271	0.00	0.07	240.52	19.53	0.24	0.07	2592673	6.30	1.62	0.95
'LTYT0100118	0.10	0.09	231.26	29.93	0.19	0.06	866215	9.46	1.36	0.92
'LQHG0103720	0.00	-1.00	158.43	19.39	0.20	0.02	171910	0.01	3.46	0.90
'LQHE0100811	0.00	-1.00	132.45	11.97	0.19	0.02	238187	0.90	4.01	0.93
'LQHF0100525	0.00	-1.00	141.45	20.07	0.23	0.03	127367	0.80	3.46	0.89
'CP013008.1'	0.45	0.12	35.46	1.83	0.14	0.06	6520772	0.02	4.11	0.98
'KV392032.1'	0.00	0.15	28.31	1.83	0.36	0.17	6933627	0.90	8.20	0.98
'CP015048.1'	0.33	0.14	25.33	1.48	0.24	0.10	3629289	2.95	5.51	0.98
'CP015052.1'	0.33	0.14	25.40	1.51	0.24	0.10	3584464	2.27	5.55	0.98
'CP015044.1'	0.34	0.14	25.26	1.44	0.24	0.10	3649879	3.72	5.46	0.98
'CP015046.1'	0.34	0.14	25.26	1.44	0.24	0.10	3631634	3.09	5.50	0.98
'CP015050.1'	0.33	0.14	25.39	1.44	0.24	0.10	3613654	3.11	5.50	0.98
'LFHQ0100053	0.01	0.18	29.36	2.73	0.49	0.26	1444712	0.00	6.59	0.97
'LFHR0100030	0.00	0.22	35.11	4.11	0.31	0.22	1692084	0.00	6.74	0.94
'LFHP0100126	0.03	0.17	28.31	2.39	0.46	0.23	1693913	0.00	6.65	0.97
'LFHS0100044	0.00	0.30	69.60	14.53	0.08	0.08	1736781	0.00	7.09	0.82
'CM003885.1'	0.00	-1.00	111.17	1.68	0.19	0.01	45399244	8.82	3.70	0.98
'CM003897.1'	0.00	-1.00	111.07	1.71	0.19	0.01	44172756	6.11	3.83	0.98
'JYHR0100000	0.00	-1.00	51.15	3.38	0.08	0.02	10013798	0.18	8.37	0.88
'KV407454.1'	0.27	0.12	29.81	1.55	0.26	0.09	3318606	0.04	5.38	0.98
'CM003910.1'	0.00	-1.00	110.56	1.78	0.18	0.01	40619588	0.00	4.22	0.98
'CM003922.1'	0.00	-1.00	111.92	1.70	0.18	0.01	41185095	0.00	4.22	0.98
'CP015225.1'	0.06	0.23	14.87	1.19	0.60	0.35	6921098	0.03	9.55	0.98
'CM003934.1'	0.20	0.03	337.06	6.10	0.13	0.02	202555645	13.48	0.65	0.98
'CM003954.1'	0.22	0.03	333.69	5.80	0.12	0.02	194203610	8.54	0.68	0.98
'KV409358.1'	0.00	0.07	129.05	6.33	0.22	0.06	7072711	0.66	3.23	0.97
'KV408664.1'	0.00	-1.00	163.15	3.22	0.24	0.01	12170939	0.52	2.40	0.97
'KV411217.1'	0.00	-1.00	164.04	2.91	0.26	0.01	16110036	3.59	2.19	0.98
'CM003974.1'	0.22	0.03	333.64	6.09	0.12	0.02	199989126	10.89	0.67	0.98
'CM003994.1'	0.21	0.03	331.53	6.00	0.12	0.02	200424568	13.66	0.65	0.98
'CM004014.1'	0.21	0.03	334.34	6.34	0.12	0.02	216633511	18.92	0.61	0.98
'CM004034.1'	0.23	0.03	326.60	5.91	0.11	0.02	192928109	9.32	0.68	0.98
'CM004074.1'	0.21	0.03	332.82	5.97	0.12	0.02	191003994	8.32	0.69	0.98
'CM004134.1'	0.23	0.03	320.94	5.51	0.11	0.02	223333794	22.21	0.59	0.98
'CM004154.1'	0.20	0.03	339.25	5.95	0.13	0.02	199802105	11.84	0.66	0.98
'CM004114.1'	0.21	0.03	336.90	5.93	0.12	0.02	192210263	8.45	0.68	0.98
'CM004054.1'	0.23	0.03	327.91	5.85	0.11	0.02	200111453	13.94	0.65	0.98
'CM004094.1'	0.24	0.03	330.32	5.70	0.11	0.02	198846266	10.64	0.65	0.98

'LRVB0100019	0.80	0.12	38.77	2.55	0.05	0.02	887480	0.38	2.91	0.96
'LSRZ0100000	0.36	0.14	144.47	36.75	0.09	0.04	108686	0.53	1.78	0.83
'CM004174.1'	0.00	-1.00	108.36	9.33	0.02	0.00	12542233	0.15	8.06	0.79
'CM004278.1'	0.00	0.11	275.33	20.16	0.10	0.05	51465339	6.01	1.72	0.87
'LUGF0100840	0.19	0.09	59.15	3.43	0.23	0.07	1966717	5.37	3.41	0.98
'LFIV01000001	0.21	0.18	25.14	2.33	0.32	0.16	861708	0.00	6.67	0.97
'LFIW01002504	0.50	0.16	20.47	1.45	0.18	0.08	1081293	0.00	6.55	0.98
'KV411306.1'	0.00	-1.00	147.36	3.27	0.21	0.01	11880239	0.83	2.75	0.97
'KV416798.1'	0.00	-1.00	150.74	6.29	0.23	0.02	2131707	1.08	2.57	0.94
'LVCR0100126	0.11	0.10	>1000	>1000	0.11	0.04	262077	6.35	0.88	0.77
'CP015162.1'	0.84	0.15	13.43	0.70	0.12	0.04	1139223	1.17	7.43	0.99
'CP014266.1'	0.19	0.12	51.98	3.36	0.25	0.11	3976746	16.96	2.89	0.97
'KV417480.1'	0.00	-1.00	49.82	3.53	0.19	0.04	1754491	2.31	6.21	0.90
'KV417266.1'	0.00	-1.00	25.76	1.82	0.40	0.09	1238575	0.10	7.76	0.93
'JYNV0100029	0.00	-1.00	45.70	3.97	0.18	0.04	1160210	0.11	6.71	0.88
'LWBM0100002	0.00	-1.00	29.01	0.87	0.49	0.06	2751756	1.40	6.72	0.98
'LWAD0100000	0.14	0.18	26.25	2.52	0.37	0.19	796877	0.01	6.49	0.97
'LVYW0100000	0.06	0.14	24.83	1.42	0.44	0.18	6771991	0.03	7.58	0.98
'LVYX0100000	0.03	0.12	25.57	1.31	0.47	0.17	9646222	0.06	7.59	0.99
'KV419394.1'	0.69	0.17	18.64	1.25	0.13	0.06	1464488	0.07	5.91	0.98
'LNAU0100047	0.56	0.16	23.15	2.08	0.14	0.06	163649	0.25	5.68	0.97
'LNAR0100210	0.50	0.17	25.24	2.75	0.15	0.07	158618	0.00	5.60	0.97
'LMYD0100027	0.26	0.16	30.04	2.85	0.26	0.11	370448	0.00	5.69	0.97
'LNAS0100006	0.17	0.17	34.14	3.23	0.31	0.15	896619	0.00	5.32	0.96
'LTAH0113137	0.00	-1.00	>1000	>1000	0.18	0.03	42897	0.30	2.74	0.75
'CP015286.1'	0.00	-1.00	31.89	1.01	0.43	0.06	5897585	0.00	6.16	0.97
'KV429032.1'	0.44	0.18	16.19	1.15	0.25	0.12	1367360	0.16	7.74	0.98
'KV428004.1'	0.90	0.17	16.65	1.12	0.08	0.04	793452	0.00	5.89	0.98
'KV427605.1'	0.00	0.31	84.46	19.61	0.06	0.06	2261102	1.18	6.80	0.78
'KV425882.1'	0.58	0.20	11.69	0.78	0.24	0.11	1669733	0.16	9.76	0.99
'KV425551.1'	0.49	0.17	20.10	1.38	0.19	0.09	1528150	0.47	6.26	0.98
'KV423914.1'	0.32	0.18	17.46	1.48	0.31	0.14	592208	0.33	8.18	0.98
'KV424459.1'	0.51	0.20	14.79	1.25	0.23	0.11	847827	0.02	8.37	0.98
'LRGB0100051	0.00	-1.00	68.10	3.05	0.24	0.03	3718170	15.50	3.71	0.94
'CM004194.1'	0.22	0.03	331.42	5.75	0.12	0.02	194908223	9.15	0.68	0.98
'CM004215.1'	0.22	0.03	331.19	5.96	0.12	0.02	208915005	16.13	0.63	0.98
'CM004235.1'	0.22	0.03	331.80	5.86	0.12	0.02	200880650	12.38	0.66	0.98
'CM004256.1'	0.21	0.03	333.89	6.03	0.12	0.02	203663563	14.39	0.64	0.98
'LWBZ0100002	0.22	0.11	27.88	1.33	0.30	0.10	4887614	0.20	5.93	0.99
'LKCN0100000	0.00	-1.00	43.39	1.53	0.24	0.03	5272936	5.77	6.88	0.97
'LUCG0100659	0.36	0.15	741.13	>1000	0.07	0.03	55679	0.01	1.42	0.70
'LRBV0100011	0.17	0.08	269.53	35.63	0.14	0.05	861014	1.35	1.21	0.91
'KV775244.1'	0.00	-1.00	95.47	6.56	0.22	0.03	503854	11.22	3.99	0.93
'CM004297.1'	0.77	0.11	40.02	2.11	0.05	0.02	1556355	0.32	2.84	0.97
'CM004191.1'	0.00	-1.00	8.86	0.35	0.64	0.13	3380454	0.00	16.70	0.98
'AZGY0100000	0.00	-1.00	30.55	1.73	0.30	0.07	4538731	0.39	7.16	0.93
'AZGZ0100000	0.00	-1.00	48.47	2.67	0.34	0.06	1592427	1.67	4.77	0.93
'AZHB0100000	0.00	0.15	27.35	1.88	0.49	0.22	3594368	0.09	7.50	0.98
'AZHA0100000	0.00	0.26	73.76	12.72	0.09	0.09	2279171	0.18	6.52	0.85
'AZHC0100000	0.00	-1.00	55.94	6.17	0.14	0.04	2401918	0.05	6.00	0.82
'AZHF0100000	0.00	0.27	35.76	4.23	0.24	0.21	6903712	0.01	7.07	0.92
'AZHD0100000	0.00	-1.00	25.95	0.59	0.44	0.04	3551027	0.19	8.47	0.99
'KV440971.1'	0.10	0.08	117.53	7.00	0.24	0.08	4452461	1.71	2.13	0.96
'CP014501.1'	0.49	0.09	37.14	1.52	0.12	0.04	5930846	0.00	4.06	0.98
'KV441051.1'	0.00	0.15	33.99	2.60	0.49	0.23	2667860	0.00	5.60	0.97
'LXGT0100000	0.42	0.12	33.53	2.51	0.16	0.06	458991	0.00	4.76	0.97
'LXGU0100000	0.41	0.15	34.56	3.24	0.16	0.07	473958	0.01	4.52	0.96
'LLXD0100009	0.31	0.05	187.51	5.89	0.09	0.02	18910200	0.00	1.19	0.97
'CP014278.2'	0.62	0.09	50.95	2.59	0.07	0.02	1811936	0.01	2.62	0.97
'KV441386.1'	0.00	-1.00	56.05	2.61	0.29	0.04	2552699	0.00	4.92	0.94
'LVLJ01002295	0.00	-1.00	153.23	10.90	0.14	0.02	1551447	6.33	3.18	0.88
'LWCA0100000	0.59	0.13	112.18	18.39	0.05	0.02	159981	0.00	1.54	0.86
'KV441548.1'	0.14	0.17	22.84	1.52	0.42	0.20	5402283	0.73	6.99	0.98
'KV441469.1'	0.69	0.12	18.24	0.78	0.13	0.04	2531235	0.00	6.25	0.99
'LSBG0100045	0.35	0.18	73.87	18.30	0.12	0.07	65819	0.06	2.97	0.88
'LTDL0100001	0.63	0.15	29.72	2.26	0.10	0.05	651558	0.00	4.14	0.96
'LTDK0100017	0.38	0.12	128.09	17.91	0.09	0.04	377182	0.01	1.60	0.89
'LWRU0100018	0.00	0.22	47.45	6.62	0.29	0.20	900278	0.03	5.53	0.92
'LWRT0100020	0.29	0.17	28.95	2.59	0.24	0.12	813103	0.00	5.62	0.97
'LSRY0100000	0.38	0.11	83.96	8.96	0.11	0.04	397962	0.49	2.30	0.94
'LWKT0100000	0.52	0.07	44.49	1.27	0.11	0.03	24575218	0.25	3.25	0.99
'LFJK0100000	0.60	0.07	42.95	1.24	0.08	0.02	18579143	2.98	3.09	0.99
'KV441705.1'	0.17	0.14	24.94	1.51	0.37	0.16	3247906	0.38	6.36	0.98
'KV441636.1'	0.00	0.17	31.71	2.52	0.50	0.26	3419696	0.19	6.00	0.97
'CM004348.2'	0.00	-1.00	180.56	3.68	0.06	0.00	310925244	1.85	4.30	0.94
'LWDD0100000	0.26	0.28	18.93	2.85	0.32	0.22	203074	2.16	7.82	0.95
'LWDF0100000	0.18	0.28	22.51	3.67	0.35	0.25	303331	0.36	7.21	0.94
'LWDE0100000	0.45	0.16	84.86	16.94	0.08	0.04	113829	0.00	2.35	0.87
'LWDG0100000	0.48	0.21	17.35	1.81	0.21	0.11	260180	0.30	7.44	0.97
'LVCJ0100000	0.59	0.13	20.07	1.08	0.15	0.05	1192800	0.00	6.14	0.99
'LVCI01000064	0.64	0.15	19.09	1.19	0.14	0.05	1047054	0.00	6.17	0.98
'LTAE0100000	0.00	-1.00	330.37	57.75	0.19	0.02	249015	3.30	2.15	0.87
'LVCG0100000	0.00	-1.00	161.44	21.93	0.21	0.02	143306	0.45	3.37	0.90
'LVCH0100005	0.00	0.14	256.29	124.28	0.22	0.10	81532	0.59	2.95	0.89
'LWBQ0100000	0.00	0.20	25.72	2.44	0.42	0.24	1919695	3.82	7.66	0.97
'LVTN0100000	0.00	-1.00	290.18	15.77	0.17	0.01	1648447	2.44	1.97	0.93
'CM004386.1'	0.03	0.24	12.35	1.06	0.69	0.41	3003247	0.00	11.04	0.98
'LWHD0100000	0.14	0.06	150.66	5.96	0.19	0.04	11911191	3.21	1.78	0.97
'KV441875.1'	0.13	0.14	31.01	2.43	0.33	0.14	1252801	2.77	6.25	0.98
'LXWW010002	0.00	-1.00	29.99	1.83	0.46	0.07	286747	2.45	6.82	0.96
'LVKQ01S0010	0.27	0.06	384.36	34.41	0.08	0.02	2424496	0.62	0.71	0.90
'KV442011.1'	0.37	0.11	33.81	1.88	0.18	0.06	1526291	0.03	4.61	0.98
'LHPN0100000	0.00	-1.00	43.35	1.51	0.42	0.05	4062027	0.27	4.82	0.97
'LHPM0100001	0.21	0.13	35.03	2.07	0.28	0.12	4066456	0.08	4.78	0.98
'CM004359.1'	0.10	0.07	104.51	3.56	0.26	0.07	29387870	0.93	2.29	0.98
'LVYI01000001	0.51	0.14	20.32	1.04	0.18	0.07	4892217	0.05	6.32	0.99
'LSNX0103146	0.00	-1.00	40.52	1.22	0.30	0.03	3675446	1.83	6.70	0.98
'KV442483.1'	0.36	0.11	30.49	1.43	0.20	0.07	4225797	0.25	4.98	0.99
'CP015814.2'	0.27	0.16	26.84	1.88	0.27	0.13	3550837	0.01	5.58	0.97
'CM004372.1'	0.00	-1.00	112.79	1.96	0.15	0.01	65039919	1.43	4.29	0.97
'BDDP0100362	0.65	0.22	23.10	3.02	0.11	0.06	118917	0.08	5.23	0.95
'LSBH0100000	0.00	0.39	34.73	5.95	0.12	0.15	6692040	0.18	8.67	0.86
'LUKN0100319	0.62	0.20	18.53	1.98	0.15	0.07	134276	1.27	6.38	0.97
'LSBJ0100000	0.19	0.16	29.92	1.92	0.33	0.16	7099765	0.18	5.33	0.97
'LSBI01000001	0.00	-1.00	31.20	2.30	0.17	0.05	5703399	0.11	8.63	0.89
'CP014639.1'	0.77	0.11	43.37	2.51	0.05	0.02	1113233	0.01	2.71	0.97

'LSRL0100000	0.00	-1.00	94.31	2.15	0.20	0.02	24697304	0.59	3.83	0.97
'LSRM0100000	0.00	-1.00	79.24	1.39	0.28	0.02	29979347	2.98	3.70	0.98
'LVXX0100000	0.00	0.06	333.17	23.97	0.25	0.07	6409783	6.06	1.08	0.93
'LVYE0100000	0.58	0.15	18.48	1.30	0.16	0.06	637988	0.00	6.66	0.98
'LTDI01000013	0.42	0.18	29.89	3.27	0.17	0.09	304831	0.02	4.93	0.95
'LUFC0100008	0.56	0.19	27.23	3.06	0.13	0.07	232868	0.00	4.83	0.95
'LXPA0100501	0.50	0.09	>1000	>1000	0.03	0.01	221254	0.16	0.53	0.59
'LXOY0100551	0.49	0.10	>1000	>1000	0.03	0.01	104990	0.28	0.68	0.59
'LXOZ0103202	0.76	0.12	788.57	726.81	0.02	0.01	55426	0.22	0.78	0.42
'LPNZ0100987	0.48	0.14	64.61	9.07	0.09	0.04	135689	0.99	2.66	0.92
'CP010558.1'	0.82	0.16	13.61	0.77	0.12	0.05	1139466	0.04	7.50	0.99
'CP010559.1'	0.82	0.16	13.61	0.77	0.12	0.05	1139722	0.04	7.51	0.99
'CP010560.1'	0.82	0.16	13.62	0.77	0.12	0.05	1138471	0.04	7.50	0.99
'CP010561.1'	0.82	0.16	13.61	0.77	0.12	0.05	1139641	0.04	7.51	0.99
'CP010562.1'	0.82	0.16	13.61	0.77	0.12	0.05	1139594	0.04	7.50	0.99
'CP010563.1'	0.82	0.16	13.62	0.77	0.12	0.05	1139829	0.04	7.51	0.99
'CP010564.1'	0.82	0.16	13.61	0.77	0.12	0.05	1140059	0.04	7.51	0.99
'CP010565.1'	0.82	0.16	13.61	0.77	0.12	0.05	1139865	0.04	7.50	0.99
'CP010566.1'	0.82	0.16	13.61	0.77	0.12	0.05	1139777	0.04	7.50	0.99
'LQHL0100187	0.57	0.16	79.82	13.63	0.06	0.03	138529	0.07	2.04	0.86
'LQMZ0100223	0.56	0.14	80.89	12.35	0.06	0.03	145420	0.00	2.05	0.88
'CP010029.1'	0.00	-1.00	32.68	1.24	0.36	0.05	4275406	0.01	6.64	0.96
'LQMS0100238	0.59	0.12	66.09	6.21	0.06	0.02	330748	0.07	2.22	0.94
'JNNX0100000	0.13	0.12	31.29	1.73	0.36	0.14	4236361	0.01	5.81	0.98
'KV448122.1'	0.92	0.14	18.80	1.11	0.07	0.03	560457	0.00	5.19	0.98
'CM004387.1'	0.02	0.05	195.17	5.78	0.27	0.06	34959721	12.48	1.47	0.98
'CP015922.1'	0.55	0.14	30.93	1.93	0.12	0.05	2232205	0.00	4.19	0.97
'KV452433.1'	0.82	0.11	37.02	1.91	0.05	0.02	1544349	2.73	2.92	0.97
'KV452459.1'	0.00	-1.00	35.69	1.59	0.39	0.05	956893	3.30	6.29	0.97
'CM004405.1'	0.56	0.10	45.37	2.54	0.09	0.03	1808275	2.33	3.06	0.97
'CM004414.1'	0.00	0.05	188.89	5.72	0.28	0.06	37510255	1.03	1.79	0.98
'LGUA0100000	0.57	0.18	29.52	3.70	0.12	0.05	79735	0.00	4.62	0.95
'LVCL01S0001	0.36	0.06	503.98	57.74	0.05	0.02	1905173	0.41	0.54	0.86
'LSRQ0100111	0.00	-1.00	129.45	5.07	0.25	0.02	1287057	0.55	2.92	0.96
'BCYN0100000	0.00	-1.00	135.48	7.23	0.26	0.02	1053114	3.96	2.50	0.93
'BDDV0100003	0.54	0.16	21.45	1.43	0.16	0.08	1481398	0.01	5.79	0.98
'KV454001.1'	0.25	0.11	47.64	2.94	0.20	0.07	2697971	0.84	4.12	0.97
'KV454011.1'	0.29	0.08	157.39	10.33	0.11	0.04	2514465	1.97	1.33	0.94
'KV454208.1'	0.38	0.08	130.22	7.16	0.09	0.03	3342607	2.03	1.40	0.95
'KV454254.1'	0.44	0.14	20.28	1.22	0.21	0.08	1476293	0.34	6.66	0.98
'KV454406.1'	0.57	0.11	43.69	2.42	0.09	0.03	2480304	0.32	3.16	0.97
'KV454289.1'	0.41	0.14	24.57	1.46	0.20	0.08	2131963	3.57	5.65	0.98
'KV454426.1'	0.24	0.13	33.20	2.10	0.26	0.10	2639018	1.01	5.16	0.98
'KV454475.1'	0.26	0.08	128.29	8.11	0.14	0.04	3341275	0.27	1.70	0.95
'KV454538.1'	0.43	0.10	75.73	4.66	0.10	0.04	2734067	0.19	2.14	0.95
'KV453925.1'	0.65	0.11	37.86	1.96	0.08	0.03	1920980	0.81	3.37	0.98
'KV453909.1'	0.34	0.11	44.74	2.46	0.17	0.06	2646005	0.22	3.78	0.98
'KV453847.1'	0.05	0.10	145.22	14.66	0.27	0.11	1544913	1.07	1.86	0.93
'KV453841.1'	0.52	0.11	27.39	1.29	0.14	0.05	3117240	0.69	4.96	0.98
'LUVW0100000	0.59	0.10	54.43	2.94	0.07	0.02	1890184	0.14	2.56	0.97
'BCNH0100013	0.13	0.06	88.35	2.92	0.23	0.06	15115857	3.19	2.71	0.98
'BDCZ0100000	0.51	0.12	27.51	1.80	0.14	0.05	644638	2.59	5.00	0.98
'BDDA0100000	0.44	0.17	21.08	1.72	0.20	0.09	888611	3.58	6.49	0.97
'BDDB0100000	0.33	0.24	14.97	1.72	0.32	0.18	262958	4.60	8.81	0.97
'BDDC0100000	0.19	0.25	13.47	1.56	0.45	0.26	403447	1.15	10.83	0.98
'BCFZ0100000	0.00	-1.00	35.42	1.31	0.24	0.03	5014741	0.04	8.27	0.96
'LYNE0100000	0.07	0.10	158.24	21.11	0.23	0.09	548636	0.71	1.99	0.93
'KV454974.1'	0.15	0.14	32.07	2.44	0.33	0.14	990412	0.08	5.75	0.97
'KV454565.1'	0.00	-1.00	54.62	4.52	0.23	0.05	741106	0.05	5.58	0.89
'KV455952.1'	0.36	0.23	28.15	4.55	0.20	0.12	129019	0.26	5.74	0.94
'KV458779.1'	0.24	0.16	29.52	2.83	0.27	0.12	867888	0.01	5.89	0.97
'KV459350.1'	0.12	0.18	33.03	3.84	0.33	0.16	361480	0.16	5.96	0.96
'KV460206.1'	0.05	0.16	34.44	2.99	0.42	0.21	1768408	0.02	5.75	0.97
'LVCK0100103	0.58	0.18	21.77	2.09	0.14	0.07	266087	1.15	5.79	0.97
'LZRM0100156	0.44	0.19	23.12	2.51	0.19	0.10	315561	0.91	6.10	0.96
'JTCL0100411	0.45	0.10	228.90	40.28	0.05	0.02	263365	0.09	1.08	0.85
'LZCU0114795	0.00	-1.00	582.97	440.82	0.20	0.03	48998	0.18	2.66	0.82
'LHSH0100001	0.12	0.08	185.81	15.01	0.19	0.06	1759740	2.40	1.57	0.94
'CM004466.1'	0.30	0.03	180.08	2.92	0.09	0.02	219802468	10.48	1.29	0.99
'LXPE0100000	0.49	0.09	632.42	170.40	0.03	0.01	834636	29.28	0.38	0.69
'LXTC0100000	0.00	0.14	53.82	4.01	0.27	0.14	5390915	2.18	5.14	0.96
'CP011806.1'	0.74	0.23	6.85	0.41	0.28	0.14	5989545	0.05	12.65	0.99
'CP007916.1'	0.76	0.11	40.91	2.21	0.05	0.02	1530234	4.22	2.73	0.97
'CP007814.1'	0.79	0.11	39.78	2.21	0.05	0.02	1530729	4.53	2.72	0.97
'CP007831.1'	0.76	0.12	40.65	2.34	0.05	0.02	1531424	4.18	2.73	0.97
'CP007848.1'	0.78	0.11	39.90	2.21	0.05	0.02	1530735	4.44	2.72	0.97
'CP007865.1'	0.75	0.11	41.27	2.25	0.06	0.02	1530485	4.44	2.72	0.97
'CP007882.1'	0.80	0.10	39.49	1.99	0.05	0.02	1530802	4.46	2.72	0.97
'CP007899.1'	0.77	0.10	40.44	2.09	0.05	0.02	1530305	5.09	2.70	0.97
'CP007933.1'	0.75	0.11	40.95	2.30	0.06	0.02	1530852	4.34	2.73	0.97
'CP007950.1'	0.78	0.11	40.05	2.11	0.05	0.02	1530742	3.95	2.74	0.97
'CP007967.1'	0.77	0.11	40.16	2.08	0.05	0.02	1530513	4.16	2.73	0.97
'CP008035.1'	0.79	0.10	39.67	1.99	0.05	0.02	1531016	4.35	2.73	0.97
'CP008052.1'	0.77	0.10	40.36	2.06	0.05	0.02	1530644	4.58	2.72	0.97
'CP008069.1'	0.74	0.11	41.54	2.23	0.06	0.02	1530684	4.43	2.72	0.97
'CP008120.1'	0.77	0.11	40.33	2.14	0.05	0.02	1530749	4.11	2.73	0.97
'CP007984.1'	0.78	0.10	40.26	2.02	0.05	0.02	1530792	4.24	2.73	0.97
'CP008001.1'	0.76	0.10	40.87	2.16	0.05	0.02	1530765	4.46	2.72	0.97
'CP008018.1'	0.76	0.11	40.84	2.19	0.05	0.02	1530513	4.44	2.72	0.97
'CP008154.1'	0.77	0.11	40.47	2.14	0.05	0.02	1530499	3.96	2.74	0.97
'CP008324.1'	0.79	0.11	39.87	2.08	0.05	0.02	1530881	4.42	2.73	0.97
'CP008290.1'	0.76	0.12	40.53	2.36	0.05	0.02	1530443	4.51	2.72	0.97
'CP008222.1'	0.78	0.10	40.06	2.04	0.05	0.02	1530511	4.21	2.73	0.97
'CP008409.1'	0.77	0.11	40.48	2.28	0.05	0.02	1530452	4.81	2.71	0.97
'CP008392.1'	0.78	0.10	40.13	2.06	0.05	0.02	1530795	4.36	2.72	0.97
'CP008341.1'	0.79	0.11	39.73	2.13	0.05	0.02	1530217	4.48	2.72	0.97
'CP008494.1'	0.80	0.11	39.40	2.17	0.05	0.02	1530775	4.46	2.72	0.97
'CP008460.1'	0.76	0.10	40.60	2.13	0.05	0.02	1530726	4.40	2.73	0.97
'CP008443.1'	0.77	0.10	40.40	2.06	0.05	0.02	1530407	4.50	2.72	0.97
'CP008426.1'	0.77	0.11	40.31	2.15	0.05	0.02	1530770	4.60	2.72	0.97
'CP008562.1'	0.78	0.11	40.11	2.10	0.05	0.02	1530685	4.53	2.72	0.97
'CP008545.1'	0.78	0.11	40.03	2.17	0.05	0.02	1530871	4.12	2.73	0.97
'CP008528.1'	0.77	0.11	40.41	2.16	0.05	0.02	1530872	4.16	2.73	0.97
'CP008511.1'	0.75	0.11	40.85	2.25	0.05	0.02	1530875	4.10	2.73	0.97
'CP008647.1'	0.74	0.10	41.27	2.19	0.06	0.02	1531134	4.50	2.72	0.97
'CP008613.1'	0.76	0.10	41.01	2.15	0.05	0.02	1530074	4.53	2.72	0.97

'CP008596.1'	0.77	0.11	40.29	2.14	0.05	0.02	1530476	4.05	2.74	0.97
'CP008205.1'	0.77	0.11	40.65	2.17	0.05	0.02	1530699	4.55	2.72	0.97
'CP008188.1'	0.77	0.09	40.49	1.89	0.05	0.02	1530444	4.89	2.71	0.98
'CP008171.1'	0.76	0.11	40.82	2.26	0.05	0.02	1530586	4.97	2.71	0.97
'CP008086.1'	0.75	0.11	40.98	2.18	0.05	0.02	1531039	4.47	2.73	0.97
'CP008103.1'	0.76	0.11	40.55	2.17	0.05	0.02	1530432	4.30	2.73	0.97
'CP008137.1'	0.71	0.11	42.46	2.36	0.06	0.02	1530886	4.03	2.73	0.97
'CP008681.1'	0.78	0.11	40.17	2.13	0.05	0.02	1530728	4.42	2.72	0.97
'CP008630.1'	0.72	0.11	42.12	2.29	0.06	0.02	1530791	4.42	2.72	0.97
'CP008664.1'	0.76	0.11	40.58	2.21	0.05	0.02	1530085	4.49	2.72	0.97
'CP008579.1'	0.77	0.10	40.27	2.08	0.05	0.02	1530497	3.91	2.74	0.97
'CP008477.1'	0.78	0.10	40.15	2.05	0.05	0.02	1530455	4.23	2.73	0.97
'CP008358.1'	0.77	0.11	40.25	2.16	0.05	0.02	1530678	4.55	2.72	0.97
'CP008375.1'	0.80	0.11	39.52	2.08	0.05	0.02	1530696	4.97	2.71	0.97
'CP008256.1'	0.75	0.11	41.15	2.32	0.06	0.02	1530977	4.23	2.73	0.97
'CP008307.1'	0.78	0.11	40.07	2.14	0.05	0.02	1530667	4.50	2.72	0.97
'CP008273.1'	0.78	0.10	40.21	2.07	0.05	0.02	1530687	5.00	2.71	0.97
'CP008239.1'	0.74	0.11	41.44	2.20	0.06	0.02	1530860	4.44	2.72	0.97
'CP016034.1'	0.00	-1.00	31.77	1.29	0.29	0.05	4893592	0.43	7.40	0.96
'JRVM0100000	0.57	0.17	39.36	5.73	0.10	0.04	70990	0.00	3.74	0.93
'CM004455.1'	0.00	-1.00	234.06	45.99	0.01	0.00	6042495	0.00	7.06	0.66
'CP013643.1'	0.14	0.26	10.83	0.95	0.58	0.36	4561148	0.00	12.12	0.98
'CP013636.1'	0.14	0.26	10.83	0.95	0.58	0.36	4562893	0.00	12.12	0.98
'LYXU0100000	0.00	-1.00	>1000	767.65	0.01	0.00	11790407	0.00	4.62	0.61
'LNFW0100093	0.37	0.20	25.00	2.69	0.22	0.12	348108	0.80	5.86	0.96
'LZPO0105748	0.47	0.05	353.19	20.66	0.04	0.01	4085094	3.77	0.53	0.92
'LXOO0100000	0.16	0.24	30.49	6.17	0.30	0.18	125452	1.28	6.54	0.94
'LXOP0100000	0.28	0.17	28.69	3.09	0.24	0.11	257108	1.92	6.02	0.97
'LXON0100000	0.24	0.24	27.90	5.19	0.27	0.16	89170	0.53	6.41	0.94
'LXOQ0100000	0.37	0.14	26.18	1.86	0.21	0.08	823909	0.80	5.96	0.98
'LZPB0100000	0.00	-1.00	22.50	1.05	0.44	0.06	398088	0.01	9.50	0.98
'CP016227.1'	0.43	0.11	59.50	3.68	0.11	0.04	2126190	3.58	2.60	0.96
'LVWM0100000	0.67	0.19	19.74	1.99	0.12	0.06	132493	0.00	5.90	0.97
'CP016285.1'	0.40	0.09	51.28	2.29	0.13	0.04	5113413	0.10	3.11	0.98
'LWUH0100107	0.78	0.20	20.19	2.24	0.09	0.05	157180	0.00	5.40	0.96
'CP016250.1'	0.00	-1.00	82.19	2.70	0.32	0.03	4930710	0.00	3.21	0.96
'LYYD0100031	0.66	0.12	30.33	1.67	0.09	0.03	1730003	16.28	3.37	0.98
'CP008725.1'	0.67	0.09	61.48	3.25	0.05	0.02	1714148	4.61	2.03	0.97
'CP015470.1'	0.00	0.28	51.85	7.99	0.20	0.20	3779356	0.00	5.06	0.87
'KV575286.1'	0.30	0.05	175.84	5.53	0.10	0.02	16950971	0.62	1.26	0.97
'CM004498.1'	0.00	-1.00	147.47	2.48	0.22	0.01	44809562	11.04	2.28	0.97
'CP016201.1'	0.00	-1.00	23.79	1.02	0.29	0.05	2886041	1.23	10.11	0.97
'CP016204.1'	0.40	0.12	48.12	3.11	0.14	0.06	1859892	0.08	3.24	0.96
'CP016207.1'	0.47	0.10	50.57	2.70	0.11	0.04	2007206	0.21	3.01	0.97
'LPWI0100000	0.00	-1.00	162.40	3.37	0.23	0.01	23816425	1.12	2.25	0.97
'LUGH0100000	0.54	0.15	79.80	12.59	0.07	0.03	155986	0.00	2.10	0.88
'CP018146.1'	0.36	0.11	42.47	2.12	0.17	0.06	4221879	0.05	3.71	0.98
'KV581049.1'	0.00	-1.00	136.71	18.78	0.03	0.01	4471846	2.12	6.38	0.73
'KV580561.1'	0.00	-1.00	60.24	6.11	0.10	0.03	4526020	1.86	6.54	0.82
'KV580651.1'	0.17	0.21	27.57	3.85	0.32	0.17	187720	0.02	6.70	0.96
'KV580213.1'	0.29	0.17	23.81	2.58	0.26	0.12	231435	0.03	7.02	0.97
'LOEQ0100002	0.34	0.07	167.63	10.76	0.09	0.03	2242254	3.88	1.21	0.94
'LOJH0100000	0.52	0.11	388.00	134.95	0.03	0.01	160076	0.41	0.88	0.71
'LOJG0100000	0.40	0.11	429.95	181.91	0.05	0.02	160323	0.41	0.99	0.75
'JSZB0100023	0.13	0.08	195.88	15.93	0.19	0.06	2314370	1.18	1.38	0.93
'MATR0100014	0.21	0.08	135.78	8.38	0.16	0.05	2949784	0.91	1.75	0.96
'MAMN0100002	0.02	0.08	218.52	24.90	0.25	0.08	1233026	10.81	1.50	0.93
'MATQ0100003	0.20	0.08	137.75	10.38	0.15	0.05	1614553	2.55	1.78	0.95
'CP011109.1'	0.50	0.12	34.95	1.86	0.13	0.05	3659360	0.45	3.84	0.98
'KV700387.1'	0.14	0.11	63.83	4.21	0.26	0.10	2471670	0.67	3.25	0.97
'CM007197.1'	0.00	-1.00	168.06	2.92	0.17	0.01	83567108	36.79	1.67	0.97
'KV722330.1'	0.53	0.18	16.59	1.26	0.20	0.09	721539	0.11	7.41	0.98
'MATU0100395	0.41	0.19	>1000	>1000	0.07	0.04	63469	68.92	0.56	0.64
'MAPY0100042	0.53	0.25	17.56	2.50	0.18	0.11	112423	0.03	7.28	0.96
'MAPW0100000	0.17	0.20	23.75	2.33	0.36	0.19	924144	0.00	7.06	0.97
'JNHY0100386	0.75	0.15	19.86	1.37	0.10	0.04	384155	0.00	5.51	0.98
'JNHX0101099	0.77	0.15	21.48	1.82	0.09	0.04	158560	0.00	5.19	0.97
'JNHW0100584	0.73	0.13	22.05	1.09	0.10	0.04	2165298	0.01	5.08	0.98
'JNHV0101611	0.81	0.14	18.89	1.23	0.09	0.03	496966	0.19	5.64	0.98
'JNHZ0100357	0.90	0.15	19.23	1.13	0.07	0.03	1066430	0.04	5.11	0.98
'KV744805.1'	0.67	0.15	21.83	1.66	0.11	0.04	320835	0.00	5.47	0.98
'KV748176.1'	0.00	-1.00	77.15	2.06	0.34	0.03	6408476	0.06	3.39	0.97
'KV749029.1'	0.58	0.18	23.44	2.54	0.13	0.06	150620	0.44	5.45	0.96
'MBSK0100000	0.32	0.08	122.48	8.47	0.11	0.04	1768557	0.12	1.71	0.95
'MASO0200000	0.00	-1.00	30.54	2.08	0.37	0.08	623116	0.00	7.06	0.93
'MASP0200002	0.00	-1.00	>1000	993.66	0.02	0.00	1106886	0.01	3.85	0.74
'LWMJ0200000	0.70	0.09	33.81	1.33	0.07	0.02	4220646	0.70	3.62	0.98
'LWMK0200000	0.64	0.08	36.08	1.21	0.08	0.02	7334011	0.27	3.55	0.99
'CM004509.1'	0.34	0.10	64.48	3.70	0.15	0.05	2901642	0.00	2.69	0.97
'MBPM0100000	0.49	0.14	25.13	1.80	0.16	0.07	763219	1.68	5.34	0.98
'JYJY0100023	0.40	0.17	26.15	2.31	0.20	0.09	591266	0.13	5.67	0.97
'MBGJ0100010	0.23	0.10	141.58	13.04	0.14	0.05	1541094	1.03	1.62	0.92
'LSHC0100035	0.62	0.15	18.61	1.16	0.15	0.06	1086544	0.00	6.32	0.98
'CP016787.1'	0.08	0.15	35.46	2.63	0.38	0.18	2902310	0.01	5.18	0.97
'CP016788.1'	0.07	0.15	35.93	2.82	0.40	0.19	2759298	0.01	5.16	0.97
'CP016789.1'	0.16	0.15	33.59	2.43	0.32	0.15	2680328	0.01	5.15	0.97
'MCGX0100381	0.34	0.04	279.49	9.80	0.07	0.02	14887386	4.34	0.77	0.96
'KV751334.1'	0.36	0.05	108.76	2.80	0.10	0.02	32686547	1.58	1.80	0.98
'LUXX0107424	0.08	0.05	281.53	11.53	0.20	0.04	11394010	0.70	1.15	0.97
'LUXR0110179	0.25	0.04	325.89	11.43	0.10	0.02	15393348	0.65	0.77	0.96
'LUXS0107603	0.00	-1.00	284.40	7.05	0.27	0.01	9165455	0.55	1.24	0.96
'LUXQ0111673	0.00	-1.00	206.88	5.09	0.28	0.02	8219058	0.63	1.64	0.96
'LUXT0105095	0.00	-1.00	203.21	4.16	0.28	0.01	13569045	0.95	1.64	0.97
'LUXV0100974	0.00	0.06	231.69	10.32	0.29	0.07	13407224	1.41	1.41	0.96
'LUXU0103831	0.07	0.06	186.03	8.69	0.24	0.06	9963048	0.72	1.57	0.96
'LUXY0107512	0.00	0.08	146.90	7.80	0.26	0.08	10060138	0.87	2.31	0.96
'LUXW0108837	0.00	-1.00	126.96	3.37	0.23	0.02	8406765	0.42	2.86	0.96
'CP007484.1'	0.00	-1.00	29.29	1.27	0.27	0.05	4891923	0.00	8.44	0.96
'KV756872.1'	0.00	-1.00	45.18	2.65	0.38	0.06	1370908	0.71	4.81	0.93
'LGRB0100000	0.00	-1.00	29.65	1.55	0.32	0.07	4529175	0.01	7.01	0.93
'CM004529.1'	0.52	0.03	347.06	5.26	0.03	0.00	147836772	1.03	0.54	0.98
'MAVT0100106	0.13	0.25	38.14	9.61	0.30	0.19	54299	0.00	5.74	0.92
'MABJ0100002	0.43	0.12	29.17	1.63	0.17	0.06	2001134	0.02	4.97	0.98
'MAKY0100035	0.37	0.15	30.80	2.28	0.19	0.08	988797	0.00	4.93	0.97
'MAKZ0100007	0.51	0.14	27.42	1.89	0.14	0.06	1018461	0.04	4.92	0.97
'MABM0100000	0.00	0.21	47.32	5.18	0.30	0.22	4224935	0.02	4.94	0.92

'MABL0100031	0.24	0.14	33.45	2.27	0.26	0.11	1938854	0.01	4.93	0.97
'MABK0100011	0.40	0.14	30.09	2.10	0.18	0.08	1267461	0.00	4.96	0.97
'MABN0100002	0.42	0.12	29.25	1.56	0.18	0.07	3464760	0.01	4.96	0.98
'MABP0100002	0.56	0.14	26.97	1.79	0.13	0.05	987741	0.02	4.90	0.98
'MABS0100003	0.40	0.11	30.02	1.48	0.18	0.06	3357292	0.01	4.96	0.98
'MABO0100012	0.50	0.12	27.86	1.57	0.15	0.05	941764	0.01	4.98	0.98
'MABQ0100000	0.46	0.12	28.27	1.45	0.16	0.06	2409929	0.02	4.95	0.98
'MAMH0100002	0.49	0.13	27.40	1.56	0.16	0.06	2079380	0.00	5.01	0.98
'MABR0100002	0.42	0.13	28.92	1.68	0.18	0.07	2476658	0.01	4.96	0.98
'MALA0100004	0.48	0.13	27.73	1.62	0.16	0.06	2247237	0.01	5.02	0.98
'MALC0100009	0.35	0.14	30.86	2.08	0.20	0.08	1308757	0.02	4.95	0.97
'MALB0100015	0.38	0.13	30.88	1.93	0.19	0.07	1543545	0.00	4.93	0.98
'MALD0100003	0.46	0.11	28.37	1.40	0.17	0.06	2415299	0.00	4.95	0.98
'MALE0100008	0.34	0.15	31.78	2.40	0.21	0.09	1132243	0.00	4.93	0.97
'MALF0100004	0.00	-1.00	41.92	1.99	0.40	0.06	2513951	0.02	4.89	0.95
'MALG0100006	0.38	0.15	30.79	2.28	0.19	0.08	989948	0.01	4.93	0.97
'MALH0100002	0.62	0.13	25.83	1.57	0.11	0.04	878909	0.00	4.91	0.98
'MALI01000064	0.43	0.15	29.70	2.18	0.17	0.07	951960	0.00	4.94	0.97
'MALK0100000	0.36	0.15	30.96	2.50	0.20	0.09	858320	0.00	4.92	0.97
'MALJ0100004	0.33	0.14	31.77	2.27	0.21	0.09	1021840	0.00	4.95	0.97
'MALL0100002	0.48	0.15	28.34	2.02	0.15	0.07	1012475	0.00	4.94	0.97
'MALM0100014	0.48	0.14	27.82	1.91	0.15	0.06	855033	0.01	4.99	0.98
'MALO0100013	0.44	0.12	29.39	1.81	0.17	0.06	835465	0.00	4.96	0.98
'MALN0100000	0.31	0.14	32.17	2.18	0.22	0.09	1184915	0.00	4.95	0.98
'MALP0100003	0.38	0.13	30.21	1.85	0.19	0.07	1253049	0.00	4.99	0.98
'MALQ0100002	0.32	0.13	31.09	1.95	0.23	0.09	2228049	0.00	4.98	0.98
'MALR0100004	0.49	0.15	27.95	2.07	0.15	0.06	809029	0.00	5.00	0.97
'MALS0100007	0.33	0.14	31.77	2.28	0.21	0.09	1022034	0.00	4.95	0.97
'MALU0100000	0.41	0.14	29.31	2.03	0.18	0.07	1535963	0.00	5.00	0.97
'MALT0100004	0.43	0.12	29.73	1.75	0.17	0.06	1517907	0.01	4.94	0.98
'MALW0100004	0.36	0.15	30.51	2.30	0.20	0.09	1364783	0.01	4.92	0.97
'MALY0100004	0.56	0.12	26.59	1.49	0.13	0.05	983939	0.00	4.95	0.98
'MALX0100001	0.43	0.12	28.87	1.53	0.17	0.06	2769734	0.34	4.98	0.98
'MALZ0100001	0.52	0.14	27.29	1.79	0.14	0.06	1364775	0.00	4.96	0.98
'MAMA0100000	0.40	0.14	29.79	1.82	0.19	0.08	2828477	1.26	4.90	0.98
'MAMC0100011	0.50	0.12	27.87	1.50	0.15	0.05	1419445	0.00	4.98	0.98
'MAMB0100004	0.59	0.14	26.10	1.84	0.12	0.05	753059	0.00	4.94	0.97
'MAME0100013	0.45	0.13	28.80	1.86	0.17	0.07	1350666	0.00	4.97	0.98
'MAMD0100011	0.35	0.15	31.64	2.58	0.20	0.09	1022864	0.01	4.94	0.97
'MAMG0100002	0.60	0.13	26.20	1.64	0.12	0.05	878799	0.00	4.91	0.98
'MAMF0100012	0.48	0.15	27.76	2.01	0.16	0.07	959539	0.00	5.02	0.97
'MABT0100003	0.43	0.12	29.18	1.63	0.17	0.06	2000702	0.03	4.97	0.98
'LQRL0115027	0.40	0.12	55.05	5.83	0.12	0.05	244066	1.01	3.27	0.95
'CP016880.1'	0.00	0.26	33.94	4.57	0.20	0.16	2207174	0.00	8.71	0.93
'CP016882.1'	0.00	-1.00	34.94	2.16	0.18	0.04	2173015	0.00	8.75	0.92
'CP016881.1'	0.00	-1.00	34.11	2.09	0.19	0.04	2162082	0.00	8.77	0.93
'CP016883.1'	0.00	-1.00	34.65	2.11	0.19	0.04	2168169	0.00	8.74	0.93
'CP016884.1'	0.00	-1.00	34.78	2.19	0.18	0.04	2193498	0.00	8.76	0.92
'CP016886.1'	0.00	0.26	34.77	4.76	0.19	0.15	2211323	0.00	8.68	0.93
'CP016885.1'	0.00	0.26	34.81	4.76	0.19	0.15	2204403	0.00	8.70	0.93
'CM004556.1'	0.00	-1.00	71.84	6.03	0.11	0.03	11717239	0.18	4.92	0.82
'CM004562.1'	0.21	0.03	314.17	5.54	0.12	0.02	157403528	0.00	0.85	0.98
'MBPS0100005	0.31	0.13	31.69	1.95	0.22	0.09	2003318	0.00	4.98	0.98
'CP015855.1'	0.00	-1.00	35.25	1.42	0.25	0.04	5351654	0.00	7.28	0.96
'MAYM0100109	0.61	0.18	19.89	1.77	0.14	0.06	431013	0.00	6.08	0.97
'LYOV0200001	0.40	0.03	302.28	5.80	0.05	0.01	83665133	0.39	0.69	0.98
'CP014708.1'	0.80	0.10	40.07	1.75	0.05	0.02	2891404	0.00	2.79	0.98
'CP014584.1'	0.86	0.09	38.44	1.57	0.04	0.01	3325186	0.00	2.79	0.98
'CP016906.1'	0.13	0.16	17.27	1.06	0.48	0.21	4854065	8.49	7.88	0.99
'KV757199.1'	0.07	0.13	17.74	0.95	0.51	0.18	5253760	0.14	9.63	0.99
'KV757211.1'	0.42	0.15	23.35	1.49	0.21	0.09	1746732	3.76	5.65	0.98
'MBAC0100028	0.65	0.30	12.31	1.66	0.20	0.13	161542	0.00	8.76	0.96
'MBAD0100050	0.55	0.17	20.25	1.96	0.16	0.07	221611	0.00	6.32	0.97
'MBAB0100036	0.61	0.18	19.89	1.77	0.14	0.06	431008	0.00	6.08	0.97
'CM004594.1'	0.24	0.03	278.00	4.45	0.11	0.02	237214278	1.18	0.91	0.98
'MBDO0100000	0.69	0.18	18.15	1.50	0.13	0.06	429611	0.00	6.21	0.97
'KV757425.1'	0.61	0.18	19.90	1.77	0.14	0.06	431179	0.08	6.08	0.97
'CP013830.1'	0.00	-1.00	47.19	2.04	0.34	0.05	2554082	0.00	4.86	0.95
'CP016045.1'	0.82	0.16	13.61	0.77	0.12	0.05	1139168	0.01	7.51	0.99
'CP016047.1'	0.82	0.16	13.62	0.77	0.12	0.05	1139148	0.01	7.51	0.99
'CP016049.1'	0.82	0.16	13.61	0.78	0.12	0.05	1139067	0.22	7.49	0.99
'CP016050.1'	0.82	0.16	13.61	0.77	0.12	0.05	1139143	0.01	7.51	0.99
'CP016051.1'	0.82	0.16	13.61	0.77	0.12	0.05	1139126	0.01	7.51	0.99
'CP016052.1'	0.82	0.16	13.61	0.77	0.12	0.05	1139178	0.01	7.51	0.99
'CP016053.1'	0.82	0.16	13.61	0.77	0.12	0.05	1139145	0.01	7.51	0.99
'CP016054.1'	0.82	0.16	13.61	0.77	0.12	0.05	1138929	0.60	7.46	0.99
'CP016055.1'	0.82	0.16	13.61	0.77	0.12	0.05	1139184	0.01	7.51	0.99
'CP016056.1'	0.83	0.16	13.54	0.77	0.12	0.05	1139185	0.63	7.46	0.99
'CP016057.1'	0.82	0.16	13.61	0.77	0.12	0.05	1139159	0.01	7.51	0.99
'CP016058.1'	0.82	0.16	13.61	0.77	0.12	0.05	1139151	0.01	7.51	0.99
'CP016059.1'	0.82	0.16	13.61	0.77	0.12	0.05	1139129	0.01	7.51	0.99
'CP016060.1'	0.82	0.16	13.61	0.77	0.12	0.05	1139138	0.01	7.51	0.99
'CP016061.1'	0.82	0.16	13.61	0.77	0.12	0.05	1139146	0.01	7.51	0.99
'CP016062.1'	0.82	0.16	13.62	0.78	0.12	0.05	1139130	0.01	7.51	0.99
'CP016063.1'	0.82	0.16	13.61	0.77	0.12	0.05	1139123	0.01	7.51	0.99
'CP016064.1'	0.82	0.16	13.62	0.77	0.12	0.05	1139132	0.01	7.51	0.99
'CP016065.1'	0.82	0.16	13.62	0.78	0.12	0.05	1139131	0.01	7.51	0.99
'CP016066.1'	0.82	0.16	13.62	0.77	0.12	0.05	1139163	0.01	7.51	0.99
'CP016067.1'	0.82	0.16	13.62	0.77	0.12	0.05	1139135	0.01	7.51	0.99
'CP016068.1'	0.82	0.16	13.61	0.77	0.12	0.05	1139137	0.01	7.51	0.99
'CP016069.1'	0.82	0.16	13.61	0.77	0.12	0.05	1139158	0.01	7.51	0.99
'LZYO0100000	0.00	0.18	56.79	9.18	0.37	0.21	342430	0.26	4.29	0.93
'LYUC0100000	0.00	-1.00	61.62	4.10	0.30	0.05	718332	0.61	4.36	0.92
'MCQI0200013	0.12	0.14	32.77	2.32	0.36	0.16	2524342	0.00	5.58	0.98
'CP016975.1'	0.00	-1.00	16.12	0.81	0.57	0.12	2107069	0.02	9.86	0.96
'CP016985.1'	0.00	0.28	16.46	1.93	0.55	0.40	2126370	0.02	9.83	0.97
'CP016632.1'	0.44	0.10	59.42	3.40	0.11	0.04	2088090	4.19	2.57	0.97
'CP016633.1'	0.42	0.10	59.98	3.68	0.12	0.04	2069271	1.86	2.64	0.96
'CP017004.1'	0.50	0.06	283.65	18.22	0.04	0.01	3291235	0.72	0.63	0.91
'MCBO0100000	0.55	0.03	273.55	5.17	0.03	0.00	64048162	2.34	0.62	0.98
'MAZY0100000	0.00	-1.00	151.12	16.00	0.06	0.01	1836714	0.00	5.28	0.81
'KV766071.1'	0.31	0.16	20.33	1.43	0.28	0.12	1260241	0.00	7.31	0.98
'CP017074.1'	0.32	0.28	8.43	0.75	0.52	0.32	2992353	0.08	13.05	0.98
'JXNT0100000	0.00	-1.00	44.47	1.60	0.31	0.04	5851576	0.38	5.62	0.96
'CP016855.1'	0.60	0.09	53.09	2.55	0.07	0.02	2871469	0.00	2.55	0.97
'LHUA0100010	0.45	0.15	29.35	2.43	0.16	0.07	492973	0.04	4.94	0.97

'LHTZ0100002(	0.59	0.12	26.49	1.40	0.12	0.04	1491250	0.01	4.92	0.98
'LHTY0100008	0.39	0.13	29.95	1.75	0.19	0.07	2245598	0.00	4.98	0.98
'LHUB0100011	0.50	0.14	27.34	1.80	0.15	0.06	1526710	0.01	5.01	0.97
'LHUD0100019	0.30	0.15	32.40	2.56	0.22	0.10	1286584	0.02	4.97	0.97
'LHUC0100001	0.53	0.14	26.68	1.92	0.14	0.06	667238	0.01	5.03	0.97
'LJIJ01000001.	0.88	0.10	44.47	2.32	0.03	0.01	807113	0.00	2.40	0.97
'MDGX010000(	0.75	0.16	46.03	5.86	0.05	0.02	142479	0.00	2.64	0.91
'AWGH010000(	0.61	0.15	20.13	1.17	0.14	0.06	1690844	0.35	6.00	0.98
'AWGK010000(	0.67	0.15	32.55	2.44	0.08	0.04	839580	1.74	3.71	0.96
'AWGJ010000C	0.34	0.17	23.56	1.67	0.25	0.12	2227464	0.01	6.03	0.97
'MEKH010000C	0.43	0.17	22.43	1.57	0.21	0.10	2238178	0.58	5.99	0.97
'AWGL010000(	0.66	0.12	32.57	1.78	0.09	0.03	1977542	0.28	3.84	0.97
'KV766159.1'	0.26	0.12	27.27	1.37	0.28	0.10	5861214	0.11	5.83	0.98
'MEIZ0100000	0.00	-1.00	47.54	1.64	0.38	0.04	2517252	1.05	4.98	0.97
'CP012555.1'	0.00	-1.00	22.58	0.87	0.35	0.06	5419815	0.15	9.15	0.97
'CM004629.1'	0.06	0.05	187.89	5.83	0.24	0.06	37529183	6.92	1.47	0.98
'MDVP010000(	0.39	0.03	252.17	3.53	0.06	0.01	270692262	4.32	0.80	0.99
'MDVQ010000(	0.42	0.03	269.34	3.82	0.05	0.01	264820526	26.36	0.55	0.99
'MEDX0100001	0.00	-1.00	44.71	8.63	0.13	0.04	102742	0.00	10.93	0.82
'MEDY0100004	0.00	-1.00	21.34	1.36	0.39	0.07	337331	0.00	10.76	0.96
'CP016418.1'	0.83	0.11	32.21	1.65	0.05	0.02	1044343	2.52	3.29	0.98
'CP016420.1'	0.82	0.12	32.23	1.78	0.05	0.02	1044417	0.02	3.38	0.97
'CP016422.1'	0.83	0.11	32.05	1.72	0.05	0.02	1044415	0.75	3.35	0.98
'CP016424.1'	0.82	0.12	32.26	1.79	0.05	0.02	1044446	0.01	3.38	0.97
'CP016426.1'	0.82	0.11	32.33	1.77	0.06	0.02	1044377	0.04	3.38	0.97
'MINK0118759	0.10	0.14	387.35	218.42	0.17	0.08	116828	0.05	1.64	0.83
'MINJ01166222	0.15	0.16	156.38	41.90	0.16	0.09	691679	59.54	0.79	0.83
'MEHO010000(	0.00	-1.00	102.69	33.05	0.22	0.04	20690	0.00	5.97	0.86
'CM004674.1'	0.76	0.10	40.79	2.09	0.05	0.02	1502857	1.56	2.80	0.97
'CM004690.1'	0.76	0.10	40.89	2.10	0.05	0.02	1481369	5.45	2.69	0.97
'CM004706.1'	0.78	0.11	40.11	2.13	0.05	0.02	1507785	1.67	2.80	0.97
'CM004738.1'	0.74	0.11	41.43	2.23	0.06	0.02	1475394	0.25	2.84	0.97
'CM004722.1'	0.77	0.10	40.64	2.07	0.05	0.02	1497917	0.88	2.82	0.97
'CM004754.1'	0.77	0.10	40.36	2.08	0.05	0.02	1557401	0.18	2.85	0.97
'CM004770.1'	0.73	0.10	41.89	2.09	0.06	0.02	1478252	2.89	2.76	0.97
'CM004802.1'	0.79	0.10	39.66	2.00	0.05	0.02	1497563	2.51	2.78	0.97
'CM004786.2'	0.79	0.11	39.61	2.09	0.05	0.02	1399620	2.19	2.79	0.97
'CM004818.2'	0.78	0.11	39.94	2.23	0.05	0.02	1488466	2.99	2.76	0.97
'CM004834.2'	0.77	0.11	40.47	2.22	0.05	0.02	1516198	3.26	2.75	0.97
'CM004850.2'	0.77	0.11	40.12	2.13	0.05	0.02	1509076	1.03	2.82	0.97
'CM004866.2'	0.77	0.11	40.10	2.23	0.05	0.02	1466461	2.44	2.79	0.97
'CM004882.2'	0.77	0.11	40.02	2.12	0.05	0.02	1494703	1.55	2.81	0.97
'CM004898.1'	0.76	0.10	40.89	2.10	0.05	0.02	1481369	5.45	2.69	0.97
'CM004914.2'	0.76	0.11	40.45	2.16	0.05	0.02	1498560	1.58	2.81	0.97
'CM004930.2'	0.77	0.11	40.45	2.10	0.05	0.02	1670164	1.31	2.81	0.97
'CM004946.2'	0.76	0.11	40.35	2.21	0.05	0.02	1482814	1.91	2.79	0.97
'CM004962.2'	0.79	0.10	39.86	1.98	0.05	0.02	1463829	0.75	2.83	0.97
'CM004978.2'	0.75	0.11	40.88	2.23	0.05	0.02	1490065	1.51	2.81	0.97
'CM004994.2'	0.78	0.10	40.06	2.04	0.05	0.02	1494899	12.59	2.49	0.97
'CM005010.2'	0.76	0.11	40.59	2.15	0.05	0.02	1482753	1.51	2.81	0.97
'CM005026.2'	0.77	0.11	40.15	2.14	0.05	0.02	1494160	1.86	2.80	0.97
'CM005042.2'	0.78	0.10	40.12	2.07	0.05	0.02	1487422	7.19	2.65	0.97
'CM005058.2'	0.76	0.10	41.05	2.11	0.05	0.02	1472589	6.56	2.65	0.97
'CM005074.2'	0.76	0.11	40.41	2.20	0.05	0.02	1483962	2.03	2.79	0.97
'CM005090.1'	0.78	0.11	39.90	2.12	0.05	0.02	1485107	1.61	2.81	0.97
'CM005106.1'	0.80	0.10	39.49	1.96	0.05	0.02	1493346	1.82	2.80	0.97
'CM005122.1'	0.77	0.11	40.34	2.15	0.05	0.02	1491031	0.82	2.83	0.97
'CM005138.1'	0.78	0.11	39.80	2.23	0.05	0.02	1498679	2.08	2.79	0.97
'CM005154.1'	0.79	0.10	39.84	2.02	0.05	0.02	1503001	6.99	2.65	0.97
'CM005170.1'	0.78	0.11	40.06	2.13	0.05	0.02	1508833	6.06	2.68	0.97
'CM005186.1'	0.78	0.11	39.97	2.04	0.05	0.02	1488695	1.28	2.81	0.97
'CM005218.1'	0.78	0.11	40.01	2.26	0.05	0.02	1405954	3.04	2.76	0.97
'CM005202.1'	0.76	0.11	40.70	2.19	0.05	0.02	1493630	0.79	2.83	0.97
'CM005234.1'	0.79	0.11	39.71	2.07	0.05	0.02	1489640	1.44	2.81	0.97
'CM005250.2'	0.79	0.11	39.76	2.09	0.05	0.02	1526254	2.02	2.80	0.97
'CM005266.1'	0.77	0.11	40.58	2.28	0.05	0.02	1479350	1.32	2.81	0.97
'CM005282.1'	0.75	0.11	41.37	2.32	0.06	0.02	1451198	0.25	2.84	0.97
'CM005298.1'	0.76	0.11	40.74	2.24	0.05	0.02	1476497	2.56	2.78	0.97
'CM005314.1'	0.76	0.11	40.84	2.16	0.05	0.02	1489212	2.13	2.79	0.97
'CM005330.1'	0.77	0.10	40.66	2.14	0.05	0.02	1466691	1.16	2.81	0.97
'CM005346.1'	0.77	0.10	40.49	2.09	0.05	0.02	1483415	5.70	2.68	0.97
'CM005362.1'	0.76	0.11	40.98	2.25	0.05	0.02	1460824	0.88	2.82	0.97
'CM005378.1'	0.77	0.10	40.57	2.09	0.05	0.02	1539057	3.65	2.74	0.97
'CM005394.1'	0.80	0.10	39.68	1.90	0.05	0.02	1478964	6.02	2.67	0.98
'CM005426.1'	0.79	0.11	39.76	2.09	0.05	0.02	1401464	2.10	2.79	0.97
'CM005442.1'	0.76	0.10	40.88	2.15	0.05	0.02	1467517	3.54	2.74	0.97
'CM005410.1'	0.79	0.11	39.68	2.03	0.05	0.02	1480459	0.58	2.83	0.97
'CM005506.1'	0.76	0.11	40.63	2.15	0.05	0.02	1503833	1.14	2.82	0.97
'CM005458.1'	0.74	0.11	41.40	2.28	0.06	0.02	1498247	7.28	2.65	0.97
'CM005490.1'	0.77	0.11	40.28	2.10	0.05	0.02	1479048	2.11	2.79	0.97
'CM005474.1'	0.80	0.10	39.49	1.90	0.05	0.02	1475347	5.02	2.72	0.98
'CM005522.1'	0.73	0.11	41.73	2.44	0.06	0.02	1479259	4.69	2.71	0.97
'CM005538.1'	0.77	0.11	40.40	2.12	0.05	0.02	1472516	1.61	2.81	0.97
'CM005554.1'	0.75	0.11	40.73	2.25	0.05	0.02	1474803	1.89	2.80	0.97
'CM005570.1'	0.78	0.11	39.89	2.06	0.05	0.02	1501807	2.10	2.79	0.97
'CM005602.1'	0.77	0.10	40.46	2.10	0.05	0.02	1496075	2.27	2.79	0.97
'CM005586.1'	0.74	0.11	41.18	2.27	0.06	0.02	1485087	1.91	2.80	0.97
'CM005618.1'	0.74	0.11	41.30	2.36	0.06	0.02	1474444	1.87	2.80	0.97
'CM005634.1'	0.77	0.11	40.19	2.15	0.05	0.02	1494497	2.28	2.79	0.97
'CM005666.1'	0.80	0.10	39.38	1.98	0.05	0.02	1500138	2.39	2.78	0.97
'CM005650.1'	0.79	0.11	39.63	2.11	0.05	0.02	1491944	2.10	2.79	0.97
'CM005682.1'	0.76	0.10	40.69	2.04	0.05	0.02	1486039	2.09	2.79	0.97
'CM005698.1'	0.78	0.10	40.15	1.95	0.05	0.02	1490266	1.90	2.80	0.98
'CM005714.1'	0.82	0.10	38.76	1.86	0.05	0.02	1497068	1.29	2.81	0.98
'CM005730.1'	0.80	0.10	39.41	1.98	0.05	0.02	1494433	1.55	2.81	0.97
'CM005746.1'	0.75	0.11	40.67	2.23	0.05	0.02	1486401	2.00	2.79	0.97
'CM005762.1'	0.77	0.11	40.32	2.14	0.05	0.02	1487973	1.77	2.80	0.97
'CM005778.1'	0.78	0.11	39.96	2.14	0.05	0.02	1499290	1.64	2.80	0.97
'CM005794.1'	0.74	0.11	41.18	2.34	0.06	0.02	1481993	2.10	2.79	0.97
'CM005810.1'	0.81	0.10	39.06	1.92	0.05	0.02	1491577	1.13	2.82	0.97
'CM005826.1'	0.78	0.11	39.95	2.09	0.05	0.02	1500739	1.29	2.81	0.97
'CM005842.1'	0.79	0.11	39.60	2.09	0.05	0.02	1489994	1.84	2.79	0.97
'CM005858.1'	0.79	0.11	39.70	2.10	0.05	0.02	1476886	0.68	2.83	0.97
'CM005874.1'	0.75	0.11	40.92	2.28	0.06	0.02	1478102	1.86	2.80	0.97
'CM005890.1'	0.79	0.11	39.78	2.14	0.05	0.02	1493739	1.30	2.81	0.97
'CM005906.1'	0.78	0.11	39.97	2.08	0.05	0.02	1502977	1.63	2.81	0.97

'CM005922.1'	0.78	0.11	40.22	2.16	0.05	0.02	1479878	3.23	2.75	0.97
'CM005938.1'	0.79	0.11	39.78	2.12	0.05	0.02	1501536	1.46	2.81	0.97
'CM005954.1'	0.77	0.11	40.34	2.08	0.05	0.02	1501181	1.79	2.80	0.97
'CM005970.1'	0.76	0.11	40.65	2.17	0.05	0.02	1480531	0.68	2.83	0.97
'CM005986.1'	0.77	0.10	40.36	2.08	0.05	0.02	1506347	1.64	2.80	0.97
'CM006002.1'	0.76	0.10	40.68	1.98	0.05	0.02	1491306	2.20	2.79	0.98
'CM006018.1'	0.73	0.11	41.56	2.32	0.06	0.02	1480446	1.96	2.80	0.97
'CM006034.1'	0.76	0.11	40.61	2.17	0.05	0.02	1489654	2.11	2.80	0.97
'CM006050.1'	0.75	0.11	41.04	2.28	0.06	0.02	1469206	1.62	2.81	0.97
'CM006066.1'	0.76	0.11	40.60	2.20	0.05	0.02	1465851	1.15	2.82	0.97
'CM006082.1'	0.76	0.11	40.64	2.12	0.05	0.02	1491979	2.40	2.78	0.97
'CM006098.1'	0.74	0.11	41.18	2.39	0.06	0.02	1462430	1.41	2.82	0.97
'CM006114.1'	0.74	0.11	41.01	2.30	0.06	0.02	1457865	0.76	2.84	0.97
'CM006130.1'	0.75	0.11	40.78	2.20	0.05	0.02	1456980	0.40	2.84	0.97
'CM006146.1'	0.76	0.11	40.57	2.20	0.05	0.02	1493731	2.76	2.78	0.97
'CM006162.1'	0.76	0.11	40.81	2.14	0.05	0.02	1487608	1.77	2.80	0.97
'CM006178.1'	0.80	0.10	39.48	1.99	0.05	0.02	1488119	1.47	2.81	0.97
'CM006194.1'	0.80	0.11	39.38	2.03	0.05	0.02	1495720	1.54	2.81	0.97
'CM006210.1'	0.75	0.11	41.17	2.32	0.06	0.02	1488859	0.91	2.83	0.97
'CM006226.1'	0.77	0.11	40.29	2.14	0.05	0.02	1503394	1.64	2.80	0.97
'CM006242.1'	0.78	0.11	40.01	2.10	0.05	0.02	1503664	2.63	2.78	0.97
'CM006258.1'	0.76	0.11	40.75	2.29	0.05	0.02	1481267	2.03	2.79	0.97
'CM006274.1'	0.77	0.11	40.07	2.15	0.05	0.02	1488565	1.80	2.80	0.97
'CM006290.1'	0.78	0.11	39.96	2.15	0.05	0.02	1488480	1.97	2.80	0.97
'CM006306.1'	0.75	0.11	40.82	2.20	0.05	0.02	1488616	2.37	2.79	0.97
'CM006322.1'	0.78	0.11	40.02	2.15	0.05	0.02	1478880	1.35	2.81	0.97
'CM006338.1'	0.75	0.10	40.97	2.08	0.05	0.02	1482533	1.70	2.80	0.97
'CM006354.1'	0.76	0.11	40.54	2.25	0.05	0.02	1498948	2.72	2.78	0.97
'CM006370.1'	0.76	0.11	40.76	2.12	0.05	0.02	1480137	1.01	2.82	0.97
'CM006386.1'	0.75	0.11	40.96	2.17	0.06	0.02	1492939	2.06	2.79	0.97
'CM006402.1'	0.76	0.11	40.46	2.14	0.05	0.02	1491456	1.69	2.80	0.97
'CM006418.1'	0.78	0.11	39.97	2.13	0.05	0.02	1485149	2.00	2.80	0.97
'CM006434.1'	0.76	0.10	40.73	2.07	0.05	0.02	1480463	1.36	2.81	0.97
'CM006450.1'	0.74	0.11	41.26	2.31	0.06	0.02	1480784	1.49	2.81	0.97
'CM006466.1'	0.76	0.11	40.63	2.17	0.05	0.02	1482394	1.91	2.80	0.97
'CM006482.1'	0.76	0.11	40.30	2.14	0.05	0.02	1474736	2.43	2.79	0.97
'CM006498.1'	0.79	0.11	39.51	2.17	0.05	0.02	1489425	1.03	2.82	0.97
'CM006514.1'	0.74	0.11	41.35	2.28	0.06	0.02	1474865	4.11	2.73	0.97
'CM006530.1'	0.76	0.12	40.34	2.33	0.05	0.02	1568797	4.05	2.74	0.97
'CM006546.1'	0.78	0.11	40.12	2.11	0.05	0.02	1483708	1.85	2.80	0.97
'CM006562.1'	0.77	0.11	40.27	2.19	0.05	0.02	1490300	3.34	2.75	0.97
'CM006594.1'	0.77	0.11	40.60	2.23	0.05	0.02	1485192	0.90	2.82	0.97
'CM006578.1'	0.78	0.11	40.05	2.11	0.05	0.02	1473735	1.59	2.80	0.97
'CM006610.1'	0.76	0.11	40.90	2.20	0.05	0.02	1465691	4.20	2.72	0.97
'CM006626.1'	0.77	0.11	40.36	2.13	0.05	0.02	1496823	1.07	2.82	0.97
'CM006642.1'	0.78	0.11	39.88	2.14	0.05	0.02	1509760	4.05	2.73	0.97
'CM006674.1'	0.77	0.11	40.27	2.18	0.05	0.02	1494865	3.63	2.75	0.97
'CM006658.1'	0.77	0.10	40.20	2.06	0.05	0.02	1483138	1.06	2.82	0.97
'CM006690.1'	0.78	0.11	39.94	2.14	0.05	0.02	1489079	2.27	2.78	0.97
'CM006706.1'	0.77	0.10	40.50	2.08	0.05	0.02	1496762	2.28	2.79	0.97
'CM006738.1'	0.79	0.10	39.67	1.99	0.05	0.02	1490855	2.08	2.79	0.97
'CM006722.1'	0.81	0.11	39.29	2.05	0.05	0.02	1390203	1.83	2.79	0.97
'CM006754.1'	0.78	0.11	40.22	2.11	0.05	0.02	1494852	1.86	2.79	0.97
'CM006770.1'	0.78	0.11	39.85	2.11	0.05	0.02	1486763	0.94	2.82	0.97
'CM006802.1'	0.77	0.11	40.35	2.14	0.05	0.02	1494002	3.01	2.77	0.97
'CM006786.1'	0.78	0.11	39.83	2.11	0.05	0.02	1563978	5.71	2.69	0.97
'CM006818.1'	0.79	0.11	39.83	2.12	0.05	0.02	1385839	1.84	2.80	0.97
'CM006834.1'	0.77	0.10	40.57	2.10	0.05	0.02	1475989	2.83	2.76	0.97
'CM006850.1'	0.78	0.11	39.99	2.10	0.05	0.02	1475865	1.06	2.82	0.97
'CM006866.1'	0.75	0.11	41.08	2.31	0.06	0.02	1487406	4.53	2.72	0.97
'CM006882.1'	0.76	0.10	40.80	2.11	0.05	0.02	1483164	7.28	2.64	0.97
'CM006898.1'	0.79	0.10	39.70	2.03	0.05	0.02	1516113	3.15	2.76	0.97
'CM006930.1'	0.74	0.11	41.26	2.23	0.06	0.02	1509293	2.77	2.77	0.97
'CM006914.1'	0.76	0.11	41.01	2.21	0.05	0.02	1487136	4.07	2.73	0.97
'CM006946.1'	0.77	0.11	40.59	2.19	0.05	0.02	1460686	0.98	2.82	0.97
'CM006978.1'	0.77	0.11	40.46	2.13	0.05	0.02	1491316	2.33	2.79	0.97
'CM006962.1'	0.79	0.11	39.75	2.15	0.05	0.02	1488668	1.69	2.80	0.97
'CM006994.1'	0.74	0.11	41.41	2.28	0.06	0.02	1474583	1.67	2.80	0.97
'CM007010.1'	0.79	0.11	39.64	2.04	0.05	0.02	1490017	3.30	2.75	0.97
'CM007026.1'	0.79	0.09	40.26	1.89	0.05	0.01	1503105	13.26	2.47	0.98
'CM007042.1'	0.75	0.10	40.88	2.10	0.05	0.02	1477786	1.01	2.82	0.97
'CM007058.1'	0.75	0.11	41.40	2.37	0.06	0.02	1383966	4.62	2.71	0.97
'CM007074.1'	0.75	0.11	40.77	2.26	0.05	0.02	1469751	1.86	2.80	0.97
'CM007090.1'	0.77	0.10	40.46	2.05	0.05	0.02	1502146	1.79	2.80	0.97
'CM007106.1'	0.83	0.10	38.58	1.92	0.05	0.02	1494304	9.31	2.59	0.97
'CM007122.1'	0.78	0.11	39.84	2.08	0.05	0.02	1497472	1.35	2.81	0.97
'CM007138.1'	0.75	0.10	41.09	2.12	0.06	0.02	1480572	3.56	2.75	0.97
'CM007154.1'	0.74	0.10	41.37	2.16	0.06	0.02	1471902	1.28	2.81	0.97
'CM007170.1'	0.78	0.11	39.96	2.13	0.05	0.02	1471608	0.68	2.83	0.97
'LMYC0100031	0.27	0.16	30.91	2.63	0.25	0.11	793829	0.04	5.44	0.97
'KV766587.1'	0.23	0.08	95.34	5.54	0.16	0.05	2967516	0.06	2.31	0.96
'BDCQ0100001	0.00	0.11	169.60	23.71	0.26	0.10	641516	0.46	2.26	0.92
'BDGN0100208	0.78	0.22	17.93	2.43	0.10	0.05	87685	0.00	6.00	0.95
'DF977685.1'	0.11	0.13	31.16	1.73	0.38	0.15	7344524	0.03	5.51	0.98
'DF973114.1'	0.18	0.07	163.78	10.47	0.17	0.05	2878652	3.91	1.49	0.95
'MDVK0100000	0.85	0.11	47.49	3.06	0.04	0.01	504328	0.00	2.33	0.96
'CP017298.1'	0.16	0.27	9.18	0.87	0.63	0.36	2141493	2.86	13.29	0.99
'MDLI0100000	0.39	0.04	398.42	12.81	0.05	0.01	22945223	2.02	0.53	0.95
'CP014715.1'	0.80	0.10	40.05	1.74	0.05	0.02	2887587	0.00	2.79	0.98
'LPNM0100000	0.44	0.11	69.84	4.53	0.10	0.04	1932387	0.84	2.26	0.95
'LPNN0100000	0.67	0.10	116.70	8.41	0.03	0.01	1922431	0.03	1.19	0.91
'LPNL0100000	0.64	0.10	110.27	8.23	0.04	0.01	1912120	0.03	1.29	0.91
'MKHR010393	0.29	0.22	93.89	45.48	0.14	0.08	25455	7.35	3.08	0.84
'KV784179.1'	0.00	-1.00	46.91	1.78	0.34	0.05	5141995	0.91	4.95	0.95
'KV784220.1'	0.14	0.13	35.40	2.06	0.33	0.13	4666273	0.04	5.14	0.98
'KV878785.1'	0.45	0.11	30.96	1.63	0.16	0.05	1693895	0.44	4.83	0.98
'KV784353.1'	0.00	0.14	97.49	8.86	0.18	0.10	5926375	1.68	3.61	0.92
'KV784802.1'	0.35	0.03	276.00	4.94	0.07	0.01	113921103	1.76	0.79	0.98
'MCNH010000	0.64	0.12	45.12	3.52	0.07	0.03	552249	0.38	2.90	0.96
'CP017312.1'	0.00	-1.00	44.40	1.30	0.41	0.04	4217945	0.01	4.90	0.97
'CP017313.1'	0.00	0.19	38.68	3.81	0.44	0.27	4217957	0.04	4.74	0.95
'MEJB0100000	0.33	0.06	120.06	4.04	0.11	0.03	16402300	5.32	1.56	0.98
'LWKS010015	0.19	0.16	89.53	19.76	0.19	0.09	120495	0.30	2.93	0.90
'LSBY0100000	0.58	0.27	16.48	2.45	0.17	0.11	109425	0.00	7.27	0.95
'JXYK0100000	0.31	0.15	14.95	0.85	0.33	0.13	4840925	0.32	9.44	0.99
'JXYL0100000	0.30	0.15	15.59	0.90	0.34	0.13	2459564	0.58	9.11	0.99

'JXYM0100000	0.00	-1.00	15.95	0.52	0.57	0.08	3669090	0.48	10.86	0.98
'JXYN0100000	0.25	0.12	13.83	0.63	0.40	0.12	3718887	0.04	10.42	0.99
'CP017448.1'	0.00	-1.00	17.33	0.74	0.37	0.07	3363634	0.18	11.37	0.97
'MKEJ0110461	0.83	0.20	28.04	3.53	0.06	0.03	130601	0.00	3.83	0.93
'MDSA010000C	0.88	0.12	27.83	1.45	0.05	0.02	1746572	0.00	3.73	0.98
'MDSB010000C	0.58	0.11	46.36	2.58	0.08	0.03	1824870	0.05	2.93	0.97
'MEHF010000C	0.37	0.13	30.51	1.75	0.20	0.08	2918844	0.64	4.93	0.98
'CP011148.1'	0.72	0.09	62.21	3.52	0.04	0.01	1267840	3.53	1.96	0.96
'CP011149.1'	0.66	0.09	66.53	3.93	0.05	0.02	1267664	9.83	1.82	0.96
'CP017557.1'	0.37	0.12	31.43	1.59	0.19	0.07	4198534	0.00	4.81	0.98
'CP016046.1'	0.82	0.16	13.61	0.77	0.12	0.05	1139160	0.01	7.51	0.99
'CP016048.1'	0.82	0.16	13.61	0.77	0.12	0.05	1139139	0.01	7.51	0.99
'MJCC0100000	0.00	-1.00	46.76	1.53	0.31	0.03	2034363	2.41	6.17	0.98
'CP017126.1'	0.66	0.09	199.84	20.00	0.02	0.01	907278	0.00	0.79	0.86
'LXJU0100000	0.38	0.12	29.06	1.60	0.20	0.07	2696425	0.02	5.29	0.98
'MJBS0100000	0.00	0.15	54.56	5.85	0.34	0.16	844029	0.00	4.88	0.95
'LQCH0100000	0.00	-1.00	308.67	40.98	0.01	0.00	17084797	0.03	5.14	0.67
'LRBQ0100001	0.43	0.08	157.07	12.21	0.07	0.02	1292604	0.00	1.17	0.92
'LRBR0100019	0.58	0.11	164.79	26.07	0.04	0.01	278257	0.00	1.12	0.83
'LRBT0100022	0.47	0.09	324.46	60.29	0.04	0.02	434208	0.00	0.78	0.80
'LRBU0100130	0.55	0.14	>1000	>1000	0.03	0.01	63770	0.00	0.88	0.49
'LPXJ0100713	0.29	0.07	186.49	11.11	0.10	0.03	3259308	2.14	1.20	0.95
'MLAK0100000	0.32	0.12	96.70	11.58	0.12	0.05	406868	1.19	2.15	0.92
'MIMC0100000	0.26	0.09	55.72	2.53	0.20	0.06	6301587	1.95	3.28	0.98
'MBSD020004	0.38	0.19	26.39	3.31	0.20	0.10	186500	0.00	5.86	0.96
'CP017712.1'	0.13	0.20	23.37	2.26	0.39	0.22	2098685	12.76	6.21	0.97
'CP017708.1'	0.13	0.09	60.82	2.92	0.29	0.10	9373345	0.28	3.26	0.98
'CP015054.1'	0.53	0.11	46.88	2.72	0.10	0.03	1734505	0.00	3.12	0.97
'MAMS010105	0.00	-1.00	73.25	1.71	0.30	0.02	11178615	1.03	3.90	0.98
'KV841770.1'	0.11	0.16	29.49	2.61	0.37	0.18	1056626	0.44	6.25	0.97
'JUFY0103446	0.23	0.11	118.41	14.76	0.15	0.06	419800	0.06	2.05	0.92
'CM007353.1'	0.44	0.09	64.05	3.31	0.11	0.03	2642169	1.23	2.46	0.97
'KV844967.1'	0.00	0.09	189.90	18.29	0.28	0.10	2152292	7.95	1.62	0.94
'LXKZ0100007	0.41	0.14	35.11	3.05	0.16	0.07	483719	0.06	4.59	0.96
'LXJY0100000	0.00	-1.00	123.39	4.82	0.26	0.02	2199663	0.39	2.82	0.95
'LWME010001	0.00	0.11	105.34	8.56	0.29	0.13	3256488	1.40	2.69	0.94
'KV860272.1'	0.00	-1.00	318.66	3.79	0.25	0.01	84051066	3.13	1.17	0.98
'JRPV0100099	0.26	0.19	29.81	2.83	0.27	0.15	1140195	4.57	4.97	0.96
'CP017814.1'	0.00	-1.00	68.76	2.52	0.35	0.04	3951982	0.00	3.50	0.95
'CM007488.1'	0.28	0.04	139.58	3.07	0.12	0.02	62059223	0.35	1.59	0.98
'JPRY0100172	0.40	0.09	906.16	433.15	0.04	0.02	271026	0.00	0.68	0.73
'JPRZ0100000	0.57	0.10	>1000	840.44	0.02	0.01	164654	0.10	0.64	0.56
'JPSA0100000	0.43	0.10	>1000	>1000	0.04	0.01	232970	0.00	0.65	0.65
'JPSE0100720	0.85	0.18	>1000	>1000	0.02	0.01	24583	0.23	0.76	0.18
'JPSI01001686	0.50	0.08	368.44	55.27	0.03	0.01	645552	0.00	0.65	0.81
'JPSC0100235	0.66	0.11	757.09	461.45	0.02	0.01	157437	0.02	0.62	0.50
'LRBP0100001	0.41	0.09	165.34	15.62	0.07	0.02	890313	0.00	1.19	0.91
'LRBS0100006	0.54	0.09	207.10	23.04	0.04	0.01	587635	0.00	0.91	0.87
'CM007361.1'	0.00	0.05	247.62	8.46	0.30	0.07	36457581	0.69	1.25	0.97
'MODV010048	0.00	0.13	250.04	95.01	0.25	0.11	131537	1.36	2.16	0.88
'CP017675.1'	0.24	0.15	28.98	2.02	0.26	0.12	3049282	1.72	5.51	0.97
'MIEF0100922	0.68	0.11	33.50	1.75	0.08	0.03	1802680	0.00	3.74	0.98
'MOXI0100000	0.54	0.04	237.67	4.75	0.03	0.01	54145758	0.27	0.71	0.98
'MPCS010000	0.32	0.15	28.85	2.11	0.23	0.10	1666775	6.75	5.12	0.97
'CP017964.1'	0.27	0.21	13.15	0.91	0.43	0.23	7247640	10.24	8.47	0.98
'MKPU010000C	0.73	0.25	15.28	1.94	0.13	0.08	85208	0.00	6.94	0.96
'CM007387.1'	0.00	-1.00	189.93	3.07	0.12	0.01	151357931	2.93	2.77	0.96
'CP017979.1'	0.00	-1.00	27.75	0.95	0.40	0.05	4541211	0.00	7.46	0.97
'CP017951.1'	0.00	0.20	14.19	1.07	0.68	0.35	5758958	7.69	9.74	0.99
'CM007397.1'	0.00	-1.00	185.95	4.22	0.10	0.01	114765206	15.97	2.33	0.94
'KV875093.1'	0.00	0.19	35.80	3.27	0.31	0.19	4640074	0.18	6.65	0.96
'BDFN0100104	0.00	-1.00	174.29	3.09	0.26	0.01	14449934	0.00	2.11	0.98
'KV875228.1'	0.00	-1.00	170.70	27.73	0.03	0.01	5148149	0.44	5.77	0.70
'LVVM0100267	0.82	0.17	19.48	1.50	0.09	0.04	446864	0.51	5.34	0.97
'CM007422.1'	0.13	0.03	343.23	6.86	0.16	0.03	123854990	13.24	0.80	0.98
'LGTZ0100000	0.51	0.18	30.75	4.24	0.13	0.06	71402	0.00	4.81	0.95
'LGRN0100000	0.20	0.17	41.70	5.22	0.25	0.13	267908	0.00	4.49	0.95
'MNUE010000C	0.32	0.16	17.90	1.20	0.29	0.13	2090884	0.00	8.21	0.98
'JRZF0100109	0.55	0.17	33.92	3.75	0.11	0.06	292464	6.17	3.85	0.95
'MKKT0100000	0.00	-1.00	331.95	77.11	0.16	0.02	134516	0.00	2.84	0.86
'MKLA0200024	0.63	0.11	123.57	15.33	0.04	0.01	344535	0.16	1.28	0.87
'LYUU0100208	0.73	0.11	67.00	4.47	0.04	0.02	2798100	0.02	1.85	0.94
'CP015518.1'	0.00	-1.00	22.48	1.06	0.38	0.07	3176363	0.00	8.73	0.96
'CM007505.1'	0.31	0.04	198.28	3.57	0.10	0.02	126111278	1.22	1.10	0.99
'CM007525.1'	0.48	0.03	232.71	3.19	0.05	0.01	202061739	1.24	0.76	0.99
'MIQA0105964	0.50	0.19	26.97	2.87	0.15	0.08	305893	0.51	5.03	0.96
'CM007575.1'	0.14	0.03	347.56	7.24	0.15	0.03	123855249	16.90	0.75	0.98
'LVEH0100003	0.22	0.05	203.94	6.92	0.13	0.03	18681901	0.84	1.14	0.97
'MPIL01001537	0.37	0.13	30.53	1.87	0.20	0.08	2452528	0.01	4.90	0.98
'CP018148.1'	0.18	0.13	45.83	3.16	0.26	0.11	3054454	0.02	4.02	0.97
'CP018149.1'	0.23	0.12	43.92	2.72	0.24	0.09	2991300	0.76	3.99	0.97
'CM007596.1'	0.00	-1.00	113.56	1.86	0.17	0.01	44746683	0.00	4.23	0.98
'KV878679.1'	0.05	0.12	36.68	2.10	0.40	0.15	4536051	0.18	5.48	0.98
'JXIK01000001	0.08	0.05	235.95	10.52	0.21	0.05	12387654	9.10	1.13	0.96
'KV878336.1'	0.00	-1.00	55.33	2.38	0.19	0.03	3522795	0.15	6.27	0.94
'KV878125.1'	0.12	0.15	29.79	1.91	0.39	0.18	5023850	0.01	5.75	0.98
'KV878236.1'	0.00	0.26	147.43	32.14	0.04	0.05	4264968	0.03	5.30	0.75
'KV878582.1'	0.00	-1.00	89.29	10.19	0.06	0.02	5537812	0.02	5.79	0.78
'KV878209.1'	0.00	0.17	48.32	4.01	0.31	0.18	5070162	0.06	5.00	0.95
'KV878176.1'	0.00	-1.00	134.71	15.08	0.05	0.01	4803603	0.01	5.35	0.77
'KV878888.1'	0.00	-1.00	51.02	2.77	0.26	0.04	1831800	0.86	5.39	0.93
'KV878970.1'	0.05	0.13	32.63	1.96	0.42	0.16	3727362	0.77	6.02	0.98
'KV880534.1'	0.13	0.08	65.78	2.72	0.26	0.07	9810584	5.21	3.37	0.98
'CP015758.1'	0.58	0.09	56.08	2.77	0.07	0.02	2754198	0.01	2.46	0.97
'MNAD010015	0.64	0.22	11.71	1.01	0.20	0.10	367072	0.01	9.54	0.98
'MPSW010005	0.00	-1.00	189.12	18.92	0.21	0.02	495086	17.65	2.15	0.89
'MIKG0100000	0.39	0.11	27.88	1.31	0.20	0.07	3931711	0.00	5.24	0.99
'MRJL0100000	0.52	0.13	23.21	1.18	0.16	0.06	3755581	2.10	5.45	0.98
'CP018262.1'	0.68	0.09	148.73	12.87	0.03	0.01	905394	0.00	0.97	0.89
'MJAE0100000	0.62	0.16	20.87	1.42	0.13	0.06	1097294	1.21	5.63	0.98
'LFMY0100000	0.00	0.28	193.93	57.06	0.04	0.04	3269259	0.02	5.05	0.69
'CP017834.1'	0.09	0.09	102.00	6.72	0.26	0.09	3342382	0.55	2.39	0.96
'MKXE010000C	0.00	-1.00	346.32	29.18	0.24	0.02	871187	1.30	1.30	0.89
'MNBE0100071	0.31	0.14	31.12	2.28	0.22	0.09	984881	0.09	5.29	0.97
'CP017012.1'	0.00	-1.00	16.44	0.81	0.54	0.11	2109434	0.02	9.87	0.96
'CP018743.1'	0.06	0.20	15.03	1.05	0.60	0.32	6267580	0.07	9.60	0.98

'CP018615.1'	0.44	0.16	28.33	2.07	0.17	0.08	1953716	0.01	5.06	0.97
'LHCJ01S0172	0.11	0.14	594.30	599.96	0.16	0.07	76066	1.21	1.72	0.82
'LCWK01S013	0.11	0.16	271.27	174.50	0.18	0.09	67078	0.68	2.17	0.83
'LHVS01S0000	0.22	0.07	119.77	5.75	0.15	0.04	4870262	9.08	1.81	0.97
'KV880664.1'	0.23	0.14	24.04	1.42	0.31	0.12	2087327	0.00	6.62	0.98
'MLYV0100262	0.85	0.15	17.52	1.11	0.09	0.03	443622	0.03	5.79	0.98
'CM007615.1'	0.00	-1.00	35.23	1.12	0.37	0.04	4246687	0.07	6.35	0.97
'MSDY010001	0.66	0.16	20.75	1.49	0.12	0.05	763196	0.07	5.67	0.98
'MSDX010028	0.67	0.22	17.38	1.88	0.13	0.07	165144	0.07	6.53	0.96
'LXTM0100003	0.27	0.12	83.22	8.96	0.15	0.06	521601	6.05	2.47	0.94
'CP018861.1'	0.05	0.12	61.83	4.30	0.36	0.15	4142132	0.00	3.37	0.96
'CP018810.1'	0.00	-1.00	27.74	1.02	0.34	0.05	5098116	0.00	7.82	0.97
'MRVE010007	0.57	0.19	14.79	1.15	0.20	0.09	732016	0.00	7.97	0.98
'MRVK010016	0.12	0.04	319.32	7.04	0.16	0.03	96776607	0.49	0.97	0.98
'LIEC0101215	0.37	0.12	78.87	8.65	0.11	0.05	405366	0.02	2.43	0.93
'LXFE010001	0.78	0.19	25.87	2.95	0.07	0.04	131778	0.00	4.38	0.95
'CP016908.1'	0.42	0.13	30.65	1.93	0.17	0.07	1821632	0.00	4.72	0.98
'MPVS010000	0.60	0.16	14.39	0.90	0.19	0.07	1100126	0.04	8.05	0.99
'MSJD0100015	0.18	0.14	32.78	2.43	0.30	0.13	1390245	0.01	5.49	0.97
'CP015998.1'	0.43	0.12	41.07	2.31	0.14	0.06	2711602	0.09	3.57	0.97
'MSJG0100094	0.27	0.17	22.97	1.96	0.29	0.13	669699	0.01	7.10	0.98
'MSJH0100266	0.00	-1.00	132.86	21.19	0.08	0.02	1034423	0.05	5.25	0.77
'MSJI0100194	0.70	0.17	17.71	1.48	0.13	0.05	262869	0.01	6.40	0.98
'MSJE0100022	0.10	0.16	28.34	2.36	0.39	0.18	1348435	0.00	6.52	0.97
'MSJJ010009	0.00	-1.00	79.39	8.30	0.12	0.03	2546576	0.01	4.88	0.82
'MSJK0100000	0.00	-1.00	40.69	1.81	0.38	0.06	2436850	0.00	5.41	0.95
'MSHP010001	0.82	0.12	37.10	2.35	0.05	0.02	664174	0.84	2.97	0.96
'CP010584.1'	0.00	-1.00	22.34	1.07	0.26	0.06	5506053	0.04	10.52	0.95
'MNCL010000	0.35	0.09	53.67	2.45	0.15	0.05	4070316	0.00	3.28	0.98
'KV881464.1'	0.49	0.24	15.84	1.82	0.22	0.13	218711	0.48	7.82	0.97
'KV881039.1'	0.61	0.18	16.08	1.47	0.17	0.07	170823	0.02	7.41	0.98
'MLIX0100000	0.63	0.23	15.29	1.66	0.17	0.09	233428	0.00	7.58	0.97
'KV881840.1'	0.54	0.23	15.40	1.66	0.20	0.11	216826	0.05	7.83	0.97
'KV882206.1'	0.50	0.25	15.77	1.95	0.22	0.13	219531	0.44	7.82	0.96
'MLJC0100000	0.62	0.20	16.14	1.63	0.17	0.08	151937	0.00	7.32	0.97
'MLJD0100000	0.58	0.22	15.78	1.66	0.18	0.09	265853	0.00	7.62	0.97
'KV883196.1'	0.57	0.25	15.00	1.87	0.20	0.11	161871	0.09	7.96	0.96
'KV883521.1'	0.60	0.17	16.30	1.43	0.17	0.07	170813	0.20	7.40	0.98
'MLJG0100000	0.62	0.23	15.36	1.65	0.17	0.09	266307	0.00	7.60	0.97
'CP013853.1'	0.70	0.09	90.45	4.98	0.03	0.01	1877932	0.01	1.43	0.95
'CP012809.1'	0.52	0.09	54.37	2.41	0.09	0.03	4911352	0.00	2.69	0.98
'CP012823.1'	0.52	0.09	54.55	2.51	0.09	0.03	4889271	0.00	2.69	0.97
'CP015978.1'	0.53	0.09	54.22	2.37	0.09	0.03	4909981	0.10	2.69	0.98
'MKXH010054	0.48	0.11	151.45	22.84	0.06	0.02	327144	0.00	1.30	0.86
'MPUG010000	0.49	0.29	9.73	1.18	0.33	0.20	201307	0.16	11.48	0.98
'MPGH010000	0.00	-1.00	36.59	2.00	0.29	0.06	2381854	0.09	6.58	0.93
'LOQR010000	0.81	0.10	39.10	1.95	0.05	0.02	1465478	0.00	2.85	0.97
'MLJV0100000	0.00	-1.00	21.05	0.92	0.41	0.07	997273	0.01	10.05	0.97
'MQRK012180	0.15	0.12	143.87	26.01	0.18	0.08	267531	8.35	1.94	0.91
'MPKX012863	0.24	0.84	16.09	16.15	0.34	0.44	3569	0.00	11.01	0.80
'MQRL016479	0.49	0.16	627.48	924.97	0.05	0.02	36739	1.43	1.37	0.62
'LSRX0100000	0.00	0.12	65.39	5.04	0.32	0.14	3144590	5.53	3.91	0.96
'MJVA0100102	0.00	-1.00	41.06	1.91	0.38	0.06	1690671	0.02	5.51	0.95
'MKKQ010045	0.68	0.18	11.61	0.84	0.19	0.08	427429	0.45	9.30	0.99
'CM007619.1'	0.00	-1.00	11.69	0.53	0.66	0.15	5198476	0.00	12.20	0.97
'CM007622.1'	0.00	-1.00	11.46	0.53	0.68	0.16	5167049	0.00	12.23	0.97
'CP010422.1'	0.82	0.16	13.61	0.77	0.12	0.05	1139844	0.04	7.51	0.99
'CP016401.1'	0.35	0.21	8.47	0.54	0.48	0.22	4324961	0.01	12.73	0.99
'DF978496.1'	0.15	0.07	93.48	3.49	0.22	0.06	11795022	1.76	2.46	0.98
'DF980259.1'	0.16	0.07	91.45	4.44	0.21	0.06	3939554	2.04	2.58	0.98
'BDGG010000	0.77	0.10	19.08	0.63	0.10	0.03	9333084	0.89	5.58	0.99
'DF983528.1'	0.78	0.12	40.65	2.41	0.05	0.02	1375271	2.37	2.76	0.96
'BCGG010000	0.00	-1.00	31.08	1.15	0.46	0.07	4317362	0.48	6.09	0.96
'BCGH010000	0.00	0.31	69.56	12.71	0.07	0.08	6486390	0.08	6.59	0.80
'BCJR0100000	0.45	0.17	21.28	1.61	0.20	0.09	977895	5.71	5.88	0.98
'BCJS0100000	0.00	0.28	72.75	13.88	0.10	0.10	2076502	2.01	5.76	0.83
'BCKA0100000	0.00	-1.00	47.22	1.61	0.37	0.05	6574720	0.22	4.75	0.96
'BCKB0100000	0.56	0.14	24.56	1.41	0.13	0.06	2842153	0.84	5.12	0.98
'BCFM010000	0.28	0.22	15.21	1.71	0.36	0.18	209930	0.36	9.46	0.98
'BCFN0100000	0.24	0.24	14.25	1.82	0.39	0.21	182581	0.29	10.35	0.98
'BCFO0100000	0.25	0.22	14.55	1.62	0.38	0.19	411794	0.10	10.18	0.98
'BCFP0100000	0.53	0.28	16.38	3.23	0.19	0.11	30744	1.48	7.79	0.95
'BCFQ0100000	0.31	0.22	14.32	1.72	0.34	0.16	142967	1.53	9.80	0.98
'BCFR0100000	0.04	0.34	19.98	5.45	0.48	0.34	55800	1.40	9.43	0.94
'BCFS0100000	0.26	0.29	13.83	2.09	0.39	0.25	111336	0.16	10.23	0.97
'BCFT0100000	0.01	0.32	16.48	3.11	0.58	0.41	150908	0.13	10.51	0.96
'BCFU0100000	0.25	0.25	14.13	1.82	0.39	0.21	182337	0.29	10.36	0.98
'KV884608.1'	0.24	0.12	33.04	2.02	0.26	0.09	1569905	0.53	5.32	0.98
'CP019059.1'	0.47	0.13	22.78	1.19	0.19	0.07	3013583	0.00	5.70	0.98
'CP013857.1'	0.41	0.06	305.10	17.83	0.05	0.01	3982791	0.00	0.68	0.93
'MQVQ010000	0.00	-1.00	269.79	48.29	0.02	0.00	2214490	1.11	6.10	0.70
'CM007627.1'	0.00	-1.00	113.95	1.74	0.18	0.01	44711178	0.02	4.15	0.98
'KV884691.1'	0.42	0.05	172.07	5.41	0.06	0.01	11676166	5.03	1.07	0.97
'LSSL010001	0.53	0.12	132.67	21.37	0.05	0.02	204087	1.07	1.43	0.86
'KV886423.1'	0.50	0.26	15.74	2.00	0.22	0.14	219297	0.89	7.78	0.96
'MSTJ0100000	0.67	0.15	21.07	1.40	0.12	0.05	694821	0.02	5.55	0.98
'CP019153.1'	0.00	-1.00	8.55	0.34	0.75	0.16	4590718	27.67	11.45	0.98
'MAYC010000	0.62	0.17	18.73	1.29	0.15	0.07	1366164	0.81	6.19	0.98
'CM007645.1'	0.33	0.22	8.56	0.58	0.50	0.24	4408292	0.26	12.71	0.99
'CM007646.1'	0.33	0.22	8.56	0.58	0.50	0.24	4408238	0.26	12.71	0.99
'CP019159.1'	0.42	0.16	19.63	1.30	0.23	0.10	2435437	0.01	6.75	0.98
'CP019289.1'	0.39	0.16	19.95	1.34	0.24	0.11	2320434	0.02	6.75	0.98
'MPLB0100000	0.44	0.05	112.41	3.21	0.08	0.02	23627241	18.42	1.33	0.98
'LSSN0100000	0.39	0.12	110.89	17.39	0.09	0.04	206626	0.26	1.92	0.90
'LSSM0100774	0.16	0.13	485.25	329.63	0.13	0.06	120745	0.00	1.52	0.81
'BCHE0100000	0.00	-1.00	31.89	1.13	0.46	0.07	5810324	0.45	6.00	0.97
'BCII01000001	0.76	0.13	18.75	0.82	0.11	0.04	6229779	1.03	5.61	0.99
'BDDD010000	0.00	0.09	128.80	9.99	0.31	0.10	2219130	0.98	2.40	0.96
'BCLB0100000	0.00	0.18	380.35	114.16	0.04	0.03	1986796	9.54	3.64	0.74
'BDGX010000	0.80	0.12	37.32	2.23	0.05	0.02	1474404	0.12	3.01	0.97
'BCLT0100000	0.32	0.15	15.40	0.89	0.32	0.13	4053335	0.29	9.11	0.99
'MPGU010001	0.26	0.09	86.23	4.62	0.16	0.05	4117863	6.10	2.24	0.97
'MECZ010000	0.00	-1.00	9.51	0.44	0.44	0.10	3991583	0.00	17.95	0.98
'AWWV010108	0.00	0.12	266.90	69.63	0.25	0.11	342011	0.00	1.75	0.88
'MTPI0100004	0.60	0.15	22.93	1.77	0.13	0.06	805677	42.71	3.14	0.97
'CP019435.1'	0.28	0.12	70.77	6.10	0.16	0.07	1344557	0.01	2.70	0.94

'CP019434.1'	0.00	-1.00	13.79	0.57	0.58	0.11	3448835	0.17	11.62	0.98
'MSZU0100007	0.09	0.16	20.61	1.33	0.47	0.21	4877926	0.92	8.21	0.98
'CP019124.1'	0.31	0.27	9.02	0.78	0.51	0.31	3192699	0.30	12.41	0.98
'CP019093.1'	0.00	-1.00	209.15	7.04	0.09	0.01	14772968	7.49	3.13	0.93
'MQVM010000	0.68	0.11	52.67	3.65	0.06	0.02	782669	0.01	2.46	0.95
'MIPV0100804	0.00	-1.00	60.95	8.28	0.34	0.06	84164	6.74	4.34	0.89
'MEHY0100001	0.78	0.16	18.71	1.40	0.10	0.04	276830	0.00	5.73	0.98
'CP019420.1'	0.76	0.19	7.25	0.36	0.25	0.11	6407860	0.00	12.33	0.99
'CP019490.1'	0.68	0.11	33.76	1.69	0.08	0.03	2110500	0.00	3.62	0.98
'MTKA0100063	0.11	0.05	351.06	23.07	0.16	0.04	4235486	0.01	0.98	0.94
'CP011180.1'	0.00	-1.00	10.92	0.51	0.73	0.17	4106424	0.00	13.18	0.97
'CP015999.1'	0.00	-1.00	9.54	0.36	0.69	0.13	3241870	0.00	15.32	0.99
'CP018344.1'	0.76	0.08	99.74	5.71	0.03	0.01	1656133	0.01	1.24	0.94
'CP018345.1'	0.76	0.08	100.21	5.77	0.03	0.01	1657699	0.01	1.25	0.94
'CP018346.1'	0.76	0.08	99.82	5.73	0.03	0.01	1656322	0.01	1.24	0.94
'CP011458.1'	0.34	0.14	28.16	1.68	0.23	0.10	3468040	14.45	4.43	0.98
'CP010036.1'	0.14	0.14	32.76	2.22	0.36	0.16	3060958	6.69	4.82	0.97
'CP011466.1'	0.34	0.15	27.49	1.71	0.23	0.10	3190451	11.65	4.63	0.98
'CP010046.1'	0.12	0.15	33.37	2.35	0.37	0.17	3060867	6.45	4.82	0.97
'CP010034.1'	0.13	0.15	32.98	2.27	0.36	0.16	3060738	6.45	4.82	0.97
'CP011470.1'	0.15	0.15	32.61	2.27	0.35	0.16	3060857	9.78	4.65	0.97
'CP010032.1'	0.17	0.14	32.12	2.06	0.33	0.14	3060702	3.99	4.96	0.98
'CP010030.1'	0.13	0.14	33.04	2.10	0.36	0.15	3060902	3.52	4.97	0.98
'CP010040.1'	0.13	0.14	33.03	2.10	0.36	0.15	3060815	3.57	4.97	0.98
'CP011462.1'	0.28	0.14	30.08	1.88	0.25	0.11	3440457	9.06	4.64	0.98
'CP010042.1'	0.13	0.15	32.97	2.29	0.36	0.16	3060835	6.36	4.83	0.97
'CP011468.1'	0.13	0.14	33.15	2.24	0.36	0.16	3060915	6.64	4.81	0.97
'CP011472.1'	0.23	0.15	30.48	1.99	0.29	0.13	2979966	3.06	5.00	0.98
'CP010038.1'	0.14	0.15	32.79	2.24	0.35	0.16	3060807	3.29	4.98	0.97
'CP010044.1'	0.13	0.15	32.95	2.27	0.36	0.17	3060464	4.36	4.93	0.97
'CP019545.1'	0.37	0.17	15.75	1.02	0.29	0.12	2714535	8.12	7.95	0.99
'CP018604.1'	0.38	0.18	15.65	1.07	0.29	0.13	2743999	9.12	7.86	0.98
'CP010084.1'	0.26	0.14	30.32	1.94	0.27	0.12	3326187	6.83	4.76	0.98
'CP011475.1'	0.12	0.15	33.00	2.27	0.37	0.17	3060914	3.36	4.98	0.97
'CP010082.1'	0.16	0.14	32.32	2.16	0.34	0.15	3061930	6.48	4.82	0.97
'CP010076.1'	0.16	0.14	32.22	2.15	0.34	0.15	3060713	3.85	4.96	0.97
'CP010291.1'	0.14	0.15	32.93	2.26	0.36	0.16	3060833	6.34	4.83	0.97
'CP010078.1'	0.15	0.14	32.51	2.12	0.35	0.15	3060699	4.20	4.94	0.98
'CP011438.1'	0.14	0.15	32.79	2.24	0.35	0.16	3060807	3.29	4.98	0.97
'CP019634.1'	0.23	0.14	28.82	1.79	0.30	0.13	3515540	0.19	5.37	0.98
'CP011464.1'	0.20	0.14	30.80	2.07	0.31	0.14	3123307	8.43	4.79	0.98
'CP008702.1'	0.34	0.22	8.54	0.58	0.49	0.24	4411532	1.19	12.54	0.99
'CP009198.1'	0.34	0.22	8.53	0.58	0.49	0.24	4411532	0.41	12.66	0.99
'CP009199.1'	0.34	0.22	8.54	0.58	0.49	0.24	4411532	1.02	12.58	0.99
'CP009200.1'	0.34	0.22	8.53	0.58	0.49	0.24	4411532	0.41	12.67	0.99
'CP009201.1'	0.34	0.22	8.52	0.58	0.49	0.24	4411532	0.38	12.66	0.99
'CP009202.1'	0.34	0.23	8.49	0.60	0.49	0.25	4411532	0.04	12.73	0.99
'CP009203.1'	0.34	0.22	8.53	0.58	0.49	0.24	4411532	0.59	12.64	0.99
'CP009204.1'	0.34	0.22	8.53	0.58	0.49	0.24	4411532	0.71	12.62	0.99
'CP009205.1'	0.35	0.21	8.48	0.55	0.48	0.23	4411532	0.64	12.64	0.99
'CP009206.1'	0.33	0.21	8.58	0.57	0.50	0.24	4411532	0.54	12.65	0.99
'CP009207.1'	0.34	0.22	8.52	0.58	0.49	0.24	4411532	0.67	12.62	0.99
'CP009172.1'	0.34	0.22	8.53	0.58	0.49	0.24	4411532	0.37	12.67	0.99
'CP009173.1'	0.34	0.22	8.53	0.58	0.49	0.24	4411532	0.49	12.65	0.99
'CP009174.1'	0.34	0.22	8.51	0.58	0.49	0.24	4411532	0.49	12.65	0.99
'CP009175.1'	0.36	0.22	8.44	0.57	0.47	0.24	4411532	1.01	12.60	0.99
'CP009176.1'	0.34	0.22	8.53	0.58	0.49	0.24	4411532	1.04	12.56	0.99
'CP009177.1'	0.34	0.22	8.52	0.58	0.49	0.24	4411532	0.41	12.67	0.99
'CP009178.1'	0.33	0.21	8.56	0.56	0.50	0.23	4411532	0.81	12.60	0.99
'CP009179.1'	0.34	0.22	8.54	0.58	0.49	0.24	4411532	0.75	12.64	0.99
'CP009180.1'	0.34	0.22	8.53	0.58	0.49	0.24	4411532	0.12	12.72	0.99
'CP009181.1'	0.40	0.21	8.29	0.53	0.45	0.21	4411532	1.10	12.59	0.99
'CP009182.1'	0.35	0.21	8.48	0.55	0.48	0.23	4411532	0.78	12.60	0.99
'CP009184.1'	0.33	0.23	8.56	0.59	0.50	0.25	4411532	0.45	12.66	0.99
'CP009185.1'	0.34	0.22	8.53	0.58	0.49	0.24	4411532	0.08	12.72	0.99
'CP009186.1'	0.34	0.22	8.54	0.58	0.49	0.24	4411532	0.82	12.60	0.99
'CP009188.1'	0.34	0.22	8.52	0.58	0.49	0.24	4411532	0.68	12.61	0.99
'CP009189.1'	0.34	0.22	8.52	0.58	0.49	0.24	4411532	0.81	12.59	0.99
'CP009194.1'	0.33	0.23	8.55	0.59	0.50	0.25	4411532	0.35	12.69	0.99
'CP009196.1'	0.39	0.21	8.35	0.53	0.45	0.21	4411532	1.12	12.58	0.99
'CP009197.1'	0.34	0.22	8.53	0.58	0.49	0.24	4411532	0.99	12.57	0.99
'CP009190.1'	0.34	0.22	8.53	0.58	0.49	0.24	4411532	1.12	12.55	0.99
'CP009191.1'	0.34	0.22	8.53	0.58	0.49	0.24	4411532	0.33	12.69	0.99
'CP009193.1'	0.33	0.22	8.58	0.57	0.50	0.24	4411532	1.03	12.56	0.99
'CP011436.1'	0.13	0.15	33.08	2.31	0.36	0.17	3060756	6.38	4.83	0.97
'CP019666.1'	0.00	-1.00	10.13	0.46	0.46	0.11	6028390	0.00	16.55	0.97
'CP019678.1'	0.00	-1.00	9.28	0.36	0.62	0.12	3818057	0.00	16.42	0.98
'CP009192.1'	0.34	0.22	8.54	0.58	0.49	0.24	4411532	0.23	12.71	0.99
'CP009195.1'	0.34	0.22	8.53	0.58	0.49	0.24	4411532	1.02	12.56	0.99
'CP009187.1'	0.36	0.23	8.43	0.58	0.47	0.24	4411532	0.41	12.68	0.99
'CP009183.1'	0.34	0.22	8.53	0.58	0.49	0.24	4411532	1.19	12.53	0.99
'CP019726.1'	0.39	0.09	49.45	2.11	0.14	0.04	5209112	0.00	3.22	0.98
'CCYD0100061	0.54	0.11	25.11	1.16	0.14	0.05	3420761	12.06	4.63	0.99
'CCYT0100000	0.56	0.09	58.69	2.99	0.08	0.03	2399970	2.37	2.42	0.97
'CCYA0100026	0.48	0.18	16.05	1.10	0.23	0.11	1618306	3.95	7.25	0.98
'LK022890.1'	0.74	0.07	110.72	5.03	0.03	0.01	2616835	0.00	1.14	0.95
'LK023128.1'	0.75	0.07	117.50	5.68	0.02	0.01	2504534	0.48	1.06	0.95
'LM993667.1'	0.65	0.07	139.80	7.50	0.03	0.01	2795668	0.03	1.00	0.94
'LK934641.1'	0.65	0.07	140.91	7.52	0.03	0.01	2639166	1.32	0.99	0.94
'CVKT0100758	0.60	0.12	38.90	2.74	0.09	0.03	573988	0.00	3.50	0.97
'CVRI0100004	0.55	0.08	55.19	2.18	0.08	0.02	5381421	0.70	2.56	0.98
'HG983337.1'	0.22	0.14	28.41	1.96	0.29	0.12	1466191	0.79	5.96	0.98
'HG975513.1'	0.00	-1.00	176.46	2.53	0.32	0.02	90311507	4.63	1.50	0.97
'FAXB0100000	0.35	0.21	16.33	1.71	0.29	0.15	262854	0.00	8.56	0.98
'CZCC0100000	0.24	0.23	17.82	2.19	0.35	0.20	263113	0.00	8.55	0.97
'CZCL0100000	0.49	0.12	42.97	2.76	0.11	0.04	1156483	0.00	3.36	0.97
'CZCD0100000	0.25	0.23	17.63	2.06	0.35	0.19	263072	0.00	8.55	0.97
'CZCM0100000	0.30	0.17	17.53	1.55	0.31	0.12	341788	0.00	8.54	0.98
'CZCO0100000	0.00	0.23	25.31	3.00	0.41	0.26	970878	0.00	8.01	0.96
'CZCK0100000	0.27	0.22	17.20	1.99	0.33	0.18	262052	0.03	8.73	0.97
'CZCE0100000	0.35	0.21	16.35	1.69	0.29	0.15	262595	0.00	8.55	0.98
'CZCP0100000	0.33	0.26	16.86	2.25	0.30	0.18	215875	0.00	8.37	0.96
'CZCN0100000	0.38	0.22	15.79	1.66	0.28	0.15	262886	0.00	8.56	0.97
'LN907826.1'	0.17	0.18	9.34	0.53	0.63	0.25	9022917	0.15	13.05	0.99
'CZCJ0100000	0.37	0.20	16.03	1.58	0.28	0.14	263057	0.00	8.56	0.98
'CZCI0100000	0.17	0.25	18.42	2.48	0.39	0.23	247527	0.00	8.78	0.97

'CZCH0100000	0.26	0.22	17.43	1.98	0.34	0.18	263419	0.00	8.55	0.97
'CZCF0100000	0.10	0.28	18.07	2.53	0.47	0.32	413416	0.00	9.02	0.96
'CZCG0100000	0.30	0.17	17.52	1.58	0.31	0.13	341457	0.00	8.54	0.98
'CZCR0100000	0.16	0.23	18.58	2.37	0.40	0.23	247342	0.00	8.79	0.97
'FCQG0100000	0.24	0.14	33.66	2.26	0.26	0.11	2238694	0.00	4.93	0.97
'FCQH0100000	0.32	0.11	31.15	1.50	0.22	0.08	4752789	0.29	4.97	0.98
'LT160033.1'	0.75	0.07	117.19	5.74	0.02	0.01	2497646	0.10	1.06	0.94
'FIZV01054470	0.22	0.20	69.82	19.43	0.18	0.11	54847	0.00	3.62	0.88
'LT160614.1'	0.00	0.24	24.08	2.36	0.32	0.23	5159348	0.00	8.89	0.96
'FIZR01004686	0.63	0.14	45.63	4.36	0.07	0.03	322873	0.00	2.91	0.94
'FIZS01000981	0.52	0.13	38.84	3.21	0.11	0.05	393850	0.00	3.65	0.96
'FIZU01005539	0.38	0.16	51.36	7.02	0.14	0.07	182788	0.00	3.35	0.93
'FIZT01056129	0.00	-1.00	54.67	6.84	0.35	0.06	76575	0.01	5.40	0.91
'FJOF0100000	0.00	-1.00	40.61	1.35	0.43	0.06	6348331	0.26	4.92	0.96
'FAOM0157674	0.00	-1.00	93.74	5.44	0.23	0.03	823974	4.84	3.78	0.93
'CVIS01000001	0.53	0.10	>1000	>1000	0.03	0.01	102664	4.20	0.65	0.54
'LN813019.1'	0.00	-1.00	24.71	0.89	0.41	0.06	5031573	0.00	7.79	0.97
'FJOG0100000	0.00	-1.00	40.75	1.69	0.42	0.06	3933241	3.19	4.73	0.95
'FJUW0100000	0.19	0.14	39.46	2.69	0.28	0.13	3320302	0.53	4.37	0.97
'FJVC0100028	0.74	0.13	23.80	1.42	0.09	0.03	728490	5.40	4.53	0.98
'FJUX0100000	0.69	0.13	25.84	1.53	0.10	0.04	1165341	0.48	4.56	0.98
'FJVB0100000	0.20	0.07	94.89	4.62	0.18	0.05	4302264	8.79	2.19	0.97
'LT554468.1'	0.54	0.11	38.17	2.16	0.10	0.04	1761326	4.36	3.69	0.98
'LT555307.1'	0.12	0.05	166.72	5.37	0.21	0.05	32560536	13.48	1.32	0.97
'HF679023.1'	0.00	-1.00	368.27	62.98	0.02	0.00	6564208	0.05	4.75	0.68
'FKKZ0100000	0.38	0.14	28.98	2.21	0.19	0.08	688384	5.00	5.33	0.98
'LT558118.1'	0.00	-1.00	67.23	5.19	0.12	0.03	2105630	0.00	5.98	0.87
'FKLU0100000	0.48	0.06	121.90	4.36	0.06	0.02	9127245	0.00	1.41	0.97
'LT593974.1'	0.00	-1.00	25.73	1.41	0.28	0.06	4144542	0.00	8.42	0.94
'LT576315.1'	0.12	0.14	39.43	2.62	0.34	0.15	4019465	0.20	4.71	0.97
'FLRD0100191	0.61	0.09	160.48	16.19	0.03	0.01	569460	0.00	1.00	0.88
'FLRE0100016	0.19	0.12	87.60	9.35	0.19	0.08	657385	0.01	2.60	0.94
'FLQV0100012	0.14	0.15	181.84	67.73	0.17	0.08	94411	0.06	2.21	0.85
'FLQU0100019	0.70	0.09	185.95	20.43	0.02	0.01	490540	0.01	0.84	0.85
'FLQW0100129	0.56	0.16	261.74	141.85	0.04	0.02	55696	1.13	1.35	0.65
'LT594323.1'	0.39	0.29	6.39	0.50	0.56	0.34	6758600	0.00	15.30	0.99
'LT594324.1'	0.00	-1.00	7.80	0.34	0.91	0.23	6612057	0.00	15.12	0.98
'LT598496.1'	0.01	0.30	7.43	0.64	0.97	0.62	7074838	0.00	15.50	0.99
'FLSR0100488	0.06	0.05	152.96	4.02	0.24	0.05	37168685	1.62	1.96	0.98
'LT599501.1'	0.61	0.11	19.89	0.78	0.14	0.05	8560927	0.03	5.95	0.99
'FMJQ0100000	0.20	0.15	34.95	2.95	0.28	0.13	1245440	0.20	5.31	0.97
'LT615372.1'	0.00	-1.00	27.80	0.96	0.39	0.05	4638126	0.00	7.48	0.97
'LT615375.1'	0.00	-1.00	27.80	0.97	0.39	0.05	4638126	0.00	7.48	0.97
'LT615373.1'	0.00	-1.00	27.80	0.97	0.39	0.05	4638126	0.00	7.48	0.97
'LT615374.1'	0.00	-1.00	27.72	0.96	0.39	0.05	4638126	0.87	7.43	0.97
'LT615376.1'	0.00	-1.00	27.80	0.97	0.39	0.05	4638126	0.00	7.48	0.97
'LT615379.1'	0.00	-1.00	27.80	0.97	0.39	0.05	4638126	0.00	7.48	0.97
'LT615371.1'	0.00	-1.00	27.81	0.97	0.39	0.05	4638126	0.00	7.48	0.97
'LT629695.1'	0.00	-1.00	6.45	0.29	0.84	0.20	3400178	0.00	18.66	0.98
'LT629694.1'	0.00	0.31	7.72	0.78	0.93	0.60	4141280	0.01	15.47	0.99
'LT629693.1'	0.20	0.20	9.75	0.60	0.57	0.26	8339215	0.00	12.61	0.99
'LT629696.1'	0.00	0.31	7.72	0.78	0.93	0.60	4141447	0.00	15.47	0.99
'LT629697.1'	0.48	0.29	7.51	0.61	0.41	0.26	4228702	0.00	13.33	0.99
'LT629698.1'	0.01	0.31	7.72	0.78	0.93	0.60	4141652	0.02	15.46	0.99
'LT627734.1'	0.77	0.15	16.72	1.01	0.11	0.05	1663904	0.01	6.19	0.98
'LT629692.1'	0.31	0.26	6.61	0.48	0.64	0.34	3668670	0.00	15.33	0.99
'LT629699.1'	0.13	0.19	15.07	1.00	0.53	0.27	6263198	0.00	9.19	0.98
'LT629687.1'	0.03	0.19	14.25	1.01	0.64	0.32	6123913	0.00	10.38	0.99
'FMVU0100005	0.00	-1.00	55.86	4.51	0.25	0.04	480981	1.11	5.44	0.91
'LT627735.1'	0.58	0.10	39.21	1.74	0.09	0.03	3253959	0.00	3.39	0.98
'LT629688.1'	0.70	0.28	6.31	0.45	0.33	0.19	4461788	0.00	13.83	0.99
'LT627736.1'	0.27	0.36	6.21	0.63	0.71	0.51	2922123	0.00	16.18	0.99
'LT629689.1'	0.02	0.20	15.47	1.16	0.64	0.34	6674429	0.00	9.73	0.98
'LT629690.1'	0.56	0.22	11.84	0.80	0.26	0.14	4852066	0.00	8.89	0.98
'LT629691.1'	0.00	-1.00	14.93	0.46	0.68	0.10	6604078	0.00	9.83	0.98
'LT629712.1'	0.00	-1.00	14.76	0.67	0.59	0.12	2392976	0.00	10.58	0.97
'LT629713.1'	0.00	-1.00	16.04	0.49	0.60	0.09	6776659	0.00	9.64	0.98
'LT629702.1'	0.06	0.22	14.78	1.10	0.62	0.35	6727386	0.00	9.60	0.98
'LT629703.1'	0.21	0.19	9.41	0.54	0.58	0.25	7584236	0.00	12.84	0.99
'LT629700.1'	0.00	-1.00	11.25	0.49	0.69	0.13	2266470	0.00	13.14	0.98
'LT629701.1'	0.22	0.24	7.52	0.52	0.68	0.36	8565619	0.00	14.55	0.99
'LT629704.1'	0.07	0.20	15.35	1.09	0.59	0.32	6377174	0.00	9.42	0.98
'LT629705.1'	0.08	0.19	14.93	1.02	0.57	0.28	6502978	0.00	9.57	0.99
'LT629707.1'	0.00	0.31	7.72	0.78	0.93	0.60	4141489	0.01	15.47	0.99
'LT629706.1'	0.00	-1.00	17.04	0.59	0.55	0.09	5950238	0.00	9.67	0.98
'LT629709.1'	0.00	-1.00	16.13	0.56	0.60	0.10	6312685	0.00	9.65	0.98
'LT629708.1'	0.20	0.18	14.03	0.84	0.46	0.21	6373103	0.00	9.58	0.99
'LT629302.1'	0.61	0.10	39.68	1.89	0.09	0.03	3817662	0.00	3.25	0.98
'LT629711.1'	0.19	0.31	7.41	0.69	0.71	0.47	3942164	0.00	14.57	0.99
'LT629710.1'	0.39	0.33	7.22	0.67	0.51	0.36	5013970	0.00	13.77	0.98
'LT629755.1'	0.05	0.34	6.27	0.63	0.98	0.64	3692408	0.00	17.39	0.99
'LT629734.1'	0.00	-1.00	6.36	0.23	0.98	0.19	2773310	0.00	18.19	0.99
'LT629738.1'	0.00	-1.00	16.87	0.67	0.47	0.09	7047615	0.00	10.12	0.97
'LT629758.1'	0.08	0.26	8.38	0.64	0.79	0.46	10654181	0.00	13.95	0.99
'LT629746.1'	0.20	0.19	14.00	0.91	0.46	0.22	6529836	0.00	9.52	0.99
'LT629737.1'	0.75	0.09	61.08	2.56	0.04	0.01	3476061	0.00	1.96	0.97
'LT629733.1'	0.79	0.09	43.35	1.69	0.05	0.01	3098771	0.00	2.60	0.98
'LT629751.1'	0.00	-1.00	13.17	0.53	0.55	0.10	4642193	0.00	12.16	0.98
'LT629760.1'	0.00	-1.00	16.08	0.51	0.62	0.09	5634667	0.00	9.83	0.98
'LT629742.1'	0.12	0.25	8.35	0.69	0.71	0.38	3837821	0.00	14.56	0.99
'LT629753.1'	0.03	0.22	15.06	1.17	0.64	0.37	6539015	0.00	9.79	0.98
'LT629735.1'	0.00	-1.00	12.41	0.51	0.58	0.11	4150883	0.00	12.51	0.98
'LT629768.1'	0.00	-1.00	8.40	0.28	0.86	0.16	8620263	0.00	14.79	0.99
'LT629736.1'	0.69	0.16	9.23	0.44	0.24	0.08	3537092	0.00	10.43	0.99
'LT629757.1'	0.21	0.32	6.90	0.67	0.71	0.46	4058582	0.00	15.46	0.99
'LT629756.1'	0.02	0.19	14.08	0.92	0.66	0.32	6113481	0.00	10.46	0.99
'LT629747.1'	0.00	-1.00	14.98	0.55	0.63	0.11	7120591	0.00	10.17	0.98
'LT629761.1'	0.00	-1.00	16.64	0.60	0.50	0.09	7065202	0.00	10.02	0.98
'LT629748.1'	0.29	0.17	15.42	0.95	0.37	0.16	3987942	0.00	8.46	0.99
'LT629759.1'	0.00	-1.00	13.13	0.56	0.55	0.11	2353193	0.00	12.56	0.98
'LT629745.1'	0.74	0.09	46.45	2.00	0.05	0.02	3545181	0.00	2.56	0.98
'LT629739.1'	0.23	0.22	10.40	0.76	0.50	0.25	4441057	0.00	11.61	0.99
'LT629749.1'	0.29	0.35	6.83	0.68	0.64	0.45	4717614	0.00	15.09	0.98
'LT629744.1'	0.37	0.16	13.11	0.68	0.34	0.14	5868846	0.00	9.24	0.99
'LT629743.1'	0.00	-1.00	8.93	0.47	0.71	0.19	3191968	0.00	15.03	0.97
'LT629754.1'	0.66	0.08	48.58	1.88	0.06	0.02	4505800	0.00	2.63	0.98

'LT629750.1'	0.32	0.20	9.57	0.59	0.46	0.22	7842044	0.00	12.18	0.99
'LT629741.1'	0.83	0.10	43.24	2.02	0.04	0.01	3158583	0.00	2.57	0.97
'LT629752.1'	0.59	0.08	99.12	4.55	0.05	0.01	3951560	0.00	1.46	0.96
'LT629762.1'	0.00	-1.00	14.60	0.45	0.64	0.09	6092055	0.00	10.63	0.99
'LT629740.1'	0.43	0.10	36.31	1.68	0.15	0.05	6014056	0.00	4.00	0.98
'LT629771.1'	0.00	-1.00	7.54	0.33	0.73	0.19	7486057	0.00	16.77	0.98
'LT629764.1'	0.00	-1.00	29.89	1.01	0.38	0.05	5519819	0.00	7.12	0.97
'LT629763.1'	0.27	0.22	14.21	1.09	0.40	0.23	4030203	0.00	9.09	0.98
'LT629767.1'	0.17	0.17	14.18	0.86	0.49	0.22	6749275	0.00	9.54	0.99
'LT629769.1'	0.22	0.20	13.67	0.95	0.45	0.24	5874625	0.00	9.47	0.98
'LT629765.1'	0.00	-1.00	11.28	0.39	0.69	0.11	2633185	0.00	13.23	0.99
'LT629766.1'	0.07	0.28	10.22	0.97	0.68	0.44	4023083	0.00	12.50	0.98
'LT629770.1'	0.03	0.31	7.47	0.71	0.91	0.57	3552313	0.00	15.48	0.99
'LT629772.1'	0.54	0.21	7.04	0.39	0.39	0.18	6733014	0.00	13.57	0.99
'LT629775.1'	0.00	-1.00	8.28	0.34	0.81	0.19	9900053	0.00	15.08	0.98
'LT629773.1'	0.07	0.12	48.51	3.71	0.34	0.14	1684415	0.01	4.49	0.97
'LT629774.1'	0.73	0.08	48.07	1.81	0.05	0.01	3688445	0.00	2.49	0.98
'LT629776.1'	0.00	-1.00	7.54	0.34	0.91	0.21	2986943	0.00	16.06	0.98
'LT629777.1'	0.00	-1.00	15.83	0.52	0.62	0.10	6529636	0.00	9.68	0.98
'LT629778.1'	0.11	0.22	13.17	0.98	0.57	0.32	5943170	0.00	10.55	0.98
'LT629779.1'	0.57	0.22	9.60	0.64	0.28	0.14	4459178	0.00	10.91	0.99
'LT629801.1'	0.09	0.18	15.29	1.00	0.54	0.25	6417799	0.00	9.59	0.99
'LT629784.1'	0.00	-1.00	12.22	0.57	0.51	0.12	4626254	0.00	12.93	0.97
'LT630003.1'	0.43	0.11	39.97	1.95	0.14	0.05	5300235	0.00	3.68	0.98
'LT629787.1'	0.30	0.18	16.48	1.10	0.35	0.17	3796105	0.00	7.80	0.98
'LT629786.1'	0.00	-1.00	18.31	0.72	0.53	0.10	6842016	0.00	9.02	0.97
'LT629781.1'	0.00	-1.00	12.81	0.51	0.55	0.10	3763544	0.00	12.50	0.98
'LT629793.1'	0.16	0.19	14.46	0.96	0.50	0.25	7087525	0.00	9.38	0.99
'LT629799.1'	0.27	0.32	6.19	0.56	0.70	0.44	4405285	0.00	16.29	0.99
'LT629782.1'	0.15	0.18	14.63	0.95	0.50	0.24	6130232	0.00	9.64	0.99
'LT629788.1'	0.00	0.23	13.99	1.12	0.69	0.40	6092541	0.00	10.46	0.98
'LT629800.1'	0.26	0.19	14.43	0.97	0.40	0.21	6032706	0.00	8.99	0.98
'LT629803.1'	0.00	-1.00	15.58	0.57	0.61	0.11	6424905	0.00	9.73	0.98
'LT629783.1'	0.22	0.19	15.48	1.03	0.43	0.21	5072304	0.00	8.64	0.98
'LT629789.1'	0.00	-1.00	8.56	0.30	0.81	0.16	9472622	0.00	14.97	0.99
'LT629804.1'	0.30	0.19	20.05	1.55	0.30	0.16	1998342	0.01	7.07	0.98
'LT629780.1'	0.00	-1.00	12.43	0.61	0.56	0.13	3191154	0.00	12.61	0.97
'LT629791.1'	0.00	-1.00	7.58	0.28	0.84	0.18	7716600	0.00	16.32	0.99
'LT629785.1'	0.05	0.22	16.79	1.45	0.60	0.35	3769689	0.00	8.73	0.98
'LT629790.1'	0.14	0.20	13.86	0.92	0.53	0.27	6346235	0.00	9.78	0.99
'LT629797.1'	0.00	-1.00	15.88	0.65	0.52	0.10	5476367	0.00	10.19	0.97
'LT629802.1'	0.27	0.16	13.93	0.75	0.41	0.17	5853167	0.00	9.29	0.99
'LT629792.1'	0.33	0.21	12.59	0.93	0.38	0.20	2430293	0.00	9.67	0.98
'LT629796.1'	0.22	0.19	13.57	0.86	0.46	0.23	7041758	0.00	9.71	0.99
'LT629794.1'	0.70	0.08	76.46	3.36	0.04	0.01	3227923	0.00	1.67	0.97
'LT629798.1'	0.16	0.23	14.00	1.11	0.51	0.31	6150838	0.00	9.49	0.98
'LT629795.1'	0.33	0.22	15.10	1.14	0.34	0.20	5322478	0.00	8.24	0.98
'LT635756.1'	0.51	0.12	40.59	2.20	0.11	0.04	2919042	0.01	3.44	0.97
'LT635764.1'	0.63	0.10	37.62	1.60	0.08	0.03	3422859	0.12	3.42	0.98
'LT629867.1'	0.16	0.27	7.08	0.56	0.76	0.42	3937879	0.00	15.65	0.99
'LT629970.1'	0.27	0.23	11.95	0.91	0.46	0.26	4332289	0.00	10.07	0.98
'LT629971.1'	0.16	0.24	7.61	0.54	0.71	0.36	5987931	0.00	14.97	0.99
'LT629972.1'	0.00	-1.00	15.96	0.47	0.60	0.09	6592354	0.00	9.70	0.98
'FNXT0100122	0.59	0.11	28.77	1.54	0.11	0.04	1112289	0.12	4.64	0.98
'FNXX0100000	0.00	-1.00	140.86	3.64	0.22	0.02	15208799	2.99	2.49	0.96
'LT630002.1'	0.00	0.31	7.72	0.78	0.93	0.60	4141508	0.00	15.47	0.99
'LT630032.1'	0.01	0.31	7.72	0.78	0.93	0.60	4141231	0.00	15.47	0.99
'FPAK0100000	0.00	-1.00	114.17	2.20	0.30	0.02	22393718	1.66	2.53	0.97
'FQNF0100000	0.62	0.12	221.18	45.06	0.03	0.01	247000	0.00	0.92	0.75
'LT634571.1'	0.00	-1.00	411.56	4.46	0.20	0.01	129927919	0.70	1.10	0.98
'LT632320.1'	0.00	0.18	29.97	2.40	0.33	0.19	5174631	0.01	7.37	0.97
'LT649234.1'	0.44	0.14	71.14	11.42	0.10	0.04	95011	0.00	2.71	0.92
'LT635910.1'	0.46	0.17	78.51	16.72	0.09	0.04	92454	0.18	2.48	0.87
'LT658380.1'	0.03	0.22	90.70	38.48	0.30	0.18	91304	0.00	4.02	0.88
'LT642146.1'	0.00	-1.00	56.11	5.86	0.35	0.05	88335	0.12	5.07	0.93
'LT667505.1'	0.37	0.26	18.34	2.86	0.26	0.15	110906	0.09	7.99	0.96
'FQTN0100167	0.00	0.07	140.24	6.56	0.24	0.07	11737105	6.17	2.57	0.97
'LT670849.1'	0.12	0.22	10.39	0.71	0.65	0.34	7525217	0.00	12.34	0.99
'LT671675.1'	0.00	-1.00	12.27	0.55	0.61	0.12	2009306	0.11	12.51	0.98
'FTPI01000002	0.24	0.10	115.51	10.99	0.15	0.05	884900	11.30	1.79	0.94
'LT671813.1'	0.01	0.15	21.22	1.53	0.52	0.21	1507387	0.00	9.23	0.98
'LT671821.1'	0.00	0.14	21.32	1.41	0.52	0.20	1508930	0.00	9.23	0.99
'LT671789.1'	0.00	-1.00	21.62	0.65	0.51	0.06	1515123	0.00	9.24	0.99
'LT671798.1'	0.00	-1.00	21.09	0.69	0.48	0.06	1509484	0.00	9.63	0.98
'LT671805.1'	0.00	0.15	21.57	1.53	0.51	0.21	1509448	0.00	9.22	0.99
'LT707061.1'	0.35	0.19	12.93	0.84	0.36	0.17	5729155	0.00	9.34	0.99
'LT707063.1'	0.18	0.17	12.70	0.71	0.50	0.21	5897393	0.00	10.42	0.99
'LT707062.1'	0.27	0.19	13.45	0.84	0.42	0.21	6077628	0.00	9.43	0.99
'LT707064.1'	0.41	0.19	11.59	0.67	0.35	0.17	6090301	0.00	9.84	0.99
'LT707065.1'	0.00	0.21	10.81	0.74	0.78	0.39	6227325	0.00	12.99	0.99

**Sequence header**	**category**
AE014297.3 Drosophila melanogaster chromosome 3R	invertebrates'
CM000663.2 Homo sapiens chromosome 1, GRCh38 reference primary assembly	vertebrates_mammals'
CM000314.3 Pan troglodytes isolate Yerkes chimp pedigree #C0471 (Clint) chromosome 1, whole genome shotgun sequence	vertebrates_mammals'
CM000550.1 Pongo abelii chromosome 1	vertebrates_mammals'
CM000994.2 Mus musculus chromosome 1, GRC primary reference assembly	vertebrates_mammals'
CP002684.1 Arabidopsis thaliana chromosome 1 sequence	plants'
CM000070.3 Drosophila pseudoobscura pseudoobscura strain MV2-25 chromosome 2, whole genome shotgun sequence	invertebrates'
CM000072.5 Rattus norvegicus strain mixed chromosome 1, whole genome shotgun sequence	vertebrates_mammals'
GL010027.1 Loxodonta africana unplaced genomic scaffold scaffold_0, whole genome shotgun sequence	vertebrates_mammals'
DS995899.1 Penicillium marneffei ATCC 18224 scf_1105668340764 genomic scaffold, whole genome shotgun sequence	fungi'
CM002888.1 Danio rerio chromosome 4, GRCz10 reference primary assembly	vertebrates_non_mammals'
KB941397.1 Aplysia californica isolate F4 #8 unplaced genomic scaffold scaffold00001, whole genome shotgun sequence	invertebrates'
CH003449.1 Homo sapiens chromosome 2, whole genome shotgun sequence	vertebrates_mammals'
CM000463.1 Homo sapiens chromosome 2, whole genome shotgun sequence	vertebrates_mammals'
BL000001.2 TPA: Homo sapiens chromosome 7	vertebrates_mammals'
CM000209.2 Mus musculus chromosome 1, whole genome shotgun sequence	vertebrates_mammals'
BA000047.1 Pan troglodytes verus DNA, chromosome Y, partial sequence	vertebrates_mammals'
CM000054.5 Apis mellifera strain DH4 linkage group 1, whole genome shotgun sequence	invertebrates'
KN913731.1 Strongylocentrotus purpuratus isolate Spur 01 unplaced genomic scaffold Scaffold2, whole genome shotgun sequence	invertebrates'
CM000308.1 Macaca mulatta chromosome 1, whole genome shotgun sequence	vertebrates_mammals'
CM000231.2 Rattus norvegicus chromosome 1, whole genome shotgun sequence	vertebrates_mammals'
CM000411.1 Ornithorhynchus anatinus chromosome 3, whole genome shotgun sequence	vertebrates_mammals'
CM000039.3 Canis lupus familiaris chromosome X, whole genome shotgun sequence	vertebrates_mammals'
CM000368.1 Monodelphis domestica chromosome 1, whole genome shotgun sequence	vertebrates_mammals'
CM000377.2 Equus caballus chromosome 1, whole genome shotgun sequence	vertebrates_mammals'
CM000093.4 Gallus gallus isolate RJF #256 breed Red Jungle fowl, inbred line UCD001 chromosome 1, whole genome shotgun sequence	vertebrates_non_mammals'
CM000915.2 Nasonia vitripennis chromosome 1, whole genome shotgun sequence	invertebrates'
CM000278.3 Tribolium castaneum strain Georgia GA2 linkage group LG3, whole genome shotgun sequence	invertebrates'
CM000455.1 Plasmodium vivax chromosome 14, whole genome shotgun sequence	protozoa'
DS544890.1 Physcomitrella patens subsp. patens PHYPAscaffold_1 genomic scaffold, whole genome shotgun sequence	plants'
CH991767.1 Giardia lamblia ATCC 50803 SC_577 genomic scaffold, whole genome shotgun sequence	protozoa'
CH464491.1 Trypanosoma brucei brucei strain 927/4 GUTat10.1 chromosome 11 chr11_scaffold01 genomic scaffold, whole genome shotgun sequence	protozoa'
CM001232.1 Magnaporthe oryzae 70-15 chromosome 2, whole genome shotgun sequence	fungi'
CR382126.1 Kluyveromyces lactis strain NRRL Y-1140 chromosome F complete sequence	fungi'
CR382131.1 Yarrowia lipolytica CLIB122 chromosome E complete sequence	fungi'
CR380958.2 Candida glabrata strain CBS138 chromosome L complete sequence	fungi'
DS496108.1 Chlamydomonas reinhardtii strain CC-503 cw92 mt+ CHLREscaffold_1 genomic scaffold, whole genome shotgun sequence	plants'
CM000169.1 Aspergillus fumigatus Af293 chromosome 1, whole genome shotgun sequence	fungi'
DS027059.1 Aspergillus clavatus NRRL 1 1099423829805 genomic scaffold, whole genome shotgun sequence	fungi'
FR796432.1 Leishmania major strain Friedlin complete genome, chromosome 36	protozoa'
AE014187.2 Plasmodium falciparum 3D7 chromosome 14, complete sequence	protozoa'
CM000337.2 Populus trichocarpa linkage group LGI, whole genome shotgun sequence	plants'
DS113177.1 Trichomonas vaginalis G3 1047229024141 genomic scaffold, whole genome shotgun sequence	protozoa'
AM269894.1 Eimeria tenella chromosome 1, ordered contigs	protozoa'
FR799010.1 Leishmania braziliensis MHOM/BR/75/M2904 complete genome, chromosome 36	protozoa'
AM270981.1 Aspergillus niger supercontig An02	fungi'
CH991543.1 Monosiga brevicollis MX1 MONBRscaffold_2 genomic scaffold, whole genome shotgun sequence	invertebrates'
FR796468.1 Leishmania infantum JPCM5 genome chromosome 36	protozoa'
CU329670.1 Schizosaccharomyces pombe chromosome I, complete sequence	fungi'
BX284605.5 Caenorhabditis elegans chromosome V	invertebrates'
LN857024.1 Brugia malayi FR3 supercontig from assembly B_malayi-3.1 Bmal_v3_scaffold1, whole genome shotgun sequence	invertebrates'
CM000812.5 Sus scrofa isolate TJ Tabasco breed Duroc chromosome 1, whole genome shotgun sequence	vertebrates_mammals'
GK000001.2 TPA_asm: Bos taurus chromosome 1, whole genome shotgun sequence	vertebrates_mammals'
CAAJ01001934.1 Plasmodium chabaudi whole genome shotgun assembly, contig PC_RP2108, whole genome shotgun sequence	protozoa'
CM000695.1 Felis catus breed mixed chromosome A1, whole genome shotgun sequence	vertebrates_mammals'
EQ962652.1 Talaromyces stipitatus ATCC 10500 scf_1105507295523 genomic scaffold, whole genome shotgun sequence	fungi'
CM000761.2 Sorghum bicolor cultivar BTx623 chromosome 2, whole genome shotgun sequence	plants'
CM000177.6 Bos taurus breed Hereford chromosome 1, whole genome shotgun sequence	vertebrates_mammals'
CM000441.2 Clostridium difficile QCD-66c26 chromosome, whole genome shotgun sequence	bacteria'
CR940347.1 Theileria annulata strain Ankara isolate clone C9, *** SEQUENCING IN PROGRESS ***	protozoa'
CH476615.1 Uncinocarpus reesii 1704 scaffold_1 genomic scaffold, whole genome shotgun sequence	fungi'
EQ999973.1 Blastomyces dermatitidis ER-3 genomic scaffold supercont1.1, whole genome shotgun sequence	fungi'
GL014402.1 Saccoglossus kowalevskii unplaced genomic scaffold scaffold25907, whole genome shotgun sequence	invertebrates'
CM000790.1 Oryctolagus cuniculus chromosome 1, whole genome shotgun sequence	vertebrates_mammals'
CM000714.1 Bacillus cereus m1293 chromosome, whole genome shotgun sequence	bacteria'
FN597038.1 Vitis vinifera, whole genome shotgun sequence of line PN40024, unoriented chromosome 14, chr14	plants'
GG666612.1 Branchiostoma floridae genomic scaffold BRAFLscaffold_196, whole genome shotgun sequence	invertebrates'
CH408076.1 Clavispora lusitaniae ATCC 42720 scaffold_1 genomic scaffold, whole genome shotgun sequence	fungi'
GG657448.1 Blastomyces gilchristii SLH14081 genomic scaffold supercont1.1, whole genome shotgun sequence	fungi'
CM000742.1 Bacillus mycoides DSM 2048 chromosome, whole genome shotgun sequence	bacteria'
CM000741.1 Bacillus cereus AH1273 chromosome, whole genome shotgun sequence	bacteria'
CH477186.1 Aedes aegypti strain Liverpool supercont1.1 genomic scaffold, whole genome shotgun sequence	invertebrates'
GL048072.1 Macropus eugenii unplaced genomic scaffold Scaffold_4001, whole genome shotgun sequence	vertebrates_mammals'
CM002924.1 Cucumis sativus cultivar 9930 chromosome 3, whole genome shotgun sequence	plants'
GL020027.1 Hydra magnipapillata strain 105 unplaced genomic scaffold HYDRAscaffold_39356, whole genome shotgun sequence	invertebrates'
CM000831.1 Drosophila virilis strain 15010-1051.88 chromosome 6	invertebrates'
KE546988.1 Schizosaccharomyces cryophilus OY26 unplaced genomic scaffold supercont4.1, whole genome shotgun sequence	fungi'
CM004443.1 Xenopus tropicalis strain Nigerian chromosome 1, whole genome shotgun sequence	vertebrates_non_mammals'
GL348713.1 Arabidopsis lyrata subsp. lyrata unplaced genomic scaffold ARALYscaffold_1, whole genome shotgun sequence	plants'
GL192338.1 Ailuropoda melanoleuca unplaced genomic scaffold scaffold292, whole genome shotgun sequence	vertebrates_mammals'
CM000851.2 Glycine max cultivar Williams 82 chromosome 18, whole genome shotgun sequence	plants'
FR847123.2 Caenorhabditis briggsae complete genome, chrX, whole genome shotgun sequence	invertebrates'
CM000126.1 Oryza sativa (indica cultivar-group) chromosome 1, whole genome shotgun sequence	plants'
CM000856.1 Callithrix jacchus chromosome 1, whole genome shotgun sequence	vertebrates_mammals'
CM000151.3 Dictyostelium discoideum AX4 chromosome 2, whole genome shotgun sequence	protozoa'
GL277768.1 Nasonia giraulti strain RV2x(U) unplaced genomic scaffold SCAFFOLD1, whole genome shotgun sequence	invertebrates'
GL277950.1 Nasonia longicornis strain IV7(U) unplaced genomic scaffold SCAFFOLD1, whole genome shotgun sequence	invertebrates'
GL290984.1 Polysphondylium pallidum PN500 unplaced genomic scaffold PPL_scaffold2, whole genome shotgun sequence	protozoa'
KE141673.1 Homo sapiens unplaced genomic scaffold scaffold34, whole genome shotgun sequence	vertebrates_mammals'
GG738845.1 Naegleria gruberi genomic scaffold NAEGRscaffold_1, whole genome shotgun sequence	protozoa'
CM007647.1 Zea mays cultivar B73 chromosome 1, whole genome shotgun sequence	plants'
CH902617.1 Drosophila ananassae strain TSC#14024-0371.13 scaffold_13340 genomic scaffold, whole genome shotgun sequence	invertebrates'
CH954177.1 Drosophila erecta strain TSC#14021-0224.01 scaffold_4929 genomic scaffold, whole genome shotgun sequence	invertebrates'
CH916366.1 Drosophila grimshawi strain TSC#15287-2541.00 scaffold_15110 genomic scaffold, whole genome shotgun sequence	invertebrates'
CH933806.1 Drosophila mojavensis strain TSC#15081-1352.22 scaffold_6540 genomic scaffold, whole genome shotgun sequence	invertebrates'
CH479179.1 Drosophila persimilis strain MSH-3 scaffold_0 genomic scaffold, whole genome shotgun sequence	invertebrates'
CH480815.1 Drosophila sechellia strain Rob3c scaffold_0 genomic scaffold, whole genome shotgun sequence	invertebrates'
CH940647.1 Drosophila virilis strain TSC#15010-1051.87 scaffold_13049 genomic scaffold, whole genome shotgun sequence	invertebrates'
CAAI01000730.1 Plasmodium berghei whole genome shotgun assembly, contig PB_RP0829, whole genome shotgun sequence	protozoa'
AP008207.2 Oryza sativa Japonica Group DNA, chromosome 1, complete sequence, cultivar: Nipponbare	plants'
GJ065086.1 TPA_asm: Homo sapiens unplaced genomic scaffold scaffold1475, whole genome shotgun sequence	vertebrates_mammals'
CM000880.2 Brachypodium distachyon strain Bd21 chromosome 1, whole genome shotgun sequence	plants'
CM000885.1 Ovis aries chromosome 1, whole genome shotgun sequence	vertebrates_mammals'
CM000357.1 Anopheles gambiae str. PEST chromosome 2R, whole genome shotgun sequence	invertebrates'
CH964272.1 Drosophila willistoni strain TSC#14030-0811.24 scf2_1100000004943 genomic scaffold, whole genome shotgun sequence	invertebrates'
CM000160.2 Drosophila yakuba strain Tai18E2 chromosome 3R, whole genome shotgun sequence	invertebrates'
AAAC01000001.1 Bacillus anthracis str. A2012 chromosome, whole genome shotgun sequence	bacteria'
EQ966233.1 Postia placenta Mad-698-R POSPLscaffold_1 genomic scaffold, whole genome shotgun sequence	fungi'
EQ963472.1 Aspergillus flavus NRRL3357 scf_1106286418772 genomic scaffold, whole genome shotgun sequence	fungi'
DS235882.1 Pediculus humanus corporis 1103172108318 genomic scaffold, whole genome shotgun sequence	invertebrates'
GG692395.1 Candida tropicalis MYA-3404 genomic scaffold supercont3.1, whole genome shotgun sequence	fungi'
AM910996.2 Plasmodium knowlesi strain H chromosome 14, complete genome	protozoa'
GG680918.1 Perkinsus marinus ATCC 50983 genomic scaffold scf_1104296958712, whole genome shotgun sequence	protozoa'
AAEL02000352.1 Cryptosporidium hominis TU502 Ch_c3416, whole genome shotgun sequence	protozoa'
CR382138.2 Debaryomyces hansenii CBS767 chromosome F complete sequence	fungi'
DS989726.1 Cryptosporidium muris RN66 scf_1106632373453 genomic scaffold, whole genome shotgun sequence	protozoa'
CM002045.1 Toxoplasma gondii ME49 chromosome X, whole genome shotgun sequence	protozoa'
AE005174.2 Escherichia coli O157:H7 EDL933, complete genome	bacteria'
BN001308.1 TPA_asm: Aspergillus nidulans FGSC A4 chromosome VIII	fungi'
BX908798.1 Parachlamydia-related symbiont UWE25, complete genome	bacteria'
CP000048.1 Borrelia hermsii DAH, complete genome	bacteria'
CP000143.2 Rhodobacter sphaeroides 2.4.1 chromosome 1, complete sequence	bacteria'
CP000805.1 Treponema pallidum subsp. pallidum SS14, complete genome	bacteria'
CP000878.1 Prochlorococcus marinus str. MIT 9211 genome	bacteria'
CP001139.1 Vibrio fischeri MJ11 chromosome I, complete sequence	bacteria'
CP001277.1 Candidatus Hamiltonella defensa 5AT (Acyrthosiphon pisum) genome	bacteria'
CP001752.1 Treponema pallidum subsp. pallidum str. Chicago, complete genome	bacteria'
CU928162.2 Escherichia coli ED1a chromosome, complete genome	bacteria'
CU928163.2 Escherichia coli UMN026 chromosome, complete genome	bacteria'
CU928179.1 Zygosaccharomyces rouxii strain CBS732 chromosome G complete sequence	fungi'
FM864216.2 Mycoplasma conjunctivae HRC/581T complete genome	bacteria'
FM992688.1 Candida dubliniensis CD36 chromosome 1, complete sequence	fungi'
FN392235.1 Erwinia pyrifoliae DSM 12163 complete genome, culture collection DSM:12163	bacteria'
FN392319.1 Pichia pastoris GS115 chromosome 1, complete sequence	fungi'
FN545816.1 Clostridium difficile R20291 complete genome	bacteria'
CU468230.2 Acinetobacter baumannii SDF, complete genome	bacteria'
CU633749.1 Cupriavidus taiwanensis str. LMG19424 chromosome 1, complete genome	bacteria'
AM999887.1 Wolbachia endosymbiont of Culex quinquefasciatus Pel strain wPip complete genome	bacteria'
CM000937.1 Anolis carolinensis chromosome 1, whole genome shotgun sequence	vertebrates_non_mammals'
GL345134.1 Amphimedon queenslandica unplaced genomic scaffold AMPQUscaffold_13522, whole genome shotgun sequence	invertebrates'
BA000046.3 Pan troglodytes DNA, chromosome 22, complete sequence	vertebrates_mammals'
CP001574.1 Micromonas sp. RCC299 chromosome 1, complete sequence	plants'
AE016819.5 Ashbya gossypii ATCC 10895 chromosome VI, complete sequence	fungi'
AE017341.1 Cryptococcus neoformans var. neoformans JEC21 chromosome 1, complete sequence	fungi'

X59720.2 S.cerevisiae chromosome III complete DNA sequence	fungi'
AP006502.2 Cyanidioschyzon merolae strain 10D DNA, chromosome 20, complete genome	plants'
AL590450.1 chromosome XI of strain GB-M1 of Encephalitozoon cuniculi (Microspora)	fungi'
FN692037.1 Lactobacillus crispatus ST1 complete genome, strain ST1	bacteria'
CP000581.1 Ostreococcus lucimarinus CCE9901 chromosome 1, complete sequence	plants'
CU928171.1 Lachancea thermotolerans CBS 6340 chromosome G complete sequence	fungi'
GL349433.1 Thecamonas trahens ATCC 50062 unplaced genomic scaffold supercont1.1, whole genome shotgun sequence	protozoa'
DS028118.1 Phytophthora infestans T30-4 supercont1.1 genomic scaffold, whole genome shotgun sequence	protozoa'
GL349630.1 Acyrthosiphon pisum unplaced genomic scaffold Scaffold10, whole genome shotgun sequence	invertebrates'
GL376636.1 Pythium ultimum DAOM BR144 unplaced genomic scaffold scf_1117875582039, whole genome shotgun sequence	protozoa'
GL629765.1 Grosmannia clavigera kw1407 unplaced genomic scaffold GCSC_140, whole genome shotgun sequence	fungi'
GG742441.1 Grosmannia clavigera kw1407 genomic scaffold scaffold_2, whole genome shotgun sequence	fungi'
GL377302.1 Schizophyllum commune H4-8 unplaced genomic scaffold SCHCOscaffold_1, whole genome shotgun sequence	fungi'
CH408029.1 Chaetomium globosum CBS 148.51 scaffold_1 genomic scaffold, whole genome shotgun sequence	fungi'
GL377338.1 Atta cephalotes unplaced genomic scaffold scaffold00001, whole genome shotgun sequence	invertebrates'
GL377565.1 Selaginella moellendorffii unplaced genomic scaffold SELMOscaffold_0, whole genome shotgun sequence	plants'
GL378323.1 Volvox carteri f. nagariensis unplaced genomic scaffold VOLCAscaffold_1, whole genome shotgun sequence	plants'
JH798064.1 Botryotinia fuckeliana B05.10 unplaced genomic scaffold B0510_SuperContig_J3_V4, whole genome shotgun sequence	fungi'
DS547091.1 Laccaria bicolor S238N-H82 LACBIscaffold_1 genomic scaffold, whole genome shotgun sequence	fungi'
CM000961.1 Corynebacterium genitalium ATCC 33030 chromosome, whole genome shotgun sequence	bacteria'
GL379786.1 Caenorhabditis brenneri unplaced genomic scaffold Scfld02_0, whole genome shotgun sequence	invertebrates'
GL385395.1 Gaeumannomyces graminis var. tritici R3-111a-1 unplaced genomic scaffold supercont2.1, whole genome shotgun sequence	fungi'
BK006938.2 TPA_inf: Saccharomyces cerevisiae S288c chromosome IV, complete sequence	fungi'
CP001952.1 Encephalitozoon intestinalis ATCC 50506 chromosome XI, complete sequence	fungi'
CM000962.2 Meleagris gallopavo isolate NT-WF06-2002-E0010 breed Aviagen turkey brand Nicholas breeding stock chromosome 1, whole genome shotgun sequence	vertebrates_non_mammals'
CM000960.1 Escherichia coli str. K-12 substr. MG1655star chromosome, whole genome shotgun sequence	bacteria'
CM001648.1 Nomascus leucogenys chromosome 2, whole genome shotgun sequence	vertebrates_mammals'
CH445325.1 Parastagonospora nodorum SN15 scaffold_1, whole genome shotgun sequence	fungi'
CH476621.1 Sclerotinia sclerotiorum 1980 scaffold_1 genomic scaffold, whole genome shotgun sequence	fungi'
GL429767.1 Myotis lucifugus unplaced genomic scaffold scaffold_0, whole genome shotgun sequence	vertebrates_mammals'
GL475358.1 Caenorhabditis japonica strain DF5081 unplaced genomic scaffold Scaffold18210, whole genome shotgun sequence	invertebrates'
GL438234.1 Camponotus floridanus unplaced genomic scaffold scaffold107, whole genome shotgun sequence	invertebrates'
GL451853.1 Harpegnathos saltator unplaced genomic scaffold scaffold271, whole genome shotgun sequence	invertebrates'
JH119215.1 Wickerhamomyces anomalus NRRL Y-366 unplaced genomic scaffold scaffold00001, whole genome shotgun sequence	fungi'
GL455988.1 Oryza glaberrima chromosome 3 short arm genomic scaffold glaberrima_Chr3	plants'
GL433835.1 Chlorella variabilis unplaced genomic scaffold CHLNCscaffold_1, whole genome shotgun sequence	plants'
CM001015.1 Streptomyces clavuligerus ATCC 27064 chromosome, whole genome shotgun sequence	bacteria'
CM001020.1 Pseudomonas aeruginosa 39016 chromosome, whole genome shotgun sequence	bacteria'
CM001040.1 Malus x domestica chromosome 15, whole genome shotgun sequence	plants'
GL476399.1 Petromyzon marinus unplaced genomic scaffold scaffold_71, whole genome shotgun sequence	vertebrates_non_mammals'
GG697331.1 Glomerella graminicola M1.001 genomic scaffold supercont1.1, whole genome shotgun sequence	fungi'
GL501517.1 Mayetiola destructor strain Kansas Great Plain chromosome X1 unlocalized genomic scaffold X1.7, whole genome shotgun sequence	invertebrates'
CH236920.1 Aspergillus nidulans FGSC A4 chromosome VIII scaffold_1, whole genome shotgun sequence	fungi'
CM000694.1 Saccharomyces kluyveri NRRL Y-12651 chromosome H, whole genome shotgun sequence	fungi'
CP003820.1 Cryptococcus neoformans var. grubii H99 chromosome 1, complete sequence	fungi'
CH003412.1 Ciona savignyi psa_67 genomic scaffold, whole genome shotgun sequence	invertebrates'
CM000138.1 Oryza sativa Japonica Group chromosome 1, whole genome shotgun sequence	plants'
CH476732.1 Rhizopus oryzae RA 99-880 supercont3.1 genomic scaffold, whole genome shotgun sequence	fungi'
GG704911.1 Coccidioides immitis RS genomic scaffold supercont3.1, whole genome shotgun sequence	fungi'
CH408043.1 Saccharomyces cerevisiae RM11-1a scaffold_1 genomic scaffold, whole genome shotgun sequence	fungi'
CM000040.1 Cryptococcus neoformans var. neoformans B-3501A chromosome 1, whole genome shotgun sequence	fungi'
CM000638.1 Thalassiosira pseudonana CCMP1335 chromosome 1, whole genome shotgun sequence	protozoa'
CH408155.1 Pichia guilliermondii ATCC 6260 scaffold_1 genomic scaffold, whole genome shotgun sequence	fungi'
CH672346.1 Candida albicans WO-1 chromosome 1 supercont1.1 genomic scaffold, whole genome shotgun sequence	fungi'
KQ410557.1 Cryptococcus gattii R265 unplaced genomic scaffold supercont2.1, whole genome shotgun sequence	fungi'
CH396049.1 Drosophila pseudoobscura pseudoobscura strain MV2-25 scaff_89412 genomic scaffold, whole genome shotgun sequence	invertebrates'
DS268407.1 Caenorhabditis remanei Scfld02_0 genomic scaffold, whole genome shotgun sequence	invertebrates'
CM000578.1 Fusarium verticillioides 7600 chromosome 1, whole genome shotgun sequence	fungi'
CH476655.1 Ajellomyces capsulatus NAm1 scaffold_1 genomic scaffold, whole genome shotgun sequence	fungi'
CH476594.1 Aspergillus terreus NIH2624 scaffold_1 genomic scaffold, whole genome shotgun sequence	fungi'
DS027696.1 Neosartorya fischeri NRRL 181 1099437636264 genomic scaffold, whole genome shotgun sequence	fungi'
CH671918.1 Plasmodium falciparum HB3 supercont1.1 genomic scaffold, whole genome shotgun sequence	protozoa'
CH981524.1 Lodderomyces elongisporus NRRL YB-4239 supercont1.1 genomic scaffold, whole genome shotgun sequence	fungi'
KE387274.1 Toxoplasma gondii GT1 unplaced genomic scaffold scaffold00001, whole genome shotgun sequence	protozoa'
DS565998.1 Phytophthora ramorum strain Pr102 Scaffold1 genomic scaffold, whole genome shotgun sequence	protozoa'
JH159151.1 Phytophthora sojae unplaced genomic scaffold PHYSOscaffold_1, whole genome shotgun sequence	protozoa'
CH980625.1 Ascosphaera apis USDA-ARSEF 7405 SCAFFOLD1 genomic scaffold, whole genome shotgun sequence	fungi'
DS016058.1 Plasmodium falciparum Dd2 supercont1.1 genomic scaffold, whole genome shotgun sequence	protozoa'
DS016981.1 Coccidioides immitis H538.4 supercont1.1 genomic scaffold, whole genome shotgun sequence	fungi'
KE651166.1 Schizosaccharomyces japonicus yFS275 unplaced genomic scaffold supercont5.1, whole genome shotgun sequence	fungi'
DS022300.1 Batrachochytrium dendrobatidis JEL423 supercont1.1 genomic scaffold, whole genome shotgun sequence	fungi'
DS028093.1 Coccidioides immitis RMSCC 2394 supercont1.1 genomic scaffold, whole genome shotgun sequence	fungi'
DS178262.1 Puccinia graminis f. sp. tritici CRL 75-36-700-3 supercont2.1 genomic scaffold, whole genome shotgun sequence	fungi'
CM000589.1 Fusarium oxysporum f. sp. lycopersici 4287 chromosome 1, whole genome shotgun sequence	fungi'
DS231615.1 Pyrenophora tritici-repentis Pt-1C-BFP supercont1.1 genomic scaffold, whole genome shotgun sequence	fungi'
KI544492.1 Toxoplasma gondii VEG unplaced genomic scaffold scaffold00001, whole genome shotgun sequence	protozoa'
DS480378.1 Vanderwaltozyma polyspora DSM 70294 KpolScaffold1018 genomic scaffold, whole genome shotgun sequence	fungi'
DS268109.1 Coccidioides posadasii RMSCC 3488 supercont1.1 genomic scaffold, whole genome shotgun sequence	fungi'
DS268118.1 Coccidioides immitis RMSCC 3703 supercont1.1 genomic scaffold, whole genome shotgun sequence	fungi'
GG663363.1 Ajellomyces capsulatus G186AR genomic scaffold supercont2.1, whole genome shotgun sequence	fungi'
DS499594.1 Aspergillus fumigatus A1163 scf_000001 genomic scaffold, whole genome shotgun sequence	fungi'
DS544195.1 Coccidioides posadasii RMSCC 2133 supercont1.1 genomic scaffold, whole genome shotgun sequence	fungi'
DS544250.1 Coccidioides posadasii RMSCC 3700 supercont1.1 genomic scaffold, whole genome shotgun sequence	fungi'
DS544493.1 Coccidioides posadasii CPA 0001 supercont1.1 genomic scaffold, whole genome shotgun sequence	fungi'
DS985241.1 Trichoplax adhaerens TRIADscaffold_1 genomic scaffold, whole genome shotgun sequence	invertebrates'
DS499746.1 Plasmodium falciparum VS/1 supercont1.2 genomic scaffold, whole genome shotgun sequence	protozoa'
DS505602.1 Plasmodium falciparum Senegal_V34.04 supercont1.2 genomic scaffold, whole genome shotgun sequence	protozoa'
DS509930.1 Plasmodium falciparum RO-33 supercont1.1 genomic scaffold, whole genome shotgun sequence	protozoa'
DS514921.1 Plasmodium falciparum K1 supercont1.1 genomic scaffold, whole genome shotgun sequence	protozoa'
DS519693.1 Plasmodium falciparum FCC-2/Hainan supercont1.1 genomic scaffold, whole genome shotgun sequence	protozoa'
DS524649.1 Plasmodium falciparum D10 supercont1.1 genomic scaffold, whole genome shotgun sequence	protozoa'
DS529121.1 Plasmodium falciparum D6 supercont1.2 genomic scaffold, whole genome shotgun sequence	protozoa'
KE123623.1 Plasmodium falciparum 7G8 unplaced genomic scaffold supercont1.57, whole genome shotgun sequence	protozoa'
KE123511.1 Plasmodium falciparum Santa Lucia unplaced genomic scaffold supercont1.50, whole genome shotgun sequence	protozoa'
KN305531.1 Paracoccidioides brasiliensis Pb03 unplaced genomic scaffold supercont2.1, whole genome shotgun sequence	fungi'
KE503206.1 Schizosaccharomyces octosporus yFS286 unplaced genomic scaffold supercont6.1, whole genome shotgun sequence	fungi'
DS981520.1 Carica papaya supercontig_0 genomic scaffold, whole genome shotgun sequence	plants'
DS560032.1 Coccidioides posadasii RMSCC 1037 supercont1.1 genomic scaffold, whole genome shotgun sequence	fungi'
DS560667.1 Coccidioides posadasii RMSCC 1038 supercont1.1 genomic scaffold, whole genome shotgun sequence	fungi'
DS561724.1 Coccidioides posadasii CPA 0020 supercont1.1 genomic scaffold, whole genome shotgun sequence	fungi'
DS562368.1 Coccidioides posadasii CPA 0066 supercont1.1 genomic scaffold, whole genome shotgun sequence	fungi'
DS572695.1 Verticillium dahliae VdLs.17 supercont1.1 genomic scaffold, whole genome shotgun sequence	fungi'
KN293992.1 Paracoccidioides sp. 'lutzii' Pb01 unplaced genomic scaffold supercont2.1, whole genome shotgun sequence	fungi'
KN275957.1 Paracoccidioides brasiliensis Pb18 unplaced genomic scaffold supercont2.1, whole genome shotgun sequence	fungi'
EQ090207.1 Anopheles gambiae M scf_1925491379 genomic scaffold, whole genome shotgun sequence	invertebrates'
EQ099730.1 Anopheles gambiae S scf_1106392397136 genomic scaffold, whole genome shotgun sequence	invertebrates'
DS572940.1 Heterodera glycines HG2_15 genomic scaffold, whole genome shotgun sequence	invertebrates'
DS985214.1 Verticillium albo-atrum VaMs.102 supercont1.1 genomic scaffold, whole genome shotgun sequence	fungi'
EQ151724.1 Mchenga conophoros CCONA1000001 genomic scaffold, whole genome shotgun sequence	vertebrates_non_mammals'
EQ169210.1 Labeotropheus fuelleborni LFUEA1000001 genomic scaffold, whole genome shotgun sequence	vertebrates_non_mammals'
EQ185432.1 Melanochromis auratus MAURA1000001 genomic scaffold, whole genome shotgun sequence	vertebrates_non_mammals'
EQ202275.1 Maylandia zebra MZEBA1000001 genomic scaffold, whole genome shotgun sequence	vertebrates_non_mammals'
EQ220184.1 Rhamphochromis esox RESOA1000001 genomic scaffold, whole genome shotgun sequence	vertebrates_non_mammals'
CM000605.1 Phaeodactylum tricornutum CCAP 1055/1 chromosome 1, whole genome shotgun sequence	protozoa'
DS989822.1 Arthroderma gypseum CBS 118893 supercont1.1 genomic scaffold, whole genome shotgun sequence	fungi'
DS990636.1 Ajellomyces capsulatus H88 supercont1.1 genomic scaffold, whole genome shotgun sequence	fungi'
GG692419.1 Ajellomyces capsulatus H143 genomic scaffold supercont2.1, whole genome shotgun sequence	fungi'
HF943549.1 Blumeria graminis f. sp. hordei DH14 genomic scaffold, sca005486, whole genome shotgun sequence	fungi'
DS995701.1 Microsporum canis CBS 113480 supercont1.1 genomic scaffold, whole genome shotgun sequence	fungi'
DS995718.1 Trichophyton equinum CBS 127.97 supercont1.1 genomic scaffold, whole genome shotgun sequence	fungi'
GG663735.1 Micromonas pusilla CCMP1545 genomic scaffold MICPUCscaffold_1, whole genome shotgun sequence	plants'
GG745328.1 Allomyces macrogynus ATCC 38327 genomic scaffold supercont3.1, whole genome shotgun sequence	fungi'
KE346360.1 Capsaspora owczarzaki ATCC 30864 unplaced genomic scaffold supercont2.1, whole genome shotgun sequence	invertebrates'
GG698896.1 Nectria haematococca mpVI 77-13-4 chromosome 1 genomic scaffold NECHAsca_1_chr1_3_0, whole genome shotgun sequence	fungi'
GG700648.1 Trichophyton rubrum CBS 118892 genomic scaffold supercont2.1, whole genome shotgun sequence	fungi'
GG698477.1 Trichophyton tonsurans CBS 112818 genomic scaffold supercont1.1, whole genome shotgun sequence	fungi'
CM000924.1 Saccharomyces cerevisiae Sigma1278b chromosome 4, whole genome shotgun sequence	fungi'
ACYE01000120.1 Trichophyton verrucosum HKI 0517 contig00205_1.f1.exp, whole genome shotgun sequence	fungi'
ADAS02000001.1 Puccinia triticina 1-1 BBBD Race 1 supercont2.1, whole genome shotgun sequence	fungi'
KK583189.1 Saprolegnia parasitica CBS 223.65 unplaced genomic scaffold supercont2.1, whole genome shotgun sequence	protozoa'
JH379045.1 Epichloe festucae E2368 unplaced genomic scaffold scaffold00001, whole genome shotgun sequence	fungi'
GG749407.1 Blastomyces dermatitidis ATCC 18188 supercont1.1, whole genome shotgun sequence	fungi'
DF090316.1 Bombyx mori DNA, scaffold: Bm_scaf1, strain: p50T/Dazao	invertebrates'
FN429986.1 Tuber melanosporum whole genome shotgun sequence assembly, scaffold_1, strain Mel28	fungi'
FN668638.1 Blastocystis hominis, Singapore isolate B (sub-type 7) whole genome shotgun sequence assembly, scaffold_0	protozoa'
EQ973772.1 Ricinus communis genomic scaffold scf_1106159304512, whole genome shotgun sequence	plants'
CH379587.1 Bombyx mori strain Dazao Scaffold000001 genomic scaffold, whole genome shotgun sequence	invertebrates'
DS562855.1 Cavia porcellus supercont2_0 genomic scaffold, whole genome shotgun sequence	vertebrates_mammals'
FN330975.1 Schistosoma japonicum isolate Anhui clone SJC_S000000, complete sequence, whole genome shotgun sequence	invertebrates'
CM000518.1 Taeniopygia guttata chromosome 2, whole genome shotgun sequence	vertebrates_non_mammals'
DF000001.1 Oryzias latipes DNA, scaffold1, strain: HNI	vertebrates_non_mammals'
KN525718.1 Pteropus vampyrus isolate Shadow unplaced genomic scaffold Scaffold1, whole genome shotgun sequence	vertebrates_mammals'
JH472452.1 Tursiops truncatus unplaced genomic scaffold Scaffold6, whole genome shotgun sequence	vertebrates_mammals'
KN672347.1 Dipodomys ordii isolate 6190 unplaced genomic scaffold Scaffold1, whole genome shotgun sequence	vertebrates_mammals'
FR853080.2 Gorilla gorilla gorilla genomic chromosome, chr1, whole genome shotgun sequence	vertebrates_mammals'

KN676130.1 Procavia capensis isolate Dallas Zoo Isis #01D731 unplaced genomic scaffold Scaffold1, whole genome shotgun sequence	vertebrates_mammals'
CM000438.1 Burkholderia thailandensis ATCC 700388 chromosome 1, whole genome shotgun sequence	bacteria'
CM000287.4 Clostridium difficile QCD-32g58 chromosome, whole genome shotgun sequence	bacteria'
CM002299.1 Congregibacter litoralis KT71 chromosome, whole genome shotgun sequence	bacteria'
CM000440.1 Fusobacterium nucleatum subsp. polymorphum ATCC 10953 chromosome, whole genome shotgun sequence	bacteria'
CM000637.1 Clostridium difficile QCD-63q42 chromosome, whole genome shotgun sequence	bacteria'
CM000661.1 Clostridium difficile QCD-76w55 chromosome, whole genome shotgun sequence	bacteria'
CM000657.1 Clostridium difficile QCD-97b34 chromosome, whole genome shotgun sequence	bacteria'
CM000658.1 Clostridium difficile QCD-37x79 chromosome, whole genome shotgun sequence	bacteria'
CM002917.1 Hoeflea phototrophica DFL-43 chromosome, whole genome shotgun sequence	bacteria'
CM000950.1 Streptomyces pristinaespiralis ATCC 25486 chromosome, whole genome shotgun sequence	bacteria'
CM000951.1 Streptomyces sviceus ATCC 29083 chromosome, whole genome shotgun sequence	bacteria'
CM000604.1 Clostridium difficile ATCC 43255 chromosome, whole genome shotgun sequence	bacteria'
CM000659.1 Clostridium difficile CIP 107932 chromosome, whole genome shotgun sequence	bacteria'
CM000660.1 Clostridium difficile QCD-23m63 chromosome, whole genome shotgun sequence	bacteria'
CM000662.1 Escherichia coli O157:H7 str. TW14588 chromosome, whole genome shotgun sequence	bacteria'
CM000487.1 Bacillus subtilis subsp. subtilis str. 168 chromosome, whole genome shotgun sequence	bacteria'
CM000489.1 Bacillus subtilis subsp. subtilis str. JH642 chromosome, whole genome shotgun sequence	bacteria'
CM000490.1 Bacillus subtilis subsp. subtilis str. SMY chromosome, whole genome shotgun sequence	bacteria'
CM000953.1 Lactobacillus jensenii JV-V16 chromosome, whole genome shotgun sequence	bacteria'
CM000955.1 Finegoldia magna ATCC 53516 chromosome, whole genome shotgun sequence	bacteria'
CM000952.1 Staphylococcus aureus subsp. aureus MN8 chromosome, whole genome shotgun sequence	bacteria'
CM000770.1 Rickettsia endosymbiont of Ixodes scapularis chromosome, whole genome shotgun sequence	bacteria'
CM001024.1 Aeromicrobium marinum DSM 15272 chromosome, whole genome shotgun sequence	bacteria'
CM000715.1 Bacillus cereus ATCC 10876 chromosome, whole genome shotgun sequence	bacteria'
CM000716.1 Bacillus cereus BGSC 6E1 chromosome, whole genome shotgun sequence	bacteria'
CM000717.1 Bacillus cereus 172560W chromosome, whole genome shotgun sequence	bacteria'
CM000718.1 Bacillus cereus MM3 chromosome, whole genome shotgun sequence	bacteria'
CM000719.1 Bacillus cereus AH621 chromosome, whole genome shotgun sequence	bacteria'
CM000720.1 Bacillus cereus R309803 chromosome, whole genome shotgun sequence	bacteria'
CM000721.1 Bacillus cereus ATCC 4342 chromosome, whole genome shotgun sequence	bacteria'
CM000722.1 Bacillus cereus m1550 chromosome, whole genome shotgun sequence	bacteria'
CM000723.1 Bacillus cereus BDRD-ST24 chromosome, whole genome shotgun sequence	bacteria'
CM000724.1 Bacillus cereus BDRD-ST26 chromosome, whole genome shotgun sequence	bacteria'
CM000725.1 Bacillus cereus BDRD-ST196 chromosome, whole genome shotgun sequence	bacteria'
CM000726.1 Bacillus cereus BDRD-Cer4 chromosome, whole genome shotgun sequence	bacteria'
CM000727.1 Bacillus cereus 95/8201 chromosome, whole genome shotgun sequence	bacteria'
CM000728.1 Bacillus cereus Rock1-3 chromosome, whole genome shotgun sequence	bacteria'
CM000729.1 Bacillus cereus Rock1-15 chromosome, whole genome shotgun sequence	bacteria'
CM000730.1 Bacillus cereus Rock3-28 chromosome, whole genome shotgun sequence	bacteria'
CM000731.1 Bacillus cereus Rock3-29 chromosome, whole genome shotgun sequence	bacteria'
CM000732.1 Bacillus cereus Rock3-42 chromosome, whole genome shotgun sequence	bacteria'
CM000733.1 Bacillus cereus Rock3-44 chromosome, whole genome shotgun sequence	bacteria'
CM000734.1 Bacillus cereus Rock4-2 chromosome, whole genome shotgun sequence	bacteria'
CM000735.1 Bacillus cereus Rock4-18 chromosome, whole genome shotgun sequence	bacteria'
CM000736.1 Bacillus cereus F65185 chromosome, whole genome shotgun sequence	bacteria'
CM000737.1 Bacillus cereus AH603 chromosome, whole genome shotgun sequence	bacteria'
CM000738.1 Bacillus cereus AH676 chromosome, whole genome shotgun sequence	bacteria'
CM000739.1 Bacillus cereus AH1271 chromosome, whole genome shotgun sequence	bacteria'
CM000740.1 Bacillus cereus AH1272 chromosome, whole genome shotgun sequence	bacteria'
CM000743.1 Bacillus mycoides Rock1-4 chromosome, whole genome shotgun sequence	bacteria'
CM000744.1 Bacillus mycoides Rock3-17 chromosome, whole genome shotgun sequence	bacteria'
CM000745.1 Bacillus pseudomycoides DSM 12442 chromosome, whole genome shotgun sequence	bacteria'
CM000746.1 Bacillus thuringiensis serovar tochigiensis BGSC 4Y1 chromosome, whole genome shotgun sequence	bacteria'
CM000747.1 Bacillus thuringiensis Bt407 chromosome, whole genome shotgun sequence	bacteria'
CM000748.1 Bacillus thuringiensis serovar thuringiensis str. T01001 chromosome, whole genome shotgun sequence	bacteria'
CM000749.1 Bacillus thuringiensis serovar sotto str. T04001 chromosome, whole genome shotgun sequence	bacteria'
CM000750.1 Bacillus thuringiensis serovar pakistani str. T13001 chromosome, whole genome shotgun sequence	bacteria'
CM000751.1 Bacillus thuringiensis serovar kurstaki str. T03a001 chromosome, whole genome shotgun sequence	bacteria'
CM000752.1 Bacillus thuringiensis serovar monterrey BGSC 4AJ1 chromosome, whole genome shotgun sequence	bacteria'
CM000753.1 Bacillus thuringiensis serovar berliner ATCC 10792 chromosome, whole genome shotgun sequence	bacteria'
CM000754.1 Bacillus thuringiensis serovar andalousiensis BGSC 4AW1 chromosome, whole genome shotgun sequence	bacteria'
CM000755.1 Bacillus thuringiensis serovar pondicheriensis BGSC 4BA1 chromosome, whole genome shotgun sequence	bacteria'
CM000756.1 Bacillus thuringiensis serovar huazhongensis BGSC 4BD1 chromosome, whole genome shotgun sequence	bacteria'
CM000757.1 Bacillus thuringiensis serovar pulsiensis BGSC 4CC1 chromosome, whole genome shotgun sequence	bacteria'
CM000758.1 Bacillus thuringiensis IBL 200 chromosome, whole genome shotgun sequence	bacteria'
CM000759.1 Bacillus thuringiensis IBL 4222 chromosome, whole genome shotgun sequence	bacteria'
CM000776.2 Helicobacter canadensis MIT 98-5491 chromosome, whole genome shotgun sequence	bacteria'
CM000787.2 Mycobacterium tuberculosis KZN 4207 chromosome, whole genome shotgun sequence	bacteria'
CM000788.2 Mycobacterium tuberculosis KZN V2475 chromosome, whole genome shotgun sequence	bacteria'
CM000789.2 Mycobacterium tuberculosis KZN R506 chromosome, whole genome shotgun sequence	bacteria'
CM000913.1 Streptomyces clavuligerus ATCC 27064 chromosome, whole genome shotgun sequence	bacteria'
CM000854.1 Campylobacter jejuni subsp. jejuni 1336 chromosome, whole genome shotgun sequence	bacteria'
CM000855.1 Campylobacter jejuni subsp. jejuni 414 chromosome, whole genome shotgun sequence	bacteria'
CM001834.1 Pseudomonas savastanoi pv. savastanoi NCPPB 3335 chromosome, whole genome shotgun sequence	bacteria'
CM001149.1 Rhodococcus equi ATCC 33707 chromosome, whole genome shotgun sequence	bacteria'
CM000920.1 Gluconacetobacter hansenii ATCC 23769 chromosome, whole genome shotgun sequence	bacteria'
KN196458.1 Choloepus hoffmanni isolate OR478-KB4484 unplaced genomic scaffold Scaffold264165, whole genome shotgun sequence	vertebrates_mammals'
KE950501.1 Tarsius syrichta isolate Samal-C Ts95f unplaced genomic scaffold Scaffold316281, whole genome shotgun sequence	vertebrates_mammals'
DS362266.1 Ochotona princeps scaffold_0 genomic scaffold, whole genome shotgun sequence	vertebrates_mammals'
KB632434.1 Vicugna pacos unplaced genomic scaffold Scaffold0, whole genome shotgun sequence	vertebrates_mammals'
DG000025.1 Oryza sativa Japonica Group DNA, chromosome 1, cultivar: Koshihikari, whole genome shotgun sequence	plants'
GL530680.1 Caenorhabditis sp. PS1010 unplaced genomic scaffold ps1010rel4_scafRNAPATHr22055, whole genome shotgun sequence	invertebrates'
KI635855.1 Callorhinchus milii isolate IMCB2004 unplaced genomic scaffold scaffold_1, whole genome shotgun sequence	vertebrates_non_mammals'
CM000436.1 Cryptosporidium parvum Iowa II chromosome 8, whole genome shotgun sequence	protozoa'
AAGK01000001.1 Theileria parva strain Muguga chromosome 1, whole genome shotgun sequence	protozoa'
AAXT01000001.1 Babesia bovis strain T2Bo chromosome 3, whole genome shotgun sequence	protozoa'
CT867985.1 Paramecium tetraurelia chromosome undetermined scaffold_1, whole genome shotgun sequence	protozoa'
CM007668.1 Microcebus murinus isolate mixed chromosome 8, whole genome shotgun sequence	vertebrates_mammals'
CP000395.1 Borrelia afzelii PKo, complete genome	bacteria'
CM001022.1 Aminomonas paucivorans DSM 12260 chromosome, whole genome shotgun sequence	bacteria'
GL537212.1 Pyrenophora teres f. teres 0-1 unplaced genomic scaffold scaffold_193518, whole genome shotgun sequence	fungi'
GL538361.1 Ajellomyces dermatitidis ATCC 26199 unplaced genomic scaffold supercont1.1, whole genome shotgun sequence	fungi'
GL541643.1 Microbotryum lychnidis-dioicae p1A1 Lamole unplaced genomic scaffold supercont1.1, whole genome shotgun sequence	fungi'
CM001403.1 Mucilaginibacter paludis DSM 18603 chromosome, whole genome shotgun sequence	bacteria'
CM001023.1 Algoriphagus sp. PR1 chromosome, whole genome shotgun sequence	bacteria'
CM001025.1 Pseudomonas fluorescens WH6 chromosome, whole genome shotgun sequence	bacteria'
AABW01000001.1 Rickettsia sibirica 246 chromosome, complete sequence, whole genome shotgun sequence	bacteria'
AACF01000001.1 Naumovozyma castellii NRRL Y-12630 YM476-Contig721, whole genome shotgun sequence	fungi'
AACG02000001.1 Saccharomyces bayanus 623-6C YM4911-Contig02.678, whole genome shotgun sequence	fungi'
AACH01000001.1 Saccharomyces mikatae IFO 1815 YM4906-Contig2900, whole genome shotgun sequence	fungi'
JH797040.1 Saccharomyces kudriavzevii IFO 1802 unplaced genomic scaffold c2070, whole genome shotgun sequence	fungi'
GL985056.1 Trichoderma reesei QM6a unplaced genomic scaffold TRIREscaffold_1, whole genome shotgun sequence	fungi'
AAQW01000001.1 Pseudomonas aeruginosa PACS2 chromosome, whole genome shotgun sequence	bacteria'
AASL01000001.1 Campylobacter jejuni subsp. jejuni 81-176 chromosome, whole genome shotgun sequence	bacteria'
AAXK01002635.1 Malassezia restricta CBS 7877 MRE_CONTIG_2694, whole genome shotgun sequence	fungi'
GL636486.1 Coccidioides posadasii str. Silveira unplaced genomic scaffold supercont2.1, whole genome shotgun sequence	fungi'
ABCY02000001.1 Borreliella valaisiana VS116 chromosome, whole genome shotgun sequence	bacteria'
ABDF02000006.1 Trichoderma virens Gv29-8 TRIVIscaffold_6, whole genome shotgun sequence	fungi'
ABIZ01000001.1 Verrucomicrobium spinosum DSM 4136 chromosome, whole genome shotgun sequence	bacteria'
JH597776.1 Hyaloperonospora arabidopsidis Emoy2 unplaced genomic scaffold SuperContig0, whole genome shotgun sequence	protozoa'
ABYD01000001.1 Chlamydia trachomatis 6276 chromosome, whole genome shotgun sequence	bacteria'
ABYE01000001.1 Chlamydia trachomatis 6276s chromosome, whole genome shotgun sequence	bacteria'
ABYF01000001.1 Chlamydia trachomatis 70 chromosome, whole genome shotgun sequence	bacteria'
ABYG01000001.1 Chlamydia trachomatis 70s chromosome, whole genome shotgun sequence	bacteria'
ACDA01005048.1 Nothobranchius kuhntae clone Nku-G-d-27c09 Nku-G-d-27c09.f1, whole genome shotgun sequence	vertebrates_non_mammals'
ACFJ01000001.1 Chlamydia trachomatis D(s)2923 chromosome, whole genome shotgun sequence	bacteria'
ACFK01000001.1 Buchnera aphidicola str. LSR1 (Acyrthosiphon pisum) chromosome, whole genome shotgun sequence	bacteria'
ACUI01000001.1 Chlamydia trachomatis L2tet1 chromosome, whole genome shotgun sequence	bacteria'
ACUJ01000001.1 Chlamydia muridarum MopnTet14 chromosome, whole genome shotgun sequence	bacteria'
CM001368.1 Desulfovibrio sp. FW1012B chromosome, whole genome shotgun sequence	bacteria'
CM001435.1 Shewanella baltica OS183 chromosome, whole genome shotgun sequence	bacteria'
HE602535.1 Takifugu rubripes chromosome, Chr_1	vertebrates_non_mammals'
ADBW01000001.1 Onchocerca volvulus cont1.1, whole genome shotgun sequence	invertebrates'
AAXM01000001.1 Rattus norvegicus strain SHRSP/Bbb clone SHRSPa45h11 SHRSPa45h11.s1, whole genome shotgun sequence	vertebrates_mammals'
CAAE01014581.1 Tetraodon nigroviridis chromosome 5 SCAF14581, whole genome shotgun sequence	vertebrates_non_mammals'
ADBV01000001.1 Wuchereria bancrofti cont1.1, whole genome shotgun sequence	invertebrates'
AAXL01000001.1 Rattus norvegicus strain WKY/Bbb clone WKY-G-j-48b04 WKY-G-j-48b04.r1, whole genome shotgun sequence	vertebrates_mammals'
AASU01007866.1 Drosophila simulans strain New Caledonia 48S Cont6.2, whole genome shotgun sequence	invertebrates'
AASW01016250.1 Drosophila simulans strain SIM6 Cont6.3, whole genome shotgun sequence	invertebrates'
AAXN01000001.1 Rattus norvegicus strain SS/JrNhsd clone DS-g-a-89h10 DS-g-a-89h10.f1, whole genome shotgun sequence	vertebrates_mammals'
KQ034056.1 Rhodnius prolixus unplaced genomic scaffold Scaffold0, whole genome shotgun sequence	invertebrates'
BABP01002216.1 Solanum lycopersicum DNA, contig: SlSBM_S00131_04, strain: Heinz 1706, whole genome shotgun sequence	plants'
GL739410.1 Phoenix dactylifera unplaced genomic scaffold PDK_30s6550926, whole genome shotgun sequence	plants'
ADMZ02082066.1 Rhipicephalus microplus strain Deutsch Ctg17386, whole genome shotgun sequence	invertebrates'
JH798146.1 Sorex araneus unplaced genomic scaffold scaffold00001, whole genome shotgun sequence	vertebrates_mammals'
GL873520.1 Otolemur garnettii unplaced genomic scaffold scaffold00001, whole genome shotgun sequence	vertebrates_mammals'
CM001378.2 Felis catus isolate Cinnamon breed Abyssinian chromosome A1, whole genome shotgun sequence	vertebrates_mammals'
AAFW02000145.1 Saccharomyces cerevisiae YJM789 chromosome 4 chrIV.Contig338, whole genome shotgun sequence	fungi'
GL622787.1 Trichinella spiralis unplaced genomic scaffold Scfld4, whole genome shotgun sequence	invertebrates'
ABLO02019976.1 Nothobranchius furzeri strain GRZ clone GRZ-G-b-1202b10 GRZ-G-b-1202b10.r1, whole genome shotgun sequence	vertebrates_non_mammals'
ABPO01000001.1 Saccharomyces pastorianus Weihenstephan 34/70 Contig9.2, whole genome shotgun sequence	fungi'
CM002639.1 Oryza barthii chromosome 1, whole genome shotgun sequence	plants'
ACCZ01000471.1 Nothobranchius furzeri strain MZM-0403 clone Nf3-G-g-05c12 Nf3-G-g-05c12.f1, whole genome shotgun sequence	vertebrates_non_mammals'
ACFL01000033.1 Saccharomyces cerevisiae JAY291 contig245_180, whole genome shotgun sequence	fungi'

KQ257450.1 Spizellomyces punctatus DAOM BR117 chromosome Unknown supercont1.1, whole genome shotgun sequence	fungi'
CP001408.1 Burkholderia pseudomallei MSHR346 chromosome I, complete sequence	bacteria'
HE605206.1 Candida parapsilosis strain CDC317 annotated contig 005809	fungi'
CABT02000001.1 Sordaria macrospora k-hell, WGS project CABT00000000 data, contig 2.1, whole genome shotgun sequence	fungi'
CP002338.1 Lactobacillus amylovorus GRL 1112, complete genome	bacteria'
CM002236.1 Neurospora crassa OR74A linkage group I, whole genome shotgun sequence	fungi'
CP017623.1 Candida albicans SC5314 chromosome 1 sequence	fungi'
ABRE01017988.1 Moniliophthora perniciosa FA553 Contig18273, whole genome shotgun sequence	fungi'
AABS01000001.1 Ciona intestinalis ciona0001, whole genome shotgun sequence	invertebrates'
CM001487.1 Eubacterium cellulosolvens 6 chromosome, whole genome shotgun sequence	bacteria'
JH712066.1 Loa loa unplaced genomic scaffold supercont3.1, whole genome shotgun sequence	invertebrates'
CM001046.1 Listeria monocytogenes FSL F2-208 chromosome, whole genome shotgun sequence	bacteria'
CM001048.1 Listeria innocua FSL S4-378 chromosome, whole genome shotgun sequence	bacteria'
CM001049.1 Listeria innocua FSL J1-023 chromosome, whole genome shotgun sequence	bacteria'
CM001050.1 Listeria ivanovii FSL F6-596 chromosome, whole genome shotgun sequence	bacteria'
CM001051.1 Listeria seeligeri FSL N1-067 chromosome, whole genome shotgun sequence	bacteria'
CM001052.1 Listeria seeligeri FSL S4-171 chromosome, whole genome shotgun sequence	bacteria'
CM001043.1 Mycobacterium tuberculosis HN878 chromosome, whole genome shotgun sequence	bacteria'
CM001044.1 Mycobacterium tuberculosis X122 chromosome, whole genome shotgun sequence	bacteria'
CM001045.1 Mycobacterium tuberculosis R1207 chromosome, whole genome shotgun sequence	bacteria'
GL573169.1 Geomyces destructans 20631-21 unplaced genomic scaffold supercont1.1, whole genome shotgun sequence	fungi'
CM001058.1 Fragaria vesca subsp. vesca linkage group LG6, whole genome shotgun sequence	plants'
BA000049.1 Aspergillus oryzae RIB40 DNA, chromosome 1	fungi'
GL577060.1 Apis florea unplaced genomic scaffold scaffold02040, whole genome shotgun sequence	invertebrates'
JH801365.1 Mus musculus strain C57BL/6J unplaced genomic scaffold scaffold00001, whole genome shotgun sequence	vertebrates_mammals'
GL589130.1 Mus musculus strain C57BL/6J unplaced genomic scaffold scaffold_0, whole genome shotgun sequence	vertebrates_mammals'
GL582980.1 Homo sapiens unplaced genomic scaffold scaffold_0, whole genome shotgun sequence	vertebrates_mammals'
CP000289.1 Cryptococcus gattii WM276 chromosome D, complete sequence	fungi'
GG663757.1 Plasmodium falciparum RAJ116 genomic scaffold supercont1.2, whole genome shotgun sequence	protozoa'
GG664959.1 Plasmodium falciparum IGH-CR14 genomic scaffold supercont1.1, whole genome shotgun sequence	protozoa'
CM000488.1 Bacillus subtilis subsp. subtilis str. NCIB 3610 chromosome, whole genome shotgun sequence	bacteria'
KE952971.1 Python bivittatus unplaced genomic scaffold Scaffold0, whole genome shotgun sequence	vertebrates_non_mammals'
GL637601.1 Caenorhabditis tropicalis strain JU1373 unplaced genomic scaffold Scaffold629, whole genome shotgun sequence	invertebrates'
GL833120.1 Aureococcus anophagefferens unplaced genomic scaffold AURANscaffold_1, whole genome shotgun sequence	protozoa'
AEUI02000001.1 Ascaris suum AG00001, whole genome shotgun sequence	invertebrates'
CM002306.1 Ogataea parapolymorpha DL-1 chromosome VII, whole genome shotgun sequence	fungi'
GL698470.1 Metarhizium acridum CQMa 102 unplaced genomic scaffold Scf_001, whole genome shotgun sequence	fungi'
ADNJ02000001.1 Metarhizium robertsii ARSEF 23 MAA_Scf_1, whole genome shotgun sequence	fungi'
CM001062.1 Salmonella enterica subsp. enterica serovar Choleraesuis str. A50 chromosome, whole genome shotgun sequence	bacteria'
GL732523.1 Daphnia pulex unplaced genomic scaffold DAPPUscaffold_1, whole genome shotgun sequence	invertebrates'
GL738599.1 Pogonomyrmex barbatus unplaced genomic scaffold scf7180000350380, whole genome shotgun sequence	invertebrates'
AEUT02000001.1 Streptococcus parauberis NCFD 2020 chromosome, whole genome shotgun sequence	bacteria'
GL764026.1 Solenopsis invicta unplaced genomic scaffold Si_gnG.scaffold02694, whole genome shotgun sequence	invertebrates'
JH157811.1 Bombus impatiens unplaced genomic scaffold scf_0102, whole genome shotgun sequence	invertebrates'
CM001064.2 Solanum lycopersicum cultivar Heinz 1706 chromosome 1, whole genome shotgun sequence	plants'
CM001450.1 Oreochromis niloticus linkage group LG7, whole genome shotgun sequence	vertebrates_non_mammals'
CM001076.1 Streptococcus dysgalactiae subsp. dysgalactiae ATCC 27957 chromosome, whole genome shotgun sequence	bacteria'
GL832955.1 Salpingoeca sp. ATCC 50818 unplaced genomic scaffold supercont1.1, whole genome shotgun sequence	invertebrates'
GL849905.1 Sarcophilus harrisii chromosome 3 unlocalized genomic scaffold Chr3_supercontig_000000383, whole genome shotgun sequence	vertebrates_mammals'
GG662845.1 Tetrahymena thermophila SB210 genomic scaffold scf_8254803, whole genome shotgun sequence	protozoa'
CM001081.1 Saccharomyces cerevisiae AWRI796 chromosome IV, whole genome shotgun sequence	fungi'
CM001097.1 Saccharomyces cerevisiae Vin13 chromosome IV, whole genome shotgun sequence	fungi'
CM001129.1 Saccharomyces cerevisiae VL3 chromosome IV, whole genome shotgun sequence	fungi'
CM001113.1 Saccharomyces cerevisiae FostersB chromosome IV, whole genome shotgun sequence	fungi'
GL870876.1 Nematocida parisii ERTm3 unplaced genomic scaffold supercont1.1, whole genome shotgun sequence	fungi'
GL870941.1 Dictyostelium purpureum unplaced genomic scaffold DICPUscaffold_1, whole genome shotgun sequence	protozoa'
CM001142.1 Escherichia fergusonii ECD227 chromosome, whole genome shotgun sequence	bacteria'
CM001148.1 Parascardovia denticolens DSM 10105 chromosome, whole genome shotgun sequence	bacteria'
CM001151.1 Salmonella enterica subsp. enterica serovar Dublin str. 3246 chromosome, whole genome shotgun sequence	bacteria'
CM001153.1 Salmonella enterica subsp. enterica serovar Gallinarum str. 9 chromosome, whole genome shotgun sequence	bacteria'
GL872453.1 Saccharomyces cerevisiae Y10 unplaced genomic scaffold Scfld1, whole genome shotgun sequence	fungi'
GL873277.1 Saccharomyces cerevisiae CBS 7960 unplaced genomic scaffold Scaffold1, whole genome shotgun sequence	fungi'
GL875079.1 Saccharomyces cerevisiae CLIB324 unplaced genomic scaffold Scfld0, whole genome shotgun sequence	fungi'
GL875736.1 Saccharomyces cerevisiae YJM269 unplaced genomic scaffold Scfld1, whole genome shotgun sequence	fungi'
GL875992.1 Saccharomyces cerevisiae FL100 unplaced genomic scaffold Scaffold1, whole genome shotgun sequence	fungi'
GL876586.1 Saccharomyces cerevisiae CLIB215 unplaced genomic scaffold Scaffold1, whole genome shotgun sequence	fungi'
GL877404.1 Vavraia culicis subsp. floridensis unplaced genomic scaffold supercont1.1, whole genome shotgun sequence	fungi'
GL877354.1 Acanthamoeba castellanii strain Neff unplaced genomic scaffold SCAFFOLD1185, whole genome shotgun sequence	protozoa'
GL876966.1 Magnaporthe poae ATCC 64411 unplaced genomic scaffold supercont1.1, whole genome shotgun sequence	fungi'
CM000832.1 Burkholderia pseudomallei 1710a chromosome I, whole genome shotgun sequence	bacteria'
CM000774.1 Burkholderia pseudomallei 1106b chromosome I, whole genome shotgun sequence	bacteria'
CM001156.1 Burkholderia pseudomallei Bp22 chromosome I, whole genome shotgun sequence	bacteria'
FP885897.1 Ralstonia solanacearum CFBP2957 chromosome complete genome	bacteria'
GL882879.1 Batrachochytrium dendrobatidis JAM81 unplaced genomic scaffold BATDEscaffold_1, whole genome shotgun sequence	fungi'
GL883010.1 Dictyostelium fasciculatum unplaced genomic scaffold Scf_Dfasci-F-a-25c12.r2-5, whole genome shotgun sequence	protozoa'
GL883090.1 Melampsora larici-populina 98AG31 unplaced genomic scaffold MELLAscaffold_1, whole genome shotgun sequence	fungi'
GL888128.1 Acromyrmex echinatior unplaced genomic scaffold scaffold244, whole genome shotgun sequence	invertebrates'
DS891538.1 Ixodes scapularis 1108463063928 genomic scaffold, whole genome shotgun sequence	invertebrates'
JH573670.1 Dasypus novemcinctus unplaced genomic scaffold Scaffold30424, whole genome shotgun sequence	vertebrates_mammals'
DF145384.1 Jatropha curcas DNA, scaffold: Jcr4S00002, strain: Palawan, whole genome shotgun sequence	plants'
LT594789.1 Theobroma cacao genome assembly, chromosome: II	plants'
FR823391.1 Neospora caninum Liverpool complete genome, chromosome X	protozoa'
DS571145.1 Entamoeba histolytica HM-1:IMSS scf_1104750518587 genomic scaffold, whole genome shotgun sequence	protozoa'
CH473309.1 Trypanosoma cruzi strain CL Brener 1047053517087 genomic scaffold, whole genome shotgun sequence	protozoa'
DS548800.1 Entamoeba dispar SAW760 scf_1105229459701 genomic scaffold, whole genome shotgun sequence	protozoa'
CM000437.1 Pichia stipitis CBS 6054 chromosome 1, whole genome shotgun sequence	fungi'
DS231813.1 Culex pipiens quinquefasciatus supercont3.1 genomic scaffold, whole genome shotgun sequence	invertebrates'
DS469507.1 Nematostella vectensis NEMVEscaffold_1 genomic scaffold, whole genome shotgun sequence	invertebrates'
GL889000.1 Saccharomyces cerevisiae PW5 unplaced genomic scaffold Scfld20, whole genome shotgun sequence	fungi'
GL889064.1 Saccharomyces cerevisiae UC5 unplaced genomic scaffold Scfld39, whole genome shotgun sequence	fungi'
GL889345.1 Saccharomyces cerevisiae T73 unplaced genomic scaffold Scfld284, whole genome shotgun sequence	fungi'
GL889895.1 Saccharomyces cerevisiae CLIB382 unplaced genomic scaffold Scfld402, whole genome shotgun sequence	fungi'
GL890460.1 Saccharomyces cerevisiae T7 unplaced genomic scaffold Scfld0, whole genome shotgun sequence	fungi'
CH991540.1 Enterocytozoon bieneusi H348 SC_2496 genomic scaffold, whole genome shotgun sequence	fungi'
FN653015.1 Oikopleura dioica, whole genome shotgun assembly, reference scaffold set, scaffold scaffold_1, whole genome shotgun sequence	invertebrates'
FN654275.1 Oikopleura dioica, whole genome shotgun assembly, allelic scaffold set, scaffold scaffoldA_1, whole genome shotgun sequence	invertebrates'
BX526834.1 Cryptosporidium parvum chromosome 6, complete sequence	protozoa'
AP009608.1 Mycoplasma fermentans PG18 DNA, nearly complete genome	bacteria'
FP929036.1 Butyrivibrio fibrisolvens 16/4 draft genome	bacteria'
FP929053.1 Ruminococcus sp. SR1/5 draft genome	bacteria'
FP929046.1 Faecalibacterium prausnitzii SL3/3 draft genome	bacteria'
FP929051.1 Ruminococcus bromii L2-63 draft genome	bacteria'
FP929062.1 Clostridiales sp. SS3/4 draft genome	bacteria'
FP929044.1 Eubacterium siraeum 70/3 draft genome	bacteria'
FP929042.1 Eubacterium rectale DSM 17629 draft genome	bacteria'
FP929043.1 Eubacterium rectale M104/1 draft genome	bacteria'
FP929048.1 Megamonas hypermegale ART12/1 draft genome	bacteria'
FP929049.1 Roseburia intestinalis M50/1 draft genome	bacteria'
FP929054.1 Ruminococcus obeum A2-162 draft genome	bacteria'
FP929055.1 Ruminococcus torques L2-14 draft genome	bacteria'
FP929047.1 Gordonibacter pamelaeae 7-10-1-b draft genome	bacteria'
FP929033.1 Bacteroides xylanisolvens XB1A draft genome	bacteria'
FP929052.1 Ruminococcus champanellensis type strain 18P13T draft genome	bacteria'
FP929058.1 Enterococcus sp. 7L76 draft genome	bacteria'
FN554974.1 Trypanosoma brucei gambiense DAL972 chromosome 11, complete sequence	protozoa'
FN645450.1 Triticum aestivum chromosome 3B-specific BAC library, contig ctg0011b	plants'
AP011615.1 Arthrospira platensis NIES-39, *** SEQUENCING IN PROGRESS ***, 19 ordered pieces	bacteria'
FN668375.1 Clostridium difficile M68, complete genome	bacteria'
FP929037.1 Clostridium saccharolyticum-like K10 draft genome	bacteria'
FP929038.1 Coprococcus catus GD/7 draft genome	bacteria'
FP929032.1 Alistipes shahii WAL 8301 draft genome	bacteria'
FP929039.1 Coprococcus sp. ART55/1 draft genome	bacteria'
FP929041.1 Eubacterium cylindroides T2-87 draft genome	bacteria'
FP929059.1 Eubacterium siraeum V10Sc8a draft genome	bacteria'
FP929050.1 Roseburia intestinalis XB6B4 draft genome	bacteria'
FP929060.1 Clostridiales sp. SM4/1 draft genome	bacteria'
FP929061.1 Anaerostipes hadrus draft genome	bacteria'
FP929056.1 Fretibacterium fastidiosum draft genome	bacteria'
FP929045.1 Faecalibacterium prausnitzii L2/6 draft genome	bacteria'
FP929034.1 Bifidobacterium longum subsp. longum F8 draft genome	bacteria'
FP929040.1 Enterobacter cloacae subsp. cloacae NCTC 9394 draft genome	bacteria'
FR720325.1 Listeria monocytogenes serotype 7 str. SLCC2482 complete genome	bacteria'
FQ482149.1 Chlamydophila psittaci RD1 genome	bacteria'
BA000015.5 Arabidopsis thaliana DNA, chromosome 5, complete sequence	plants'
ADNL01003213.1 Astrammina rara contig02625, whole genome shotgun sequence	protozoa'
CM001159.1 Listeria monocytogenes str. Scott A chromosome, whole genome shotgun sequence	bacteria'
CM001161.1 Rhodobacter sphaeroides WS8N chromosome chrI, whole genome shotgun sequence	bacteria'
CM001167.1 Bacteroides coprosuis DSM 18011 chromosome, whole genome shotgun sequence	bacteria'
CM000492.1 Homo sapiens chromosome 2, whole genome shotgun sequence	vertebrates_mammals'
CM001165.1 Streptomyces sp. Tu6071 chromosome, whole genome shotgun sequence	bacteria'
GL891382.1 Neurospora tetrasperma FGSC 2508 unplaced genomic scaffold NEUTE1scaffold_81, whole genome shotgun sequence	fungi'
GL890999.1 Neurospora tetrasperma FGSC 2509 unplaced genomic scaffold NEUTE2scaffold_1, whole genome shotgun sequence	fungi'
CM001168.1 Chlamydophila abortus LLG chromosome, whole genome shotgun sequence	bacteria'
CAID01000001.2 Ostreococcus tauri WGS project CAID00000000 data, contig chromosome 01, whole genome shotgun sequence	plants'
CM001175.1 Bombus terrestris linkage group LG B07, whole genome shotgun sequence	invertebrates'

CM001475.1 Methylomicrobium album BG8 chromosome, whole genome shotgun sequence	bacteria'
GL896898.1 Mustela putorius furo unplaced genomic scaffold scaffold00001, whole genome shotgun sequence	vertebrates_mammals'
GL905323.1 Linepithema humile unplaced genomic scaffold scf7180001005018, whole genome shotgun sequence	invertebrates'
GL913935.1 Prunus persica unplaced genomic scaffold DD_scaffold1, whole genome shotgun sequence	plants'
GL931709.1 Prunus persica unplaced genomic scaffold GB_scaffold1, whole genome shotgun sequence	plants'
GL921761.1 Prunus persica unplaced genomic scaffold F8_scaffold1, whole genome shotgun sequence	plants'
CM001187.1 Eutrema parvulum chromosome 1, whole genome shotgun sequence	plants'
CM002368.1 Fusobacterium nucleatum subsp. animalis 21_1A chromosome, whole genome shotgun sequence	bacteria'
GL945428.1 Serpula lacrymans var. lacrymans S7.9 unplaced genomic scaffold SERLAscaffold_1, whole genome shotgun sequence	fungi'
GL945474.1 Serpula lacrymans var. lacrymans S7.3 unplaced genomic scaffold SERLA73scaffold_1, whole genome shotgun sequence	fungi'
FN393063.1 Saccharomyces cerevisiae EC1118 chromosome IV, EC1118_1D0 genomic scaffold, whole genome shotgun sequence	fungi'
EQ258278.1 Hydra magnipapillata scf_1101284939866 genomic scaffold, whole genome shotgun sequence	invertebrates'
CM001194.1 Acinetobacter lwoffii WJ10621 chromosome, whole genome shotgun sequence	bacteria'
CM001220.2 Medicago truncatula strain A17 chromosome 4, whole genome shotgun sequence	plants'
CM001196.1 Mycosphaerella graminicola IPO323 chromosome 1, whole genome shotgun sequence	fungi'
CM001195.1 Bradyrhizobiaceae bacterium SG-6C chromosome, whole genome shotgun sequence	bacteria'
GL982313.1 Sarcophilus harrisii unplaced genomic scaffold scf7180002673897, whole genome shotgun sequence	vertebrates_mammals'
AGTD01000001.1 Escherichia coli UMNF18 chromosome, whole genome shotgun sequence	bacteria'
GL983042.1 Ichthyophthirius multifiliis unplaced genomic scaffold scaff_1120509249393, whole genome shotgun sequence	protozoa'
CM001225.1 Mycobacterium tuberculosis S96-129 chromosome, whole genome shotgun sequence	bacteria'
CM001226.1 Mycobacterium tuberculosis BTB05-552 chromosome, whole genome shotgun sequence	bacteria'
CM001227.1 Mycobacterium tuberculosis BTB05-559 chromosome, whole genome shotgun sequence	bacteria'
KB457523.1 Drosophila ficusphila unplaced genomic scaffold scf7180000454073, whole genome shotgun sequence	invertebrates'
GL985818.1 Megachile rotundata unplaced genomic scaffold scf_0147, whole genome shotgun sequence	invertebrates'
GL988041.1 Chaetomium thermophilum var. thermophilum DSM 1495 unplaced genomic scaffold scf7180000011816, whole genome shotgun sequence	fungi'
FR874244.1 Macaca fascicularis draft chromosome, chr1, whole genome shotgun sequence	vertebrates_mammals'
AEVR02000013.1 Rhodotorula toruloides ATCC 204091 scaffold00013, whole genome shotgun sequence	fungi'
GL990379.1 Arabidopsis thaliana ecotype Bur-0 unplaced genomic scaffold Scaffold_2223, whole genome shotgun sequence	plants'
GL992820.1 Arabidopsis thaliana ecotype C24 unplaced genomic scaffold Scaffold_1951, whole genome shotgun sequence	plants'
GL991508.1 Arabidopsis thaliana ecotype LER-1 unplaced genomic scaffold Scaffold_612, whole genome shotgun sequence	plants'
GL995175.1 Arabidopsis thaliana ecotype Kro-0 unplaced genomic scaffold Scaffold_4779, whole genome shotgun sequence	plants'
CM001228.1 Campylobacter fetus subsp. venerealis NCTC 10354 chromosome, whole genome shotgun sequence	bacteria'
DF970692.1 Acropora digitifera DNA, scaffold: Scaffold1, whole genome shotgun sequence	invertebrates'
JH000001.1 Cricetulus griseus cell line CHO-K1 unplaced genomic scaffold scaffold329, whole genome shotgun sequence	vertebrates_mammals'
GL996469.1 Heterorhabditis bacteriophora unplaced genomic scaffold Scaffold1346, whole genome shotgun sequence	invertebrates'
GL996527.1 Candida tenuis ATCC 10573 unplaced genomic scaffold CANTEscaffold_00021, whole genome shotgun sequence	fungi'
GL996499.1 Spathaspora passalidarum NRRL Y-27907 unplaced genomic scaffold SPAPAscaffold_1, whole genome shotgun sequence	fungi'
AECO01000197.1 Zea mays subsp. mays, whole genome shotgun sequence	plants'
FR839628.1 Pichia pastoris CBS 7435 chromosome 1, complete replicon sequence	fungi'
KK211454.1 Gregarina niphandrodes unplaced genomic scaffold scaffold00001, whole genome shotgun sequence	protozoa'
AFXJ01000001.1 Pseudomonas aeruginosa 19BR chromosome, whole genome shotgun sequence	bacteria'
AFXK01000001.1 Pseudomona aeruginosa 213BR chromosome, whole genome shotgun sequence	bacteria'
JH113294.1 Cucumis sativus unplaced genomic scaffold CSB10A_v1_scaffold_1, whole genome shotgun sequence	plants'
HT000001.2 TPA_asm: Ciona intestinalis DNA, scaffold: KhC1, whole genome shotgun sequence	invertebrates'
KB458607.1 Drosophila elegans unplaced genomic scaffold scf7180000491249, whole genome shotgun sequence	invertebrates'
KB459691.1 Drosophila kikkawai unplaced genomic scaffold scf7180000302476, whole genome shotgun sequence	invertebrates'
KB461572.1 Drosophila takahashii unplaced genomic scaffold scf7180000415769, whole genome shotgun sequence	invertebrates'
KN039985.1 Toxoplasma gondii RUB unplaced genomic scaffold scaffold00001, whole genome shotgun sequence	protozoa'
KN042471.1 Toxoplasma gondii VAND unplaced genomic scaffold scaffold00001, whole genome shotgun sequence	protozoa'
KN044612.1 Toxoplasma gondii MAS unplaced genomic scaffold scaffold00002, whole genome shotgun sequence	protozoa'
KN000222.1 Toxoplasma gondii p89 unplaced genomic scaffold scaffold00001, whole genome shotgun sequence	protozoa'
KN000235.1 Toxoplasma gondii FOU unplaced genomic scaffold scaffold00001, whole genome shotgun sequence	protozoa'
JH119053.1 Arthrobotrys oligospora ATCC 24927 unplaced genomic scaffold scaffold00054, whole genome shotgun sequence	fungi'
CM001230.1 Brenneria sp. EniD312 chromosome, whole genome shotgun sequence	bacteria'
JH126399.1 Cordyceps militaris CM01 unplaced genomic scaffold CCM_S00001, whole genome shotgun sequence	fungi'
JH126562.1 Latimeria chalumnae unplaced genomic scaffold scaffold00001, whole genome shotgun sequence	vertebrates_non_mammals'
JH154184.1 Mnemiopsis leidyi unplaced genomic scaffold ML1541, whole genome shotgun sequence	invertebrates'
JH137791.1 Solanum tuberosum unplaced genomic scaffold scf00001, whole genome shotgun sequence	plants'
CP003002.1 Myceliophthora thermophila ATCC 42464 chromosome 1, complete sequence	fungi'
CP003009.1 Thielavia terrestris NRRL 8126 chromosome 1, complete sequence	fungi'
KV751188.1 Epichloe festucae Fl1 unplaced genomic scaffold scaffold00001, whole genome shotgun sequence	fungi'
AM920437.1 Penicillium chrysogenum Wisconsin 54-1255 complete genome, contig Pc00c22	fungi'
CU640366.1 Podospora anserina S mat+ genomic DNA chromosome 2, supercontig 2	fungi'
DF093573.1 Aspergillus sojae NBRC 4239 DNA, scaffold: 00036, whole genome shotgun sequence	fungi'
FQ790278.1 Botryotinia fuckeliana T4 SuperContig_34_1 genomic supercontig	fungi'
HE580267.1 Naumovozyma dairenensis CBS 421 chromosome 1, complete genome	fungi'
FR799623.2 Leishmania donovani BPK282A1 complete genome, chromosome 36	protozoa'
CAEQ01001170.1 Trypanosoma congolense IL3000 WGS project CAEQ00000000 data, annotated contig 1735, whole genome shotgun sequence	protozoa'
FQ311430.1 Sporisorium reilianum SRZ2 chromosome 1 complete DNA sequence	fungi'
FP929139.1 Leptosphaeria maculans JN3 lm_SuperContig_0_v2 genomic supercontig, whole genome, isolate v23.1.3	fungi'
JH173571.1 Heterocephalus glaber unplaced genomic scaffold scaffold208, whole genome shotgun sequence	vertebrates_mammals'
JH208680.1 Pelodiscus sinensis unplaced genomic scaffold scaffold470, whole genome shotgun sequence	vertebrates_non_mammals'
JH226299.1 Cannabis sativa unplaced genomic scaffold scaffold5190, whole genome shotgun sequence	plants'
AFBI03000001.1 Edhazardia aedis USNM 41457 supercont1.1, whole genome shotgun sequence	fungi'
JH226130.1 Exophiala dermatitidis NIH/UT8656 unplaced genomic scaffold supercont1.1, whole genome shotgun sequence	fungi'
CM001253.1 Macaca mulatta chromosome 1, whole genome shotgun sequence	vertebrates_mammals'
CM001276.1 Macaca fascicularis chromosome 1, whole genome shotgun sequence	vertebrates_mammals'
CM001240.1 Clostridium sp. DL-VIII chromosome, whole genome shotgun sequence	bacteria'
CM001274.1 Salmonella enterica subsp. enterica serovar Infantis str. SARB27 chromosome, whole genome shotgun sequence	bacteria'
CM001466.1 Saccharomonospora azurea NA-128 chromosome, whole genome shotgun sequence	bacteria'
CM001241.2 Oryza brachyantha chromosome 1, whole genome shotgun sequence	plants'
JH370130.1 Vittaforma corneae ATCC 50505 unplaced genomic scaffold supercont1.1, whole genome shotgun sequence	fungi'
HE569754.1 Gadus morhua draft genomic scaffold, scaffold07711, whole genome shotgun sequence	vertebrates_non_mammals'
JH720293.1 Tetrahymena elliotti 4EA unplaced genomic scaffold supercont2.1, whole genome shotgun sequence	protozoa'
JH719946.1 Tetrahymena malaccensis 436 unplaced genomic scaffold supercont2.1, whole genome shotgun sequence	protozoa'
CM003279.1 Salmo salar isolate Sally breed double haploid chromosome ssa01, whole genome shotgun sequence	vertebrates_non_mammals'
KB462838.1 Drosophila biarmipes unplaced genomic scaffold scf7180000302428, whole genome shotgun sequence	invertebrates'
CM001488.1 Desulfobacter postgatei 2ac9 chromosome, whole genome shotgun sequence	bacteria'
JH376955.1 Saccharomyces cerevisiae EC9-8 unplaced genomic scaffold scaffold00001, whole genome shotgun sequence	fungi'
FR799573.1 Leishmania mexicana MHOM/GT/2001/U1103 complete genome, chromosome 20	protozoa'
CP002498.1 Eremothecium cymbalariae DBVPG#7215 chromosome 2, complete sequence	fungi'
JH378105.1 Saimiri boliviensis boliviensis unplaced genomic scaffold scaffold00001, whole genome shotgun sequence	vertebrates_mammals'
JH392917.1 Rhizophydiales sp. JEL142 unplaced genomic scaffold JEL142_assembled86, whole genome shotgun sequence	fungi'
JH379599.1 Danaus plexippus unplaced genomic scaffold scaffold1, whole genome shotgun sequence	invertebrates'
CM001371.1 Fluoribacter dumoffii Tex-KL chromosome, whole genome shotgun sequence	bacteria'
CM001373.1 Fluoribacter dumoffii NY 23 chromosome, whole genome shotgun sequence	bacteria'
JH393279.1 Spermophilus tridecemlineatus unplaced genomic scaffold scaffold00001, whole genome shotgun sequence	vertebrates_mammals'
KB464369.1 Drosophila bipectinata unplaced genomic scaffold scf7180000396708, whole genome shotgun sequence	invertebrates'
KB452396.1 Drosophila rhopaloa unplaced genomic scaffold scf7180000779985, whole genome shotgun sequence	invertebrates'
KB465221.1 Drosophila eugracilis unplaced genomic scaffold scf7180000409672, whole genome shotgun sequence	invertebrates'
DF142838.1 Clonorchis sinensis DNA, scaffold: 11, strain: Henan, whole genome shotgun sequence	invertebrates'
CP003147.1 Pseudovibrio sp. FO-BEG1, complete genome	bacteria'
HE612856.1 Tetrapisispora phaffii CBS 4417 chromosome 1, complete genome	fungi'
FR871757.1 Helicobacter bizzozeronii CIII-1 main chromosome, complete genome	bacteria'
HE576752.1 Naumovozyma castellii CBS 4309 chromosome 1, complete genome	fungi'
HE577327.1 Azospirillum brasilense Sp245 main chromosome complete genome	bacteria'
CM001376.1 Jonquetella anthropi DSM 22815 chromosome, whole genome shotgun sequence	bacteria'
CM001377.1 Thermanaerovibrio velox DSM 12556 chromosome, whole genome shotgun sequence	bacteria'
HE601624.1 Schistosoma mansoni strain Puerto Rico chromosome 1, complete genome	invertebrates'
JH413862.1 Glarea lozoyensis 74030 unplaced genomic scaffold M7I_scaffold12, whole genome shotgun sequence	fungi'
JH556617.1 Melopsittacus undulatus unplaced genomic scaffold budgerigar_v6.3_scf900160277070, whole genome shotgun sequence	vertebrates_non_mammals'
AGTA03000001.1 Maylandia zebra isolate 2_Kocher and #3_Kocher Contig0, whole genome shotgun sequence	vertebrates_non_mammals'
JH417973.1 Mustela putorius furo unplaced genomic scaffold scaffold00001, whole genome shotgun sequence	vertebrates_mammals'
JH419206.1 Pundamilia nyererei unplaced genomic scaffold scaffold00001, whole genome shotgun sequence	vertebrates_non_mammals'
JH422273.1 Neolamprologus brichardi unplaced genomic scaffold scaffold00001, whole genome shotgun sequence	vertebrates_non_mammals'
JH425323.1 Haplochromis burtoni unplaced genomic scaffold scaffold00002, whole genome shotgun sequence	vertebrates_non_mammals'
HE587301.1 Tetranychus urticae London genomic scaffold, scaffold_1, whole genome shotgun sequence	invertebrates'
JH432010.1 Strigamia maritima strain Brora unplaced genomic scaffold scf7180001248676, whole genome shotgun sequence	invertebrates'
KN955244.1 Lytechinus variegatus isolate Lvar 01 unplaced genomic scaffold Scaffold93, whole genome shotgun sequence	invertebrates'
DF126447.1 Aspergillus kawachii IFO 4308 DNA, contig: scaffold00001, whole genome shotgun sequence	fungi'
CM000574.1 Fusarium graminearum PH-1 chromosome 1, whole genome shotgun sequence	fungi'
JH470562.1 Nannochloropsis gaditana CCMP526 unplaced genomic scaffold scaffold001, whole genome shotgun sequence	protozoa'
CM001398.1 Oenococcus kitaharae DSM 17330 chromosome, whole genome shotgun sequence	bacteria'
JH556661.1 Xiphophorus maculatus strain JP 163 A unplaced genomic scaffold Scfld0, whole genome shotgun sequence	vertebrates_non_mammals'
AGTM011526392.1 Daubentonia madagascariensis isolate Dm6514m contig_1529285, whole genome shotgun sequence	vertebrates_mammals'
CM002655.1 Chrysemys picta bellii isolate RCT428 chromosome 1, whole genome shotgun sequence	vertebrates_non_mammals'
CM001400.1 Vibrio fischeri SR5 chromosome I, whole genome shotgun sequence	bacteria'
CM001402.1 Caldithrix abyssi DSM 13497 chromosome, whole genome shotgun sequence	bacteria'
CP003244.1 Rahnella aquatilis CIP 78.65 = ATCC 33071, complete genome	bacteria'
CM001407.1 Lepisosteus oculatus linkage group LG4, whole genome shotgun sequence	vertebrates_non_mammals'
AFRZ01000001.1 Sulfurimonas gotlandica GD1 SMGD1, whole genome shotgun sequence	bacteria'
CM001437.1 Myroides odoratus DSM 2801 chromosome, whole genome shotgun sequence	bacteria'
JH594607.1 Trichechus manatus latirostris unplaced genomic scaffold scaffold00001, whole genome shotgun sequence	vertebrates_mammals'
HE616742.1 Torulaspora delbrueckii CBS 1146 chromosome 1, complete genome	fungi'
CM001484.1 Saccharomonospora glauca K62 chromosome, whole genome shotgun sequence	bacteria'
CM001441.1 Desulfosporosinus youngiae DSM 17734 chromosome, whole genome shotgun sequence	bacteria'
CM001442.1 Bradyrhizobium sp. WSM471 chromosome, whole genome shotgun sequence	bacteria'
CM001439.1 Saccharomonospora marina XMU15 chromosome, whole genome shotgun sequence	bacteria'
CM001440.1 Saccharomonospora cyanea NA-134 chromosome, whole genome shotgun sequence	bacteria'
CM001438.1 Burkholderiales bacterium JOSHI_001 chromosome, whole genome shotgun sequence	bacteria'
CP002374.1 Treponema pallidum subsp. pertenue str. SamoaD, complete genome	bacteria'
CP002375.1 Treponema pallidum subsp. pertenue str. CDC2, complete genome	bacteria'
CP002376.1 Treponema pallidum subsp. pertenue str. Gauthier, complete genome	bacteria'
CP003115.1 Treponema pallidum subsp. pallidum DAL-1, complete genome	bacteria'
KI669561.1 Phytophthora parasitica INRA-310 unplaced genomic scaffold supercont2.1, whole genome shotgun sequence	protozoa'

JH602043.1 Heterocephalus glaber unplaced genomic scaffold scaffold00001, whole genome shotgun sequence	vertebrates_mammals'
CM003021.1 Bos indicus isolate QUIL7308 breed Nelore chromosome 1, whole genome shotgun sequence	vertebrates_mammals'
CM001990.1 Ficedula albicollis isolate OC2 chromosome 2, whole genome shotgun sequence	vertebrates_non_mammals'
JH604633.1 Nematocida sp. 1 ERTm2 unplaced genomic scaffold supercont1.1, whole genome shotgun sequence	fungi'
CM001469.1 Listeria monocytogenes FSL J1-208 chromosome, whole genome shotgun sequence	bacteria'
JH605123.1 Leishmania major strain SD 75.1 unplaced genomic scaffold Scfld36, whole genome shotgun sequence	protozoa'
KQ973609.1 Toxoplasma gondii ARI unplaced genomic scaffold scaffold00003, whole genome shotgun sequence	protozoa'
JH605023.1 Nematocida parisii ERTm1 unplaced genomic scaffold supercont1.1, whole genome shotgun sequence	fungi'
CM001471.1 Salmonella enterica subsp. houtenae str. ATCC BAA-1581 chromosome, whole genome shotgun sequence	bacteria'
CM000492.1 Homo sapiens chromosome 2, whole genome shotgun sequence	vertebrates_mammals'
CM001474.1 Shigella flexneri 5a str. M90T chromosome, whole genome shotgun sequence	bacteria'
FN667742.1 Xenorhabdus nematophila ATCC 19061 chromosome, complete genome	bacteria'
FR668087.1 Mycoplasma leachii 99/014/6 complete genome	bacteria'
FP475956.1 Thiomonas sp. str. 3As chromosome, complete genome	bacteria'
FR845719.1 Streptomyces venezuelae ATCC 10712 complete genome	bacteria'
JH621224.1 Metaseiulus occidentalis unplaced genomic scaffold scf7180000077524, whole genome shotgun sequence	invertebrates'
CM001467.1 Rickettsia helvetica C9P9 chromosome, whole genome shotgun sequence	bacteria'
CM001477.1 Clostridium perfringens F262 chromosome, whole genome shotgun sequence	bacteria'
JH622822.1 Saccharomyces kudriavzevii FM1056 unplaced genomic scaffold c2070, whole genome shotgun sequence	fungi'
KQ983268.1 Toxoplasma gondii TgCatPRC2 unplaced genomic scaffold scaffold00002, whole genome shotgun sequence	protozoa'
JH624252.1 Saccharomyces kudriavzevii FM1057 unplaced genomic scaffold c2070, whole genome shotgun sequence	fungi'
JH625538.1 Saccharomyces kudriavzevii FM1066 unplaced genomic scaffold c2072, whole genome shotgun sequence	fungi'
JH626928.1 Saccharomyces kudriavzevii FM1062 unplaced genomic scaffold c2070, whole genome shotgun sequence	fungi'
JH631522.1 Saccharomyces kudriavzevii FM1073 unplaced genomic scaffold c2070, whole genome shotgun sequence	fungi'
JH630079.1 Saccharomyces kudriavzevii FM1071 unplaced genomic scaffold c2070, whole genome shotgun sequence	fungi'
JH632993.1 Saccharomyces kudriavzevii FM1072 unplaced genomic scaffold c2070, whole genome shotgun sequence	fungi'
JH628500.1 Saccharomyces kudriavzevii FM1069 unplaced genomic scaffold c2070, whole genome shotgun sequence	fungi'
JH638420.1 Saccharomyces kudriavzevii FM1076 unplaced genomic scaffold c2070, whole genome shotgun sequence	fungi'
JH635991.1 Saccharomyces kudriavzevii FM1075 unplaced genomic scaffold c2072, whole genome shotgun sequence	fungi'
JH634502.1 Saccharomyces kudriavzevii FM1074 unplaced genomic scaffold c2070, whole genome shotgun sequence	fungi'
JH649412.1 Saccharomyces kudriavzevii FM1094 unplaced genomic scaffold c2070, whole genome shotgun sequence	fungi'
JH648157.1 Saccharomyces kudriavzevii FM1077 unplaced genomic scaffold c2070, whole genome shotgun sequence	fungi'
JH646584.1 Saccharomyces kudriavzevii FM1078 unplaced genomic scaffold c2070, whole genome shotgun sequence	fungi'
JH645006.1 Saccharomyces kudriavzevii FM1079 unplaced genomic scaffold c2070, whole genome shotgun sequence	fungi'
JH641959.1 Saccharomyces kudriavzevii ZP591 unplaced genomic scaffold c2070, whole genome shotgun sequence	fungi'
JH641353.1 Saccharomyces kudriavzevii IFO1803 unplaced genomic scaffold c2070, whole genome shotgun sequence	fungi'
JH643483.1 Saccharomyces kudriavzevii IFO10991 unplaced genomic scaffold c2070, whole genome shotgun sequence	fungi'
JH639923.1 Saccharomyces kudriavzevii IFO10990 unplaced genomic scaffold c2070, whole genome shotgun sequence	fungi'
JH929243.1 Entamoeba nuttalli P19 unplaced genomic scaffold asmbl_8287, whole genome shotgun sequence	protozoa'
KL544027.1 Hammondia hammondi strain H.H.34 unplaced genomic scaffold asmbl.1, whole genome shotgun sequence	protozoa'
CP012110.1 Bacillus licheniformis WX-02 genome	bacteria'
CM003385.1 Pan paniscus isolate Ulindi chromosome 2B, whole genome shotgun sequence	vertebrates_mammals'
CP003045.1 Staphylococcus aureus subsp. aureus 71193, complete genome	bacteria'
CM001483.1 Geobacillus thermoglucosidans TNO-09.020 chromosome, whole genome shotgun sequence	bacteria'
JH650723.1 Rhodosporidium toruloides MTCC 457 unplaced genomic scaffold contig610, whole genome shotgun sequence	fungi'
CM000166.1 Drosophila simulans strain white501 chromosome 3R, whole genome shotgun sequence	invertebrates'
CM000364.1 Drosophila simulans chromosome 3R, whole genome shotgun sequence	invertebrates'
CP003426.1 Borrelia crocidurae str. Achema, complete genome	bacteria'
JH650827.1 Fusarium oxysporum f. sp. lycopersici MN25 unplaced genomic scaffold supercont1.1, whole genome shotgun sequence	fungi'
JH650968.1 Fusarium oxysporum f. sp. pisi HDV247 unplaced genomic scaffold supercont1.1, whole genome shotgun sequence	fungi'
JH651225.1 Tetrahymena borealis unplaced genomic scaffold supercont1.1, whole genome shotgun sequence	protozoa'
JH651385.1 Fusarium oxysporum f. sp. radicis-lycopersici 26381 unplaced genomic scaffold supercont1.1, whole genome shotgun sequence	fungi'
JH657918.1 Fusarium oxysporum f. sp. vasinfectum 25433 unplaced genomic scaffold supercont1.1, whole genome shotgun sequence	fungi'
JH658272.1 Fusarium oxysporum f. sp. cubense tropical race 4 54006 unplaced genomic scaffold supercont1.1, whole genome shotgun sequence	fungi'
JH658799.1 Fusarium oxysporum f. sp. conglutinans race 2 54008 unplaced genomic scaffold supercont1.1, whole genome shotgun sequence	fungi'
JH658362.1 Fusarium oxysporum f. sp. raphani 54005 unplaced genomic scaffold supercont1.1, whole genome shotgun sequence	fungi'
JH651516.1 Octodon degus unplaced genomic scaffold scaffold00001, whole genome shotgun sequence	vertebrates_mammals'
JH655878.1 Condylura cristata unplaced genomic scaffold scaffold00001, whole genome shotgun sequence	vertebrates_mammals'
JH659329.1 Fusarium oxysporum f. sp. melonis 26406 unplaced genomic scaffold supercont1.1, whole genome shotgun sequence	fungi'
DG000040.1 Saccharomyces cerevisiae Kyokai no. 7 (K7) DNA, chromosome IV scaffold, whole genome shotgun sequence	fungi'
JH659590.1 Tetrahymena thermophila SB210 unplaced genomic scaffold supercont2.1, whole genome shotgun sequence	protozoa'
KL650049.1 Cladosporium sphaerospermum UM 843 unplaced genomic scaffold scaffold9, whole genome shotgun sequence	fungi'
CM001486.1 Thermoanaerobacter siderophilus SR4 chromosome, whole genome shotgun sequence	bacteria'
CM001489.1 Frankia sp. QA3 chromosome, whole genome shotgun sequence	bacteria'
JH668279.1 Manduca sexta unplaced genomic scaffold scaffold00001, whole genome shotgun sequence	invertebrates'
JH660758.1 Ganoderma lucidum Xiangnong No.1 unplaced genomic scaffold scaffold_80, whole genome shotgun sequence	fungi'
JH660948.1 Phlebotomus papatasi unplaced genomic scaffold Scaffold1, whole genome shotgun sequence	invertebrates'
CM001515.1 Mycobacterium tuberculosis H37RvCO chromosome, whole genome shotgun sequence	bacteria'
CM003536.1 Setaria italica strain Yugu1 chromosome IX, whole genome shotgun sequence	plants'
JH668223.1 Wallemia sebi CBS 633.66 unplaced genomic scaffold WALSEscaffold_1, whole genome shotgun sequence	fungi'
CM001513.1 Pseudomonas fluorescens SS101 chromosome, whole genome shotgun sequence	bacteria'
CM001512.1 Pseudomonas fluorescens Q8r1-96 chromosome, whole genome shotgun sequence	bacteria'
CM001514.1 Pseudomonas synxantha BG33R chromosome, whole genome shotgun sequence	bacteria'
CM001490.1 Pseudomonas chlororaphis O6 chromosome, whole genome shotgun sequence	bacteria'
CM001491.1 Papio anubis chromosome 1, whole genome shotgun sequence	vertebrates_mammals'
JH687379.1 Stereum hirsutum FP-91666 SS1 unplaced genomic scaffold STEHIscaffold_1, whole genome shotgun sequence	fungi'
JH687538.1 Punctularia strigosozonata HHB-11173 SS5 unplaced genomic scaffold PUNSTscaffold_1, whole genome shotgun sequence	fungi'
JH687733.1 Auricularia subglabra TFB-10046 SS5 unplaced genomic scaffold AURDEscaffold_1, whole genome shotgun sequence	fungi'
CP003563.1 Sinorhizobium fredii USDA 257, complete genome	bacteria'
JH689321.1 Lutzomyia longipalpis unplaced genomic scaffold Scaffold1, whole genome shotgun sequence	invertebrates'
CM001518.2 Drosophila miranda strain MSH22 chromosome 2, whole genome shotgun sequence	invertebrates'
AKBU01000001.1 Rhodobacter sphaeroides 2.4.1 chromosome 1, complete sequence, whole genome shotgun sequence	bacteria'
AKBV01000001.1 Escherichia coli str. K-12 substr. MG1655 chromosome, whole genome shotgun sequence	bacteria'
CM001525.1 Saccharomyces cerevisiae CEN.PK113-7D chromosome IV, whole genome shotgun sequence	fungi'
AP012200.1 Melissococcus plutonius ATCC 35311 DNA, complete genome	bacteria'
FN668944.1 Clostridium difficile BI9 chromosome	bacteria'
CM001538.1 Lactobacillus pentosus KCA1 chromosome, whole genome shotgun sequence	bacteria'
JH711484.1 Ganoderma lucidum G.260125-1 unplaced genomic scaffold GaLu96scf_1, whole genome shotgun sequence	fungi'
JH711783.1 Trametes versicolor FP-101664 SS1 unplaced genomic scaffold TRAVEscaffold_1, whole genome shotgun sequence	fungi'
JH717967.1 Fomitiporia mediterranea MF3/22 unplaced genomic scaffold FOMMEscaffold_1, whole genome shotgun sequence	fungi'
JH711573.1 Coniophora puteana RWD-64-598 SS2 unplaced genomic scaffold CONPUscaffold_1, whole genome shotgun sequence	fungi'
JH711528.1 Tremella mesenterica DSM 1558 unplaced genomic scaffold TREMEscaffold_1, whole genome shotgun sequence	fungi'
JH717896.1 Fusarium oxysporum Fo47 unplaced genomic scaffold supercont1.1, whole genome shotgun sequence	fungi'
JH717839.1 Fusarium oxysporum FOSC 3-a unplaced genomic scaffold supercont1.1, whole genome shotgun sequence	fungi'
CM001557.1 Bartonella birtlesii IBS 325 chromosome, whole genome shotgun sequence	bacteria'
AKVW01000001.1 Rhodobacter sphaeroides 2.4.1 chromosome 1, complete sequence, whole genome shotgun sequence	bacteria'
AKVX01000001.1 Escherichia coli str. K-12 substr. MG1655 chromosome, whole genome shotgun sequence	bacteria'
AKVY01000001.1 Streptococcus pneumoniae TIGR4 chromosome, whole genome shotgun sequence	bacteria'
AFRY01000001.1 Streptococcus pyogenes HKU QMH11M0907901 chromosome, whole genome shotgun sequence	bacteria'
AKGH01000001.1 Vibrio cholerae H1 chromosome 1, whole genome shotgun sequence	bacteria'
CM001542.1 Saccharomyces cerevisiae YJSH1 chromosome IV, whole genome shotgun sequence	fungi'
JH719397.1 Dichomitus squalens LYAD-421 SS1 unplaced genomic scaffold DICSQscaffold_1, whole genome shotgun sequence	fungi'
JH721862.1 Chinchilla lanigera unplaced genomic scaffold scaffold00001, whole genome shotgun sequence	vertebrates_mammals'
CP003394.1 Rickettsia prowazekii str. Dachau genome	bacteria'
JH722767.1 Dictyostelium intermedium strain PJ-11 unplaced genomic scaffold scaffold00001, whole genome shotgun sequence	protozoa'
JH723757.1 Dictyostelium firmibasis strain TNS-C-0014 unplaced genomic scaffold scaffold00001, whole genome shotgun sequence	protozoa'
CP002723.1 Encephalitozoon hellem ATCC 50504 chromosome X, complete sequence	fungi'
JH739887.1 Geospiza fortis unplaced genomic scaffold scaffold40, whole genome shotgun sequence	vertebrates_non_mammals'
CP003530.1 Encephalitozoon romaleae SJ-2008 chromosome XI, complete sequence	fungi'
JH725150.1 Beauveria bassiana ARSEF 2860 unplaced genomic scaffold BBA_S00001, whole genome shotgun sequence	fungi'
JH725437.1 Jaculus jaculus unplaced genomic scaffold scaffold00001, whole genome shotgun sequence	vertebrates_mammals'
JH767132.1 Saprolegnia diclina VS20 unplaced genomic scaffold supercont1.1, whole genome shotgun sequence	protozoa'
JH767554.1 Coniosporium apollinis CBS 100218 chromosome Unknown supercont1.1, whole genome shotgun sequence	fungi'
AKHW03000533.1 Alligator mississippiensis isolate KSC_2009_1 ScZkoYb_72, whole genome shotgun sequence	vertebrates_non_mammals'
CM001558.1 Pseudomonas fluorescens Q2-87 chromosome, whole genome shotgun sequence	bacteria'
CM001559.1 Pseudomonas chlororaphis subsp. aureofaciens 30-84 chromosome, whole genome shotgun sequence	bacteria'
CP007549.3 UNVERIFIED: Acinetobacter baumannii AC12 genome	bacteria'
JH767723.1 Ceratotherium simum simum unplaced genomic scaffold scaffold00001, whole genome shotgun sequence	vertebrates_mammals'
HE576794.1 Megasphaera elsdenii strain DSM 20460 draft genome	bacteria'
CP003318.1 Rickettsia rickettsii str. Hauke, complete genome	bacteria'
CP003319.1 Rickettsia massiliae str. AZT80, complete genome	bacteria'
HE616890.1 Sinorhizobium fredii HH103 main chromosome, complete sequence	bacteria'
JH768609.1 Patiria miniata unplaced genomic scaffold Scaffold2, whole genome shotgun sequence	invertebrates'
CAGI01000150.1 Ustilago hordei Uh4857-4 WGS project CAGI00000000 data, contig UHOR_scaffold_5.00019, whole genome shotgun sequence	fungi'
JH790073.1 Dictyostelium citrinum unplaced genomic scaffold scaffold00008, whole genome shotgun sequence	protozoa'
JH792473.1 Mycosphaerella populicola P02.02b unplaced genomic scaffold sPop_v1_scaffold00001, whole genome shotgun sequence	fungi'
JH793700.1 Magnaporthe oryzae Y34 unplaced genomic scaffold Y34_scaffold00666, whole genome shotgun sequence	fungi'
JH795166.1 Magnaporthe oryzae P131 unplaced genomic scaffold P131_scaffold00314, whole genome shotgun sequence	fungi'
JH795855.1 Dacryopinax primogenitus strain DJM-731 SS1 chromosome Unknown DACRYscaffold_1, whole genome shotgun sequence	fungi'
JH795954.1 Botryotinia fuckeliana T4 unplaced genomic scaffold BcT4_SuperContig_O_V3__V4, whole genome shotgun sequence	fungi'
CM001575.1 Saccharomyces arboricola H-6 chromosome XIII, whole genome shotgun sequence	fungi'
CM001561.1 Pseudomonas fluorescens R124 chromosome, whole genome shotgun sequence	bacteria'
CM001809.1 Saccharomyces cerevisiae W303 chromosome IV, whole genome shotgun sequence	fungi'
JH802061.1 Ochotona princeps unplaced genomic scaffold scaffold00001, whole genome shotgun sequence	vertebrates_mammals'
JH977583.1 Trichosporon asahii var. asahii CBS 2479 unplaced genomic scaffold scaffold00050, whole genome shotgun sequence	fungi'
JH814812.1 Omphalotus olearius VT 653.13 unplaced genomic scaffold scaffold_1, whole genome shotgun sequence	fungi'
JH823257.1 Chrysochloris asiatica unplaced genomic scaffold scaffold00001, whole genome shotgun sequence	vertebrates_mammals'
JH835289.1 Erinaceus europaeus unplaced genomic scaffold scaffold00001, whole genome shotgun sequence	vertebrates_mammals'
JH817099.1 Crassostrea gigas unplaced genomic scaffold scaffold22, whole genome shotgun sequence	invertebrates'
JH863719.1 Orycteropus afer afer unplaced genomic scaffold scaffold00001, whole genome shotgun sequence	vertebrates_mammals'
JH859027.1 Drosophila albomicans strain KM55-5 unplaced genomic scaffold Dalb_scaffold_62607, whole genome shotgun sequence	invertebrates'
JH881270.1 Bos grunniens mutus unplaced genomic scaffold scaffold644_1, whole genome shotgun sequence	vertebrates_mammals'
CM001580.1 Pasteurella multocida subsp. gallicida X73 chromosome, whole genome shotgun sequence	bacteria'
CM001581.1 Pasteurella multocida subsp. gallicida P1059 chromosome, whole genome shotgun sequence	bacteria'
CM001582.2 Ovis aries breed Texel chromosome 1, whole genome shotgun sequence	vertebrates_mammals'

JH878907.1 Ascaris suum unplaced genomic scaffold Scaffold122, whole genome shotgun sequence	invertebrates'
JH921428.1 Marssonina brunnea f. sp. 'multigermtubi' MB_m1 unplaced genomic scaffold M6_S00001, whole genome shotgun sequence	fungi'
JH947130.1 Elephantulus edwardii unplaced genomic scaffold scaffold00001, whole genome shotgun sequence	vertebrates_mammals'
KQ039398.1 Kluyveromyces marxianus var. marxianus KCTC 17555 unplaced genomic scaffold KM1_scaffold1, whole genome shotgun sequence	fungi'
JH925096.1 Trichosporon asahii var. asahii CBS 8904 unplaced genomic scaffold scaffold00190, whole genome shotgun sequence	fungi'
JH971385.1 Agaricus bisporus var. burnettii JB137-S8 unplaced genomic scaffold AGABI1scaffold_1, whole genome shotgun sequence	fungi'
CP015457.1 Agaricus bisporus var. bisporus H97 chromosome 1 sequence	fungi'
JH930468.1 Phanerochaete carnosa HHB-10118-sp unplaced genomic scaffold PHACAscaffold_1, whole genome shotgun sequence	fungi'
KE388890.1 Anopheles stephensi strain Indian Wild Type (Walter Reed) unplaced genomic scaffold scaffold_00001, whole genome shotgun sequence	invertebrates'
JH932235.1 Passalora fulva CBS 131901 unplaced genomic scaffold scf7180000130789, whole genome shotgun sequence	fungi'
JH932306.1 Ajellomyces dermatitidis ATCC 18187 unplaced genomic scaffold supercont1.1, whole genome shotgun sequence	fungi'
CM003198.1 Fusarium pseudograminearum CS3096 chromosome 1, whole genome shotgun sequence	fungi'
CP003064.1 Treponema pallidum subsp. pallidum str. Mexico A, complete genome	bacteria'
HE650821.1 Kazachstania africana CBS 2517 chromosome 1, complete genome	fungi'
HE608151.1 Mycobacterium tuberculosis UT205 complete genome	bacteria'
CM001609.2 Homo sapiens chromosome 1, whole genome shotgun sequence	vertebrates_mammals'
CP003875.1 Actinobacillus suis H91-0380, complete genome	bacteria'
CM001632.1 Arthrospira platensis C1 chromosome, whole genome shotgun sequence	bacteria'
JH977611.1 Eptesicus fuscus unplaced genomic scaffold scaffold00001, whole genome shotgun sequence	vertebrates_mammals'
CM001772.1 Acinetobacter venetianus VE-C3 chromosome, whole genome shotgun sequence	bacteria'
CM001633.1 Oscillatoriales cyanobacterium JSC-12 chromosome, whole genome shotgun sequence	bacteria'
CM001642.1 Brassica rapa chromosome A9, whole genome shotgun sequence	plants'
FN649727.1 Ectocarpus siliculosus strain Ec 32, whole genome shotgun sequence assembly, chromosome LG02	protozoa'
CP003920.1 Candidatus Sulfuricurvum sp. RIFRC-1, complete genome	bacteria'
KB019377.1 Camelus ferus unplaced genomic scaffold scaffold56579, whole genome shotgun sequence	vertebrates_mammals'
HE965032.1 Taphrina deformans PYCC 5710 genomic scaffold, scaffold00001, whole genome shotgun sequence	fungi'
HF534880.1 Cucumis melo genomic scaffold, scaffold00004, whole genome shotgun sequence	plants'
KB007974.1 Acanthamoeba castellanii str. Neff unplaced genomic scaffold scf7180000084599, whole genome shotgun sequence	protozoa'
HE797160.1 Fibroporia radiculosa TFFH 294 genomic supercontigs, scaffold8592, whole genome shotgun sequence	fungi'
CAFZ01000001.1 Piriformospora indica DSM 11827, WGS project CAFZ01000000 data, contig: PIRI_contig_0001, whole genome shotgun sequence	fungi'
DG000001.5 Oryzias latipes DNA, chromosome1, strain: Hd-rR	vertebrates_non_mammals'
CACQ02001347.1 Colletotrichum higginsianum IMI 349063 WGS project CACQ00000000 data, contig cont1.1, whole genome shotgun sequence	fungi'
HE671591.1 Heliconius melpomene melpomene genomic scaffold, scf7180001250781, whole genome shotgun sequence	invertebrates'
HE813982.1 Musa acuminata subsp. malaccensis Doubled-haploid Pahang (DH-Pahang) genomic chromosome, chr8	plants'
JH980293.1 Echinops telfairi isolate 266 unplaced genomic scaffold scaffold00001, whole genome shotgun sequence	vertebrates_mammals'
CM001774.1 Providencia rettgeri Dmel1 chromosome, whole genome shotgun sequence	bacteria'
CM001773.1 Providencia sneebia DSM 19967 chromosome, whole genome shotgun sequence	bacteria'
KB205985.1 Fusarium fujikuroi B14 unplaced genomic scaffold KF1_scaffold29, whole genome shotgun sequence	fungi'
JH994112.1 Pyrus x bretschneideri cultivar Dangshansuli unplaced genomic scaffold scaffold1.0.1, whole genome shotgun sequence	plants'
JH992965.1 Guillardia theta CCMP2712 unplaced genomic scaffold GUITHscaffold_1, whole genome shotgun sequence	protozoa'
JH993675.1 Penicillium digitatum Pd1 unplaced genomic scaffold Scaffold_42, whole genome shotgun sequence	fungi'
JH993736.1 Penicillium digitatum PHI26 unplaced genomic scaffold Scaffold_50, whole genome shotgun sequence	fungi'
HE681719.1 Candida orthopsilosis Co 90-125, chromosome 1 draft sequence	fungi'
FO082046.1 Pichia sorbitophila strain CBS 7064 chromosome N complete sequence	fungi'
HE806316.1 Tetrapisispora blattae CBS 6284 chromosome 1, complete genome	fungi'
JH993832.1 Trachipleistophora hominis chromosome Unknown scaffold00035, whole genome shotgun sequence	fungi'
CP003600.1 Chamaesiphon minutus PCC 6605, complete genome	bacteria'
CM001672.1 Microtus ochrogaster chromosome 1, whole genome shotgun sequence	vertebrates_mammals'
CM001705.1 Citrus sinensis chromosome 5, whole genome shotgun sequence	plants'
CP003642.1 Cylindrospermum stagnale PCC 7417, complete genome	bacteria'
KB209920.1 Ophiostoma novo-ulmi subsp. novo-ulmi H327 unplaced genomic scaffold scaffold00005, whole genome shotgun sequence	fungi'
CM001710.2 Capra hircus breed Yunnan black goat chromosome 1, whole genome shotgun sequence	vertebrates_mammals'
HF545614.1 Chlamydia psittaci 01DC12 complete genome	bacteria'
AMZP02007909.1 Phytophthora lateralis MPF4 scf_22317_100921.contig_1, whole genome shotgun sequence	protozoa'
HE601810.1 Chlamydia trachomatis A/5291 complete genome	bacteria'
HE601798.1 Chlamydia trachomatis D/SotonD1 high quality draft genome sequence	bacteria'
HE601799.1 Chlamydia trachomatis D/SotonD5 complete genome	bacteria'
HE601800.1 Chlamydia trachomatis D/SotonD6 high quality draft genome sequence	bacteria'
HE601802.1 Chlamydia trachomatis E/SotonE4 complte genome	bacteria'
HE601803.1 Chlamydia trachomatis E/SotonE8 high quality draft genome	bacteria'
HE601806.1 Chlamydia trachomatis F/SotonF3 high quality draft genome sequence	bacteria'
HE601807.1 Chlamydia trachomatis G/SotonG1 complete genome	bacteria'
HE601794.1 Chlamydia trachomatis K/SotonK1 complete genome	bacteria'
HE601958.1 Chlamydia trachomatis L2b/LST high quality draft genome sequence	bacteria'
HE601960.1 Chlamydia trachomatis L2b/CV204 high quality draft genome sequence	bacteria'
HE601959.1 Chlamydia trachomatis L2b/Ams1 complete genome	bacteria'
HE601961.1 Chlamydia trachomatis L2b/Ams2 high quality draft genome sequence	bacteria'
HE601962.1 Chlamydia trachomatis L2b/Ams3 high quality draft genome sequence	bacteria'
HE601964.1 Chlamydia trachomatis L2b/Ams4 high quality draft genome sequence	bacteria'
HE601965.1 Chlamydia trachomatis L2b/Ams5 high quality draft genome sequence	bacteria'
HE601963.1 Chlamydia trachomatis L2b/Canada1 high quality draft genome sequence	bacteria'
HE601957.1 Chlamydia trachomatis L2b/Canada2 high quality draft genome sequence	bacteria'
HE601951.1 Chlamydia trachomatis L1/1322/p2 high quality draft genome	bacteria'
HE793032.1 Brachyspira pilosicoli WesB complete genome	bacteria'
HE984298.2 Helicobacter heilmannii ASB1.4 main chromosome, draft genome	bacteria'
KB020503.1 Colletotrichum gloeosporioides Nara gc5 unplaced genomic scaffold scaffold15, whole genome shotgun sequence	fungi'
KB456260.1 Sphaerulina musiva SO2202 unplaced genomic scaffold SEPMUscaffold_1, whole genome shotgun sequence	fungi'
KI925454.1 Heterobasidion irregulare TC 32-1 unplaced genomic scaffold scaffold_01, whole genome shotgun sequence	fungi'
KB445791.1 Ceriporiopsis subvermispora B unplaced genomic scaffold CERSUscaffold_1, whole genome shotgun sequence	fungi'
KQ234361.1 Plasmodium vivax India VII chromosome Unknown supercont1.221, whole genome shotgun sequence	protozoa'
KQ234858.1 Plasmodium vivax Brazil I chromosome Unknown supercont1.150, whole genome shotgun sequence	protozoa'
KQ235088.1 Plasmodium vivax Mauritania I chromosome Unknown supercont1.120, whole genome shotgun sequence	protozoa'
KQ235548.1 Plasmodium vivax North Korean chromosome Unknown supercont1.375, whole genome shotgun sequence	protozoa'
KB722642.1 Rhodosporidium toruloides NP11 unplaced genomic scaffold Scaffold1, whole genome shotgun sequence	fungi'
KB228879.1 Odobenus rosmarus divergens isolate Ivan unplaced genomic scaffold Scaffold2, whole genome shotgun sequence	vertebrates_mammals'
DF157104.1 Plasmodium cynomolgi strain B DNA, chromosome 12, whole genome shotgun sequence	protozoa'
DG000053.1 Oryza sativa Japonica Group DNA, complete genome, chromosome 1, cultivar: Hitomebore, whole genome shotgun sequence	plants'
KN003119.1 Toxoplasma gondii GAB2-2007-GAL-DOM2 unplaced genomic scaffold scaffold00002, whole genome shotgun sequence	protozoa'
KB031068.1 Pteropus alecto unplaced genomic scaffold scaffold3, whole genome shotgun sequence	vertebrates_mammals'
KB445548.1 Oryza granulata chromosome 3 short arm genomic scaffold Ogran_chr3s, whole genome shotgun sequence	plants'
CM002476.1 Leersia perrieri chromosome 1, whole genome shotgun sequence	plants'
AFYB01000004.1 Solanum lycopersicum cultivar Heinz 1706 chromosome SlmtSC_A Scaffold_001_4 mitochondrial, whole genome shotgun sequence	plants'
CM001783.1 Eutrema salsugineum chromosome 6, whole genome shotgun sequence	plants'
KQ004500.1 Sus scrofa isolate L1-53 breed Wuzhishan unplaced genomic scaffold scaffold93, whole genome shotgun sequence	vertebrates_mammals'
ADVV01000082.1 Saccharomyces cerevisiae Lalvin QA23 chromosome XVI QA23_c16_82, whole genome shotgun sequence	fungi'
DF159777.1 Latimeria chalumnae DNA, scaffold: scaffold3038, isolate: TCC041-004, whole genome shotgun sequence	vertebrates_non_mammals'
CAJW011558480.1 Hordeum vulgare subsp. vulgare WGS project CAJW00000000 data, cultivar Morex, contig morex_contig_1558480, whole genome shotgun sequence	plants'
CAJX010058213.1 Hordeum vulgare subsp. vulgare WGS project CAJX00000000 data, cultivar Bowman, contig bowman_contig_62047, whole genome shotgun sequence	plants'
KB095811.1 Helobdella robusta unplaced genomic scaffold HELROscaffold_1, whole genome shotgun sequence	invertebrates'
CAOK01002904.1 Betula nana WGS project CAOK00000000 data, contig 2904, whole genome shotgun sequence	plants'
KB199562.1 Myotis davidii unplaced genomic scaffold scaffold6, whole genome shotgun sequence	vertebrates_mammals'
CM001748.1 Gossypium raimondii chromosome 9, whole genome shotgun sequence	plants'
KB199650.1 Lottia gigantea unplaced genomic scaffold LOTGIsca_1, whole genome shotgun sequence	invertebrates'
KB205139.1 Trypanosoma cruzi strain Esmeraldo unplaced genomic scaffold Scaffold1, whole genome shotgun sequence	protozoa'
KB291798.1 Capitella teleta unplaced genomic scaffold CAPTEscaffold_1, whole genome shotgun sequence	invertebrates'
DG000065.1 Cyberlindnera jadinii NBRC 0988 DNA, chromosome: 1, whole genome shotgun sequence	fungi'
CM003140.1 Ustilago maydis 521 chromosome 1, whole genome shotgun sequence	fungi'
FO203509.1 Mycobacterium canettii CIPT 140070010 complete genome	bacteria'
FO203507.1 Mycobacterium canettii CIPT 140060008 complete genome	bacteria'
FO203510.1 Mycobacterium canettii CIPT 140070017 complete genome	bacteria'
FO203508.1 Mycobacterium canettii CIPT 140070008 complete genome	bacteria'
KB205939.1 Ascocoryne sarcoides NRRL 50072 unplaced genomic scaffold scaffold00001, whole genome shotgun sequence	fungi'
KB206292.1 Entamoeba invadens IP1 unplaced genomic scaffold scaff_1105074726711, whole genome shotgun sequence	protozoa'
KB207269.1 Plutella xylostella unplaced genomic scaffold scaffold_16, whole genome shotgun sequence	invertebrates'
FO203522.1 Desulfovibrio hydrothermalis AM13 = DSM 14728 chromosome, complete genome	bacteria'
KB208804.1 Gossypium raimondii cultivar CMD10 unplaced genomic scaffold scaffold13, whole genome shotgun sequence	plants'
HE796683.1 Fibrella aestuarina BUZ 2 drat genome	bacteria'
CM001769.1 Cicer arietinum chromosome Ca6, whole genome shotgun sequence	plants'
CM001762.1 Mycobacterium smegmatis MKD8 chromosome, whole genome shotgun sequence	bacteria'
CM003407.1 Crithidia fasciculata strain Cf-Cl chromosome 30, whole genome shotgun sequence	protozoa'
KB217888.1 Leishmania major strain LV39c5 unplaced genomic scaffold Scaffold1, whole genome shotgun sequence	protozoa'
KQ490441.1 Ensete ventricosum unplaced genomic scaffold scf_3064_1461, whole genome shotgun sequence	plants'
CM001763.1 Pseudomonas syringae pv. syringae B64 chromosome, whole genome shotgun sequence	bacteria'
KB222877.1 Trypanosoma cruzi JR cl. 4 unplaced genomic scaffold Scaffold1, whole genome shotgun sequence	protozoa'
KB221188.1 Pseudopodoces humilis unplaced genomic scaffold scaffold4, whole genome shotgun sequence	vertebrates_non_mammals'
KQ479111.1 Trichoderma hamatum GD12 unplaced genomic scaffold scf_32592_1, whole genome shotgun sequence	fungi'
KB233244.1 Ascaris suum unplaced genomic scaffold AS00001, whole genome shotgun sequence	invertebrates'
KB316842.1 Orcinus orca unplaced genomic scaffold Scaffold1, whole genome shotgun sequence	vertebrates_mammals'
CM001775.1 Geminocystis herdmanii PCC 6308 chromosome, whole genome shotgun sequence	bacteria'
CM001776.1 Synechococcus sp. PCC 7336 chromosome, whole genome shotgun sequence	bacteria'
KB285691.1 Amazona vittata unplaced genomic scaffold scaffold-66279, whole genome shotgun sequence	vertebrates_non_mammals'
KB290581.1 Trichoderma longibrachiatum SMF2 unplaced genomic scaffold scaffold001, whole genome shotgun sequence	fungi'
AOFH02000001.1 Phytophthora lateralis strain SMSTG scf_44827_1.contig_1, whole genome shotgun sequence	protozoa'
AOFI02000001.1 Phytophthora kernoviae 00238/432 scf_16495_1.contig_1, whole genome shotgun sequence	protozoa'
AOFJ02000001.1 Phytophthora kernoviae 00629/1 scf_16591_1.contig_1, whole genome shotgun sequence	protozoa'
AOFK02000001.1 Phytophthora kernoviae 00844/4 scf_16689_1.contig_1, whole genome shotgun sequence	protozoa'
CM007320.1 Endotrypanum monterogeii strain LV88 chromosome 36, whole genome shotgun sequence	protozoa'
ANAF02000182.1 Leptomonas seymouri BHU1095 Scaffold_182, whole genome shotgun sequence	protozoa'
KB317696.1 Rhizoctonia solani AG-1 IA unplaced genomic scaffold scaffold1, whole genome shotgun sequence	fungi'
DF196775.1 Pseudozyma antarctica T-34 DNA, contig: scaffold00009, whole genome shotgun sequence	fungi'
KB320563.1 Tupaia chinensis unplaced genomic scaffold Scaffold000146_1, whole genome shotgun sequence	vertebrates_mammals'
AOBL02000001.1 Phytophthora ramorum strain EU2_996_3 scf_16392_1.contig_1, whole genome shotgun sequence	protozoa'
KB375528.1 Columba livia unplaced genomic scaffold scaffold102, whole genome shotgun sequence	vertebrates_non_mammals'
KB390952.1 Falco peregrinus unplaced genomic scaffold scaffold26_1, whole genome shotgun sequence	vertebrates_non_mammals'
KB397780.1 Falco cherrug unplaced genomic scaffold scaffold45_1, whole genome shotgun sequence	vertebrates_non_mammals'

CM001785.1 Vibrio cholerae O1 str. Inaba G4222 chromosome I, whole genome shotgun sequence	bacteria'
KB423929.1 Lupinus angustifolius unplaced genomic scaffold scaffold45357, whole genome shotgun sequence	plants'
CM001787.1 Bacillus cereus F chromosome, whole genome shotgun sequence	bacteria'
ANHN02000026.1 Phytophthora lateralis MPF6 scf_18899_23.contig_1, whole genome shotgun sequence	protozoa'
KQ479425.1 Phytophthora lateralis SMST21 unplaced genomic scaffold scf_4885_6, whole genome shotgun sequence	protozoa'
KB444497.1 Entamoeba histolytica KU27 unplaced genomic scaffold scaffold00001, whole genome shotgun sequence	protozoa'
CM002524.1 Oryza meridionalis cultivar OR44 (W2112) chromosome 1, whole genome shotgun sequence	plants'
KB445550.1 Baudoinia panamericana UAMH 10762 unplaced genomic scaffold BAUCOscaffold_1, whole genome shotgun sequence	fungi'
KB445569.1 Cochliobolus heterostrophus C5 unplaced genomic scaffold COCHEscaffold_1, whole genome shotgun sequence	fungi'
KB445637.1 Cochliobolus sativus ND90Pr unplaced genomic scaffold COCSAscaffold_1, whole genome shotgun sequence	fungi'
KB446535.1 Dothistroma septosporum NZE10 unplaced genomic scaffold DOTSEscaffold_1, whole genome shotgun sequence	fungi'
KB446555.1 Pseudocercospora fijiensis CIRAD86 unplaced genomic scaffold MYCFIscaffold_1, whole genome shotgun sequence	fungi'
CM007313.1 Leishmania braziliensis MHOM/BR/75/M2903 chromosome 35, whole genome shotgun sequence	protozoa'
KV887145.1 Phytophthora ramorum strain EU1 2275 unplaced genomic scaffold scf_54375_1, whole genome shotgun sequence	protozoa'
CM001792.1 Oceanobacillus kimchii X50 chromosome, whole genome shotgun sequence	bacteria'
KB453278.1 Leishmania panamensis MHOM/COL/81/L13 unplaced genomic scaffold Scaffold1, whole genome shotgun sequence	protozoa'
KB616792.1 Hevea brasiliensis unplaced genomic scaffold scaffold142103, whole genome shotgun sequence	plants'
CP007203.2 Nodularia spumigena CCY9414 genome	bacteria'
CM001794.1 Treponema denticola H1-T chromosome, whole genome shotgun sequence	bacteria'
CM001795.1 Treponema denticola H-22 chromosome, whole genome shotgun sequence	bacteria'
CM003613.1 Cajanus cajan chromosome 11, whole genome shotgun sequence	plants'
CM001796.1 Treponema denticola ATCC 35404 chromosome, whole genome shotgun sequence	bacteria'
CM001797.1 Treponema denticola OTK chromosome, whole genome shotgun sequence	bacteria'
CM001798.1 Treponema denticola AL-2 chromosome, whole genome shotgun sequence	bacteria'
KB454452.1 Dekkera bruxellensis CBS 2499 unplaced genomic scaffold DEKBRscaffold_1, whole genome shotgun sequence	fungi'
KB454484.1 Galdieria sulphuraria unplaced genomic scaffold scaf_0, whole genome shotgun sequence	plants'
KB455460.1 Panagrellus redivivus unplaced genomic scaffold scaffold823, whole genome shotgun sequence	invertebrates'
FO203427.1 Desulfovibrio piezophilus C1TLV30 complete genome	bacteria'
HF689366.1 Megaselia scalaris Durham, NC isolate 2 -- Noor lab genomic scaffold, scaffold9042, whole genome shotgun sequence	invertebrates'
KB465683.1 Cephus cinctus unplaced genomic scaffold Ccin1_scaffold0168, whole genome shotgun sequence	invertebrates'
CM001804.1 Bacillus thuringiensis DAR 81934 chromosome chromosome, whole genome shotgun sequence	bacteria'
CM001799.1 Vibrio vulnificus VVyb1(BT3) chromosome I, whole genome shotgun sequence	bacteria'
CP001669.1 Babesia equi strain WA chromosome 1, complete sequence	protozoa'
KB467406.1 Athalia rosae unplaced genomic scaffold Scaffold10, whole genome shotgun sequence	invertebrates'
CM001824.1 Stenotrophomonas maltophilia EPM1 chromosome, whole genome shotgun sequence	bacteria'
KB528689.1 Chelonia mydas unplaced genomic scaffold scaffold27, whole genome shotgun sequence	vertebrates_non_mammals'
KB467831.1 Wolfiporia cocos MD-104 SS10 unplaced genomic scaffold WOLCOscaffold_1, whole genome shotgun sequence	fungi'
KE504123.1 Fomitopsis pinicola FP-58527 SS1 unplaced genomic scaffold FOMPIscaffold_2, whole genome shotgun sequence	fungi'
KB469296.1 Gloeophyllum trabeum ATCC 11539 unplaced genomic scaffold GLOTRscaffold_00001, whole genome shotgun sequence	fungi'
KB610562.1 Entamoeba histolytica HM-1:IMSS-B unplaced genomic scaffold scaffold00001, whole genome shotgun sequence	protozoa'
KB632399.1 Dendroctonus ponderosae unplaced genomic scaffold Seq_985524, whole genome shotgun sequence	invertebrates'
APGM01000001.1 Klebsiella pneumoniae RYC492 contig0001, whole genome shotgun sequence	bacteria'
KB637218.1 Entamoeba histolytica HM-3:IMSS unplaced genomic scaffold scaffold00001, whole genome shotgun sequence	protozoa'
CM007651.1 Prunus persica cultivar Lovell chromosome G1, whole genome shotgun sequence	plants'
KB640820.1 Lasioglossum albipes unplaced genomic scaffold scaffold53, whole genome shotgun sequence	invertebrates'
CM001827.1 Prunus mume isolate 4 linkage group 2, whole genome shotgun sequence	plants'
KB644415.1 Penicillium oxalicum 114-2 unplaced genomic scaffold scaffold_8, whole genome shotgun sequence	fungi'
CM003631.1 Cicer arietinum cultivar ICC4958 chromosome Ca4, whole genome shotgun sequence	plants'
KD503936.1 Aegilops tauschii cultivar AL8/78 unplaced genomic scaffold Scaffold4725, whole genome shotgun sequence	plants'
CAGA01000001.1 Claviceps purpurea 20.1 WGS project CAGA00000000 data, contig scaffold00001, whole genome shotgun sequence	fungi'
KD050940.1 Triticum urartu cultivar G1812 unplaced genomic scaffold scaffold9858, whole genome shotgun sequence	plants'
KB644853.1 Penicillium paxilli ATCC 26601 unplaced genomic scaffold scaffold306, whole genome shotgun sequence	fungi'
KB660352.1 Ceratitis capitata unplaced genomic scaffold Scaffold3, whole genome shotgun sequence	invertebrates'
CP004012.1 Ralstonia solanacearum FQY_4, complete genome	bacteria'
CM001835.1 Streptococcus pneumoniae PCS8235 chromosome, whole genome shotgun sequence	bacteria'
HF562300.1 Chlamydia trachomatis IU888 complete genome	bacteria'
HF562298.1 Chlamydia trachomatis IU824 complete genome	bacteria'
HE978315.1 Kazachstania naganishii CBS 8797 chromosome 2, complete genome	fungi'
KB663610.1 Anopheles minimus strain MINIMUS1 unplaced genomic scaffold supercont1.1, whole genome shotgun sequence	invertebrates'
KB664288.1 Anopheles stephensi strain SDA-500 unplaced genomic scaffold supercont1.1, whole genome shotgun sequence	invertebrates'
KB666509.1 Anopheles quadriannulatus strain QUAD4_A unplaced genomic scaffold supercont1.2, whole genome shotgun sequence	invertebrates'
KB668221.1 Anopheles funestus strain FUMOZ unplaced genomic scaffold supercont1.1, whole genome shotgun sequence	invertebrates'
KB669613.1 Anopheles epiroticus strain epiroticus2 unplaced genomic scaffold supercont1.1, whole genome shotgun sequence	invertebrates'
KB672286.1 Anopheles albimanus strain ALBI9_A unplaced genomic scaffold supercont1.1, whole genome shotgun sequence	invertebrates'
KB672490.1 Anopheles dirus strain WRAIR2 unplaced genomic scaffold supercont1.1, whole genome shotgun sequence	invertebrates'
KB673756.1 Anopheles christyi strain ACHKN1017 unplaced genomic scaffold supercont1.1, whole genome shotgun sequence	invertebrates'
KB704125.1 Anopheles arabiensis strain DONG5_A unplaced genomic scaffold supercont1.1, whole genome shotgun sequence	invertebrates'
HE999557.1 Malassezia sympodialis ATCC 42132 genomic scaffold, msy_sf_9, whole genome shotgun sequence	fungi'
HE971709.1 Streptomyces davawensis strain JCM 4913 complete genome	bacteria'
KB707241.1 Eutypa lata UCREL1 unplaced genomic scaffold EL1_03_scaffold_1903, whole genome shotgun sequence	fungi'
KB707785.1 Botryotinia fuckeliana BcDW1 unplaced genomic scaffold Scaffold_113, whole genome shotgun sequence	fungi'
KB708127.1 Mesocricetus auratus isolate Golden Hamster female 1 unplaced genomic scaffold scaffold00001, whole genome shotgun sequence	vertebrates_mammals'
KB714707.1 Leptonychotes weddellii isolate WS11-02 unplaced genomic scaffold scaffold00001, whole genome shotgun sequence	vertebrates_mammals'
KB722736.1 Volvariella volvacea V23 unplaced genomic scaffold VVO_S00001, whole genome shotgun sequence	fungi'
KB725665.1 Colletotrichum orbiculare MAFF 240422 unplaced genomic scaffold Scaffold_127, whole genome shotgun sequence	fungi'
HG001459.1 Chondrus crispus Stackhouse genomic scaffold, scaffold_1, whole genome shotgun sequence	plants'
KB731261.1 Fusarium oxysporum f. sp. cubense race 1 unplaced genomic scaffold scaffold80, whole genome shotgun sequence	fungi'
KB726998.1 Fusarium oxysporum f. sp. cubense race 4 unplaced genomic scaffold scaffold22, whole genome shotgun sequence	fungi'
KB726999.1 Mus musculus strain 129Sv/Jae isolate 1 unplaced genomic scaffold scaffold00001, whole genome shotgun sequence	vertebrates_mammals'
KB733444.1 Bipolaris maydis ATCC 48331 unplaced genomic scaffold COCC4scaffold_1, whole genome shotgun sequence	fungi'
KB741277.1 Dendroctonus ponderosae unplaced genomic scaffold Seq_1103023, whole genome shotgun sequence	invertebrates'
CM001837.1 Dokdonia sp. PRO95 chromosome, whole genome shotgun sequence	bacteria'
KB742833.1 Anas platyrhynchos breed Pekin duck unplaced genomic scaffold scaffold376, whole genome shotgun sequence	vertebrates_non_mammals'
AOUM01000001.1 Pyrenochaeta sp. UM 256 sctg_0001_0001, whole genome shotgun sequence	fungi'
KB908481.1 Setosphaeria turcica Et28A unplaced genomic scaffold SETTUscaffold_1, whole genome shotgun sequence	fungi'
CM001919.1 Macaca fascicularis chromosome 1, whole genome shotgun sequence	vertebrates_mammals'
KB822804.1 Saccharomyces cerevisiae M3707 unplaced genomic scaffold C395scaffold_1, whole genome shotgun sequence	fungi'
KB822858.1 Saccharomyces cerevisiae M3836 unplaced genomic scaffold C396scaffold_1, whole genome shotgun sequence	fungi'
KB822910.1 Saccharomyces cerevisiae M3837 unplaced genomic scaffold C397scaffold_1, whole genome shotgun sequence	fungi'
KB822770.1 Saccharomyces cerevisiae M3838 unplaced genomic scaffold C398scaffold_1, whole genome shotgun sequence	fungi'
KB822726.1 Saccharomyces cerevisiae M3839 unplaced genomic scaffold C399scaffold_1, whole genome shotgun sequence	fungi'
KB822711.1 Cyphellophora europaea CBS 101466 unplaced genomic scaffold supercont1.1, whole genome shotgun sequence	fungi'
KB822697.1 Cladophialophora carrionii CBS 160.54 unplaced genomic scaffold supercont1.1, whole genome shotgun sequence	fungi'
KB846784.1 Nelumbo nucifera unplaced genomic scaffold scaffold00001, whole genome shotgun sequence	plants'
KB851304.1 Trypanosoma cruzi Tula cl2 unplaced genomic scaffold Scaffold1, whole genome shotgun sequence	protozoa'
CM001839.1 Dickeya solani MK10 chromosome, whole genome shotgun sequence	bacteria'
CM001841.1 Dickeya dianthicola NCPPB 453 chromosome, whole genome shotgun sequence	bacteria'
CM001842.1 Dickeya solani MK16 chromosome, whole genome shotgun sequence	bacteria'
CM001838.1 Dickeya dianthicola GBBC 2039 chromosome, whole genome shotgun sequence	bacteria'
CM001840.1 Dickeya dianthicola NCPPB 3534 chromosome, whole genome shotgun sequence	bacteria'
CH003497.1 Homo sapiens chromosome 2, whole genome shotgun sequence	vertebrates_mammals'
KB838940.1 Parasteatoda tepidariorum isolate Goettingen unplaced genomic scaffold Scaffold3, whole genome shotgun sequence	invertebrates'
KB824662.1 Entamoeba histolytica HM-1:IMSS-A unplaced genomic scaffold scaffold00001, whole genome shotgun sequence	protozoa'
HF937103.1 Staphylococcus aureus M1 complete genome	bacteria'
KB854187.1 Musca domestica strain aabys unplaced genomic scaffold Scaffold0, whole genome shotgun sequence	invertebrates'
CP012035.1 Acinetobacter baumannii strain PR07 genome	bacteria'
KB871578.1 Astyanax mexicanus unplaced genomic scaffold Scaffold0, whole genome shotgun sequence	vertebrates_non_mammals'
KB853656.1 Cerataphis brasiliensis yeast-like symbiont unplaced genomic scaffold YLSCB_531356, whole genome shotgun sequence	fungi'
KB863010.1 Emiliania huxleyi CCMP1516 unplaced genomic scaffold EMIHUscaffold_1, whole genome shotgun sequence	protozoa'
KB870805.1 Capsella rubella cultivar Monte Gargano unplaced genomic scaffold scaffold_1, whole genome shotgun sequence	plants'
CP005385.1 Meiothermus ruber DSM 1279 genome	bacteria'
KK214815.1 Magnaporthe oryzae KJ201 unplaced genomic scaffold Scaffold_2, whole genome shotgun sequence	fungi'
KB886750.1 Ladona fulva unplaced genomic scaffold Scaffold5, whole genome shotgun sequence	invertebrates'
CM001843.1 Porphyromonas gingivalis JCVI SC001 chromosome, whole genome shotgun sequence	bacteria'
ANIS01001780.1 Leucoagaricus gongylophorus Ac12 contig01780, whole genome shotgun sequence	fungi'
KB908904.1 Penicillium decumbens JU-A10-T unplaced genomic scaffold scaffold_17, whole genome shotgun sequence	fungi'
KB908914.1 Nosema bombycis CQ1 unplaced genomic scaffold NBO_6, whole genome shotgun sequence	fungi'
CM001844.1 Bartonella bovis 91-4 chromosome, whole genome shotgun sequence	bacteria'
KB913038.1 Zonotrichia albicollis isolate Tan morph unplaced genomic scaffold Scaffold0, whole genome shotgun sequence	vertebrates_non_mammals'
KB915786.1 Neofusicoccum parvum UCRNP2 chromosome Unknown NP2_03_scaffold_148, whole genome shotgun sequence	fungi'
KB929668.1 Apalone spinifera unplaced genomic scaffold scaffold2762, whole genome shotgun sequence	vertebrates_non_mammals'
KK358017.1 Anncaliia algerae PRA109 unplaced genomic scaffold supercont4.1, whole genome shotgun sequence	fungi'
KK365130.1 Anncaliia algerae PRA339 unplaced genomic scaffold supercont2.1, whole genome shotgun sequence	fungi'
CP011802.1 Bacillus atrophaeus UCMB-5137 genome	bacteria'
KE476611.1 Pythium irregulare DAOM BR486 unplaced genomic scaffold pir_scaffold_1, whole genome shotgun sequence	protozoa'
KE463865.1 Pythium aphanidermatum DAOM BR444 unplaced genomic scaffold pag1_scaffold_1, whole genome shotgun sequence	protozoa'
KE482498.1 Pythium iwayamai DAOM BR242034 unplaced genomic scaffold piw_scaffold_1, whole genome shotgun sequence	protozoa'
CP003902.1 Treponema pallidum str. Fribourg-Blanc, complete genome	bacteria'
KE465639.1 Pythium arrhenomanes ATCC 12531 unplaced genomic scaffold par_scaffold_1, whole genome shotgun sequence	protozoa'
KE494039.1 Pythium ultimum var. sporangiiferum BR650 strain DAOM BR650 unplaced genomic scaffold pug3_scaffold_1, whole genome shotgun sequence	protozoa'
KE499521.1 Pythium vexans DAOM BR484 unplaced genomic scaffold pve_scaffold_1, whole genome shotgun sequence	protozoa'
KB932201.1 Fonticula alba strain ATCC 38817 unplaced genomic scaffold supercont2.1, whole genome shotgun sequence	invertebrates'
CM001848.2 Lactobacillus casei LcY chromosome, whole genome shotgun sequence	bacteria'
BAAG010040742.1 Mus musculus DNA, chromosome 1, contig: ctg1_40742, strain: MSM/Ms, whole genome shotgun sequence	vertebrates_mammals'
CM001852.1 Nocardioides sp. CF8 chromosome, whole genome shotgun sequence	bacteria'
KB933500.1 Anoplophora glabripennis unplaced genomic scaffold Scaffold1, whole genome shotgun sequence	invertebrates'
KB964490.1 Nicotiana tomentosiformis unplaced genomic scaffold Ntom_scaffold4015, whole genome shotgun sequence	plants'
KB933264.1 Togninia minima UCRPA7 unplaced genomic scaffold PA7_03_scaffold_496, whole genome shotgun sequence	fungi'
KD962201.1 Nicotiana sylvestris unplaced genomic scaffold Nsyl_scaffold4564, whole genome shotgun sequence	plants'
ARYA01024894.1 Beta vulgaris subsp. vulgaris cultivar KDH13 Contig-4705942, whole genome shotgun sequence	plants'
KE007224.1 Wallemia ichthyophaga EXF-994 unplaced genomic scaffold scaffold1, whole genome shotgun sequence	fungi'
CM001857.1 Dickeya paradisiaca NCPPB 2511 chromosome, whole genome shotgun sequence	bacteria'
CM001858.1 Dickeya zeae NCPPB 3532 chromosome, whole genome shotgun sequence	bacteria'
CM001860.1 Dickeya solani GBBC 2040 chromosome, whole genome shotgun sequence	bacteria'
CM001861.1 Lactobacillus casei LcA chromosome, whole genome shotgun sequence	bacteria'

KE029017.1 Ara macao unplaced genomic scaffold s_1_1NewScaffoldedcontig_10990, whole genome shotgun sequence	vertebrates_non_mammals'
KE123234.1 Takifugu flavidus unplaced genomic scaffold SCAFFOLD_3202, whole genome shotgun sequence	vertebrates_non_mammals'
CM001859.1 Dickeya solani IPO 2222 chromosome, whole genome shotgun sequence	bacteria'
CP009075.1 Verticillium dahliae JR2 chromosome 1, complete sequence	fungi'
KE113671.1 Pantholops hodgsonii unplaced genomic scaffold Scaffold21, whole genome shotgun sequence	vertebrates_mammals'
KE123896.1 Mucor circinelloides f. circinelloides 1006PhL unplaced genomic scaffold supercont1.1, whole genome shotgun sequence	fungi'
KE123882.1 Plasmodium falciparum NF54 unplaced genomic scaffold supercont1.338, whole genome shotgun sequence	protozoa'
KE124552.1 Plasmodium falciparum UGT5.1 unplaced genomic scaffold supercont1.242, whole genome shotgun sequence	protozoa'
CM001871.1 Xanthomonas campestris pv. campestris str. CN14 chromosome, whole genome shotgun sequence	bacteria'
KE124775.1 Ancylostoma ceylanicum unplaced genomic scaffold A_ceylanicum-1.0_Cont1, whole genome shotgun sequence	invertebrates'
DF238808.1 Pseudozyma hubeiensis SY62 DNA, scaffold: PHS45, whole genome shotgun sequence	fungi'
CM001880.1 Theobroma cacao cultivar Matina 1-6 chromosome 2, whole genome shotgun sequence	plants'
CM001897.1 Xanthomonas campestris pv. campestris str. CN16 chromosome, whole genome shotgun sequence	bacteria'
CM001890.1 Xanthomonas campestris pv. campestris str. CN15 chromosome, whole genome shotgun sequence	bacteria'
CM001889.1 Streptomyces lividans 1326 chromosome, whole genome shotgun sequence	bacteria'
APFE031145623.1 Pinus taeda scaffold30106, whole genome shotgun sequence	plants'
CM001972.1 Dickeya zeae CSL RW192 chromosome, whole genome shotgun sequence	bacteria'
CM001904.1 Dickeya chrysanthemi NCPPB 516 chromosome, whole genome shotgun sequence	bacteria'
CM001973.2 Dickeya sp. CSL RW240 chromosome, whole genome shotgun sequence	bacteria'
CM001974.1 Dickeya chrysanthemi NCPPB 402 chromosome, whole genome shotgun sequence	bacteria'
CM001975.1 Dickeya sp. NCPPB 569 chromosome, whole genome shotgun sequence	bacteria'
CM001976.1 Dickeya dadantii NCPPB 898 chromosome, whole genome shotgun sequence	bacteria'
CM001977.1 Dickeya zeae NCPPB 2538 chromosome, whole genome shotgun sequence	bacteria'
CM001978.1 Dickeya dadantii subsp. dadantii NCPPB 2976 chromosome, whole genome shotgun sequence	bacteria'
CM001979.1 Dickeya sp. NCPPB 3274 chromosome, whole genome shotgun sequence	bacteria'
CM001980.1 Dickeya zeae NCPPB 3531 chromosome, whole genome shotgun sequence	bacteria'
CM001981.1 Dickeya chrysanthemi NCPPB 3533 chromosome, whole genome shotgun sequence	bacteria'
CM001982.1 Dickeya dadantii NCPPB 3537 chromosome, whole genome shotgun sequence	bacteria'
CM001983.1 Dickeya sp. DW 0440 chromosome, whole genome shotgun sequence	bacteria'
CM001984.1 Dickeya sp. MK7 chromosome, whole genome shotgun sequence	bacteria'
CM001985.1 Dickeya zeae MK19 chromosome, whole genome shotgun sequence	bacteria'
CM004347.1 Leishmania sp. MAR LEM2494 chromosome 36, whole genome shotgun sequence	protozoa'
KE145367.1 Glarea lozoyensis ATCC 20868 chromosome Unknown GLAREA3, whole genome shotgun sequence	fungi'
CM001948.2 Chlorocebus sabaeus isolate 1994-021 chromosome 8, whole genome shotgun sequence	vertebrates_mammals'
CP004010.2 Treponema pallidum subsp. pallidum str. Nichols, complete genome	bacteria'
CP004011.1 Treponema pallidum subsp. pallidum SS14, complete genome	bacteria'
CM004641.1 Leishmania arabica strain LEM1108 chromosome 36, whole genome shotgun sequence	protozoa'
KE147309.1 Leishmania tropica L590 unplaced genomic scaffold Scaffold0, whole genome shotgun sequence	protozoa'
KE148146.1 Ophiostoma piceae UAMH 11346 chromosome Unknown scf01, whole genome shotgun sequence	fungi'
CM007249.1 Leishmania enriettii strain LEM3045 chromosome 36, whole genome shotgun sequence	protozoa'
AIJO01004611.1 Hortaea werneckii EXF-2000 contig65257_0, whole genome shotgun sequence	fungi'
KE158016.1 Leavenworthia alabamica unplaced genomic scaffold LA_scaffold272, whole genome shotgun sequence	plants'
KE156110.1 Sisymbrium irio isolate GC 1146-67 unplaced genomic scaffold SI_scaffold843, whole genome shotgun sequence	plants'
KE151199.1 Aethionema arabicum unplaced genomic scaffold AA_scaffold2718, whole genome shotgun sequence	plants'
KE159915.1 Pochonia chlamydosporia 123 unplaced genomic scaffold scaffold267, whole genome shotgun sequence	fungi'
ALWZ04S2422157.1 Picea glauca Pg-01r141201s2433421, whole genome shotgun sequence	plants'
CM001986.1 Pseudomonas syringae pv. syringae SM chromosome, whole genome shotgun sequence	bacteria'
CP006026.1 Saccharomycetaceae sp. 'Ashbya aceri' chromosome VII, complete sequence	fungi'
KE161240.1 Myotis brandtii unplaced genomic scaffold scaffold115, whole genome shotgun sequence	vertebrates_mammals'
CM002276.1 Pasteurella multocida subsp. multocida P1062 chromosome, whole genome shotgun sequence	bacteria'
KE332545.1 Phoenix dactylifera cultivar Khalas unplaced genomic scaffold pdS000001, whole genome shotgun sequence	plants'
KL558888.1 Physarum polycephalum strain LU352 unplaced genomic scaffold Scaffold0, whole genome shotgun sequence	protozoa'
KE346217.1 Morus notabilis unplaced genomic scaffold Scaffold78, whole genome shotgun sequence	plants'
ASJL01017490.1 Blumeria graminis f. sp. tritici JIW2 contig_25846_1, whole genome shotgun sequence	fungi'
KE362319.1 Blumeria graminis f. sp. tritici 94202 unplaced genomic scaffold scaffold_37481, whole genome shotgun sequence	fungi'
KE361623.1 Pseudozyma flocculosa PF-1 unplaced genomic scaffold scaffold00001, whole genome shotgun sequence	fungi'
CM002022.1 Mycobacterium tuberculosis PanR0202 chromosome, whole genome shotgun sequence	bacteria'
KE370772.1 Zizania latifolia unplaced genomic scaffold scaffold_0, whole genome shotgun sequence	plants'
KE374994.1 Blumeria graminis f. sp. tritici 96224 unplaced genomic scaffold Scaffold-14, whole genome shotgun sequence	fungi'
CP006567.1 Streptomyces rapamycinicus NRRL 5491 genome	bacteria'
KE382941.1 Cricetulus griseus unplaced genomic scaffold scaffold787, whole genome shotgun sequence	vertebrates_mammals'
KE384729.1 Metarhizium anisopliae BRIP 53293 unplaced genomic scaffold Scaffold_12, whole genome shotgun sequence	fungi'
KE385298.1 Metarhizium anisopliae BRIP 53284 unplaced genomic scaffold Scaffold_4, whole genome shotgun sequence	fungi'
HF951689.1 Chthonomonas calidirosea T49 complete genome	bacteria'
FP885895.1 Ralstonia solanacearum CMR15 chromosome, complete genome	bacteria'
CM002023.1 Dickeya dianthicola IPO 980 chromosome, whole genome shotgun sequence	bacteria'
KE391414.1 Leishmania amazonensis unplaced genomic scaffold Scaffold3675, whole genome shotgun sequence	protozoa'
KE392324.1 Rhizophagus irregularis DAOM 181602 strain DAOM 197198 unplaced genomic scaffold GLOINscaffold_523, whole genome shotgun sequence	fungi'
ATLZ01001265.1 Anopheles nili Ctg1265, whole genome shotgun sequence	invertebrates'
CM002024.1 Agrobacterium tumefaciens WRT31 chromosome 1, whole genome shotgun sequence	bacteria'
KE444511.1 Phytophthora alni subsp. alni unplaced genomic scaffold scaffold4195323, whole genome shotgun sequence	protozoa'
CP004849.1 Alteromonas macleodii str. 'Ionian Sea U4', complete genome	bacteria'
CP006618.1 Anaplasma phagocytophilum str. Dog2 genome	bacteria'
KE504634.1 Elaeis oleifera unplaced genomic scaffold o8_sc00002, whole genome shotgun sequence	plants'
KE523052.1 Blumeria graminis f. sp. tritici Bgt#70 unplaced genomic scaffold scaffold_76457, whole genome shotgun sequence	fungi'
KE525347.1 Anopheles sinensis unplaced genomic scaffold AS2_scf7180000696055, whole genome shotgun sequence	invertebrates'
KE526711.1 Genlisea aurea unplaced genomic scaffold scaffold9, whole genome shotgun sequence	plants'
KE525743.1 Dactylellina haptotyla CBS 200.50 unplaced genomic scaffold scaffold00320, whole genome shotgun sequence	fungi'
KE537387.1 Leishmania turanica strain LEM423 unplaced genomic scaffold Scaffold0, whole genome shotgun sequence	protozoa'
KE561209.1 Rozella allomycis CSF55 unplaced genomic scaffold scaffold_4359, whole genome shotgun sequence	fungi'
KE546965.1 Gyalolechia flavorubescens KoLRI002931 unplaced genomic scaffold Caloplaca_flavorubescens_scaffold_0, whole genome shotgun sequence	fungi'
KE549796.1 Haemonchus contortus strain McMaster unplaced genomic scaffold Hco_v4_scaf_scaffold11179, whole genome shotgun sequence	invertebrates'
KE558520.1 Lipotes vexillifer unplaced genomic scaffold scaffold382, whole genome shotgun sequence	vertebrates_mammals'
KE557376.1 Homo sapiens unplaced genomic scaffold scaf00388_reg01, whole genome shotgun sequence	vertebrates_mammals'
GK000053.2 TPA_asm: Homo sapiens chromosome 18 map 18cen assembly LinearCen1.1, whole genome shotgun sequence	vertebrates_mammals'
ATMH01009925.1 Strigomonas culicis Cont9925, whole genome shotgun sequence	protozoa'
ATMG01014581.1 Angomonas deanei Cont14584, whole genome shotgun sequence	protozoa'
CM002081.1 Elaeis guineensis chromosome 1, whole genome shotgun sequence	plants'
CAUG01000632.1 Cryptococcus flavescens NRRL Y-50378 WGS project CAUG00000000 data, contig NODE_1893_length_347047_cov_46_114624, whole genome shotgun sequence	fungi'
CM002048.1 Mycobacterium tuberculosis PanR0704 chromosome, whole genome shotgun sequence	bacteria'
HG316454.1 Zygosaccharomyces bailii CLIB 213 genomic, scaffold01	fungi'
CM002049.1 Mycobacterium tuberculosis PanR0201 chromosome, whole genome shotgun sequence	bacteria'
CM002050.1 Mycobacterium tuberculosis PanR0802 chromosome, whole genome shotgun sequence	bacteria'
CM002258.1 Agrobacterium tumefaciens P4 chromosome 1, whole genome shotgun sequence	bacteria'
CM002051.1 Mycobacterium tuberculosis PanR1005 chromosome, whole genome shotgun sequence	bacteria'
KE561408.1 Leishmania gerbilli strain LEM452 unplaced genomic scaffold Scaffold0, whole genome shotgun sequence	protozoa'
KE581409.1 Phytophthora cambivora strain CBS 114087 unplaced genomic scaffold scaffold1928270, whole genome shotgun sequence	protozoa'
CM002052.1 Mycobacterium tuberculosis PanR0203 chromosome, whole genome shotgun sequence	bacteria'
CM002053.1 Mycobacterium tuberculosis PanR0205 chromosome, whole genome shotgun sequence	bacteria'
CM002054.1 Mycobacterium tuberculosis PanR0206 chromosome, whole genome shotgun sequence	bacteria'
CM002055.1 Mycobacterium tuberculosis PanR0208 chromosome, whole genome shotgun sequence	bacteria'
CM002057.1 Mycobacterium tuberculosis PanR0209 chromosome, whole genome shotgun sequence	bacteria'
CM002056.1 Mycobacterium tuberculosis PanR0207 chromosome, whole genome shotgun sequence	bacteria'
CM002058.1 Mycobacterium tuberculosis PanR0412 chromosome, whole genome shotgun sequence	bacteria'
CM002059.1 Mycobacterium tuberculosis PanR0410 chromosome, whole genome shotgun sequence	bacteria'
CM002060.1 Mycobacterium tuberculosis PanR0411 chromosome, whole genome shotgun sequence	bacteria'
CM002061.1 Mycobacterium tuberculosis PanR0409 chromosome, whole genome shotgun sequence	bacteria'
CM002062.1 Mycobacterium tuberculosis PanR0407 chromosome, whole genome shotgun sequence	bacteria'
CM002063.1 Mycobacterium tuberculosis PanR0405 chromosome, whole genome shotgun sequence	bacteria'
CM002064.1 Mycobacterium tuberculosis PanR0404 chromosome, whole genome shotgun sequence	bacteria'
CM002065.1 Mycobacterium tuberculosis PanR0403 chromosome, whole genome shotgun sequence	bacteria'
CM002066.1 Mycobacterium tuberculosis PanR0402 chromosome, whole genome shotgun sequence	bacteria'
CM002067.1 Mycobacterium tuberculosis PanR0401 chromosome, whole genome shotgun sequence	bacteria'
CM002068.1 Mycobacterium tuberculosis PanR0317 chromosome, whole genome shotgun sequence	bacteria'
CM002069.1 Mycobacterium tuberculosis PanR0316 chromosome, whole genome shotgun sequence	bacteria'
CM002070.1 Mycobacterium tuberculosis PanR0315 chromosome, whole genome shotgun sequence	bacteria'
CM002073.1 Mycobacterium tuberculosis PanR0314 chromosome, whole genome shotgun sequence	bacteria'
CM002071.1 Mycobacterium tuberculosis PanR0309 chromosome, whole genome shotgun sequence	bacteria'
CM002072.1 Mycobacterium tuberculosis PanR0313 chromosome, whole genome shotgun sequence	bacteria'
CM002076.1 Mycobacterium tuberculosis PanR0308 chromosome, whole genome shotgun sequence	bacteria'
CM002077.1 Mycobacterium tuberculosis PanR0307 chromosome, whole genome shotgun sequence	bacteria'
CM002079.1 Mycobacterium tuberculosis PanR0306 chromosome, whole genome shotgun sequence	bacteria'
CM002074.1 Mycobacterium tuberculosis PanR0304 chromosome, whole genome shotgun sequence	bacteria'
CM002075.1 Mycobacterium tuberculosis PanR0301 chromosome, whole genome shotgun sequence	bacteria'
CM002078.1 Mycobacterium tuberculosis PanR0305 chromosome, whole genome shotgun sequence	bacteria'
CM002080.1 Mycobacterium tuberculosis PanR0311 chromosome, whole genome shotgun sequence	bacteria'
KE598558.1 Cladonia macilenta KoLRI003786 unplaced genomic scaffold scaffold_0, whole genome shotgun sequence	fungi'
ATSW01011630.1 Botryllus schlosseri strain 356a chromosome 10 contig11630, whole genome shotgun sequence	invertebrates'
CM007213.1 Leishmania aethiopica L147 chromosome 36, whole genome shotgun sequence	protozoa'
CM002127.1 Mycobacterium tuberculosis PanR0501 chromosome, whole genome shotgun sequence	bacteria'
CM002126.1 Mycobacterium tuberculosis PanR0503 chromosome, whole genome shotgun sequence	bacteria'
CM002125.1 Mycobacterium tuberculosis PanR0505 chromosome, whole genome shotgun sequence	bacteria'
CM002124.1 Mycobacterium tuberculosis PanR0601 chromosome, whole genome shotgun sequence	bacteria'
CM002122.1 Mycobacterium tuberculosis PanR0602 chromosome, whole genome shotgun sequence	bacteria'
CM002121.1 Mycobacterium tuberculosis PanR0603 chromosome, whole genome shotgun sequence	bacteria'
CM002120.1 Mycobacterium tuberculosis PanR0604 chromosome, whole genome shotgun sequence	bacteria'
CM002123.1 Mycobacterium tuberculosis PanR0605 chromosome, whole genome shotgun sequence	bacteria'
CM002119.1 Mycobacterium tuberculosis PanR0606 chromosome, whole genome shotgun sequence	bacteria'
CM002114.1 Mycobacterium tuberculosis PanR0607 chromosome, whole genome shotgun sequence	bacteria'
CM002116.1 Mycobacterium tuberculosis PanR0609 chromosome, whole genome shotgun sequence	bacteria'
CM002118.1 Mycobacterium tuberculosis PanR0610 chromosome, whole genome shotgun sequence	bacteria'
CM002115.1 Mycobacterium tuberculosis PanR0611 chromosome, whole genome shotgun sequence	bacteria'
CM002117.1 Mycobacterium tuberculosis PanR0702 chromosome, whole genome shotgun sequence	bacteria'
CM002113.1 Mycobacterium tuberculosis PanR0703 chromosome, whole genome shotgun sequence	bacteria'
CM002111.1 Mycobacterium tuberculosis PanR0707 chromosome, whole genome shotgun sequence	bacteria'

CM002109.1 Mycobacterium tuberculosis PanR0708 chromosome, whole genome shotgun sequence	bacteria'
CM002112.1 Mycobacterium tuberculosis PanR0801 chromosome, whole genome shotgun sequence	bacteria'
CM002108.1 Mycobacterium tuberculosis PanR0803 chromosome, whole genome shotgun sequence	bacteria'
CM002110.1 Mycobacterium tuberculosis PanR0804 chromosome, whole genome shotgun sequence	bacteria'
CM002107.1 Mycobacterium tuberculosis PanR0805 chromosome, whole genome shotgun sequence	bacteria'
CM002106.1 Mycobacterium tuberculosis PanR0902 chromosome, whole genome shotgun sequence	bacteria'
CM002105.1 Mycobacterium tuberculosis PanR0903 chromosome, whole genome shotgun sequence	bacteria'
CM002104.1 Mycobacterium tuberculosis PanR0904 chromosome, whole genome shotgun sequence	bacteria'
CM002102.1 Mycobacterium tuberculosis PanR0906 chromosome, whole genome shotgun sequence	bacteria'
CM002103.1 Mycobacterium tuberculosis PanR0907 chromosome, whole genome shotgun sequence	bacteria'
CM002101.1 Mycobacterium tuberculosis PanR0908 chromosome, whole genome shotgun sequence	bacteria'
CM002100.1 Mycobacterium tuberculosis PanR0909 chromosome, whole genome shotgun sequence	bacteria'
CM002098.1 Mycobacterium tuberculosis PanR1007 chromosome, whole genome shotgun sequence	bacteria'
CM002099.1 Mycobacterium tuberculosis PanR1101 chromosome, whole genome shotgun sequence	bacteria'
CM002097.1 Mycobacterium tuberculosis PanR1006 chromosome, whole genome shotgun sequence	bacteria'
KE647164.1 Nosema apis BRL 01 unplaced genomic scaffold scf7180000046882, whole genome shotgun sequence	fungi'
KE650805.1 Candida albicans A123 unplaced genomic scaffold supercont1.48, whole genome shotgun sequence	fungi'
KE650838.1 Candida albicans A92 unplaced genomic scaffold supercont1.30, whole genome shotgun sequence	fungi'
KE650869.1 Candida albicans A203 unplaced genomic scaffold supercont1.32, whole genome shotgun sequence	fungi'
KE650910.1 Candida albicans A67 unplaced genomic scaffold supercont1.7, whole genome shotgun sequence	fungi'
KE650951.1 Candida albicans A48 unplaced genomic scaffold supercont1.55, whole genome shotgun sequence	fungi'
KE651002.1 Candida albicans CHN1 unplaced genomic scaffold supercont1.7, whole genome shotgun sequence	fungi'
KE651030.1 Candida albicans A20 unplaced genomic scaffold supercont1.6, whole genome shotgun sequence	fungi'
KE651071.1 Candida albicans 3153A unplaced genomic scaffold supercont1.58, whole genome shotgun sequence	fungi'
KE651119.1 Candida albicans A155 unplaced genomic scaffold supercont1.60, whole genome shotgun sequence	fungi'
KE651128.1 Candida albicans A84 unplaced genomic scaffold supercont1.1, whole genome shotgun sequence	fungi'
KV447881.1 Phytophthora kernoviae strain CBS 122049 unplaced genomic scaffold scaffold58756, whole genome shotgun sequence	protozoa'
KE680611.1 Cricetulus griseus strain 17A/GY chromosome 6 unlocalized genomic scaffold chr6_scaffold_0, whole genome shotgun sequence	vertebrates_mammals'
KE652172.1 Ophiocordyceps sinensis CO18 unplaced genomic scaffold OCS_Scf_1, whole genome shotgun sequence	fungi'
CM002128.1 Streptococcus sp. HSISM1 chromosome, whole genome shotgun sequence	bacteria'
CM002129.1 Enterococcus sp. HSIEG1 chromosome, whole genome shotgun sequence	bacteria'
CM002130.1 Streptococcus sp. HSISS3 chromosome, whole genome shotgun sequence	bacteria'
CM002131.1 Streptococcus sp. HSISB1 chromosome, whole genome shotgun sequence	bacteria'
CM002132.1 Streptococcus sp. HSISS1 chromosome, whole genome shotgun sequence	bacteria'
CM002133.1 Streptococcus sp. HSISS2 chromosome, whole genome shotgun sequence	bacteria'
CM002135.1 Veillonella parvula HSIVP1 chromosome, whole genome shotgun sequence	bacteria'
CM002136.1 Xanthomonas citri pv. malvacearum X18 chromosome, whole genome shotgun sequence	bacteria'
CM002029.1 Xanthomonas citri pv. malvacearum X20 chromosome, whole genome shotgun sequence	bacteria'
CM002139.1 Xanthomonas cassavae CFBP 4642 chromosome, whole genome shotgun sequence	bacteria'
KE695867.1 Alligator sinensis unplaced genomic scaffold scaffold442_1, whole genome shotgun sequence	vertebrates_non_mammals'
KE706420.1 Biomphalaria glabrata isolate BB02 unplaced genomic scaffold LGUN_random_Scaffold1, whole genome shotgun sequence	invertebrates'
KE705278.1 Cleome hassleriana unplaced genomic scaffold Cleome1, whole genome shotgun sequence	plants'
KE720815.1 Endocarpon pusillum Z07020 unplaced genomic scaffold C734253, whole genome shotgun sequence	fungi'
KE722155.1 Panthera tigris altaica isolate TaeGuk unplaced genomic scaffold scaffold53, whole genome shotgun sequence	vertebrates_mammals'
KE728806.1 Melampsora pinitorqua Mpini7 unplaced genomic scaffold scaffold1661264, whole genome shotgun sequence	fungi'
KE744832.1 Cronartium comandrae C4 unplaced genomic scaffold scaffold1895657, whole genome shotgun sequence	fungi'
KE747824.1 Pyrenophora seminiperda CCB06 unplaced genomic scaffold scaffold00028, whole genome shotgun sequence	fungi'
KE749393.1 Eidolon helvum unplaced genomic scaffold EH_scaffold_2191, whole genome shotgun sequence	vertebrates_mammals'
KI026023.1 Megaderma lyra unplaced genomic scaffold ML_scaffold_38462, whole genome shotgun sequence	vertebrates_mammals'
KI197197.1 Vigna angularis var. angularis cultivar IT213134 unplaced genomic scaffold scaffoldk_135, whole genome shotgun sequence	plants'
KE856091.1 Pteronotus parnellii unplaced genomic scaffold PP_scaffold_55135, whole genome shotgun sequence	vertebrates_mammals'
KI127945.1 Rhinolophus ferrumequinum unplaced genomic scaffold RF_scaffold_2952, whole genome shotgun sequence	vertebrates_mammals'
KE993672.1 Lethenteron camtschaticum unplaced genomic scaffold scaffold00001, whole genome shotgun sequence	vertebrates_non_mammals'
KI242139.1 Lagenaria siceraria unplaced genomic scaffold scaffold46647, whole genome shotgun sequence	plants'
KV744154.1 Phytophthora cryptogea strain CBS 418.71 unplaced genomic scaffold scaffold829328, whole genome shotgun sequence	protozoa'
CP006585.1 Desulfovibrio gigas DSM 1382 = ATCC 19364 genome	bacteria'
CP006705.1 Pseudomonas aeruginosa PAO581 genome	bacteria'
CP006728.1 Pseudomonas aeruginosa c7447m genome	bacteria'
KI273213.1 Apis dorsata unplaced genomic scaffold scaffold_74, whole genome shotgun sequence	invertebrates'
HF955515.1 Haemonchus contortus ISE/inbred ISE genomic scaffold, scaffold_pathogens_Hcontortus_scaffold_1, whole genome shotgun sequence	invertebrates'
LN902841.1 Echinococcus multilocularis genomic scaffold, pathogen_EmW_scaffold_01, whole genome shotgun sequence	invertebrates'
LN906329.1 Hymenolepis microstoma genomic scaffold, pathogens_HYM_scaffold_0001, whole genome shotgun sequence	invertebrates'
HG326877.1 Campylobacter coli 76339, complete genome	bacteria'
KI304388.1 Anguilla japonica unplaced genomic scaffold scaffold_1, whole genome shotgun sequence	vertebrates_non_mammals'
CM002176.1 Leishmania donovani strain BHU 1220 chromosome 36, whole genome shotgun sequence	protozoa'
CM002177.1 Rhodococcus sp. BCP1 chromosome, whole genome shotgun sequence	bacteria'
KI419148.1 Bubalus bubalis breed Mediterranean unplaced genomic scaffold scf7180021618025, whole genome shotgun sequence	vertebrates_mammals'
KI397142.1 Amborella trichopoda unplaced genomic scaffold AmTr_v1.0_scaffold00001, whole genome shotgun sequence	plants'
KI402176.1 Physeter catodon unplaced genomic scaffold Scaffold0, whole genome shotgun sequence	vertebrates_mammals'
KI916183.1 Anopheles sinensis strain SINENSIS unplaced genomic scaffold supercont2.1, whole genome shotgun sequence	invertebrates'
AORO02004902.1 Sus scrofa breed Tibetan scaffold487.1, whole genome shotgun sequence	vertebrates_mammals'
KI419149.1 Drosophila suzukii unplaced genomic scaffold scaffold1, whole genome shotgun sequence	invertebrates'
KI433418.1 Anopheles maculatus strain maculatus3 unplaced genomic scaffold supercont1.2, whole genome shotgun sequence	invertebrates'
KI422464.1 Anopheles culicifacies strain species A-37_1 unplaced genomic scaffold supercont1.1, whole genome shotgun sequence	invertebrates'
KI915040.1 Anopheles farauti strain FAR1 unplaced genomic scaffold supercont2.1, whole genome shotgun sequence	invertebrates'
KI421882.1 Anopheles atroparvus strain EBRO unplaced genomic scaffold supercont1.1, whole genome shotgun sequence	invertebrates'
KI919284.1 Anopheles melas strain CM1001059_A unplaced genomic scaffold supercont2.1, whole genome shotgun sequence	invertebrates'
KI915156.1 Anopheles merus strain MAF unplaced genomic scaffold supercont2.1, whole genome shotgun sequence	invertebrates'
CM002796.1 Mesorhizobium ciceri ca181 chromosome chr, whole genome shotgun sequence	bacteria'
KI440842.1 Sporothrix schenckii ATCC 58251 unplaced genomic scaffold supercont1.1, whole genome shotgun sequence	fungi'
KI516373.1 Puccinia striiformis f. sp. tritici CY32 unplaced genomic scaffold scaffold_2555, whole genome shotgun sequence	fungi'
ATNT01000586.1 Magnaporthe oryzae HN19311 NODE_1728_length_698218_cov_2.626876, whole genome shotgun sequence	fungi'
KI472552.1 Diaphorina citri unplaced genomic scaffold scaffold1, whole genome shotgun sequence	invertebrates'
KI441020.1 Sebastes rubrivinctus unplaced genomic scaffold scaffold78, whole genome shotgun sequence	vertebrates_non_mammals'
AUPR02S000001.1 Sebastes nigrocinctus scaffold_0, whole genome shotgun sequence	vertebrates_non_mammals'
ANOI01000001.1 Staphylococcus pseudintermedius E140 chromosome, whole genome shotgun sequence	bacteria'
ARWD01000001.1 Serratia plymuthica RVH1 C786_chromosome1.1_C, whole genome shotgun sequence	bacteria'
KI517384.1 Eutrema salsugineum unplaced genomic scaffold scaffold_1, whole genome shotgun sequence	plants'
KI911114.1 Cladonia metacorallifera KoLRI002260 unplaced genomic scaffold scaffold6, whole genome shotgun sequence	fungi'
CP006831.1 Pseudomonas aeruginosa PAO1-VE2 genome	bacteria'
CP006832.1 Pseudomonas aeruginosa PAO1-VE13 genome	bacteria'
KI519610.1 Poecilia formosa unplaced genomic scaffold Scaffold0, whole genome shotgun sequence	vertebrates_non_mammals'
KQ714883.1 Priapulus caudatus unplaced genomic scaffold Scaffold1, whole genome shotgun sequence	invertebrates'
CM002264.1 Xanthomonas axonopodis pv. glycines CFBP 7119 chromosome, whole genome shotgun sequence	bacteria'
CM002261.1 Xanthomonas alfalfae subsp. alfalfae CFBP 3836 chromosome, whole genome shotgun sequence	bacteria'
KI536661.1 Citrus clementina cultivar Clemenules unplaced genomic scaffold scaffold_3, whole genome shotgun sequence	plants'
HG428755.1 Escherichia coli PMV-1 main chromosome, complete genome	bacteria'
KI537095.1 Balaenoptera acutorostrata scammoni unplaced genomic scaffold scaffold1, whole genome shotgun sequence	vertebrates_mammals'
CP006844.1 Streptococcus pneumoniae A026 genome	bacteria'
KI538821.1 Populus euphratica unplaced genomic scaffold scaffold1.1, whole genome shotgun sequence	plants'
CM002268.1 Xanthomonas axonopodis pv. glycines CFBP 2526 chromosome, whole genome shotgun sequence	bacteria'
CP006846.1 Anaplasma marginale str. Gypsy Plains genome	bacteria'
CP006847.1 Anaplasma marginale str. Dawn genome	bacteria'
CM002271.1 Streptomyces sp. GBA 94-10 chromosome, whole genome shotgun sequence	bacteria'
CM002273.1 Streptomyces sp. PVA 94-07 chromosome, whole genome shotgun sequence	bacteria'
AUUT01000001.1 Camelina sativa scaffold00001, whole genome shotgun sequence	plants'
KI545873.1 Pseudozyma brasiliensis GHG001 unplaced genomic scaffold PSEUBRA_SCAF3, whole genome shotgun sequence	fungi'
KV441991.1 Candida glabrata CCTCC M202019 chromosome C unlocalized genomic scaffold CGRCMScaffold_02, whole genome shotgun sequence	fungi'
KI546166.1 Spironucleus salmonicida unplaced genomic scaffold scf7180000020683, whole genome shotgun sequence	protozoa'
CM002277.1 Burkholderia dolosa PC543 chromosome 1 chr1, whole genome shotgun sequence	bacteria'
KI546415.1 Amanita jacksonii TRTC168611 unplaced genomic scaffold V175_0106, whole genome shotgun sequence	fungi'
CM004511.1 Fusarium circinatum strain FSP 34 chromosome 1, whole genome shotgun sequence	fungi'
CM002280.1 Streptomyces niveus NCIMB 11891 chromosome, whole genome shotgun sequence	bacteria'
CM002285.1 Streptomyces roseochromogenes subsp. oscitans DS 12.976 chromosome, whole genome shotgun sequence	bacteria'
KI547134.1 Spathaspora arborariae UFMG-19.1A unplaced genomic scaffold scaffold00001, whole genome shotgun sequence	fungi'
CM002287.1 Bifidobacterium longum E18 chromosome, whole genome shotgun sequence	bacteria'
ATNU01000407.1 Magnaporthe oryzae FJ81278 NODE_1454_length_676449_cov_14.228848, whole genome shotgun sequence	fungi'
AYRD02012334.1 Diaporthe longicolla MSPL 10-6 scaffold17, whole genome shotgun sequence	fungi'
AWXF01000040.1 Naegleria fowleri strain ATCC 30863 Nfow_contig_40, whole genome shotgun sequence	protozoa'
HG723041.1 Eimeria necatrix Houghton genomic scaffold, Enh_scaff1, whole genome shotgun sequence	protozoa'
HG738137.1 Onchocerca volvulus assembly O_volvulus_Cameroon_v3 genomic scaffold, OVOC_OM1b, whole genome shotgun sequence	invertebrates'
HG670307.1 Eimeria acervulina Houghton genomic scaffold, Eah_scaff1, whole genome shotgun sequence	protozoa'
HG688746.1 Eimeria praecox Houghton genomic scaffold, Eph_scaff1, whole genome shotgun sequence	protozoa'
HF969015.2 Salmonella enterica subsp. enterica serovar Bovismorbificans str. 3114 complete genome	bacteria'
HG673746.1 Eimeria tenella Houghton genomic scaffold, Eth_scaff1, whole genome shotgun sequence	protozoa'
HG530631.1 Cryptococcus gattii CBS 7750 genomic scaffold, supercontig_1_01_of_Cryptococcus_neoformans_Serotype_B_CBS7750_SOAP_DE_NOVO, whole genome shotgun sequence	fungi'
HG718748.1 Eimeria maxima Weybridge genomic scaffold, Emw_scaff1, whole genome shotgun sequence	protozoa'
HG738131.1 Elaeophora elaphi genomic scaffold, EEL_scaffold_00001_1479205, whole genome shotgun sequence	invertebrates'
HG710173.1 Eimeria brunetti Houghton genomic scaffold, Ebh_scaff1, whole genome shotgun sequence	protozoa'
HG678410.1 Eimeria mitis Houghton genomic scaffold, Emh_scaff1, whole genome shotgun sequence	protozoa'
CM002295.1 Phaseolus vulgaris cultivar G19833 chromosome 8, whole genome shotgun sequence	plants'
KV724674.1 Phytophthora lateralis strain CBS 168.42 unplaced genomic scaffold scaffold144566, whole genome shotgun sequence	protozoa'
KV737129.1 Phytophthora pinifolia strain CBS 122922 unplaced genomic scaffold scaffold948885, whole genome shotgun sequence	protozoa'
KI570111.1 Cronartium ribicola 11-2 unplaced genomic scaffold scaffold2067960, whole genome shotgun sequence	fungi'
KI578484.1 Leptinotarsa decemlineata unplaced genomic scaffold Scaffold2, whole genome shotgun sequence	invertebrates'
KI615321.1 Peromyscus maniculatus bairdii unplaced genomic scaffold Scaffold4, whole genome shotgun sequence	vertebrates_mammals'
KI603969.1 Cronartium quercuum f. sp. banksianae CqE3WM unplaced genomic scaffold scaffold6044139, whole genome shotgun sequence	fungi'
KI614512.1 Endocronartium harknessii PhW48OC unplaced genomic scaffold scaffold971408, whole genome shotgun sequence	fungi'
KI628541.1 Sclerotinia borealis F-4128 unplaced genomic scaffold scaffold00001, whole genome shotgun sequence	fungi'
KI629971.1 Ceratosolen solmsi marchali unplaced genomic scaffold scaffold10, whole genome shotgun sequence	invertebrates'
KI630171.1 Mimulus guttatus cultivar IM62 unplaced genomic scaffold scaffold_1, whole genome shotgun sequence	plants'
KI635482.1 Lecanosticta acicola CBS 871.95 unplaced genomic scaffold scaffold59563, whole genome shotgun sequence	fungi'
AWYD01000740.1 Pseudocercospora pini-densiflorae CBS 125139 contig101650_0, whole genome shotgun sequence	fungi'
KI633359.1 Mycosphaerella laricina CBS 326.52 unplaced genomic scaffold scaffold90652, whole genome shotgun sequence	fungi'
KI633058.1 Mycosphaerella sp. Ston1 unplaced genomic scaffold scaffold279717, whole genome shotgun sequence	fungi'

KI635736.1 Plasmodium yoelii 17X unplaced genomic scaffold supercont2.21, whole genome shotgun sequence	protozoa'
CM002307.1 Xanthomonas hortorum pv. carotae str. M081 chromosome, whole genome shotgun sequence	bacteria'
KI639851.1 Steinernema monticolum unplaced genomic scaffold MONTI_736, whole genome shotgun sequence	invertebrates'
CP006735.1 Thermosynechococcus sp. NK55 genome	bacteria'
KI652990.1 Ephemera danica unplaced genomic scaffold Scaffold28, whole genome shotgun sequence	invertebrates'
DF239015.1 Symbiodinium minutum Mf 1.05b.01 DNA, scaffold: scaffold2.1, whole genome shotgun sequence	protozoa'
KI657455.1 Necator americanus unplaced genomic scaffold N_americanus-1.0_Cont1, whole genome shotgun sequence	invertebrates'
KI669492.1 Kwoniella heveanensis BCC8398 unplaced genomic scaffold supercont1.1, whole genome shotgun sequence	fungi'
KI669518.1 Kwoniella heveanensis CBS 569 unplaced genomic scaffold supercont2.1, whole genome shotgun sequence	fungi'
KI669472.1 Kwoniella mangroviensis CBS 8507 unplaced genomic scaffold supercont2.1, whole genome shotgun sequence	fungi'
KI669547.1 Kwoniella mangroviensis CBS 8886 unplaced genomic scaffold supercont1.1, whole genome shotgun sequence	fungi'
KI669459.1 Kwoniella mangroviensis CBS 10435 unplaced genomic scaffold supercont2.1, whole genome shotgun sequence	fungi'
CM002308.1 Chlamydia pecorum DBDeUG chromosome, whole genome shotgun sequence	bacteria'
CM002309.1 Chlamydia pecorum IPTaLE chromosome, whole genome shotgun sequence	bacteria'
CM002310.1 Chlamydia pecorum MC/MarsBar chromosome, whole genome shotgun sequence	bacteria'
CM002311.1 Chlamydia pecorum VR629 chromosome, whole genome shotgun sequence	bacteria'
DF396901.1 Saccharomyces cerevisiae NAM34-4C DNA, contig: scaffold021, whole genome shotgun sequence	fungi'
DF396954.1 Saccharomyces cerevisiae IR-2 DNA, contig: scaffold018, whole genome shotgun sequence	fungi'
KI685158.1 Phytophthora parasitica strain CJ02B3 unplaced genomic scaffold supercont1.1353, whole genome shotgun sequence	protozoa'
KI671675.1 Phytophthora parasitica strain CJ05E6 unplaced genomic scaffold supercont1.1407, whole genome shotgun sequence	protozoa'
KI678687.1 Phytophthora parasitica strain CHvinca01 unplaced genomic scaffold supercont1.1606, whole genome shotgun sequence	protozoa'
KI691933.1 Phytophthora parasitica strain IAC_01/95 unplaced genomic scaffold supercont1.1639, whole genome shotgun sequence	protozoa'
KI697133.1 Beta vulgaris subsp. vulgaris cultivar KWS_DH1440 unplaced genomic scaffold scaffold61, whole genome shotgun sequence	plants'
KI744491.1 Beta vulgaris subsp. vulgaris cultivar STR06A6001 unplaced genomic scaffold scaffold603, whole genome shotgun sequence	plants'
KI723259.1 Beta vulgaris subsp. vulgaris cultivar SynMono DH unplaced genomic scaffold scaffold8048, whole genome shotgun sequence	plants'
KI784202.1 Beta vulgaris subsp. vulgaris cultivar SynTilling DH unplaced genomic scaffold scaffold1035, whole genome shotgun sequence	plants'
CM002317.1 Beta vulgaris subsp. vulgaris cultivar KWS2320 chromosome 6, whole genome shotgun sequence	plants'
KQ207791.1 Spinacia oleracea cultivar SynViroflay unplaced genomic scaffold scaffold77373, whole genome shotgun sequence	plants'
CM002326.2 Beta vulgaris subsp. vulgaris chromosome 6, whole genome shotgun sequence	plants'
BASJ02000084.1 Coturnix japonica DNA, scaffold: scaffold328_cov160, strain: L, whole genome shotgun sequence	vertebrates_non_mammals'
BATS01000001.1 Fragaria x ananassa DNA, contig: FANhyb_rscf00000001.1, whole genome shotgun sequence	plants'
DF277771.1 Fragaria x ananassa DNA, scaffold: FAN_iscf00031532.1, whole genome shotgun sequence	plants'
BATU01046052.1 Fragaria iinumae DNA, contig: FII_iscf00046135.1, whole genome shotgun sequence	plants'
BATW01092732.1 Fragaria nubicola DNA, contig: FNU_iscf00093430.1, whole genome shotgun sequence	plants'
BATV01027002.1 Fragaria nipponica DNA, contig: FNI_iscf00027903.1, whole genome shotgun sequence	plants'
KI867152.1 Capsicum annuum cultivar CM334 unplaced genomic scaffold PGAv.1.5.scaffold1, whole genome shotgun sequence	plants'
CM002330.1 Pseudomonas moraviensis R28-S chromosome, whole genome shotgun sequence	bacteria'
DF340865.1 Dianthus caryophyllus DNA, scaffold: scaffold2, whole genome shotgun sequence	plants'
KI894027.1 Cryptococcus dejecticola CBS 10117 unplaced genomic scaffold supercont1.1, whole genome shotgun sequence	fungi'
KI894018.1 Cryptococcus bestiolae CBS 10118 unplaced genomic scaffold supercont1.1, whole genome shotgun sequence	fungi'
KI894007.1 Cryptococcus pinus CBS 10737 unplaced genomic scaffold supercont1.1, whole genome shotgun sequence	fungi'
CM002361.1 Sesamum indicum cultivar Zhongzhi No. 13 linkage group LG6, whole genome shotgun sequence	plants'
HG380758.1 Adineta vaga genomic scaffold, scaffold_1, whole genome shotgun sequence	invertebrates'
HG792015.1 Penicillium roqueforti FM164 genomic scaffold, ProqFM164S01, whole genome shotgun sequence	fungi'
HG793134.1 Penicillium camemberti str. FM013, whole genome shotgun sequence	fungi'
CBVU010000006.1 Erwinia amylovora LA637 WGS project CBVU000000000 data, contig LA637_CONTIG_6, whole genome shotgun sequence	bacteria'
CBVT010000006.1 Erwinia amylovora LA636 WGS project CBVT000000000 data, contig LA636_CONTIG_6, whole genome shotgun sequence	bacteria'
CBVS010000006.1 Erwinia amylovora LA635 WGS project CBVS000000000 data, contig LA635_CONTIG_6, whole genome shotgun sequence	bacteria'
KI911139.1 Trichoderma reesei RUT C-30 unplaced genomic scaffold TRIREscaffold_1, whole genome shotgun sequence	fungi'
AZIM01000080.1 Ophiophagus hannah scaffold80.1, whole genome shotgun sequence	vertebrates_non_mammals'
KI912109.1 Pestalotiopsis fici W106-1 unplaced genomic scaffold PFICI_Scf_1, whole genome shotgun sequence	fungi'
BATX01138262.1 Fragaria orientalis DNA, contig: FOR_iscf00143214.1, whole genome shotgun sequence	plants'
AWNI01000008.1 Moesziomyces aphidis DSM 70725 Seq17, whole genome shotgun sequence	fungi'
KI894189.1 Limulus polyphemus unplaced genomic scaffold Scaffold0, whole genome shotgun sequence	invertebrates'
KI913114.1 Aphanomyces astaci strain APO3 unplaced genomic scaffold supercont1.1, whole genome shotgun sequence	protozoa'
KI913954.1 Aphanomyces invadans strain NJM9701 unplaced genomic scaffold supercont1.3, whole genome shotgun sequence	protozoa'
DF260912.1 Rhodovulum sulfidophilum DSM 1374 DNA, scaffold: scaffoldContig1, whole genome shotgun sequence	bacteria'
KI925184.1 Plasmodium falciparum Vietnam Oak-Knoll (FVO) unplaced genomic scaffold supercont1.178, whole genome shotgun sequence	protozoa'
KI925625.1 Plasmodium falciparum MaliPS096_E11 unplaced genomic scaffold supercont1.157, whole genome shotgun sequence	protozoa'
KI926445.1 Plasmodium falciparum Tanzania (2000708) unplaced genomic scaffold supercont1.196, whole genome shotgun sequence	protozoa'
KI926126.1 Plasmodium falciparum NF135/5.C10 unplaced genomic scaffold supercont1.111, whole genome shotgun sequence	protozoa'
KI927384.1 Plasmodium falciparum Palo Alto/Uganda unplaced genomic scaffold supercont1.147, whole genome shotgun sequence	protozoa'
KI927620.1 Plasmodium falciparum CAMP/Malaysia unplaced genomic scaffold supercont1.165, whole genome shotgun sequence	protozoa'
KI928025.1 Plasmodium falciparum FCH/4 unplaced genomic scaffold supercont2.239, whole genome shotgun sequence	protozoa'
CM002372.1 Streptococcus thermophilus M17PTZA496 chromosome, whole genome shotgun sequence	bacteria'
CM002369.1 Streptococcus thermophilus TH1435 chromosome, whole genome shotgun sequence	bacteria'
CM002370.1 Streptococcus thermophilus TH1436 chromosome, whole genome shotgun sequence	bacteria'
CM002371.1 Streptococcus thermophilus MTH17CL396 chromosome, whole genome shotgun sequence	bacteria'
BASO01002930.1 Arabidopsis halleri subsp. gemmifera DNA, contig: scaffold2930, whole genome shotgun sequence	plants'
CM002373.1 Cynoglossus semilaevis isolate Cse_v1.0 chromosome 1, whole genome shotgun sequence	vertebrates_non_mammals'
KI964537.1 Bipolaris zeicola 26-R-13 unplaced genomic scaffold scaffold_0, whole genome shotgun sequence	fungi'
KI963918.1 Bipolaris oryzae ATCC 44560 unplaced genomic scaffold scaffold_0, whole genome shotgun sequence	fungi'
KI963911.1 Penicillium chrysogenum NCPC10086 unplaced genomic scaffold NCPC10086_scaf148, whole genome shotgun sequence	fungi'
KI965460.1 Plasmodium inui San Antonio 1 unplaced genomic scaffold supercont1.1, whole genome shotgun sequence	protozoa'
KI965394.1 Plasmodium vinckei petteri strain CR unplaced genomic scaffold supercont1.1, whole genome shotgun sequence	protozoa'
JATN01000322.1 Rhizoctonia solani AG-3 Rhs1AP strain AG-3 scf1119142671170, whole genome shotgun sequence	fungi'
BASP01010265.1 Arabidopsis lyrata subsp. petraea DNA, contig: scaffold10265, whole genome shotgun sequence	plants'
KI966371.1 Drechslerella stenobrocha 248 unplaced genomic scaffold DRE_Scf_1, whole genome shotgun sequence	fungi'
JAQJ01010622.1 Drosophila simulans strain tsimbazaza sim_ext_bow_ExtContig_13582, whole genome shotgun sequence	invertebrates'
JAQR01002560.1 Drosophila sechellia strain 14021-0248.25 Sec_plus_rsq_contig_826, whole genome shotgun sequence	invertebrates'
KI968691.1 Bipolaris victoriae FI3 unplaced genomic scaffold scaffold_0, whole genome shotgun sequence	fungi'
HG803175.1 Brucella canis str. Oliveri chromosome I, genome	bacteria'
CBTC010000046.1 Zygosaccharomyces bailii ISA1307 WGS project CBTC000000000, contig ZBAI_A_scaffold_046, whole genome shotgun sequence	fungi'
HG530068.1 Pseudomonas aeruginosa PA38182, complete genome	bacteria'
HG009209.1 Serinus canaria genomic scaffold, SCA1_u1, whole genome shotgun sequence	vertebrates_non_mammals'
CM002401.1 Bacillus anthracis 8903-G chromosome, whole genome shotgun sequence	bacteria'
CM002398.1 Bacillus anthracis 9080-G chromosome, whole genome shotgun sequence	bacteria'
CM002395.1 Bacillus anthracis 52-G chromosome, whole genome shotgun sequence	bacteria'
CP006940.1 Listeria monocytogenes serotype 1/2a str. 01-1280 genome	bacteria'
CM002407.1 Saccharomyces cerevisiae R008 chromosome IV, whole genome shotgun sequence	fungi'
CM002425.1 Saccharomyces cerevisiae P301 chromosome IV, whole genome shotgun sequence	fungi'
CM002335.1 Saccharomyces cerevisiae P283 chromosome IV, whole genome shotgun sequence	fungi'
CM002441.1 Saccharomyces cerevisiae R103 chromosome IV, whole genome shotgun sequence	fungi'
CP005829.1 Borrelia anserina BA2, complete genome	bacteria'
CP004146.1 Borrelia hermsii YOR, complete genome	bacteria'
CP004217.2 Borrelia miyamotoi FR64b, complete genome	bacteria'
CP004267.1 Borrelia crocidurae DOU, complete genome	bacteria'
CP005851.2 Borrelia parkeri SLO, complete genome	bacteria'
CP005745.1 Borrelia coriaceae Co53, complete genome	bacteria'
CP005706.1 Borrelia hermsii YBT, complete genome	bacteria'
CP005680.1 Borrelia hermsii MTW, complete genome	bacteria'
CM002455.1 Nannochloropsis gaditana strain B-31 chromosome 1, whole genome shotgun sequence	protozoa'
KQ464259.1 Microplitis demolitor strain Queensland-Clemson subculture unplaced genomic scaffold Mdem_scaffold_0079, whole genome shotgun sequence	invertebrates'
CM003136.1 Streptococcus thermophilus TH982 chromosome, whole genome shotgun sequence	bacteria'
CM003135.1 Streptococcus thermophilus TH1477 chromosome chr, whole genome shotgun sequence	bacteria'
KK060624.1 Schmidtea mediterranea strain CIW4 unplaced genomic scaffold scaffold7446, whole genome shotgun sequence	invertebrates'
CM002488.1 Oryza punctata chromosome 1, whole genome shotgun sequence	plants'
CM002500.1 Oryza nivara chromosome 1, whole genome shotgun sequence	plants'
CM002512.1 Oryza glumipatula chromosome 1, whole genome shotgun sequence	plants'
CP007225.1 Agrobacterium tumefaciens LBA4213 (Ach5) circular chromosome	bacteria'
CP006963.1 Acinetobacter baumannii PKAB07 genome	bacteria'
HG793125.1 Kuraishia capsulata CBS 1993 genomic scaffold, Kuraishia_capsulata_scaffold_1, whole genome shotgun sequence	fungi'
HG814183.1 Penicillium fuscoglaucum FM041 genomic scaffold, PfusFM041S001, whole genome shotgun sequence	fungi'
HG916852.1 Rhizobium sp. LPU83 main chrosome complete genome	bacteria'
HG815136.1 Penicillium nalgiovense FM193 genomic scaffold, PnalFM193S001, whole genome shotgun sequence	fungi'
HG816029.1 Penicillium carneum LCP05634 genomic scaffold, PcarLCP05634S0001, whole genome shotgun sequence	fungi'
HG813308.1 Penicillium paneum FM227 genomic scaffold, PpanFM227S001, whole genome shotgun sequence	fungi'
HG813601.1 Penicillium biforme FM169 genomic scaffold, PbifFM169S001, whole genome shotgun sequence	fungi'
HF955061.1 Phytomonas sp. isolate EM1 genomic scaffold, scaffold_1, whole genome shotgun sequence	protozoa'
CBMN010001971.1 Hordeum pubiflorum WGS project CBMN00000000 data, cultivar BCC2028, contig Hpubiflorum_contig_1971, whole genome shotgun sequence	plants'
JARH01000012.1 Colletotrichum fioriniae PJ7 Scaffold_1008.1, whole genome shotgun sequence	fungi'
CP007236.1 Ensifer adhaerens OV14 chromosome 1 sequence	bacteria'
CP007268.1 Halorhodospira halochloris str. A genome	bacteria'
CP006980.1 Pseudomonas aeruginosa LESlike5 sequence	bacteria'
CP006981.1 Pseudomonas aeruginosa LESlike7 sequence	bacteria'
CP006982.1 Pseudomonas aeruginosa LES400 sequence	bacteria'
CP006983.1 Pseudomonas aeruginosa LESB65 sequence	bacteria'
CP006984.1 Pseudomonas aeruginosa LESlike1 sequence	bacteria'
CP006985.1 Pseudomonas aeruginosa LESlike4 sequence	bacteria'
AMGW01000001.1 Cladophialophora yegresii CBS 114405 aczIN-supercont1.1, whole genome shotgun sequence	fungi'
AMGX01000001.1 Cladophialophora psammophila CBS 110553 aczJe-supercont1.1, whole genome shotgun sequence	fungi'
AMGY01000001.1 Capronia epimyces CBS 606.96 aczIQ-supercont1.1, whole genome shotgun sequence	fungi'
AMWN01000001.1 Capronia coronata CBS 617.96 acBCG-supercont1.1, whole genome shotgun sequence	fungi'
AZCJ01000001.1 Saccharomyces pastorianus CBS 1513 scaffold00001, whole genome shotgun sequence	fungi'
KK076434.1 Mucor irregularis B50 unplaced genomic scaffold scaffold12.1, whole genome shotgun sequence	fungi'
CM002545.1 Xanthomonas campestris pv. campestris str. CFBP 1869 chromosome, whole genome shotgun sequence	bacteria'
CM002673.1 Xanthomonas campestris pv. campestris str. CFBP 5817 chromosome, whole genome shotgun sequence	bacteria'
CM002635.1 Xanthomonas campestris pv. incanae strain CFBP 1606R chromosome, whole genome shotgun sequence	bacteria'
CM002636.1 Xanthomonas campestris pv. incanae strain CFBP 2527R chromosome, whole genome shotgun sequence	bacteria'
CM002637.1 Xanthomonas campestris pv. raphani strain CFBP 5828R chromosome, whole genome shotgun sequence	bacteria'
CM002638.1 Xanthomonas campestris strain CFBP 5825R chromosome, whole genome shotgun sequence	bacteria'
CP006672.1 Chlamydia trachomatis strain F/2-93 genome	bacteria'
CP006675.1 Chlamydia trachomatis strain E/12-94 genome	bacteria'
CP006677.1 Chlamydia trachomatis strain D/14-96 genome	bacteria'
CP006678.1 Chlamydia trachomatis strain Ia20-97 genome	bacteria'

CP006679.1 Chlamydia trachomatis strain J/27-97 genome	bacteria'
CM002782.1 Yarrowia lipolytica PO1f chromosome E, whole genome shotgun sequence	fungi'
KK076539.1 Eurytemora affinis strain Atlantic clade unplaced genomic scaffold Scaffold1, whole genome shotgun sequence	invertebrates'
JELW01000001.1 Metarhizium robertsii strain ARSEF 2575 contig001, whole genome shotgun sequence	fungi'
JAQW01000001.1 Rhizophagus irregularis DAOM 197198w isolate N31 N31_scaffold1, whole genome shotgun sequence	fungi'
JAQX01000001.1 Rhizophagus irregularis DAOM 197198w isolate N33 N33_scaffold1, whole genome shotgun sequence	fungi'
JAQY01000001.1 Rhizophagus irregularis DAOM 197198w isolate N36 N36_scaffold1, whole genome shotgun sequence	fungi'
JAQZ01000001.1 Rhizophagus irregularis DAOM 197198w isolate N6 Scaffold1, whole genome shotgun sequence	fungi'
JARA01000001.1 Rhizophagus irregularis DAOM 197198w isolate DNA1 scaffold1, whole genome shotgun sequence	fungi'
JARB01000001.1 Rhizophagus irregularis DAOM 197198w isolate DNA2 scaffold1, whole genome shotgun sequence	fungi'
JEMT01027768.1 Rhizophagus irregularis DAOM 197198w jcf7180003193370, whole genome shotgun sequence	fungi'
CM002651.1 Fusarium graminearum strain CS3005 chromosome 1, whole genome shotgun sequence	fungi'
AWGT01000406.1 Colinus virginianus strain Texas NorthernBobwhite_ScaffoldedDeNovo_contig_406, whole genome shotgun sequence	vertebrates_non_mammals'
AWGU01031816.1 Colinus virginianus strain Texas NorthernBobwhite_SimpleDeNovo_contig_31816, whole genome shotgun sequence	vertebrates_non_mammals'
KK082680.1 Trichogramma pretiosum unplaced genomic scaffold Scaffold1, whole genome shotgun sequence	invertebrates'
KK088411.1 Aspergillus ruber CBS 135680 unplaced genomic scaffold scaffold00001, whole genome shotgun sequence	fungi'
KK090061.1 Pyrenochaeta lycopersici CRA-PAV_ER 1211 unplaced genomic scaffold NODE_58991_length_768071_cov_66.298904, whole genome shotgun sequence	fungi'
KK090494.1 Brassica oleracea var. capitata cultivar line 02-12 unplaced genomic scaffold Scaffold000002, whole genome shotgun sequence	plants'
HF972804.1 Neurospora sublineolata FGSC 5508 genomic scaffold, SC_118, whole genome shotgun sequence	fungi'
HF970592.1 Neurospora africana FGSC 1740 genomic scaffold, SC_2, whole genome shotgun sequence	fungi'
HF975737.1 Neurospora pannonica FGSC 7221 genomic scaffold, SC_82, whole genome shotgun sequence	fungi'
HF979336.1 Neurospora terricola FGSC 1889 genomic scaffold, SC_130, whole genome shotgun sequence	fungi'
CAOW010096036.1 Camelus bactrianus WGS project CAOW00000000 data, contig 113605, whole genome shotgun sequence	vertebrates_mammals'
CM002678.1 Monoraphidium neglectum strain SAG 48.87 chloroplast CP, complete sequence, whole genome shotgun sequence	plants'
KK100010.1 Rhizomucor miehei CAU432 unplaced genomic scaffold RhzM_scaffold1, whole genome shotgun sequence	fungi'
KK106941.1 Endocarpon pusillum strain KoLRI No. LF000583 unplaced genomic scaffold Scaffold_1, whole genome shotgun sequence	fungi'
KK106981.1 Umbilicaria muehlenbergii strain KoLRI No. LF000956 unplaced genomic scaffold Scaffold_1, whole genome shotgun sequence	fungi'
KK107063.1 Cerapachys biroi unplaced genomic scaffold scaffold32, whole genome shotgun sequence	invertebrates'
KK115971.1 Stegodyphus mimosarum unplaced genomic scaffold scaffold1156, whole genome shotgun sequence	invertebrates'
KK198108.1 Orussus abietinus isolate OABI.00-Male unplaced genomic scaffold Scaffold1, whole genome shotgun sequence	invertebrates'
CM002679.1 Gossypium arboreum cultivar Shixiya1 chromosome 1, whole genome shotgun sequence	plants'
KK198755.1 Eucalyptus grandis cultivar BRASUZ1 unplaced genomic scaffold scaffold_3, whole genome shotgun sequence	plants'
HG803548.1 Trichuris muris Edinburgh genomic scaffold, scaffold1, whole genome shotgun sequence	invertebrates'
CBZK010000001.2 Ursus arctos WGS project CBZK000000000 data, contig scaffold_297, whole genome shotgun sequence	vertebrates_mammals'
HG805809.1 Trichuris trichiura genomic scaffold, TTRE_000001, whole genome shotgun sequence	invertebrates'
HG934063.1 Yarrowia lipolytica WSH-Z06 complete genome, chromosome YALIOE	fungi'
KK203934.1 Trichophyton rubrum MR850 unplaced genomic scaffold supercont1.232, whole genome shotgun sequence	fungi'
KK204238.1 Trichophyton interdigitale H6 unplaced genomic scaffold supercont2.1, whole genome shotgun sequence	fungi'
KK207357.1 Trichophyton rubrum CBS 100081 chromosome Unknown supercont1.289, whole genome shotgun sequence	fungi'
KK207889.1 Trichophyton rubrum CBS 288.86 unplaced genomic scaffold supercont1.271, whole genome shotgun sequence	fungi'
KK208427.1 Trichophyton rubrum CBS 289.86 unplaced genomic scaffold supercont1.270, whole genome shotgun sequence	fungi'
KK208846.1 Trichophyton soudanense CBS 452.61 unplaced genomic scaffold supercont1.168, whole genome shotgun sequence	fungi'
KK209394.1 Trichophyton rubrum MR1448 unplaced genomic scaffold supercont1.296, whole genome shotgun sequence	fungi'
KK209984.1 Trichophyton rubrum MR1459 unplaced genomic scaffold supercont1.314, whole genome shotgun sequence	fungi'
KK210299.1 Trichophyton rubrum CBS 735.88 unplaced genomic scaffold supercont1.143, whole genome shotgun sequence	fungi'
KK210757.1 Trichophyton rubrum CBS 202.88 unplaced genomic scaffold supercont2.259, whole genome shotgun sequence	fungi'
KL203742.1 Nannospalax galili isolate Female #2095 unplaced genomic scaffold scaffold140, whole genome shotgun sequence	vertebrates_mammals'
CP007270.2 Salmonella enterica subsp. enterica serovar Enteritidis str. EC20121176 genome	bacteria'
CP007274.2 Salmonella enterica subsp. enterica serovar Enteritidis str. SA20093266 genome	bacteria'
CP007266.2 Salmonella enterica subsp. enterica serovar Enteritidis str. EC20110223 genome	bacteria'
CP007362.2 Salmonella enterica subsp. enterica serovar Enteritidis str. EC20120544 genome	bacteria'
CP007363.2 Salmonella enterica subsp. enterica serovar Enteritidis str. EC20120548 genome	bacteria'
CP007364.2 Salmonella enterica subsp. enterica serovar Enteritidis str. EC20120555 genome	bacteria'
CP007289.2 Salmonella enterica subsp. enterica serovar Enteritidis str. SA19982831 genome	bacteria'
CP007290.2 Salmonella enterica subsp. enterica serovar Enteritidis str. SA19983126 genome	bacteria'
CP007293.2 Salmonella enterica subsp. enterica serovar Enteritidis str. SA20083456 genome	bacteria'
CP007294.2 Salmonella enterica subsp. enterica serovar Enteritidis str. SA20083636, complete genome	bacteria'
CP007295.2 Salmonella enterica subsp. enterica serovar Enteritidis str. SA20084384 genome	bacteria'
CP007277.2 Salmonella enterica subsp. enterica serovar Enteritidis str. SA19930684 genome	bacteria'
CP007278.2 Salmonella enterica subsp. enterica serovar Enteritidis str. SA19942384 genome	bacteria'
CP007279.2 Salmonella enterica subsp. enterica serovar Enteritidis str. SA19943269 genome	bacteria'
CP007281.2 Salmonella enterica subsp. enterica serovar Enteritidis str. SA19961622 genome	bacteria'
CP007284.2 Salmonella enterica subsp. enterica serovar Enteritidis str. SA19971331 genome	bacteria'
CP007320.2 Salmonella enterica subsp. enterica serovar Enteritidis str. EC20090135 genome	bacteria'
CP007321.2 Salmonella enterica subsp. enterica serovar Enteritidis str. EC20090193 genome	bacteria'
CP007322.2 Salmonella enterica subsp. enterica serovar Enteritidis str. EC20090332 genome	bacteria'
CP007300.2 Salmonella enterica subsp. enterica serovar Enteritidis str. SA20090877 genome	bacteria'
CP007301.2 Salmonella enterica subsp. enterica serovar Enteritidis str. SA20091739 genome	bacteria'
CP007302.2 Salmonella enterica subsp. enterica serovar Enteritidis str. SA20093421 genome	bacteria'
CP007303.2 Salmonella enterica subsp. enterica serovar Enteritidis str. SA20093430 genome	bacteria'
CP007304.2 Salmonella enterica subsp. enterica serovar Enteritidis str. SA20093538 genome	bacteria'
CP007305.2 Salmonella enterica subsp. enterica serovar Enteritidis str. SA20093543 genome	bacteria'
CP007306.2 Salmonella enterica subsp. enterica serovar Enteritidis str. SA20093784 genome	bacteria'
CP007307.2 Salmonella enterica subsp. enterica serovar Enteritidis str. SA20093788 genome	bacteria'
CP007308.2 Salmonella enterica subsp. enterica serovar Enteritidis str. SA20093950 genome	bacteria'
CP007309.2 Salmonella enterica subsp. enterica serovar Enteritidis str. SA20093977 genome	bacteria'
CP007310.2 Salmonella enterica subsp. enterica serovar Enteritidis str. SA20094079 genome	bacteria'
CP007311.2 Salmonella enterica subsp. enterica serovar Enteritidis str. SA20094350 genome	bacteria'
CP007313.2 Salmonella enterica subsp. enterica serovar Enteritidis str. SA20094383 genome	bacteria'
CP007314.2 Salmonella enterica subsp. enterica serovar Enteritidis str. SA20094389 genome	bacteria'
CP007315.2 Salmonella enterica subsp. enterica serovar Enteritidis str. SA20094521 genome	bacteria'
CP007316.2 Salmonella enterica subsp. enterica serovar Enteritidis str. SA20094642 genome	bacteria'
CP007334.2 Salmonella enterica subsp. enterica serovar Enteritidis str. SA20092320 genome	bacteria'
CP007317.2 Salmonella enterica subsp. enterica serovar Enteritidis str. SA20094803 genome	bacteria'
CP007318.2 Salmonella enterica subsp. enterica serovar Enteritidis str. SA20095309 genome	bacteria'
CP007319.2 Salmonella enterica subsp. enterica serovar Enteritidis str. SA20095440, complete genome	bacteria'
CP007323.2 Salmonella enterica subsp. enterica serovar Enteritidis str. EC20110222 genome	bacteria'
CP007324.2 Salmonella enterica subsp. enterica serovar Enteritidis str. EC20111514 genome	bacteria'
CP007325.2 Salmonella enterica subsp. enterica serovar Enteritidis str. EC20111515 genome	bacteria'
CP007326.2 Salmonella enterica subsp. enterica serovar Enteritidis str. EC20111554 genome	bacteria'
CP007327.2 Salmonella enterica subsp. enterica serovar Enteritidis str. EC20111561 genome	bacteria'
CP007328.2 Salmonella enterica subsp. enterica serovar Enteritidis str. EC20111576 genome	bacteria'
CP007330.2 Salmonella enterica subsp. enterica serovar Enteritidis str. EC20120003 genome	bacteria'
CP007331.2 Salmonella enterica subsp. enterica serovar Enteritidis str. EC20120007 genome	bacteria'
CP007344.2 Salmonella enterica subsp. enterica serovar Enteritidis str. EC20120213 genome	bacteria'
CP007345.2 Salmonella enterica subsp. enterica serovar Enteritidis str. EC20120219 genome	bacteria'
CP007346.2 Salmonella enterica subsp. enterica serovar Enteritidis str. EC20120229 genome	bacteria'
CP007347.2 Salmonella enterica subsp. enterica serovar Enteritidis str. EC20120240 genome	bacteria'
CP007348.2 Salmonella enterica subsp. enterica serovar Enteritidis str. EC20120356 genome	bacteria'
CP007349.2 Salmonella enterica subsp. enterica serovar Enteritidis str. EC20120469 genome	bacteria'
CP007350.2 Salmonella enterica subsp. enterica serovar Enteritidis str. EC20120496 genome	bacteria'
CP007351.2 Salmonella enterica subsp. enterica serovar Enteritidis str. EC20120497 genome	bacteria'
CP007352.2 Salmonella enterica subsp. enterica serovar Enteritidis str. EC20120498 genome	bacteria'
CP007353.2 Salmonella enterica subsp. enterica serovar Enteritidis str. EC20120505 genome	bacteria'
CP007354.2 Salmonella enterica subsp. enterica serovar Enteritidis str. EC20120528 genome	bacteria'
CP007365.2 Salmonella enterica subsp. enterica serovar Enteritidis str. EC20120994 genome	bacteria'
CP007366.2 Salmonella enterica subsp. enterica serovar Enteritidis str. EC20121004 genome	bacteria'
CP007367.2 Salmonella enterica subsp. enterica serovar Enteritidis str. EC20121541 genome	bacteria'
CP007368.2 Salmonella enterica subsp. enterica serovar Enteritidis str. EC20121542 genome	bacteria'
CP007369.2 Salmonella enterica subsp. enterica serovar Enteritidis str. EC20121671 genome	bacteria'
CP007370.2 Salmonella enterica subsp. enterica serovar Enteritidis str. EC20121672 genome	bacteria'
CP007371.2 Salmonella enterica subsp. enterica serovar Enteritidis str. EC20121689 genome	bacteria'
CP007372.2 Salmonella enterica subsp. enterica serovar Enteritidis str. SA20121703 genome	bacteria'
CP007373.2 Salmonella enterica subsp. enterica serovar Enteritidis str. EC20121744 genome	bacteria'
CP007374.2 Salmonella enterica subsp. enterica serovar Enteritidis str. EC20121746 genome	bacteria'
CP007395.2 Salmonella enterica subsp. enterica serovar Enteritidis str. EC20121748 genome	bacteria'
CP007396.2 Salmonella enterica subsp. enterica serovar Enteritidis str. EC20121750 genome	bacteria'
CP007397.2 Salmonella enterica subsp. enterica serovar Enteritidis str. EC20121751 genome	bacteria'
CP007398.2 Salmonella enterica subsp. enterica serovar Enteritidis str. EC20121753 genome	bacteria'
CP007400.2 Salmonella enterica subsp. enterica serovar Enteritidis str. EC20120734 genome	bacteria'
CP007401.2 Salmonella enterica subsp. enterica serovar Enteritidis str. EC20120738 genome	bacteria'
CP007402.2 Salmonella enterica subsp. enterica serovar Enteritidis str. EC20120765 genome	bacteria'
CP007403.2 Salmonella enterica subsp. enterica serovar Enteritidis str. EC20120773 genome	bacteria'
CP007404.2 Salmonella enterica subsp. enterica serovar Enteritidis str. EC20120774 genome	bacteria'
CP007405.2 Salmonella enterica subsp. enterica serovar Enteritidis str. EC20120775 genome	bacteria'
CP007406.2 Salmonella enterica subsp. enterica serovar Enteritidis str. EC20120776 genome	bacteria'
CP007407.2 Salmonella enterica subsp. enterica serovar Enteritidis str. EC20120917 genome	bacteria'
CP007408.2 Salmonella enterica subsp. enterica serovar Enteritidis str. EC20120918 genome	bacteria'
CP007375.2 Salmonella enterica subsp. enterica serovar Enteritidis str. EC20120925 genome	bacteria'
CP007376.2 Salmonella enterica subsp. enterica serovar Enteritidis str. EC20120927 genome	bacteria'
CP007377.2 Salmonella enterica subsp. enterica serovar Enteritidis str. EC20120963 genome	bacteria'
CP007378.2 Salmonella enterica subsp. enterica serovar Enteritidis str. EC20120968 genome	bacteria'
CP007379.2 Salmonella enterica subsp. enterica serovar Enteritidis str. EC20120969 genome	bacteria'
CP007380.2 Salmonella enterica subsp. enterica serovar Enteritidis str. EC20120970 genome	bacteria'
CP007381.2 Salmonella enterica subsp. enterica serovar Enteritidis str. EC20121812 genome	bacteria'
CP007382.2 Salmonella enterica subsp. enterica serovar Enteritidis str. EC20121825 genome	bacteria'
CP007383.2 Salmonella enterica subsp. enterica serovar Enteritidis str. EC20121826 genome	bacteria'
CP007384.2 Salmonella enterica subsp. enterica serovar Enteritidis str. EC20121969 genome	bacteria'
CP007385.2 Salmonella enterica subsp. enterica serovar Enteritidis str. EC20121970 genome	bacteria'
CP007296.2 Salmonella enterica subsp. enterica serovar Enteritidis str. SA20090419 genome	bacteria'
CP007297.2 Salmonella enterica subsp. enterica serovar Enteritidis str. SA20090435 genome	bacteria'
CP007298.2 Salmonella enterica subsp. enterica serovar Enteritidis str. EC20090530 genome	bacteria'
CP007355.2 Salmonella enterica subsp. enterica serovar Enteritidis str. EC20100088 genome	bacteria'
CP007356.2 Salmonella enterica subsp. enterica serovar Enteritidis str. EC20100089 genome	bacteria'
CP007335.2 Salmonella enterica subsp. enterica serovar Enteritidis str. EC20120580 genome	bacteria'

CP007336.2 Salmonella enterica subsp. enterica serovar Enteritidis str. EC20120581 genome	bacteria'
CP007337.2 Salmonella enterica subsp. enterica serovar Enteritidis str. EC20120590 genome	bacteria'
CP007338.2 Salmonella enterica subsp. enterica serovar Enteritidis str. EC20120597 genome	bacteria'
CP007339.2 Salmonella enterica subsp. enterica serovar Enteritidis str. EC20120685 genome	bacteria'
CP007340.2 Salmonella enterica subsp. enterica serovar Enteritidis str. EC20120686 genome	bacteria'
CP007341.2 Salmonella enterica subsp. enterica serovar Enteritidis str. EC20120687 genome	bacteria'
CP007342.2 Salmonella enterica subsp. enterica serovar Enteritidis str. EC20120697 genome	bacteria'
CP007343.2 Salmonella enterica subsp. enterica serovar Enteritidis str. EC20120722 genome	bacteria'
CP007386.2 Salmonella enterica subsp. enterica serovar Enteritidis str. EC20121976 genome	bacteria'
CP007426.2 Salmonella enterica subsp. enterica serovar Enteritidis str. SA20085285, complete genome	bacteria'
CP007430.2 Salmonella enterica subsp. enterica serovar Enteritidis str. EC20090195 genome	bacteria'
CP007431.2 Salmonella enterica subsp. enterica serovar Enteritidis str. SA20094682 genome	bacteria'
CP007432.2 Salmonella enterica subsp. enterica serovar Enteritidis str. EC20100131 genome	bacteria'
CP007427.2 Salmonella enterica subsp. enterica serovar Enteritidis str. SA20100239 genome	bacteria'
CP007428.2 Salmonella enterica subsp. enterica serovar Enteritidis str. EC20120677 genome	bacteria'
CP007429.2 Salmonella enterica subsp. enterica serovar Enteritidis str. EC20121765 genome	bacteria'
CP007387.2 Salmonella enterica subsp. enterica serovar Enteritidis str. EC20121986 genome	bacteria'
CP007388.2 Salmonella enterica subsp. enterica serovar Enteritidis str. EC20121989 genome	bacteria'
CP007433.2 Salmonella enterica subsp. enterica serovar Enteritidis str. EC20120051 genome	bacteria'
CP007411.2 Salmonella enterica subsp. enterica serovar Enteritidis str. EC20121990 genome	bacteria'
CP007412.2 Salmonella enterica subsp. enterica serovar Enteritidis str. EC20122022 genome	bacteria'
CP007413.2 Salmonella enterica subsp. enterica serovar Enteritidis str. EC20122026 genome	bacteria'
CP007414.2 Salmonella enterica subsp. enterica serovar Enteritidis str. EC20122031 genome	bacteria'
CP007415.2 Salmonella enterica subsp. enterica serovar Enteritidis str. EC20122033 genome	bacteria'
CP007416.2 Salmonella enterica subsp. enterica serovar Enteritidis str. EC20122045 genome	bacteria'
CP007417.2 Salmonella enterica subsp. enterica serovar Enteritidis str. SA20123395 genome	bacteria'
CP007418.2 Salmonella enterica subsp. enterica serovar Enteritidis str. EC20130345 genome	bacteria'
CP007419.2 Salmonella enterica subsp. enterica serovar Enteritidis str. EC20130346 genome	bacteria'
CP007423.2 Salmonella enterica subsp. enterica serovar Enteritidis str. EC20130347 genome	bacteria'
CP007424.2 Salmonella enterica subsp. enterica serovar Enteritidis str. EC20130348 genome	bacteria'
CP007420.2 Salmonella enterica subsp. enterica serovar Enteritidis str. EC20100103 genome	bacteria'
CP007421.2 Salmonella enterica subsp. enterica serovar Enteritidis str. EC20090884 genome	bacteria'
CP007422.2 Salmonella enterica subsp. enterica serovar Enteritidis str. EC20090531 genome	bacteria'
CP007438.2 Salmonella enterica subsp. enterica serovar Enteritidis str. EC20120009 genome	bacteria'
CP007463.2 Salmonella enterica subsp. enterica serovar Enteritidis str. EC20120929 genome	bacteria'
CP007464.2 Salmonella enterica subsp. enterica serovar Enteritidis str. EC20121747 genome	bacteria'
CP007465.1 Salmonella enterica subsp. enterica serovar Enteritidis str. SA19940857 genome	bacteria'
CP007466.2 Salmonella enterica subsp. enterica serovar Enteritidis str. SA20084644 genome	bacteria'
CP007467.2 Salmonella enterica subsp. enterica serovar Enteritidis str. SA20084824 genome	bacteria'
CP007468.2 Salmonella enterica subsp. enterica serovar Enteritidis str. SA20094177 genome	bacteria'
CP007469.2 Salmonella enterica subsp. enterica serovar Enteritidis str. SA20094301 genome	bacteria'
CP007498.2 Salmonella enterica subsp. enterica serovar Enteritidis str. EC20111510 genome	bacteria'
CP007224.1 Pseudomonas aeruginosa PA96 genome	bacteria'
CM002692.1 Geobacillus stearothermophilus NUB3621 isolate 9A5 chromosome, whole genome shotgun sequence	bacteria'
CM002694.1 Oryza minuta chromosome CC3 short arm, whole genome shotgun sequence	plants'
KK214010.1 Aspergillus niger SH-2 unplaced genomic scaffold contig1, whole genome shotgun sequence	fungi'
CM002705.1 Flammulina velutipes KACC42780 chromosome 11, whole genome shotgun sequence	fungi'
CM002707.1 Poecilia reticulata strain Guanapo linkage group LG2, whole genome shotgun sequence	vertebrates_non_mammals'
KK214768.1 Candida tropicalis strain 121 unplaced genomic scaffold Scaffold1, whole genome shotgun sequence	fungi'
CM002739.1 Camelina sativa cultivar DH55 chromosome 11, whole genome shotgun sequence	plants'
CP007439.1 Serratia plymuthica strain V4 genome	bacteria'
KK241008.1 Copidosoma floridanum isolate CFLO.00-Male unplaced genomic scaffold Scaffold3, whole genome shotgun sequence	invertebrates'
KK244445.1 Cimex lectularius isolate CLEC.00 strain Harlan unplaced genomic scaffold Scaffold1, whole genome shotgun sequence	invertebrates'
KK245170.1 Onthophagus taurus breed undomesticated insect unplaced genomic scaffold Scaffold5, whole genome shotgun sequence	invertebrates'
KK249926.1 Limnephilus lunatus isolate LLUN.00-Female unplaced genomic scaffold Scaffold50, whole genome shotgun sequence	invertebrates'
CM002753.1 Pseudomonas syringae pv. actinidiae ICMP 9617 chromosome Chr, whole genome shotgun sequence	bacteria'
AZMS01S00000178.1 Acanthoscurria geniculata scaffold178, whole genome shotgun sequence	invertebrates'
CP007569.1 Bradyrhizobium japonicum SEMIA 5079 genome	bacteria'
CP007597.1 Stenotrophomonas rhizophila strain DSM14405 genome	bacteria'
CM002755.1 Ralstonia solanacearum Rs-10-244 chromosome, whole genome shotgun sequence	bacteria'
CM002757.1 Ralstonia solanacearum Rs-09-161 chromosome, whole genome shotgun sequence	bacteria'
AXCF01000091.1 Diplodia sapinea CMW 190 strain CMW190 contig_96, whole genome shotgun sequence	fungi'
KK342514.1 Centruroides exilicauda isolate CEXI.00-Female breed non domesticated species of scorpion unplaced genomic scaffold Scaffold1, whole genome shotgun sequence	invertebrates'
KK351785.1 Glossina fuscipes fuscipes unplaced genomic scaffold Scaffold0, whole genome shotgun sequence	invertebrates'
KK351011.1 Glossina brevipalpis unplaced genomic scaffold Scaffold0, whole genome shotgun sequence	invertebrates'
JHVZ03000002.1 Phytophthora fragariae strain 309.62 scaffold2, whole genome shotgun sequence	protozoa'
CM002772.1 Brassica napus cultivar ZS11 chromosome C4, whole genome shotgun sequence	plants'
KK502053.1 Egretta garzetta isolate BGI_Z169 unplaced genomic scaffold scaffold287, whole genome shotgun sequence	vertebrates_non_mammals'
KK382893.1 Tyto alba isolate BGI_N341 unplaced genomic scaffold scaffold1014, whole genome shotgun sequence	vertebrates_non_mammals'
KK498653.1 Ursus maritimus isolate Baiyulong unplaced genomic scaffold scaffold1, whole genome shotgun sequence	vertebrates_mammals'
KK409426.1 Phoenicopterus ruber ruber isolate BGI_N337 unplaced genomic scaffold scaffold8371, whole genome shotgun sequence	vertebrates_non_mammals'
KK448873.1 Phaethon lepturus isolate BGI_N335 unplaced genomic scaffold scaffold1483, whole genome shotgun sequence	vertebrates_non_mammals'
KK494852.1 Pelecanus crispus isolate BGI_N334 unplaced genomic scaffold scaffold1068, whole genome shotgun sequence	vertebrates_non_mammals'
JHTR01000001.1 Ustilaginoidea virens strain UV-8b Sca_1, whole genome shotgun sequence	fungi'
JARK01001337.1 Ancylostoma ceylanicum strain HY135 Acey_s0001_scaf, whole genome shotgun sequence	invertebrates'
AVPZ01000001.1 Cavia aperea scaffold0, whole genome shotgun sequence	vertebrates_mammals'
KK499764.1 Glossina pallidipes unplaced genomic scaffold Scaffold0, whole genome shotgun sequence	invertebrates'
KK502400.1 Glossina austeni unplaced genomic scaffold Scaffold0, whole genome shotgun sequence	invertebrates'
HG916851.1 Dicentrarchus labrax genomic scaffold, UN, whole genome shotgun sequence	vertebrates_non_mammals'
HG966617.1 Candidatus Phaeomarinobacter ectocarpi complete genome	bacteria'
KK528063.1 Cariama cristata isolate BGI_N322 unplaced genomic scaffold scaffold3759, whole genome shotgun sequence	vertebrates_non_mammals'
KK551537.1 Colius striatus isolate BGI_N325 unplaced genomic scaffold scaffold7429, whole genome shotgun sequence	vertebrates_non_mammals'
KK580071.1 Stegastes partitus isolate 25-593 unplaced genomic scaffold Scaffold0, whole genome shotgun sequence	vertebrates_non_mammals'
KK570360.1 Eurypyga helias isolate BGI_N326 unplaced genomic scaffold scaffold5746, whole genome shotgun sequence	vertebrates_non_mammals'
KK599561.1 Fulmarus glacialis isolate BGI_N327 unplaced genomic scaffold scaffold2225, whole genome shotgun sequence	vertebrates_non_mammals'
KK617602.1 Gavia stellata isolate BGI_N328 unplaced genomic scaffold scaffold6952, whole genome shotgun sequence	vertebrates_non_mammals'
JMRU01000001.1 Trypanosoma grayi strain ANR4 Tgr_1_V1, whole genome shotgun sequence	protozoa'
KK652047.1 Haliaeetus albicilla isolate BGI_N329 unplaced genomic scaffold scaffold3684, whole genome shotgun sequence	vertebrates_non_mammals'
KK667223.1 Candida albicans Ca529L unplaced genomic scaffold supercont1.1, whole genome shotgun sequence	fungi'
KK669244.1 Leptosomus discolor isolate BGI_N330 unplaced genomic scaffold scaffold1120, whole genome shotgun sequence	vertebrates_non_mammals'
KK704440.1 Merops nubicus isolate BGI_N331 unplaced genomic scaffold scaffold195, whole genome shotgun sequence	vertebrates_non_mammals'
FO082874.1 Babesia microti strain RI chromosome III, complete sequence	protozoa'
KK719449.1 Corvus brachyrhynchos isolate BGI_N302 unplaced genomic scaffold scaffold115, whole genome shotgun sequence	vertebrates_non_mammals'
KK726493.1 Schmidtea mediterranea strain s2f2 unplaced genomic scaffold scaffold8598, whole genome shotgun sequence	invertebrates'
KL671375.1 Manacus vitellinus isolate BGI_N305 unplaced genomic scaffold scaffold11, whole genome shotgun sequence	vertebrates_non_mammals'
KK735119.1 Opisthocomus hoazin isolate BGI_N306 unplaced genomic scaffold scaffold164, whole genome shotgun sequence	vertebrates_non_mammals'
AZBK01S000001.1 Anguilla anguilla scaffold_1, whole genome shotgun sequence	vertebrates_non_mammals'
KK757047.1 Chlamydotis macqueenii isolate BGI_N324 unplaced genomic scaffold scaffold10934, whole genome shotgun sequence	vertebrates_non_mammals'
AVPN01000006.1 Pleurobrachia bachei scaffold1.1, whole genome shotgun sequence	invertebrates'
JHQJ01000001.1 Bactrocera tryoni strain bent wings Btry1, whole genome shotgun sequence	invertebrates'
KK764649.1 Rhizoctonia solani AG-8 WAC10335 unplaced genomic scaffold Scaffold_1, whole genome shotgun sequence	fungi'
CM002786.1 Brassica oleracea var. oleracea cultivar TO1000 chromosome C3, whole genome shotgun sequence	plants'
KK784873.1 Citrus sinensis cultivar Ridge Pineapple sweet orange unplaced genomic scaffold scaffold00001, whole genome shotgun sequence	plants'
KK765506.1 Pachypsylla venusta isolate PVEN.00 unplaced genomic scaffold Scaffold1, whole genome shotgun sequence	invertebrates'
KK811377.1 Mesitornis unicolor isolate BGI_N332 unplaced genomic scaffold scaffold2165, whole genome shotgun sequence	vertebrates_non_mammals'
KK839031.1 Acanthisitta chloris isolate BGI_N310 unplaced genomic scaffold scaffold1876, whole genome shotgun sequence	vertebrates_non_mammals'
KK848034.1 Aquila chrysaetos canadensis unplaced genomic scaffold scaffoldkmer70-min200.2284220, whole genome shotgun sequence	vertebrates_non_mammals'
JMSE01000001.1 Colletotrichum sublineola strain TX430BB Scaffolds0001.1, whole genome shotgun sequence	fungi'
KK852463.1 Zootermopsis nevadensis unplaced genomic scaffold scaffold24, whole genome shotgun sequence	invertebrates'
KK854002.1 Oncopeltus fasciatus isolate OFAS.00 unplaced genomic scaffold Scaffold1, whole genome shotgun sequence	invertebrates'
KL142781.1 Galeopterus variegatus unplaced genomic scaffold Scaffold0, whole genome shotgun sequence	vertebrates_mammals'
KK898996.1 Aquilaria agallochum cultivar A.agall-S2m unplaced genomic scaffold scaffold2, whole genome shotgun sequence	plants'
KK915662.1 Jatropha curcas cultivar GZQX0401 unplaced genomic scaffold scaffold684, whole genome shotgun sequence	plants'
KK998761.1 Equus caballus breed Mongolian isolate Ajinai unplaced genomic scaffold scaffold1, whole genome shotgun sequence	vertebrates_mammals'
KK949850.1 Equus przewalskii isolate Burgud unplaced genomic scaffold scaffold1, whole genome shotgun sequence	vertebrates_mammals'
KK920213.1 Halyomorpha halys isolate HHAL.00 unplaced genomic scaffold Scaffold4, whole genome shotgun sequence	invertebrates'
KK961494.1 Homalodisca vitripennis isolate HVIT.00 unplaced genomic scaffold Scaffold1, whole genome shotgun sequence	invertebrates'
KK948332.1 Nestor notabilis isolate BGI_N333 unplaced genomic scaffold scaffold4209, whole genome shotgun sequence	vertebrates_non_mammals'
KK960494.1 Rhizopus oryzae HUMC 02 unplaced genomic scaffold NODE_5514_length_540393_cov_12.073347, whole genome shotgun sequence	fungi'
KL142367.1 Galerina marginata CBS 339.88 unplaced genomic scaffold GALMAscaffold_1, whole genome shotgun sequence	fungi'
KL197709.1 Jaapia argillacea MUCL 33604 unplaced genomic scaffold JAAARscaffold_1, whole genome shotgun sequence	fungi'
KL198004.1 Pleurotus ostreatus PC15 unplaced genomic scaffold scaffold_01, whole genome shotgun sequence	fungi'
KL198016.1 Botryobasidium botryosum FD-172 SS1 unplaced genomic scaffold BOTBOscaffold_1, whole genome shotgun sequence	fungi'
KK998513.1 Rhizopus oryzae 99-892, whole genome shotgun sequence	fungi'
KL027845.1 Latrodectus hesperus isolate LHES.00 unplaced genomic scaffold Scaffold1, whole genome shotgun sequence	invertebrates'
KL023694.1 Frankliniella occidentalis isolate FOCC.00 unplaced genomic scaffold Scaffold1, whole genome shotgun sequence	invertebrates'
JHEK01291953.1 Pampus argenteus breed Wild-type isolate Kuwaiti scaffold291955, whole genome shotgun sequence	vertebrates_non_mammals'
CP007637.1 Pseudomonas simiae strain WCS417 genome	bacteria'
CP007638.1 Pseudomonas sp. WCS374 genome	bacteria'
KL206666.1 Struthio camelus australis isolate BGI_N308 unplaced genomic scaffold scaffold155, whole genome shotgun sequence	vertebrates_non_mammals'
KL215552.1 Picoides pubescens isolate BGI_N307 unplaced genomic scaffold scaffold283, whole genome shotgun sequence	vertebrates_non_mammals'
KL218800.1 Agrilus planipennis isolate EAB-ADULT unplaced genomic scaffold Scaffold3, whole genome shotgun sequence	invertebrates'
KL221432.1 Lucilia cuprina strain LS unplaced genomic scaffold Scaffold23, whole genome shotgun sequence	invertebrates'
KL217905.1 Calypte anna isolate BGI_N300 unplaced genomic scaffold scaffold1, whole genome shotgun sequence	vertebrates_non_mammals'
KL225530.1 Pygoscelis adeliae isolate BGI_AS28 unplaced genomic scaffold Scaffold38, whole genome shotgun sequence	vertebrates_non_mammals'
KL226002.1 Aptenodytes forsteri isolate BGI_AS27 unplaced genomic scaffold Scaffold15, whole genome shotgun sequence	vertebrates_non_mammals'
KL230200.1 Pterocles gutturalis isolate BGI_N339 unplaced genomic scaffold scaffold2666, whole genome shotgun sequence	vertebrates_non_mammals'
KL250487.1 Schistosoma haematobium, whole genome shotgun sequence	invertebrates'
KL274534.1 Podiceps cristatus isolate BGI_N338 unplaced genomic scaffold scaffold1533, whole genome shotgun sequence	vertebrates_non_mammals'
KL314543.1 Cathartes aura isolate BGI_N323 unplaced genomic scaffold scaffold5665, whole genome shotgun sequence	vertebrates_non_mammals'
CM002797.1 Oryza rufipogon chromosome 3, whole genome shotgun sequence	plants'
KL361834.1 Caprimulgus carolinensis isolate BGI_N321 unplaced genomic scaffold scaffold7979, whole genome shotgun sequence	vertebrates_non_mammals'
JNCD01006054.1 Pimephales promelas scaffold6903, whole genome shotgun sequence	vertebrates_non_mammals'
JNCE01299955.1 Pimephales promelas scaffold-299978, whole genome shotgun sequence	vertebrates_non_mammals'
KL363182.1 Trichuris suis isolate DCEP-RM93M unplaced genomic scaffold scaffold42, whole genome shotgun sequence	invertebrates'

KL367474.1 Trichuris suis isolate DCEP-RM93F unplaced genomic scaffold scaffold62, whole genome shotgun sequence	invertebrates'
KL371215.1 Apaloderma vittatum isolate BGI_N311 unplaced genomic scaffold scaffold12370, whole genome shotgun sequence	vertebrates_non_mammals'
KL886562.1 Tinamus guttatus isolate BGI_N309 unplaced genomic scaffold scaffold110, whole genome shotgun sequence	vertebrates_non_mammals'
KL871737.1 Charadrius vociferus isolate BGI_N301 unplaced genomic scaffold scaffold163, whole genome shotgun sequence	vertebrates_non_mammals'
KL411542.1 Mixia osmundae IAM 14324 unplaced genomic scaffold scaffold_1, whole genome shotgun sequence	fungi'
KL411536.1 Nipponia nippon isolate BGI_Y956 unplaced genomic scaffold Scaffold108, whole genome shotgun sequence	vertebrates_non_mammals'
DF236950.1 Klebsormidium flaccidum DNA, scaffold: kfl00001, whole genome shotgun sequence	plants'
KL430423.1 Phalacrocorax carbo isolate BGI_N336 unplaced genomic scaffold scaffold2215, whole genome shotgun sequence	vertebrates_non_mammals'
KL446944.1 Plasmodium vinckei vinckei unplaced genomic scaffold supercont1.1, whole genome shotgun sequence	protozoa'
AMGV01000001.1 Exophiala aquamarina CBS 119918 aczIA-supercont1.1, whole genome shotgun sequence	fungi'
KL448103.1 Cuculus canorus isolate BGI_N303 unplaced genomic scaffold scaffold15, whole genome shotgun sequence	vertebrates_non_mammals'
KL449087.1 Tauraco erythrolophus isolate BGI_N340 unplaced genomic scaffold scaffold830, whole genome shotgun sequence	vertebrates_non_mammals'
CP006581.1 Mesorhizobium huakuii 7653R genome	bacteria'
KL487749.1 Balearica regulorum gibbericeps isolate BGI_N312 unplaced genomic scaffold scaffold2944, whole genome shotgun sequence	vertebrates_non_mammals'
CM002798.1 Penicillium chrysogenum strain P2niaD18 chromosome I, whole genome shotgun sequence	fungi'
KL509103.1 Buceros rhinoceros silvestris isolate BGI_N320 unplaced genomic scaffold scaffold1641, whole genome shotgun sequence	vertebrates_non_mammals'
AZAH01000001.1 Eremothecium coryli CBS 5749 scaffold_0, whole genome shotgun sequence	fungi'
CM002803.1 Planktothrix agardhii NIVA-CYA 126/8 chromosome, whole genome shotgun sequence	bacteria'
CM002809.1 Oryza punctata chromosome 3, whole genome shotgun sequence	plants'
CM002811.2 Oryza nivara chromosome 3, whole genome shotgun sequence	plants'
CM002810.1 Oryza brachyantha chromosome 3, whole genome shotgun sequence	plants'
CM002812.1 Capsicum annuum cultivar Zunla-1 chromosome 1, whole genome shotgun sequence	plants'
JJRZ01000001.1 Ceratocystis manginecans strain CMW17570 scaffold1, whole genome shotgun sequence	fungi'
JMSH01000001.1 Huntiella moniliformis strain CBS 118127 CMon001, whole genome shotgun sequence	fungi'
CP008749.1 Pseudomonas aeruginosa PAO1H2O genome	bacteria'
AWOJ01S027074.1 Nicotiana tabacum cultivar K326 Ntab-K326_scaffold27074, whole genome shotgun sequence	plants'
AWOK01S000758.1 Nicotiana tabacum cultivar Basma Xanthi Ntab-BX_scaffold758, whole genome shotgun sequence	plants'
AWOL01S0001529.1 Nicotiana otophora Noto_scaffold1529, whole genome shotgun sequence	plants'
AYMY01S002778.1 Nicotiana tabacum cultivar TN90 Ntab-TN90_scaffold2778, whole genome shotgun sequence	plants'
KL567389.1 Gyrodactylus salaris strain Lier unplaced genomic scaffold scf7180006953439, whole genome shotgun sequence	invertebrates'
JMSD01000001.1 Paramecium caudatum strain 43c3d scaffold_0001, whole genome shotgun sequence	protozoa'
KL596619.1 Opisthorchis viverrini unplaced genomic scaffold opera_v5_1, whole genome shotgun sequence	invertebrates'
DF384229.1 Raphanus sativus DNA, scaffold: Rsa1.0_00016.1, whole genome shotgun sequence	plants'
KL580752.1 Melitaea cinxia unplaced genomic scaffold scaffold1689, whole genome shotgun sequence	invertebrates'
CM002825.1 Oryza officinalis chromosome 3, whole genome shotgun sequence	plants'
KL584749.1 Aureobasidium subglaciale EXF-2481 unplaced genomic scaffold scaffold_0, whole genome shotgun sequence	fungi'
KL584702.1 Aureobasidium namibiae CBS 147.97 unplaced genomic scaffold scaffold_1, whole genome shotgun sequence	fungi'
KL584824.1 Aureobasidium pullulans var. melanogenum CBS 110374 unplaced genomic scaffold scaffold_1, whole genome shotgun sequence	fungi'
KL584974.1 Aureobasidium pullulans var. pullulans EXF-150 unplaced genomic scaffold scaffold_1, whole genome shotgun sequence	fungi'
CM002832.3 Esox lucius isolate CL-BC-CA-002 linkage group LG07, whole genome shotgun sequence	vertebrates_non_mammals'
KN671115.1 Glycine soja cultivar W05 unplaced genomic scaffold scaffold1660, whole genome shotgun sequence	plants'
HG315671.1 Formosa agariphila KMM 3901, complete genome	bacteria'
CBTN010000001.1 Lichtheimia corymbifera JMRC:FSU:9682 WGS project CBTN000000000 data, contig SCAF00001, whole genome shotgun sequence	fungi'
HG810775.1 Plasmodium reichenowi CDC genomic scaffold, PrCDC_14_v3, whole genome shotgun sequence	protozoa'
HG820002.1 Globodera pallida Lindley genomic scaffold, pathogens_Gpal_scaffold_1, whole genome shotgun sequence	invertebrates'
CM003871.1 Oryza sativa Indica Group cultivar HR-12 chromosome 1, whole genome shotgun sequence	plants'
CM002865.1 Plasmodium coatneyi strain Hackeri chromosome 14, whole genome shotgun sequence	protozoa'
KL647288.1 Sarcocystis neurona strain SN3 unplaced genomic scaffold scaffold00001, whole genome shotgun sequence	protozoa'
JHUM01000033.1 Diplodia sapinea strain CMW39103 Contig_33, whole genome shotgun sequence	fungi'
CM002866.1 Xanthomonas axonopodis pv. allii CFBP6369 chromosome, whole genome shotgun sequence	bacteria'
KL648097.1 Stachybotrys chartarum IBT 7711 unplaced genomic scaffold scaffold727, whole genome shotgun sequence	fungi'
JHOI01000634.1 Aspergillus fumigatus var. RP-2014 strain niveus afvn_670, whole genome shotgun sequence	fungi'
JOWA01000110.1 Scedosporium apiospermum strain IHEM 14462 SEQ_SAPIO_0110, whole genome shotgun sequence	fungi'
JPET01000001.1 Verticillium tricorpus MUCL 9792 Scaffold01, whole genome shotgun sequence	fungi'
CP007023.1 Streptococcus pyogenes STAB1102 genome	bacteria'
KL652500.1 Cyprinodon variegatus isolate N-32 unplaced genomic scaffold Scaffold0, whole genome shotgun sequence	vertebrates_non_mammals'
KL650235.1 Stachybotrys chartarum IBT 40293 unplaced genomic scaffold scaffold82, whole genome shotgun sequence	fungi'
KL657812.1 Stachybotrys chartarum IBT 40288 unplaced genomic scaffold scaffold1018, whole genome shotgun sequence	fungi'
KL659601.1 Stachybotrys chlorohalonata IBT 40285 unplaced genomic scaffold scaffold505, whole genome shotgun sequence	fungi'
CM002876.1 Arabis alpina cultivar Pajares chromosome 8, whole genome shotgun sequence	plants'
KL662111.1 Auxenochlorella protothecoides strain 0710 unplaced genomic scaffold scaffold18, whole genome shotgun sequence	plants'
CM002964.1 Saccharomyces cerevisiae UFMG A-905 chromosome IV, whole genome shotgun sequence	fungi'
JNVV01000001.1 Falciphora oryzae strain R5-6-1 scaffold1, whole genome shotgun sequence	fungi'
JPFM01000001.1 Paramecium sexaurelia strain CA1 scaffold_001, whole genome shotgun sequence	protozoa'
JPFL01000001.1 Paramecium biaurelia strain V1-4 scaffold_0001, whole genome shotgun sequence	protozoa'
CM002954.1 Saccharomyces sp. 'boulardii' strain 17 chromosome IV, whole genome shotgun sequence	fungi'
KL668297.1 Notothenia coriiceps isolate Sejong01 unplaced genomic scaffold NC01_scaffold00037246, whole genome shotgun sequence	vertebrates_non_mammals'
CP008959.1 Mycobacterium tuberculosis strain 6A024XDR genome	bacteria'
CP008960.1 Mycobacterium tuberculosis strain 0B329XDR genome	bacteria'
CP008961.1 Mycobacterium tuberculosis strain 0B259XDR genome	bacteria'
CP008962.1 Mycobacterium tuberculosis strain 0B235DS genome	bacteria'
CP008963.1 Mycobacterium tuberculosis strain 0B229DS genome	bacteria'
CP008964.1 Mycobacterium tuberculosis strain 0B228DS genome	bacteria'
CP008965.1 Mycobacterium tuberculosis strain 0B222DS genome	bacteria'
CP008966.1 Mycobacterium tuberculosis strain 0B218DS genome	bacteria'
CP008967.1 Mycobacterium tuberculosis strain 0B169XDR genome	bacteria'
CP008968.1 Mycobacterium tuberculosis strain 0B123ND genome	bacteria'
CP008969.1 Mycobacterium tuberculosis strain 0B076XDR genome	bacteria'
CP008970.1 Mycobacterium tuberculosis strain 0B070XDR genome	bacteria'
CP008971.1 Mycobacterium tuberculosis strain 0B049XDR genome	bacteria'
CP008972.1 Mycobacterium tuberculosis strain 0B026XDR genome	bacteria'
CP008973.1 Mycobacterium tuberculosis strain 0A117DS genome	bacteria'
CP008974.1 Mycobacterium tuberculosis strain 0A115DS genome	bacteria'
CP008975.1 Mycobacterium tuberculosis strain 0A094DS genome	bacteria'
CP008976.1 Mycobacterium tuberculosis strain 0A093DS genome	bacteria'
CP008977.1 Mycobacterium tuberculosis strain 0A092DS genome	bacteria'
CP008978.1 Mycobacterium tuberculosis strain 0A087DS genome	bacteria'
CP008979.1 Mycobacterium tuberculosis strain 0A036DS genome	bacteria'
CP008980.1 Mycobacterium tuberculosis strain 0A033DS genome	bacteria'
CP008981.1 Mycobacterium tuberculosis strain 0A029DS genome	bacteria'
CP008982.1 Mycobacterium tuberculosis strain 0A012DS genome	bacteria'
CP008983.1 Mycobacterium tuberculosis strain 0A005DS genome	bacteria'
CP008947.1 Rhodococcus opacus strain R7 sequence	bacteria'
KL772050.1 Manihot esculenta subsp. flabellifolia cultivar W14 unplaced genomic scaffold MW036205, whole genome shotgun sequence	plants'
KL808647.1 Manihot esculenta cultivar KU50 unplaced genomic scaffold MK060789, whole genome shotgun sequence	plants'
JPMF01003769.1 Crotalus mitchellii pyrrhus CMI_contig_3798, whole genome shotgun sequence	vertebrates_non_mammals'
KL869011.1 Haliaeetus leucocephalus isolate CR32 unplaced genomic scaffold Scaffold0, whole genome shotgun sequence	vertebrates_non_mammals'
KL996993.1 Rhizopus delemar Type II NRRL 21477 unplaced genomic scaffold NODE_10761_length_1104326_cov_9.435610, whole genome shotgun sequence	fungi'
KL995831.1 Rhizopus delemar Type II NRRL 21447 unplaced genomic scaffold NODE_1165_length_583167_cov_9.728719, whole genome shotgun sequence	fungi'
KL995122.1 Rhizopus delemar Type II NRRL 21446 unplaced genomic scaffold NODE_1768_length_589868_cov_9.067561, whole genome shotgun sequence	fungi'
JEMP01000025.1 Venturia pyrina ICMP 11032 scaffold1, whole genome shotgun sequence	fungi'
KL997524.1 Corvus cornix cornix isolate S_Up_H32 unplaced genomic scaffold scaffold_0, whole genome shotgun sequence	vertebrates_non_mammals'
JDSR01000322.1 Papiliotrema laurentii RY1 contig_334, whole genome shotgun sequence	fungi'
AKIJ01000001.1 Nematocida sp. 1 ERTm6 supercont2.1, whole genome shotgun sequence	fungi'
KN005617.1 Manis pentadactyla isolate MPE899 unplaced genomic scaffold Scaffold0, whole genome shotgun sequence	vertebrates_mammals'
KN042416.1 Mortierella verticillata NRRL 6337 unplaced genomic scaffold supercont1.1, whole genome shotgun sequence	fungi'
CP007756.1 Porphyromonas gingivalis strain HG66 genome	bacteria'
CP007789.1 Brucella melitensis bv. 2 str. 63/9 chromosome 1, complete sequence	bacteria'
AP011946.1 Theileria orientalis strain Shintoku DNA, chromosome 1, complete genome	protozoa'
CP007707.1 Brucella abortus strain 3196 chromosome 1 sequence	bacteria'
CM003510.1 Vigna radiata var. radiata cultivar VC1973A chromosome 7, whole genome shotgun sequence	plants'
CM002879.1 Bacillus anthracis strain Smith 1013 chromosome, whole genome shotgun sequence	bacteria'
CP007640.1 Bacillus atrophaeus subsp. globigii strain BSS genome	bacteria'
CP008752.1 Bacillus anthracis strain delta Sterne genome	bacteria'
CP007704.1 Bacillus anthracis strain BFV genome	bacteria'
KN046829.1 Larimichthys crocea isolate lyc2010zjou1 breed Donghai unplaced genomic scaffold scf2, whole genome shotgun sequence	vertebrates_non_mammals'
CP008838.1 Stenotrophomonas maltophilia strain 13637 genome	bacteria'
KN049970.1 Lignosus rhinocerotis TM02 unplaced genomic scaffold scaffold1, whole genome shotgun sequence	fungi'
KN050433.1 Curvularia lunata CX-3 unplaced genomic scaffold scaffold1, whole genome shotgun sequence	fungi'
KN122054.1 Fukomys damarensis unplaced genomic scaffold scaffold185, whole genome shotgun sequence	vertebrates_mammals'
JPUL02000001.1 Blastocystis sp. subtype 4 strain WR1 seqid_0001, whole genome shotgun sequence	protozoa'
CM002881.1 Bordetella bronchiseptica strain ATCC 10580 chromosome, whole genome shotgun sequence	bacteria'
CM002882.1 Mycobacterium tuberculosis H37RvAE chromosome, whole genome shotgun sequence	bacteria'
DF830068.1 Pseudozyma antarctica DNA, scaffold: scaffold_v10001, strain JCM 10317, whole genome shotgun sequence	fungi'
KN126570.1 Chaetura pelagica isolate M959 unplaced genomic scaffold scaffold90, whole genome shotgun sequence	vertebrates_non_mammals'
JPHH03000001.1 Tolypocladium sp. Sup5 PDA-1 contig_1, whole genome shotgun sequence	fungi'
JPOX01000001.1 Talaromyces marneffei PM1 scf001, whole genome shotgun sequence	fungi'
CP008955.1 Yersinia kristensenii strain ATCC 33639 genome	bacteria'
HG974234.1 Pseudomonas aeruginosa strain PSE305, genome	bacteria'
LK031787.1 Brassica napus genomic scaffold, scaffold_1, whole genome shotgun sequence	plants'
CCEW01000001.1 Phytophthora pisi genome assembly P. pisi assembly, contig contig_1, whole genome shotgun sequence	protozoa'
CCOE01000287.1 Amphilophus citrinellus genome assembly Midas_v5, contig scaffold-286, whole genome shotgun sequence	vertebrates_non_mammals'
CM002883.1 Mycobacterium tuberculosis H37RvLP chromosome, whole genome shotgun sequence	bacteria'
CCMK010016035.1 Capreolus capreolus genome assembly kmer631, contig DEER_K63_SCAFFOLD16035, whole genome shotgun sequence	vertebrates_mammals'
CM002884.1 Mycobacterium tuberculosis H37RvMA chromosome, whole genome shotgun sequence	bacteria'
CCDZ01000001.1 Meloidogyne floridensis genome assembly nMf_1_1, contig nMf_1_1_scaf00001, whole genome shotgun sequence	invertebrates'
JQCY02000003.1 Spodoptera frugiperda scaffold3, whole genome shotgun sequence	invertebrates'
KN127243.1 Amaranthus hypochondriacus isolate AhG2s unplaced genomic scaffold scaffold12, whole genome shotgun sequence	plants'
CM002913.1 Drosophila simulans strain w501 chromosome 3R, whole genome shotgun sequence	invertebrates'
KN265167.1 Bison bison bison isolate TAMUID 2011002044 unplaced genomic scaffold scf7180017864471, whole genome shotgun sequence	vertebrates_mammals'
CP009098.1 Brucella abortus strain ZW053 chromosome 1 sequence	bacteria'
CP009404.1 Leishmania panamensis strain MHOM/PA/94/PSC-1 chromosome 35 sequence	protozoa'
CP008764.1 Burkholderia pseudomallei strain MSHR346, complete sequence	bacteria'
CCSE01000001.1 Jeotgalicoccus sp. 13MG44_air genome assembly PRJEB5505_assembly_1, contig CONTIG000001, whole genome shotgun sequence	bacteria'
CCSF01000001.1 Pseudomonas sp. 20_BN genome assembly PRJEB6478_assembly_1, contig CONTIG000001, whole genome shotgun sequence	bacteria'

KN151326.1 Steinernema carpocapsae strain ALL unplaced genomic scaffold CARPO_906, whole genome shotgun sequence	invertebrates'
KN151899.1 Nilaparvata lugens isolate NLH13 unplaced genomic scaffold scaffold2, whole genome shotgun sequence	invertebrates'
KN165825.1 Steinernema feltiae strain SN unplaced genomic scaffold FELT_5455, whole genome shotgun sequence	invertebrates'
KN167705.1 Steinernema scapterisci strain FL unplaced genomic scaffold SCAPT_2260, whole genome shotgun sequence	invertebrates'
KN172139.1 Steinernema glaseri strain NC unplaced genomic scaffold GLAS_5991, whole genome shotgun sequence	invertebrates'
KN173723.1 Nilaparvata lugens yeast-like symbiont strain Hangzhou unplaced genomic scaffold scaffold1.1, whole genome shotgun sequence	fungi'
KN196491.1 Blattella germanica strain American Cyanamid = Orlando Normal breed German cockroach unplaced genomic scaffold Scaffold1, whole genome shotgun sequence	invertebrates'
CM002918.1 Lactobacillus plantarum CMPG5300 chromosome, whole genome shotgun sequence	bacteria'
CP008916.1 Burkholderia sp. BGK chromosome 1, complete sequence	bacteria'
KN214215.1 Castanea mollissima cultivar Vanuxem unplaced genomic scaffold scaffold00001, whole genome shotgun sequence	plants'
JRHR01003679.1 Pythium insidiosum strain CDC B5653 contig03679, whole genome shotgun sequence	protozoa'
JQDR02000001.1 Hyalella azteca isolate HAZT.00-mixed Contig1_fixed, whole genome shotgun sequence	invertebrates'
JQFK01000001.1 Pichia kudriavzevii strain SD108 scaffold00001, whole genome shotgun sequence	fungi'
CCBP010000119.1 Trametes cinnabarina BRFM137 WGS project CCBP000000000 data, contig scf185007, whole genome shotgun sequence	fungi'
HG918267.1 Capra aegagrus genomic scaffold, scaffold00001, whole genome shotgun sequence	vertebrates_mammals'
HG924883.1 Ovis aries musimon genomic scaffold, scaffold00001, whole genome shotgun sequence	vertebrates_mammals'
CP009578.1 Escherichia coli FAP1 genome	bacteria'
JRIB01000569.1 Saccharomyces cerevisiae strain YS9 scaffold-682, whole genome shotgun sequence	fungi'
JRIC01000262.1 Saccharomyces cerevisiae strain YPS163 scaffold-270, whole genome shotgun sequence	fungi'
JRIE01000200.1 Saccharomyces cerevisiae strain YJM339 scaffold-208, whole genome shotgun sequence	fungi'
JRID01000075.1 Saccharomyces cerevisiae strain YPS128 scaffold-78, whole genome shotgun sequence	fungi'
JRIF01000017.1 Saccharomyces cerevisiae strain Y55 scaffold-19, whole genome shotgun sequence	fungi'
JRIH01000084.1 Saccharomyces cerevisiae strain SK1 scaffold-103, whole genome shotgun sequence	fungi'
JRIG01000095.1 Saccharomyces cerevisiae strain DBVPG6044 scaffold-99, whole genome shotgun sequence	fungi'
JRIL01000146.1 Saccharomyces cerevisiae strain RedStar scaffold-222, whole genome shotgun sequence	fungi'
JRII01000088.1 Saccharomyces cerevisiae strain BC187 scaffold-90, whole genome shotgun sequence	fungi'
JRIO01000036.1 Saccharomyces cerevisiae strain YPH499 scaffold-43, whole genome shotgun sequence	fungi'
JRIN01000192.1 Saccharomyces cerevisiae strain FY1679 scaffold-244, whole genome shotgun sequence	fungi'
JRIQ01000037.1 Saccharomyces cerevisiae strain 10560-6B scaffold-50, whole genome shotgun sequence	fungi'
JRIR01000143.1 Saccharomyces cerevisiae strain BY4742 scaffold-188, whole genome shotgun sequence	fungi'
JRIZ01000011.1 Saccharomyces cerevisiae strain JK9-3d scaffold-12, whole genome shotgun sequence	fungi'
JRIU01000133.1 Saccharomyces cerevisiae strain W303 scaffold-169, whole genome shotgun sequence	fungi'
JRIV01000074.1 Saccharomyces cerevisiae strain CEN.PK2-1Ca scaffold-97, whole genome shotgun sequence	fungi'
JRIT01000140.1 Saccharomyces cerevisiae strain FL100 scaffold-178, whole genome shotgun sequence	fungi'
JRIX01000074.1 Saccharomyces cerevisiae strain X2180-1A scaffold-91, whole genome shotgun sequence	fungi'
JRIY01000123.1 Saccharomyces cerevisiae strain D273-10B scaffold-169, whole genome shotgun sequence	fungi'
JRIS01000158.1 Saccharomyces cerevisiae strain BY4741 scaffold-202, whole genome shotgun sequence	fungi'
JRIW01000103.1 Saccharomyces cerevisiae strain SEY6210 scaffold-124, whole genome shotgun sequence	fungi'
JRIK01000357.1 Saccharomyces cerevisiae strain L1528 scaffold-371, whole genome shotgun sequence	fungi'
JRIP01000004.1 Saccharomyces cerevisiae strain RM11-1A scaffold-3, whole genome shotgun sequence	fungi'
KN265652.1 Aquila chrysaetos canadensis isolate GSEH35GE unplaced genomic scaffold Scaffold0, whole genome shotgun sequence	vertebrates_non_mammals'
KN880357.1 Phlebopus portentosus strain PP33 unplaced genomic scaffold Scaffold1, whole genome shotgun sequence	fungi'
JNBV01000001.1 Taiwanofungus camphoratus strain monokaryon S27 scaf1, whole genome shotgun sequence	fungi'
KN266224.1 Dermatophagoides farinae isolate Derf unplaced genomic scaffold scaffold_1, whole genome shotgun sequence	invertebrates'
CP007555.1 Coxiella burnetii str. Namibia genome	bacteria'
FN827340.1 Danio rerio strain Tuebingen, whole genome shotgun sequence assembly, Supercontig_0000001	vertebrates_non_mammals'
KN268387.1 Vicugna pacos huacaya unplaced genomic scaffold scaffold1, whole genome shotgun sequence	vertebrates_mammals'
KN271213.1 Camelus dromedarius breed Arabia unplaced genomic scaffold scaffold76, whole genome shotgun sequence	vertebrates_mammals'
KN277429.1 Camelus bactrianus breed Alxa unplaced genomic scaffold scaffold37, whole genome shotgun sequence	vertebrates_mammals'
JRIJ01000167.1 Saccharomyces cerevisiae strain K11 scaffold-173, whole genome shotgun sequence	fungi'
JRIM01000215.1 Saccharomyces cerevisiae strain UWOPS05_217_3 scaffold-215, whole genome shotgun sequence	fungi'
KN284664.1 Crocodylus porosus isolate Cpor-Errol unplaced genomic scaffold scaffold-9763, whole genome shotgun sequence	vertebrates_non_mammals'
JROU02000464.1 Cyclospora cayetanensis strain CHN_HEN01 contig_143, whole genome shotgun sequence	protozoa'
KN296311.1 Rhinopithecus roxellana isolate Xiao Hai unplaced genomic scaffold ENSRROG024130, whole genome shotgun sequence	vertebrates_mammals'
JPYM01000001.1 Fusarium avenaceum strain Fa05001 Fave_05001_scaffold1.1, whole genome shotgun sequence	fungi'
CM002938.1 Saccharomyces sp. 'boulardii' strain ATCC MYA-796 chromosome IV, whole genome shotgun sequence	fungi'
JPKY01000001.1 Acremonium chrysogenum ATCC 11550 scaffold1, whole genome shotgun sequence	fungi'
JQGE01000019.1 Fusarium avenaceum strain FaLH27 Fave_LH27_contig_3, whole genome shotgun sequence	fungi'
JQGD01000026.1 Fusarium avenaceum strain FaLH03 Fave_LH03_contig_2, whole genome shotgun sequence	fungi'
CP009684.1 Bacillus subtilis strain B-1 genome	bacteria'
CP009656.1 Borrelia burgdorferi strain B31, complete genome	bacteria'
CP009270.1 Burkholderia pseudomallei MSHR2243 chromosome 1, complete sequence	bacteria'
CP009271.1 Burkholderia pseudomallei MSHR1153 chromosome 1, complete sequence	bacteria'
CP009153.1 Burkholderia pseudomallei MSHR3965 chromosome 1 sequence	bacteria'
CM002998.1 Nasalis larvatus isolate Charlie chromosome align_Mm1, whole genome shotgun sequence	vertebrates_mammals'
KN305596.1 Sporisorium scitamineum strain 2014001 unplaced genomic scaffold scaffold_1, whole genome shotgun sequence	fungi'
CM002977.3 Macaca mulatta isolate 17573 chromosome 1, whole genome shotgun sequence	vertebrates_mammals'
AJIX01000003.1 Candida albicans P78048 supercont1.3, whole genome shotgun sequence	fungi'
AJIW01000012.1 Candida albicans GC75 supercont1.12, whole genome shotgun sequence	fungi'
AJIU01000001.1 Candida albicans P37005 supercont2.1, whole genome shotgun sequence	fungi'
AJIS01000033.1 Candida albicans P94015 supercont1.33, whole genome shotgun sequence	fungi'
AJIY01000001.1 Candida albicans P57072 supercont1.1, whole genome shotgun sequence	fungi'
AJJA01000002.1 Candida albicans P37037 supercont1.2, whole genome shotgun sequence	fungi'
AJIQ01000005.1 Candida albicans 12C supercont2.5, whole genome shotgun sequence	fungi'
AJIT01000001.1 Candida albicans P87 supercont2.1, whole genome shotgun sequence	fungi'
JRBH01000517.1 Diospyros lotus cultivar Kunsenshi Contig517, whole genome shotgun sequence	plants'
CM003019.1 Burkholderia pseudomallei MSHR435 chromosome 1, whole genome shotgun sequence	bacteria'
JPPO02000002.1 Hanseniaspora uvarum strain 34-9 scaffold_1, whole genome shotgun sequence	fungi'
JPYR01000001.1 Belgica antarctica isolate Palmer_Station_2011 Ban.contig0001, whole genome shotgun sequence	invertebrates'
JOTO01200203.1 Vaccinium macrocarpon cultivar Ben Lear (CNJ99-125-1 inbred clone) scaffold_3 mitochondrial, whole genome shotgun sequence	plants'
KN323322.1 Gavialis gangeticus isolate Ggan-Ray unplaced genomic scaffold scaffold60, whole genome shotgun sequence	vertebrates_non_mammals'
AJIV01000007.1 Candida albicans 19F supercont2.7, whole genome shotgun sequence	fungi'
AJIR01000008.1 Candida albicans L26 supercont2.8, whole genome shotgun sequence	fungi'
AJIZ01000001.1 Candida albicans P34048 supercont1.1, whole genome shotgun sequence	fungi'
AJJB01000009.1 Candida albicans P57055 supercont1.9, whole genome shotgun sequence	fungi'
AJJC01000012.1 Candida albicans P75063 supercont1.12, whole genome shotgun sequence	fungi'
JSUU01002258.1 Cyprinodon nevadensis pectoralis isolate South Scruggs Spring subpopulation scaffold2258, whole genome shotgun sequence	vertebrates_non_mammals'
AJJE01000008.1 Candida albicans P76067 supercont1.8, whole genome shotgun sequence	fungi'
AJJD01000004.1 Candida albicans P76055 supercont1.4, whole genome shotgun sequence	fungi'
JSXQ01000004.1 Candida albicans P37039 supercont1.4, whole genome shotgun sequence	fungi'
JSXP01000002.1 Candida albicans P60002 supercont1.2, whole genome shotgun sequence	fungi'
JSXR01000016.1 Candida albicans P75010 supercont1.16, whole genome shotgun sequence	fungi'
JPEV01000011.1 Candida albicans P75016 supercont1.11, whole genome shotgun sequence	fungi'
JPEW01000006.1 Candida albicans P78042 supercont1.6, whole genome shotgun sequence	fungi'
AJJF01000001.1 Candida albicans SC5314 supercont4.1, whole genome shotgun sequence	fungi'
JTBX01000023.1 Candida albicans SC5314 supercont3.23, whole genome shotgun sequence	fungi'
AJJG01000010.1 Candida albicans Ca6 supercont2.10, whole genome shotgun sequence	fungi'
CP009909.1 Paenibacillus polymyxa strain CF05 genome	bacteria'
KN360965.1 Uromyces viciae-fabae strain I2 unplaced genomic scaffold scaffold_1, whole genome shotgun sequence	fungi'
JPWU02000001.1 Phytophthora kernoviae strain NZFS 3630 scf_22359_1.contig_1, whole genome shotgun sequence	protozoa'
JPWV02000002.1 Phytophthora kernoviae strain NZFS 2646 scf_22199_2.contig_1, whole genome shotgun sequence	protozoa'
JTEQ01000322.1 Piezodorus guildinii isolate RBSB-Aug2013 contig_323, whole genome shotgun sequence	invertebrates'
KN707963.1 Homo sapiens unplaced genomic scaffold decoy20001	vertebrates_mammals'
LN625285.1 Cryptococcus gattii R265 genomic scaffold, contig_6, whole genome shotgun sequence	fungi'
HG428765.1 Chironomus tentans genomic scaffold, ct01_s1, whole genome shotgun sequence	invertebrates'
KN389625.1 Neurospora crassa strain 73 unplaced genomic scaffold GE21scaffold_1, whole genome shotgun sequence	fungi'
KN476293.1 Periophthalmodon schlosseri unplaced genomic scaffold scaffold2269, whole genome shotgun sequence	vertebrates_non_mammals'
KN462367.1 Periophthalmus magnuspinnatus unplaced genomic scaffold scaffold2, whole genome shotgun sequence	vertebrates_non_mammals'
KN493925.1 Scartelaos histophorus unplaced genomic scaffold scaffold2025, whole genome shotgun sequence	vertebrates_non_mammals'
DF837573.1 Acytostelium subglobosum LB1 DNA, scaffold: scaffold1, whole genome shotgun sequence	protozoa'
DF357216.1 Solanum melongena DNA, scaffold: Sme2.5_00003.1, whole genome shotgun sequence	plants'
KN398398.1 Gossypium arboreum cultivar AKA8401 unplaced genomic scaffold scaffold_14, whole genome shotgun sequence	plants'
KN521806.1 Boleophthalmus pectinirostris unplaced genomic scaffold scaffold1, whole genome shotgun sequence	vertebrates_non_mammals'
JTKG01000001.1 Primula veris cultivar Vogt026P Contig0, whole genome shotgun sequence	plants'
KN538681.1 Oryza longistaminata unplaced genomic scaffold Scaffold119344, whole genome shotgun sequence	plants'
JFBF01000001.1 Bactrocera dorsalis strain Punador scaffold00001, whole genome shotgun sequence	invertebrates'
KN538375.1 Trichuris suis unplaced genomic scaffold T_suis-1.0_Cont0, whole genome shotgun sequence	invertebrates'
KN549200.1 Oesophagostomum dentatum strain OD-Hann unplaced genomic scaffold O_dentatum_1.0_Cont1, whole genome shotgun sequence	invertebrates'
JNVN01000008.1 Erysiphe necator strain c c-strain-scaffold_14, whole genome shotgun sequence	fungi'
JNUS01000009.1 Erysiphe necator strain branching branching-scaffold_10, whole genome shotgun sequence	fungi'
JNUT01000020.1 Erysiphe necator strain ranch-9 ranch9-scaffold_23, whole genome shotgun sequence	fungi'
JNUU01000038.1 Erysiphe necator strain lodi lodi-scaffold_43, whole genome shotgun sequence	fungi'
KN613455.1 Vipera berus berus isolate VBER.BE-female unplaced genomic scaffold scaffold_0, whole genome shotgun sequence	vertebrates_non_mammals'
JUJX01000001.1 Diaporthe longicolla strain TWH P74 scaffold_1, whole genome shotgun sequence	fungi'
JRUI02000006.1 Raphanus sativus cultivar WK10039 R6, whole genome shotgun sequence	plants'
JPDR01000001.1 Penicillium chrysogenum strain IB 08/921 Pc3_contig_1, whole genome shotgun sequence	fungi'
JWIN01034149.1 Camelus dromedarius breed African isolate Drom800 8669507_scaffold, whole genome shotgun sequence	vertebrates_mammals'
JPKZ01022848.1 Toxocara canis isolate PN_DK_2014 scaffold806, whole genome shotgun sequence	invertebrates'
AZHE01000001.1 Metarhizium album ARSEF 1941 scaffold_1, whole genome shotgun sequence	fungi'
JRXJ01000010.1 Cryptosporidium hominis isolate 37999 contig_10, whole genome shotgun sequence	protozoa'
KN709239.1 Nelumbo nucifera cultivar Chinese Tai-zi lotus unplaced genomic scaffold scaffold396, whole genome shotgun sequence	plants'
KN711236.1 Magnaporthe oryzae strain 98-06 unplaced genomic scaffold scaffold_1, whole genome shotgun sequence	fungi'
KN711580.1 Bactrocera cucurbitae strain USDA-PBARC White Pupae T1 unplaced genomic scaffold scaffold00001, whole genome shotgun sequence	invertebrates'
KN713669.1 Fopius arisanus strain USDA-PBARC FA_bdor unplaced genomic scaffold scaffold00001, whole genome shotgun sequence	invertebrates'
JRUC01000001.1 Leptographium procerum strain CMW34542 scaffold1, whole genome shotgun sequence	fungi'
CM003052.1 Xanthomonas translucens pv. cerealis strain CFBP 2541 chromosome, whole genome shotgun sequence	bacteria'
CP005977.1 Lactobacillus brevis BSO 464 genome	bacteria'
CP010342.1 Bacillus anthracis strain A0157 sequence	bacteria'
KN714663.1 Coccomyxa sp. LA000219 unplaced genomic scaffold scaffold2, whole genome shotgun sequence	plants'
JUDQ01000001.1 Scedosporium aurantiacum strain WM 09.24 scaffold-1, whole genome shotgun sequence	fungi'
JOMC01000025.1 Aspergillus ustus strain 3.3904 AUSC_25, whole genome shotgun sequence	fungi'
JWJA01004519.1 Rutstroemia echinophila strain CBS 111548 Joined_contig_4, whole genome shotgun sequence	fungi'
CP007548.1 Treponema pallidum subsp. endemicum str. Bosnia A genome	bacteria'
KN714666.1 Valsa mali var. pyri strain SXYL134 unplaced genomic scaffold scaffold00001, whole genome shotgun sequence	fungi'
JSSU01001405.1 Ceratocystis albifundus strain CMW17620 scaffold1, whole genome shotgun sequence	fungi'

AZNE01000001.1 Metarhizium majus ARSEF 297 Scaffold_1, whole genome shotgun sequence	fungi'
AZNH01000001.1 Metarhizium guizhouense ARSEF 977 scaffold_1, whole genome shotgun sequence	fungi'
AZNG01000001.1 Metarhizium brunneum ARSEF 3297 scaffold_1, whole genome shotgun sequence	fungi'
AZNF01000001.1 Metarhizium anisopliae ARSEF 549 scaffold_1, whole genome shotgun sequence	fungi'
KN715323.1 Mrakia frigida strain Nwmf-AP1 unplaced genomic scaffold scaffold1, whole genome shotgun sequence	fungi'
KN715895.1 Penicillium chrysogenum KF-25 unplaced genomic scaffold scaffold5, whole genome shotgun sequence	fungi'
KN716150.1 Dictyocaulus viviparus strain HannoverDv2000 unplaced genomic scaffold D_viviparus-1.0_Cont1, whole genome shotgun sequence	invertebrates'
KN726132.1 Ancylostoma duodenale strain Zhejiang unplaced genomic scaffold A_duodenale-1.0_Cont1, whole genome shotgun sequence	invertebrates'
CP006902.1 Clostridium botulinum Prevot_594 genome	bacteria'
CBXB010000009.1 Saprochaete clavata CNRMA 12.647 WGS project CBXB000000000 data, contig scaffold_9, whole genome shotgun sequence	fungi'
HG417165.1 Oryza rufipogon genomic scaffold, chr01, whole genome shotgun sequence	plants'
CM003064.1 Oryza sativa Japonica Group cultivar HEG4 isolate HEG4_2.0 chromosome 1, whole genome shotgun sequence	plants'
CM003076.1 Oryza sativa Japonica Group cultivar A123 chromosome 1, whole genome shotgun sequence	plants'
CM003098.1 Valsa mali strain 03-8 chromosome 1, whole genome shotgun sequence	fungi'
JTFG02000001.1 Ensete ventricosum cultivar Bedadit scf_6473_1.contig_1, whole genome shotgun sequence	plants'
KN796172.1 Glossina palpalis gambiensis isolate 146720 unplaced genomic scaffold Scaffold1, whole genome shotgun sequence	invertebrates'
CM003111.1 Vibrio cholerae O1 biovar El Tor strain I-1471 chromosome I, whole genome shotgun sequence	bacteria'
JROL01007040.1 Triticum aestivum cultivar Chinese Spring chromosome 4D map short arm chr4DS00002, whole genome shotgun sequence	plants'
KN805369.1 Trebouxia gelatinosa isolate LA000220 unplaced genomic scaffold scaffold1, whole genome shotgun sequence	plants'
KN805370.1 Schizochytrium sp. CCTCC M209059 unplaced genomic scaffold scaffold_1, whole genome shotgun sequence	protozoa'
JSUV01450192.1 Apis mellifera intermissa strain Blida - Haddad scaffold1_size134357, whole genome shotgun sequence	invertebrates'
CP009313.1 Streptomyces nodosus strain ATCC 14899 genome	bacteria'
JMCE01000007.1 Drosophila simulans strain M252 chromosome 3R, whole genome shotgun sequence	invertebrates'
HG977522.1 Acinetobacter baumannii CS01 genomic scaffold, scaffold00001, whole genome shotgun sequence	bacteria'
HG977526.1 Acinetobacter baumannii CR17 genomic scaffold, scaffold00001, whole genome shotgun sequence	bacteria'
CDMT01009900.1 Fasciola hepatica genome assembly Fh_reapr10kb, contig contig_9900, whole genome shotgun sequence	invertebrates'
CDHN01000001.1 Torrubiella hemipterigena genome assembly Allpaths-LG, scaffold SCAF1, whole genome shotgun sequence	fungi'
CDGI01000001.1 Rhizopus microsporus genome assembly Rmicro_CBS_344.29_Allpaths-LG, scaffold SCAF1, whole genome shotgun sequence	fungi'
CDFH01096868.1 Acanthamoeba astronyxis genome assembly Acanthamoeba astronyxis, scaffold 396559, whole genome shotgun sequence	protozoa'
CDFF01071297.1 Acanthamoeba culbertsoni genome assembly Acanthamoeba culbertsoni genome assembly, scaffold 254822, whole genome shotgun sequence	protozoa'
CDFG01077627.1 Acanthamoeba lenticulata genome assembly Acanthamoeba lenticulata, scaffold 353499, whole genome shotgun sequence	protozoa'
CDFA01025757.1 Acanthamoeba healyi genome assembly Acanthamoeba healyi, scaffold 209159, whole genome shotgun sequence	protozoa'
CDFD01054303.1 Acanthamoeba palestinensis genome assembly Acanthamoeba palestinensis, scaffold 352945, whole genome shotgun sequence	protozoa'
CDFK01217088.1 Acanthamoeba polyphaga genome assembly Acanthamoeba polyphaga, scaffold 749387, whole genome shotgun sequence	protozoa'
CDEZ01022494.1 Acanthamoeba royreba genome assembly Acanthamoeba royreba, scaffold 139054, whole genome shotgun sequence	protozoa'
CDFC01062057.1 Acanthamoeba rhysodes genome assembly Acanthamoeba rhysodes, scaffold 348153, whole genome shotgun sequence	protozoa'
CDFI01106708.1 Acanthamoeba divionensis genome assembly Acanthamoeba divionensis, scaffold 404273, whole genome shotgun sequence	protozoa'
CDFB01062555.1 Acanthamoeba lugdunensis genome assembly Acanthamoeba lugdunensis, scaffold 422590, whole genome shotgun sequence	protozoa'
CDFN01041723.1 Acanthamoeba quina genome assembly Acanthamoeba quina, scaffold 369852, whole genome shotgun sequence	protozoa'
CDFE01062597.1 Acanthamoeba mauritaniensis genome assembly Acanthamoeba mauritaniensis, scaffold 417904, whole genome shotgun sequence	protozoa'
CDFL01219416.1 Acanthamoeba castellanii genome assembly Acanthamoeba castellanii, scaffold 753047, whole genome shotgun sequence	protozoa'
CDFJ01214958.1 Acanthamoeba pearcei genome assembly Acanthamoeba pearcei, scaffold 744952, whole genome shotgun sequence	protozoa'
CM003114.1 Ziziphus jujuba cultivar Dongzao chromosome 1, whole genome shotgun sequence	plants'
KN805525.1 Fundulus heteroclitus isolate Maine unplaced genomic scaffold Scaffold0, whole genome shotgun sequence	vertebrates_non_mammals'
JTLS01000001.1 Basidioascus undulatus strain DAOM 241956 scaffold1, whole genome shotgun sequence	fungi'
CP010455.1 Synthetic Escherichia coli C321.deltaA substrain rEc.y.dC.46, complete sequence	bacteria'
CP010456.1 Synthetic Escherichia coli C321.deltaA substrain rEc.b.dC.12, complete sequence	bacteria'
JRZD01019964.1 Eimeria nieschulzi isolate Landers Enie_427, whole genome shotgun sequence	protozoa'
KN839835.1 Hydnomerulius pinastri MD-312 unplaced genomic scaffold scaffold_1, whole genome shotgun sequence	fungi'
KN838536.1 Laccaria amethystina LaAM-08-1 unplaced genomic scaffold K443scaffold_1, whole genome shotgun sequence	fungi'
KN832554.1 Plicaturopsis crispa FD-325 SS-3 unplaced genomic scaffold PLICRscaffold_1, whole genome shotgun sequence	fungi'
KN837076.1 Sphaerobolus stellatus SS14 unplaced genomic scaffold SPHSTscaffold_1, whole genome shotgun sequence	fungi'
KN835132.1 Suillus luteus UH-Slu-Lm8-n1 unplaced genomic scaffold CY34scaffold_1, whole genome shotgun sequence	fungi'
KN834749.1 Gymnopus luxurians FD-317 M1 unplaced genomic scaffold GYMLUscaffold_1, whole genome shotgun sequence	fungi'
KN833685.1 Pisolithus microcarpus 441 unplaced genomic scaffold scaffold_1, whole genome shotgun sequence	fungi'
KN832970.1 Piloderma croceum F 1598 unplaced genomic scaffold scaffold_00001, whole genome shotgun sequence	fungi'
KN832870.1 Oidiodendron maius Zn unplaced genomic scaffold scaffold_1, whole genome shotgun sequence	fungi'
KN831944.1 Pisolithus tinctorius Marx 270 unplaced genomic scaffold scaffold_1, whole genome shotgun sequence	fungi'
KN831768.1 Hebeloma cylindrosporum h7 unplaced genomic scaffold scaffold_1, whole genome shotgun sequence	fungi'
KN824823.1 Paxillus rubicundulus Ve08.2h10 unplaced genomic scaffold scaffold_1, whole genome shotgun sequence	fungi'
KN824277.1 Serendipita vermifera MAFF 305830 unplaced genomic scaffold scaffold_1, whole genome shotgun sequence	fungi'
KN822004.1 Scleroderma citrinum Foug A unplaced genomic scaffold scaffold_1, whole genome shotgun sequence	fungi'
KN822942.1 Tulasnella calospora MUT 4182 unplaced genomic scaffold scaffold_1, whole genome shotgun sequence	fungi'
KN819323.1 Paxillus involutus ATCC 200175 unplaced genomic scaffold PAXINscaffold_1, whole genome shotgun sequence	fungi'
KN818222.1 Amanita muscaria Koide BX008 unplaced genomic scaffold scaffold_1, whole genome shotgun sequence	fungi'
KN817518.1 Hypholoma sublateritium FD-334 SS-4 unplaced genomic scaffold scaffold_1, whole genome shotgun sequence	fungi'
JWZT01005339.1 Thelohanellus kitauei isolate Wuqing scaffold04623, whole genome shotgun sequence	invertebrates'
JXLN01012231.1 Sarcoptes scabiei isolate Arlian Lab Contig12230, whole genome shotgun sequence	invertebrates'
AP014655.1 Pseudomonas sp. MT-1 DNA, nearly complete genome	bacteria'
DF933830.1 Talaromyces cellulolyticus DNA, scaffold: scf_tce0_034, strain Y-94, whole genome shotgun sequence	fungi'
LD000290.1 Humulus lupulus var. cordifolius DNA, scaffold: KR_scaffold1_size296610, whole genome shotgun sequence	plants'
CP007359.2 Salmonella enterica subsp. enterica serovar Enteritidis str. EC20100134 genome	bacteria'
CP007358.2 Salmonella enterica subsp. enterica serovar Enteritidis str. EC20100130 genome	bacteria'
LD132766.1 Humulus lupulus var. lupulus DNA, contig: SW_scaffold1_size296610, whole genome shotgun sequence	plants'
AP014548.1 Nonlabens marinus S1-08 DNA, nearly complete genome	bacteria'
CP010813.1 Bacillus anthracis strain Pollino sequence	bacteria'
KN840438.1 Phlebiopsis gigantea 11061_1 CR5-6 unplaced genomic scaffold PHLGIscaffold_1, whole genome shotgun sequence	fungi'
CDGK01002114.1 Rhizoctonia solani AG-1 IB genome assembly 7/3/14, contig contig02187, whole genome shotgun sequence	fungi'
CP009805.1 Botrytis cinerea B05.10 chromosome 1, complete sequence	fungi'
CP010287.1 Francisella tularensis subsp. tularensis strain Scherm genome	bacteria'
JSUI01006542.1 Huntiella omanensis strain CMW 11056 scaffold1, whole genome shotgun sequence	fungi'
CP009846.1 Yersinia enterocolitica strain 8081, complete genome	bacteria'
CP009996.1 Yersinia pestis strain Java9, complete genome	bacteria'
CP010293.1 Yersinia pestis strain Nairobi genome	bacteria'
CP007299.1 Mycobacterium tuberculosis 18b genome	bacteria'
CP010005.1 Bacillus thuringiensis serovar kurstaki strain HD 1, complete genome	bacteria'
KN846951.1 Exophiala sideris strain CBS 121828 unplaced genomic scaffold supercont1.1, whole genome shotgun sequence	fungi'
KN846956.1 Capronia semiimmersa strain CBS 27337 unplaced genomic scaffold supercont1.1, whole genome shotgun sequence	fungi'
KN846969.1 Fonsecaea pedrosoi CBS 271.37 unplaced genomic scaffold supercont1.1, whole genome shotgun sequence	fungi'
KN846980.1 Cladophialophora bantiana CBS 173.52 unplaced genomic scaffold supercont1.1, whole genome shotgun sequence	fungi'
KN847040.1 Cladophialophora immunda strain CBS 83496 unplaced genomic scaffold supercont1.1, whole genome shotgun sequence	fungi'
KN847317.1 Exophiala xenobiotica strain CBS 118157 unplaced genomic scaffold supercont1.1, whole genome shotgun sequence	fungi'
KN847332.1 Exophiala oligosperma strain CBS 72588 unplaced genomic scaffold supercont1.1, whole genome shotgun sequence	fungi'
KN847475.1 Rhinocladiella mackenziei CBS 650.93 unplaced genomic scaffold supercont1.1, whole genome shotgun sequence	fungi'
KN848630.1 Cryptococcus gattii EJB2 unplaced genomic scaffold supercont1.82, whole genome shotgun sequence	fungi'
KN848855.1 Cryptococcus gattii IND107 unplaced genomic scaffold supercont2.1, whole genome shotgun sequence	fungi'
KN848831.1 Cryptococcus gattii CBS 10090 unplaced genomic scaffold supercont1.1, whole genome shotgun sequence	fungi'
KN848525.1 Cryptococcus gattii 2001/935-1 unplaced genomic scaffold supercont1.1, whole genome shotgun sequence	fungi'
KN847492.1 Exophiala spinifera strain CBS 89968 unplaced genomic scaffold supercont1.1, whole genome shotgun sequence	fungi'
DF818471.1 Taphrina wiesneri JCM 22204 DNA, scaffold: Tw.scaffold0001.584812, whole genome shotgun sequence	fungi'
DF818696.1 Taphrina deformans JCM 22205 DNA, scaffold: Td.scaffold0001.398723, whole genome shotgun sequence	fungi'
DF819225.1 Taphrina flavorubra JCM 22207 DNA, scaffold: Tf.scaffold0001.480923, whole genome shotgun sequence	fungi'
DF820090.1 Taphrina populina JCM 22190 DNA, scaffold: Tp.scaffold0001.892765, whole genome shotgun sequence	fungi'
DF822067.1 Papilio polytes DNA, scaffold: scaffold1447, whole genome shotgun sequence	invertebrates'
DF825946.1 Papilio xuthus DNA, scaffold: scaffold1453, whole genome shotgun sequence	invertebrates'
DF938580.1 Fungal sp. No.11243 DNA, scaffold: sccafold0001, whole genome shotgun sequence	fungi'
KN847520.1 Exophiala mesophila strain CBS 40295 unplaced genomic scaffold supercont1.1, whole genome shotgun sequence	fungi'
KN847529.1 Verruconis gallopava strain CBS 43764 unplaced genomic scaffold supercont1.1, whole genome shotgun sequence	fungi'
KN848129.1 Cryptococcus gattii LA55 unplaced genomic scaffold supercont1.1, whole genome shotgun sequence	fungi'
KN847973.1 Cryptococcus gattii CA1280 unplaced genomic scaffold supercont1.1, whole genome shotgun sequence	fungi'
KN847920.1 Cryptococcus gattii Ru294 unplaced genomic scaffold supercont1.1, whole genome shotgun sequence	fungi'
KN847896.1 Cryptococcus gattii Ram5 unplaced genomic scaffold supercont1.1, whole genome shotgun sequence	fungi'
KN848062.1 Fonsecaea multimorphosa CBS 102226 unplaced genomic scaffold supercont1.1, whole genome shotgun sequence	fungi'
KN848947.1 Cryptococcus gattii 99/473 unplaced genomic scaffold supercont1.1, whole genome shotgun sequence	fungi'
CM003138.1 Streptococcus thermophilus 1F8CT chromosome, whole genome shotgun sequence	bacteria'
CM003139.1 Streptococcus thermophilus TH985 chromosome, whole genome shotgun sequence	bacteria'
KN848889.1 Cryptococcus gattii CA1873 unplaced genomic scaffold supercont1.1, whole genome shotgun sequence	fungi'
KN848504.1 Cryptococcus gattii CA1014 unplaced genomic scaffold supercont1.1, whole genome shotgun sequence	fungi'
KN849052.1 Cryptococcus gattii E566 unplaced genomic scaffold supercont1.1, whole genome shotgun sequence	fungi'
KN848922.1 Cryptococcus gattii MMRL2647 unplaced genomic scaffold supercont2.1, whole genome shotgun sequence	fungi'
JXWP01000001.1 Sarcocystis neurona strain SN1 scaffold00001, whole genome shotgun sequence	protozoa'
JPID01000044.1 Picochlorum sp. SENEW3 Picochlorum_contig_45, whole genome shotgun sequence	plants'
JRVE01000001.1 Fusarium circinatum strain GL1327 Scaffold1, whole genome shotgun sequence	fungi'
CP007437.1 Micrococcus luteus strain trpE16 genome	bacteria'
KN849314.1 Nothobranchius furzeri breed GRZ isolate 7 unplaced genomic scaffold GapFilledScaffold_73, whole genome shotgun sequence	vertebrates_non_mammals'
JWHW01028663.1 Papilio glaucus scaffold_247, whole genome shotgun sequence	invertebrates'
JWTJ01000563.1 Rhodotorula mucilaginosa strain C2.5t1 scaffold_563, whole genome shotgun sequence	fungi'
CP010940.1 Staphylococcus aureus strain 71A_S11 genome	bacteria'
CP010941.1 Staphylococcus aureus strain SA17_S6 genome	bacteria'
CP010942.1 Staphylococcus epidermidis strain 949_S8 genome	bacteria'
CP010943.1 Staphylococcus aureus strain 144_S7 genome	bacteria'
CP010944.1 Staphylococcus aureus strain 79_S10 genome	bacteria'
KN880431.1 Cylindrobasidium torrendii FP15055 ss-10 unplaced genomic scaffold CYLTOscaffold_1, whole genome shotgun sequence	fungi'
KN882095.1 Fistulina hepatica ATCC 64428 unplaced genomic scaffold FISHEscaffold_516, whole genome shotgun sequence	fungi'
KN882203.1 Parastichopus parvimensis isolate Sea Cucumber 01 unplaced genomic scaffold Scaffold1, whole genome shotgun sequence	invertebrates'
CP010895.1 Mycobacterium tuberculosis strain PR08 genome	bacteria'
KN902229.1 Catajapyx aquilonaris unplaced genomic scaffold scaffold339_cov111, whole genome shotgun sequence	invertebrates'
CP010952.1 Staphylococcus aureus strain 93b_S9 genome	bacteria'
KN905583.1 Cryptococcus gattii NT-10 unplaced genomic scaffold supercont1.1, whole genome shotgun sequence	fungi'
CP016603.1 Comamonas aquatica strain CJG genome	bacteria'
JYGD01000118.1 Dactylonectria macrodidyma strain JAC 15-245 Ilyonectria_contig_120, whole genome shotgun sequence	fungi'
KN905849.1 Nanorana parkeri isolate BGI_ZX_2015 unplaced genomic scaffold scaffold1, whole genome shotgun sequence	vertebrates_non_mammals'
LM525555.1 Strongyloides papillosus genome assembly S_papillosus_LIN, scaffold SPAL_scaffold0000001	invertebrates'
JYIL01003741.1 Lasallia pustulata isolate Sardinia_28052013 scaffold79, whole genome shotgun sequence	fungi'
LN736360.1 Lachancea lanzarotensis genome assembly LALA0, scaffold LALA0S01, whole genome shotgun sequence	fungi'

LN719426.1 Parasitella parasitica genome assembly PP.v1, scaffold scf_1359, whole genome shotgun sequence	fungi'
LK996042.1 Syphacia muris genome assembly S_muris_Valencia, scaffold SMUV_scaffold0000001	invertebrates'
LK927530.1 Caenorhabditis elegans genome assembly C_elegans_Bristol_N2_v1_5_4, scaffold CELN2_scaffold0000001	invertebrates'
CP010969.1 Rickettsia raoultii strain Khabarovsk genome	bacteria'
CP009770.1 Ureaplasma diversum strain ATCC 49782 genome	bacteria'
LM523158.1 Parastrongyloides trichosuri genome assembly P_trichosuri_KNP, scaffold PTRK_scaffold0000001	invertebrates'
LK995546.1 Rhabditophanes sp. KR3021 genome assembly Rhabditophanes_sp_KR3021, scaffold RSKR_scaffold0000001	invertebrates'
LK023313.1 Lichtheimia ramosa strain JMRC FSU:6197 genome assembly, scaffold: SCAF1	fungi'
LK978189.1 Dracunculus medinensis genome assembly D_medinensis_Ghana, scaffold DME_scaffold0000001	invertebrates'
LN627129.1 Fasciola hepatica genome assembly Fhepatica_v1, scaffold scaffold112, whole genome shotgun sequence	invertebrates'
LL999048.1 Strongyloides stercoralis genome assembly S_stercoralis_PV0001, scaffold SSTP_scaffold0000001	invertebrates'
JQHZ01077877.1 Catharanthus roseus cultivar Sunstorm Apricot cro_scaffold_3069583, whole genome shotgun sequence	plants'
DF939120.1 Vollenhovia emeryi DNA, scaffold: scaffold1, whole genome shotgun sequence	invertebrates'
LM676446.1 Protopolystoma xenopodis genome assembly P_xenopodis_South_Africa, scaffold PXEA_scaffold0000001	invertebrates'
LL188370.1 Heligmosomoides polygyrus genome assembly H_bakeri_Edinburgh, scaffold HPBE_scaffold0000001	invertebrates'
FJNM01000001.1 Onchocerca ochengi genome assembly, contig: OOCN_scaffold0000001, whole genome shotgun sequence	invertebrates'
LL569982.1 Diphyllobothrium latum genome assembly D_latum_Geneva, scaffold DILT_scaffold0000001	invertebrates'
LL233096.1 Echinostoma caproni genome assembly E_caproni_Egypt, scaffold ECPE_scaffold0000001	invertebrates'
DF836290.1 Mucor ambiguus DNA, scaffold: scf_mam1_v10001, strain NBRC 6742, whole genome shotgun sequence	fungi'
DF933568.1 Acanthaster planci DNA, scaffold: A_planci_scaf59_V0.5, isolate: AUS-Apl0001, whole genome shotgun sequence	invertebrates'
LK393106.1 Dicrocoelium dendriticum genome assembly D_dendriticum_Leon_v1_0_4, scaffold DDEL_scaffold0000001	invertebrates'
CM003179.1 Capsicum annuum var. glabriusculum chromosome 1, whole genome shotgun sequence	plants'
LL000001.1 Trichobilharzia regenti genome assembly T_regenti_v1_0_4, scaffold TRE_scaffold0000001	invertebrates'
LK903567.1 Brugia timori genome assembly B_timori_Indonesia_v1_0_4, scaffold BTMF_scaffold0000001	invertebrates'
LK945525.1 Angiostrongylus cantonensis genome assembly A_cantonensis_China, scaffold ACAC_scaffold0000001	invertebrates'
LK939141.1 Angiostrongylus costaricensis genome assembly A_costaricensis_Costa_Rica, scaffold ACOC_scaffold0000001	invertebrates'
KN983729.1 Colobus angolensis palliatus isolate OR3802 unplaced genomic scaffold Scaffold5, whole genome shotgun sequence	vertebrates_mammals'
KN972984.1 Mandrillus leucophaeus isolate KB7577 unplaced genomic scaffold Scaffold1, whole genome shotgun sequence	vertebrates_mammals'
LK871857.1 Ascaris lumbricoides genome assembly A_lumbricoides_Ecuador_v1_5_4, scaffold ALUE_scaffold0000001	invertebrates'
LL319179.1 Anisakis simplex genome assembly A_simplex, scaffold ASIM_scaffold0000001	invertebrates'
LK964160.1 Brugia pahangi genome assembly B_pahangi_Glasgow, scaffold BPAG_scaffold0000001	invertebrates'
LL361184.1 Cylicostephanus goldi genome assembly C_goldi_Cheshire, scaffold CGOC_scaffold0000001	invertebrates'
LL710276.1 Elaeophora elaphi genome assembly E_elaphi, scaffold EEL_scaffold0000001	invertebrates'
LM413552.1 Enterobius vermicularis genome assembly E_vermicularis_Canary_Islands, scaffold EVEC_scaffold0000001	invertebrates'
LL748445.1 Gongylonema pulchrum genome assembly G_pulchrum_Hokkaido, scaffold GPUH_scaffold0000001	invertebrates'
LM383430.1 Hymenolepis diminuta genome assembly H_diminuta_Denmark, scaffold HDID_scaffold0000001	invertebrates'
LM397340.1 Hymenolepis nana genome assembly H_nana_Japan, scaffold HNAJ_scaffold0000001	invertebrates'
LM583056.1 Haemonchus placei genome assembly H_placei_MHpl1, scaffold HPLM_scaffold0000001	invertebrates'
LM530258.1 Mesocestoides corti genome assembly M_corti_Specht_Voge, scaffold MCOS_scaffold0000001	invertebrates'
LM433384.1 Nippostrongylus brasiliensis genome assembly N_brasiliensis_RM07_v1_5_4, scaffold NBR_scaffold0000001	invertebrates'
LM537584.1 Onchocerca flexuosa genome assembly O_flexuosa_Cordoba, scaffold OFLC_scaffold0000001	invertebrates'
LM462759.1 Parascaris equorum genome assembly P_equorum, scaffold PEQ_scaffold0000001	invertebrates'
LM013521.1 Soboliphyme baturini genome assembly S_baturini_Dall_Island, scaffold SBAD_scaffold0000001	invertebrates'
LM065042.1 Schistosoma curassoni genome assembly S_curassoni_Dakar, scaffold SCUD_scaffold0000001	invertebrates'
LL876846.1 Schistosoma margrebowiei genome assembly S_margrebowiei_Zambia, scaffold SMRZ_scaffold0000001	invertebrates'
LM149319.1 Schistosoma mattheei genome assembly S_mattheei_Denwood, scaffold SMTD_scaffold0000001	invertebrates'
LL956979.1 Schistosoma rodhaini genome assembly S_rodhaini_Burundi, scaffold SROB_scaffold0000001	invertebrates'
LL900201.1 Schistocephalus solidus genome assembly S_solidus_NST_G2, scaffold SSLN_scaffold0000001	invertebrates'
LM211380.1 Strongylus vulgaris genome assembly S_vulgaris_Kentucky, scaffold SVUK_scaffold0000001	invertebrates'
LM125182.1 Taenia asiatica genome assembly T_asiatica_South_Korea, scaffold TASK_scaffold0000001	invertebrates'
LM035857.1 Toxocara canis genome assembly T_canis_Equador, scaffold TCNE_scaffold0000001	invertebrates'
LL718354.1 Hydatigera taeniaeformis genome assembly H_taeniaeformis_Canary_Islands, scaffold TTAC_scaffold0000001	invertebrates'
LM000001.1 Wuchereria bancrofti genome assembly W_bancrofti_Jakarta, scaffold WBA_scaffold0000001	invertebrates'
LN590686.1 Cyprinus carpio genome assembly common carp genome, scaffold: LG35, chromosome: 35	vertebrates_non_mammals'
LN000001.1 Spirometra erinaceieuropaei genome assembly S_erinaceieuropaei, scaffold SPER_scaffold0000001	invertebrates'
CP010981.1 Verticillium dahliae VdLs.17 chromosome 2, complete sequence	fungi'
KN985990.1 Aotus nancymaae isolate 85725 unplaced genomic scaffold Scaffold1, whole genome shotgun sequence	vertebrates_mammals'
LK931502.1 Salmonella enterica subsp. enterica serovar Dublin genome assembly SC50_1, chromosome : I	bacteria'
LN681234.1 [Clostridium] sordellii genome assembly JGS6382, chromosome : 1	bacteria'
LK931482.1 Salmonella enterica subsp. enterica serovar Pullorum genome assembly S44987_1, chromosome : I	bacteria'
LN679998.1 [Clostridium] sordellii genome assembly ATCC9714_, chromosome : 1	bacteria'
KQ012552.1 Cercocebus atys isolate FAK unplaced genomic scaffold Scaffold6, whole genome shotgun sequence	vertebrates_mammals'
KQ030498.1 Hirsutella minnesotensis 3608 unplaced genomic scaffold MNSD_Scf_1, whole genome shotgun sequence	fungi'
KQ009060.1 Macaca nemestrina isolate M95218 unplaced genomic scaffold Scaffold6, whole genome shotgun sequence	vertebrates_mammals'
KQ012994.1 Propithecus coquereli isolate 6110/MARCELLA unplaced genomic scaffold Scaffold1, whole genome shotgun sequence	vertebrates_mammals'
LD336106.1 Wasmannia auropunctata DNA, scaffold: scf7180000741428, whole genome shotgun sequence	invertebrates'
KQ001645.1 Plasmodium fragile strain nilgiri unplaced genomic scaffold supercont1.1, whole genome shotgun sequence	protozoa'
AZNP01000002.1 Thlaspi arvense cultivar MN106 Ta_scaffold_2, whole genome shotgun sequence	plants'
CP009942.1 Burkholderia mallei strain KC_1092 chromosome 1, complete sequence	bacteria'
CP010430.1 Geobacter sulfurreducens strain AM-1 genome	bacteria'
JZRJ01000184.1 Blastocystis sp. subtype 2 isolate Flemming ST2_BLS4_contig_189, whole genome shotgun sequence	protozoa'
JZRK01000279.1 Blastocystis sp. subtype 3 strain ZGR ST3_BLS3_contig_319, whole genome shotgun sequence	protozoa'
JZRL01000013.1 Blastocystis sp. subtype 4 strain BT1 ST4_BLS1_contig_14, whole genome shotgun sequence	protozoa'
JZRM01000029.1 Blastocystis sp. subtype 6 isolate SSI:754 ST6_BLS5_contig_38, whole genome shotgun sequence	protozoa'
JZRN01000032.1 Blastocystis sp. subtype 8 isolate Dmp/08-128 ST8_BLS6_contig_33, whole genome shotgun sequence	protozoa'
JZRO01000236.1 Blastocystis sp. subtype 9 isolate F5323 ST9_BLS7_contig_285, whole genome shotgun sequence	protozoa'
JYFM02000450.1 Clonostachys rosea strain 67-1 contig_2471, whole genome shotgun sequence	fungi'
JZUQ01000006.1 Trichoderma atroviride strain XS2015 scaffold6, whole genome shotgun sequence	fungi'
JZJM01000108.1 Aspergillus oryzae RIB40, whole genome shotgun sequence	fungi'
KQ031394.1 Clupea harengus unplaced genomic scaffold scaffold100, whole genome shotgun sequence	vertebrates_non_mammals'
LAJZ01000005.1 Fusarium graminearum strain 233423 contig_68, whole genome shotgun sequence	fungi'
LAKA01000051.1 Fusarium graminearum strain 241165 contig_13, whole genome shotgun sequence	fungi'
JZKD01S2518827.1 Picea glauca Pg-02r141203s2521875, whole genome shotgun sequence	plants'
JWIW01000100.1 Fusarium sp. JS1030 scaffold1, whole genome shotgun sequence	fungi'
JWIV01000063.1 Fusarium sp. JS626 scaffold1, whole genome shotgun sequence	fungi'
JWIU01000092.1 Xylaria sp. JS573 scaffold1, whole genome shotgun sequence	fungi'
HE798385.1 Achromobacter xylosoxidans NH44784-1996 complete genome	bacteria'
CM003192.1 Vibrio cholerae strain I-1300 chromosome I, whole genome shotgun sequence	bacteria'
FO203501.1 Klebsiella pneumoniae subsp. rhinoscleromatis strain SB3432, complete genome	bacteria'
LAEV01002237.1 Thielaviopsis punctulata isolate CR-DP1 NODE_1, whole genome shotgun sequence	fungi'
CP007357.2 Salmonella enterica subsp. enterica serovar Enteritidis str. EC20100100 genome	bacteria'
CP007361.2 Salmonella enterica subsp. enterica serovar Enteritidis str. SA20100349 genome	bacteria'
CP002710.1 Ashbya gossypii FDAG1 chromosome VI, complete sequence	fungi'
JXSR01S000001.1 Ophiothrix spiculata isolate Osp01 Scaffold1, whole genome shotgun sequence	invertebrates'
HE605379.1 Chlamydia trachomatis E/C599 high quality draft genome sequence	bacteria'
HE605380.2 Chlamydia trachomatis F/SWFPminus high quality draft genome sequence	bacteria'
CP009836.1 Yersinia pestis strain KIM5, complete genome	bacteria'
FO393427.1 strain 284/09 Stolbur phytoplasma draft	bacteria'
FO393428.1 strain 231/09 Stolbur phytoplasma draft	bacteria'
LAPY01000001.1 Eragrostis tef cultivar Tsedey (DZ-Cr-37) scaffold105, whole genome shotgun sequence	plants'
CM003196.1 Burkholderia pseudomallei MSHR1328 chromosome I, whole genome shotgun sequence	bacteria'
CM003194.1 Burkholderia pseudomallei MSHR1079 chromosome I, whole genome shotgun sequence	bacteria'
KQ039406.1 Anser cygnoides domesticus breed Zhedong unplaced genomic scaffold scaffold65, whole genome shotgun sequence	vertebrates_non_mammals'
CP010274.2 Flavobacterium psychrophilum strain 5 genome	bacteria'
CP010275.2 Flavobacterium psychrophilum strain MH1 genome	bacteria'
CP010276.2 Flavobacterium psychrophilum strain PG2, complete genome	bacteria'
CP010277.2 Flavobacterium psychrophilum strain VQ50 genome	bacteria'
CP010278.2 Flavobacterium psychrophilum strain 3 genome	bacteria'
CP010946.1 Mycobacterium chelonae genome	bacteria'
KQ041443.1 Larimichthys crocea isolate SSNF unplaced genomic scaffold scaffold2, whole genome shotgun sequence	vertebrates_non_mammals'
JXMZ02111976.1 Ixodes ricinus strain Charles River Contig10567, whole genome shotgun sequence	invertebrates'
FO818640.1 Arthrospira sp. str. PCC 8005 chromosome, complete genome	bacteria'
CM003202.2 Fusarium pseudograminearum strain CS3270 chromosome 1, whole genome shotgun sequence	fungi'
CM003210.1 Caenorhabditis elegans strain CB4856 chromosome V, whole genome shotgun sequence	invertebrates'
JPLX01000001.1 Sesamum indicum cultivar Swetha Scaffold_1, whole genome shotgun sequence	plants'
CP006437.1 Saccharomyces cerevisiae YJM195 chromosome XII sequence	fungi'
CP006438.1 Saccharomyces cerevisiae YJM244 chromosome XII sequence	fungi'
CP004102.1 Saccharomyces cerevisiae YJM1078 chromosome IV genomic sequence	fungi'
CP006377.1 Saccharomyces cerevisiae YJM1083 chromosome XII sequence	fungi'
CP006378.1 Saccharomyces cerevisiae YJM1129 chromosome XII sequence	fungi'
CP006435.1 Saccharomyces cerevisiae YJM189 chromosome XII sequence	fungi'
CP006436.1 Saccharomyces cerevisiae YJM193 chromosome XII sequence	fungi'
CP006439.1 Saccharomyces cerevisiae YJM248 chromosome XII sequence	fungi'
CP006440.1 Saccharomyces cerevisiae YJM270 chromosome XII sequence	fungi'
CP006441.1 Saccharomyces cerevisiae YJM271 chromosome XII sequence	fungi'
CP006442.1 Saccharomyces cerevisiae YJM320 chromosome XII sequence	fungi'
CP006443.1 Saccharomyces cerevisiae YJM326 chromosome XII sequence	fungi'
CP006444.1 Saccharomyces cerevisiae YJM428 chromosome XII sequence	fungi'
CP006445.1 Saccharomyces cerevisiae YJM450 chromosome XII sequence	fungi'
CP006446.1 Saccharomyces cerevisiae YJM451 chromosome XII sequence	fungi'
CP006447.1 Saccharomyces cerevisiae YJM453 chromosome XII sequence	fungi'
CP006448.1 Saccharomyces cerevisiae YJM456 chromosome XII sequence	fungi'
CP006449.1 Saccharomyces cerevisiae YJM470 chromosome XII sequence	fungi'
CP006450.1 Saccharomyces cerevisiae YJM541 chromosome XII sequence	fungi'
CP006451.1 Saccharomyces cerevisiae YJM554 chromosome XII sequence	fungi'
CP006452.1 Saccharomyces cerevisiae YJM555 chromosome XII sequence	fungi'
CP006453.1 Saccharomyces cerevisiae YJM627 chromosome XII sequence	fungi'
CP006454.1 Saccharomyces cerevisiae YJM681 chromosome XII sequence	fungi'
CP006455.1 Saccharomyces cerevisiae YJM682 chromosome XII sequence	fungi'
CP006456.1 Saccharomyces cerevisiae YJM683 chromosome XII sequence	fungi'
CP006457.1 Saccharomyces cerevisiae YJM689 chromosome XII sequence	fungi'
CP006458.1 Saccharomyces cerevisiae YJM693 chromosome XII sequence	fungi'
CP006459.1 Saccharomyces cerevisiae YJM969 chromosome XII sequence	fungi'
CP006460.1 Saccharomyces cerevisiae YJM972 chromosome XII sequence	fungi'
CP006461.1 Saccharomyces cerevisiae YJM975 chromosome XII sequence	fungi'

CP006462.1 Saccharomyces cerevisiae YJM978 chromosome XII sequence	fungi'
CP006463.1 Saccharomyces cerevisiae YJM981 chromosome XII sequence	fungi'
CP006464.1 Saccharomyces cerevisiae YJM984 chromosome XII sequence	fungi'
CP006465.1 Saccharomyces cerevisiae YJM987 chromosome XII sequence	fungi'
CP006466.1 Saccharomyces cerevisiae YJM990 chromosome XII sequence	fungi'
CP006468.1 Saccharomyces cerevisiae YJM996 chromosome XII sequence	fungi'
CP006379.1 Saccharomyces cerevisiae YJM1133 chromosome XII sequence	fungi'
CP006380.1 Saccharomyces cerevisiae YJM1190 chromosome XII sequence	fungi'
CP006381.1 Saccharomyces cerevisiae YJM1199 chromosome XII sequence	fungi'
CP006382.1 Saccharomyces cerevisiae YJM1202 chromosome XII sequence	fungi'
CP006383.1 Saccharomyces cerevisiae YJM1208 chromosome XII sequence	fungi'
CP006384.1 Saccharomyces cerevisiae YJM1242 chromosome XII sequence	fungi'
CP006385.1 Saccharomyces cerevisiae YJM1244 chromosome XII sequence	fungi'
CP006386.1 Saccharomyces cerevisiae YJM1248 chromosome XII sequence	fungi'
CP006387.1 Saccharomyces cerevisiae YJM1250 chromosome XII sequence	fungi'
CP006388.1 Saccharomyces cerevisiae YJM1252 chromosome XII sequence	fungi'
CP006389.1 Saccharomyces cerevisiae YJM1273 chromosome XII sequence	fungi'
CP006390.1 Saccharomyces cerevisiae YJM1304 chromosome XII sequence	fungi'
CP006391.1 Saccharomyces cerevisiae YJM1307 chromosome XII sequence	fungi'
CP006392.1 Saccharomyces cerevisiae YJM1311 chromosome XII sequence	fungi'
CP006393.1 Saccharomyces cerevisiae YJM1326 chromosome XII sequence	fungi'
CP006394.1 Saccharomyces cerevisiae YJM1332 chromosome XII sequence	fungi'
CP006395.1 Saccharomyces cerevisiae YJM1336 chromosome XII sequence	fungi'
CP006396.1 Saccharomyces cerevisiae YJM1338 chromosome XII sequence	fungi'
CP006397.1 Saccharomyces cerevisiae YJM1341 chromosome XII sequence	fungi'
CP006398.1 Saccharomyces cerevisiae YJM1342 chromosome XII sequence	fungi'
CP006399.1 Saccharomyces cerevisiae YJM1355 chromosome XII sequence	fungi'
CP006400.1 Saccharomyces cerevisiae YJM1356 chromosome XII sequence	fungi'
CP006401.1 Saccharomyces cerevisiae YJM1381 chromosome XII sequence	fungi'
CP006402.1 Saccharomyces cerevisiae YJM1383 chromosome XII sequence	fungi'
CP006403.1 Saccharomyces cerevisiae YJM1385 chromosome XII sequence	fungi'
CP006404.1 Saccharomyces cerevisiae YJM1386 chromosome XII sequence	fungi'
CP006405.1 Saccharomyces cerevisiae YJM1387 chromosome XII sequence	fungi'
CP006406.1 Saccharomyces cerevisiae YJM1388 chromosome XII sequence	fungi'
CP006407.1 Saccharomyces cerevisiae YJM1389 chromosome XII sequence	fungi'
CP006408.1 Saccharomyces cerevisiae YJM1399 chromosome XII sequence	fungi'
CP006409.1 Saccharomyces cerevisiae YJM1400 chromosome XII sequence	fungi'
CP006410.1 Saccharomyces cerevisiae YJM1401 chromosome XII sequence	fungi'
CP006411.1 Saccharomyces cerevisiae YJM1402 chromosome XII sequence	fungi'
CP006412.1 Saccharomyces cerevisiae YJM1415 chromosome XII sequence	fungi'
CP006413.1 Saccharomyces cerevisiae YJM1417 chromosome XII sequence	fungi'
CP006414.1 Saccharomyces cerevisiae YJM1418 chromosome XII sequence	fungi'
CP006415.1 Saccharomyces cerevisiae YJM1419 chromosome XII sequence	fungi'
CP006416.1 Saccharomyces cerevisiae YJM1433 chromosome XII sequence	fungi'
CP006417.1 Saccharomyces cerevisiae YJM1434 chromosome XII sequence	fungi'
CP006418.1 Saccharomyces cerevisiae YJM1439 chromosome XII sequence	fungi'
CP006419.1 Saccharomyces cerevisiae YJM1443 chromosome XII sequence	fungi'
CP006420.1 Saccharomyces cerevisiae YJM1444 chromosome XII sequence	fungi'
CP006421.1 Saccharomyces cerevisiae YJM1447 chromosome XII sequence	fungi'
CP006422.1 Saccharomyces cerevisiae YJM1450 chromosome XII sequence	fungi'
CP006423.1 Saccharomyces cerevisiae YJM1460 chromosome XII sequence	fungi'
CP006424.1 Saccharomyces cerevisiae YJM1463 chromosome XII sequence	fungi'
CP006425.1 Saccharomyces cerevisiae YJM1477 chromosome XII sequence	fungi'
CP006426.1 Saccharomyces cerevisiae YJM1478 chromosome XII sequence	fungi'
CP006427.1 Saccharomyces cerevisiae YJM1479 chromosome XII sequence	fungi'
CP006428.1 Saccharomyces cerevisiae YJM1526 chromosome XII sequence	fungi'
CP006429.1 Saccharomyces cerevisiae YJM1527 chromosome XII sequence	fungi'
CP006430.1 Saccharomyces cerevisiae YJM1549 chromosome XII sequence	fungi'
CP006431.1 Saccharomyces cerevisiae YJM1573 chromosome XII sequence	fungi'
CP006432.1 Saccharomyces cerevisiae YJM1574 chromosome XII sequence	fungi'
CP006433.1 Saccharomyces cerevisiae YJM1592 chromosome XII sequence	fungi'
CP006434.1 Saccharomyces cerevisiae YJM1615 chromosome XII sequence	fungi'
DF850543.1 Ipomoea trifida DNA, scaffold: Itr_sc000011.1, whole genome shotgun sequence	plants'
CM003214.1 Capra aegagrus isolate BamuBGIKM chromosome 1, whole genome shotgun sequence	vertebrates_mammals'
JXRI01000001.1 Saccharina japonica cultivar Ja scaffold1, whole genome shotgun sequence	protozoa'
DF424115.1 Balaenoptera bonaerensis DNA, contig: scaffold27089, whole genome shotgun sequence	vertebrates_mammals'
DF955598.1 Monomorium pharaonis DNA, scaffold: scaffold_1144, whole genome shotgun sequence	invertebrates'
DF884991.1 Ipomoea trifida DNA, scaffold: Itrk_sc0000001.1, whole genome shotgun sequence	plants'
BBSZ01013815.1 Subanguina moxae DNA, contig: SAMX.v0.8.1.Gap.contig_13830, strain: Nikko, whole genome shotgun sequence	invertebrates'
LK979539.1 Thelazia callipaeda genome assembly T_callipaeda_Ticino, scaffold TCLT_scaffold0000001	invertebrates'
LK391709.1 Babesia bigemina genome assembly Bbig001, chromosome : III	protozoa'
HF935907.1 Pyronema omphalodes CBS100304 genomic scaffold, Pcon_v1_scaffold862, whole genome shotgun sequence	fungi'
HF955199.1 Phytomonas sp. isolate Hart1 genomic scaffold, scaffold_1, whole genome shotgun sequence	protozoa'
LN625279.1 Candida parapsilosis GA1 genomic scaffold, supercontig16, whole genome shotgun sequence	fungi'
HG423343.1 Halomonas sp. A3H3 genomic chromosome, HALA3H3, whole genome shotgun sequence	bacteria'
JYJC01001720.1 Zymoseptoria ardabiliae isolate ST11IR_6.1.1 scaffold1720, whole genome shotgun sequence	fungi'
JYJD01002450.1 Zymoseptoria pseudotritici isolate ST04IR_5.5 scaffold2451, whole genome shotgun sequence	fungi'
JYJE01000085.1 Zymoseptoria brevis isolate ZB163 scaffold85, whole genome shotgun sequence	fungi'
CVMT01000001.1 Talaromyces islandicus genome assembly PIS, scaffold WF-38-12_scaffold00001, whole genome shotgun sequence	fungi'
CM003258.1 Gossypium hirsutum cultivar TM-1 chromosome 9, whole genome shotgun sequence	plants'
CP011369.1 Pseudomonas aeruginosa strain S04 90 genome	bacteria'
LBFM01006749.1 Achipteria coleoptrata isolate Acpool1_GW1 scaffold32656_cov275, whole genome shotgun sequence	invertebrates'
LCTU01000022.1 Rhodotorula toruloides strain IFO0559 JMS1_denovo_22, whole genome shotgun sequence	fungi'
LBFL01132443.1 Hypochthonius rufulus isolate Hrpool1_GW1 scaffold725479_cov129, whole genome shotgun sequence	invertebrates'
JOKZ01000001.1 Trichoderma harzianum strain T6776 Scaffolds0001.1, whole genome shotgun sequence	fungi'
LCTV01000023.1 Rhodotorula toruloides strain IFO0880 IFO0880denovo_23, whole genome shotgun sequence	fungi'
LBFN01100479.1 Steganacarus magnus isolate Smpool1_GW1, whole genome shotgun sequence	invertebrates'
LBFO01101358.1 Platynothrus peltifer isolate Pppool1_GW1, whole genome shotgun sequence	invertebrates'
LBNK01000001.1 Candida apicola strain NRRL Y-50540 Capi_0000001, whole genome shotgun sequence	fungi'
LCTY01000001.1 Brettanomyces anomalus strain YV396 scaffold1, whole genome shotgun sequence	fungi'
JARO02000007.1 Scleropages formosus isolate aro1 scaffold7, whole genome shotgun sequence	vertebrates_non_mammals'
LCTD01000014.1 Saccharomyces cerevisiae strain 4124-S60 PE150_denovo_14, whole genome shotgun sequence	fungi'
CP011421.1 Klebsiella pneumoniae strain yzusk-4 genome	bacteria'
LCWF01000064.1 Phaeomoniella chlamydospora isolate UCRPC4 PC4_UCR_scaffold_v01_64, whole genome shotgun sequence	fungi'
LAQI01000077.1 Diplodia seriata isolate DS831 DS_831_scaffold_v01_77, whole genome shotgun sequence	fungi'
LCUC01000059.1 Diaporthe ampelina isolate DA912 DA_912_scaffold_v01_60, whole genome shotgun sequence	fungi'
LAXH01000001.1 Tilletia horrida strain QB-1 smut0001, whole genome shotgun sequence	fungi'
LN483142.1 Xanthophyllomyces dendrorhous genome assembly Xden1, scaffold Scaffold_52	fungi'
KQ061688.1 Atkinsonella texensis strain B6155 unplaced genomic scaffold scaffold00001, whole genome shotgun sequence	fungi'
KQ061256.1 Epichloe bromicola strain AL0426/2 unplaced genomic scaffold scaffold00002, whole genome shotgun sequence	fungi'
KQ062009.1 Epichloe bromicola strain AL0434 unplaced genomic scaffold scaffold00001, whole genome shotgun sequence	fungi'
KQ062599.1 Atkinsonella texensis strain B6156 unplaced genomic scaffold scaffold00001, whole genome shotgun sequence	fungi'
LCZI01001061.1 Emmonsia crescens UAMH 3008 supercont1.392, whole genome shotgun sequence	fungi'
JMDK01000207.1 Penicillium italicum strain B3 scaffold204, whole genome shotgun sequence	fungi'
LBLR01014380.1 Lates calcarifer breed P12C03 Contig_14380, whole genome shotgun sequence	vertebrates_non_mammals'
KQ062924.1 Gerris buenoi breed undomesticated insect unplaced genomic scaffold Scaffold4, whole genome shotgun sequence	invertebrates'
CP010913.1 Sporisorium scitamineum strain SSC39 chromosome 1, complete sequence	fungi'
LBGY01002310.1 Hypothenemus hampei isolate Beltsville Coffee Borer Beetle colony scaffold2373, whole genome shotgun sequence	invertebrates'
KQ079791.1 Homo sapiens unplaced genomic scaffold Super-Scaffold_206, whole genome shotgun sequence	vertebrates_mammals'
JXOR01000175.1 Coboldia fuscipes isolate BV_Coboldia scaffold196, whole genome shotgun sequence	invertebrates'
JXOZ01001874.1 Drosophila melanogaster isolate BV_Dmelanogaster scaffold2410, whole genome shotgun sequence	invertebrates'
JXOY01000445.1 Drosophila busckii isolate BV_Dbusckii scaffold622, whole genome shotgun sequence	invertebrates'
JXPA01000737.1 Drosophila miranda isolate BV_Dmiranda scaffold1073, whole genome shotgun sequence	invertebrates'
JXPM01001559.1 Phortica variegata isolate BV_Pvariegata scaffold1919, whole genome shotgun sequence	invertebrates'
JXPK01019781.1 Trichoceridae sp. BV-2014 isolate BV_WinterCrane scaffold92161, whole genome shotgun sequence	invertebrates'
JXPD01001128.1 Mayetiola destructor isolate BV_Mayetiola scaffold1448, whole genome shotgun sequence	invertebrates'
JXPJ01006549.1 Scaptodrosophila lebanonensis isolate BV_Scaptodrosophila scaffold8349, whole genome shotgun sequence	invertebrates'
JXPY01000502.1 Drosophila pseudoobscura isolate BV_Dpseudo scaffold588, whole genome shotgun sequence	invertebrates'
JXPV01002434.1 Chironomus riparius isolate BV_Chironomus scaffold3280, whole genome shotgun sequence	invertebrates'
JXPS01000662.1 Glossina morsitans isolate BV_Glossina scaffold717, whole genome shotgun sequence	invertebrates'
JXPR01002951.1 Anopheles gambiae isolate BV_Anopheles scaffold4120, whole genome shotgun sequence	invertebrates'
JXPZ01004200.1 Themira minor isolate BV_Tminor scaffold7060, whole genome shotgun sequence	invertebrates'
JXPT01002265.1 Bactrocera oleae isolate BV_Boleae scaffold3132, whole genome shotgun sequence	invertebrates'
JXQA01001125.1 Trupanea jonesi isolate BV_Trupanea scaffold4127, whole genome shotgun sequence	invertebrates'
JXPQ01003030.1 Ephydra gracilis isolate BV_Egracilis scaffold3412, whole genome shotgun sequence	invertebrates'
LDEV01001580.1 Emmonsia parva UAMH 139 supercont1.242, whole genome shotgun sequence	fungi'
JXOU01025361.1 Chaoborus trivitattus isolate BV_Chaoborus scaffold36001, whole genome shotgun sequence	invertebrates'
JXPF01007241.1 Lucilia sericata isolate BV_Lucilia scaffold9482, whole genome shotgun sequence	invertebrates'
JXPH01004001.1 Mochlonyx cinctipes isolate BV_Mochlonyx scaffold4502, whole genome shotgun sequence	invertebrates'
JXOW01205685.1 Condylostylus patibulatus isolate BV_Cpatibulatus scaffold529165, whole genome shotgun sequence	invertebrates'
JXPU01079123.1 Aedes aegypti isolate BV_Aedes scaffold120614, whole genome shotgun sequence	invertebrates'
JXPW01013553.1 Hermetia illucens isolate BV_Hillucens scaffold20825, whole genome shotgun sequence	invertebrates'
JXHJ01001262.1 Liriomyza trifolii isolate BV_Liromyza scaffold2706, whole genome shotgun sequence	invertebrates'
JXOV01015551.1 Clogmia albipunctata isolate BV_Clogmia scaffold18959, whole genome shotgun sequence	invertebrates'
JXOX01006947.1 Drosophila albomicans isolate BV_Dalbomicans scaffold5486, whole genome shotgun sequence	invertebrates'
JXOS01017684.1 Cirrula hians isolate BV_Ehians scaffold31894, whole genome shotgun sequence	invertebrates'
JXPB01012640.1 Eutreta diana isolate BV_Ediana scaffold40725, whole genome shotgun sequence	invertebrates'
JXPC01098847.1 Eristalis dimidiata isolate BV_Edimidiata scaffold271803, whole genome shotgun sequence	invertebrates'
JXPG01001436.1 Megaselia abdita isolate BV_Megaselia scaffold1678, whole genome shotgun sequence	invertebrates'
JXPE01003786.1 Holcocephala fusca isolate BV_Holcocephala scaffold5076, whole genome shotgun sequence	invertebrates'
JXPL01032583.1 Sphyracephala brevicornis isolate BV_stalky scaffold62831, whole genome shotgun sequence	invertebrates'
KQ079922.1 Stomoxys calcitrans breed 8C7A2A5H3J4 unplaced genomic scaffold Scaffold0, whole genome shotgun sequence	invertebrates'
JXOT01102672.1 Calliphora vicina isolate BV_Calliphora scaffold202971, whole genome shotgun sequence	invertebrates'
JXPI01004095.1 Neobellieria bullata isolate BV_Sarcophaga scaffold7003, whole genome shotgun sequence	invertebrates'
JXPN01054074.1 Tephritis californica isolate BV_Tcalifornica scaffold141641, whole genome shotgun sequence	invertebrates'

JXPO01026306.1 Teleopsis dalmanni isolate BV_Teleopsis scaffold43585, whole genome shotgun sequence	invertebrates'
JXPP01004248.1 Tipula oleracea isolate BV_Tipulida scaffold6692, whole genome shotgun sequence	invertebrates'
KQ085882.1 Schizopora paradoxa strain KUC8140 unplaced genomic scaffold SCHPAscaffold_00001, whole genome shotgun sequence	fungi'
LDKB01000080.1 Chlorella vulgaris strain UTEX 395 Scaffold_89, whole genome shotgun sequence	plants'
LDJE01105914.1 Selaginella kraussiana cultivar Aurea, whole genome shotgun sequence	plants'
LDEE01000001.1 Stachybotrys chartarum strain 51-11 scaffold1, whole genome shotgun sequence	fungi'
JRVG01000122.1 Fusarium fujikuroi strain KSU X-10626 seq_122, whole genome shotgun sequence	fungi'
JRVF01000719.1 Fusarium fujikuroi strain FGSC 8932 seq_719, whole genome shotgun sequence	fungi'
JRVH01001900.1 Fusarium fujikuroi strain KSU 3368 seq_1900, whole genome shotgun sequence	fungi'
LDKF01000021.1 Rotylenchulus reniformis isolate USA_MS RREN_21, whole genome shotgun sequence	invertebrates'
CP011497.1 Streptomyces incarnatus strain NRRL 8089 sequence	bacteria'
KQ087177.1 Trichosporon oleaginosus strain IBC0246 unplaced genomic scaffold CC85scaffold_1, whole genome shotgun sequence	fungi'
LM524968.1 Strongyloides venezuelensis genome assembly S_venezuelensis_HH1, scaffold SVE_scaffold0000001	invertebrates'
CP011778.1 Torulaspora delbrueckii strain NRRL Y-50541 chromosome 1 sequence	fungi'
CP011813.1 Saccharomyces cerevisiae strain NCIM3186 chromosome IV sequence	fungi'
KQ087363.1 Aspergillus fumigatus Z5 chromosome Unknown scaffold_7, whole genome shotgun sequence	fungi'
BBXB01000001.1 Pythium insidiosum DNA, contig: PINS000001, strain: Pi-S, whole genome shotgun sequence	protozoa'
LFEI01000001.1 Lingula anatina isolate Amm_Jpn scaffold1, whole genome shotgun sequence	invertebrates'
CP011886.1 Ovis canadensis canadensis isolate 43U chromosome 1 sequence	vertebrates_mammals'
CAQS01000001.1 Romanomermis culicivorax WGS project CAQS00000000 data, contig scaf00001, whole genome shotgun sequence	invertebrates'
LK391419.1 Apteryx australis mantelli genome assembly AptMant0, scaffold scaffold27	vertebrates_non_mammals'
CP011941.1 Synechococcus sp. WH 8020 genome	bacteria'
LN609529.1 Strongyloides ratti genome assembly S_ratti_ED321, chromosome : 2	invertebrates'
CP011799.1 Streptomyces sp. PBH53 genome	bacteria'
CP011218.1 [Haemophilus] ducreyi strain CLU1 genome	bacteria'
CP011219.1 [Haemophilus] ducreyi strain CLU2 genome	bacteria'
CP011220.1 [Haemophilus] ducreyi strain CLU3 genome	bacteria'
CP011221.1 [Haemophilus] ducreyi strain CLU4 genome	bacteria'
CP011222.1 [Haemophilus] ducreyi strain GU1 genome	bacteria'
CP011223.1 [Haemophilus] ducreyi strain GU2 genome	bacteria'
CP011224.1 [Haemophilus] ducreyi strain GU3 genome	bacteria'
CP011225.1 [Haemophilus] ducreyi strain GU4 genome	bacteria'
CP011226.1 [Haemophilus] ducreyi strain GU5 genome	bacteria'
CP011227.1 [Haemophilus] ducreyi strain CLU5 genome	bacteria'
CP011228.1 [Haemophilus] ducreyi strain GU6 genome	bacteria'
CP011229.1 [Haemophilus] ducreyi strain GU7 genome	bacteria'
CP011230.1 [Haemophilus] ducreyi strain GU8 genome	bacteria'
CP011231.1 [Haemophilus] ducreyi strain GU9 genome	bacteria'
LELE01000001.1 Epichloe uncinata strain e167 Contig00001, whole genome shotgun sequence	fungi'
CP011488.2 Francisella tularensis subsp. holarctica strain OR96-0246, genome	bacteria'
CP011001.1 Methylophilales bacterium MBRSF5 genome	bacteria'
CP011002.1 Methylophilales bacterium MBRSH7 genome	bacteria'
CP011003.1 Methylophilales bacterium MBRSG12 genome	bacteria'
CP011967.1 Salmonella enterica subsp. enterica serovar Paratyphi A strain CMCC50093 genome	bacteria'
LBMM01000001.1 Lasius niger Lnig_2.1_1, whole genome shotgun sequence	invertebrates'
CP011952.1 Lactobacillus farciminis strain CNCM-I-3699-S genome	bacteria'
DF196826.1 Raphanus sativus DNA, scaffold: Rs_scaf1, whole genome shotgun sequence	plants'
JXPX01047056.1 Sarcophagidae sp. BV-2014 isolate BV_Sarcophagidae scaffold124557, whole genome shotgun sequence	invertebrates'
CDHK01000001.1 Penicillium brasilianum genome assembly Pbras_Allpaths-LG, scaffold SCAF1, whole genome shotgun sequence	fungi'
CDSF01000001.1 Plasmodiophora brassicae genome assembly pbe3.h15, scaffold scaffold_1, whole genome shotgun sequence	protozoa'
CVRJ01000001.1 Candida auris genome assembly VPCI 479/P/13, scaffold C_auris_VPCI_479_scaffold1, whole genome shotgun sequence	fungi'
LFMI01000206.1 Trichoderma parareesei isolate CBS 125925 contig_233, whole genome shotgun sequence	fungi'
CP011550.1 Saccharomyces cerevisiae strain ySR127 chromosome IV, complete sequence	fungi'
CBUC010000012.1 Triticum aestivum WGS project CBUC00000000 data, cultivar Chinese Spring, contig v443_0056, whole genome shotgun sequence	plants'
CDJJ020000213.1 Primula vulgaris genome assembly Primula Slocus, contig S-locus_groupB_ctg18, whole genome shotgun sequence	plants'
CAWC010000001.1 Onchocerca ochengi WGS project CAWC000000000 data, contig 00001_1, whole genome shotgun sequence	invertebrates'
CBLZ010042120.1 Hordeum vulgare subsp. vulgare WGS project CBLZ00000000 data, cultivar Morex, contig morex_assembly1_contig_42120, whole genome shotgun sequence	plants'
CCSG01000002.1 Babesia divergens genome assembly 454hybrid_PBjelly_v2, contig Contig1, whole genome shotgun sequence	protozoa'
LFLD01S000001.1 Thamnophis sirtalis isolate EDBJR-23777 Scaffold0, whole genome shotgun sequence	vertebrates_non_mammals'
CP012038.1 Salmonella enterica subsp. enterica serovar Ouakam strain GNT-01 genome	bacteria'
LBNP01000031.1 Saccharomyces cerevisiae strain ISO12 ISO12_TMB_C31, whole genome shotgun sequence	fungi'
JWJK01000100.1 Saccharomyces cerevisiae strain Ethanol Red ER_TMB_C100, whole genome shotgun sequence	fungi'
CAIX01000001.1 Albugo candida WGS project CAIX00000000 data, strain Ac Nc2, contig AcNc2_CONTIG_1_length_398508, whole genome shotgun sequence	protozoa'
CDMY01000227.1 Vitrella brassicaformis CCMP3155 genome assembly Vbrassicaformis, scaffold scaffold14, whole genome shotgun sequence	protozoa'
CEMG01181877.1 Baeospora myosura genome assembly Baeospora_myosura-8, contig 389920, whole genome shotgun sequence	fungi'
CEMH01203078.1 Macrocystidia cucumis genome assembly Macrocystidia_cucumis-8, contig 392262, whole genome shotgun sequence	fungi'
CVRD01107653.1 Clavaria fumosa genome assembly Cla_fum_l100.fa, contig 1723078, whole genome shotgun sequence	fungi'
CVRE01181062.1 Crepidotus sp. BD-2015 genome assembly Mycena-l100.fa, contig 1865608, whole genome shotgun sequence	fungi'
LFUI01000001.1 Balamuthia mandrillaris strain CDC-V039 unitig_1, whole genome shotgun sequence	protozoa'
LFYR01000729.1 Zostera marina strain Finnish scaffold_1, whole genome shotgun sequence	plants'
JTLQ01140304.1 Pantherophis guttatus isolate motley scaffold146349, whole genome shotgun sequence	vertebrates_non_mammals'
KQ236993.1 Amyelois transitella strain UIUC subculture of SPIRL-1966 unplaced genomic scaffold Atra_scaffold_0021, whole genome shotgun sequence	invertebrates'
KQ241597.1 Sphaeroforma arctica JP610 unplaced genomic scaffold supercont1.1, whole genome shotgun sequence	invertebrates'
JOTR01000002.1 Diuraphis noxia strain RWA2 scaffold_1, whole genome shotgun sequence	invertebrates'
JRES01001160.1 Lucilia cuprina strain LS Scaffold23, whole genome shotgun sequence	invertebrates'
JJRW01S0000001.1 Loxosceles reclusa isolate LREC.00 Scaffold1, whole genome shotgun sequence	invertebrates'
JZLH01S0094352.1 Eucidaris tribuloides isolate Etri01 Scaffold18492, whole genome shotgun sequence	invertebrates'
CP012177.1 Lactobacillus farciminis strain CNCM-I-3699-R genome	bacteria'
CP006806.1 Yersinia pestis 790 genome	bacteria'
CP006794.1 Yersinia pestis 1045 sequence	bacteria'
LGAM01000001.1 Bactrocera oleae isolate Demokritus scaffold1, whole genome shotgun sequence	invertebrates'
LFRF01000001.1 Tolypocladium ophioglossoides CBS 100239 scaffold_0, whole genome shotgun sequence	fungi'
LGST01000041.1 [Candida] auris strain 6684 Scaffold41, whole genome shotgun sequence	fungi'
CM003371.1 Vigna angularis cultivar Jingnong 6 chromosome 1, whole genome shotgun sequence	plants'
CP011326.1 Streptococcus agalactiae strain YM001 genome	bacteria'
CP011327.1 Streptococcus agalactiae strain GX064 genome	bacteria'
LGLR01000170.1 Stemphylium lycopersici strain CIDEFI 216 scaffold11, whole genome shotgun sequence	fungi'
AJIL01000002.1 Puccinia striiformis f. sp. tritici PST-78 strain race PST-78 isolate 2K041-Yr9 supercont1.1, whole genome shotgun sequence	fungi'
KQ417173.1 Octopus bimaculoides isolate UCB-OBI-ISO-001 unplaced genomic scaffold Scaffold12941, whole genome shotgun sequence	invertebrates'
LFNC01000001.1 Perkinsela sp. CCAP 1560/4 XU18scaffold_1, whole genome shotgun sequence	protozoa'
CP012251.1 Xanthomonas arboricola pv. juglandis strain Xaj 417 genome	bacteria'
JXZD01S001272.1 Solanum commersonii cultivar cmm1t scaffold1248, whole genome shotgun sequence	plants'
LN774881.1 Candidatus Westeberhardia cardiocondylae genome assembly Wobs_v01, chromosome : I	bacteria'
LM651872.1 Sporisorium scitamineum genome assembly Sporisorium scitamineum, scaffold SCAFFOLD000409, whole genome shotgun sequence	fungi'
LN717261.1 Microbotryum violaceum p1A1 Lamole genome assembly MvSlA1A2r3c, scaffold MvSlA1A2r3c_A1, whole genome shotgun sequence	fungi'
CDQK01000001.1 Cyberlindnera jadinii genome assembly cj1, scaffold CJ1, whole genome shotgun sequence	fungi'
CWKC01009650.1 Drosophila americana genome assembly D. americana W11 strain genome assembly, scaffold W11B_2217, whole genome shotgun sequence	invertebrates'
CWKB01009259.1 Drosophila americana genome assembly D. americana H5 strain genome assembly, scaffold H5C_670, whole genome shotgun sequence	invertebrates'
LGRX01000001.1 Cymbomonas tetramitiformis isolate PLY_AMNH scaffold_0, whole genome shotgun sequence	plants'
LGKQ01000001.1 Ciborinia camelliae strain ICMP 19812 scaffold1, whole genome shotgun sequence	fungi'
CM003382.1 Listeria monocytogenes strain JF5171 chromosome, whole genome shotgun sequence	bacteria'
LEOU01000017.1 Balamuthia mandrillaris strain 2046 scaffold16_cov0, whole genome shotgun sequence	protozoa'
LBNR01000001.1 Fusarium nygamai strain MRC8546 scaffold0000001, whole genome shotgun sequence	fungi'
LFLW010003085.1 Conus tribblei isolate 0317_1 scaffold3373, whole genome shotgun sequence	invertebrates'
LGHX01002207.1 Eulemur macaco isolate Harmonia scaffold2169, whole genome shotgun sequence	vertebrates_mammals'
LGHW01002226.1 Eulemur flavifrons isolate Harlow scaffold2169, whole genome shotgun sequence	vertebrates_mammals'
KQ412180.1 Termitomyces sp. J132 unplaced genomic scaffold scaffold66, whole genome shotgun sequence	fungi'
KQ414666.1 Habropoda laboriosa isolate 0110345459 unplaced genomic scaffold scaffold22, whole genome shotgun sequence	invertebrates'
LAVV01013495.1 Puccinia sorghi strain RO10H11247 Scaffold_7, whole genome shotgun sequence	fungi'
LGIB01000001.1 Neodiprion lecontei isolate Nlec_01 scaffold_1, whole genome shotgun sequence	invertebrates'
APLD01S000002.1 Phalaenopsis equestris Scaffold000002, whole genome shotgun sequence	plants'
CP012342.1 Corynebacterium riegelii strain PUDD_83A45 genome	bacteria'
LFFV01000213.1 Malassezia caprae strain CBS 10434 MC10434_173, whole genome shotgun sequence	fungi'
LFFW01000021.1 Malassezia cuniculi strain CBS 11721 MC11721_29, whole genome shotgun sequence	fungi'
LFFX01000059.1 Malassezia dermatis strain CBS 9169 MD9169_78, whole genome shotgun sequence	fungi'
LFFY01000025.1 Malassezia equina strain CBS 9969 ME9969_99, whole genome shotgun sequence	fungi'
LFGA01000522.1 Malassezia sympodialis strain ATCC 96806 MS96806_opera_scaffold_1, whole genome shotgun sequence	fungi'
LFCY01000347.1 Malassezia sympodialis strain ATCC 44340 MS44340_opera_scaffold_1, whole genome shotgun sequence	fungi'
LFCZ01000064.1 Malassezia restricta strain CBS 8742 MR8742_opera_scaffold_1, whole genome shotgun sequence	fungi'
LFDA01000068.1 Malassezia restricta strain CBS 7877 MR7877_opera_scaffold_1, whole genome shotgun sequence	fungi'
LFDB01000044.1 Malassezia japonica strain CBS 9431 MJ9431_opera_scaffold_1, whole genome shotgun sequence	fungi'
LFGE01000078.1 Malassezia globosa strain CBS 7990 MG7990_opera_scaffold_1, whole genome shotgun sequence	fungi'
LFGF01000040.1 Malassezia globosa strain CBS 7966 MG7966_opera_scaffold_1, whole genome shotgun sequence	fungi'
LFDC01000014.1 Malassezia globosa strain CBS 7874 MG7874_opera_scaffold_1, whole genome shotgun sequence	fungi'
LFDD01002563.1 Malassezia furfur strain CBS 7710 MF7710_opera_scaffold_1, whole genome shotgun sequence	fungi'
LFGG01003125.1 Malassezia furfur strain CBS 7019 MF7019_opera_scaffold_1, whole genome shotgun sequence	fungi'
LFCX01000032.1 Malassezia yamatoensis strain MY9725 MY9725_opera_scaffold_1, whole genome shotgun sequence	fungi'
LFGH01001658.1 Malassezia furfur strain CBS 4172 MF4172_opera_scaffold_1, whole genome shotgun sequence	fungi'
LFFZ01000464.1 Malassezia sympodialis strain ATCC 42132 MS42132_opera_scaffold_1, whole genome shotgun sequence	fungi'
LFGK01000345.1 Malassezia slooffiae strain CBS 7956 MSL7956_opera_scaffold_1, whole genome shotgun sequence	fungi'
LFGB01000018.1 Malassezia pachydermatis strain CBS 1879 MP1879_opera_scaffold_1, whole genome shotgun sequence	fungi'
LFGC01000610.1 Malassezia obtusa strain CBS 7876 MO7876_opera_scaffold_1, whole genome shotgun sequence	fungi'
LFGD01000081.1 Malassezia nana strain CBS 9557 MN9557_21, whole genome shotgun sequence	fungi'
LFGL01000113.1 Malassezia furfur strain CBS 7982 MF7982_opera_scaffold_1, whole genome shotgun sequence	fungi'
LFGI01002187.1 Malassezia furfur strain CBS 1878 MF1878_opera_scaffold_1, whole genome shotgun sequence	fungi'
LFGJ01001078.1 Malassezia furfur strain JPLK23 MFJPLK23_opera_scaffold_1, whole genome shotgun sequence	fungi'
JTDY01000043.1 Operophtera brumata isolate WM2013NL OBRU01_Sc00043, whole genome shotgun sequence	invertebrates'
LDAR01S000001.1 Austrofundulus limnaeus strain Quisiro Scaffold0, whole genome shotgun sequence	vertebrates_non_mammals'
CP012365.1 Pseudomonadaceae bacterium E5571 genome	bacteria'
CVQH01000001.1 Verticillium longisporum genome assembly vl1.denovo.v1, scaffold scaffold_1, whole genome shotgun sequence	fungi'
CVQI01000001.1 Verticillium longisporum genome assembly vl2.denovo.v1, scaffold scaffold_1, whole genome shotgun sequence	fungi'
LHQP01004371.1 Cyprinus carpio isolate UL-001 Contig4374, whole genome shotgun sequence	vertebrates_non_mammals'
KQ434319.1 Talaromyces purpureogenus strain MYA-38 unplaced genomic scaffold PPV01_SCF00067, whole genome shotgun sequence	fungi'
KQ434324.1 Amorphotheca resinae strain ZN1 unplaced genomic scaffold scaffold_1, whole genome shotgun sequence	fungi'
CM003438.1 Macaca mulatta chromosome Y	vertebrates_mammals'

CM003439.1 Lactobacillus plantarum subsp. plantarum strain CGMCC 1.557 chromosome, whole genome shotgun sequence	bacteria'
KQ434809.1 Dufourea novaeangliae isolate 0120121106 unplaced genomic scaffold scaffold8, whole genome shotgun sequence	invertebrates'
LAZP01000001.1 Ophiocordyceps unilateralis strain SC16a Contig_1, whole genome shotgun sequence	fungi'
CM003440.1 Candidatus Hodgkinia cicadicola isolate HCMAGTRE chromosome variant1, complete sequence, whole genome shotgun sequence	bacteria'
CP012094.1 Burkholderia pseudomallei strain 350105 chromosome 1 sequence	bacteria'
LCTW02000001.1 Madurella mycetomatis strain mm55 MMYC01_Sc2001, whole genome shotgun sequence	fungi'
KQ435302.1 Mucor circinelloides strain WJ11 unplaced genomic scaffold scaffold00001, whole genome shotgun sequence	fungi'
KQ435794.1 Melipona quadrifasciata isolate 0111107301 unplaced genomic scaffold scaffold9, whole genome shotgun sequence	invertebrates'
CP012523.1 Drosophila busckii chromosome 2L sequence	invertebrates'
CP012519.1 Bacillus anthracis strain Larissa, complete genome	bacteria'
LHTS01000013.1 Phytophthora ramorum strain EU2 isolate SOD58/12 scf_18997_12.contig_1, whole genome shotgun sequence	protozoa'
KQ438824.1 Phytophthora ramorum strain EU2 isolate SOD69/12 unplaced genomic scaffold scf_19143_22, whole genome shotgun sequence	protozoa'
KQ438555.1 Phytophthora ramorum strain EU2 isolate SOD22/12 unplaced genomic scaffold scf_18821_14, whole genome shotgun sequence	protozoa'
KQ439017.1 Phytophthora ramorum strain EU2 isolate SOD158/11 unplaced genomic scaffold scf_18469_19, whole genome shotgun sequence	protozoa'
LHTV01000025.1 Phytophthora ramorum strain EU2 isolate SOD136/11 scf_18210_18.contig_1, whole genome shotgun sequence	protozoa'
LHTX01000012.1 Phytophthora ramorum strain EU2 isolate SOD L51 scf_19300_8.contig_1, whole genome shotgun sequence	protozoa'
LHTW01000023.1 Phytophthora ramorum strain EU2 isolate SOD169/11 scf_18644_20.contig_1, whole genome shotgun sequence	protozoa'
LGAV01000001.1 Malassezia pachydermatis strain CBS 1879 scaffold1, whole genome shotgun sequence	fungi'
LGAG01000467.1 Lerema accius isolate 1769 scaffold992_cov65, whole genome shotgun sequence	invertebrates'
LGSR01000020.1 Escovopsis weberi scaffold00001, whole genome shotgun sequence	fungi'
LHQQ01000001.1 Penicillium nordicum strain DAOMC 185683 scaffold_1, whole genome shotgun sequence	fungi'
CP012600.1 Bacillus sp. FJAT-4402 genome	bacteria'
CP012601.1 Bacillus sp. FJAT-22090 genome	bacteria'
CP012602.1 Bacillus sp. FJAT-18017 genome	bacteria'
JRWH01000495.1 Brugia pahangi isolate LYL_UMHIR_BP2014 BP495, whole genome shotgun sequence	invertebrates'
LAII01000001.1 Zosterops lateralis melanops scaffold_0, whole genome shotgun sequence	vertebrates_non_mammals'
LJCY01000104.1 Chrysoporthe cubensis strain CMW 10028 scaffold_0001, whole genome shotgun sequence	fungi'
LJDH01000197.1 Saccharomyces cerevisiae NRRL Y-12632 contig197, whole genome shotgun sequence	fungi'
CCJE01118240.1 Vitis cinerea x Vitis riparia genome assembly BoeWGS1.0, contig BoeWGS1.0_c488, whole genome shotgun sequence	plants'
CYGV01000001.1 Rhizoctonia solani genome assembly AG22IIIB, scaffold RSOLAG22IIIB_scaffold00001, whole genome shotgun sequence	fungi'
JXCE01000001.1 Fusarium langsethiae strain Fl201059 Fl201059_contig1, whole genome shotgun sequence	fungi'
CM003473.1 Homo sapiens isolate CMT-001 chromosome 2, whole genome shotgun sequence	vertebrates_mammals'
LIDP01000001.1 Sus scrofa breed Goettingen chromosome 1, whole genome shotgun sequence	vertebrates_mammals'
CP011455.1 Mycobacterium bovis strain BCG-1 genome	bacteria'
LGTL01000001.1 Leptomonas pyrrhocoris isolate H10 LpyrH10_01, whole genome shotgun sequence	protozoa'
CP011946.1 Xanthomonas campestris pv. campestris strain 17 genome	bacteria'
LIHB01000001.1 Mycosphaerella arachidis isolate CALF-13A scaffold00001, whole genome shotgun sequence	fungi'
CM004383.1 Vibrio coralliilyticus strain RE22 chromosome 2, complete sequence, whole genome shotgun sequence	bacteria'
KQ459606.1 Papilio xuthus isolate ya'a_city_454_Px unplaced genomic scaffold scaffold4, whole genome shotgun sequence	invertebrates'
KQ461108.1 Papilio machaon isolate ya'a_city_454_Pm unplaced genomic scaffold scaffold1, whole genome shotgun sequence	invertebrates'
CM003540.1 Saccharomyces sp. 'boulardii' strain biocodex chromosome IV, whole genome shotgun sequence	fungi'
CP012036.1 Nostoc piscinale CENA21 genome	bacteria'
CM003583.1 Saccharomyces eubayanus strain FM1318 chromosome II, whole genome shotgun sequence	fungi'
LJSK01000001.1 Leptomonas seymouri strain ATCC 30220 Lsey_0001, whole genome shotgun sequence	protozoa'
LIYB01000001.1 Trametes hirsuta strain 072 scaffold00001, whole genome shotgun sequence	fungi'
LJJJ01000001.1 Trametes sp. AH28-2 contig_1, whole genome shotgun sequence	fungi'
CP012609.1 Oryza sativa Indica Group cultivar RP Bio-226 chromosome 1 sequence	plants'
LHCL01000001.1 Talaromyces verruculosus strain TS63-9 Scaffold1, whole genome shotgun sequence	fungi'
LKHI01000010.1 Cryptosporidium hominis strain UKH4 scf_01, whole genome shotgun sequence	protozoa'
LKCJ01000010.1 Cryptosporidium parvum strain UKP8 scf_01, whole genome shotgun sequence	protozoa'
LKHJ01000010.1 Cryptosporidium hominis strain UKH5 scf_01, whole genome shotgun sequence	protozoa'
LKHM01000010.1 Cryptosporidium parvum strain UKP4 scf_01, whole genome shotgun sequence	protozoa'
LKHN01000010.1 Cryptosporidium parvum strain UKP5 scf_01, whole genome shotgun sequence	protozoa'
LKHK02000010.1 Cryptosporidium parvum strain UKP2 scf_1, whole genome shotgun sequence	protozoa'
LKHL01000011.1 Cryptosporidium parvum strain UKP3 scf_02, whole genome shotgun sequence	protozoa'
LDPK01000189.1 Neonectria ditissima isolate RS 305p N305S1, whole genome shotgun sequence	fungi'
LDPL01000040.1 Neonectria ditissima isolate RS 324p N324S1, whole genome shotgun sequence	fungi'
JREZ01000001.1 Equus asinus breed Guanzhong donkey isolate Maral har scaffold1, whole genome shotgun sequence	vertebrates_mammals'
LIPI01003878.1 Myodes glareolus isolate BV_1_Jyvaskyla scaffold3892, whole genome shotgun sequence	vertebrates_mammals'
LIPJ01003348.1 Apodemus sylvaticus isolate WM_1_Wirral scaffold3351, whole genome shotgun sequence	vertebrates_mammals'
LIQJ01002592.1 Microtus agrestis isolate FV_M396_Kielder scaffold2597, whole genome shotgun sequence	vertebrates_mammals'
LKCK01000010.1 Cryptosporidium parvum strain UKP6 scf_01, whole genome shotgun sequence	protozoa'
LKCL01000010.1 Cryptosporidium parvum strain UKP7 scf_01, whole genome shotgun sequence	protozoa'
LKBE01000025.1 Aspergillus westerdijkiae strain CBS 112803 scaffold27, whole genome shotgun sequence	fungi'
LJBI01000005.1 Yarrowia lipolytica strain W29 chromosome E, whole genome shotgun sequence	fungi'
LJRW01000034.1 Cryptosporidium hominis isolate UKH3 scf_001, whole genome shotgun sequence	protozoa'
LHSL01000001.1 Penicillium digitatum PDC 102 PDC1021, whole genome shotgun sequence	fungi'
LHTN01002440.1 Macrophomina phaseolina strain MO00014 NODE_9, whole genome shotgun sequence	fungi'
LHTM01004081.1 Macrophomina phaseolina strain MP00003 NODE_1, whole genome shotgun sequence	fungi'
LHTP01003651.1 Macrophomina phaseolina strain MP00325 NODE_7, whole genome shotgun sequence	fungi'
LHTO01004223.1 Macrophomina phaseolina strain MP00065 NODE_5, whole genome shotgun sequence	fungi'
LHTQ01000491.1 Macrophomina phaseolina strain MP00327 NODE_6, whole genome shotgun sequence	fungi'
CP010988.1 Carica papaya chromosome Y sequence	plants'
KQ468087.1 Dinoponera quadriceps isolate SergipeBrazil2010 unplaced genomic scaffold scaffold566, whole genome shotgun sequence	invertebrates'
KQ465187.1 Polistes canadensis isolate GaletaPanama2010 unplaced genomic scaffold scaffold_0, whole genome shotgun sequence	invertebrates'
LGSM01000001.1 Phytophthora multivora isolate NZFS 3378 scf_21718_1.contig_1, whole genome shotgun sequence	protozoa'
LGSL01000001.1 Phytophthora multivora isolate NZFS 3448 scf_21858_1.contig_1, whole genome shotgun sequence	protozoa'
LGSJ01000006.1 Phytophthora cinnamomi isolate MP94-48 scf_37789_6.contig_1, whole genome shotgun sequence	protozoa'
LGSN01000008.1 Phytophthora taxon totara isolate NZFS 3642 scf_38044_8.contig_1, whole genome shotgun sequence	protozoa'
LGTU01000004.1 Phytophthora pluvialis isolate LC9-1 scf_62525_3.contig_1, whole genome shotgun sequence	protozoa'
LGTR01000003.1 Phytophthora agathidicida isolate NZFS 3772 scf_22787_3.contig_1, whole genome shotgun sequence	protozoa'
LGTS01000003.1 Phytophthora agathidicida isolate NZFS 3770 scf_22940_3.contig_1, whole genome shotgun sequence	protozoa'
LGTT01000001.1 Phytophthora pluvialis isolate NZFS 3000 scf_62265_1.contig_1, whole genome shotgun sequence	protozoa'
LGSK01000001.1 Phytophthora cinnamomi isolate NZFS 3750 scf_37547_1.contig_1, whole genome shotgun sequence	protozoa'
LGSO01000001.1 Phytophthora taxon totara isolate NZFS 3727 scf_38357_1.contig_1, whole genome shotgun sequence	protozoa'
LJPC01000001.1 Rotaria magnacalcarata isolate TS_RM01 R_mag_scaffold_1, whole genome shotgun sequence	invertebrates'
CP010059.1 Enterococcus cecorum strain CE1 genome	bacteria'
CP010060.1 Enterococcus cecorum strain SA1 genome	bacteria'
CP010061.1 Enterococcus cecorum strain SA2 genome	bacteria'
CP010062.1 Enterococcus cecorum strain CE2 genome	bacteria'
CP010063.1 Enterococcus cecorum strain CE3 genome	bacteria'
CP010064.1 Enterococcus cecorum strain SA3 genome	bacteria'
KQ474073.1 Rhodotorula graminis WP1 unplaced genomic scaffold RHOBAscaffold_1, whole genome shotgun sequence	fungi'
CENE01000001.1 Sporidiobolus salmonicolor genome assembly SPOSA68321A01, scaffold scaff000, whole genome shotgun sequence	fungi'
CCBN010000001.1 Galactomyces candidum WGS project CCBN00000000 data, scaffold01, whole genome shotgun sequence	fungi'
LN609233.1 Leishmania peruviana genome assembly Leishmania peruviana PAB-4377_V1, chromosome : 35	protozoa'
LN609196.1 Leishmania peruviana genome assembly Leishmania peruviana LEM-1537_V1, chromosome : 35	protozoa'
LN881072.1 Primula vulgaris genome assembly Primula_Slocus, scaffold PvSloc001_scaffold_213, whole genome shotgun sequence	plants'
LN877770.1 Escherichia coli genome assembly ERS742059, chromosome : I	bacteria'
HG975440.1 Solanum pennellii chromosome ch01, complete genome	plants'
JRUX01001409.1 Kudoa iwatai isolate KIW-1.11.2010, whole genome shotgun sequence	invertebrates'
JRUY01005471.1 Kudoa iwatai isolate KIW-1.11.2010, whole genome shotgun sequence	invertebrates'
LIHL01055726.1 Juglans regia cultivar Chandler jcf7180001222233, whole genome shotgun sequence	plants'
LHUT01000002.1 Silene latifolia subsp. alba cultivar K-line scaffold2.1, whole genome shotgun sequence	plants'
LJCH01000020.1 Nicrophorus vespilloides isolate i.1.1-1 Super_scaffold_19, whole genome shotgun sequence	invertebrates'
CP013020.1 Bacteroides vulgatus strain mpk genome	bacteria'
KQ476056.1 Diachasma alloeum isolate Michigan unplaced genomic scaffold Dall_scaffold_00176, whole genome shotgun sequence	invertebrates'
CP012092.1 Francisella tularensis strain T01 genome	bacteria'
CM003560.1 Saccharomyces sp. 'boulardii' strain unique28 chromosome IV, whole genome shotgun sequence	fungi'
KQ475712.1 Hyaloperonospora arabidopsidis isolate Cala2 unplaced genomic scaffold scf_11684_19, whole genome shotgun sequence	protozoa'
LLKM01000001.1 Hyaloperonospora arabidopsidis isolate Noks1 scf_59133_1.contig_1, whole genome shotgun sequence	protozoa'
CP013032.1 Campylobacter coli strain CO2-160 genome	bacteria'
CP013036.1 Campylobacter coli strain CF2-75 genome	bacteria'
AP014599.1 Kluyveromyces marxianus DNA, chromosome 1, complete genome, strain: NBRC 1777	fungi'
AP012213.1 Kluyveromyces marxianus DMKU3-1042 DNA, complete genome, chromosome 1	fungi'
LJWW01000002.1 Exaiptasia pallida isolate CC7 scaffold2, whole genome shotgun sequence	invertebrates'
LMAW01000001.1 Amazona aestiva isolate FVVF132 scaffold_0, whole genome shotgun sequence	vertebrates_non_mammals'
LAQG01000458.1 Micromonas sp. ASP10-01a contig3690, whole genome shotgun sequence	plants'
KQ481983.1 Chlorella pyrenoidosa strain FACHB-9 unplaced genomic scaffold scaffold00366, whole genome shotgun sequence	plants'
LJPH01000577.1 Byssoonygena ceratinophila isolate UAMH 5669 scaffold553, whole genome shotgun sequence	fungi'
LJPJ01000294.1 Amauroascus mutatus isolate UAMH 3576 scaffold20, whole genome shotgun sequence	fungi'
LJPK01000179.1 Amauroascus niger isolate UAMH 3544 scaffold107, whole genome shotgun sequence	fungi'
LJPI01000825.1 Chrysosporium queenslandicum isolate CBS 280.77 scaffold794, whole genome shotgun sequence	fungi'
KQ483353.1 Calidris pugnax isolate Ruff unplaced genomic scaffold scaffold44, whole genome shotgun sequence	vertebrates_non_mammals'
CP013116.1 Campylobacter jejuni strain T1-21 genome	bacteria'
CP013111.1 Bordetella sp. N genome	bacteria'
AP014957.1 Oryza sativa Japonica Group DNA, chromosome 1, cultivar: Nipponbare, complete sequence	plants'
CM003601.1 Streptomyces anulatus strain ATCC 11523 chromosome, whole genome shotgun sequence	bacteria'
CM003594.1 Psychrobacter sp. P11F6 chromosome, whole genome shotgun sequence	bacteria'
CM003596.1 Psychrobacter sp. P11G3 chromosome, whole genome shotgun sequence	bacteria'
CM003641.1 Wolbachia endosymbiont of Trichogramma pretiosum strain wTpre chromosome, whole genome shotgun sequence	bacteria'
CP013140.1 Lysobacter enzymogenes strain C3 genome	bacteria'
JANR01000001.1 Apis cerana strain Korean Scaffold_1, whole genome shotgun sequence	invertebrates'
LMZO01000001.1 [Candida] boidinii strain GF002 contig_1, whole genome shotgun sequence	fungi'
KQ503367.1 Puccinia psidii isolate Aus_3 unplaced genomic scaffold scaffold_1, whole genome shotgun sequence	fungi'
KQ545315.1 Poecilia latipinna isolate Pla-1442-1.1 unplaced genomic scaffold Scaffold0, whole genome shotgun sequence	vertebrates_non_mammals'
CP013147.1 Leptospira interrogans serovar Hardjo-prajitno strain Hardjoprajitno chromosome 1 sequence	bacteria'
KQ550984.1 Poecilia mexicana isolate Pme_1353_1.1 unplaced genomic scaffold Scaffold0, whole genome shotgun sequence	vertebrates_non_mammals'
KQ556858.1 Xiphophorus hellerii strain Sarabia unplaced genomic scaffold Scaffold24, whole genome shotgun sequence	vertebrates_non_mammals'
LLWD01000124.1 Acinonyx jubatus isolate AJU 981 Chewbacca scaffold71, whole genome shotgun sequence	vertebrates_mammals'
KQ560120.1 Aedes albopictus isolate Foshan unplaced genomic scaffold scaffold1, whole genome shotgun sequence	invertebrates'
KQ557225.1 Xiphophorus couchianus unplaced genomic scaffold Scaffold24, whole genome shotgun sequence	vertebrates_non_mammals'
DF967598.1 Rosellinia necatrix DNA, scaffold: rne01sc001, whole genome shotgun sequence	fungi'
LMTP010010475.1 Pinus lambertiana isolate USFS_5038 super10479, whole genome shotgun sequence	plants'
LJJS01001513.1 Diaporthe aspalathi isolate MS-SSC91 scaffold_2492, whole genome shotgun sequence	fungi'

KQ728331.1 Sturnus vulgaris isolate 715 unplaced genomic scaffold Scaffold0, whole genome shotgun sequence	vertebrates_non_mammals'
JYDJ01000001.1 Trichinella murrelli isolate ISS417 scaffold1s, whole genome shotgun sequence	invertebrates'
JYDK01000001.1 Trichinella sp. T6 isolate ISS34 scaffold1s, whole genome shotgun sequence	invertebrates'
JYDU01000001.1 Trichinella pseudospiralis isolate ISS141 scaffold1s, whole genome shotgun sequence	invertebrates'
JYDL01000001.1 Trichinella nelsoni isolate ISS37 scaffold1s, whole genome shotgun sequence	invertebrates'
JYDN01000001.1 Trichinella sp. T9 isolate ISS409 scaffold1s, whole genome shotgun sequence	invertebrates'
LDAU01000110.1 Pseudocohnilembus persalinus isolate 36N120E scaffold2116, whole genome shotgun sequence	protozoa'
JYDW01000001.1 Trichinella nativa isolate ISS10 scaffold1s, whole genome shotgun sequence	invertebrates'
JYDT01000001.1 Trichinella pseudospiralis isolate ISS470 (USA) scaffold1s, whole genome shotgun sequence	invertebrates'
JYDI01000001.1 Trichinella britovi isolate ISS120 scaffold1s, whole genome shotgun sequence	invertebrates'
JYDH01000001.1 Trichinella spiralis isolate ISS3 (POL-1) scaffold1s, whole genome shotgun sequence	invertebrates'
JYDV01000001.1 Trichinella pseudospiralis isolate ISS176 (KAZ) scaffold1s, whole genome shotgun sequence	invertebrates'
JYDQ01000001.1 Trichinella patagoniensis isolate ISS2496 scaffold1s, whole genome shotgun sequence	invertebrates'
JYDP01000001.1 Trichinella zimbabwensis isolate ISS1029 scaffold1s, whole genome shotgun sequence	invertebrates'
JYDR01000001.1 Trichinella pseudospiralis isolate ISS13 (GAR) scaffold1s, whole genome shotgun sequence	invertebrates'
JYDS01000001.1 Trichinella pseudospiralis isolate ISS588 (KAM) scaffold1s, whole genome shotgun sequence	invertebrates'
JYDM01000001.1 Trichinella sp. T8 isolate ISS272 scaffold1s, whole genome shotgun sequence	invertebrates'
JYDO01000001.1 Trichinella papuae isolate ISS1980 scaffold1s, whole genome shotgun sequence	invertebrates'
KQ748387.1 Gekko japonicus isolate JY-2015 unplaced genomic scaffold scaffold1, whole genome shotgun sequence	vertebrates_non_mammals'
LMYF01000001.1 Hypsibius dujardini isolate Chapel Hill scaffold1, whole genome shotgun sequence	invertebrates'
CP013328.1 Fusobacterium nucleatum subsp. polymorphum strain ChDC F319 genome	bacteria'
LDMZ01012624.1 Sphaeromyxa zaharoni isolate 24.2.2011, whole genome shotgun sequence	invertebrates'
LDNA01065285.1 Enteromyxum leei isolate 2.6.2011, whole genome shotgun sequence	invertebrates'
CP013048.1 Escherichia coli strain RS76 genome	bacteria'
LDEF01000001.1 Leptographium lundbergii strain CBS 138716 Scaffold_1, whole genome shotgun sequence	fungi'
JXRH01010416.1 Ambystoma mexicanum strain laboratory chromosome 13 AM13_scaffold10417, whole genome shotgun sequence	vertebrates_non_mammals'
LKTN01099830.1 Cottus rhenanus strain Naaf scaffold4162, whole genome shotgun sequence	vertebrates_non_mammals'
LAEG01000507.1 Diplodia scrobiculata strain CMW30223 contig508, whole genome shotgun sequence	fungi'
LNDK02000066.1 Paecilomyces hepiali strain FENG scaffold1, whole genome shotgun sequence	fungi'
LKER01000001.1 Rhodotorula toruloides strain CGMCC 2.1609 scaff_414a, whole genome shotgun sequence	fungi'
CVKU01000001.1 Heterobasidion annosum genome assembly 137OC, scaffold PseudoContig_scaffold_01, whole genome shotgun sequence	fungi'
CVKY01000001.1 Heterobasidion irregulare genome assembly 48NB, scaffold PseudoContig_scaffold_01, whole genome shotgun sequence	fungi'
CVKW01000001.1 Heterobasidion irregulare genome assembly 9OA, scaffold PseudoContig_scaffold_01, whole genome shotgun sequence	fungi'
CVKZ01000001.1 Heterobasidion annosum genome assembly BM42NG, scaffold PseudoContig_scaffold_01, whole genome shotgun sequence	fungi'
CVKV01000001.1 Heterobasidion annosum genome assembly 109SA, scaffold PseudoContig_scaffold_01, whole genome shotgun sequence	fungi'
CVKX01000001.1 Heterobasidion irregulare genome assembly 49SA, scaffold PseudoContig_scaffold_01, whole genome shotgun sequence	fungi'
CZLE01000094.1 Calidris pugnax genome assembly ruff_EG1, scaffold Contig93, whole genome shotgun sequence	vertebrates_non_mammals'
LN899469.1 Idiomarinaceae bacterium HL-53 genome assembly JGI assembly, chromosome : I	bacteria'
CZRN01000001.1 Marmota marmota marmota genome assembly marMar2.1, scaffold chr2_part1, whole genome shotgun sequence	vertebrates_mammals'
JRLE01000001.1 Cimex lectularius isolate BBFD1 CLS00002, whole genome shotgun sequence	invertebrates'
LN906597.1 bacterium 2013Arg42i genome assembly Ark11, chromosome : I	bacteria'
CM003626.1 Burkholderia sp. MSMB122 chromosome 1, complete sequence, whole genome shotgun sequence	bacteria'
KQ758850.1 Aurantiochytrium sp. T66 unplaced genomic scaffold Scaffold_0001, whole genome shotgun sequence	protozoa'
CP012043.1 Klebsiella pneumoniae strain U25 genome	bacteria'
LD344849.1 Ptychodera flava DNA, scaffold: scaffold4887_cov142, isolate: pfl_male_2006_01, whole genome shotgun sequence	invertebrates'
CP013655.1 Enterococcus rotai strain LMG 26678 genome	bacteria'
LN609383.1 Nothobranchius furzeri genome assembly Nfu_20140520, chromosome : sgr01, whole genome shotgun sequence	vertebrates_non_mammals'
LNKV01000002.1 Sclerotinia homoeocarpa strain HRI11 scaffold2, whole genome shotgun sequence	fungi'
LNGN01000003.1 Sclerotinia homoeocarpa isolate HRS10 scaffold3, whole genome shotgun sequence	fungi'
LMBU01000001.1 Polistes dominula isolate PdomPennStateAug2009 PdomSCFr1.2-0001, whole genome shotgun sequence	invertebrates'
LDJA01000001.1 Kluyveromyces marxianus strain IIPE453 Scaffold_1, whole genome shotgun sequence	fungi'
LLWO01000031.1 [Candida] glabrata isolate 1A scaffold1, whole genome shotgun sequence	fungi'
LMAA01000056.1 [Candida] glabrata isolate 1B scaffold1, whole genome shotgun sequence	fungi'
LMAB01000088.1 Candida glabrata isolate 3B scaffold17, whole genome shotgun sequence	fungi'
LLZY01000038.1 [Candida] glabrata isolate 2B scaffold14, whole genome shotgun sequence	fungi'
LLZZ01000106.1 [Candida] glabrata isolate 2A scaffold1, whole genome shotgun sequence	fungi'
LMAY01000112.1 Candida glabrata isolate 3A scaffold2, whole genome shotgun sequence	fungi'
LATX01001986.1 Moniliophthora roreri strain MCA 2952 kmer90.96428, whole genome shotgun sequence	fungi'
LOCP02000001.1 Rousettus aegyptiacus isolate 1219 scaffold1, whole genome shotgun sequence	vertebrates_mammals'
CP012350.1 Propionibacterium acnes strain PA_30_2_L1, complete genome	bacteria'
CP012353.1 Propionibacterium acnes strain PA_12_1_R1, complete genome	bacteria'
CP014258.1 Mycobacterium fortuitum subsp. fortuitum DSM 46621 = ATCC 6841 genome	bacteria'
LNZH02000216.1 Sanghuangporus baumii strain 821 scaffold_46214, whole genome shotgun sequence	fungi'
CP013693.1 Propionibacterium acnes strain A1-14 genome	bacteria'
JPDN01000001.1 Trichoderma gamsii strain T6085 Scaffolds0001.1, whole genome shotgun sequence	fungi'
LNFP01000118.1 Phytophthora nicotianae strain race 1 scaffold5, whole genome shotgun sequence	protozoa'
LNFO01005619.1 Phytophthora nicotianae strain race 0 contig5619, whole genome shotgun sequence	protozoa'
LKPD01008108.1 Danio rerio strain CG2 CG2_v1.0_scaffold9679, whole genome shotgun sequence	vertebrates_non_mammals'
LCZG01000002.1 Huntiella savannae strain CBS 121151 Contig_0000002, whole genome shotgun sequence	fungi'
LOQJ01000824.1 Saccharomyces pastorianus strain Hybrid yeast 1 sequence_920, whole genome shotgun sequence	fungi'
LOQK01000001.1 Saccharomyces pastorianus strain Hybrid yeast 7 sequence_1, whole genome shotgun sequence	fungi'
JTAI01000007.1 Cryptosporidium hominis isolate 30976 contig_3, whole genome shotgun sequence	protozoa'
LOQL01007207.1 Homo sapiens Scv5G3v_35, whole genome shotgun sequence	vertebrates_mammals'
LOQM01002472.1 Homo sapiens ScAWZkm_38, whole genome shotgun sequence	vertebrates_mammals'
LPXP01000079.1 Homo sapiens Scaffold1549, whole genome shotgun sequence	vertebrates_mammals'
LOEK01000371.1 Homo sapiens ScbYBnM_46, whole genome shotgun sequence	vertebrates_mammals'
KQ760267.1 Eufriesea mexicana isolate 0111107269 unplaced genomic scaffold scaffold124, whole genome shotgun sequence	invertebrates'
LPVI01001605.1 Colletotrichum falcatum strain Cf671 transcript_1605, whole genome shotgun sequence	fungi'
LQCJ01000003.1 Gorilla gorilla gorilla isolate KB3781 chromosome Y Contig2, whole genome shotgun sequence	vertebrates_mammals'
LNCG01249645.1 Arabis nordmanniana contig_797, whole genome shotgun sequence	plants'
FAPD01000289.1 TPA: Heliconius cydno cordula genome assembly Heliconius_cydno_cordula.M2255, contig 110329_0009_707UEAAXX_7_GP-PE-008_1_(paired)_contig_289, whole genome shotgun sequence	invertebrates'
FAPB01000227.1 TPA: Heliconius ismenius metaphorus genome assembly Heliconius_ismenius_metaphorus.mj10_9995, contig 110414_0050_70JE0AAXX_2_GP-PE-008_1_(paired)_contig_227, whole genome shotgun sequence	invertebrates'
FAPA01000735.1 TPA: Heliconius cydno cordula genome assembly Heliconius_cydno_cordula.M2258, contig 110329_0009_707UEAAXX_7_GP-PE-007_1_(paired)_contig_735, whole genome shotgun sequence	invertebrates'
FAPC01000682.1 TPA: Heliconius cydno cordula genome assembly Heliconius_cydno_cordula.M2253, contig 110329_0009_707UEAAXX_7_GP-PE-004_1_(paired)_contig_682, whole genome shotgun sequence	invertebrates'
FAPI01001401.1 TPA: Heliconius ethilla aerotome genome assembly Heliconius_ethilla_aerotome.09-63, contig Ethilla_09-36_1.94_(paired)_contig_1401, whole genome shotgun sequence	invertebrates'
FARF01000010.1 TPA: Heliconius melpomene amaryllis genome assembly Heliconius_melpomene_amaryllis.09-75, contig Amaryllis_09-75_1.94_(paired)_contig_10, whole genome shotgun sequence	invertebrates'
FAPL01003617.1 TPA: Heliconius ethilla genome assembly Heliconius_ethilla.JM-09-49, contig Hel_8_GCCAAT_L001_R1_001_(paired)_contig_3617, whole genome shotgun sequence	invertebrates'
FAPO01008990.1 TPA: Heliconius ethilla genome assembly Heliconius_ethilla.JM-09-62, contig HEL_2_CAGATC_L002_R1_001_(paired)_contig_8990, whole genome shotgun sequence	invertebrates'
FAPE01000146.1 TPA: Heliconius cydno cordula genome assembly Heliconius_cydno_cordula.M2157, contig 110329_0009_707UEAAXX_7_GP-PE-012_1_(paired)_contig_146, whole genome shotgun sequence	invertebrates'
FAQM01000005.1 TPA: Heliconius melpomene aglaope genome assembly Heliconius_melpomene_aglaope.09-246, contig Aglaope_09-246_1.94_(paired)_contig_5, whole genome shotgun sequence	invertebrates'
FAPR01008863.1 TPA: Heliconius ethilla genome assembly Heliconius_ethilla.JM-09-66, contig HEL_2_CTTGTA_L002_R1_001_(paired)_contig_8863, whole genome shotgun sequence	invertebrates'
FAQR01000800.1 TPA: Heliconius melpomene aglaope genome assembly Heliconius_melpomene_aglaope.09-267, contig Aglaope_09-267_1.94_(paired)_contig_800, whole genome shotgun sequence	invertebrates'
FARD01000157.1 TPA: Heliconius melpomene amaryllis genome assembly Heliconius_melpomene_amaryllis.09-332, contig Amaryllis_09-332_1.94_(paired)_contig_157, whole genome shotgun sequence	invertebrates'
FAPP01001005.1 TPA: Heliconius ismenius genome assembly Heliconius_ismenius.STRI_003, contig STRI_003_(paired)_contig_1005, whole genome shotgun sequence	invertebrates'
FAPJ01010847.1 TPA: Heliconius ethilla aerotome genome assembly Heliconius_ethilla_aerotome.09-67, contig Hel_8_CGATGT_L001_R1_001_(paired)_contig_10847, whole genome shotgun sequence	invertebrates'
FAQN01000035.1 TPA: Heliconius melpomene aglaope genome assembly Heliconius_melpomene_aglaope.09-357, contig Aglaope_09-357_1.94_(paired)_contig_35, whole genome shotgun sequence	invertebrates'
FARH01000302.1 TPA: Heliconius melpomene amaryllis genome assembly Heliconius_melpomene_amaryllis.09-79, contig Amaryllis_09-79_1.94_(paired)_contig_302, whole genome shotgun sequence	invertebrates'
FAQQ01000008.1 TPA: Heliconius melpomene aglaope genome assembly Heliconius_melpomene_aglaope.09-268, contig Aglaope_09-268_1.94_(paired)_contig_8, whole genome shotgun sequence	invertebrates'
FAPG01026250.1 TPA: Heliconius hecale felix genome assembly Heliconius_hecale_felix.09-273, contig Hel_9_CGATGT_L002_R1_001_(paired)_contig_26250, whole genome shotgun sequence	invertebrates'
FAPH01000113.1 TPA: Heliconius elevatus genome assembly Heliconius_elevatus.09-343, contig Elevatus_09-343_1.94_(paired)_contig_113, whole genome shotgun sequence	invertebrates'
FAPK01003260.1 TPA: Heliconius melpomene aglaope genome assembly Heliconius_melpomene_aglaope.CAM009998, contig heliconius_agalope_eas034_IWG_HELCOM.H._agalope-1_1pA_9_10_10_s_6_1_sequence_(paired)_contig_3260, whole genome shotgun sequence	invertebrates'
FAPF01000270.1 TPA: Heliconius hecale felix genome assembly Heliconius_hecale_felix.09-345, contig Hecale_09-345_1.94_(paired)_contig_270, whole genome shotgun sequence	invertebrates'
FARE01000088.1 TPA: Heliconius melpomene amaryllis genome assembly Heliconius_melpomene_amaryllis.09-333, contig Amaryllis_09-333_1.94_(paired)_contig_88, whole genome shotgun sequence	invertebrates'
FATR01000006.1 TPA: Heliconius timareta ssp. JD-2011 genome assembly Heliconius_timareta_thelxinoe.8624, contig 110414_0050_70JE0AAXX_1_GP-PE-012_1_(paired)_contig_6, whole genome shotgun sequence	invertebrates'
FATD01000536.1 TPA: Heliconius timareta timareta genome assembly Heliconius_timareta_timareta_cont.8523, contig 110414_0050_70JE0AAXX_1_GP-PE-008_1_(paired)_contig_536, whole genome shotgun sequence	invertebrates'
FASU01003512.1 TPA: Heliconius pardalinus sergestus genome assembly Heliconius_pardalinus_sergestus.09-202, contig Hel_8_TGACCA_L001_R1_001_(paired)_contig_3512, whole genome shotgun sequence	invertebrates'
FASN01000033.1 TPA: Heliconius numata silvana genome assembly Heliconius_numata_silvana.09-364, contig Numata_09-364_1.94_(paired)_contig_33, whole genome shotgun sequence	invertebrates'
FATF01000442.1 TPA: Heliconius timareta ssp. NG-2008 genome assembly Heliconius_timareta_florencia.2403, contig 110329_0009_707UEAAXX_6_GP-PE-004_1_(paired)_contig_442, whole genome shotgun sequence	invertebrates'
FASR01000003.1 TPA: Heliconius pardalinus sergestus genome assembly Heliconius_pardalinus_sergestus.09-326, contig Pardalinus_sergestus_09-326_1.94_(paired)_contig_3, whole genome shotgun sequence	invertebrates'
FATN01000024.1 TPA: Heliconius timareta genome assembly Heliconius_timareta.2409, contig 110329_0009_707UEAAXX_5_GP-PE-007_1_(paired)_contig_24, whole genome shotgun sequence	invertebrates'
FASX01004263.1 Heliconius pardalinus sergestus genome assembly Heliconius_pardalinus_sergestus.JM-09-210, contig HEL_5_GCCAAT_L006_R1_001_(paired)_contig_4263, whole genome shotgun sequence	invertebrates'
FATU01000154.1 TPA: Heliconius timareta ssp. JD-2011 genome assembly Heliconius_timareta_thelxinoe.8631, contig 110414_0050_70JE0AAXX_2_GP-PE-007_1_(paired)_contig_154, whole genome shotgun sequence	invertebrates'
FATX01000033.1 TPA: Heliconius timareta timareta genome assembly Heliconius_timareta_timareta.9184, contig 110414_0050_70JE0AAXX_1_GP-PE-007_1_(paired)_contig_33, whole genome shotgun sequence	invertebrates'
FATW01000149.1 TPA: Heliconius timareta timareta genome assembly Heliconius_timareta_timareta.8533, contig 110414_0050_70JE0AAXX_1_GP-PE-004_1_(paired)_contig_149, whole genome shotgun sequence	invertebrates'
FATP01000690.1 TPA: Heliconius timareta genome assembly Heliconius_timareta.2435, contig 110329_0009_707UEAAXX_5_GP-PE-012_1_(paired)_contig_690, whole genome shotgun sequence	invertebrates'
FATM01000556.1 TPA: Heliconius timareta genome assembly Heliconius_timareta.2234, contig 110329_0009_707UEAAXX_5_GP-PE-004_1_(paired)_contig_556, whole genome shotgun sequence	invertebrates'
FATE01000309.1 TPA: Heliconius timareta timareta genome assembly Heliconius_timareta_timareta_cont.8520, contig Timareta_Ecuador_8520_1.94_(paired)_contig_309, whole genome shotgun sequence	invertebrates'
FATK01000837.1 TPA: Heliconius timareta ssp. NG-2008 genome assembly Heliconius_timareta_florencia.2407, contig 110329_0009_707UEAAXX_6_GP-PE-008_1_(paired)_contig_837, whole genome shotgun sequence	invertebrates'
FATS01000460.1 TPA: Heliconius timareta ssp. JD-2011 genome assembly Heliconius_timareta_thelxinoe.8628, contig 110414_0050_70JE0AAXX_2_GP-PE-004_1_(paired)_contig_460, whole genome shotgun sequence	invertebrates'
FATJ01000037.1 TPA: Heliconius timareta ssp. NG-2008 genome assembly Heliconius_timareta_florencia.2406, contig 110329_0009_707UEAAXX_6_GP-PE-007_1_(paired)_contig_37, whole genome shotgun sequence	invertebrates'
FATB01000048.1 TPA: Heliconius pardalinus ssp. JD-2011 genome assembly Heliconius_pardalinus_ssp._JD-2011.09-387, contig Pardalinus_ssp_09-387_1.94_(paired)_contig_48, whole genome shotgun sequence	invertebrates'
FASW01004603.1 TPA: Heliconius pardalinus sergestus genome assembly Heliconius_pardalinus_sergestus.JM-09-209, contig HEL_4_ACAGTG_L005_R1_001_(paired)_contig_4603, whole genome shotgun sequence	invertebrates'
FATL01000196.1 TPA: Heliconius timareta ssp. NG-2008 genome assembly Heliconius_timareta_florencia.2410, contig 110329_0009_707UEAAXX_6_GP-PE-012_1_(paired)_contig_196, whole genome shotgun sequence	invertebrates'
FATQ01001197.1 TPA: Heliconius timareta ssp. JD-2011 genome assembly Heliconius_timareta_thelxinoe.09-312, contig Timareta_Peru_09-312_1.94_(paired)_contig_1197, whole genome shotgun sequence	invertebrates'
FATO01000387.1 TPA: Heliconius timareta genome assembly Heliconius_timareta.2434, contig 110329_0009_707UEAAXX_5_GP-PE-008_1_(paired)_contig_387, whole genome shotgun sequence	invertebrates'
FASM01000487.1 TPA: Heliconius numata arcuella genome assembly Heliconius_numata_arcuella.05-1277, contig 110414_0050_70JE0AAXX_2_GP-PE-012_1_(paired)_contig_487, whole genome shotgun sequence	invertebrates'
FASP01000205.1 TPA: Heliconius numata tarapotensis genome assembly Heliconius_numata_tarapotensis.05-1358, contig 110414_0050_70JE0AAXX_2_GP-PE-001_1_(paired)_contig_205, whole genome shotgun sequence	invertebrates'
FASV01013459.1 TPA: Heliconius pardalinus sergestus genome assembly Heliconius_pardalinus_sergestus.JM-09-201, contig HEL_4_CGATGT_L005_R1_001_(paired)_contig_13459, whole genome shotgun sequence	invertebrates'
LN930111.1 Danio rerio genome assembly Zebrafish Genome Assembly WGS32, scaffold zfish-wgs32_202, whole genome shotgun sequence	vertebrates_non_mammals'
LN606820.1 Xanthomonas citri pv. citri genome assembly ASX_PRJEB7163_v1, chromosome : XAC40, whole genome shotgun sequence	bacteria'
LN590509.1 Xanthomonas citri pv. citri genome assembly ASX_PRJEB7164_v1, chromosome : XACJK, whole genome shotgun sequence	bacteria'
LN609302.1 Acetobacter ghanensis genome assembly Acetobacter ghanensis, chromosome : I	bacteria'
LOQP01008121.1 Homo sapiens Scaffold8940, whole genome shotgun sequence	vertebrates_mammals'
LOQO01006935.1 Homo sapiens ScmjKUh_39, whole genome shotgun sequence	vertebrates_mammals'
KQ947404.1 Phialocephala scopiformis strain CBS 120377 unplaced genomic scaffold LY89scaffold_1, whole genome shotgun sequence	fungi'
LKUB01159003.1 Cannabis sativa cultivar Chemdog91 contig22461, whole genome shotgun sequence	plants'
LNGL01000005.1 Knoxdaviesia proteae strain CMW40885 scaffold5, whole genome shotgun sequence	fungi'
LNGK01000008.1 Knoxdaviesia capensis strain CMW40890 scaffold8, whole genome shotgun sequence	fungi'
FAPM01000036.1 Heliconius cydno hermogenes genome assembly Heliconius_cydno_hermogenes.CS003278, contig 120315_0312_D0T4UACXX_1_SA-PE-083_1_(paired)_contig_36, whole genome shotgun sequence	invertebrates'
FARO01000912.1 Heliconius melpomene malleti genome assembly Heliconius_melpomene_malleti.CAM017162, contig malletiF.17162_[malletiF.17162]_(paired)_un-mapped_reads_contig_912, whole genome shotgun sequence	invertebrates'
CZLF01000286.1 Ramularia collo-cygni genome assembly DK05_1.1, contig contig_37, whole genome shotgun sequence	fungi'
CDMC01000001.1 Aspergillus calidoustus genome assembly Acal_Allpaths_LG, scaffold SCAF1, whole genome shotgun sequence	fungi'
LN998033.1 Brucella sp. F965 genome assembly BVF965, chromosome : 1	bacteria'
LNBV01021943.1 Oscheius sp. TEL-2014 NODE_75240_length_146261_cov_23.683867, whole genome shotgun sequence	invertebrates'
LJZU01000033.1 Davidsoniella virescens strain CMW17339 CMW17339_contig_35, whole genome shotgun sequence	fungi'

LJOA01000070.1 Ceratocystis eucalypticola strain CMW9998 contig_82, whole genome shotgun sequence	fungi'
LJDD01001638.1 Chrysoporthe deuterocubensis strain CMW 8650 scaffold_1639, whole genome shotgun sequence	fungi'
LJGR01000001.1 Fusarium temperatum strain CMWF389 Scaffold1, whole genome shotgun sequence	fungi'
LKBB01000001.1 Thielaviopsis musarum strain CMW1546 scaffold1, whole genome shotgun sequence	fungi'
LLKO01000001.1 Graphilbum fragrans strain CBS 138720 CMW19357_scaffold_1, whole genome shotgun sequence	fungi'
LLXE01000001.1 Penicillium freii strain DAOM 242723 scaffold_1, whole genome shotgun sequence	fungi'
CM003662.1 Oryza longistaminata cultivar RD23 chromosome 3, whole genome shotgun sequence	plants'
BCMY01000001.1 Aspergillus niger DNA, contig: scaffold669_001, strain: An76, whole genome shotgun sequence	fungi'
LAVE01S013150.1 Sinocyclocheilus anshuiensis isolate Anshui scaffold283, whole genome shotgun sequence	vertebrates_non_mammals'
LAVF01S005297.1 Sinocyclocheilus rhinocerous isolate Xijiao scaffold72, whole genome shotgun sequence	vertebrates_non_mammals'
LCYQ01S000003.1 Sinocyclocheilus grahami isolate Dianchi scaffold3.1, whole genome shotgun sequence	vertebrates_non_mammals'
LPNX010160114.1 Pseudotsuga menziesii isolate Weyco1 jcf7190000012883, whole genome shotgun sequence	plants'
LOQN01000001.1 Homo sapiens flattened_line_0, whole genome shotgun sequence	vertebrates_mammals'
CM003679.1 Phaseolus vulgaris cultivar BAT93 chromosome 8, whole genome shotgun sequence	plants'
CP013656.1 Rheinheimera sp. F8 genome	bacteria'
CP013962.1 Escherichia coli strain SEC470 genome	bacteria'
LPZQ01000239.1 Fusarium oxysporum f. sp. conglutinans strain 1 cENTRYscaffold699_cov430, whole genome shotgun sequence	fungi'
CM003710.1 Parus major isolate Abel chromosome 2, whole genome shotgun sequence	vertebrates_non_mammals'
CM003683.1 Homo sapiens isolate NA19240 chromosome 1, whole genome shotgun sequence	vertebrates_mammals'
LD636966.1 Protobothrops mucrosquamatus DNA, contig: scaffold6, isolate: PMUCROS, whole genome shotgun sequence	vertebrates_non_mammals'
CM003813.1 Ananas comosus cultivar F153 linkage group 1, whole genome shotgun sequence	plants'
LPUV01000532.1 Alligator mississippiensis isolate KSC_2009_1 ScZkoYb_72, whole genome shotgun sequence	vertebrates_non_mammals'
LPUU01003284.1 Alligator mississippiensis isolate KSC_2009_1 Scaffold12379, whole genome shotgun sequence	vertebrates_non_mammals'
KQ954464.1 Rhodotorula sp. JG-1b unplaced genomic scaffold RHOSPscaffold_1, whole genome shotgun sequence	fungi'
LNKU01000001.1 Rhodotorula toruloides strain ATCC 10657 scaffold_0, whole genome shotgun sequence	fungi'
LNQQ01000001.1 Rhodotorula toruloides strain ATCC 10788 scaffold_0, whole genome shotgun sequence	fungi'
CM003707.1 Escherichia coli strain PPECC42 chromosome PPECC42, whole genome shotgun sequence	bacteria'
CP014135.1 Pseudomonas agarici strain NCPPB 2472 genome	bacteria'
CP014165.1 Bacillus pumilus strain ku-bf1 genome	bacteria'
JXTI01000001.1 Giardia intestinalis assemblage B strain BAH15c1 BAH15c1_001, whole genome shotgun sequence	protozoa'
LNQF01000003.1 Saccharomyces sp. 'boulardii' strain Unisankyo Contig3, whole genome shotgun sequence	fungi'
LNJS01000001.1 Burkholderia sp. MSMB1588 MSMB1588_2, whole genome shotgun sequence	bacteria'
CM003771.1 Burkholderia ubonensis strain MSMB1157 chromosome 1, complete sequence, whole genome shotgun sequence	bacteria'
CM003768.1 Burkholderia anthina strain AZ-4-2-10-S1-D7 chromosome 1, complete sequence, whole genome shotgun sequence	bacteria'
CP014242.1 Eremothecium sinecaudum strain ATCC 58844 chromosome II, complete sequence	fungi'
LOFC01000030.1 Magnaporthe oryzae strain EI9411 EI9411_contig_30_501659bp, whole genome shotgun sequence	fungi'
LOFD01000076.1 Magnaporthe oryzae strain EI9604 EI9604_contig_76_472660bp, whole genome shotgun sequence	fungi'
LOFB01000074.1 Magnaporthe grisea strain DS9461 DS9461_contig_74, whole genome shotgun sequence	fungi'
LOEM01000323.1 Magnaporthe grisea strain DS0505 DS0505_contig_323, whole genome shotgun sequence	fungi'
LOFE01000022.1 Magnaporthe oryzae strain SV9610 SV9610_contig_22, whole genome shotgun sequence	fungi'
LOFF01000216.1 Magnaporthe oryzae strain SV9623 SV9623_contig_216, whole genome shotgun sequence	fungi'
LAQH01005105.1 Wuchereria bancrofti isolate PNG22 PairedContig_2686, whole genome shotgun sequence	invertebrates'
LSAY01000001.1 Reticulascus tulasneorum strain NRRL18230 scaffold00001, whole genome shotgun sequence	fungi'
LSDU01000027.1 Lentinula edodes strain B17 Scaffold7, whole genome shotgun sequence	fungi'
CP014280.1 Enterobacter cloacae isolate MBRL1077 genome	bacteria'
JEMN01000491.1 Colletotrichum nymphaeae SA-01 Scaffold_144.1, whole genome shotgun sequence	fungi'
JFFI01001038.1 Colletotrichum salicis strain CBS 607.94 Scaffold_1932.1, whole genome shotgun sequence	fungi'
JFBX01000510.1 Colletotrichum simmondsii strain CBS122122 Scaffold_558.1, whole genome shotgun sequence	fungi'
CP014092.1 Escherichia coli strain 268-78-1, complete genome	bacteria'
KQ962167.1 Leucoagaricus sp. SymC.cos unplaced genomic scaffold scaffold343, whole genome shotgun sequence	fungi'
CP014347.1 Xanthomonas axonopodis pv. dieffenbachiae LMG 695 genome	bacteria'
KQ964245.1 Microdochium bolleyi strain J235TASD1 unplaced genomic scaffold Micbo1qcscaffold_1, whole genome shotgun sequence	fungi'
KQ964418.1 Conidiobolus coronatus NRRL 28638 unplaced genomic scaffold CONCOscaffold_1, whole genome shotgun sequence	fungi'
KQ965521.1 Talaromyces pinophilus strain 1-95 unplaced genomic scaffold scaffold_1, whole genome shotgun sequence	fungi'
CP014354.1 Coxiella burnetii strain 3345937 sequence	bacteria'
LSAJ01000001.1 Pythium oligandrum strain Po37 sc_001, whole genome shotgun sequence	protozoa'
KQ965731.1 Gonapodya prolifera JEL478 unplaced genomic scaffold M427scaffold_1, whole genome shotgun sequence	fungi'
CP013015.1 Delta proteobacterium HotSeep1 strain HS1 genome	bacteria'
CM003781.1 Coturnix japonica isolate 7356 chromosome 1, whole genome shotgun sequence	vertebrates_non_mammals'
LFZO01000001.1 Pseudocercospora musae strain CBS 116634 scaffold1, whole genome shotgun sequence	fungi'
LFZN01000004.1 Mycosphaerella eumusae strain CBS 114824 scaffold4, whole genome shotgun sequence	fungi'
CP014210.1 Pseudomonas aeruginosa strain KU genome	bacteria'
LSTP01000001.1 Ditylenchus destructor isolate Dd01 scaffold1, whole genome shotgun sequence	invertebrates'
LSVH01000001.1 Phaffia rhodozyma strain CBS 7918 Prho_CBS7918_scaffold_001, whole genome shotgun sequence	fungi'
LRSR01014122.1 Hypsibius dujardini strain Sciento H_dujardini_16, whole genome shotgun sequence	invertebrates'
CP014480.1 Kocuria turfanensis strain HO-9042 genome	bacteria'
CP013251.1 Endozoicomonas montiporae CL-33 genome	bacteria'
LJIH01000001.1 Vigna radiata var. radiata cultivar RIL59 scaffold_0, whole genome shotgun sequence	plants'
LSYV01000002.1 Gonium pectorale isolate NIES-2863 scaffold00001, whole genome shotgun sequence	plants'
CP010968.1 Mycobacterium tuberculosis strain PR10 genome	bacteria'
CP010996.1 Mycobacterium simiae strain MO323 genome	bacteria'
LQNK01000508.1 Phoebis sennae isolate eubule-3314 scaffold802_cov111, whole genome shotgun sequence	invertebrates'
CP011233.1 Salmonella enterica subsp. enterica serovar Typhimurium strain SL1344RX genome	bacteria'
LQNS01004683.1 Parhyale hawaiensis isolate isofemale 4 phaw_30.0004683, whole genome shotgun sequence	invertebrates'
CP014645.1 Lactobacillus rhamnosus strain ASCC 290 genome	bacteria'
JPDO01000001.1 Acidomyces richmondensis BFW scaffold_1, whole genome shotgun sequence	fungi'
CP014735.1 Saccharomyces cerevisiae strain BSPX042 chromosome IV sequence	fungi'
LJAO01000001.1 Peltaster fructicola strain LNHT1506 kmer90-min200.178, whole genome shotgun sequence	fungi'
LLXH01000019.1 Rhizophagus irregularis strain A1 scaffold19_size294061, whole genome shotgun sequence	fungi'
LLXK01000081.1 Rhizophagus irregularis strain B3 scaffold81_size301976, whole genome shotgun sequence	fungi'
LLXI01000001.1 Rhizophagus irregularis strain A4 scaffold1_size317905, whole genome shotgun sequence	fungi'
LLXL01000053.1 Rhizophagus irregularis strain C2 scaffold53_size267296, whole genome shotgun sequence	fungi'
LLXJ01000001.1 Rhizophagus irregularis strain A5 scaffold1_size353628, whole genome shotgun sequence	fungi'
CP014617.1 Mycobacterium africanum strain UT307 genome	bacteria'
LLKL01000320.1 Toxoplasma gondii RH scaffold1, whole genome shotgun sequence	protozoa'
JXSJ01000001.1 Miichthys miiuy scaffold1, whole genome shotgun sequence	vertebrates_non_mammals'
KQ976417.1 Atta colombica isolate Treedump-2 unplaced genomic scaffold scaffold12, whole genome shotgun sequence	invertebrates'
KQ982080.1 Trachymyrmex zeteki isolate Tzet28-1 chromosome Unknown scaffold106, whole genome shotgun sequence	invertebrates'
KQ977279.1 Cyphomyrmex costatus isolate MS0001 chromosome Unknown scaffold193, whole genome shotgun sequence	invertebrates'
KQ979074.1 Trachymyrmex cornetzi isolate Tcor2-1 chromosome Unknown scaffold16, whole genome shotgun sequence	invertebrates'
KQ981727.1 Trachymyrmex septentrionalis isolate Tsep2-gDNA-1 chromosome Unknown scaffold37, whole genome shotgun sequence	invertebrates'
CP013963.1 Brucella suis 019 chromosome 1 sequence	bacteria'
LDJU01000001.1 Miniopterus natalensis isolate MN2012-01 scaff1, whole genome shotgun sequence	vertebrates_mammals'
KQ991163.1 Dorcoceras hygrometricum cultivar XS01 unplaced genomic scaffold scaffold200, whole genome shotgun sequence	plants'
CP014844.1 Cupriavidus nantongensis strain X1 chromosome 1, complete sequence	bacteria'
CP014808.1 Borrelia hermsii strain DAH-2E7, complete genome	bacteria'
BCFV01000001.1 Trichosporon montevideense DNA, scaffold: scaffold_0, strain: JCM 9937, whole genome shotgun sequence	fungi'
BCFW01000001.1 Trichosporon domesticum DNA, scaffold: scaffold_0, strain: JCM 9580, whole genome shotgun sequence	fungi'
BCFX01000001.1 Trichoderma atroviride DNA, scaffold: scaffold_0, strain: JCM 9410, whole genome shotgun sequence	fungi'
BCFY01000001.1 Thielaviopsis paradoxa DNA, scaffold: scaffold_0, strain: JCM 6961, whole genome shotgun sequence	fungi'
BCGA01000001.1 Ambrosiozyma kashinagacola DNA, scaffold: scaffold_0, strain: JCM 15019, whole genome shotgun sequence	fungi'
BCGB01000001.1 Candida homilentoma DNA, scaffold: scaffold_0, strain: JCM 1507, whole genome shotgun sequence	fungi'
BCGC01000001.1 Candida sorboxylosa DNA, scaffold: scaffold_0, strain: JCM 1536, whole genome shotgun sequence	fungi'
BCGD01000001.1 Candida intermedia DNA, scaffold: scaffold_0, strain: JCM 1607, whole genome shotgun sequence	fungi'
BCGE01000001.1 Wickerhamia fluorescens DNA, scaffold: scaffold_0, strain: JCM 1821, whole genome shotgun sequence	fungi'
BCGF01000001.1 Pestalotiopsis sp. JCM 9685 DNA, scaffold: scaffold_0, strain: JCM 9685, whole genome shotgun sequence	fungi'
BCGI01000001.1 Cyberlindnera fabianii DNA, scaffold: scaffold_0, strain: JCM 3601, whole genome shotgun sequence	fungi'
BCGJ01000001.1 Saccharomycopsis malanga DNA, scaffold: scaffold_0, strain: JCM 7620, whole genome shotgun sequence	fungi'
BCGK01000001.1 Candida carpophila DNA, scaffold: scaffold_0, strain: JCM 9396, whole genome shotgun sequence	fungi'
BCGL01000001.1 Candida succiphila DNA, scaffold: scaffold_0, strain: JCM 9445, whole genome shotgun sequence	fungi'
BCGM01000001.1 Wickerhamiella domercqiae DNA, scaffold: scaffold_0, strain: JCM 9478, whole genome shotgun sequence	fungi'
BCGN01000001.1 Sporopachydermia quercuum DNA, scaffold: scaffold_0, strain: JCM 9486, whole genome shotgun sequence	fungi'
BCGO01000001.1 Starmerella bombicola DNA, scaffold: scaffold_0, strain: JCM 9596, whole genome shotgun sequence	fungi'
BCGP01000001.1 Candida boidinii DNA, scaffold: scaffold_0, strain: JCM 9604, whole genome shotgun sequence	fungi'
BCGQ01000001.1 Nakazawaea peltata DNA, scaffold: scaffold_0, strain: JCM 9829, whole genome shotgun sequence	fungi'
BCGR01000001.1 Phoma herbarum DNA, scaffold: scaffold_0, strain: JCM 15942, whole genome shotgun sequence	fungi'
BCGS01000001.1 Scheffersomyces lignosus DNA, scaffold: scaffold_0, strain: JCM 9837, whole genome shotgun sequence	fungi'
BCGT01000001.1 Aspergillus awamori var. piceus DNA, scaffold: scaffold_0, strain: JCM 22320, whole genome shotgun sequence	fungi'
BCGU01000001.1 Chalaropsis thielavioides DNA, scaffold: scaffold_0, strain: JCM 1933, whole genome shotgun sequence	fungi'
BCGY01000001.1 Aspergillus fumigatus var. fumigatus DNA, scaffold: scaffold_0, strain: JCM 10253, whole genome shotgun sequence	fungi'
BCGZ01000001.1 Schizophyllum commune DNA, scaffold: scaffold_0, strain: JCM 22674, whole genome shotgun sequence	fungi'
BCHA01000001.1 Ustilago maydis DNA, scaffold: scaffold_0, strain: JCM 2005, whole genome shotgun sequence	fungi'
BCHB01000001.1 Fusarium oxysporum DNA, scaffold: scaffold_0, strain: JCM 11502, whole genome shotgun sequence	fungi'
BCHD01000001.1 Exophiala spinifera DNA, scaffold: scaffold_0, strain: JCM 15939, whole genome shotgun sequence	fungi'
BCHF01000001.1 Stachybotrys echinata DNA, scaffold: scaffold_0, strain: JCM 22618, whole genome shotgun sequence	fungi'
BCHG01000002.1 Mucor circinelloides DNA, scaffold: scaffold_1, strain: JCM 22480, whole genome shotgun sequence	fungi'
BCHH01000001.1 Beverwykella pulmonaria DNA, scaffold: scaffold_0, strain: JCM 9230, whole genome shotgun sequence	fungi'
BCHJ01000001.1 Thielaviopsis paradoxa DNA, scaffold: scaffold_0, strain: JCM 6020, whole genome shotgun sequence	fungi'
BCHK01000001.1 Actinomucor elegans DNA, scaffold: scaffold_0, strain: JCM 22485, whole genome shotgun sequence	fungi'
BCHO01000001.1 Tilletiopsis pallescens DNA, scaffold: scaffold_0, strain: JCM 5230, whole genome shotgun sequence	fungi'
BCHP01000001.1 Basipetospora chlamydospora DNA, scaffold: scaffold_0, strain: JCM 23157, whole genome shotgun sequence	fungi'
BCHT01000001.1 Cryptococcus skinneri DNA, scaffold: scaffold_0, strain: JCM 9039, whole genome shotgun sequence	fungi'
BCHU01000001.1 Cryptococcus fagi DNA, scaffold: scaffold_0, strain: JCM 13614, whole genome shotgun sequence	fungi'
BCHV01000001.1 Cryptococcus albidus DNA, scaffold: scaffold_0, strain: JCM 2334, whole genome shotgun sequence	fungi'
BCHX01000001.1 Gliomastix tumulicola DNA, scaffold: scaffold_0, strain: JCM 17184, whole genome shotgun sequence	fungi'
BCHY01000001.1 Exophiala alcalophila DNA, scaffold: scaffold_0, strain: JCM 1751, whole genome shotgun sequence	fungi'
BCHZ01000001.1 Exophiala calicioides DNA, scaffold: scaffold_0, strain: JCM 6030, whole genome shotgun sequence	fungi'
BCIA01000001.1 Acremonium furcatum DNA, scaffold: scaffold_0, strain: JCM 9210, whole genome shotgun sequence	fungi'
BCIC01000001.1 Thermoascus crustaceus DNA, scaffold: scaffold_0, strain: JCM 12817, whole genome shotgun sequence	fungi'
BCID01000001.1 Penicillium roqueforti DNA, scaffold: scaffold_0, strain: JCM 22842, whole genome shotgun sequence	fungi'
BCIE01000001.1 Aspergillus chevalieri DNA, scaffold: scaffold_0, strain: JCM 23047, whole genome shotgun sequence	fungi'
BCIF01000001.1 Priceomyces haplophilus DNA, scaffold: scaffold_0, strain: JCM 1635, whole genome shotgun sequence	fungi'
BCIG01000001.1 Symbiotaphrina buchneri DNA, scaffold: scaffold_0, strain: JCM 9740, whole genome shotgun sequence	fungi'
BCIJ01000001.1 Holtermannia corniformis DNA, scaffold: scaffold_0, strain: JCM 1743, whole genome shotgun sequence	fungi'
BCIL01000001.1 Cystobasidium pallidum DNA, scaffold: scaffold_0, strain: JCM 3780, whole genome shotgun sequence	fungi'

BCIO01000001.1 Cystobasidiopsis lactophilus DNA, scaffold: scaffold_0, strain: JCM 7595, whole genome shotgun sequence	fungi'
BCIP01000001.1 Ambrosiozyma monospora DNA, scaffold: scaffold_0, strain: JCM 7599, whole genome shotgun sequence	fungi'
BCIS01000001.1 Cryptococcus phenolicus DNA, scaffold: scaffold_0, strain: JCM 11743, whole genome shotgun sequence	fungi'
BCIT01000001.1 Holtermanniella nyarrowii DNA, scaffold: scaffold_0, strain: JCM 11471, whole genome shotgun sequence	fungi'
BCIV01000001.1 Cryptococcus wieringae DNA, scaffold: scaffold_0, strain: JCM 11695, whole genome shotgun sequence	fungi'
BCIW01000001.1 Yarrowia deformans DNA, scaffold: scaffold_0, strain: JCM 1694, whole genome shotgun sequence	fungi'
BCIX01000001.1 Bulleromyces albus DNA, scaffold: scaffold_0, strain: JCM 2954, whole genome shotgun sequence	fungi'
BCIY01000001.1 Rhodotorula toruloides DNA, scaffold: scaffold_0, strain: JCM 10020, whole genome shotgun sequence	fungi'
BCIZ01000001.1 Rhodotorula toruloides DNA, scaffold: scaffold_0, strain: JCM 10021, whole genome shotgun sequence	fungi'
BCJA01000001.1 Rhodotorula toruloides DNA, scaffold: scaffold_0, strain: JCM 10049, whole genome shotgun sequence	fungi'
BCJC01000001.1 Erythrobasidium yunnanense DNA, scaffold: scaffold_0, strain: JCM 10687, whole genome shotgun sequence	fungi'
BCJD01000001.1 Yarrowia keelungensis DNA, scaffold: scaffold_0, strain: JCM 14894, whole genome shotgun sequence	fungi'
BCJE01000001.1 Rhodotorula toruloides DNA, scaffold: scaffold_0, strain: JCM 24501, whole genome shotgun sequence	fungi'
BCJF01000001.1 Vanrija humicola DNA, scaffold: scaffold_0, strain: JCM 1457, whole genome shotgun sequence	fungi'
BCJG01000001.1 Trichosporon porosum DNA, scaffold: scaffold_0, strain: JCM 1458, whole genome shotgun sequence	fungi'
BCJH01000001.1 Cryptococcus curvatus DNA, scaffold: scaffold_0, strain: JCM 1532, whole genome shotgun sequence	fungi'
BCJI01000001.1 Trichosporon brassicae DNA, scaffold: scaffold_0, strain: JCM 1599, whole genome shotgun sequence	fungi'
BCJN01000001.1 Trichosporon gamsii DNA, scaffold: scaffold_0, strain: JCM 9941, whole genome shotgun sequence	fungi'
BCJO01000001.1 Trichosporon gracile DNA, scaffold: scaffold_0, strain: JCM 10018, whole genome shotgun sequence	fungi'
BCJU01000001.1 Meira nashicola DNA, scaffold: scaffold_0, strain: JCM 18503, whole genome shotgun sequence	fungi'
BCJV01000001.1 Candida versatilis DNA, scaffold: scaffold_0, strain: JCM 5958, whole genome shotgun sequence	fungi'
BCJW01000001.1 Mrakia frigida DNA, scaffold: scaffold_0, strain: JCM 7857, whole genome shotgun sequence	fungi'
BCJX01000001.1 Trichosporon guehoae DNA, scaffold: scaffold_0, strain: JCM 10690, whole genome shotgun sequence	fungi'
BCJZ01000001.1 Xanthophyllomyces dendrorhous DNA, scaffold: scaffold_0, strain: JCM 9681, whole genome shotgun sequence	fungi'
BCKC01000001.1 Endocalyx cinctus DNA, scaffold: scaffold_0, strain: JCM 7946, whole genome shotgun sequence	fungi'
BCKD01000001.1 Pilasporangium apinafurcum DNA, scaffold: scaffold_0, strain: JCM 30513, whole genome shotgun sequence	protozoa'
BCKE01000001.1 Pilasporangium apinafurcum DNA, scaffold: scaffold_0, strain: JCM 30514, whole genome shotgun sequence	protozoa'
BCKF01000001.1 Yarrowia sp. JCM 30694 DNA, scaffold: scaffold_0, strain: JCM 30694, whole genome shotgun sequence	fungi'
BCKG01000001.1 Yarrowia sp. JCM 30696 DNA, scaffold: scaffold_0, strain: JCM 30696, whole genome shotgun sequence	fungi'
BCKH01000001.1 Albophoma yamanashiensis DNA, scaffold: scaffold_0, strain: JCM 11844, whole genome shotgun sequence	fungi'
BCKI01000001.1 Didymobotryum rigidum DNA, scaffold: scaffold_0, strain: JCM 8837, whole genome shotgun sequence	fungi'
BCKJ01000001.1 Trichosporon veenhuisii DNA, scaffold: scaffold_0, strain: JCM 10691, whole genome shotgun sequence	fungi'
BCKK01000001.1 Dioszegia crocea DNA, scaffold: scaffold_0, strain: JCM 2961, whole genome shotgun sequence	fungi'
BCKL01000001.1 Leucosporidium creatinivorum DNA, scaffold: scaffold_0, strain: JCM 10699, whole genome shotgun sequence	fungi'
BCKN01000001.1 Dioszegia aurantiaca DNA, scaffold: scaffold_0, strain: JCM 2956, whole genome shotgun sequence	fungi'
BCKO01000001.1 Millerozyma acaciae DNA, scaffold: scaffold_0, strain: JCM 10732, whole genome shotgun sequence	fungi'
BCKQ01000001.1 Ascoidea asiatica DNA, scaffold: scaffold_0, strain: JCM 7603, whole genome shotgun sequence	fungi'
BCKU01000001.1 Trichosporon cutaneum DNA, scaffold: scaffold_0, strain: JCM 1462, whole genome shotgun sequence	fungi'
BCKV01000001.1 Trichosporon laibachii DNA, scaffold: scaffold_0, strain: JCM 2947, whole genome shotgun sequence	fungi'
BCKW01000001.1 Ogataea methanolica DNA, scaffold: scaffold_0, strain: JCM 10240, whole genome shotgun sequence	fungi'
BCKX01000001.1 Malassezia dermatis DNA, scaffold: scaffold_0, strain: JCM 11348, whole genome shotgun sequence	fungi'
BCKY01000001.1 Malassezia japonica DNA, scaffold: scaffold_0, strain: JCM 11963, whole genome shotgun sequence	fungi'
BCKZ01000001.1 Alloascoidea hylecoeti DNA, scaffold: scaffold_0, strain: JCM 7604, whole genome shotgun sequence	fungi'
BCLA01000001.1 Malassezia nana DNA, scaffold: scaffold_0, strain: JCM 12085, whole genome shotgun sequence	fungi'
BCLC01000001.1 Cryptococcus sp. JCM 24511 DNA, scaffold: scaffold_0, strain: JCM 24511, whole genome shotgun sequence	fungi'
BCLD01000001.1 Cryptococcus terricola DNA, scaffold: scaffold_0, strain: JCM 24518, whole genome shotgun sequence	fungi'
CM003855.1 Plasmodium reichenowi strain SY57 chromosome 14, whole genome shotgun sequence	protozoa'
CM003869.1 Plasmodium gaboni strain SY75 chromosome 14, whole genome shotgun sequence	protozoa'
CP014868.1 Pseudomonas fluorescens strain KENGFT3 genome	bacteria'
BCLF01000001.1 Zoysia japonica DNA, contig: Zjn_sc00001.1, cultivar: Nagirizaki, whole genome shotgun sequence	plants'
BCLG01000001.1 Zoysia matrella DNA, contig: Zmw_sc00001.1, cultivar: Wakaba, whole genome shotgun sequence	plants'
BCLH01000001.1 Zoysia pacifica DNA, contig: Zpz_sc00001.1, cultivar: Zanpa, whole genome shotgun sequence	plants'
BDAN01000128.1 Flammulina velutipes DNA, contig: NODE_182, strain: TR19, whole genome shotgun sequence	fungi'
BCLX01000001.1 Yarrowia sp. JCM 30695 DNA, scaffold: scaffold_1, strain: JCM 30695, whole genome shotgun sequence	fungi'
KV388093.1 Lepidothrix coronata isolate B3197 unplaced genomic scaffold Scaffold0, whole genome shotgun sequence	vertebrates_non_mammals'
KV389426.1 Cebus capucinus imitator isolate Cc_AM_T3 unplaced genomic scaffold Scaffold0 whole genome shotgun sequence	vertebrates_mammals'
LUEZ01000001.1 Hypsizygus marmoreus strain 51987-8 scaffold_0, whole genome shotgun sequence	fungi'
CP010347.1 Bordetella pertussis strain I475 genome	bacteria'
JSDN01S027139.1 Dendrobium catenatum Dcat_scaffold_358, whole genome shotgun sequence	plants'
LTYT01001188.1 Anguilla rostrata isolate LakeOntario-01-2011 scaffold001188, whole genome shotgun sequence	vertebrates_non_mammals'
LQHG01037208.1 Oryza sativa Indica Group cultivar Tetep OsTET_37363, whole genome shotgun sequence	plants'
LQHE01008114.1 Oryza sativa Indica Group cultivar Co39 OsCo39_8114, whole genome shotgun sequence	plants'
LQHF01005255.1 Oryza sativa Indica Group cultivar Tadukan OsTAD_5256, whole genome shotgun sequence	plants'
CP013008.1 Arthrospira platensis YZ genome	bacteria'
KV392032.1 Trichosporon cutaneum strain CGMCC 2.1374 unplaced genomic scaffold scaffold1, whole genome shotgun sequence	fungi'
CP015048.1 Leptospira borgpetersenii serovar Hardjo strain BK-30 chromosome 1 sequence	bacteria'
CP015052.1 Leptospira borgpetersenii serovar Hardjo strain NVSL S 818 chromosome 1 sequence	bacteria'
CP015044.1 Leptospira borgpetersenii serovar Hardjo strain BK-6 chromosome 1 sequence	bacteria'
CP015046.1 Leptospira borgpetersenii serovar Hardjo strain BK-9 chromosome 1, complete sequence	bacteria'
CP015050.1 Leptospira borgpetersenii serovar Hardjo strain NVSL S 1343 chromosome 1 sequence	bacteria'
LFHQ01000537.1 Colletotrichum tofieldiae strain CBS 168.49 contig615, whole genome shotgun sequence	fungi'
LFHR01000304.1 Colletotrichum tofieldiae strain CBS 127615 contig369, whole genome shotgun sequence	fungi'
LFHP01001266.1 Colletotrichum tofieldiae strain CBS 495.85 contig1635, whole genome shotgun sequence	fungi'
LFHS01000445.1 Colletotrichum tofieldiae strain CBS 130851 contig599, whole genome shotgun sequence	fungi'
CM003885.1 Oryza sativa Indica Group cultivar Minghui 63 chromosome 1, whole genome shotgun sequence	plants'
CM003897.1 Oryza sativa Indica Group cultivar Zhenshan 97 chromosome 1, whole genome shotgun sequence	plants'
JYHR01000001.1 Drechmeria coniospora strain ATCC 96282 omap49267b, whole genome shotgun sequence	fungi'
KV407454.1 Xylona heveae TC161 unplaced genomic scaffold L228scaffold_1, whole genome shotgun sequence	fungi'
CM003910.1 Oryza sativa Indica Group cultivar Zhenshan 97 chromosome 1, whole genome shotgun sequence	plants'
CM003922.1 Oryza sativa Indica Group cultivar Minghui 63 chromosome 1, whole genome shotgun sequence	plants'
CP015225.1 Pseudomonas fluorescens strain FW300-N2E2 genome	bacteria'
CM003934.1 Mus musculus strain 129S1/SvImJ chromosome 1, whole genome shotgun sequence	vertebrates_mammals'
CM003954.1 Mus musculus strain A/J chromosome 1, whole genome shotgun sequence	vertebrates_mammals'
KV409358.1 Scleropages formosus breed green arowana unplaced genomic scaffold scaffold119, whole genome shotgun sequence	vertebrates_non_mammals'
KV408664.1 Scleropages formosus breed red arowana unplaced genomic scaffold scaffold42, whole genome shotgun sequence	vertebrates_non_mammals'
KV411217.1 Scleropages formosus breed golden arowana unplaced genomic scaffold scaffold72, whole genome shotgun sequence	vertebrates_non_mammals'
CM003974.1 Mus musculus strain AKR/J chromosome 1, whole genome shotgun sequence	vertebrates_mammals'
CM003994.1 Mus musculus castaneus strain CAST/EiJ chromosome 1, whole genome shotgun sequence	vertebrates_mammals'
CM004014.1 Mus musculus strain CBA/J chromosome 1, whole genome shotgun sequence	vertebrates_mammals'
CM004034.1 Mus musculus strain DBA/2J chromosome 1, whole genome shotgun sequence	vertebrates_mammals'
CM004074.1 Mus musculus strain FVB/NJ chromosome 1, whole genome shotgun sequence	vertebrates_mammals'
CM004134.1 Mus musculus strain NOD/ShiLtJ chromosome 1, whole genome shotgun sequence	vertebrates_mammals'
CM004154.1 Mus musculus strain NZO/HiLtJ chromosome 1, whole genome shotgun sequence	vertebrates_mammals'
CM004114.1 Mus musculus musculus strain PWK/PhJ chromosome 1, whole genome shotgun sequence	vertebrates_mammals'
CM004054.1 Mus musculus domesticus strain WSB/EiJ chromosome 1, whole genome shotgun sequence	vertebrates_mammals'
CM004094.1 Mus spretus strain SPRET/EiJ chromosome 1, whole genome shotgun sequence	vertebrates_mammals'
LRVB01000198.1 Saccharomyces sp. 'boulardii' strain ATCC MYA-797 contig_338, whole genome shotgun sequence	fungi'
LSRZ01000001.1 Haemoproteus tartakovskyi strain SISKIN1 HtScaffold0001, whole genome shotgun sequence	protozoa'
CM004174.1 Drechmeria coniospora strain ARSEF 6962 chromosome 01, whole genome shotgun sequence	fungi'
CM004278.1 Daucus carota subsp. sativus cultivar DH1 chromosome 1, whole genome shotgun sequence	plants'
LUGF01008404.1 Calycopis cecrops scaffold1367, whole genome shotgun sequence	invertebrates'
LFIV01000001.1 Colletotrichum tofieldiae strain 0861 Ct_v4_contig1, whole genome shotgun sequence	fungi'
LFIW01002504.1 Colletotrichum incanum strain MAFF 238704 contig2939, whole genome shotgun sequence	fungi'
KV411306.1 Branchiostoma belcheri isolate BF01 breed outbred unplaced genomic scaffold scaffold1, whole genome shotgun sequence	invertebrates'
KV416798.1 Branchiostoma belcheri isolate BF01 breed outbred unplaced genomic scaffold 220164597062, whole genome shotgun sequence	invertebrates'
LVCR01001269.1 Crotalus horridus isolate 016-059-111 Sequence_1269_262077, whole genome shotgun sequence	vertebrates_non_mammals'
CP015162.1 Treponema pallidum subsp. pallidum strain Amoy genome	bacteria'
CP014266.1 Acinetobacter baumannii strain Ab421_GEIH-2010 genome	bacteria'
KV417480.1 Fibulorhizoctonia sp. CBS 109695 unplaced genomic scaffold FIBSPscaffold_1, whole genome shotgun sequence	fungi'
KV417266.1 Calocera viscosa TUFC12733 unplaced genomic scaffold CALVIscaffold_1, whole genome shotgun sequence	fungi'
JYNV01000290.1 Ascochyta rabiei strain ArDII scaffold_336, whole genome shotgun sequence	fungi'
LWBM01000026.1 Aspergillus terreus strain 45A scaffold_26, whole genome shotgun sequence	fungi'
LWAD01000001.1 Diaporthe ampelina strain S3MP scaffold_1, whole genome shotgun sequence	fungi'
LVYW01000001.1 Sporothrix globosa strain CBS 120340 Scaffold_1, whole genome shotgun sequence	fungi'
LVYX01000001.1 Sporothrix globosa strain SS01 Scaffold_1, whole genome shotgun sequence	fungi'
KV419394.1 Sistotremastrum niveocremeum HHB9708 unplaced genomic scaffold SISNIscaffold_1, whole genome shotgun sequence	fungi'
LNAU01000470.1 Pseudogymnoascus sp. 04NY16 scaffold469, whole genome shotgun sequence	fungi'
LNAR01002106.1 Pseudogymnoascus sp. BL549 C15672, whole genome shotgun sequence	fungi'
LMYD01000275.1 Pseudogymnoascus sp. BL308 scaffold276, whole genome shotgun sequence	fungi'
LNAS01000063.1 Pseudogymnoascus pannorum var. pannorum strain ATCC 16222 scaffold59, whole genome shotgun sequence	fungi'
LTAH01131375.1 Oscheius sp. TEL-2014 isolate Inbred line 7 NODE_638708_length_42873_cov_3.493621, whole genome shotgun sequence	invertebrates'
CP015286.1 Paenibacillus glucanolyticus strain 5162 genome	bacteria'
KV429032.1 Daedalea quercina L-15889 unplaced genomic scaffold DAEQUscaffold_1, whole genome shotgun sequence	fungi'
KV428004.1 Sistotremastrum suecicum HHB10207 ss-3 unplaced genomic scaffold SISSUscaffold_1, whole genome shotgun sequence	fungi'
KV427605.1 Laetiporus sulphureus 93-53 unplaced genomic scaffold LAESUscaffold_1, whole genome shotgun sequence	fungi'
KV425882.1 Exidia glandulosa HHB12029 unplaced genomic scaffold EXIGLscaffold_1, whole genome shotgun sequence	fungi'
KV425551.1 Neolentinus lepideus HHB14362 ss-1 unplaced genomic scaffold NEOLEscaffold_1, whole genome shotgun sequence	fungi'
KV423914.1 Calocera cornea HHB12733 unplaced genomic scaffold CALCOscaffold_1, whole genome shotgun sequence	fungi'
KV424459.1 Peniophora sp. CONT unplaced genomic scaffold PENSPscaffold_1, whole genome shotgun sequence	fungi'
LRGB01000512.1 Daphnia magna strain Xinb3 scaffold00512, whole genome shotgun sequence	invertebrates'
CM004194.1 Mus musculus strain BALB/cJ chromosome 1, whole genome shotgun sequence	vertebrates_mammals'
CM004215.1 Mus musculus strain C57BL/6NJ chromosome 1, whole genome shotgun sequence	vertebrates_mammals'
CM004235.1 Mus musculus strain C3H/HeJ chromosome 1, whole genome shotgun sequence	vertebrates_mammals'
CM004256.1 Mus musculus strain LP/J chromosome 1, whole genome shotgun sequence	vertebrates_mammals'
LWBZ01000028.1 Fusarium sp. FSSC_6 Chr_1_0, whole genome shotgun sequence	fungi'
LKCN01000001.1 Ophiocordyceps polyrhachis-furcata BCC 54312 scaffold1, whole genome shotgun sequence	fungi'
LUCG01006598.1 Carthamus tinctorius cultivar AC SUNSET scaffold6606, whole genome shotgun sequence	plants'
LRBV01000112.1 Quercus lobata isolate SW786 scaffold95, whole genome shotgun sequence	plants'
KV775244.1 Dichanthelium oligosanthes cultivar Kellogg 1175 unplaced genomic scaffold Do_version_2_scaffold1, whole genome shotgun sequence	plants'
CM004297.1 Saccharomyces cerevisiae strain GLBRCY22-3 chromosome IV, whole genome shotgun sequence	fungi'
CM004191.1 Burkholderia territorii strain MSMB2203WGS chromosome 1, complete sequence, whole genome shotgun sequence	bacteria'
AZGY01000001.1 Aschersonia aleyrodis RCEF 2490 scaffold_1, whole genome shotgun sequence	fungi'
AZGZ01000001.1 Ascosphaera apis ARSEF 7405 Scaffold_1, whole genome shotgun sequence	fungi'
AZHB01000001.1 Isaria fumosorosea ARSEF 2679 scaffold_1, whole genome shotgun sequence	fungi'
AZHA01000001.1 Cordyceps brongniartii RCEF 3172 Scf_1, whole genome shotgun sequence	fungi'

AZHC01000001.1 Metarhizium rileyi RCEF 4871 scaffold_1, whole genome shotgun sequence	fungi'
AZHF01000001.1 Cordyceps confragosa RCEF 1005 scaffold_1, whole genome shotgun sequence	fungi'
AZHD01000001.1 Sporothrix insectorum RCEF 264 scaffold_1, whole genome shotgun sequence	fungi'
KV440971.1 Phycomyces blakesleeanus NRRL 1555(-) unplaced genomic scaffold PHYBLscaffold_1, whole genome shotgun sequence	fungi'
CP014501.1 Sugiyamaella lignohabitans strain CBS 10342 chromosome A, complete sequence	fungi'
KV441051.1 Clonostachys rosea strain YKD0085 unplaced genomic scaffold Scaffold1, whole genome shotgun sequence	fungi'
LXGT01000001.1 Endoconidiophora laricicola strain CBS100207 Contig0000001, whole genome shotgun sequence	fungi'
LXGU01000003.1 Ceratocystis adiposa strain CBS136.34 Contig0000001, whole genome shotgun sequence	fungi'
LLXD01000095.1 Lates calcarifer isolate ASB-BC8 unitig_2_quiver, whole genome shotgun sequence	vertebrates_non_mammals'
CP014278.2 Streptococcus pyogenes strain STAB13021 genome	bacteria'
KV441386.1 Pseudogymnoascus destructans isolate 20631-21 unplaced genomic scaffold scaffold_1, whole genome shotgun sequence	fungi'
LVLJ01002295.1 Marchantia polymorpha subsp. polymorpha scaffold4368, whole genome shotgun sequence	plants'
LWCA01000001.1 Intoshia linei isolate Intl2013 Contig_50380, whole genome shotgun sequence	invertebrates'
KV441548.1 Paraphaeosphaeria sporulosa strain AP3s5-JAC2a unplaced genomic scaffold CC84scaffold_1, whole genome shotgun sequence	fungi'
KV441469.1 Alternaria alternata strain SRC1lrK2f unplaced genomic scaffold CC77scaffold_1, whole genome shotgun sequence	fungi'
LSBG01000452.1 Mentha longifolia Contig454, whole genome shotgun sequence	plants'
LTDL01000014.1 Nematocida displodere strain JUm2807, whole genome shotgun sequence	fungi'
LTDK01000174.1 Nematocida sp. ERTm5 1, whole genome shotgun sequence	fungi'
LWRU01000187.1 Aspergillus fumigatus strain IF1SW-F4 jcf7180000007699, whole genome shotgun sequence	fungi'
LWRT01000208.1 Aspergillus fumigatus strain ISSFT-021 jcf7180000025878, whole genome shotgun sequence	fungi'
LSRY01000001.1 Monocercomonoides sp. PA203 scaffold00001, whole genome shotgun sequence	protozoa'
LWKT01000005.1 Caenorhabditis nigoni strain JU1421 chromosome X cniX, whole genome shotgun sequence	invertebrates'
LFJK01000005.1 Caenorhabditis remanei strain PX356 contig_1, whole genome shotgun sequence	invertebrates'
KV441705.1 Stagonospora sp. SRC1lsM3a unplaced genomic scaffold IQ06scaffold_1, whole genome shotgun sequence	fungi'
KV441636.1 Pyrenochaeta sp. DS3sAY3a unplaced genomic scaffold IQ07scaffold_1, whole genome shotgun sequence	fungi'
CM004348.2 Zea mays subsp. mays cultivar W22 chromosome 1, whole genome shotgun sequence	plants'
LWDD01000001.1 Tilletia caries strain DAOM 238032 scaffold_1, whole genome shotgun sequence	fungi'
LWDF01000001.1 Tilletia indica strain DAOM 236416 scaffold_1, whole genome shotgun sequence	fungi'
LWDE01000001.1 Tilletia controversa strain DAOM 236426 scaffold_1, whole genome shotgun sequence	fungi'
LWDG01000001.1 Tilletia walkeri strain DAOM 236422 scaffold_1, whole genome shotgun sequence	fungi'
LVCJ01000001.1 Fonsecaea nubica strain CBS 269.64 Scaff1, whole genome shotgun sequence	fungi'
LVCI01000064.1 Fonsecaea multimorphosa strain CBS 980.96 Scaff64, whole genome shotgun sequence	fungi'
LTAE01000001.1 Eichhornia paniculata isolate Mex scaffold1size249015, whole genome shotgun sequence	plants'
LVCG01000001.1 Oryza sativa cultivar KMR3 scaffold1, whole genome shotgun sequence	plants'
LVCH01000055.1 Oryza sativa cultivar IL50-13 scaffold55, whole genome shotgun sequence	plants'
LWBQ01000001.1 Ophiocordyceps sinensis strain ZJB12195 scaffold_1, whole genome shotgun sequence	fungi'
LVTN01000001.1 Musa itinerans isolate HN9 scaffold148, whole genome shotgun sequence	plants'
CM004386.1 Agrobacterium rhizogenes strain NCPPB2659 chromosome, complete sequence, whole genome shotgun sequence	bacteria'
LWHD01000001.1 Kryptolebias marmoratus isolate JLee-2015 Scaffold_0001, whole genome shotgun sequence	vertebrates_non_mammals'
KV441875.1 Gongronella sp. w5 unplaced genomic scaffold scaffold_1, whole genome shotgun sequence	fungi'
LXWW01000238.1 Blastocystis sp. ATCC 50177/Nand II strain NandII AV274scaffold_34, whole genome shotgun sequence	protozoa'
LVKQ01S001049.1 Giraffa camelopardalis tippelskirchi isolate MA1 scaffold268b2827433e5251928, whole genome shotgun sequence	vertebrates_mammals'
KV442011.1 Mortierella elongata AG-77 unplaced genomic scaffold K457scaffold_1, whole genome shotgun sequence	fungi'
LHPN01000001.1 Trichophyton violaceum strain CMCC(F)T3l scaffold1, whole genome shotgun sequence	fungi'
LHPM01000018.1 Trichophyton rubrum strain CMCC(F)T1i scaffold_2, whole genome shotgun sequence	fungi'
CM004359.1 Arabidopsis thaliana ecotype Landsberg erecta chromosome 1, whole genome shotgun sequence	plants'
LVYI01000001.1 Fonsecaea erecta strain CBS 125763 Scaff1, whole genome shotgun sequence	fungi'
LSNX01031463.1 Ceratina calcarata isolate Rehan_Ccalc_2016 scaffold39.2, whole genome shotgun sequence	invertebrates'
KV442483.1 Fusarium oxysporum f. sp. medicaginis isolate Fom-5190a unplaced genomic scaffold Scaffold_1, whole genome shotgun sequence	fungi'
CP015814.2 Leptospira borgpetersenii str. 4E chromosome 1 sequence	bacteria'
CM004372.1 Setaria italica cultivar TT8 chromosome IX, whole genome shotgun sequence	plants'
BDDP01003629.1 Tricholoma matsutake DNA, contig: contig_3629, strain: NBRC 30605, whole genome shotgun sequence	fungi'
LSBH01000001.1 Purpureocillium lilacinum isolate PLBJ-1 scaffold00001, whole genome shotgun sequence	fungi'
LUKN01003195.1 Cordyceps confragosa strain UM487 NODE_9271_length_134222_cov_23.660257, whole genome shotgun sequence	fungi'
LSBJ01000001.1 Pochonia chlamydosporia 170 scaffold00001, whole genome shotgun sequence	fungi'
LSBI01000001.1 Purpureocillium lilacinum isolate PLFJ-1 scaffold00001, whole genome shotgun sequence	fungi'
CP014639.1 Chlamydia sp. 2742-308 genome	bacteria'
LSRL01000005.1 Drosophila navojoa isolate navoj_Jal97 chromosome 2 MullerE, whole genome shotgun sequence	invertebrates'
LSRM01000005.1 Drosophila arizonae isolate ariz_Son04 chromosome 2 MullerE, whole genome shotgun sequence	invertebrates'
LVXX01000001.1 Hevea brasiliensis cultivar reyan7-33-97 scaffold0001, whole genome shotgun sequence	plants'
LVYE01000003.1 Ustilago trichophora strain RK089 scaffold3, whole genome shotgun sequence	fungi'
LTDI01000013.1 Fusarium sp. NRRL 54464 contig0013, whole genome shotgun sequence	fungi'
LUFC01000082.1 Fusarium sp. NRRL 31653 contig0082, whole genome shotgun sequence	fungi'
LXPA01005013.1 Phylloscopus trochiloides viridanus isolate TL2 scaffold5084, whole genome shotgun sequence	vertebrates_non_mammals'
LXOY01005518.1 Phylloscopus plumbeitarsus isolate BK2 scaffold5609, whole genome shotgun sequence	vertebrates_non_mammals'
LXOZ01032028.1 Phylloscopus trochiloides trochiloides isolate LN10 scaffold32496, whole genome shotgun sequence	vertebrates_non_mammals'
LPNZ01009878.1 Eukaryota sp. EH-2015 contig_14460, whole genome shotgun sequence	protozoa'
CP010558.1 Treponema pallidum subsp. pallidum strain Chicago Population genome	bacteria'
CP010559.1 Treponema pallidum subsp. pallidum strain CDC-A genome	bacteria'
CP010560.1 Treponema pallidum subsp. pallidum strain Nichols Houston clone E genome	bacteria'
CP010561.1 Treponema pallidum subsp. pallidum strain Nichols Houston clone J genome	bacteria'
CP010562.1 Treponema pallidum subsp. pallidum strain UW074B genome	bacteria'
CP010563.1 Treponema pallidum subsp. pallidum strain UW189B genome	bacteria'
CP010564.1 Treponema pallidum subsp. pallidum strain UW228B genome	bacteria'
CP010565.1 Treponema pallidum subsp. pallidum strain UW254B genome	bacteria'
CP010566.1 Treponema pallidum subsp. pallidum strain UW391B genome	bacteria'
LQHL01001873.1 Spathaspora hagerdaliae strain UFMG-CM-Y303 scf7180000106235, whole genome shotgun sequence	fungi'
LQMZ01002231.1 Spathaspora gorwiae strain UFMG-CM-Y312 scf7180000072932, whole genome shotgun sequence	fungi'
CP010029.1 Yersinia entomophaga strain MH96 genome	bacteria'
LQMS01002380.1 Spathaspora girioi strain UFMG-CM-Y302 NODE_1_length_330751_cov_14.2792_ID_1, whole genome shotgun sequence	fungi'
JNNX01000005.1 Talaromyces piceae strain 9-3 scaffold3, whole genome shotgun sequence	fungi'
KV448122.1 Rhizopogon vinicolor AM-OR11-026 unplaced genomic scaffold K503scaffold_1, whole genome shotgun sequence	fungi'
CM004387.1 Manihot esculenta cultivar AM560-2 chromosome LG1, whole genome shotgun sequence	plants'
CP015922.1 Polynucleobacter sp. QLW-P1FAT50C-4 genome	bacteria'
KV452433.1 Candida versatilis strain t-1 unplaced genomic scaffold scaffold00001, whole genome shotgun sequence	fungi'
KV452459.1 Angomonas deanei strain ATCC PRA-265 unplaced genomic scaffold Scaffold154, whole genome shotgun sequence	protozoa'
CM004405.1 Kluyveromyces marxianus isolate B0399 chromosome 1, whole genome shotgun sequence	fungi'
CM004414.1 Ictalurus punctatus breed USDA103 chromosome 1, whole genome shotgun sequence	vertebrates_non_mammals'
LGUA01000001.1 Emmonsia sp. CAC-2015a strain CBS 136260 supercont2.1, whole genome shotgun sequence	fungi'
LVCL01S0001936.1 Okapia johnstoni isolate WOAK scaffold1356b1e1905173, whole genome shotgun sequence	vertebrates_mammals'
LSRQ01001111.1 Ananas comosus cultivar MD2 ACMD2_Scaffold_1111, whole genome shotgun sequence	plants'
BCYN01000001.1 Fagopyrum esculentum DNA, contig: Fes_sc0000001.1, strain: BC1F6, whole genome shotgun sequence	plants'
BDDV01000032.1 Hypsizygus marmoreus DNA, contig: contig_32, strain: NN12, whole genome shotgun sequence	fungi'
KV454001.1 Pichia membranifaciens NRRL Y-2026 unplaced genomic scaffold PICMEscaffold_1, whole genome shotgun sequence	fungi'
KV454011.1 Pachysolen tannophilus NRRL Y-2460 unplaced genomic scaffold PACTA_scaffold_1, whole genome shotgun sequence	fungi'
KV454208.1 Wickerhamomyces anomalus NRRL Y-366-8 unplaced genomic scaffold WICANscaffold_1, whole genome shotgun sequence	fungi'
KV454254.1 Saitoella complicata NRRL Y-17804 unplaced genomic scaffold SAICOscaffold_1, whole genome shotgun sequence	fungi'
KV454406.1 Nadsonia fulvescens var. elongata DSM 6958 unplaced genomic scaffold NADFUscaffold_1, whole genome shotgun sequence	fungi'
KV454289.1 Lipomyces starkeyi NRRL Y-11557 unplaced genomic scaffold LIPSTscaffold_1, whole genome shotgun sequence	fungi'
KV454426.1 Babjeviella inositovora NRRL Y-12698 unplaced genomic scaffold BABINscaffold_1, whole genome shotgun sequence	fungi'
KV454475.1 Ascoidea rubescens DSM 1968 unplaced genomic scaffold ASCRUscaffold_1, whole genome shotgun sequence	fungi'
KV454538.1 Hyphopichia burtonii NRRL Y-1933 unplaced genomic scaffold HYPBUscaffold_1, whole genome shotgun sequence	fungi'
KV453925.1 Cyberlindnera jadinii NRRL Y-1542 unplaced genomic scaffold CYBJAscaffold_1, whole genome shotgun sequence	fungi'
KV453909.1 Candida tanzawaensis NRRL Y-17324 unplaced genomic scaffold CANTAscaffold_1, whole genome shotgun sequence	fungi'
KV453847.1 Candida arabinofermentans NRRL YB-2248 unplaced genomic scaffold CANARscaffold_1, whole genome shotgun sequence	fungi'
KV453841.1 Tortispora caseinolytica NRRL Y-17796 unplaced genomic scaffold CANCAscaffold_1, whole genome shotgun sequence	fungi'
LUVW01000001.1 Capniomyces stellatus strain MIS-10-108 Cste_Scaff_1, whole genome shotgun sequence	fungi'
BCNH01000134.1 Metrosideros polymorpha var. glaberrima DNA, scaffold: scaffold134_cov180, whole genome shotgun sequence	plants'
BDCZ01000001.1 Chlamydomonas applanata DNA, contig: CAP0001, whole genome shotgun sequence	plants'
BDDA01000001.1 Chlamydomonas asymmetrica DNA, contig: CAS0001, whole genome shotgun sequence	plants'
BDDB01000001.1 Chlamydomonas debaryana DNA, contig: CDE00001, whole genome shotgun sequence	plants'
BDDC01000001.1 Chlamydomonas sphaeroides DNA, contig: CSP0001, whole genome shotgun sequence	plants'
BCFZ01000001.1 Raffaelea quercivora DNA, scaffold: scaffold_0, strain: JCM 11526, whole genome shotgun sequence	fungi'
LYNE01000001.1 Rosa x damascena scaffold1, whole genome shotgun sequence	plants'
KV454974.1 Pseudogymnoascus sp. 23342-1-I1 unplaced genomic scaffold scaffold_1, whole genome shotgun sequence	fungi'
KV454565.1 Pseudogymnoascus sp. WSF 3629 unplaced genomic scaffold scaffold_1, whole genome shotgun sequence	fungi'
KV455952.1 Pseudogymnoascus sp. 24MN13 unplaced genomic scaffold scaffold_1, whole genome shotgun sequence	fungi'
KV458779.1 Pseudogymnoascus sp. 05NY08 unplaced genomic scaffold scaffold_1, whole genome shotgun sequence	fungi'
KV459350.1 Pseudogymnoascus sp. 03VT05 unplaced genomic scaffold scaffold_1, whole genome shotgun sequence	fungi'
KV460206.1 Pseudogymnoascus verrucosus strain UAMH 10579 unplaced genomic scaffold scaffold_1, whole genome shotgun sequence	fungi'
LVCK01001032.1 Colletotrichum acutatum strain C71 scaffold1927, whole genome shotgun sequence	fungi'
LZRM01001561.1 Colletotrichum godetiae strain C184 scaffold1562, whole genome shotgun sequence	fungi'
JTCL01004111.1 Morone saxatilis breed North Carolina State University Domesticated Broodstock SB-2011 Msax_4111, whole genome shotgun sequence	vertebrates_non_mammals'
LZCU01147958.1 Asymmetron lucayanum isolate AD20110701 scaffold322456_cov65, whole genome shotgun sequence	invertebrates'
LHSH01000011.1 Kryptolebias marmoratus strain RHL Kmar00011, whole genome shotgun sequence	vertebrates_non_mammals'
CM004466.1 Xenopus laevis strain J chromosome 1L, whole genome shotgun sequence	vertebrates_non_mammals'
LXPE01000001.1 Hanseniaspora valbyensis NRRL Y-1626 HANVAscaffold_1, whole genome shotgun sequence	fungi'
LXTC01000001.1 Metschnikowia bicuspidata var. bicuspidata NRRL YB-4993 METBIscaffold_1, whole genome shotgun sequence	fungi'
CP011806.1 Acidobacteria bacterium Mor1 sequence	bacteria'
CP007916.1 Saccharomyces cerevisiae strain HPRMAwf_D10 chromosome IV sequence	fungi'
CP007814.1 Saccharomyces cerevisiae strain T.52_3C chromosome IV sequence	fungi'
CP007831.1 Saccharomyces cerevisiae strain T.52_3A chromosome IV sequence	fungi'
CP007848.1 Saccharomyces cerevisiae strain T.52_2H chromosome IV sequence	fungi'
CP007865.1 Saccharomyces cerevisiae strain Sol7-2 chromosome IV sequence	fungi'
CP007882.1 Saccharomyces cerevisiae strain HB_C_TUKITUKI2_4 chromosome IV sequence	fungi'
CP007899.1 Saccharomyces cerevisiae strain NSERVsf_F8 chromosome IV sequence	fungi'
CP007933.1 Saccharomyces cerevisiae strain HCNTHsf_F8 chromosome IV sequence	fungi'
CP007950.1 Saccharomyces cerevisiae strain T78 chromosome IV sequence	fungi'
CP007967.1 Saccharomyces cerevisiae strain HCNTHsf_C5 chromosome IV sequence	fungi'
CP008035.1 Saccharomyces cerevisiae strain TNPLST-4-S-2 chromosome IV sequence	fungi'
CP008052.1 Saccharomyces cerevisiae strain HB_S_GIMBLETTROAD_9 chromosome IV sequence	fungi'
CP008069.1 Saccharomyces cerevisiae strain T8 chromosome IV sequence	fungi'
CP008120.1 Saccharomyces cerevisiae strain MTKSKsf_E2 chromosome IV sequence	fungi'
CP007984.1 Saccharomyces cerevisiae strain CRIRIwf_A11 chromosome IV sequence	fungi'
CP008001.1 Saccharomyces cerevisiae strain CDRDR_sf_H chromosome IV sequence	fungi'

CP008018.1 Saccharomyces cerevisiae strain Soil7-1 chromosome IV sequence	fungi'
CP008154.1 Saccharomyces cerevisiae strain HPRMTsf_H7 chromosome IV sequence	fungi'
CP008324.1 Saccharomyces cerevisiae strain WI_S_OAKURA_4 chromosome IV sequence	fungi'
CP008290.1 Saccharomyces cerevisiae strain HB_S_BILANCHER_12 chromosome IV sequence	fungi'
CP008222.1 Saccharomyces cerevisiae strain HB_S_GIMBLETTROAD_22 chromosome IV sequence	fungi'
CP008409.1 Saccharomyces cerevisiae strain HB_C_OMARUNUI_7 chromosome IV sequence	fungi'
CP008392.1 Saccharomyces cerevisiae strain HB_C_TUKITUKI1_16 chromosome IV sequence	fungi'
CP008341.1 Saccharomyces cerevisiae strain WI_S_JASA_5 chromosome IV sequence	fungi'
CP008494.1 Saccharomyces cerevisiae strain T52 chromosome IV sequence	fungi'
CP008460.1 Saccharomyces cerevisiae strain HB_C_KOROKIPO_3 chromosome IV sequence	fungi'
CP008443.1 Saccharomyces cerevisiae strain HB_C_OMARUNUI_14 chromosome IV sequence	fungi'
CP008426.1 Saccharomyces cerevisiae strain HB_C_OMARUNUI_6 chromosome IV sequence	fungi'
CP008562.1 Saccharomyces cerevisiae strain WA_C_WAITAKEREROAD_7 chromosome IV sequence	fungi'
CP008545.1 Saccharomyces cerevisiae strain WI_C_MB95MBMZ_4 chromosome IV sequence	fungi'
CP008528.1 Saccharomyces cerevisiae strain WI_C_MBSP_15 chromosome IV sequence	fungi'
CP008511.1 Saccharomyces cerevisiae strain WI_C_MBSP_4 chromosome IV sequence	fungi'
CP008647.1 Saccharomyces cerevisiae strain T.52_5E chromosome IV sequence	fungi'
CP008613.1 Saccharomyces cerevisiae strain WA_C_KINGSMILL_10 chromosome IV sequence	fungi'
CP008596.1 Saccharomyces cerevisiae strain WA_C_MATES_10 chromosome IV sequence	fungi'
CP008205.1 Saccharomyces cerevisiae strain WSERCsf_G4 chromosome IV sequence	fungi'
CP008188.1 Saccharomyces cerevisiae strain HB_S_GIMBLETTROAD_5 chromosome IV sequence	fungi'
CP008171.1 Saccharomyces cerevisiae strain HCNKIsf_G7 chromosome IV sequence	fungi'
CP008086.1 Saccharomyces cerevisiae strain WSETAwf_B1 chromosome IV sequence	fungi'
CP008103.1 Saccharomyces cerevisiae strain NSEBRsf_A9 chromosome IV sequence	fungi'
CP008137.1 Saccharomyces cerevisiae strain MARARsf_A10 chromosome IV sequence	fungi'
CP008681.1 Saccharomyces cerevisiae strain T16 chromosome IV sequence	fungi'
CP008630.1 Saccharomyces cerevisiae strain WA_C_CODDINGTON_2 chromosome IV sequence	fungi'
CP008664.1 Saccharomyces cerevisiae strain T.52_5A chromosome IV sequence	fungi'
CP008579.1 Saccharomyces cerevisiae strain WA_C_MATES_13 chromosome IV sequence	fungi'
CP008477.1 Saccharomyces cerevisiae strain HB_C_KOROKIPO_12 chromosome IV sequence	fungi'
CP008358.1 Saccharomyces cerevisiae strain WI_S_JASA_13 chromosome IV sequence	fungi'
CP008375.1 Saccharomyces cerevisiae strain HB_C_TUKITUKI2_10 chromosome IV sequence	fungi'
CP008256.1 Saccharomyces cerevisiae strain HB_S_GIMBLETTROAD_14 chromosome IV sequence	fungi'
CP008307.1 Saccharomyces cerevisiae strain T63 chromosome IV sequence	fungi'
CP008273.1 Saccharomyces cerevisiae strain HB_S_BILANCHER_6 chromosome IV sequence	fungi'
CP008239.1 Saccharomyces cerevisiae strain HB_S_GIMBLETTROAD_16 chromosome IV sequence	fungi'
CP016034.1 Escherichia coli isolate Co6114 genome	bacteria'
JRVM01000001.1 Elaeis guineensis cultivar R10/1 Dura contig_0, whole genome shotgun sequence	plants'
CM004455.1 Colletotrichum higginsianum IMI 349063 chromosome 1, whole genome shotgun sequence	fungi'
CP013643.1 Rhizobium sp. N941, complete genome	bacteria'
CP013636.1 Rhizobium sp. N541, complete genome	bacteria'
LYXU01000001.1 Fusarium poae strain 2516 chromosome FPOA_1 Chr1, whole genome shotgun sequence	fungi'
LNFW01000933.1 Bipolaris oryzae isolate TG12bL2 contig_1320, whole genome shotgun sequence	fungi'
LZPO01057484.1 Neotoma lepida isolate 417 scaffold_0, whole genome shotgun sequence	vertebrates_mammals'
LXOO01000001.1 Magnaporthe oryzae strain BdJes16-1 scaffold00001, whole genome shotgun sequence	fungi'
LXOP01000001.1 Magnaporthe oryzae strain BdMeh16-1 scaffold00001, whole genome shotgun sequence	fungi'
LXON01000001.1 Magnaporthe oryzae strain BdBar16-1 scaffold00001, whole genome shotgun sequence	fungi'
LXOQ01000001.1 Magnaporthe oryzae strain B71 scaffold00001, whole genome shotgun sequence	fungi'
LZPB01000001.1 Ceratocystiopsis minuta strain CBS 138717 CMW4352_scaffold_1, whole genome shotgun sequence	fungi'
CP016227.1 Streptococcus pneumoniae strain D219 genome	bacteria'
LVWM01000001.1 Aureobasidium pullulans isolate Santander contig_1, whole genome shotgun sequence	fungi'
CP016285.1 Bacillus sp. B25(2016b) genome	bacteria'
LWUH01001073.1 Armillaria fuscipes strain CMW2740 Contig0.1073, whole genome shotgun sequence	fungi'
CP016250.1 Plasmodium coatneyi strain Hackeri chromosome 12, complete sequence	protozoa'
LYYD01000315.1 Toxocara canis isolate Korea scaffold315, whole genome shotgun sequence	invertebrates'
CP008725.1 Candidatus Thioglobus singularis isolate GG2 genome	bacteria'
CP015470.1 Agaricus bisporus var. bisporus strain H39 chromosome 1, complete sequence	fungi'
KV575286.1 Pygocentrus nattereri isolate Pna-1 unplaced genomic scaffold Scaffold0, whole genome shotgun sequence	vertebrates_non_mammals'
CM004498.1 Brassica nigra cultivar inbred line YZ12151 chromosome B8, whole genome shotgun sequence	plants'
CP016201.1 Selenomonas sp. oral taxon 126 strain W7667 genome	bacteria'
CP016204.1 Prevotella scopos JCM 17725 strain W2052 chromosome 1 genome	bacteria'
CP016207.1 Streptococcus sp. oral taxon 064 strain W10853 sequence	bacteria'
LPWI01000001.1 Chenopodium quinoa cultivar QQ74 C_Quinoa_Scaffold_1000, whole genome shotgun sequence	plants'
LUGH01000001.1 Choanephora cucurbitarum strain KUS-F28377 scaffold_0, whole genome shotgun sequence	fungi'
CP018146.1 Leptospira interrogans serovar Copenhageni/Icterohaemorrhagiae strain Piscina chromosome 1 sequence	bacteria'
KV581049.1 Beauveria bassiana strain ARSEF 1520 unplaced genomic scaffold BB1520_0001, whole genome shotgun sequence	fungi'
KV580561.1 Beauveria bassiana strain ARSEF 2597 unplaced genomic scaffold BB2597_0001, whole genome shotgun sequence	fungi'
KV580651.1 Beauveria bassiana strain ARSEF 5078 unplaced genomic scaffold BB5078_0001, whole genome shotgun sequence	fungi'
KV580213.1 Beauveria bassiana strain ARSEF 4305 unplaced genomic scaffold BB4305_0001, whole genome shotgun sequence	fungi'
LOEQ01000025.1 Ellobius lutescens isolate ElloLutescens_01 scaffold25_size2242254, whole genome shotgun sequence	vertebrates_mammals'
LOJH01000002.1 Ellobius talpinus isolate ElloTalpinus_01 scaffold2_size160076, whole genome shotgun sequence	vertebrates_mammals'
LOJG01000004.1 Ellobius lutescens isolate ElloLutescens_female_01 scaffold4_size160323, whole genome shotgun sequence	vertebrates_mammals'
JSZB01000232.1 Manis javanica isolate MP_PG03-UM SCAFFOLD259, whole genome shotgun sequence	vertebrates_mammals'
MATR01000147.1 Chenopodium pallidicaule isolate PI_478407 scaffold_0, whole genome shotgun sequence	plants'
MAMN01000025.1 Arachis duranensis cultivar PI475845 scaffold1, whole genome shotgun sequence	plants'
MATQ01000035.1 Chenopodium suecicum isolate BYU_1480 scaffold_0, whole genome shotgun sequence	plants'
CP011109.1 Bacillus pumilus strain C4 genome	bacteria'
KV700387.1 Rhagoletis zephyria isolate East Lansing unplaced genomic scaffold Rzeph_scaffold_00001, whole genome shotgun sequence	invertebrates'
CM007197.1 Brassica juncea var. tumida cultivar T84-66 (inbred line) chromosome B3, whole genome shotgun sequence	plants'
KV722330.1 Obba rivulosa strain 3A-2 unplaced genomic scaffold OBBRIscaffold_1, whole genome shotgun sequence	fungi'
MATU01003950.1 Vigna unguiculata subsp. unguiculata cultivar IT97K-499-35 scaffold4028, whole genome shotgun sequence	plants'
MAPY01000420.1 Tilletia indica strain PSWKBGD_1_3 scaffold4_size112495, whole genome shotgun sequence	fungi'
MAPW01000001.1 Tilletia indica strain PSWKBGH_1 scaffold1_size924144, whole genome shotgun sequence	fungi'
JNHY01003865.1 Amanita polypyramis BW_CC seq2971016, whole genome shotgun sequence	fungi'
JNHX01010997.1 Amanita muscaria var. guessowii Koide BX008 seq312457, whole genome shotgun sequence	fungi'
JNHW01005841.1 Amanita inopinata Kibby_2008 seq155632, whole genome shotgun sequence	fungi'
JNHV01016116.1 Amanita brunnescens Koide BX004 seq596096, whole genome shotgun sequence	fungi'
JNHZ01003571.1 Volvariella volvacea WC 439 seq80656, whole genome shotgun sequence	fungi'
KV744805.1 Lepidopterella palustris CBS 459.81 unplaced genomic scaffold K432scaffold_1, whole genome shotgun sequence	fungi'
KV748176.1 Cenococcum geophilum 1.58 unplaced genomic scaffold K441scaffold_1, whole genome shotgun sequence	fungi'
KV749029.1 Glonium stellatum strain CBS 207.34 unplaced genomic scaffold AOQ84NODE_701, whole genome shotgun sequence	fungi'
MBSK01000001.1 Sesamum indicum isolate Yuzhi11 scaffold00001, whole genome shotgun sequence	plants'
MASO02000001.1 Colletotrichum lindemuthianum strain 83.501 isolate-83-0000001, whole genome shotgun sequence	fungi'
MASP02000025.1 Colletotrichum lindemuthianum strain 89 A2 2-3 isolate-89-0000025, whole genome shotgun sequence	fungi'
LWMJ02000001.1 Taenia asiatica isolate TASYD01 Scaffold00001, whole genome shotgun sequence	invertebrates'
LWMK02000001.1 Taenia saginata isolate TSAYD01 Scaffold00001, whole genome shotgun sequence	invertebrates'
CM004509.1 Gilliamella apicola strain wkB7 chromosome, complete sequence, whole genome shotgun sequence	bacteria'
MBPM01000001.1 Plasmopara viticola isolate INRA-PV221 Plvit221r1_S0004, whole genome shotgun sequence	protozoa'
JYJY01000235.1 Calonectria pseudonaviculata strain CBS 139394 CpsSB_contig_260, whole genome shotgun sequence	fungi'
MBGJ01000102.1 Hibiscus syriacus cultivar Gangneung scaffold4_size1538241, whole genome shotgun sequence	plants'
LSHC01000350.1 Alternaria alternata strain B2a contig_23, whole genome shotgun sequence	fungi'
CP016787.1 Bacillus subtilis subsp. subtilis strain IIG-Bs27-47-24 genome	bacteria'
CP016788.1 Bacillus subtilis subsp. subtilis strain PG10 genome	bacteria'
CP016789.1 Bacillus subtilis subsp. subtilis strain PS38 genome	bacteria'
MCGX01003819.1 Rhinopithecus bieti isolate Rb0 scaffold_6380, whole genome shotgun sequence	vertebrates_mammals'
KV751334.1 Mola mola isolate MOLMO unplaced genomic scaffold scaffold20, whole genome shotgun sequence	vertebrates_non_mammals'
LUXX01074245.1 Sus scrofa breed LargeWhite scaffold31317.1, whole genome shotgun sequence	vertebrates_mammals'
LUXR01101794.1 Sus scrofa breed Rongchang scaffold18251.1, whole genome shotgun sequence	vertebrates_mammals'
LUXS01076039.1 Sus scrofa breed Hampshire scaffold27059.6, whole genome shotgun sequence	vertebrates_mammals'
LUXQ01116737.1 Sus scrofa breed Meishan scaffold46825, whole genome shotgun sequence	vertebrates_mammals'
LUXT01050959.1 Sus scrofa breed Landrace scaffold26153, whole genome shotgun sequence	vertebrates_mammals'
LUXV01009748.1 Sus scrofa breed Bamei scaffold29623, whole genome shotgun sequence	vertebrates_mammals'
LUXU01038316.1 Sus scrofa breed Pietrain scaffold11495, whole genome shotgun sequence	vertebrates_mammals'
LUXY01075128.1 Sus scrofa breed Jinhua scaffold13801.4, whole genome shotgun sequence	vertebrates_mammals'
LUXW01088374.1 Sus scrofa breed Berkshire scaffold21591, whole genome shotgun sequence	vertebrates_mammals'
CP007484.1 Salmonella enterica subsp. enterica strain SA972816, complete genome	bacteria'
KV756872.1 Lentinula edodes strain W1-26 unplaced genomic scaffold LE01Scaffold001, whole genome shotgun sequence	fungi'
LGRB01000008.1 Cladophialophora carrionii strain KSF Contig1623, whole genome shotgun sequence	fungi'
CM004529.1 Passer domesticus isolate 8887266 chromosome 2, whole genome shotgun sequence	vertebrates_non_mammals'
MAVT01001068.1 Diaporthe helianthi strain 7/96 Scaffold_1096.1, whole genome shotgun sequence	fungi'
MABJ01000028.1 Fusarium oxysporum f. cucumerinum strain Foc013 Foq013_contig_28, whole genome shotgun sequence	fungi'
MAKY01000358.1 Fusarium oxysporum f. sp. niveum strain Fon005 Fon005blob3c_contig_359, whole genome shotgun sequence	fungi'
MAKZ01000078.1 Fusarium oxysporum f. cucumerinum strain Foc001 Foq001_contig_78, whole genome shotgun sequence	fungi'
MABM01000081.1 Fusarium oxysporum f. cucumerinum strain Foc018 Foq018_contig_81, whole genome shotgun sequence	fungi'
MABL01000314.1 Fusarium oxysporum f. cucumerinum strain Foc021 Foq021_contig_314, whole genome shotgun sequence	fungi'
MABK01000111.1 Fusarium oxysporum f. cucumerinum strain Foc015 Foq015_contig_111, whole genome shotgun sequence	fungi'
MABN01000027.1 Fusarium oxysporum f. cucumerinum strain Foc030 Foq030blob2c_contig_27, whole genome shotgun sequence	fungi'
MABP01000025.1 Fusarium oxysporum f. cucumerinum strain Foc037 Foq037_contig_25, whole genome shotgun sequence	fungi'
MABS01000030.1 Fusarium oxysporum f. sp. radicis-cucumerinum strain Forc031 Forc031_contig_30, whole genome shotgun sequence	fungi'
MABO01000129.1 Fusarium oxysporum f. cucumerinum strain Foc035 Foq035_contig_129, whole genome shotgun sequence	fungi'
MABQ01000005.1 Fusarium oxysporum f. sp. radicis-cucumerinum strain Forc016 Forc016_contig_5, whole genome shotgun sequence	fungi'
MAMH01000023.1 Fusarium oxysporum f. sp. niveum strain Fon019 Fon019blob2c_contig_23, whole genome shotgun sequence	fungi'
MABR01000023.1 Fusarium oxysporum f. sp. radicis-cucumerinum strain Forc024 Forc024_contig_23, whole genome shotgun sequence	fungi'
MALA01000044.1 Fusarium oxysporum f. sp. niveum strain Fon002 Fon002_contig_44, whole genome shotgun sequence	fungi'
MALC01000096.1 Fusarium oxysporum f. sp. niveum strain Fon013 Fon013blob2c_contig_96, whole genome shotgun sequence	fungi'
MALB01000150.1 Fusarium oxysporum f. sp. niveum strain Fon010 Fon010_contig_150, whole genome shotgun sequence	fungi'
MALD01000034.1 Fusarium oxysporum f. sp. niveum strain Fon015 Fon015blob2c_contig_34, whole genome shotgun sequence	fungi'
MALE01000087.1 Fusarium oxysporum f. sp. niveum strain Fon020 Fon020_contig_87, whole genome shotgun sequence	fungi'
MALF01000046.1 Fusarium oxysporum f. sp. niveum strain Fon037 Fon037_contig_46, whole genome shotgun sequence	fungi'
MALG01000068.1 Fusarium oxysporum f. sp. niveum strain Fon021 Fon021blob3c_contig_68, whole genome shotgun sequence	fungi'
MALH01000023.1 Fusarium oxysporum f. sp. lycopersici strain Fol004 Fol004_contig_23, whole genome shotgun sequence	fungi'
MALI01000064.1 Fusarium oxysporum f. sp. lycopersici strain Fol007 Fol007illumina_contig_64, whole genome shotgun sequence	fungi'
MALK01000009.1 Fusarium oxysporum f. sp. lycopersici strain Fol026 Fol026_contig_9, whole genome shotgun sequence	fungi'
MALJ01000049.1 Fusarium oxysporum f. sp. lycopersici strain Fol014 Fol014_contig_49, whole genome shotgun sequence	fungi'
MALL01000022.1 Fusarium oxysporum f. sp. lycopersici strain Fol018 Fol018_contig_22, whole genome shotgun sequence	fungi'

MALM01000147.1 Fusarium oxysporum f. sp. lycopersici strain Fol016 Fol016_contig_147, whole genome shotgun sequence	fungi'
MALO01000133.1 Fusarium oxysporum f. sp. lycopersici strain Fol038 Fol038_contig_133, whole genome shotgun sequence	fungi'
MALN01000001.1 Fusarium oxysporum f. sp. lycopersici strain Fol029 Fol029_contig_1, whole genome shotgun sequence	fungi'
MALP01000036.1 Fusarium oxysporum f. sp. lycopersici strain Fol069 Fol069_contig_36, whole genome shotgun sequence	fungi'
MALQ01000021.1 Fusarium oxysporum f. sp. lycopersici strain Fol072 Fol072_contig_21, whole genome shotgun sequence	fungi'
MALR01000043.1 Fusarium oxysporum f. sp. lycopersici strain Fol073 Fol073_contig_43, whole genome shotgun sequence	fungi'
MALS01000071.1 Fusarium oxysporum f. sp. lycopersici strain Fol074 Fol074_contig_71, whole genome shotgun sequence	fungi'
MALU01000008.1 Fusarium oxysporum strain FoMN14 FoMN14_contig_8, whole genome shotgun sequence	fungi'
MALT01000041.1 Fusarium oxysporum f. sp. lycopersici strain Fol075 Fol075_contig_41, whole genome shotgun sequence	fungi'
MALW01000040.1 Fusarium oxysporum f. sp. lycopersici 4287 Fol4287illumina_contig_40, whole genome shotgun sequence	fungi'
MALY01000048.1 Fusarium oxysporum f. sp. melonis strain Fom005 Fom005pilon_contig_48, whole genome shotgun sequence	fungi'
MALX01000016.1 Fusarium oxysporum f. sp. melonis strain Fom004 Fom004pilon_contig_16, whole genome shotgun sequence	fungi'
MALZ01000010.1 Fusarium oxysporum f. sp. melonis strain Fom006 Fom006pilon_contig_10, whole genome shotgun sequence	fungi'
MAMA01000006.1 Fusarium oxysporum f. sp. melonis strain Fom009 Fom009pilon_contig_6, whole genome shotgun sequence	fungi'
MAMC01000110.1 Fusarium oxysporum f. sp. melonis strain Fom011 Fom011pilon_contig_110, whole genome shotgun sequence	fungi'
MAMB01000046.1 Fusarium oxysporum f. sp. melonis strain Fom010 Fom010pilon_contig_46, whole genome shotgun sequence	fungi'
MAME01000138.1 Fusarium oxysporum f. sp. melonis strain Fom013 Fom013pilon_contig_138, whole genome shotgun sequence	fungi'
MAMD01000116.1 Fusarium oxysporum f. sp. melonis strain Fom012 Fom012pilon_contig_116, whole genome shotgun sequence	fungi'
MAMG01000023.1 Fusarium oxysporum f. sp. lycopersici strain Fol002 Fol002_contig_23, whole genome shotgun sequence	fungi'
MAMF01000123.1 Fusarium oxysporum f. sp. melonis strain Fom016 Fom016pilon_contig_123, whole genome shotgun sequence	fungi'
MABT01000031.1 Fusarium oxysporum f. cucumerinum strain Foc011 Foq011_contig_31, whole genome shotgun sequence	fungi'
LQRL01150276.1 Hydroides elegans strain CA01 H_elegans_150303, whole genome shotgun sequence	invertebrates'
CP016880.1 Neisseria meningitidis strain M07165 genome	bacteria'
CP016882.1 Neisseria meningitidis strain M22747 genome	bacteria'
CP016881.1 Neisseria meningitidis strain M07999 genome	bacteria'
CP016883.1 Neisseria meningitidis strain M22790 genome	bacteria'
CP016884.1 Neisseria meningitidis strain M22797 genome	bacteria'
CP016886.1 Neisseria meningitidis strain M25074 genome	bacteria'
CP016885.1 Neisseria meningitidis strain M25073 genome	bacteria'
CM004556.1 Fusarium pseudograminearum strain RBG5266 chromosome 1, whole genome shotgun sequence	fungi'
CM004562.1 Capra hircus breed San Clemente chromosome 1, whole genome shotgun sequence	vertebrates_mammals'
MBPS01000051.1 Fusarium fujikuroi strain CF-295141 Contig0000051, whole genome shotgun sequence	fungi'
CP015855.1 Escherichia coli strain EDL933-1 genome	bacteria'
MAYM01001094.1 Phytophthora kernoviae isolate Chile2 scf_33878_1080.contig_1, whole genome shotgun sequence	protozoa'
LYOV02000019.1 Homo sapiens isolate HX1 Super-Scaffold_28, whole genome shotgun sequence	vertebrates_mammals'
CP014708.1 Komagataella phaffii strain WT chromosome 1 sequence	fungi'
CP014584.1 Komagataella pastoris strain ATCC 28485 chromosome 1 sequence	fungi'
CP016906.1 Enterobacter cloacae isolate SBP-8 genome	bacteria'
KV757199.1 Cutaneotrichosporon curvatus strain ATCC 20509 unplaced genomic scaffold scaffold_1, whole genome shotgun sequence	fungi'
KV757211.1 Solicoccozyma terricola strain JCM 24523 unplaced genomic scaffold scaffold_1, whole genome shotgun sequence	fungi'
MBAC01000288.1 Phytophthora sp. Chile5 scf_59142_635.contig_1, whole genome shotgun sequence	protozoa'
MBAD01000506.1 Phytophthora kernoviae isolate Chile7 scf_14714_984.contig_1, whole genome shotgun sequence	protozoa'
MBAB01000360.1 Phytophthora kernoviae isolate Chile1 scf_19415_347.contig_1, whole genome shotgun sequence	protozoa'
CM004594.1 Homo sapiens isolate KOREF chromosome 2, whole genome shotgun sequence	vertebrates_mammals'
MBDO01000001.1 Phytophthora kernoviae isolate Chile6 scf_58185_5.contig_1, whole genome shotgun sequence	protozoa'
KV757425.1 Phytophthora kernoviae isolate Chile4 unplaced genomic scaffold scf_64197_2, whole genome shotgun sequence	protozoa'
CP013830.1 Pasteurellaceae bacterium NI1060 genome	bacteria'
CP016045.1 Treponema pallidum subsp. pallidum strain PT_SIF0697 genome	bacteria'
CP016047.1 Treponema pallidum subsp. pallidum strain PT_SIF0857 genome	bacteria'
CP016049.1 Treponema pallidum subsp. pallidum strain PT_SIF0908 genome	bacteria'
CP016050.1 Treponema pallidum subsp. pallidum strain PT_SIF0954 genome	bacteria'
CP016051.1 Treponema pallidum subsp. pallidum strain PT_SIF1002 genome	bacteria'
CP016052.1 Treponema pallidum subsp. pallidum strain PT_SIF1020 genome	bacteria'
CP016053.1 Treponema pallidum subsp. pallidum strain PT_SIF1063 genome	bacteria'
CP016054.1 Treponema pallidum subsp. pallidum strain PT_SIF1127 genome	bacteria'
CP016055.1 Treponema pallidum subsp. pallidum strain PT_SIF1135 genome	bacteria'
CP016056.1 Treponema pallidum subsp. pallidum strain PT_SIF1140 genome	bacteria'
CP016057.1 Treponema pallidum subsp. pallidum strain PT_SIF1142 genome	bacteria'
CP016058.1 Treponema pallidum subsp. pallidum strain PT_SIF1156 genome	bacteria'
CP016059.1 Treponema pallidum subsp. pallidum strain PT_SIF1167 genome	bacteria'
CP016060.1 Treponema pallidum subsp. pallidum strain PT_SIF1183 genome	bacteria'
CP016061.1 Treponema pallidum subsp. pallidum strain PT_SIF1196 genome	bacteria'
CP016062.1 Treponema pallidum subsp. pallidum strain PT_SIF1200 genome	bacteria'
CP016063.1 Treponema pallidum subsp. pallidum strain PT_SIF1242 genome	bacteria'
CP016064.1 Treponema pallidum subsp. pallidum strain PT_SIF1252 genome	bacteria'
CP016065.1 Treponema pallidum subsp. pallidum strain PT_SIF1261 genome	bacteria'
CP016066.1 Treponema pallidum subsp. pallidum strain PT_SIF1278 genome	bacteria'
CP016067.1 Treponema pallidum subsp. pallidum strain PT_SIF1280 genome	bacteria'
CP016068.1 Treponema pallidum subsp. pallidum strain PT_SIF1299 genome	bacteria'
CP016069.1 Treponema pallidum subsp. pallidum strain PT_SIF1348 genome	bacteria'
LZYO01000002.1 Paracoccidioides brasiliensis strain Pb300 supercont1.2, whole genome shotgun sequence	fungi'
LYUC01000001.1 Paracoccidioides brasiliensis strain CNH supercont1.1, whole genome shotgun sequence	fungi'
MCQI02000131.1 Aspergillus fumigatus strain LMB-35Aa scaffold2_size2524342, whole genome shotgun sequence	fungi'
CP016975.1 Brucella canis strain 2009013648 chromosome 1 sequence	bacteria'
CP016985.1 Brucella melitensis strain 2010724553 chromosome 1 sequence	bacteria'
CP016632.1 Streptococcus pneumoniae strain D122 genome	bacteria'
CP016633.1 Streptococcus pneumoniae strain D141 genome	bacteria'
CP017004.1 Plasmodium falciparum 3D7 chromosome 14 sequence	protozoa'
MCBO01000001.1 Manacus vitellinus isolate BGI_N305 scaffold_0.R, whole genome shotgun sequence	vertebrates_non_mammals'
MAZY01000003.1 Pleurotus eryngii strain 183 scaffold003, whole genome shotgun sequence	fungi'
KV766071.1 Botryosphaeria berengeriana strain LW030101 unplaced genomic scaffold Scaffold1, whole genome shotgun sequence	fungi'
CP017074.1 Luteimonas sp. JM171 genome	bacteria'
JXNT01000001.1 Aspergillus cristatus strain GZAAS20.1005 Scaffold_Scf_1, whole genome shotgun sequence	fungi'
CP016855.1 Staphylococcus aureus subsp. aureus strain 5118.N, complete genome	bacteria'
LHUA01000101.1 Fusarium meridionale strain NRRL28721 contig_131, whole genome shotgun sequence	fungi'
LHTZ01000026.1 Fusarium asiaticum strain NRRL28720 contig_9, whole genome shotgun sequence	fungi'
LHTY01000088.1 Fusarium asiaticum strain NRRL6101 contig_19, whole genome shotgun sequence	fungi'
LHUB01000118.1 Fusarium meridionale strain NRRL28723 contig_21, whole genome shotgun sequence	fungi'
LHUD01000197.1 Fusarium graminearum strain NRRL28336 contig_3, whole genome shotgun sequence	fungi'
LHUC01000016.1 Fusarium graminearum strain DAOM180378 contig_5, whole genome shotgun sequence	fungi'
LJIJ01000001.1 Orchesella cincta Ocin01_Sc0001, whole genome shotgun sequence	invertebrates'
MDGX01000001.1 Brettanomyces bruxellensis strain CBS 2796 y881_scaffold1, whole genome shotgun sequence	fungi'
AWGH01000001.1 Tsuchiyaea wingfieldii CBS 7118 supercont1.1, whole genome shotgun sequence	fungi'
AWGK01000001.1 Cryptococcus depauperatus CBS 7841 supercont1.1, whole genome shotgun sequence	fungi'
AWGJ01000001.1 Cryptococcus amylolentus CBS 6039 supercont1.1, whole genome shotgun sequence	fungi'
MEKH01000001.1 Cryptococcus amylolentus CBS 6273 supercont2.1, whole genome shotgun sequence	fungi'
AWGL01000001.1 Cryptococcus depauperatus CBS 7855 supercont2.1, whole genome shotgun sequence	fungi'
KV766159.1 Penicillium oxalicum strain HP7-1 unplaced genomic scaffold scaffold_1, whole genome shotgun sequence	fungi'
MEIZ01000001.1 Globodera ellingtonae isolate Ge_1 G_ellingtonae_scaf_0001, whole genome shotgun sequence	invertebrates'
CP012555.1 Raoultella ornithinolytica strain 18 sequence	bacteria'
CM004629.1 Vigna angularis var. angularis cultivar Kyungwonpat chromosome 6, whole genome shotgun sequence	plants'
MDVP01000001.1 Crocodylus porosus isolate Cpor-Errol scaffold_1, whole genome shotgun sequence	vertebrates_non_mammals'
MDVQ01000008.1 Gavialis gangeticus isolate Ggan-Ray scaffold_8, whole genome shotgun sequence	vertebrates_non_mammals'
MEDX01000015.1 Fonticula-like sp. SCN 57-25 ABS50_C0015, whole genome shotgun sequence	invertebrates'
MEDY01000042.1 Rhizaria sp. SCN 62-66 ABS51_C0042, whole genome shotgun sequence	protozoa'
CP016418.1 Chlamydia trachomatis strain SB002739 sequence	bacteria'
CP016420.1 Chlamydia trachomatis strain SB006930 sequence	bacteria'
CP016422.1 Chlamydia trachomatis strain SB008107 sequence	bacteria'
CP016424.1 Chlamydia trachomatis strain SB013112 sequence	bacteria'
CP016426.1 Chlamydia trachomatis strain SB013321 sequence	bacteria'
MINK01187594.1 Phormia regina isolate Indy2012f PregF_Contig_187788, whole genome shotgun sequence	invertebrates'
MINJ01166222.1 Phormia regina isolate Indy2012m PregM_Contig_166753, whole genome shotgun sequence	invertebrates'
MEHO01000001.1 Lolium perenne isolate 4540-9 Ryegrass_Norm_contig_4785, whole genome shotgun sequence	plants'
CM004674.1 Saccharomyces cerevisiae strain wild007 chromosome IV, whole genome shotgun sequence	fungi'
CM004690.1 Saccharomyces cerevisiae strain wild003 chromosome IV, whole genome shotgun sequence	fungi'
CM004706.1 Saccharomyces cerevisiae strain wild005 chromosome IV, whole genome shotgun sequence	fungi'
CM004738.1 Saccharomyces cerevisiae strain wild004 chromosome IV, whole genome shotgun sequence	fungi'
CM004722.1 Saccharomyces cerevisiae strain wild006 chromosome IV, whole genome shotgun sequence	fungi'
CM004754.1 Saccharomyces cerevisiae strain wild002 chromosome IV, whole genome shotgun sequence	fungi'
CM004770.1 Saccharomyces cerevisiae strain wild001 chromosome IV, whole genome shotgun sequence	fungi'
CM004802.1 Saccharomyces cerevisiae strain wine019 chromosome IV, whole genome shotgun sequence	fungi'
CM004786.2 Saccharomyces cerevisiae strain wine018 chromosome IV, whole genome shotgun sequence	fungi'
CM004818.2 Saccharomyces cerevisiae strain wine017 chromosome IV, whole genome shotgun sequence	fungi'
CM004834.2 Saccharomyces cerevisiae strain wine016 chromosome IV, whole genome shotgun sequence	fungi'
CM004850.2 Saccharomyces cerevisiae strain wine015 chromosome IV, whole genome shotgun sequence	fungi'
CM004866.2 Saccharomyces cerevisiae strain wine014 chromosome IV, whole genome shotgun sequence	fungi'
CM004882.2 Saccharomyces cerevisiae strain wine013 chromosome IV, whole genome shotgun sequence	fungi'
CM004898.1 Saccharomyces cerevisiae strain wine012 chromosome IV, whole genome shotgun sequence	fungi'
CM004914.2 Saccharomyces cerevisiae strain wine011 chromosome IV, whole genome shotgun sequence	fungi'
CM004930.2 Saccharomyces cerevisiae strain wine010 chromosome IV, whole genome shotgun sequence	fungi'
CM004946.2 Saccharomyces cerevisiae strain wine009 chromosome IV, whole genome shotgun sequence	fungi'
CM004962.2 Saccharomyces cerevisiae strain wine008 chromosome IV, whole genome shotgun sequence	fungi'
CM004978.2 Saccharomyces cerevisiae strain wine007 chromosome IV, whole genome shotgun sequence	fungi'
CM004994.2 Saccharomyces cerevisiae strain wine006 chromosome IV, whole genome shotgun sequence	fungi'
CM005010.2 Saccharomyces cerevisiae strain wine005 chromosome IV, whole genome shotgun sequence	fungi'
CM005026.2 Saccharomyces cerevisiae strain wine004 chromosome IV, whole genome shotgun sequence	fungi'
CM005042.2 Saccharomyces cerevisiae strain wine003 chromosome IV, whole genome shotgun sequence	fungi'
CM005058.2 Saccharomyces cerevisiae strain wine002 chromosome IV, whole genome shotgun sequence	fungi'
CM005074.2 Saccharomyces cerevisiae strain wine001 chromosome IV, whole genome shotgun sequence	fungi'
CM005090.1 Saccharomyces cerevisiae strain spirits011 chromosome IV, whole genome shotgun sequence	fungi'
CM005106.1 Saccharomyces cerevisiae strain spirits010 chromosome IV, whole genome shotgun sequence	fungi'
CM005122.1 Saccharomyces cerevisiae strain spirits009 chromosome IV, whole genome shotgun sequence	fungi'
CM005138.1 Saccharomyces cerevisiae strain spirits008 chromosome IV, whole genome shotgun sequence	fungi'
CM005154.1 Saccharomyces cerevisiae strain spirits007 chromosome IV, whole genome shotgun sequence	fungi'
CM005170.1 Saccharomyces cerevisiae strain spirits006 chromosome IV, whole genome shotgun sequence	fungi'

CM005186.1 Saccharomyces cerevisiae strain spirits005 chromosome IV, whole genome shotgun sequence	fungi'
CM005218.1 Saccharomyces cerevisiae strain spirits004 chromosome IV, whole genome shotgun sequence	fungi'
CM005202.1 Saccharomyces cerevisiae strain spirits003 chromosome IV, whole genome shotgun sequence	fungi'
CM005234.1 Saccharomyces cerevisiae strain spirits002 chromosome IV, whole genome shotgun sequence	fungi'
CM005250.2 Saccharomyces cerevisiae strain spirits001 chromosome IV, whole genome shotgun sequence	fungi'
CM005266.1 Saccharomyces cerevisiae strain sake007 chromosome IV, whole genome shotgun sequence	fungi'
CM005282.1 Saccharomyces cerevisiae strain sake006 chromosome IV, whole genome shotgun sequence	fungi'
CM005298.1 Saccharomyces cerevisiae strain sake005 chromosome IV, whole genome shotgun sequence	fungi'
CM005314.1 Saccharomyces cerevisiae strain sake004 chromosome IV, whole genome shotgun sequence	fungi'
CM005330.1 Saccharomyces cerevisiae strain sake003 chromosome IV, whole genome shotgun sequence	fungi'
CM005346.1 Saccharomyces cerevisiae strain sake002 chromosome IV, whole genome shotgun sequence	fungi'
CM005362.1 Saccharomyces cerevisiae strain sake001 chromosome IV, whole genome shotgun sequence	fungi'
CM005378.1 Saccharomyces cerevisiae strain laboratory002 chromosome IV, whole genome shotgun sequence	fungi'
CM005394.1 Saccharomyces cerevisiae strain laboratory001 chromosome IV, whole genome shotgun sequence	fungi'
CM005426.1 Saccharomyces cerevisiae strain bread004 chromosome IV, whole genome shotgun sequence	fungi'
CM005442.1 Saccharomyces cerevisiae strain bread003 chromosome IV, whole genome shotgun sequence	fungi'
CM005410.1 Saccharomyces cerevisiae strain bread002 chromosome IV, whole genome shotgun sequence	fungi'
CM005506.1 Saccharomyces cerevisiae strain bread001 chromosome IV, whole genome shotgun sequence	fungi'
CM005458.1 Saccharomyces cerevisiae strain bioethanol005 chromosome IV, whole genome shotgun sequence	fungi'
CM005490.1 Saccharomyces cerevisiae strain bioethanol004 chromosome IV, whole genome shotgun sequence	fungi'
CM005474.1 Saccharomyces cerevisiae strain bioethanol003 chromosome IV, whole genome shotgun sequence	fungi'
CM005522.1 Saccharomyces cerevisiae strain bioethanol002 chromosome IV, whole genome shotgun sequence	fungi'
CM005538.1 Saccharomyces cerevisiae strain bioethanol001 chromosome IV, whole genome shotgun sequence	fungi'
CM005554.1 Saccharomyces cerevisiae strain beer102 chromosome IV, whole genome shotgun sequence	fungi'
CM005570.1 Saccharomyces cerevisiae strain beer101 chromosome IV, whole genome shotgun sequence	fungi'
CM005602.1 Saccharomyces cerevisiae strain beer100 chromosome IV, whole genome shotgun sequence	fungi'
CM005586.1 Saccharomyces cerevisiae strain beer099 chromosome IV, whole genome shotgun sequence	fungi'
CM005618.1 Saccharomyces cerevisiae strain beer098 chromosome IV, whole genome shotgun sequence	fungi'
CM005634.1 Saccharomyces cerevisiae strain beer097 chromosome IV, whole genome shotgun sequence	fungi'
CM005666.1 Saccharomyces cerevisiae strain beer096 chromosome IV, whole genome shotgun sequence	fungi'
CM005650.1 Saccharomyces cerevisiae strain beer095 chromosome IV, whole genome shotgun sequence	fungi'
CM005682.1 Saccharomyces cerevisiae strain beer094 chromosome IV, whole genome shotgun sequence	fungi'
CM005698.1 Saccharomyces cerevisiae strain beer093 chromosome IV, whole genome shotgun sequence	fungi'
CM005714.1 Saccharomyces cerevisiae strain beer092 chromosome IV, whole genome shotgun sequence	fungi'
CM005730.1 Saccharomyces cerevisiae strain beer091 chromosome IV, whole genome shotgun sequence	fungi'
CM005746.1 Saccharomyces cerevisiae strain beer090 chromosome IV, whole genome shotgun sequence	fungi'
CM005762.1 Saccharomyces cerevisiae strain beer089 chromosome IV, whole genome shotgun sequence	fungi'
CM005778.1 Saccharomyces cerevisiae strain beer088 chromosome IV, whole genome shotgun sequence	fungi'
CM005794.1 Saccharomyces cerevisiae strain beer087 chromosome IV, whole genome shotgun sequence	fungi'
CM005810.1 Saccharomyces cerevisiae strain beer086 chromosome IV, whole genome shotgun sequence	fungi'
CM005826.1 Saccharomyces cerevisiae strain beer085 chromosome IV, whole genome shotgun sequence	fungi'
CM005842.1 Saccharomyces cerevisiae strain beer084 chromosome IV, whole genome shotgun sequence	fungi'
CM005858.1 Saccharomyces cerevisiae strain beer083 chromosome IV, whole genome shotgun sequence	fungi'
CM005874.1 Saccharomyces cerevisiae strain beer082 chromosome IV, whole genome shotgun sequence	fungi'
CM005890.1 Saccharomyces cerevisiae strain beer081 chromosome IV, whole genome shotgun sequence	fungi'
CM005906.1 Saccharomyces cerevisiae strain beer080 chromosome IV, whole genome shotgun sequence	fungi'
CM005922.1 Saccharomyces cerevisiae strain beer079 chromosome IV, whole genome shotgun sequence	fungi'
CM005938.1 Saccharomyces cerevisiae strain beer078 chromosome IV, whole genome shotgun sequence	fungi'
CM005954.1 Saccharomyces cerevisiae strain beer077 chromosome IV, whole genome shotgun sequence	fungi'
CM005970.1 Saccharomyces cerevisiae strain beer076 chromosome IV, whole genome shotgun sequence	fungi'
CM005986.1 Saccharomyces cerevisiae strain beer075 chromosome IV, whole genome shotgun sequence	fungi'
CM006002.1 Saccharomyces cerevisiae strain beer074 chromosome IV, whole genome shotgun sequence	fungi'
CM006018.1 Saccharomyces cerevisiae strain beer073 chromosome IV, whole genome shotgun sequence	fungi'
CM006034.1 Saccharomyces cerevisiae strain beer072 chromosome IV, whole genome shotgun sequence	fungi'
CM006050.1 Saccharomyces cerevisiae strain beer071 chromosome IV, whole genome shotgun sequence	fungi'
CM006066.1 Saccharomyces cerevisiae strain beer070 chromosome IV, whole genome shotgun sequence	fungi'
CM006082.1 Saccharomyces cerevisiae strain beer069 chromosome IV, whole genome shotgun sequence	fungi'
CM006098.1 Saccharomyces cerevisiae strain beer068 chromosome IV, whole genome shotgun sequence	fungi'
CM006114.1 Saccharomyces cerevisiae strain beer067 chromosome IV, whole genome shotgun sequence	fungi'
CM006130.1 Saccharomyces cerevisiae strain beer066 chromosome IV, whole genome shotgun sequence	fungi'
CM006146.1 Saccharomyces cerevisiae strain beer065 chromosome IV, whole genome shotgun sequence	fungi'
CM006162.1 Saccharomyces cerevisiae strain beer064 chromosome IV, whole genome shotgun sequence	fungi'
CM006178.1 Saccharomyces cerevisiae strain beer063 chromosome IV, whole genome shotgun sequence	fungi'
CM006194.1 Saccharomyces cerevisiae strain beer062 chromosome IV, whole genome shotgun sequence	fungi'
CM006210.1 Saccharomyces cerevisiae strain beer061 chromosome IV, whole genome shotgun sequence	fungi'
CM006226.1 Saccharomyces cerevisiae strain beer060 chromosome IV, whole genome shotgun sequence	fungi'
CM006242.1 Saccharomyces cerevisiae strain beer059 chromosome IV, whole genome shotgun sequence	fungi'
CM006258.1 Saccharomyces cerevisiae strain beer058 chromosome IV, whole genome shotgun sequence	fungi'
CM006274.1 Saccharomyces cerevisiae strain beer057 chromosome IV, whole genome shotgun sequence	fungi'
CM006290.1 Saccharomyces cerevisiae strain beer056 chromosome IV, whole genome shotgun sequence	fungi'
CM006306.1 Saccharomyces cerevisiae strain beer055 chromosome IV, whole genome shotgun sequence	fungi'
CM006322.1 Saccharomyces cerevisiae strain beer054 chromosome IV, whole genome shotgun sequence	fungi'
CM006338.1 Saccharomyces cerevisiae strain beer053 chromosome IV, whole genome shotgun sequence	fungi'
CM006354.1 Saccharomyces cerevisiae strain beer052 chromosome IV, whole genome shotgun sequence	fungi'
CM006370.1 Saccharomyces cerevisiae strain beer051 chromosome IV, whole genome shotgun sequence	fungi'
CM006386.1 Saccharomyces cerevisiae strain beer050 chromosome IV, whole genome shotgun sequence	fungi'
CM006402.1 Saccharomyces cerevisiae strain beer049 chromosome IV, whole genome shotgun sequence	fungi'
CM006418.1 Saccharomyces cerevisiae strain beer048 chromosome IV, whole genome shotgun sequence	fungi'
CM006434.1 Saccharomyces cerevisiae strain beer047 chromosome IV, whole genome shotgun sequence	fungi'
CM006450.1 Saccharomyces cerevisiae strain beer046 chromosome IV, whole genome shotgun sequence	fungi'
CM006466.1 Saccharomyces cerevisiae strain beer045 chromosome IV, whole genome shotgun sequence	fungi'
CM006482.1 Saccharomyces cerevisiae strain beer044 chromosome IV, whole genome shotgun sequence	fungi'
CM006498.1 Saccharomyces cerevisiae strain beer043 chromosome IV, whole genome shotgun sequence	fungi'
CM006514.1 Saccharomyces cerevisiae strain beer042 chromosome IV, whole genome shotgun sequence	fungi'
CM006530.1 Saccharomyces cerevisiae strain beer041 chromosome IV, whole genome shotgun sequence	fungi'
CM006546.1 Saccharomyces cerevisiae strain beer040 chromosome IV, whole genome shotgun sequence	fungi'
CM006562.1 Saccharomyces cerevisiae strain beer039 chromosome IV, whole genome shotgun sequence	fungi'
CM006594.1 Saccharomyces cerevisiae strain beer038 chromosome IV, whole genome shotgun sequence	fungi'
CM006578.1 Saccharomyces cerevisiae strain beer037 chromosome IV, whole genome shotgun sequence	fungi'
CM006610.1 Saccharomyces cerevisiae strain beer036 chromosome IV, whole genome shotgun sequence	fungi'
CM006626.1 Saccharomyces cerevisiae strain beer035 chromosome IV, whole genome shotgun sequence	fungi'
CM006642.1 Saccharomyces cerevisiae strain beer034 chromosome IV, whole genome shotgun sequence	fungi'
CM006674.1 Saccharomyces cerevisiae strain beer033 chromosome IV, whole genome shotgun sequence	fungi'
CM006658.1 Saccharomyces cerevisiae strain beer032 chromosome IV, whole genome shotgun sequence	fungi'
CM006690.1 Saccharomyces cerevisiae strain beer031 chromosome IV, whole genome shotgun sequence	fungi'
CM006706.1 Saccharomyces cerevisiae strain beer030 chromosome IV, whole genome shotgun sequence	fungi'
CM006738.1 Saccharomyces cerevisiae strain beer029 chromosome IV, whole genome shotgun sequence	fungi'
CM006722.1 Saccharomyces cerevisiae strain beer028 chromosome IV, whole genome shotgun sequence	fungi'
CM006754.1 Saccharomyces cerevisiae strain beer027 chromosome IV, whole genome shotgun sequence	fungi'
CM006770.1 Saccharomyces cerevisiae strain beer026 chromosome IV, whole genome shotgun sequence	fungi'
CM006802.1 Saccharomyces cerevisiae strain beer025 chromosome IV, whole genome shotgun sequence	fungi'
CM006786.1 Saccharomyces cerevisiae strain beer024 chromosome IV, whole genome shotgun sequence	fungi'
CM006818.1 Saccharomyces cerevisiae strain beer023 chromosome IV, whole genome shotgun sequence	fungi'
CM006834.1 Saccharomyces cerevisiae strain beer022 chromosome IV, whole genome shotgun sequence	fungi'
CM006850.1 Saccharomyces cerevisiae strain beer021 chromosome IV, whole genome shotgun sequence	fungi'
CM006866.1 Saccharomyces cerevisiae strain beer020 chromosome IV, whole genome shotgun sequence	fungi'
CM006882.1 Saccharomyces cerevisiae strain beer019 chromosome IV, whole genome shotgun sequence	fungi'
CM006898.1 Saccharomyces cerevisiae strain beer018 chromosome IV, whole genome shotgun sequence	fungi'
CM006930.1 Saccharomyces cerevisiae strain beer017 chromosome IV, whole genome shotgun sequence	fungi'
CM006914.1 Saccharomyces cerevisiae strain beer016 chromosome IV, whole genome shotgun sequence	fungi'
CM006946.1 Saccharomyces cerevisiae strain beer015 chromosome IV, whole genome shotgun sequence	fungi'
CM006978.1 Saccharomyces cerevisiae strain beer014 chromosome IV, whole genome shotgun sequence	fungi'
CM006962.1 Saccharomyces cerevisiae strain beer013 chromosome IV, whole genome shotgun sequence	fungi'
CM006994.1 Saccharomyces cerevisiae strain beer012 chromosome IV, whole genome shotgun sequence	fungi'
CM007010.1 Saccharomyces cerevisiae strain beer011 chromosome IV, whole genome shotgun sequence	fungi'
CM007026.1 Saccharomyces cerevisiae strain beer010 chromosome IV, whole genome shotgun sequence	fungi'
CM007042.1 Saccharomyces cerevisiae strain beer009 chromosome IV, whole genome shotgun sequence	fungi'
CM007058.1 Saccharomyces cerevisiae strain beer008 chromosome IV, whole genome shotgun sequence	fungi'
CM007074.1 Saccharomyces cerevisiae strain beer007 chromosome IV, whole genome shotgun sequence	fungi'
CM007090.1 Saccharomyces cerevisiae strain beer006 chromosome IV, whole genome shotgun sequence	fungi'
CM007106.1 Saccharomyces cerevisiae strain beer005 chromosome IV, whole genome shotgun sequence	fungi'
CM007122.1 Saccharomyces cerevisiae strain beer004 chromosome IV, whole genome shotgun sequence	fungi'
CM007138.1 Saccharomyces cerevisiae strain beer003 chromosome IV, whole genome shotgun sequence	fungi'
CM007154.1 Saccharomyces cerevisiae strain beer001 chromosome IV, whole genome shotgun sequence	fungi'
CM007170.1 Saccharomyces cerevisiae strain beer002 chromosome IV, whole genome shotgun sequence	fungi'
LMYC01000314.1 Aspergillus niger strain A1 scf7180000037689, whole genome shotgun sequence	fungi'
KV766587.1 Arabidopsis thaliana ecotype Nd-1 unplaced genomic scaffold scaffold5230, whole genome shotgun sequence	plants'
BDCQ01000010.1 Chenopodium quinoa DNA, contig: Cqu_c00010.1, strain: Experimental strain, Kd, whole genome shotgun sequence	plants'
BDGN01002080.1 Pleurotus salmoneostramineus DNA, contig: Ps_contig_2080, strain: NBRC 31859, whole genome shotgun sequence	fungi'
DF977685.1 Cladosiphon okamuranus DNA, scaffold: oki-s_mms_scaffold_1, whole genome shotgun sequence	protozoa'
DF973114.1 Trifolium subterraneum DNA, scaffold: Tsud_sc00001.31, whole genome shotgun sequence	plants'
MDVK01000001.1 Brettanomyces custersianus strain CBS 4805 y893_scaffold1, whole genome shotgun sequence	fungi'
CP017298.1 Actinomyces hongkongensis strain HKU8 genome	bacteria'
MDLI01000001.1 Setophaga coronata coronata isolate EE26A12 scaffold1.1_size22943089, whole genome shotgun sequence	vertebrates_non_mammals'
CP014715.1 Komagataella phaffii GS115 chromosome 1 sequence	fungi'
LPNM01000007.1 Hanseniaspora osmophila strain AWRI3579 Hanseniaspora_osmophila_AWRI3579_scaffold7, whole genome shotgun sequence	fungi'
LPNN01000004.1 Hanseniaspora uvarum strain AWRI3580 Hanseniaspora_uvarum_AWRI3580_scaffold4, whole genome shotgun sequence	fungi'
LPNL01000004.1 Hanseniaspora opuntiae strain AWRI3578 Hanseniaspora_opuntiae_AWRI3578_scaffold4, whole genome shotgun sequence	fungi'
MKHR01039359.1 Salpa thompsoni scaffold_39359, whole genome shotgun sequence	invertebrates'
KV784179.1 Penicillium solitum strain NJ1 unplaced genomic scaffold scaffold1, whole genome shotgun sequence	fungi'
KV784220.1 Penicillium sclerotiorum strain 113 unplaced genomic scaffold scaffold1, whole genome shotgun sequence	fungi'
KV878785.1 Penicillium expansum strain R21 unplaced genomic scaffold scaffold2, whole genome shotgun sequence	fungi'
KV784353.1 Fragilariopsis cylindrus CCMP1102 unplaced genomic scaffold FRACYscaffold_1, whole genome shotgun sequence	protozoa'
KV784802.1 Homo sapiens isolate AK1 unplaced genomic scaffold Scaffold0147, whole genome shotgun sequence	vertebrates_mammals'
MCNH01000046.1 Saccharomyces cerevisiae strain GUJ105 scaffold_46, whole genome shotgun sequence	fungi'

CP017312.1 Bacillus subtilis subsp. subtilis strain QB5412 genome	bacteria'
CP017313.1 Bacillus subtilis subsp. subtilis strain QB5413 genome	bacteria'
MEJB01000006.1 Rhazya stricta isolate JS2016 superscaffold6, whole genome shotgun sequence	plants'
LWKS01001599.1 Zaprionus indianus strain IND_ZI_P10 Scaffold1599, whole genome shotgun sequence	invertebrates'
LSBY01000001.1 Chaetomium cochliodes strain CCM F-232 contig00001, whole genome shotgun sequence	fungi'
JXYK01000001.1 Trichosporon faecale strain JCM 2941 scaffold_0001, whole genome shotgun sequence	fungi'
JXYL01000001.1 Trichosporon coremiiforme strain JCM 2938 scaffold_0001, whole genome shotgun sequence	fungi'
JXYM01000001.1 Trichosporon inkin strain JCM 9195 scaffold_0001, whole genome shotgun sequence	fungi'
JXYN01000001.1 Trichosporon ovoides strain JCM 9940 scaffold_0001, whole genome shotgun sequence	fungi'
CP017448.1 Acidihalobacter prosperus strain V6 sequence	bacteria'
MKEJ01104618.1 Embelia ribes isolate ER1 10_Coverage_Contigs_contig_104631, whole genome shotgun sequence	plants'
MDSA01000001.1 Brettanomyces naardenensis strain CBS 7540 y922_scaffold1, whole genome shotgun sequence	fungi'
MDSB01000001.1 Brettanomyces anomalus strain CBS 7654 MyScaffold1, whole genome shotgun sequence	fungi'
MEHF01000001.1 Fusarium oxysporum f. sp. ciceris strain 38-1 Scaffold_0001, whole genome shotgun sequence	fungi'
CP011148.1 Wolbachia endosymbiont of Drosophila incompta strain wInc_Cu genome	bacteria'
CP011149.1 Wolbachia endosymbiont of Drosophila incompta strain wInc_SM genome	bacteria'
CP017557.1 Yarrowia lipolytica strain CLIB89(W29) chromosome 1E, complete sequence	fungi'
CP016046.1 Treponema pallidum subsp. pallidum strain PT_SIF0751 genome	bacteria'
CP016048.1 Treponema pallidum subsp. pallidum strain PT_SIF0877_3 genome	bacteria'
MJCC01000001.1 Phytomonas francai isolate TCC 064 Pfr_1_V1, whole genome shotgun sequence	protozoa'
CP017126.1 Borrelia miyamotoi strain CT13-2396, complete genome	bacteria'
LXJU01000001.1 Penicillium arizonense strain CBS 141311 PENARI_contig001, whole genome shotgun sequence	fungi'
MJBS01000001.1 Colletotrichum orchidophilum strain IMI 309357 Scaffold_1.1, whole genome shotgun sequence	fungi'
LQCH01000001.1 Trichoderma virens strain IMI 304061 Scaffold_1, whole genome shotgun sequence	fungi'
LRBQ01000018.1 Cryptosporidium ubiquitum isolate 39668 contig_1, whole genome shotgun sequence	protozoa'
LRBR01000199.1 Cryptosporidium ubiquitum isolate 39725 contig_30, whole genome shotgun sequence	protozoa'
LRBT01000224.1 Cryptosporidium andersoni isolate 31729 contig_77, whole genome shotgun sequence	protozoa'
LRBU01001307.1 Cryptosporidium andersoni isolate 37034 contig_91, whole genome shotgun sequence	protozoa'
LPXJ01007130.1 Ziziphus jujuba cultivar Junzao scaffold22481, whole genome shotgun sequence	plants'
MLAK01000001.1 Tritrichomonas foetus strain K scaffold_1, whole genome shotgun sequence	protozoa'
MIMC01000001.1 Bactrocera latifrons isolate USDA-ARS-PBARC rearing strain scaffold_0, whole genome shotgun sequence	invertebrates'
MBSD02000490.1 Magnaporthe oryzae strain RMg-Dl NODE_1_length_186500_cov_135.817_ID_4246047, whole genome shotgun sequence	fungi'
CP017712.1 Lactobacillus fermentum strain 47-7 genome	bacteria'
CP017708.1 Moorea producens JHB sequence	bacteria'
CP015054.1 Kluyveromyces marxianus strain CBS4857 chromosome 1 sequence	fungi'
MAMS01010534.1 Bemisia tabaci isolate MEAM1 Scaffold52, whole genome shotgun sequence	invertebrates'
KV841770.1 Colletotrichum incanum strain MAFF238712 unplaced genomic scaffold CDEM12_scaffold24_25.8, whole genome shotgun sequence	fungi'
JUFY01034462.1 Leptopilina clavipes strain GBW scf7180005174957, whole genome shotgun sequence	invertebrates'
CM007353.1 Lactococcus lactis subsp. cremoris IBB477 chromosome, whole genome shotgun sequence	bacteria'
KV844967.1 Gossypium barbadense cultivar 3-79 unplaced genomic scaffold scaffold10038, whole genome shotgun sequence	plants'
LXKZ01000076.1 Endoconidiophora polonica isolate CBS100205 Contig0000076, whole genome shotgun sequence	fungi'
LXJY01000001.1 Myzus persicae strain clone G006 scaffold_0, whole genome shotgun sequence	invertebrates'
LWME01000129.1 Pieris rapae scaffold195_cov84, whole genome shotgun sequence	invertebrates'
KV860272.1 Panthera pardus isolate Maewha unplaced genomic scaffold scaffold1, whole genome shotgun sequence	vertebrates_mammals'
JRPV01000997.1 Venturia inaequalis isolate ICMP 13258 scaffold_961, whole genome shotgun sequence	fungi'
CP017814.1 Sclerotinia sclerotiorum chromosome 1, complete sequence	fungi'
CM007488.1 Oreochromis niloticus isolate F11D_XX linkage group LG7, whole genome shotgun sequence	vertebrates_non_mammals'
JPRY01001728.1 Plasmodium falciparum 58.1 ctg7180000009723, whole genome shotgun sequence	protozoa'
JPRZ01000002.1 Plasmodium falciparum 303.1 ctg7180000004619, whole genome shotgun sequence	protozoa'
JPSA01000004.1 Plasmodium falciparum 309.1 ctg7180000005770, whole genome shotgun sequence	protozoa'
JPSE01007205.1 Plasmodium falciparum 365.1 ctg7180000145967, whole genome shotgun sequence	protozoa'
JPSI01001686.1 Plasmodium falciparum 397.1 ctg7180000006491, whole genome shotgun sequence	protozoa'
JPSC01002356.1 Plasmodium falciparum 326.1 ctg7180000009535, whole genome shotgun sequence	protozoa'
LRBP01000017.1 Cryptosporidium ubiquitum isolate 39726 contig_4, whole genome shotgun sequence	protozoa'
LRBS01000067.1 Cryptosporidium andersoni isolate 30847 contig_28, whole genome shotgun sequence	protozoa'
CM007361.1 Lupinus angustifolius cultivar Tanjil chromosome LG01, whole genome shotgun sequence	plants'
MODV01004802.1 Apostichopus japonicus isolate green-color scaffold6394_cov126, whole genome shotgun sequence	invertebrates'
CP017675.1 Candidatus Gloeomargarita lithophora strain D10 genome	bacteria'
MIEF01009229.1 Taenia solium isolate TsM TsM_Contig5113, whole genome shotgun sequence	invertebrates'
MOXI01000001.1 Tympanuchus cupido pinnatus isolate GPC 3440 Scf7LSr2_1, whole genome shotgun sequence	vertebrates_non_mammals'
MPCS01000039.1 Nannochloropsis oceanica strain IMET1 scaffold00043, whole genome shotgun sequence	protozoa'
CP017964.1 Pseudomonas protegens strain UCT genome	bacteria'
MKPU01000001.1 Amphiamblys sp. WSBS2006 Amphi_1, whole genome shotgun sequence	fungi'
CM007387.1 Asparagus officinalis chromosome 7, whole genome shotgun sequence	plants'
CP017979.1 Escherichia coli str. K-12 substr. W3110 substrain ZK126 genome	bacteria'
CP017951.1 Pseudomonas fluorescens strain CREA-C16 genome	bacteria'
CM007397.1 Nicotiana attenuata strain UT chromosome 7, whole genome shotgun sequence	plants'
KV875093.1 Coniochaeta ligniaria NRRL 30616 unplaced genomic scaffold CONLIGscaffold_1, whole genome shotgun sequence	fungi'
BDFN01001043.1 Ipomoea nil DNA, scaffold: scaffold1043, cultivar: Tokyo-kokei standard, whole genome shotgun sequence	plants'
KV875228.1 Calonectria pseudoreteaudii strain YA51 unplaced genomic scaffold Scaffold1, whole genome shotgun sequence	fungi'
LVVM01002679.1 Rhizopogon vesiculosus strain AM-OR11-056 contig22119, whole genome shotgun sequence	fungi'
CM007422.1 Lycaon pictus isolate Kenya WGF20 chromosome X, whole genome shotgun sequence	vertebrates_mammals'
LGTZ01000001.1 Blastomyces percursus strain EI222 supercont2.1, whole genome shotgun sequence	fungi'
LGRN01000001.1 Emergomyces pasteuriana Ep9510 strain UAMH 9510 supercont1.1, whole genome shotgun sequence	fungi'
MNUE01000001.1 Diplodia corticola strain CBS 112549 scaffold1, whole genome shotgun sequence	fungi'
JRZF01001096.1 Angiostrongylus cantonensis isolate YHS_UMHIR_AC2014 AC1096, whole genome shotgun sequence	invertebrates'
MKKT01000001.1 Ensete ventricosum cultivar Onjamo scf_14867_1.contig_1, whole genome shotgun sequence	plants'
MKLA02000244.1 Plasmodium brasilianum strain Bolivian I contig_268, whole genome shotgun sequence	protozoa'
LYUU01002088.1 Wolbachia endosymbiont of Armadillidium vulgare isolate WXf-1543 Wxf_contig000001, whole genome shotgun sequence	invertebrates'
CP015518.1 Pelobacter acetylenicus strain DSM 3247 genome	bacteria'
CM007505.1 Falco peregrinus chromosome 1, whole genome shotgun sequence	vertebrates_non_mammals'
CM007525.1 Columba livia breed Danish Tumbler chromosome 1, whole genome shotgun sequence	vertebrates_non_mammals'
MIQA01059644.1 Sclerospora graminicola isolate UoM-SG-Pathotype1 jcf7180002100325, whole genome shotgun sequence	protozoa'
CM007575.1 Lycaon pictus isolate South Africa Ananku chromosome X, whole genome shotgun sequence	vertebrates_mammals'
LVEH01000038.1 Rhinolophus sinicus isolate Zhonghua_01 scaffold38, whole genome shotgun sequence	vertebrates_mammals'
MPIL01001537.1 Fusarium oxysporum f. melongenae strain 14004 scaffold_001537, whole genome shotgun sequence	fungi'
CP018148.1 Listeria monocytogenes strain VIMVR081 genome	bacteria'
CP018149.1 Listeria monocytogenes strain VIMHA007 genome	bacteria'
CM007596.1 Oryza sativa Indica Group cultivar IR8 chromosome 1, whole genome shotgun sequence	plants'
KV878679.1 Aspergillus brasiliensis CBS 101740 unplaced genomic scaffold ASPBRscaffold_1, whole genome shotgun sequence	fungi'
JXIK01000001.1 Hipposideros armiger isolate ML-2016 scaffold_0, whole genome shotgun sequence	vertebrates_mammals'
KV878336.1 Aspergillus zonatus CBS 506.65 unplaced genomic scaffold ASPZOscaffold_1, whole genome shotgun sequence	fungi'
KV878125.1 Aspergillus versicolor CBS 583.65 unplaced genomic scaffold ASPVEscaffold_1, whole genome shotgun sequence	fungi'
KV878236.1 Aspergillus foetidus CBS 106.47 unplaced genomic scaffold ASPFOscaffold_1, whole genome shotgun sequence	fungi'
KV878582.1 Aspergillus sydowii CBS 593.65 unplaced genomic scaffold ASPSYscaffold_1, whole genome shotgun sequence	fungi'
KV878209.1 Aspergillus wentii DTO 134E9 unplaced genomic scaffold ASPWEscaffold_1, whole genome shotgun sequence	fungi'
KV878176.1 Aspergillus tubingensis CBS 134.48 unplaced genomic scaffold ASPTUscaffold_1, whole genome shotgun sequence	fungi'
KV878888.1 Aspergillus glaucus CBS 516.65 unplaced genomic scaffold ASPGLscaffold_1, whole genome shotgun sequence	fungi'
KV878970.1 Aspergillus aculeatus ATCC 16872 unplaced genomic scaffold ASPACscaffold_1, whole genome shotgun sequence	fungi'
KV880534.1 Hippocampus comes isolate QL1 unplaced genomic scaffold scaffold7, whole genome shotgun sequence	vertebrates_non_mammals'
CP015758.1 Staphylococcus argenteus strain BN75 genome	bacteria'
MNAD01001539.1 Trametes pubescens strain FBCC735 TRAPUBscaffold_1555, whole genome shotgun sequence	fungi'
MPSW01000575.1 Orbicella faveolata isolate FL scaffold565_size492874, whole genome shotgun sequence	invertebrates'
MIKG01000001.1 Talaromyces amestolkiae strain CIB scaffold1.1, whole genome shotgun sequence	fungi'
MRJL01000001.1 Rhizoctonia solani AG-1 IA strain 1802/KB scaffold1, whole genome shotgun sequence	fungi'
CP018262.1 Borreliella afzelii strain BO23, complete genome	bacteria'
MJAE01000001.1 Venturia effusa isolate 3Des10b scaffold00001, whole genome shotgun sequence	fungi'
LFMY01000001.1 Talaromyces atroroseus strain IBT 11181 scaffold_0, whole genome shotgun sequence	fungi'
CP017834.1 Proteobacteria bacterium Nonnen-W8red strain MWH-Nonnen-W8red genome	bacteria'
MKXE01000004.1 Hevea brasiliensis strain RRIM 600 Scaffold4, whole genome shotgun sequence	plants'
MNBE01000719.1 Penicillium subrubescens strain CBS 132785 PENSUBscaffold_719, whole genome shotgun sequence	fungi'
CP017012.1 Brucella suis strain 2011017258 chromosome 1 sequence	bacteria'
CP018743.1 Pseudomonas sp. DRA525 genome	bacteria'
CP018615.1 Lactobacillus delbrueckii subsp. delbrueckii DSM 20074 = JCM 1012 genome	bacteria'
LHCJ01S017226.1 Sebastes minor isolate S-Minor-200 scaffold17279, whole genome shotgun sequence	vertebrates_non_mammals'
LCWK01S0131098.1 Sebastes steindachneri isolate S-Stein-200 scaffold131098, whole genome shotgun sequence	vertebrates_non_mammals'
LHVS01S000001.1 Sebastes aleutianus isolate Aleu4 scaffold_0, whole genome shotgun sequence	vertebrates_non_mammals'
KV880664.1 Ilyonectria destructans strain C1 unplaced genomic scaffold Scaffold1, whole genome shotgun sequence	fungi'
MLYV01002629.1 Phlebia centrifuga strain FBCC195 PHLCENscaffold_2632, whole genome shotgun sequence	fungi'
CM007615.1 Bacillus licheniformis strain YNP1-TSU chromosome, whole genome shotgun sequence	bacteria'
MSDY01000132.1 Aureobasidium sp. FSWF8-4 FSW8-bin-4-scaffold_1785, whole genome shotgun sequence	fungi'
MSDX01002808.1 Rhodotorula sp. FNED7-22 FNED7-bin-22-scaffold_6273, whole genome shotgun sequence	fungi'
LXTM01000035.1 Barbarea vulgaris isolate G-type Contig34, whole genome shotgun sequence	plants'
CP018861.1 Acinetobacter baumannii strain 11510 genome	bacteria'
CP018810.1 Citrobacter freundii strain BD genome	bacteria'
MRVE01000784.1 Pythium periplocum strain CBS 532.74 Scaffold_784, whole genome shotgun sequence	protozoa'
MRVK01001658.1 Tursiops truncatus isolate MMES2002162SC ScBCFuT_1658, whole genome shotgun sequence	vertebrates_mammals'
LIEC01012159.1 Drosera capensis cultivar wide leaf jcf7180002684130, whole genome shotgun sequence	plants'
LXFE01000126.1 Neolecta irregularis DAH-3 scaffold_495, whole genome shotgun sequence	fungi'
CP016908.1 Pajaroellobacter abortibovis strain BTF92-0548A/99-0131, genome	bacteria'
MPVS01000002.1 Fomitopsis pinicola strain GR9-4 scaffold2, whole genome shotgun sequence	fungi'
MSJD01000151.1 Aspergillus niger strain JSC-093350089 jcf7180000011246, whole genome shotgun sequence	fungi'
CP015998.1 Enterococcus faecalis strain AMB05 genome	bacteria'
MSJG01000942.1 Beauveria bassiana strain IMV 00265 jcf7180000158989, whole genome shotgun sequence	fungi'
MSJH01002665.1 Cladosporium cladosporioides strain IMV 00236 jcf7180000200632, whole genome shotgun sequence	fungi'
MSJI01001949.1 Cladosporium sphaerospermum strain IMV 00045 jcf7180000072763, whole genome shotgun sequence	fungi'
MSJE01000225.1 Aspergillus terreus strain IMV 01167 jcf7180000027533, whole genome shotgun sequence	fungi'
MSJJ01000915.1 Fusarium solani strain IMV 00293 jcf7180000048332, whole genome shotgun sequence	fungi'
MSJK01000007.1 Trichoderma virens strain IMV 00454 jcf7180000006085, whole genome shotgun sequence	fungi'
MSHP01000155.1 Saccharomyces cerevisiae strain BG1 CTBEBG1_scaffold1, whole genome shotgun sequence	fungi'
CP010584.1 Serratia marcescens strain AS1 genome	bacteria'
MNCL01000001.1 Proctacanthus coquilletti flattened_line_0, whole genome shotgun sequence	invertebrates'
KV881464.1 Phytophthora ramorum strain EU1 CC2184 unplaced genomic scaffold scf_9287_1, whole genome shotgun sequence	protozoa'
KV881039.1 Phytophthora ramorum strain EU1 CC14654 unplaced genomic scaffold scf_4263_1, whole genome shotgun sequence	protozoa'
MLIX01000001.1 Phytophthora ramorum strain EU1 CC12475 scf_2902_1.contig_1, whole genome shotgun sequence	protozoa'

KV881840.1 Phytophthora ramorum strain EU1 isolate CC1008 unplaced genomic scaffold scf_63516_1, whole genome shotgun sequence	protozoa'
KV882206.1 Phytophthora ramorum strain EU1 isolate CC1033 unplaced genomic scaffold scf_65018_1, whole genome shotgun sequence	protozoa'
MLJC01000001.1 Phytophthora ramorum strain EU1 isolate CC1048 scf_36722_1.contig_1, whole genome shotgun sequence	protozoa'
MLJD01000001.1 Phytophthora ramorum strain EU1 isolate CC2168 scf_5670_1.contig_1, whole genome shotgun sequence	protozoa'
KV883196.1 Phytophthora ramorum strain EU1 isolate CC2176 unplaced genomic scaffold scf_62851_1, whole genome shotgun sequence	protozoa'
KV883521.1 Phytophthora ramorum strain EU1 isolate CC2186 unplaced genomic scaffold scf_38888_1, whole genome shotgun sequence	protozoa'
MLJG01000001.1 Phytophthora ramorum strain EU1 isolate CC2187 scf_11801_1.contig_1, whole genome shotgun sequence	protozoa'
CP013853.1 Francisella tularensis strain Schu4 F. tul Mut-127 genome	bacteria'
CP012809.1 Saccharomycopsis fibuligera x Saccharomycopsis cf. fibuligera strain KJJ81 chromosome A1, complete sequence	fungi'
CP012823.1 Saccharomycopsis fibuligera strain KPH12 chromosome 1, complete sequence	fungi'
CP015978.1 Saccharomycopsis fibuligera strain ATCC 36309 chromosome 1 sequence	fungi'
MKXH01005430.1 Puccinia striiformis f. sp. tritici strain 38S102 Scaffold_1, whole genome shotgun sequence	fungi'
MPUG01000001.1 Ettlia oleoabundans strain UTEX 1185 scaffold1_size201307, whole genome shotgun sequence	plants'
MPGH01000088.1 Colletotrichum chlorophyti strain NTL11 CCHL11_scaffold88, whole genome shotgun sequence	fungi'
LOQR01000004.1 Saccharomyces cerevisiae strain VTT A-81062 scaffold4, whole genome shotgun sequence	fungi'
MLJV01000001.1 Grosmannia penicillata strain CBS 116008 scaffold1, whole genome shotgun sequence	fungi'
MQRK01218062.1 Girardia tigrina isolate CS2, whole genome shotgun sequence	invertebrates'
MPKX01286338.1 Diuraphis noxia chromosome X, whole genome shotgun sequence	invertebrates'
MQRL01647977.1 Dugesia japonica isolate ML1, whole genome shotgun sequence	invertebrates'
LSRX01000001.1 Symbiodinium microadriaticum strain CCMP2467 Smic.scaffold1, whole genome shotgun sequence	protozoa'
MJVA01001025.1 Talaromyces wortmannii strain LMB-HP14 scaffold1_size1690671, whole genome shotgun sequence	fungi'
MKKQ01004520.1 Trametes polyzona strain LMB-TM5 scaffold7_size427429, whole genome shotgun sequence	fungi'
CM007619.1 Xanthomonas citri pv. citri strain LM199 chromosome, complete sequence, whole genome shotgun sequence	bacteria'
CM007622.1 Xanthomonas citri pv. citri strain LM180 chromosome, complete sequence, whole genome shotgun sequence	bacteria'
CP010422.1 Treponema pallidum subsp. pallidum strain Seattle Nichols genome	bacteria'
CP016401.1 Mycobacterium caprae strain Allgaeu genome	bacteria'
DF978496.1 Acanthaster planci DNA, scaffold: oki_scaffold8, whole genome shotgun sequence	invertebrates'
DF980259.1 Acanthaster planci DNA, scaffold: gbr_scaffold6, whole genome shotgun sequence	invertebrates'
BDGG01000001.1 Ramazzottius varieornatus DNA, contig: Scaffold001, strain: YOKOZUNA-1, whole genome shotgun sequence	invertebrates'
DF983528.1 Zygosaccharomyces rouxii DNA, scaffold: scaffold00001, strain: NBRC1876, whole genome shotgun sequence	fungi'
BCGG01000001.1 Alternaria consortialis DNA, scaffold: scaffold_0, strain: JCM 1940, whole genome shotgun sequence	fungi'
BCGH01000001.1 Trichoderma koningii DNA, scaffold: scaffold_0, strain: JCM 1883, whole genome shotgun sequence	fungi'
BCJR01000001.1 Lyophyllum decastes DNA, scaffold: scaffold_0, strain: JCM 30590, whole genome shotgun sequence	fungi'
BCJS01000001.1 Lyophyllum shimeji DNA, scaffold: scaffold_0, strain: JCM 30591, whole genome shotgun sequence	fungi'
BCKA01000001.1 Penicillium citrinum DNA, scaffold: scaffold_0, strain: JCM 22607, whole genome shotgun sequence	fungi'
BCKB01000001.1 Candida sp. JCM 15000 DNA, scaffold: scaffold_0, strain: JCM 15000, whole genome shotgun sequence	fungi'
BCFM01000001.1 Pythium insidiosum DNA, contig: MCC13_000001, strain: MCC 13, whole genome shotgun sequence	protozoa'
BCFN01000001.1 Pythium insidiosum DNA, contig: ATCC200269_000001, strain: ATCC 200269, whole genome shotgun sequence	protozoa'
BCFO01000001.1 Pythium insidiosum DNA, contig: CBS57385_000001, strain: CBS 573.85, whole genome shotgun sequence	protozoa'
BCFP01000001.1 Pythium insidiosum DNA, contig: CBS101555_000001, strain: CBS 101555, whole genome shotgun sequence	protozoa'
BCFQ01000001.1 Pythium insidiosum DNA, contig: ATC64221_000001, strain: ATCC 64221, whole genome shotgun sequence	protozoa'
BCFR01000001.1 Pythium insidiosum DNA, contig: CR02_000001, strain: CR02, whole genome shotgun sequence	protozoa'
BCFS01000001.1 Pythium insidiosum DNA, contig: Pi19_000001, strain: Pi19, whole genome shotgun sequence	protozoa'
BCFT01000001.1 Pythium insidiosum DNA, contig: MCC18_000001, strain: MCC 18, whole genome shotgun sequence	protozoa'
BCFU01000001.1 Pythium insidiosum DNA, contig: SIMI4763_000001, strain: SIMI 4763, whole genome shotgun sequence	protozoa'
KV884608.1 Daldinia eschscholzii IFB-TL01 unplaced genomic scaffold scaffold_1, whole genome shotgun sequence	fungi'
CP019059.1 Vibrio parahaemolyticus strain 1682 chromosome 1 sequence	bacteria'
CP013857.1 Clostridium botulinum strain CDC_69096, complete genome	bacteria'
MQVQ01000001.1 Colletotrichum sublineola strain CgSl1 scaffold00001, whole genome shotgun sequence	fungi'
CM007627.1 Oryza sativa aus subgroup cultivar N 22::IRGC 19379-1 (IRGC 117534) chromosome 1, whole genome shotgun sequence	plants'
KV884691.1 Monopterus albus unplaced genomic scaffold scaffold3.1, whole genome shotgun sequence	vertebrates_non_mammals'
LSSL01000161.1 Smittium mucronatum strain ALG-7-W6 scaffold-160, whole genome shotgun sequence	fungi'
KV886423.1 Phytophthora ramorum isolate CC1011 unplaced genomic scaffold scf_45908_1, whole genome shotgun sequence	protozoa'
MSTJ01000001.1 Ramalina peruviana strain YAF0012 scaffold_2, whole genome shotgun sequence	fungi'
CP019153.1 Sphingopyxis granuli strain ku-sg sequence	bacteria'
MAYC01000004.1 Pleurotus ostreatus strain CCMSSC00389 scaffold_4, whole genome shotgun sequence	fungi'
CM007645.1 Mycobacterium tuberculosis strain 6548 chromosome, whole genome shotgun sequence	bacteria'
CM007646.1 Mycobacterium tuberculosis strain 410 chromosome, whole genome shotgun sequence	bacteria'
CP019159.1 Corynebacterium pseudotuberculosis strain PA05 genome	bacteria'
CP019289.1 Corynebacterium pseudotuberculosis strain PA06 genome	bacteria'
MPLB01000001.1 Paralichthys olivaceus breed gynogenesis superscaffold20, whole genome shotgun sequence	vertebrates_non_mammals'
LSSN01000002.1 Smittium culicis strain GSMNP scaffold-1, whole genome shotgun sequence	fungi'
LSSM01007749.1 Smittium culicis strain ID-206-W2 scaffold-13652, whole genome shotgun sequence	fungi'
BCHE01000001.1 Sarocladium oryzae DNA, scaffold: scaffold_0, strain: JCM 12450, whole genome shotgun sequence	fungi'
BCII01000001.1 Erythrobasidium hasegawianum DNA, scaffold: scaffold_0, strain: JCM 1545, whole genome shotgun sequence	fungi'
BDDD01000080.1 Cephalotus follicularis DNA, scaffold: scaffold80, isolate: St1, whole genome shotgun sequence	plants'
BCLB01000001.1 Termitomyces sp. JCM 13351 DNA, scaffold: scaffold_0, strain: JCM 13351, whole genome shotgun sequence	fungi'
BDGX01000037.1 Zygosaccharomyces rouxii DNA, scaffold: Scaffold_37, strain: NBRC 110957, whole genome shotgun sequence	fungi'
BCLT01000001.1 Trichosporon asahii DNA, scaffold: scaffold_1, strain: JCM 2466, whole genome shotgun sequence	fungi'
MPGU01000180.1 Capsella bursa-pastoris isolate wt-msu scaffold_100_len4117863_cov0, whole genome shotgun sequence	plants'
MECZ01000001.1 Burkholderia pseudomallei strain MSHR6522 chromosome 1 Chr1a_MSHR6522, whole genome shotgun sequence	bacteria'
AWWV01010840.1 Corchorus capsularis cultivar CVL-1 contig10861, whole genome shotgun sequence	plants'
MTPI01000041.1 Plasmopara viticola isolate JL-7-2 Scaffold_41, whole genome shotgun sequence	protozoa'
CP019435.1 Rickettsia raoultii strain IM16 genome	bacteria'
CP019434.1 Acidihalobacter ferrooxidans strain V8 genome	bacteria'
MSZU01000076.1 Diplodia seriata strain F98.1 scaffold11, whole genome shotgun sequence	fungi'
CP019124.1 Xuhuaishuia manganoxidans strain DY6-4 genome	bacteria'
CP019093.1 Spirodela polyrhiza strain 9509 chromosome 1 sequence	plants'
MQVM01000001.1 Pichia kudriavzevii strain 129 BOH78_Sc001, whole genome shotgun sequence	fungi'
MIPV01008048.1 Amanita bisporigera strain Ingham01 Scaffold_8048, whole genome shotgun sequence	fungi'
MEHY01000011.1 Amanita phalloides strain Alameda01 Scaffold_11, whole genome shotgun sequence	fungi'
CP019420.1 Mycobacterium sp. MS1601, complete genome	bacteria'
CP019490.1 Zygosaccharomyces parabailii strain ATCC 60483 chromosome 1, complete sequence	fungi'
MTKA01000630.1 Castor canadensis isolate Ward scaffold_630, whole genome shotgun sequence	vertebrates_mammals'
CP011180.1 Bordetella pertussis strain F684, complete genome	bacteria'
CP015999.1 Burkholderia sp. KK1 chromosome I sequence	bacteria'
CP018344.1 Prochlorococcus sp. RS50 genome	bacteria'
CP018345.1 Prochlorococcus sp. RS01 genome	bacteria'
CP018346.1 Prochlorococcus sp. RS04 genome	bacteria'
CP011458.1 Vibrio anguillarum strain 4299 chromosome I sequence	bacteria'
CP010036.1 Vibrio anguillarum strain A023 chromosome I sequence	bacteria'
CP011466.1 Vibrio anguillarum strain PF430-3 chromosome I sequence	bacteria'
CP010046.1 Vibrio anguillarum strain 87-9-117 chromosome I sequence	bacteria'
CP010034.1 Vibrio anguillarum strain 91-8-178 chromosome I sequence	bacteria'
CP011470.1 Vibrio anguillarum strain 178/90 chromosome I sequence	bacteria'
CP010032.1 Vibrio anguillarum strain 261/91 chromosome I sequence	bacteria'
CP010030.1 Vibrio anguillarum strain Ba35 chromosome I sequence	bacteria'
CP010040.1 Vibrio anguillarum strain T265 chromosome I sequence	bacteria'
CP011462.1 Vibrio anguillarum strain HI610 chromosome I sequence	bacteria'
CP010042.1 Vibrio anguillarum strain 51/82/2 chromosome I sequence	bacteria'
CP011468.1 Vibrio anguillarum strain LMG12010 chromosome I sequence	bacteria'
CP011472.1 Vibrio anguillarum strain S2 2/9 chromosome I sequence	bacteria'
CP010038.1 Vibrio anguillarum strain 9014/8 chromosome I sequence	bacteria'
CP010044.1 Vibrio anguillarum strain 87-9-116 chromosome I sequence	bacteria'
CP019545.1 Leishmania donovani strain MHOM/IN/1983/AG83 isolate late passage chromosome 36 sequence	protozoa'
CP018604.1 Leishmania donovani strain MHOM/IN/1983/AG83 isolate early passage chromosome 36 sequence	protozoa'
CP010084.1 Vibrio anguillarum strain DSM 21597 chromosome I sequence	bacteria'
CP011475.1 Vibrio anguillarum strain 90-11-287 chromosome I sequence	bacteria'
CP010082.1 Vibrio anguillarum strain 91-7154 chromosome I sequence	bacteria'
CP010076.1 Vibrio anguillarum strain 601/90 chromosome I sequence	bacteria'
CP010291.1 Vibrio anguillarum strain 6018/1 chromosome I sequence	bacteria'
CP010078.1 Vibrio anguillarum strain VA1 chromosome I sequence	bacteria'
CP011438.1 Vibrio anguillarum strain VIB 93 chromosome I sequence	bacteria'
CP019634.1 Vibrio campbellii strain 1114GL chromosome I sequence	bacteria'
CP011464.1 Vibrio anguillarum strain PF7 chromosome I sequence	bacteria'
CP008702.1 Mycobacterium tuberculosis TRS9 genome	bacteria'
CP009198.1 Mycobacterium tuberculosis 1821ADB35 genome	bacteria'
CP009199.1 Mycobacterium tuberculosis 1821ADB36 genome	bacteria'
CP009200.1 Mycobacterium tuberculosis 1821ADB37 genome	bacteria'
CP009201.1 Mycobacterium tuberculosis 1821ADB38 genome	bacteria'
CP009202.1 Mycobacterium tuberculosis 1821ADB40 genome	bacteria'
CP009203.1 Mycobacterium tuberculosis 1821ADB41 genome	bacteria'
CP009204.1 Mycobacterium tuberculosis 1821ADB42 genome	bacteria'
CP009205.1 Mycobacterium tuberculosis 1821ADB44 genome	bacteria'
CP009206.1 Mycobacterium tuberculosis 1821ADB45 genome	bacteria'
CP009207.1 Mycobacterium tuberculosis TRS1 genome	bacteria'
CP009172.1 Mycobacterium tuberculosis TRS16 genome	bacteria'
CP009173.1 Mycobacterium tuberculosis TRS17 genome	bacteria'
CP009174.1 Mycobacterium tuberculosis TRS20 genome	bacteria'
CP009175.1 Mycobacterium tuberculosis TRS13 genome	bacteria'
CP009176.1 Mycobacterium tuberculosis TRS21 genome	bacteria'
CP009177.1 Mycobacterium tuberculosis TRS19 genome	bacteria'
CP009178.1 Mycobacterium tuberculosis TRS7 genome	bacteria'
CP009179.1 Mycobacterium tuberculosis TRS5 genome	bacteria'
CP009180.1 Mycobacterium tuberculosis TRS23 genome	bacteria'
CP009181.1 Mycobacterium tuberculosis TRS14 genome	bacteria'
CP009182.1 Mycobacterium tuberculosis TRS12 genome	bacteria'
CP009184.1 Mycobacterium tuberculosis TRS15 genome	bacteria'
CP009185.1 Mycobacterium tuberculosis TRS18 genome	bacteria'
CP009186.1 Mycobacterium tuberculosis TRS2 genome	bacteria'
CP009188.1 Mycobacterium tuberculosis TRS6 genome	bacteria'
CP009189.1 Mycobacterium tuberculosis TRS4 genome	bacteria'
CP009194.1 Mycobacterium tuberculosis TRS28 genome	bacteria'
CP009196.1 Mycobacterium tuberculosis TRS8 genome	bacteria'
CP009197.1 Mycobacterium tuberculosis TRS11 genome	bacteria'

CP009190.1 Mycobacterium tuberculosis TRS27 genome	bacteria'
CP009191.1 Mycobacterium tuberculosis TRS24 genome	bacteria'
CP009193.1 Mycobacterium tuberculosis TRS26 genome	bacteria'
CP011436.1 Vibrio anguillarum strain VIB 18 chromosome I sequence	bacteria'
CP019666.1 Burkholderia cenocepacia strain VC2307 chromosome 1 sequence	bacteria'
CP019678.1 Burkholderia cenocepacia strain VC1254 chromosome 1, complete sequence	bacteria'
CP009192.1 Mycobacterium tuberculosis TRS25 genome	bacteria'
CP009195.1 Mycobacterium tuberculosis TRS10 genome	bacteria'
CP009187.1 Mycobacterium tuberculosis TRS29 genome	bacteria'
CP009183.1 Mycobacterium tuberculosis TRS22 genome	bacteria'
CP019726.1 Bacillus anthracis strain Sterne 34F2 genome	bacteria'
CCYD01000610.1 Plasmopara halstedii genome assembly, contig: Scaffold_614, whole genome shotgun sequence	protozoa'
CCYT01000001.1 Rhizopus microsporus genome assembly RMATCC62417_Allpaths-LG, scaffold SCAF1, whole genome shotgun sequence	fungi'
CCYA01000265.1 Ceraceosorus bombacis genome assembly, contig: Scaffold_266, whole genome shotgun sequence	fungi'
LK022890.1 Plasmodium chabaudi chabaudi genome assembly PCHAS01, chromosome : 13	protozoa'
LK023128.1 Plasmodium berghei ANKA genome assembly PBANKA01, chromosome : 13	protozoa'
LM993667.1 Plasmodium yoelii genome assembly PY17X01, chromosome : 13	protozoa'
LK934641.1 Plasmodium yoelii genome assembly PYYM01, chromosome : 13	protozoa'
CVKT01007588.1 Echinococcus canadensis genome assembly, contig: E.canG7_contigs_6081, whole genome shotgun sequence	invertebrates'
CVRI01000047.1 Clunio marinus genome assembly, contig: CLUMA_superscaffold_47C, whole genome shotgun sequence	invertebrates'
HG983337.1 Xeromyces bisporus genome assembly Xbisp_v1.0, scaffold scaffold00001, whole genome shotgun sequence	fungi'
HG975513.1 Solanum lycopersicum chromosome ch01, complete genome	plants'
FAXB01000001.1 Microbotryum lychnidis-dioicae genome assembly, contig: MvSl-Chernobyl-1106-1-G10001, whole genome shotgun sequence	fungi'
CZCC01000001.1 Microbotryum lychnidis-dioicae genome assembly, contig: MvSl-443-2-G10001, whole genome shotgun sequence	fungi'
CZCL01000001.1 Microbotryum lychnidis-dioicae genome assembly, contig: MvSl-Chernobyl-1105-1-G10001, whole genome shotgun sequence	fungi'
CZCD01000001.1 Microbotryum lychnidis-dioicae genome assembly, contig: MvSl-Chernobyl-1101-3-A2-G10001, whole genome shotgun sequence	fungi'
CZCM01000001.1 Microbotryum lychnidis-dioicae genome assembly, contig: MvSl-Chernobyl-1162-A1-G10001, whole genome shotgun sequence	fungi'
CZCO01000001.1 Microbotryum lychnidis-dioicae genome assembly, contig: MvSl-576-A2-G10001, whole genome shotgun sequence	fungi'
CZCK01000001.1 Microbotryum lychnidis-dioicae genome assembly, contig: MvSl-Chernobyl-1102-2-G10001, whole genome shotgun sequence	fungi'
CZCE01000001.1 Microbotryum lychnidis-dioicae genome assembly, contig: MvSl-446-2-A2-G10001, whole genome shotgun sequence	fungi'
CZCP01000001.1 Microbotryum lychnidis-dioicae genome assembly, contig: MvSl-Chernobyl-1101-1-A1-G10001, whole genome shotgun sequence	fungi'
CZCN01000001.1 Microbotryum lychnidis-dioicae genome assembly, contig: MvSl-Chernobyl-1164-A2-G10001, whole genome shotgun sequence	fungi'
LN907826.1 Bradyrhizobium sp. G22 genome assembly, chromosome: I	bacteria'
CZCJ01000001.1 Microbotryum lychnidis-dioicae genome assembly, contig: MvSl-Chernobyl-1163-A1-G10001, whole genome shotgun sequence	fungi'
CZCI01000001.1 Microbotryum lychnidis-dioicae genome assembly, contig: MvSl-Chernobyl-1165-1-G10001, whole genome shotgun sequence	fungi'
CZCH01000001.1 Microbotryum lychnidis-dioicae genome assembly, contig: MvSl-Chernobyl-1102-1-G10001, whole genome shotgun sequence	fungi'
CZCF01000001.1 Microbotryum lychnidis-dioicae genome assembly, contig: MvSl-Chernobyl-1103-1-G10001, whole genome shotgun sequence	fungi'
CZCG01000001.1 Microbotryum lychnidis-dioicae genome assembly, contig: MvSl-Chernobyl-1101-2-A1-G10001, whole genome shotgun sequence	fungi'
CZCR01000001.1 Microbotryum lychnidis-dioicae genome assembly, contig: MvSl-Chernobyl-1165-2-A2-G10001, whole genome shotgun sequence	fungi'
FCQG01000001.1 Fusarium proliferatum strain NRRL62905 genome assembly, contig: FPRN_scaffold001, whole genome shotgun sequence	fungi'
FCQH01000001.1 Fusarium mangiferae strain MRC7560 genome assembly, contig: FMAN_scaffold001, whole genome shotgun sequence	fungi'
LT160033.1 Plasmodium berghei strain K173 genome assembly, chromosome: 13	protozoa'
FIZV01054470.1 Trionymus perrisii genome assembly, contig: NODE_5_length_54846_cov_20.3396_ID_9, whole genome shotgun sequence	invertebrates'
LT160614.1 Enterobacter cloacae strain NH52 genome assembly, chromosome: I, whole genome shotgun sequence	bacteria'
FIZR01004686.1 Ferrisia virgata genome assembly, contig: NODE_3_length_322879_cov_35.8404_ID_5, whole genome shotgun sequence	invertebrates'
FIZS01000981.1 Maconellicoccus hirsutus genome assembly, contig: NODE_2_length_393850_cov_27.2878_ID_3, whole genome shotgun sequence	invertebrates'
FIZU01005539.1 Pseudococcus longispinus genome assembly, contig: NODE_6_length_182788_cov_37.1905_ID_11, whole genome shotgun sequence	invertebrates'
FIZT01056129.1 Paracoccus marginatus genome assembly, contig: NODE_2_length_76577_cov_38.5081_ID_3, whole genome shotgun sequence	invertebrates'
FJOF01000001.1 Fusarium proliferatum ET1 genome assembly, contig: FPRO_scaffold001, whole genome shotgun sequence	fungi'
FAOM01576745.1 Triticum aestivum genome assembly Triticum_aestivum_CS42_TGAC_v1, scaffold Triticum_aestivum_CS42_TGACv1_scaffold_576745_7BL, whole genome shotgun sequence	plants'
CVIS01000001.1 Ficedula albicollis genome assembly fAlb_W1.2, scaffold WN0001, whole genome shotgun sequence	vertebrates_non_mammals'
LN813019.1 Halomonas sp. R57-5 genome assembly HalomonasR57-5, chromosome : I	bacteria'
FJOG01000001.1 Phialocephala subalpina strain UAMH 11012 genome assembly, contig: PAC_scaffold_001, whole genome shotgun sequence	fungi'
FJUW01000004.1 Rhynchosporium commune strain UK7 genome assembly, contig: RCO7_scaffold004, whole genome shotgun sequence	fungi'
FJVC01000281.1 Rhynchosporium secalis strain 02CH4-6a.1 genome assembly, contig: RSE6_scaffold0281, whole genome shotgun sequence	fungi'
FJUX01000001.1 Rhynchosporium agropyri strain 04CH-RAC-A.6.1 genome assembly, contig: RAG0_scaffold001, whole genome shotgun sequence	fungi'
FJVB01000001.1 Arabidopsis halleri subsp. gemmifera isolate W302 genome assembly, contig: scaffold_1, whole genome shotgun sequence	plants'
LT554468.1 Absidia glauca strain CBS 101.48 genome assembly, scaffold: scf_12295, whole genome shotgun sequence	fungi'
LT555307.1 Trifolium pratense genome assembly, chromosome: LG2, whole genome shotgun sequence	plants'
HF679023.1 Fusarium fujikuroi IMI 58289 draft genome, chromosome FFUJ_chr01	fungi'
FKKZ01000001.1 Globodera rostochiensis strain Ro1 genome assembly, contig: GROS_00001, whole genome shotgun sequence	invertebrates'
LT558118.1 Ustilago bromivora strain UB2112 genome assembly, chromosome: II	fungi'
FKLU01000001.1 Labrus bergylta genome assembly, contig: LaB_20160104_scaffold_0, whole genome shotgun sequence	vertebrates_non_mammals'
LT593974.1 Halomonas sp. HL-93 genome assembly, chromosome: I	bacteria'
LT576315.1 Yarrowia lipolytica genome assembly, scaffold: YALIA101S01, whole genome shotgun sequence	fungi'
FLRD01001914.1 Plasmodium ovale wallikeri genome assembly, contig: contig1914, whole genome shotgun sequence	protozoa'
FLRE01000165.1 Plasmodium ovale wallikeri genome assembly, contig: contig165, whole genome shotgun sequence	protozoa'
FLQV01000125.1 Plasmodium ovale curtisi genome assembly, contig: contig125, whole genome shotgun sequence	protozoa'
FLQU01000196.1 Plasmodium ovale curtisi genome assembly, contig: contig196, whole genome shotgun sequence	protozoa'
FLQW01001298.1 Plasmodium malariae genome assembly, contig: contig1298, whole genome shotgun sequence	protozoa'
LT594323.1 Micromonospora auratinigra strain DSM 44815 genome assembly, chromosome: I	bacteria'
LT594324.1 Micromonospora narathiwatensis strain DSM 45248 genome assembly, chromosome: I	bacteria'
LT598496.1 Micromonospora krabiensis strain DSM 45344 genome assembly, chromosome: I	bacteria'
FLSR01004883.1 Leuciscus waleckii genome assembly, contig: LG4, whole genome shotgun sequence	vertebrates_non_mammals'
LT599501.1 Cladophialophora bantiana genome assembly, scaffold: UM956_scaffold3, whole genome shotgun sequence	fungi'
FMJQ01000001.1 Heligmosomoides polygyrus bakeri genome assembly, contig: nHp.2.0.scaf00001, whole genome shotgun sequence	invertebrates'
LT615372.1 Escherichia coli isolate 102 genome assembly, chromosome: I	bacteria'
LT615375.1 Escherichia coli isolate 107 genome assembly, chromosome: I	bacteria'
LT615373.1 Escherichia coli isolate 105 genome assembly, chromosome: I	bacteria'
LT615374.1 Escherichia coli isolate 104 genome assembly, chromosome: I	bacteria'
LT615376.1 Escherichia coli isolate 109 genome assembly, chromosome: I	bacteria'
LT615379.1 Escherichia coli isolate 108 genome assembly, chromosome: I	bacteria'
LT615371.1 Escherichia coli isolate 101 genome assembly, chromosome: I	bacteria'
LT629695.1 Agrococcus jejuensis strain DSM 22002 genome assembly, chromosome: I	bacteria'
LT629694.1 Leifsonia sp. 197AMF genome assembly, chromosome: I	bacteria'
LT629693.1 Bradyrhizobium ottawaense strain GAS524 genome assembly, chromosome: I	bacteria'
LT629696.1 Leifsonia sp. 466MF genome assembly, chromosome: I	bacteria'
LT629697.1 Frankineae bacterium MT45 genome assembly, chromosome: I	bacteria'
LT629698.1 Leifsonia sp. 157MF genome assembly, chromosome: I	bacteria'
LT627734.1 Candidatus Aquiluna sp. UB-MaderosW2red genome assembly, chromosome: I	bacteria'
LT629692.1 Microbacterium pygmaeum strain DSM 23142 genome assembly, chromosome: I	bacteria'
LT629699.1 Pseudomonas libanensis strain BS2975 genome assembly, chromosome: I	bacteria'
LT629687.1 Pseudomonas koreensis strain BS3658 genome assembly, chromosome: I	bacteria'
FMVU01000051.1 Ustilago bromivora strain UB2 genome assembly, contig: scaffold51, whole genome shotgun sequence	fungi'
LT627735.1 Nonlabens sp. Hel1_33_55 genome assembly, chromosome: I	bacteria'
LT629688.1 Auraticoccus monumenti strain MON 2.2 genome assembly, chromosome: I	bacteria'
LT627736.1 Microbacterium sp. LKL04 genome assembly, chromosome: I	bacteria'
LT629689.1 Pseudomonas extremaustralis strain DSM 17835 genome assembly, chromosome: I	bacteria'
LT629690.1 Terriglobus roseus strain GAS232 genome assembly, chromosome: I	bacteria'
LT629691.1 Pseudomonas thivervalensis strain BS3779 genome assembly, chromosome: I	bacteria'
LT629712.1 Bifidobacterium longum strain Su859 genome assembly, chromosome: I	bacteria'
LT629713.1 Pseudomonas brassicacearum strain BS3663 genome assembly, chromosome: I	bacteria'
LT629702.1 Pseudomonas azotoformans strain LMG 21611 genome assembly, chromosome: I	bacteria'
LT629703.1 Afipia sp. GAS231 genome assembly, chromosome: I	bacteria'
LT629700.1 Corynebacterium mycetoides strain DSM 20632 genome assembly, chromosome: I	bacteria'
LT629701.1 Allokutzneria albata strain DSM 44149 genome assembly, chromosome: I	bacteria'
LT629704.1 Pseudomonas antarctica strain BS2772 genome assembly, chromosome: I	bacteria'
LT629705.1 Pseudomonas arsenicoxydans strain CECT 7543 genome assembly, chromosome: I	bacteria'
LT629707.1 Leifsonia sp. 509MF genome assembly, chromosome: I	bacteria'
LT629706.1 Pseudomonas poae strain BS2776 genome assembly, chromosome: I	bacteria'
LT629709.1 Pseudomonas reinekei strain BS3776 genome assembly, chromosome: I	bacteria'
LT629708.1 Pseudomonas extremorientalis strain BS2774 genome assembly, chromosome: I	bacteria'
LT629302.1 Flavobacteriaceae bacterium MAR_2010_188 genome assembly, chromosome: I	bacteria'
LT629711.1 Phycicoccus dokdonensis strain DSM 22329 genome assembly, chromosome: I	bacteria'
LT629710.1 Nakamurella panacisegetis strain P4-7,KCTC 19426,CECT 7604 genome assembly, chromosome: I	bacteria'
LT629755.1 Agromyces flavus strain CPCC 202695 genome assembly, chromosome: I	bacteria'
LT629734.1 Agrococcus carbonis strain DSM 22965 genome assembly, chromosome: I	bacteria'
LT629738.1 Pseudomonas chlororaphis strain ATCC 13985 genome assembly, chromosome: I	bacteria'
LT629758.1 Actinoplanes derwentensis strain DSM 43941 genome assembly, chromosome: I	bacteria'
LT629746.1 Pseudomonas lini strain BS3782 genome assembly, chromosome: I	bacteria'
LT629737.1 Gillisia sp. Hel1_33_143 genome assembly, chromosome: I	bacteria'
LT629733.1 Formosa sp. Hel1_31_208 genome assembly, chromosome: I	bacteria'
LT629751.1 Pseudomonas oryzae strain KCTC 32247 genome assembly, chromosome: I	bacteria'
LT629760.1 Pseudomonas trivialis strain BS3111 genome assembly, chromosome: I	bacteria'
LT629742.1 Microterricola viridarii strain DSM 21772 genome assembly, chromosome: I	bacteria'
LT629753.1 Pseudomonas cedrina strain BS2981 genome assembly, chromosome: I	bacteria'
LT629735.1 Opitutus sp. GAS368 genome assembly, chromosome: I	bacteria'
LT629768.1 Streptomyces sp. 2114.2 genome assembly, chromosome: I	bacteria'
LT629736.1 Pseudomonas xinjiangensis strain NRRL B-51270 genome assembly, chromosome: I	bacteria'
LT629757.1 Marmoricola scoriae strain DSM 22127 genome assembly, chromosome: I	bacteria'
LT629756.1 Pseudomonas sp. Z003-0.4C(8344-21) genome assembly, chromosome: I	bacteria'
LT629747.1 Pseudomonas chlororaphis strain LMG 21630 genome assembly, chromosome: I	bacteria'
LT629761.1 Pseudomonas chlororaphis strain DSM 21509 genome assembly, chromosome: I	bacteria'
LT629748.1 Pseudomonas litoralis strain 2SM5 genome assembly, chromosome: I	bacteria'
LT629759.1 Olsenella umbonata strain DSM 22620 genome assembly, chromosome: I	bacteria'
LT629745.1 Gramella sp. MAR_2010_102 genome assembly, chromosome: I	bacteria'
LT629739.1 Brevibacterium sandarakinum strain DSM 22082 genome assembly, chromosome: I	bacteria'
LT629749.1 Friedmanniella luteola strain DSM 21741 genome assembly, chromosome: I	bacteria'
LT629744.1 Pseudomonas sp. bs2935 genome assembly, chromosome: I	bacteria'
LT629743.1 Erythrobacter sp. HL-111 genome assembly, chromosome: I	bacteria'
LT629754.1 Maribacter sp. MAR_2009_60 genome assembly, chromosome: I	bacteria'
LT629750.1 Bradyrhizobium canariense strain GAS369 genome assembly, chromosome: I	bacteria'
LT629741.1 Gramella sp. MAR_2010_147 genome assembly, chromosome: I	bacteria'
LT629752.1 Polaribacter sp. KT25b genome assembly, chromosome: I	bacteria'
LT629762.1 Pseudomonas prosekii strain LMG 26867 genome assembly, chromosome: I	bacteria'

LT629740.1 Mucilaginibacter mallensis strain MP1X4 genome assembly, chromosome: I	bacteria'
LT629771.1 Jiangella sp. DSM 45060 genome assembly, chromosome: I	bacteria'
LT629764.1 Paenibacillaceae bacterium GAS479 genome assembly, chromosome: I	bacteria'
LT629763.1 Pseudomonas sabulinigri strain JCM 14963 genome assembly, chromosome: I	bacteria'
LT629767.1 Pseudomonas umsongensis strain BS3657 genome assembly, chromosome: I	bacteria'
LT629769.1 Pseudomonas syringae strain 31R1 genome assembly, chromosome: I	bacteria'
LT629765.1 Corynebacterium timonense strain DSM 45434 genome assembly, chromosome: I	bacteria'
LT629766.1 Brevibacterium siliguriense strain DSM 23676 genome assembly, chromosome: I	bacteria'
LT629770.1 Microbacterium paraoxydans strain DSM 15019 genome assembly, chromosome: I	bacteria'
LT629772.1 Microlunatus soli strain DSM 21800 genome assembly, chromosome: I	bacteria'
LT629775.1 Streptomyces sp. TLI_053 genome assembly, chromosome: I	bacteria'
LT629773.1 Gardnerella vaginalis strain DSM 4944 genome assembly, chromosome: I	bacteria'
LT629774.1 Winogradskyella sp. RHA_55 genome assembly, chromosome: I	bacteria'
LT629776.1 Paraoerskovia marina strain DSM 22126 genome assembly, chromosome: I	bacteria'
LT629777.1 Pseudomonas asplenii strain ATCC 23835 genome assembly, chromosome: I	bacteria'
LT629778.1 Pseudomonas granadensis strain LMG 27940 genome assembly, chromosome: I	bacteria'
LT629779.1 Pseudarthrobacter equi strain IMMIB L-1606 genome assembly, chromosome: I	bacteria'
LT629801.1 Pseudomonas rhodesiae strain BS2777 genome assembly, chromosome: I	bacteria'
LT629784.1 Stappia sp. ES.058 genome assembly, chromosome: I	bacteria'
LT630003.1 [Clostridium] sphenoides JCM 1415 strain ATCC 19403 genome assembly, chromosome: I	bacteria'
LT629787.1 Pseudomonas salegens strain CECT 8338 genome assembly, chromosome: I	bacteria'
LT629786.1 Pseudomonas synxantha strain LMG 2190 genome assembly, chromosome: I	bacteria'
LT629781.1 Verrucomicrobiaceae bacterium GAS474 genome assembly, chromosome: I	bacteria'
LT629793.1 Pseudomonas yamanorum strain LMG 27247 genome assembly, chromosome: I	bacteria'
LT629799.1 Friedmanniella sagamiharensis strain DSM 21743 genome assembly, chromosome: I	bacteria'
LT629782.1 Pseudomonas orientalis strain BS2775 genome assembly, chromosome: I	bacteria'
LT629788.1 Pseudomonas moraviensis strain BS3668 genome assembly, chromosome: I	bacteria'
LT629800.1 Pseudomonas brenneri strain BS2771 genome assembly, chromosome: I	bacteria'
LT629803.1 Pseudomonas vancouverensis strain BS3656 genome assembly, chromosome: I	bacteria'
LT629783.1 Pseudomonas fragi strain NRRL B-727 genome assembly, chromosome: I	bacteria'
LT629789.1 Amycolatopsis keratiniphila strain FH 1893 genome assembly, chromosome: I	bacteria'
LT629804.1 Arcanobacterium phocae strain DSM 10002 genome assembly, chromosome: I	bacteria'
LT629780.1 Pseudomonas guangdongensis strain CCTCC 2012022 genome assembly, chromosome: I	bacteria'
LT629791.1 Jiangella alkaliphila strain DSM 45079 genome assembly, chromosome: I	bacteria'
LT629785.1 Pseudomonas pohangensis strain DSM 17875 genome assembly, chromosome: I	bacteria'
LT629790.1 Pseudomonas mediterranea strain DSM 16733 genome assembly, chromosome: I	bacteria'
LT629797.1 Pseudomonas sihuiensis strain KCTC 32246 genome assembly, chromosome: I	bacteria'
LT629802.1 Pseudomonas mucidolens strain LMG 2223 genome assembly, chromosome: I	bacteria'
LT629792.1 Actinomyces radingae strain DSM 9169 genome assembly, chromosome: I	bacteria'
LT629796.1 Pseudomonas mandelii strain LMG 21607 genome assembly, chromosome: I	bacteria'
LT629794.1 Polaribacter sp. Hel1_33_78 genome assembly, chromosome: I	bacteria'
LT629798.1 Pseudomonas corrugata strain BS3649 genome assembly, chromosome: I	bacteria'
LT629795.1 Pseudomonas psychrophila strain BS3667 genome assembly, chromosome: I	bacteria'
LT635756.1 [Candida] intermedia strain CBS 141442 genome assembly, chromosome: I	fungi'
LT635764.1 [Candida] intermedia strain PYCC 4715 genome assembly, chromosome: I	fungi'
LT629867.1 Leifsonia sp. 21MFCrub1.1 genome assembly, chromosome: I	bacteria'
LT629970.1 Pseudomonas xanthomarina strain LMG 23572 genome assembly, chromosome: I	bacteria'
LT629971.1 Mycobacterium rutilum strain DSM 45405 genome assembly, chromosome: I	bacteria'
LT629972.1 Pseudomonas fuscovaginae strain LMG 2158 genome assembly, chromosome: I	bacteria'
FNXT01001221.1 Tetradesmus obliquus genome assembly, contig: scaf1221, whole genome shotgun sequence	plants'
FNXX01000001.1 Conringia planisiliqua genome assembly, contig: scaffold_1, whole genome shotgun sequence	plants'
LT630002.1 Leifsonia sp. 467MF genome assembly, chromosome: I	bacteria'
LT630032.1 Leifsonia sp. 98AMF genome assembly, chromosome: I	bacteria'
FPAK01000001.1 Euclidium syriacum genome assembly, contig: scaffold_1, whole genome shotgun sequence	plants'
FQNF01000001.1 Hanseniaspora guilliermondii, strain UTAD222, genome assembly, contig: HGUI_contig_001, whole genome shotgun sequence	fungi'
LT634571.1 Sus scrofa genome assembly, chromosome: X	vertebrates_mammals'
LT632320.1 Escherichia coli strain NCTC 13441 genome assembly, chromosome: 1	bacteria'
LT649234.1 Stramenopiles sp. TOSAG23-2 genome assembly, scaffold: TOSAG23-2_scaffold_1, whole genome shotgun sequence	protozoa'
LT635910.1 Stramenopiles sp. TOSAG23-6 genome assembly, scaffold: TOSAG23-6_scaffold_1, whole genome shotgun sequence	protozoa'
LT658380.1 Stramenopiles sp. TOSAG41-1 genome assembly, scaffold: TOSAG41-1_scaffold_1, whole genome shotgun sequence	protozoa'
LT642146.1 Stramenopiles sp. TOSAG23-3 genome assembly, scaffold: TOSAG23-3_scaffold_1, whole genome shotgun sequence	protozoa'
LT667505.1 Bathycoccus sp. TOSAG39-1 genome assembly, scaffold: TOSAG39-1_scaffold_1, whole genome shotgun sequence	plants'
FQTN01001675.1 Branchiostoma belcheri isolate BF01 genome assembly, contig: scaffold1, whole genome shotgun sequence	invertebrates'
LT670849.1 Bradyrhizobium erythrophlei strain GAS401 genome assembly, chromosome: I	bacteria'
LT671675.1 Olsenella sp. Marseille-P3237 strain Marseille-P3237T genome assembly, chromosome: scaffold00001	bacteria'
FTPI01000002.1 Fraxinus excelsior genome assembly, contig: Contig1, whole genome shotgun sequence	plants'
LT671813.1 Malassezia sympodialis isolate KS292 genome assembly, chromosome: 1	fungi'
LT671821.1 Malassezia sympodialis ATCC 42132 genome assembly, chromosome: 1	fungi'
LT671789.1 Malassezia sympodialis isolate KS024 genome assembly, chromosome: 1	fungi'
LT671798.1 Malassezia sympodialis isolate KS327 genome assembly, chromosome: 2	fungi'
LT671805.1 Malassezia sympodialis isolate KS004 genome assembly, chromosome: 1	fungi'
LT707061.1 Pseudomonas putida strain N1R genome assembly, chromosome: I	bacteria'
LT707063.1 Pseudomonas sp. B10 genome assembly, chromosome: I	bacteria'
LT707062.1 Pseudomonas sp. A214 genome assembly, chromosome: I	bacteria'
LT707064.1 Pseudomonas sp. 7SR1 genome assembly, chromosome: I	bacteria'
LT707065.1 Achromobacter sp. MFA1 R4 genome assembly, chromosome: I	bacteria'
